# NGS-based approach to determine the presence of HPV and their sites of integration in human cancer genome

**DOI:** 10.1038/bjc.2015.121

**Published:** 2015-05-14

**Authors:** P Chandrani, V Kulkarni, P Iyer, P Upadhyay, R Chaubal, P Das, R Mulherkar, R Singh, A Dutt

**Affiliations:** 1 Advanced Centre for Treatment, Research and Education in Cancer, Tata Memorial Centre, Kharghar, Navi Mumbai, Maharashtra 410210, India

**Keywords:** HPV detection, human cancer, next-generation sequencing (NGS)

## Abstract

**Background:**

Human papilloma virus (HPV) accounts for the most common cause of all virus-associated human cancers. Here, we describe the first graphic user interface (GUI)-based automated tool ‘HPVDetector', for non-computational biologists, exclusively for detection and annotation of the HPV genome based on next-generation sequencing data sets.

**Methods:**

We developed a custom-made reference genome that comprises of human chromosomes along with annotated genome of 143 HPV types as pseudochromosomes. The tool runs on a dual mode as defined by the user: a ‘quick mode' to identify presence of HPV types and an ‘integration mode' to determine genomic location for the site of integration. The input data can be a paired-end whole-exome, whole-genome or whole-transcriptome data set. The HPVDetector is available in public domain for download: http://www.actrec.gov.in/pi-webpages/AmitDutt/HPVdetector/HPVDetector.html.

**Results:**

On the basis of our evaluation of 116 whole-exome, 23 whole-transcriptome and 2 whole-genome data, we were able to identify presence of HPV in 20 exomes and 4 transcriptomes of cervical and head and neck cancer tumour samples. Using the inbuilt annotation module of HPVDetector, we found predominant integration of viral gene *E7*, a known oncogene, at known 17q21, 3q27, 7q35, Xq28 and novel sites of integration in the human genome. Furthermore, co-infection with high-risk HPVs such as 16 and 31 were found to be mutually exclusive compared with low-risk HPV71.

**Conclusions:**

HPVDetector is a simple yet precise and robust tool for detecting HPV from tumour samples using variety of next-generation sequencing platforms including whole genome, whole exome and transcriptome. Two different modes (quick detection and integration mode) along with a GUI widen the usability of HPVDetector for biologists and clinicians with minimal computational knowledge.

Human papilloma viral (HPV) infections has been associated with various types of cancer. Epidemiological studies indicate that about 90% of cervical cancers, 90–93% of anal canal cancers, 12–63% of oropharyngeal cancers, 36–40% of penile cancers, 40–64% of vaginal cancers and 40–51% of vulvar cancers are attributable to HPV infection (Munoz *et al*, 2003; Shukla, 2009). Currently, HPV detections are primarily carried out using PCR-based MY09/11 and CPI/II systems (Kleter *et al*, 1998). Other techniques used include the hybridisation-based SPF LiPA method, signal-amplification assays (Hybrid Capture 2 and Cervista) and nucleic-acid-based amplification-like microarray, real-time PCR-based methods (COBAS 4800 real-time test) (Kleter *et al*, 1998; Brink *et al*, 2007; Abreu *et al*, 2012). These technologies come with limitations to detect minor, low-abundance HPV genotypes and a complex mixture of co-infections that can be a negative determinant of the clinical outcome (Mendez *et al*, 2005; Trottier *et al*, 2006). Next-generation sequencing (NGS) technologies overcomes such limitations, as evident from the recently described high-risk HPV genotyping assay for primary cervical cancer screening based on self-collection (Yi *et al*, 2014), using TEN16 or HIVID methodology, and to determine co-infection among the HPV types probed along with their sites on intregration (Johansson *et al*, 2013; Xu *et al*, 2013; Li *et al*, 2013b; Ameur *et al*, 2014; Hu *et al*, 2015). However, there is an unmet need for a simplified tool for biologists with no previous experience or knowledge of informatics to analyse the data generated by whole-exome, transcriptome or genome sequencing using NGS technology to detect the presence of HPV sequences along with their integration sites. There are a variety of gene integration finding tools available that can detect different pathogen insertions in the human genome such as ViralFusionSeq (Li *et al*, 2013a), VirusSeq (Chen *et al*, 2013), VirusFinder (Wang *et al*, 2013), Path-Seq (Kostic *et al*, 2011), RINS (Bhaduri *et al*, 2012), and ReadSCAN (Naeem *et al*, 2013). These tools have their specific third-party needs, and are not specific for HPV detection. They can detect presence of a HPV sequence along with other viruses, but lack information to annotate the region of the HPV genome detected. Here, we describe ‘HPVDetector' as a specific *in silico* automated tool that is capable of multi-HPV type detection, their annotation and determination of site of HPV integration utilising raw exome, transcriptome, or whole-genome data as input with minimal requirement for third-party tools.

## Materials and methods

**Figure 1:**
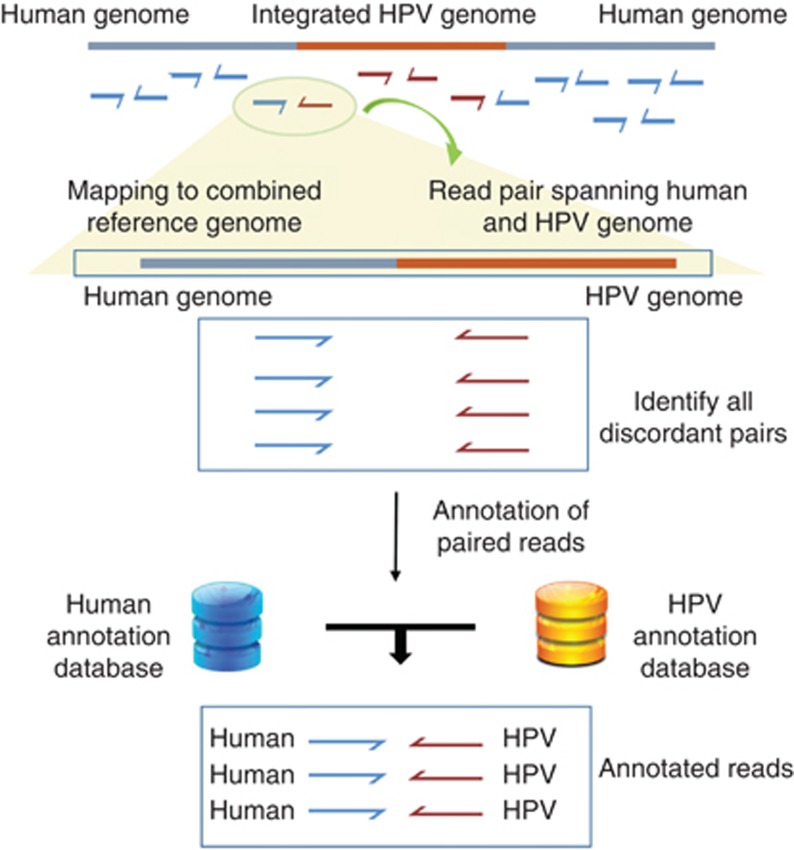
**Conceptual workflow of the HPVDetector.** The flowchart represents workflow for HPVDetector. Paired-end reads obtained from next-generation sequencing data are aligned to a combined Human–HPV reference database. All discordant read pairs with one read aligning to human and other to the HPV genome are identified and annotated utilising human and HPV database using an inbuilt annotator module.

HPV detection involves a computational subtraction-based approach, where NGS data are used for alignment against custom-made HPV multi-reference genome sequences to detect the traces of multiple HPV types using an automated pipeline (Figure 1).

### HPV reference sequences and annotation

As a first step of the pipeline, HPV genomes in fasta format is required. We have acquired GenBank (.gb) files of 143 types of HPVs from a web resource Papillomavirus Episteme (PAVE) (Van Doorslaer *et al*, 2013). We converted these GenBank (.gb) files into fasta files. All these reference sequences were concatenated to compose a multi-fasta sequence using bio-perl modules (Stajich *et al*, 2002). Apart from this, we also parsed the GenBank (.gb) files to generate a HPV gene reference having nucleotide intervals for each gene of each HPV type. This gene reference file was used to annotate the HPV gene.

### HPV type and HPV-aligned reads detection

Evaluating the HPV type and HPV-aligned reads is crucial to find HPV in the respective sample. For HPV type detection, we indexed the multi-fasta HPV reference file using BWA aligner followed by alignment of reads to indexed genome (Li and Durbin, 2009). The aligned reads were extracted from the same file using a utility ViewSam from Picard Tools package (http://broadinstitute.github.io/picard/). The alignment files were parsed using UNIX shell program to detect the type of HPV as well as number of reads that align to a particular HPV type. Number of HPV reads were normalised to the total depth of coverage per sample and with respect to different HPV gene sizes.

### Assessment of specificity and sensitivity of HPVDetector

We downloaded SiHa whole-genome sequence from Sequence Read Archive database of DDBJ (https://trace.ddbj.nig.ac.jp/DRASearch/; study: SRP048769). The data were converted from SRA to FASTQ using SRAtoolkit. The resulting FASTQ files represents >36 × genome coverage which was further downsampled to 1 × , 2 × , 3 × , 4 × , 5 × , 10 × , 15 × , 20 × , 25 × and 30 × using Picard Toolkit's DownsampleSam function (http://broadinstitute.github.io/picard/). The resulting FASTQ files were used for testing HPV detection using HPVDetector.

### Human–HPV integration loci detection

To detect integration sites, we created a custom reference genome comprised of human chromosomes and HPV fasta sequences as pseudochromosomes. HPV genomes were appended to human chromosomes to compose a multi-fasta reference genome. This custom Human–HPV reference genome was then used for aligning reads with short-read aligner BWA. The alignment files were parsed for the reads where one mate is aligned to human chromosome and another to HPV. The Human chromosomal positions, HPV type and HPV reference position were parsed and annotated with a gene reference annotation file acquired from UCSC table browser (Karolchik *et al*, 2004) to get a list of integration sites.

### RNA extraction, cDNA synthesis and E6-specific PCR

Total RNA extraction was performed from primary tumours and cell lines using Trizol reagent (Invitrogen, Grand Island, NY, USA) as per the manufacture's instruction and later resolved on 1.2% agarose gel to confirm the RNA integrity. DNase treatment was done using the DNase Free kit (Ambion, Foster City, CA, USA; cat AM1906) followed by first-strand cDNA synthesis taking 2 *μ*g of total RNA using Superscript III kit (Invitrogen, 18080-051). E6 (HPV-16) and GAPDH expression were checked as described previously (Smeets *et al*, 2007).

### HPV detection using MY09/11 and PCR primers

MY09/11 primer sequences were taken from previously reported literature (Baay *et al*, 1996). All samples were screened by PCR first using MY09/11 primer. GAPDH was used as internal control for each sample. SiHa cell line (Adler *et al*, 1997) was used as a positive control for HPV and AW13516 cell line (Tatake *et al*, 1990) as a negative control. The PCR reaction was performed in 20 *μ*l volume containing 10 *μ*l (2 × ) Biomix-Red master mix (Biomix, Port Orange, FL, USA; cat Bio25005), 5 *μ*M each primer, 50 ng gDNA. PCR condition was: initial denaturation: 95 °C, denaturation: 94 °C for 1 min, annealing: 55 °C, (MY09/11, GAPDH), extension: 72 °C for 1 min and final extension at 72 °C for 5 min on a PCR machine (Veriti 96-Well Fast Thermal Cycler, Applied Biosystems, Carlsbad, CA, USA). Primers were designed to amplify 122-, 126- and 120-bp read sequences of HPV16 identified by HPVDetector in SiHa cell line. Primers flanking the human reads were designed to amplify 119 and 290 bp, respectively. These sequences were further validated by Sanger sequencing. All the experiments were repeated at least twice independently. The details of the primers used in the study are provided in Supplementary Table 2.

## Results

HPVDetector is a tool to quickly detect hundreds of HPV types from next-generation sequence data without any prerequisite knowledge about virus types. It runs on paired-end sequenced samples. It is composed of two modes or sub pipelines as quick detect and integration detect mode.

### Quick detect mode

This mode is to quickly determine the HPV type or types to check whether multiple HPV co-infections are existing or not in a given sample. Quick detect mode starts with alignment of raw paired-end sequencing reads against the custom-made multi-HPV genome using BWA aligner. Computational subtraction of the reads is then carried out, in which HPV-aligned reads are retained using Picard Tools and further processed using UNIX shell program to distinguish reads mapping to different HPV types. Finally, HPVDetector outputs a result file, which enlists one or more HPV type(s) and number of HPV reads.

### Integration detect mode

This mode of HPVDetector determines the genomic location of HPV integrant, annotate with HPV gene, human chromosomal loci and human gene. This mode of HPVDetector pipeline starts with alignment of raw reads against a custom-made reference including a pseudochromosome such as the multi-fasta reference genome containing 143 HPV reference sequences and the HG19 human reference genome. Computational subtraction was carried out to retain discordant read pairs where the sequences are aligned to both human as well as HPV genomes. Finally, HPVDetector outputs a result file, which enlists HPV integration loci on the human genome, annotation of HPV genes, human genes and human genome cytobands.

### Detection of HPV type integrated in the host genome

#### Cervical cancer exome sequencing data

**Figure 2:**
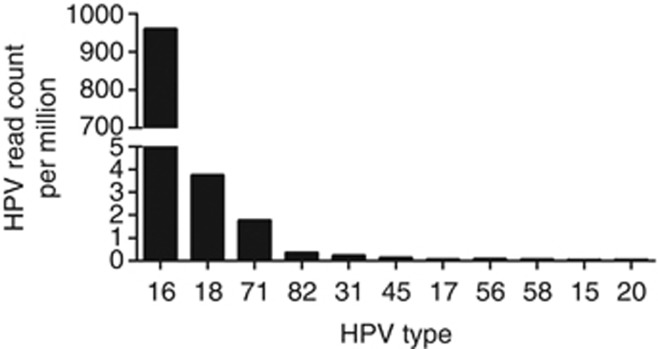
**Quantitative representation, by number of reads, of HPV types detected in cervical tumours.** The graph represents distribution of total number of HPV reads per million of total reads for all HPV types detected across 17 cervical samples. HPV16 has the highest number of reads across 17 samples followed by HPV(18, 71, 45, 31, 82, 17, 56, 58, 15, 20) in the decreasing order of their read counts.

**Figure 3:**
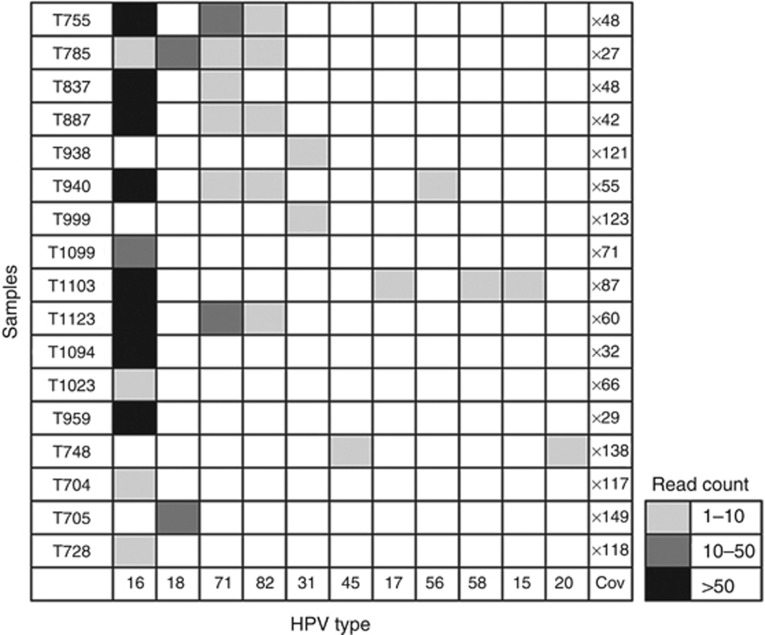
**HPV gene integration frequency across different cervical cancer samples.** Heatmap representation of HPV types detected across 17 cervical cancer samples. HPV16 in 13 samples, HPV18 in 2 samples, HPV71 in 6 samples, HPV82 in 5 samples, HPV31 in 2 samples, HPV45 in 5 samples, and HPV17, 56, 58, 15, 20 were detected in 1 sample, each. Total coverage of the exome sequencing is indicated in the last column ‘cov'. On the basis of read count, the abundance of HPV is graded in different samples: read counts 1–10 as light grey; read counts 10–50 as dark grey; and read counts >50 as black.

We analysed 22 cervical cancer exome sequencing data (generated in-house at ACTREC, unpublished data) to detect the presence of HPV. Among the 22 samples analysed, HPV was detected in 18 cervical samples, with maximum number of reads supporting the HPV16 sequence (Figure 2) (Das *et al*, 2012). We also detected the presence of additional HPV types such as HPV71 (in six samples), HPV82 (in five samples) and HPV31 (in two samples) with variable number of supporting reads as shown in (Figure 3). Co-infection with more than one HPV type is known to be associated with significantly increased risk of cervical intraepithelial neoplasia 2+ and found in 43.2% of HPV-positive women (Liaw *et al*, 2001; Mendez *et al*, 2005; Vaccarella *et al*, 2010; Chaturvedi *et al*, 2011). Six of 22 cervical cancer patients (43%) were found to be co-infected with one or more HPV subtypes in this study using HPVDetector (Figure 3). Interesting to note, based on phylogenetic analysis of HPV types, HPV16 and HPV31 of the virulent alpha 7 group infection occurred in a mutually exclusive manner (in 13 of 22 samples), whereas HPV71 of the alpha 15 subgroup, known to be involved in commensal infections that infected 6 of 14 cervical tumour samples, invariably co-occurred with other HPV subtypes (Schiffman *et al*, 2009; Harari *et al*, 2014). The HPV sequence detected in primary cervical tumour sample were independently validated by directed sequencing in T1094, the only sample with sufficient quality DNA (as shown in Supplementary Figure 1).

#### Tongue squamous cell carcinoma exome and transcriptome data

HPV is an independent risk factor in head and neck squamous cell carcinoma (HNSCC), in particular for oral and oropharyngeal carcinomas (Chaudhary *et al*, 2009; Pannone *et al*, 2011). We analysed whole-exome data from 23 paired and one orphan tongue squamous cell carcinoma (TSCC) sample and 7 HNSCC cell lines (generated in-house at ACTREC, unpublished data). None of the TSCC primary tumours were found to be HPV positive, as reported earlier (Siebers *et al*, 2008; Patel *et al*, 2014; Tsimplaki *et al*, 2014). The absence of HPV infection were further validated by PCR using MY09/11 and E6 on genomic DNA and cDNA, respectively, suggesting a low false-negative feature of the HPVDetector primers (Supplementary Figure 2). At the same time, among the cell lines, NT8e cells (Mulherkar *et al*, 1997) of seven cell lines analysed was found to be positive for HPV71. Next, we analysed whole-transcriptome data of 17 TSCC and 6 TSCC cell lines (generated in-house at ACTREC, unpublished data) using the HPVDetector. Three of 17 primary tumours were found to be HPV18 positive. In addition, HPV18 reads were found in HEP2 cell line, consistent with earlier reports in literature (Ogura *et al*, 1993). The HPV18 genes (E1, E6, and E7) were validated in Hep2 cell line by PCR and Sanger sequencing (as shown in Supplementary Figure 1 and Table 2).

#### Gall bladder and liposarcoma exome and whole-genome data

We analysed 13 gall bladder cancer whole-exome, 1 gall bladder cancer whole-transcriptome and 1 liposarcoma whole-genome sequence data (generated in-house at ACTREC, unpublished data). No trace of the HPV sequence was detected in these samples.

### Assessment of specificity and sensitivity of HPVDetector

SiHa cell line developed from a cervical squamous cell carcinoma patient represents single-copy integration of HPV16 (el Awady *et al*, 1987) We analysed SiHa whole-genome sequence using HPVDetector. Consistent with a published report (Hu *et al*, 2015), HPVDetector could detect integration at chr13 intragenic location of KLF5—KLF12 genes and other regions (Supplementary Table 1). The integration was validated by PCR followed by sequencing (Supplementary Figure 3).

#### Sesnsitivity

**Figure 4:**
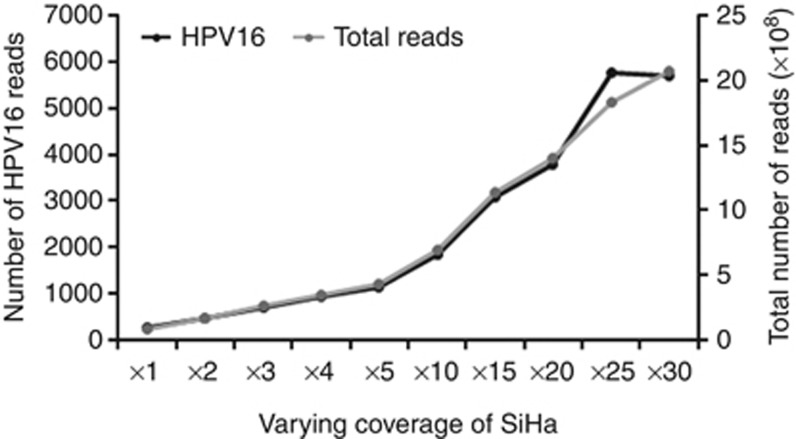
**Sensitivity of HPVDetector as a function of increasing genome sequence coverage.** SiHa WGS data were downsampled to the coverage of 1 × , 2 × , 3 × , 4 × , 5 × , 10 × , 15 × , 20 × , 25 × , and 30 × . HPV16 reads (black) were counted using HPVDetector at varying coverage and plotted along with total number of reads (grey).

To determine the sensitivity of HPVDetector, we downsampled the SiHa genome using a ‘downsampling' method, a Picard Toolkit's DownsampleSam function (http://broadinstitute.github.io/picard/) (Meynert *et al*, 2014) to generate varying coverage of the SiHa whole-genome data ranging from 1 × to 30 × coverage, and analysed using HPVDetector. Reads supporting presence of HPV reads linearly increased as a function of increasing coverage from 1 × to 25 × coverage. Beyond 25 × , no significant increase in HPV reads were found, suggesting saturation of genome coverage (Figure 4). In addition, among primary tumours, two pairs of HPV56 reads detected by the HPVDetector in T9440 as described in Supplementary Table 1 were validated earlier by Luminex array and SPF1/2 (Das *et al*, 2012). Taken together, this suggests HPVDetector could detect reads with as low as 1 × genome coverage with reads supported by as low as just two paired reads.

#### Specificity

Having benchmarked the HPVDetector against SiHa for sensitivity, next we tweaked the SiHa whole-genome sequence data to test specificity of the tool by taking reverse (not complement) of the SiHa genome to simulate as a random sequence but retaining composition of nucleotides and genome complexity, using an in-house perl script. We found no spurious HPV reads when the SiHa whole-genome sequence was reversed, suggesting the HPVDetector is specific to detect true HPV traces. Further, to address the issue of specificity among primary tumours, we performed another round of functional validation on tongue squamous tumours that were found HPV negative based on HPVDetector (Supplementary Table 1) and validated by My09/11 primers using genomic DNA. We analysed the expression of HPV E6 (Supplementary Figure 2b) in these samples. All samples were found negative for HPV presence. This suggests that the tool has low false negative.

### Annotation of the HPV genome integrated in the host genome

**Figure 5:**
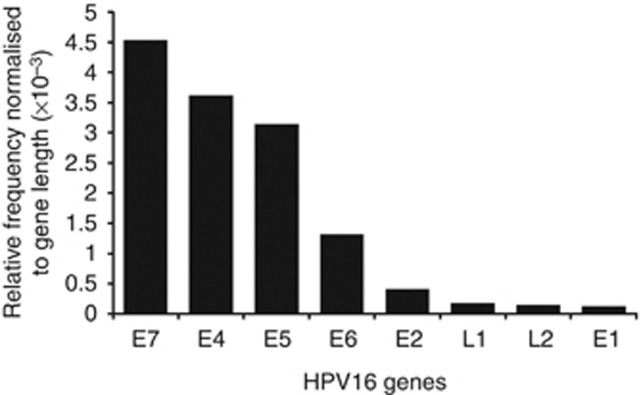
**Relative frequency of integration of HPV genes in cervical carcinoma.** HPV16 reads were annotated using an inbuilt annotation module of the HPVDetector to identify the viral genes. Number of reads per viral gene were normalised to the gene length, and frequency reads for individual genes are plotted, as shown.

To enable accurate gene annotation of the HPV genome sequenced, we prepared a gene annotation database of 143 HPV types from PAVE database (Van Doorslaer *et al*, 2013). Thirty-two reads of viral ORFs were found in 5 of 11 cervical tumours positive for HPV-16. Following the normalisation for the total number of reads against the length of individual genes, the viral gene *E7* was found to be predominantly represented among the cervical tumours infected with HPV16, followed by E4, E5 and E6, in decreasing order (Figure 5). Of these genes found to be enriched among all the integrants, it is interesting to note that the viral proteins E6 and E7 function as oncogenes by regulating the known human tumour suppressors, p53 and pRb, respectively (Lu *et al*, 2003; Yim and Park, 2005).

### Determination of the HPV integration sites in the host genome

**Figure 6:**
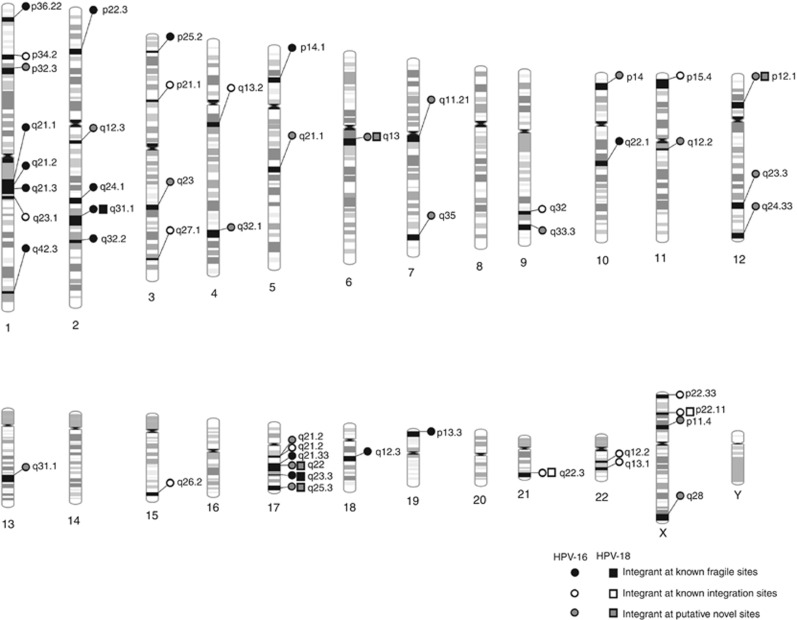
**Schematic representation of all HPV16 and 18 integration sites in the human genome detected across cervical cancer samples using HPVDetector.** Site of integration as determined by HPVDetector in cervical cancer samples is shown. HPV16 integration sites are depicted by circles and HPV18 by rectangles. Black, open and grey circles or rectangles represent integrations at known fragile sites, at known integration sites and at novel sites, respectively.

We identified 55 integration sites in 7 cervical cancer tumour samples T1099, T1123, T755, T887, T938, T1094, and T959 and 1 head and neck tumour sample using the HPVDetector (Supplementary Table 1). In this study, chromosomal loci 17q21, 3q27, 7q35, and Xq28 were observed with higher frequency compared with other loci for HPV integration, as reported earlier (Thorland *et al*, 2003). Interesting to note, we found HPV integration in the following fragile regions—(1p, 1q, 2p, 2q, 3p, 3q, 4p, 4q, 5p, 5q, 7q, 9q, 10q, 11p, 11q, 12p, 12q, 13q, 15q, 17q, 18q, 22q, Xp and Xq) that are prone to chromosome breaks to facilitate foreign DNA integration (Figure 6) (Smith *et al*, 2006). In T1123 and T755 HPV16 integration sites were detected at chr1q42.3 and chr3q23, respectively, identical to as reported earlier (Wentzensen *et al*, 2004; Schmitz *et al*, 2012). In addition, in T755 integration of HPV16 were found within the coding region at SLC25A36, a pyrimidine nucleotide carrier. This site of integration were also determined in T755 and T1123 samples using the APOT assay, as described earlier (Das *et al*, 2012) (Supplementary Table 1).

In total, we analysed 116 exome, 23 transcriptome and 2 whole-genome sequencing data, out of which we have detected presence of HPV in 20 exome and 4 transcriptome data (Table 1).

## Discussion

HPV accounts for the most common cause of all virus-associated human cancers. However, despite large-scale genome-wide DNA sequencing efforts of the cancer genome, there is no dedicated informatics tool to rapidly detect the presence of HPV in these genomes, in an exclusive manner. There are indeed a variety of gene integration finding tools available that can detect different pathogen insertions in the human genome, such as ViralFusionSeq, VirusSeq, VirusFinder, Path-Seq, RINS, and ReadSCAN. These sophisticated tools although have their specific third-party needs, necessitate intense computational infrastructure, cannot be run without specialised and advanced computational expertise of the researcher, and more importantly are not specific for HPV detection, *per se*—for example, lacks information to annotate the region of the HPV genome to predict the integrated viral gene, of which some are known to function as oncogenes.

We present a new user-friendly *in silico* tool ‘HPVDetector' as a unique tool to analyze NGS data to detect HPV sequences for non-computational biologists. Using the HPVDetector tool, we have detected 55 integration sites from the cervical exome and head and neck transcriptome data set. The tool allowed us to perform a comprehensive analysis to generate the information for co-occurrence of HPV subtypes across cervical cancer patients that is known to affect the clinical outcome of the disease. In addition, our finding of significant enrichment of viral gene *E7*>*E4*>*E5*>*E6* reads among the cervical tumour samples, using the inbuilt annotation module of the HPVDetector, is consistent with the known biology of HPV genes and their role in carcinogenesis, as *E6* and *E7* are known viral oncogenes. This unique feature of the HPVDetector with an inbuilt HPV annotation module could potentially be helpful to understand the function of other HPV ORFs with unknown function by studying their incidence against varying tumour stage and types. Although the analysis of cervical tumours were restricted to its exome data set, a complete spectrum of the load of viral genes present in a sample can similarly be determined using the whole-genome data as input to the HPVDetector.

HPVDetector demonstrate a low false-negative and false-positive rate that can detect HPV reads at as low as 1 × genome coverage. Reads supported by even two paired reads were found to be credible. No viral reads were detected across 54 head and neck primary tumour samples of Indian origin, as reported earlier (Siebers *et al*, 2008; Patel *et al*, 2014; Tsimplaki *et al*, 2014), but detected a low-risk HPV71 in a cell line that could be validated by performing MY09/11 PCR on the primary tumours as shown in Supplementary Figure 2. On the other hand, all the four HPV reads detected across different tumour types using HPVDetector could be validated by directed PCR followed by Sanger sequencing. One interesting utility of the HPVDetector would be to explore for HPV reads in NGS data from different cancer types. We analysed 13 gall bladder exome, 1 gall bladder transcriptome and 1 liposarcoma whole-genome sequencing data using HPVDetector. No HPV reads were found in these samples in this study.

Another critical feature of the HPVDetector is determination of HPV integration sites at the host genome. These integrations are known to occur at preferred regions of the genome (Thorland *et al*, 2003; Matovina *et al*, 2009). Using the integration site detection feature of the HPVDetector, we detected integration at various chromosomal locations (for e.g., 1p, 2p, 2q, 3p, 3q), some with significant overlap to the known fragile sites in literature and at several novel sites as summarised in Figure 6 and Supplementary Table 1. In summary, HPVDetector is a simple yet precise and robust tool for detecting HPV from tumour samples using variety of NGS platforms including whole genome, whole exome and transcriptome. Two different modes (quick detection and integration mode) along with a GUI widen the usability of HPVDetector for biologists with minimal computational knowledge (as described in the attached supplementary ‘HPVDetector User Guide' including Supplementary Figures 4 and 5).

## Figures and Tables

**Table 1:** Summary of HPV detection in all samples

**Study type**	**Samples tested**	**Presence of virus in samples**
Cervical cancer exome	36 (22 tumour, 14 paired normal)	18
SiHa cell line WGS	1	1
HNSCC cell line exome	7	1
TSCC exome	47 (24 tumour, 23 paired normal)	0
Gall bladder exome	26 (13 tumour, 13 paired normal)	1
TSCC transcriptome	17 (11 tumour, 6 paired normal)	3
HNSCC cell line transcriptome	5	1
Gall bladder transcriptome	1	0
Liposarcoma WGS	1	0
Total number of samples	141	25

Abbreviations: HNSCC=head and neck squamous cell carcinoma; HPV=human papilloma virus; TSCC=tongue squamous cell carcinoma; WGS=whole-genome sequencing.
