# Supplementary material for: NGS-based approach to determine the presence of HPV and their sites of integration in human cancer genome
Source: Br J Cancer. 2015 May 14;112(12):1958–65. doi: 10.1038/bjc.2015.121 (PMC4580395; doi:10.1038/bjc.2015.121)
Supplement: Supplementary Material 1 [file bjc2015121x1.doc]

**Annexure-III : additional information for reviewers only (not included in manuscript)**

[pratik@blri8w2017 ~]$ lsb_release -a

LSB Version: :base-4.0-amd64:base-4.0-noarch:core-4.0-amd64:core-4.0-noarch:graphics-4.0-amd64:graphics-4.0-noarch:printing-4.0-amd64:printing-4.0-noarch

Distributor ID: CentOS

Description: CentOS release 6.5 (Final)

Release: 6.5

Codename: Final

[pratik@blri8w2017 ~]$ ./HPVDetector config.txt /sapfs/actrec/users/pratik/down/siha/SRR1609142_1.fastq /sapfs/actrec/users/pratik/down/siha/SRR1609142_2.fastq WGS /sapfs/actrec/users/pratik/HPVDetector/siha_wgs >>log_siha_hpv.txt

Running HPVDetector:

Read 1 : SRR1609142_1.fastq

Read 2 : SRR1609142_2.fastq

sai 1 : SRR1609142_1.sai

sai 2 : SRR1609142_2.sai

sam : SRR1609142_1SRR1609142_2.sam

Setting Output Directory : /sapfs/actrec/users/pratik/HPVDetector/siha_wgs/

Actual Run Parameters

Path=/sapfs/actrec/ngs_tools/bwa-0.6.2/bwa

Mode=Integration

hpv index =/sapfs/actrec/users/pratik/HPVDetector/HPVDetector_v1.0/HPV_index_files/

hpv_human_bwa_index=/sapfs/actrec/users/pratik/HPVDetector/HPVDetector_v1.0/Human_HPV_index_files/

annotation dir=/sapfs/actrec/users/pratik/HPVDetector/HPVDetector_v1.0/annotation_files/

threads=50

picard=/sapfs/actrec/users/pratik/HPVDetector/HPVDetector_v1.0/picard-tools-1.100/

Read Type=WGS

Read Type : WGS

BWA : /sapfs/actrec/ngs_tools/bwa-0.6.2/bwa

Run Mode : Integration

HPV Index Base : /sapfs/actrec/users/pratik/HPVDetector/HPVDetector_v1.0/HPV_index_files/

HPV Human Index Base : /sapfs/actrec/users/pratik/HPVDetector/HPVDetector_v1.0/Human_HPV_index_files/

Threads : 50

Detection Mode=Integration

Alignment Initialised. Generating files in /sapfs/actrec/users/pratik/HPVDetector/siha_wgs/

[bwa_aln] 17bp reads: max_diff = 2

[bwa_aln] 38bp reads: max_diff = 3

[bwa_aln] 64bp reads: max_diff = 4

[bwa_aln] 93bp reads: max_diff = 5

[bwa_aln] 124bp reads: max_diff = 6

[bwa_aln] 157bp reads: max_diff = 7

[bwa_aln] 190bp reads: max_diff = 8

[bwa_aln] 225bp reads: max_diff = 9

[bwa_aln_core] calculate SA coordinate... 468.04 sec

[bwa_aln_core] write to the disk... 0.09 sec

[bwa_aln_core] 262144 sequences have been processed.

[bwa_aln_core] calculate SA coordinate... 416.83 sec

[bwa_aln_core] write to the disk... 0.10 sec

[bwa_aln_core] 524288 sequences have been processed.

[bwa_aln_core] calculate SA coordinate... 1182.86 sec

[bwa_aln_core] write to the disk... 0.10 sec

[bwa_aln_core] 786432 sequences have been processed.

[bwa_aln_core] calculate SA coordinate... 2467.55 sec

[bwa_aln_core] write to the disk... 0.11 sec

[bwa_aln_core] 1048576 sequences have been processed.

[bwa_aln_core] calculate SA coordinate... 2196.52 sec

[bwa_aln_core] write to the disk... 0.11 sec

[bwa_aln_core] 1310720 sequences have been processed.

[bwa_aln_core] calculate SA coordinate... 1385.29 sec

[bwa_aln_core] write to the disk... 0.11 sec

[bwa_aln_core] 1572864 sequences have been processed.

[bwa_aln_core] calculate SA coordinate... 1993.75 sec

[bwa_aln_core] write to the disk... 0.11 sec

[bwa_aln_core] 1835008 sequences have been processed.

[bwa_aln_core] calculate SA coordinate... 693.25 sec

[bwa_aln_core] write to the disk... 0.11 sec

[bwa_aln_core] 2097152 sequences have been processed.

[bwa_aln_core] calculate SA coordinate... 1596.55 sec

[bwa_aln_core] write to the disk... 0.11 sec

[bwa_aln_core] 2359296 sequences have been processed.

[bwa_aln_core] calculate SA coordinate... 2080.89 sec

[bwa_aln_core] write to the disk... 0.11 sec

[bwa_aln_core] 2621440 sequences have been processed.

[bwa_aln_core] calculate SA coordinate... 2569.97 sec

[bwa_aln_core] write to the disk... 0.11 sec

[bwa_aln_core] 2883584 sequences have been processed.

[bwa_aln_core] calculate SA coordinate... 1987.56 sec

[bwa_aln_core] write to the disk... 0.10 sec

[bwa_aln_core] 3145728 sequences have been processed.

[bwa_aln_core] calculate SA coordinate... 1500.36 sec

[bwa_aln_core] write to the disk... 0.11 sec

[bwa_aln_core] 3407872 sequences have been processed.

[bwa_aln_core] calculate SA coordinate... 2477.36 sec

[bwa_aln_core] write to the disk... 0.12 sec

[bwa_aln_core] 3670016 sequences have been processed.

[bwa_aln_core] calculate SA coordinate... 1644.33 sec

[bwa_aln_core] write to the disk... 0.10 sec

[bwa_aln_core] 3932160 sequences have been processed.

[bwa_aln_core] calculate SA coordinate... 2003.83 sec

[bwa_aln_core] write to the disk... 0.10 sec

[bwa_aln_core] 4194304 sequences have been processed.

[bwa_aln_core] calculate SA coordinate... 1599.48 sec

[bwa_aln_core] write to the disk... 0.10 sec

[bwa_aln_core] 4456448 sequences have been processed.

[bwa_aln_core] calculate SA coordinate... 1563.02 sec

[bwa_aln_core] write to the disk... 0.10 sec

[bwa_aln_core] 4718592 sequences have been processed.

[bwa_aln_core] calculate SA coordinate... 1828.80 sec

[bwa_aln_core] write to the disk... 0.10 sec

[bwa_aln_core] 4980736 sequences have been processed.

[bwa_aln_core] calculate SA coordinate... 2020.99 sec

[bwa_aln_core] write to the disk... 0.10 sec

[bwa_aln_core] 5242880 sequences have been processed.

[bwa_aln_core] calculate SA coordinate... 2050.21 sec

[bwa_aln_core] write to the disk... 0.11 sec

[bwa_aln_core] 5505024 sequences have been processed.

[bwa_aln_core] calculate SA coordinate... 1694.45 sec

[bwa_aln_core] write to the disk... 0.10 sec

[bwa_aln_core] 5767168 sequences have been processed.

[bwa_aln_core] calculate SA coordinate... 1651.28 sec

[bwa_aln_core] write to the disk... 0.11 sec

[bwa_aln_core] 6029312 sequences have been processed.

[bwa_aln_core] calculate SA coordinate... 1340.55 sec

[bwa_aln_core] write to the disk... 0.10 sec

[bwa_aln_core] 6291456 sequences have been processed.

[bwa_aln_core] calculate SA coordinate... 1278.34 sec

[bwa_aln_core] write to the disk... 0.11 sec

[bwa_aln_core] 6553600 sequences have been processed.

[bwa_aln_core] calculate SA coordinate... 2567.51 sec

[bwa_aln_core] write to the disk... 0.10 sec

[bwa_aln_core] 6815744 sequences have been processed.

[bwa_aln_core] calculate SA coordinate... 2188.96 sec

[bwa_aln_core] write to the disk... 0.10 sec

[bwa_aln_core] 7077888 sequences have been processed.

[bwa_aln_core] calculate SA coordinate... 1954.18 sec

[bwa_aln_core] write to the disk... 0.11 sec

[bwa_aln_core] 7340032 sequences have been processed.

[bwa_aln_core] calculate SA coordinate... 2080.02 sec

[bwa_aln_core] write to the disk... 0.11 sec

[bwa_aln_core] 7602176 sequences have been processed.

[bwa_aln_core] calculate SA coordinate... 1901.51 sec

[bwa_aln_core] write to the disk... 0.10 sec

[bwa_aln_core] 7864320 sequences have been processed.

[bwa_aln_core] calculate SA coordinate... 2043.69 sec

[bwa_aln_core] write to the disk... 0.10 sec

[bwa_aln_core] 8126464 sequences have been processed.

[bwa_aln_core] calculate SA coordinate... 1520.54 sec

[bwa_aln_core] write to the disk... 0.11 sec

[bwa_aln_core] 8388608 sequences have been processed.

[bwa_aln_core] calculate SA coordinate... 1773.43 sec

[bwa_aln_core] write to the disk... 0.11 sec

[bwa_aln_core] 8650752 sequences have been processed.

[bwa_aln_core] calculate SA coordinate... 1566.47 sec

[bwa_aln_core] write to the disk... 0.11 sec

[bwa_aln_core] 8912896 sequences have been processed.

[bwa_aln_core] calculate SA coordinate... 1346.12 sec

[bwa_aln_core] write to the disk... 0.11 sec

[bwa_aln_core] 9175040 sequences have been processed.

[bwa_aln_core] calculate SA coordinate... 2549.59 sec

[bwa_aln_core] write to the disk... 0.10 sec

[bwa_aln_core] 9437184 sequences have been processed.

[bwa_aln_core] calculate SA coordinate... 2245.40 sec

[bwa_aln_core] write to the disk... 0.10 sec

[bwa_aln_core] 9699328 sequences have been processed.

[bwa_aln_core] calculate SA coordinate... 2031.41 sec

[bwa_aln_core] write to the disk... 0.10 sec

[bwa_aln_core] 9961472 sequences have been processed.

[bwa_aln_core] calculate SA coordinate... 1825.80 sec

[bwa_aln_core] write to the disk... 0.10 sec

[bwa_aln_core] 10223616 sequences have been processed.

[bwa_aln_core] calculate SA coordinate... 876.62 sec

[bwa_aln_core] write to the disk... 0.10 sec

[bwa_aln_core] 10485760 sequences have been processed.

[bwa_aln_core] calculate SA coordinate... 1299.81 sec

[bwa_aln_core] write to the disk... 0.11 sec

[bwa_aln_core] 10747904 sequences have been processed.

[bwa_aln_core] calculate SA coordinate... 2020.87 sec

[bwa_aln_core] write to the disk... 0.10 sec

[bwa_aln_core] 11010048 sequences have been processed.

[bwa_aln_core] calculate SA coordinate... 1972.20 sec

[bwa_aln_core] write to the disk... 0.11 sec

[bwa_aln_core] 11272192 sequences have been processed.

[bwa_aln_core] calculate SA coordinate... 1389.05 sec

[bwa_aln_core] write to the disk... 0.09 sec

[bwa_aln_core] 11534336 sequences have been processed.

[bwa_aln_core] calculate SA coordinate... 1919.41 sec

[bwa_aln_core] write to the disk... 0.10 sec

[bwa_aln_core] 11796480 sequences have been processed.

[bwa_aln_core] calculate SA coordinate... 1620.61 sec

[bwa_aln_core] write to the disk... 0.10 sec

[bwa_aln_core] 12058624 sequences have been processed.

[bwa_aln_core] calculate SA coordinate... 1462.03 sec

[bwa_aln_core] write to the disk... 0.10 sec

[bwa_aln_core] 12320768 sequences have been processed.

[bwa_aln_core] calculate SA coordinate... 1557.83 sec

[bwa_aln_core] write to the disk... 0.10 sec

[bwa_aln_core] 12582912 sequences have been processed.

[bwa_aln_core] calculate SA coordinate... 1835.61 sec

[bwa_aln_core] write to the disk... 0.11 sec

[bwa_aln_core] 12845056 sequences have been processed.

[bwa_aln_core] calculate SA coordinate... 2097.36 sec

[bwa_aln_core] write to the disk... 0.11 sec

[bwa_aln_core] 13107200 sequences have been processed.

[bwa_aln_core] calculate SA coordinate... 1217.18 sec

[bwa_aln_core] write to the disk... 0.10 sec

[bwa_aln_core] 13369344 sequences have been processed.

[bwa_aln_core] calculate SA coordinate... 1157.78 sec

[bwa_aln_core] write to the disk... 0.10 sec

[bwa_aln_core] 13631488 sequences have been processed.

[bwa_aln_core] calculate SA coordinate... 2048.60 sec

[bwa_aln_core] write to the disk... 0.11 sec

[bwa_aln_core] 13893632 sequences have been processed.

[bwa_aln_core] calculate SA coordinate... 1915.12 sec

[bwa_aln_core] write to the disk... 0.11 sec

[bwa_aln_core] 14155776 sequences have been processed.

[bwa_aln_core] calculate SA coordinate... 1712.48 sec

[bwa_aln_core] write to the disk... 0.10 sec

[bwa_aln_core] 14417920 sequences have been processed.

[bwa_aln_core] calculate SA coordinate... 1459.38 sec

[bwa_aln_core] write to the disk... 0.10 sec

[bwa_aln_core] 14680064 sequences have been processed.

[bwa_aln_core] calculate SA coordinate... 2461.23 sec

[bwa_aln_core] write to the disk... 0.10 sec

[bwa_aln_core] 14942208 sequences have been processed.

[bwa_aln_core] calculate SA coordinate... 1689.55 sec

[bwa_aln_core] write to the disk... 0.11 sec

[bwa_aln_core] 15204352 sequences have been processed.

[bwa_aln_core] calculate SA coordinate... 1367.76 sec

[bwa_aln_core] write to the disk... 0.09 sec

[bwa_aln_core] 15466496 sequences have been processed.

[bwa_aln_core] calculate SA coordinate... 1556.09 sec

[bwa_aln_core] write to the disk... 0.11 sec

[bwa_aln_core] 15728640 sequences have been processed.

[bwa_aln_core] calculate SA coordinate... 1212.85 sec

[bwa_aln_core] write to the disk... 0.11 sec

[bwa_aln_core] 15990784 sequences have been processed.

[bwa_aln_core] calculate SA coordinate... 1655.09 sec

[bwa_aln_core] write to the disk... 0.11 sec

[bwa_aln_core] 16252928 sequences have been processed.

[bwa_aln_core] calculate SA coordinate... 610.57 sec

[bwa_aln_core] write to the disk... 0.12 sec

[bwa_aln_core] 16515072 sequences have been processed.

[bwa_aln_core] calculate SA coordinate... 381.33 sec

[bwa_aln_core] write to the disk... 0.12 sec

[bwa_aln_core] 16777216 sequences have been processed.

[bwa_aln_core] calculate SA coordinate... 403.92 sec

[bwa_aln_core] write to the disk... 0.11 sec

[bwa_aln_core] 17039360 sequences have been processed.

[bwa_aln_core] calculate SA coordinate... 727.28 sec

[bwa_aln_core] write to the disk... 0.11 sec

[bwa_aln_core] 17301504 sequences have been processed.

[bwa_aln_core] calculate SA coordinate... 727.24 sec

[bwa_aln_core] write to the disk... 0.12 sec

[bwa_aln_core] 17563648 sequences have been processed.

[bwa_aln_core] calculate SA coordinate... 296.31 sec

[bwa_aln_core] write to the disk... 0.11 sec

[bwa_aln_core] 17825792 sequences have been processed.

[bwa_aln_core] calculate SA coordinate... 290.87 sec

[bwa_aln_core] write to the disk... 0.11 sec

[bwa_aln_core] 18087936 sequences have been processed.

[bwa_aln_core] calculate SA coordinate... 307.68 sec

[bwa_aln_core] write to the disk... 0.11 sec

[bwa_aln_core] 18350080 sequences have been processed.

[bwa_aln_core] calculate SA coordinate... 297.03 sec

[bwa_aln_core] write to the disk... 0.12 sec

[bwa_aln_core] 18612224 sequences have been processed.

[bwa_aln_core] calculate SA coordinate... 299.07 sec

[bwa_aln_core] write to the disk... 0.12 sec

[bwa_aln_core] 18874368 sequences have been processed.

[bwa_aln_core] calculate SA coordinate... 300.22 sec

[bwa_aln_core] write to the disk... 0.13 sec

[bwa_aln_core] 19136512 sequences have been processed.

[bwa_aln_core] calculate SA coordinate... 290.13 sec

[bwa_aln_core] write to the disk... 0.12 sec

[bwa_aln_core] 19398656 sequences have been processed.

[bwa_aln_core] calculate SA coordinate... 298.83 sec

[bwa_aln_core] write to the disk... 0.14 sec

[bwa_aln_core] 19660800 sequences have been processed.

[bwa_aln_core] calculate SA coordinate... 304.65 sec

[bwa_aln_core] write to the disk... 0.12 sec

[bwa_aln_core] 19922944 sequences have been processed.

[bwa_aln_core] calculate SA coordinate... 309.37 sec

[bwa_aln_core] write to the disk... 0.12 sec

[bwa_aln_core] 20185088 sequences have been processed.

[bwa_aln_core] calculate SA coordinate... 314.67 sec

[bwa_aln_core] write to the disk... 0.12 sec

[bwa_aln_core] 20447232 sequences have been processed.

[bwa_aln_core] calculate SA coordinate... 311.03 sec

[bwa_aln_core] write to the disk... 0.12 sec

[bwa_aln_core] 20709376 sequences have been processed.

[bwa_aln_core] calculate SA coordinate... 305.17 sec

[bwa_aln_core] write to the disk... 0.11 sec

[bwa_aln_core] 20971520 sequences have been processed.

[bwa_aln_core] calculate SA coordinate... 317.36 sec

[bwa_aln_core] write to the disk... 0.12 sec

[bwa_aln_core] 21233664 sequences have been processed.

[bwa_aln_core] calculate SA coordinate... 321.94 sec

[bwa_aln_core] write to the disk... 0.11 sec

[bwa_aln_core] 21495808 sequences have been processed.

[bwa_aln_core] calculate SA coordinate... 320.45 sec

[bwa_aln_core] write to the disk... 0.11 sec

[bwa_aln_core] 21757952 sequences have been processed.

[bwa_aln_core] calculate SA coordinate... 341.58 sec

[bwa_aln_core] write to the disk... 0.12 sec

[bwa_aln_core] 22020096 sequences have been processed.

[bwa_aln_core] calculate SA coordinate... 318.09 sec

[bwa_aln_core] write to the disk... 0.12 sec

[bwa_aln_core] 22282240 sequences have been processed.

[bwa_aln_core] calculate SA coordinate... 327.61 sec

[bwa_aln_core] write to the disk... 0.11 sec

[bwa_aln_core] 22544384 sequences have been processed.

[bwa_aln_core] calculate SA coordinate... 337.86 sec

[bwa_aln_core] write to the disk... 0.11 sec

[bwa_aln_core] 22806528 sequences have been processed.

[bwa_aln_core] calculate SA coordinate... 349.92 sec

[bwa_aln_core] write to the disk... 0.11 sec

[bwa_aln_core] 23068672 sequences have been processed.

[bwa_aln_core] calculate SA coordinate... 358.73 sec

[bwa_aln_core] write to the disk... 0.12 sec

[bwa_aln_core] 23330816 sequences have been processed.

[bwa_aln_core] calculate SA coordinate... 354.24 sec

[bwa_aln_core] write to the disk... 0.11 sec

[bwa_aln_core] 23592960 sequences have been processed.

[bwa_aln_core] calculate SA coordinate... 351.73 sec

[bwa_aln_core] write to the disk... 0.12 sec

[bwa_aln_core] 23855104 sequences have been processed.

[bwa_aln_core] calculate SA coordinate... 351.46 sec

[bwa_aln_core] write to the disk... 0.11 sec

[bwa_aln_core] 24117248 sequences have been processed.

[bwa_aln_core] calculate SA coordinate... 370.50 sec

[bwa_aln_core] write to the disk... 0.11 sec

[bwa_aln_core] 24379392 sequences have been processed.

[bwa_aln_core] calculate SA coordinate... 370.31 sec

[bwa_aln_core] write to the disk... 0.12 sec

[bwa_aln_core] 24641536 sequences have been processed.

[bwa_aln_core] calculate SA coordinate... 378.10 sec

[bwa_aln_core] write to the disk... 0.12 sec

[bwa_aln_core] 24903680 sequences have been processed.

[bwa_aln_core] calculate SA coordinate... 381.85 sec

[bwa_aln_core] write to the disk... 0.11 sec

[bwa_aln_core] 25165824 sequences have been processed.

[bwa_aln_core] calculate SA coordinate... 390.21 sec

[bwa_aln_core] write to the disk... 0.13 sec

[bwa_aln_core] 25427968 sequences have been processed.

[bwa_aln_core] calculate SA coordinate... 391.11 sec

[bwa_aln_core] write to the disk... 0.12 sec

[bwa_aln_core] 25690112 sequences have been processed.

[bwa_aln_core] calculate SA coordinate... 416.57 sec

[bwa_aln_core] write to the disk... 0.13 sec

[bwa_aln_core] 25952256 sequences have been processed.

[bwa_aln_core] calculate SA coordinate... 671.61 sec

[bwa_aln_core] write to the disk... 0.12 sec

[bwa_aln_core] 26214400 sequences have been processed.

[bwa_aln_core] calculate SA coordinate... 433.35 sec

[bwa_aln_core] write to the disk... 0.12 sec

[bwa_aln_core] 26476544 sequences have been processed.

[bwa_aln_core] calculate SA coordinate... 2017.71 sec

[bwa_aln_core] write to the disk... 0.10 sec

[bwa_aln_core] 26738688 sequences have been processed.

[bwa_aln_core] calculate SA coordinate... 531.52 sec

[bwa_aln_core] write to the disk... 0.10 sec

[bwa_aln_core] 27000832 sequences have been processed.

[bwa_aln_core] calculate SA coordinate... 597.50 sec

[bwa_aln_core] write to the disk... 0.10 sec

[bwa_aln_core] 27262976 sequences have been processed.

[bwa_aln_core] calculate SA coordinate... 625.89 sec

[bwa_aln_core] write to the disk... 0.11 sec

[bwa_aln_core] 27525120 sequences have been processed.

[bwa_aln_core] calculate SA coordinate... 1380.60 sec

[bwa_aln_core] write to the disk... 0.11 sec

[bwa_aln_core] 27787264 sequences have been processed.

[bwa_aln_core] calculate SA coordinate... 608.40 sec

[bwa_aln_core] write to the disk... 0.10 sec

[bwa_aln_core] 28049408 sequences have been processed.

[bwa_aln_core] calculate SA coordinate... 662.59 sec

[bwa_aln_core] write to the disk... 0.10 sec

[bwa_aln_core] 28311552 sequences have been processed.

[bwa_aln_core] calculate SA coordinate... 2644.15 sec

[bwa_aln_core] write to the disk... 0.10 sec

[bwa_aln_core] 28573696 sequences have been processed.

[bwa_aln_core] calculate SA coordinate... 599.51 sec

[bwa_aln_core] write to the disk... 0.10 sec

[bwa_aln_core] 28835840 sequences have been processed.

[bwa_aln_core] calculate SA coordinate... 750.74 sec

[bwa_aln_core] write to the disk... 0.10 sec

[bwa_aln_core] 29097984 sequences have been processed.

[bwa_aln_core] calculate SA coordinate... 551.73 sec

[bwa_aln_core] write to the disk... 0.10 sec

[bwa_aln_core] 29360128 sequences have been processed.

[bwa_aln_core] calculate SA coordinate... 293.61 sec

[bwa_aln_core] write to the disk... 0.11 sec

[bwa_aln_core] 29622272 sequences have been processed.

[bwa_aln_core] calculate SA coordinate... 297.78 sec

[bwa_aln_core] write to the disk... 0.10 sec

[bwa_aln_core] 29884416 sequences have been processed.

[bwa_aln_core] calculate SA coordinate... 287.71 sec

[bwa_aln_core] write to the disk... 0.11 sec

[bwa_aln_core] 30146560 sequences have been processed.

[bwa_aln_core] calculate SA coordinate... 294.76 sec

[bwa_aln_core] write to the disk... 0.11 sec

[bwa_aln_core] 30408704 sequences have been processed.

[bwa_aln_core] calculate SA coordinate... 291.30 sec

[bwa_aln_core] write to the disk... 0.11 sec

[bwa_aln_core] 30670848 sequences have been processed.

[bwa_aln_core] calculate SA coordinate... 294.44 sec

[bwa_aln_core] write to the disk... 0.11 sec

[bwa_aln_core] 30932992 sequences have been processed.

[bwa_aln_core] calculate SA coordinate... 310.51 sec

[bwa_aln_core] write to the disk... 0.10 sec

[bwa_aln_core] 31195136 sequences have been processed.

[bwa_aln_core] calculate SA coordinate... 288.87 sec

[bwa_aln_core] write to the disk... 0.11 sec

[bwa_aln_core] 31457280 sequences have been processed.

[bwa_aln_core] calculate SA coordinate... 282.79 sec

[bwa_aln_core] write to the disk... 0.11 sec

[bwa_aln_core] 31719424 sequences have been processed.

[bwa_aln_core] calculate SA coordinate... 292.66 sec

[bwa_aln_core] write to the disk... 0.11 sec

[bwa_aln_core] 31981568 sequences have been processed.

[bwa_aln_core] calculate SA coordinate... 302.65 sec

[bwa_aln_core] write to the disk... 0.11 sec

[bwa_aln_core] 32243712 sequences have been processed.

[bwa_aln_core] calculate SA coordinate... 299.70 sec

[bwa_aln_core] write to the disk... 0.11 sec

[bwa_aln_core] 32505856 sequences have been processed.

[bwa_aln_core] calculate SA coordinate... 291.02 sec

[bwa_aln_core] write to the disk... 0.10 sec

[bwa_aln_core] 32768000 sequences have been processed.

[bwa_aln_core] calculate SA coordinate... 329.37 sec

[bwa_aln_core] write to the disk... 0.11 sec

[bwa_aln_core] 33030144 sequences have been processed.

[bwa_aln_core] calculate SA coordinate... 329.78 sec

[bwa_aln_core] write to the disk... 0.11 sec

[bwa_aln_core] 33292288 sequences have been processed.

[bwa_aln_core] calculate SA coordinate... 304.85 sec

[bwa_aln_core] write to the disk... 0.11 sec

[bwa_aln_core] 33554432 sequences have been processed.

[bwa_aln_core] calculate SA coordinate... 304.37 sec

[bwa_aln_core] write to the disk... 0.10 sec

[bwa_aln_core] 33816576 sequences have been processed.

[bwa_aln_core] calculate SA coordinate... 299.87 sec

[bwa_aln_core] write to the disk... 0.12 sec

[bwa_aln_core] 34078720 sequences have been processed.

[bwa_aln_core] calculate SA coordinate... 297.36 sec

[bwa_aln_core] write to the disk... 0.11 sec

[bwa_aln_core] 34340864 sequences have been processed.

[bwa_aln_core] calculate SA coordinate... 297.59 sec

[bwa_aln_core] write to the disk... 0.11 sec

[bwa_aln_core] 34603008 sequences have been processed.

[bwa_aln_core] calculate SA coordinate... 282.38 sec

[bwa_aln_core] write to the disk... 0.11 sec

[bwa_aln_core] 34865152 sequences have been processed.

[bwa_aln_core] calculate SA coordinate... 302.82 sec

[bwa_aln_core] write to the disk... 0.11 sec

[bwa_aln_core] 35127296 sequences have been processed.

[bwa_aln_core] calculate SA coordinate... 298.49 sec

[bwa_aln_core] write to the disk... 0.10 sec

[bwa_aln_core] 35389440 sequences have been processed.

[bwa_aln_core] calculate SA coordinate... 293.61 sec

[bwa_aln_core] write to the disk... 0.11 sec

[bwa_aln_core] 35651584 sequences have been processed.

[bwa_aln_core] calculate SA coordinate... 300.31 sec

[bwa_aln_core] write to the disk... 0.11 sec

[bwa_aln_core] 35913728 sequences have been processed.

[bwa_aln_core] calculate SA coordinate... 300.09 sec

[bwa_aln_core] write to the disk... 0.10 sec

[bwa_aln_core] 36175872 sequences have been processed.

[bwa_aln_core] calculate SA coordinate... 291.11 sec

[bwa_aln_core] write to the disk... 0.11 sec

[bwa_aln_core] 36438016 sequences have been processed.

[bwa_aln_core] calculate SA coordinate... 603.78 sec

[bwa_aln_core] write to the disk... 0.12 sec

[bwa_aln_core] 36700160 sequences have been processed.

[bwa_aln_core] calculate SA coordinate... 339.28 sec

[bwa_aln_core] write to the disk... 0.12 sec

[bwa_aln_core] 36962304 sequences have been processed.

[bwa_aln_core] calculate SA coordinate... 494.41 sec

[bwa_aln_core] write to the disk... 0.11 sec

[bwa_aln_core] 37224448 sequences have been processed.

[bwa_aln_core] calculate SA coordinate... 300.25 sec

[bwa_aln_core] write to the disk... 0.12 sec

[bwa_aln_core] 37486592 sequences have been processed.

[bwa_aln_core] calculate SA coordinate... 299.57 sec

[bwa_aln_core] write to the disk... 0.12 sec

[bwa_aln_core] 37748736 sequences have been processed.

[bwa_aln_core] calculate SA coordinate... 293.95 sec

[bwa_aln_core] write to the disk... 0.12 sec

[bwa_aln_core] 38010880 sequences have been processed.

[bwa_aln_core] calculate SA coordinate... 429.67 sec

[bwa_aln_core] write to the disk... 0.12 sec

[bwa_aln_core] 38273024 sequences have been processed.

[bwa_aln_core] calculate SA coordinate... 293.80 sec

[bwa_aln_core] write to the disk... 0.12 sec

[bwa_aln_core] 38535168 sequences have been processed.

[bwa_aln_core] calculate SA coordinate... 303.61 sec

[bwa_aln_core] write to the disk... 0.12 sec

[bwa_aln_core] 38797312 sequences have been processed.

[bwa_aln_core] calculate SA coordinate... 291.64 sec

[bwa_aln_core] write to the disk... 0.11 sec

[bwa_aln_core] 39059456 sequences have been processed.

[bwa_aln_core] calculate SA coordinate... 294.47 sec

[bwa_aln_core] write to the disk... 0.12 sec

[bwa_aln_core] 39321600 sequences have been processed.

[bwa_aln_core] calculate SA coordinate... 280.08 sec

[bwa_aln_core] write to the disk... 0.11 sec

[bwa_aln_core] 39583744 sequences have been processed.

[bwa_aln_core] calculate SA coordinate... 297.46 sec

[bwa_aln_core] write to the disk... 0.12 sec

[bwa_aln_core] 39845888 sequences have been processed.

[bwa_aln_core] calculate SA coordinate... 316.58 sec

[bwa_aln_core] write to the disk... 0.11 sec

[bwa_aln_core] 40108032 sequences have been processed.

[bwa_aln_core] calculate SA coordinate... 301.58 sec

[bwa_aln_core] write to the disk... 0.11 sec

[bwa_aln_core] 40370176 sequences have been processed.

[bwa_aln_core] calculate SA coordinate... 297.40 sec

[bwa_aln_core] write to the disk... 0.12 sec

[bwa_aln_core] 40632320 sequences have been processed.

[bwa_aln_core] calculate SA coordinate... 301.20 sec

[bwa_aln_core] write to the disk... 0.12 sec

[bwa_aln_core] 40894464 sequences have been processed.

[bwa_aln_core] calculate SA coordinate... 301.52 sec

[bwa_aln_core] write to the disk... 0.12 sec

[bwa_aln_core] 41156608 sequences have been processed.

[bwa_aln_core] calculate SA coordinate... 303.68 sec

[bwa_aln_core] write to the disk... 0.13 sec

[bwa_aln_core] 41418752 sequences have been processed.

[bwa_aln_core] calculate SA coordinate... 306.77 sec

[bwa_aln_core] write to the disk... 0.11 sec

[bwa_aln_core] 41680896 sequences have been processed.

[bwa_aln_core] calculate SA coordinate... 303.65 sec

[bwa_aln_core] write to the disk... 0.13 sec

[bwa_aln_core] 41943040 sequences have been processed.

[bwa_aln_core] calculate SA coordinate... 314.87 sec

[bwa_aln_core] write to the disk... 0.11 sec

[bwa_aln_core] 42205184 sequences have been processed.

[bwa_aln_core] calculate SA coordinate... 313.98 sec

[bwa_aln_core] write to the disk... 0.11 sec

[bwa_aln_core] 42467328 sequences have been processed.

[bwa_aln_core] calculate SA coordinate... 520.90 sec

[bwa_aln_core] write to the disk... 0.10 sec

[bwa_aln_core] 42729472 sequences have been processed.

[bwa_aln_core] calculate SA coordinate... 433.66 sec

[bwa_aln_core] write to the disk... 0.11 sec

[bwa_aln_core] 42991616 sequences have been processed.

[bwa_aln_core] calculate SA coordinate... 307.94 sec

[bwa_aln_core] write to the disk... 0.11 sec

[bwa_aln_core] 43253760 sequences have been processed.

[bwa_aln_core] calculate SA coordinate... 314.13 sec

[bwa_aln_core] write to the disk... 0.11 sec

[bwa_aln_core] 43515904 sequences have been processed.

[bwa_aln_core] calculate SA coordinate... 309.47 sec

[bwa_aln_core] write to the disk... 0.11 sec

[bwa_aln_core] 43778048 sequences have been processed.

[bwa_aln_core] calculate SA coordinate... 745.12 sec

[bwa_aln_core] write to the disk... 0.11 sec

[bwa_aln_core] 44040192 sequences have been processed.

[bwa_aln_core] calculate SA coordinate... 311.84 sec

[bwa_aln_core] write to the disk... 0.10 sec

[bwa_aln_core] 44302336 sequences have been processed.

[bwa_aln_core] calculate SA coordinate... 323.99 sec

[bwa_aln_core] write to the disk... 0.10 sec

[bwa_aln_core] 44564480 sequences have been processed.

[bwa_aln_core] calculate SA coordinate... 332.90 sec

[bwa_aln_core] write to the disk... 0.11 sec

[bwa_aln_core] 44826624 sequences have been processed.

[bwa_aln_core] calculate SA coordinate... 346.78 sec

[bwa_aln_core] write to the disk... 0.11 sec

[bwa_aln_core] 45088768 sequences have been processed.

[bwa_aln_core] calculate SA coordinate... 365.33 sec

[bwa_aln_core] write to the disk... 0.11 sec

[bwa_aln_core] 45350912 sequences have been processed.

[bwa_aln_core] calculate SA coordinate... 354.14 sec

[bwa_aln_core] write to the disk... 0.10 sec

[bwa_aln_core] 45613056 sequences have been processed.

[bwa_aln_core] calculate SA coordinate... 1165.54 sec

[bwa_aln_core] write to the disk... 0.11 sec

[bwa_aln_core] 45875200 sequences have been processed.

[bwa_aln_core] calculate SA coordinate... 356.20 sec

[bwa_aln_core] write to the disk... 0.11 sec

[bwa_aln_core] 46137344 sequences have been processed.

[bwa_aln_core] calculate SA coordinate... 354.14 sec

[bwa_aln_core] write to the disk... 0.11 sec

[bwa_aln_core] 46399488 sequences have been processed.

[bwa_aln_core] calculate SA coordinate... 340.86 sec

[bwa_aln_core] write to the disk... 0.11 sec

[bwa_aln_core] 46661632 sequences have been processed.

[bwa_aln_core] calculate SA coordinate... 338.23 sec

[bwa_aln_core] write to the disk... 0.11 sec

[bwa_aln_core] 46923776 sequences have been processed.

[bwa_aln_core] calculate SA coordinate... 339.60 sec

[bwa_aln_core] write to the disk... 0.11 sec

[bwa_aln_core] 47185920 sequences have been processed.

[bwa_aln_core] calculate SA coordinate... 344.09 sec

[bwa_aln_core] write to the disk... 0.11 sec

[bwa_aln_core] 47448064 sequences have been processed.

[bwa_aln_core] calculate SA coordinate... 338.78 sec

[bwa_aln_core] write to the disk... 0.11 sec

[bwa_aln_core] 47710208 sequences have been processed.

[bwa_aln_core] calculate SA coordinate... 766.25 sec

[bwa_aln_core] write to the disk... 0.13 sec

[bwa_aln_core] 47972352 sequences have been processed.

[bwa_aln_core] calculate SA coordinate... 432.77 sec

[bwa_aln_core] write to the disk... 0.12 sec

[bwa_aln_core] 48234496 sequences have been processed.

[bwa_aln_core] calculate SA coordinate... 346.36 sec

[bwa_aln_core] write to the disk... 0.12 sec

[bwa_aln_core] 48496640 sequences have been processed.

[bwa_aln_core] calculate SA coordinate... 339.12 sec

[bwa_aln_core] write to the disk... 0.11 sec

[bwa_aln_core] 48758784 sequences have been processed.

[bwa_aln_core] calculate SA coordinate... 343.29 sec

[bwa_aln_core] write to the disk... 0.12 sec

[bwa_aln_core] 49020928 sequences have been processed.

[bwa_aln_core] calculate SA coordinate... 342.15 sec

[bwa_aln_core] write to the disk... 0.12 sec

[bwa_aln_core] 49283072 sequences have been processed.

[bwa_aln_core] calculate SA coordinate... 356.37 sec

[bwa_aln_core] write to the disk... 0.12 sec

[bwa_aln_core] 49545216 sequences have been processed.

[bwa_aln_core] calculate SA coordinate... 355.28 sec

[bwa_aln_core] write to the disk... 0.12 sec

[bwa_aln_core] 49807360 sequences have been processed.

[bwa_aln_core] calculate SA coordinate... 362.08 sec

[bwa_aln_core] write to the disk... 0.13 sec

[bwa_aln_core] 50069504 sequences have been processed.

[bwa_aln_core] calculate SA coordinate... 1037.59 sec

[bwa_aln_core] write to the disk... 0.12 sec

[bwa_aln_core] 50331648 sequences have been processed.

[bwa_aln_core] calculate SA coordinate... 359.24 sec

[bwa_aln_core] write to the disk... 0.12 sec

[bwa_aln_core] 50593792 sequences have been processed.

[bwa_aln_core] calculate SA coordinate... 370.52 sec

[bwa_aln_core] write to the disk... 0.12 sec

[bwa_aln_core] 50855936 sequences have been processed.

[bwa_aln_core] calculate SA coordinate... 368.86 sec

[bwa_aln_core] write to the disk... 0.13 sec

[bwa_aln_core] 51118080 sequences have been processed.

[bwa_aln_core] calculate SA coordinate... 383.80 sec

[bwa_aln_core] write to the disk... 0.11 sec

[bwa_aln_core] 51380224 sequences have been processed.

[bwa_aln_core] calculate SA coordinate... 388.24 sec

[bwa_aln_core] write to the disk... 0.12 sec

[bwa_aln_core] 51642368 sequences have been processed.

[bwa_aln_core] calculate SA coordinate... 389.69 sec

[bwa_aln_core] write to the disk... 0.12 sec

[bwa_aln_core] 51904512 sequences have been processed.

[bwa_aln_core] calculate SA coordinate... 427.30 sec

[bwa_aln_core] write to the disk... 0.11 sec

[bwa_aln_core] 52166656 sequences have been processed.

[bwa_aln_core] calculate SA coordinate... 620.44 sec

[bwa_aln_core] write to the disk... 0.12 sec

[bwa_aln_core] 52428800 sequences have been processed.

[bwa_aln_core] calculate SA coordinate... 696.03 sec

[bwa_aln_core] write to the disk... 0.10 sec

[bwa_aln_core] 52690944 sequences have been processed.

[bwa_aln_core] calculate SA coordinate... 515.49 sec

[bwa_aln_core] write to the disk... 0.11 sec

[bwa_aln_core] 52953088 sequences have been processed.

[bwa_aln_core] calculate SA coordinate... 705.33 sec

[bwa_aln_core] write to the disk... 0.11 sec

[bwa_aln_core] 53215232 sequences have been processed.

[bwa_aln_core] calculate SA coordinate... 435.59 sec

[bwa_aln_core] write to the disk... 0.10 sec

[bwa_aln_core] 53477376 sequences have been processed.

[bwa_aln_core] calculate SA coordinate... 479.81 sec

[bwa_aln_core] write to the disk... 0.11 sec

[bwa_aln_core] 53739520 sequences have been processed.

[bwa_aln_core] calculate SA coordinate... 450.14 sec

[bwa_aln_core] write to the disk... 0.11 sec

[bwa_aln_core] 54001664 sequences have been processed.

[bwa_aln_core] calculate SA coordinate... 475.05 sec

[bwa_aln_core] write to the disk... 0.10 sec

[bwa_aln_core] 54263808 sequences have been processed.

[bwa_aln_core] calculate SA coordinate... 490.67 sec

[bwa_aln_core] write to the disk... 0.11 sec

[bwa_aln_core] 54525952 sequences have been processed.

[bwa_aln_core] calculate SA coordinate... 485.93 sec

[bwa_aln_core] write to the disk... 0.10 sec

[bwa_aln_core] 54788096 sequences have been processed.

[bwa_aln_core] calculate SA coordinate... 686.53 sec

[bwa_aln_core] write to the disk... 0.11 sec

[bwa_aln_core] 55050240 sequences have been processed.

[bwa_aln_core] calculate SA coordinate... 564.39 sec

[bwa_aln_core] write to the disk... 0.10 sec

[bwa_aln_core] 55312384 sequences have been processed.

[bwa_aln_core] calculate SA coordinate... 774.47 sec

[bwa_aln_core] write to the disk... 0.11 sec

[bwa_aln_core] 55574528 sequences have been processed.

[bwa_aln_core] calculate SA coordinate... 621.32 sec

[bwa_aln_core] write to the disk... 0.11 sec

[bwa_aln_core] 55836672 sequences have been processed.

[bwa_aln_core] calculate SA coordinate... 1671.35 sec

[bwa_aln_core] write to the disk... 0.10 sec

[bwa_aln_core] 56098816 sequences have been processed.

[bwa_aln_core] calculate SA coordinate... 570.24 sec

[bwa_aln_core] write to the disk... 0.11 sec

[bwa_aln_core] 56360960 sequences have been processed.

[bwa_aln_core] calculate SA coordinate... 582.67 sec

[bwa_aln_core] write to the disk... 0.11 sec

[bwa_aln_core] 56623104 sequences have been processed.

[bwa_aln_core] calculate SA coordinate... 596.58 sec

[bwa_aln_core] write to the disk... 0.10 sec

[bwa_aln_core] 56885248 sequences have been processed.

[bwa_aln_core] calculate SA coordinate... 611.98 sec

[bwa_aln_core] write to the disk... 0.10 sec

[bwa_aln_core] 57147392 sequences have been processed.

[bwa_aln_core] calculate SA coordinate... 615.20 sec

[bwa_aln_core] write to the disk... 0.10 sec

[bwa_aln_core] 57409536 sequences have been processed.

[bwa_aln_core] calculate SA coordinate... 606.10 sec

[bwa_aln_core] write to the disk... 0.10 sec

[bwa_aln_core] 57671680 sequences have been processed.

[bwa_aln_core] calculate SA coordinate... 541.48 sec

[bwa_aln_core] write to the disk... 0.10 sec

[bwa_aln_core] 57933824 sequences have been processed.

[bwa_aln_core] calculate SA coordinate... 274.31 sec

[bwa_aln_core] write to the disk... 0.12 sec

[bwa_aln_core] 58195968 sequences have been processed.

[bwa_aln_core] calculate SA coordinate... 266.12 sec

[bwa_aln_core] write to the disk... 0.11 sec

[bwa_aln_core] 58458112 sequences have been processed.

[bwa_aln_core] calculate SA coordinate... 544.83 sec

[bwa_aln_core] write to the disk... 0.12 sec

[bwa_aln_core] 58720256 sequences have been processed.

[bwa_aln_core] calculate SA coordinate... 277.16 sec

[bwa_aln_core] write to the disk... 0.11 sec

[bwa_aln_core] 58982400 sequences have been processed.

[bwa_aln_core] calculate SA coordinate... 1230.07 sec

[bwa_aln_core] write to the disk... 0.11 sec

[bwa_aln_core] 59244544 sequences have been processed.

[bwa_aln_core] calculate SA coordinate... 345.86 sec

[bwa_aln_core] write to the disk... 0.09 sec

[bwa_aln_core] 59506688 sequences have been processed.

[bwa_aln_core] calculate SA coordinate... 403.24 sec

[bwa_aln_core] write to the disk... 0.10 sec

[bwa_aln_core] 59768832 sequences have been processed.

[bwa_aln_core] calculate SA coordinate... 302.50 sec

[bwa_aln_core] write to the disk... 0.10 sec

[bwa_aln_core] 60030976 sequences have been processed.

[bwa_aln_core] calculate SA coordinate... 266.16 sec

[bwa_aln_core] write to the disk... 0.11 sec

[bwa_aln_core] 60293120 sequences have been processed.

[bwa_aln_core] calculate SA coordinate... 979.29 sec

[bwa_aln_core] write to the disk... 0.11 sec

[bwa_aln_core] 60555264 sequences have been processed.

[bwa_aln_core] calculate SA coordinate... 258.38 sec

[bwa_aln_core] write to the disk... 0.11 sec

[bwa_aln_core] 60817408 sequences have been processed.

[bwa_aln_core] calculate SA coordinate... 264.29 sec

[bwa_aln_core] write to the disk... 0.11 sec

[bwa_aln_core] 61079552 sequences have been processed.

[bwa_aln_core] calculate SA coordinate... 269.58 sec

[bwa_aln_core] write to the disk... 0.12 sec

[bwa_aln_core] 61341696 sequences have been processed.

[bwa_aln_core] calculate SA coordinate... 270.78 sec

[bwa_aln_core] write to the disk... 0.11 sec

[bwa_aln_core] 61603840 sequences have been processed.

[bwa_aln_core] calculate SA coordinate... 262.17 sec

[bwa_aln_core] write to the disk... 0.11 sec

[bwa_aln_core] 61865984 sequences have been processed.

[bwa_aln_core] calculate SA coordinate... 273.58 sec

[bwa_aln_core] write to the disk... 0.11 sec

[bwa_aln_core] 62128128 sequences have been processed.

[bwa_aln_core] calculate SA coordinate... 257.40 sec

[bwa_aln_core] write to the disk... 0.09 sec

[bwa_aln_core] 62390272 sequences have been processed.

[bwa_aln_core] calculate SA coordinate... 253.87 sec

[bwa_aln_core] write to the disk... 0.10 sec

[bwa_aln_core] 62652416 sequences have been processed.

[bwa_aln_core] calculate SA coordinate... 252.85 sec

[bwa_aln_core] write to the disk... 0.11 sec

[bwa_aln_core] 62914560 sequences have been processed.

[bwa_aln_core] calculate SA coordinate... 268.35 sec

[bwa_aln_core] write to the disk... 0.10 sec

[bwa_aln_core] 63176704 sequences have been processed.

[bwa_aln_core] calculate SA coordinate... 256.47 sec

[bwa_aln_core] write to the disk... 0.10 sec

[bwa_aln_core] 63438848 sequences have been processed.

[bwa_aln_core] calculate SA coordinate... 243.26 sec

[bwa_aln_core] write to the disk... 0.11 sec

[bwa_aln_core] 63700992 sequences have been processed.

[bwa_aln_core] calculate SA coordinate... 250.18 sec

[bwa_aln_core] write to the disk... 0.12 sec

[bwa_aln_core] 63963136 sequences have been processed.

[bwa_aln_core] calculate SA coordinate... 251.01 sec

[bwa_aln_core] write to the disk... 0.11 sec

[bwa_aln_core] 64225280 sequences have been processed.

[bwa_aln_core] calculate SA coordinate... 263.80 sec

[bwa_aln_core] write to the disk... 0.10 sec

[bwa_aln_core] 64487424 sequences have been processed.

[bwa_aln_core] calculate SA coordinate... 248.95 sec

[bwa_aln_core] write to the disk... 0.11 sec

[bwa_aln_core] 64749568 sequences have been processed.

[bwa_aln_core] calculate SA coordinate... 250.19 sec

[bwa_aln_core] write to the disk... 0.11 sec

[bwa_aln_core] 65011712 sequences have been processed.

[bwa_aln_core] calculate SA coordinate... 249.57 sec

[bwa_aln_core] write to the disk... 0.10 sec

[bwa_aln_core] 65273856 sequences have been processed.

[bwa_aln_core] calculate SA coordinate... 250.09 sec

[bwa_aln_core] write to the disk... 0.10 sec

[bwa_aln_core] 65536000 sequences have been processed.

[bwa_aln_core] calculate SA coordinate... 251.51 sec

[bwa_aln_core] write to the disk... 0.11 sec

[bwa_aln_core] 65798144 sequences have been processed.

[bwa_aln_core] calculate SA coordinate... 254.65 sec

[bwa_aln_core] write to the disk... 0.10 sec

[bwa_aln_core] 66060288 sequences have been processed.

[bwa_aln_core] calculate SA coordinate... 262.30 sec

[bwa_aln_core] write to the disk... 0.11 sec

[bwa_aln_core] 66322432 sequences have been processed.

[bwa_aln_core] calculate SA coordinate... 268.16 sec

[bwa_aln_core] write to the disk... 0.11 sec

[bwa_aln_core] 66584576 sequences have been processed.

[bwa_aln_core] calculate SA coordinate... 266.11 sec

[bwa_aln_core] write to the disk... 0.12 sec

[bwa_aln_core] 66846720 sequences have been processed.

[bwa_aln_core] calculate SA coordinate... 800.32 sec

[bwa_aln_core] write to the disk... 0.11 sec

[bwa_aln_core] 67108864 sequences have been processed.

[bwa_aln_core] calculate SA coordinate... 304.10 sec

[bwa_aln_core] write to the disk... 0.11 sec

[bwa_aln_core] 67371008 sequences have been processed.

[bwa_aln_core] calculate SA coordinate... 291.31 sec

[bwa_aln_core] write to the disk... 0.11 sec

[bwa_aln_core] 67633152 sequences have been processed.

[bwa_aln_core] calculate SA coordinate... 306.30 sec

[bwa_aln_core] write to the disk... 0.10 sec

[bwa_aln_core] 67895296 sequences have been processed.

[bwa_aln_core] calculate SA coordinate... 256.21 sec

[bwa_aln_core] write to the disk... 0.12 sec

[bwa_aln_core] 68157440 sequences have been processed.

[bwa_aln_core] calculate SA coordinate... 328.11 sec

[bwa_aln_core] write to the disk... 0.11 sec

[bwa_aln_core] 68419584 sequences have been processed.

[bwa_aln_core] calculate SA coordinate... 1420.92 sec

[bwa_aln_core] write to the disk... 0.12 sec

[bwa_aln_core] 68681728 sequences have been processed.

[bwa_aln_core] calculate SA coordinate... 332.28 sec

[bwa_aln_core] write to the disk... 0.11 sec

[bwa_aln_core] 68943872 sequences have been processed.

[bwa_aln_core] calculate SA coordinate... 725.70 sec

[bwa_aln_core] write to the disk... 0.11 sec

[bwa_aln_core] 69206016 sequences have been processed.

[bwa_aln_core] calculate SA coordinate... 274.50 sec

[bwa_aln_core] write to the disk... 0.11 sec

[bwa_aln_core] 69468160 sequences have been processed.

[bwa_aln_core] calculate SA coordinate... 282.02 sec

[bwa_aln_core] write to the disk... 0.10 sec

[bwa_aln_core] 69730304 sequences have been processed.

[bwa_aln_core] calculate SA coordinate... 264.38 sec

[bwa_aln_core] write to the disk... 0.11 sec

[bwa_aln_core] 69992448 sequences have been processed.

[bwa_aln_core] calculate SA coordinate... 274.70 sec

[bwa_aln_core] write to the disk... 0.10 sec

[bwa_aln_core] 70254592 sequences have been processed.

[bwa_aln_core] calculate SA coordinate... 254.11 sec

[bwa_aln_core] write to the disk... 0.11 sec

[bwa_aln_core] 70516736 sequences have been processed.

[bwa_aln_core] calculate SA coordinate... 259.14 sec

[bwa_aln_core] write to the disk... 0.11 sec

[bwa_aln_core] 70778880 sequences have been processed.

[bwa_aln_core] calculate SA coordinate... 256.44 sec

[bwa_aln_core] write to the disk... 0.10 sec

[bwa_aln_core] 71041024 sequences have been processed.

[bwa_aln_core] calculate SA coordinate... 256.80 sec

[bwa_aln_core] write to the disk... 0.10 sec

[bwa_aln_core] 71303168 sequences have been processed.

[bwa_aln_core] calculate SA coordinate... 257.84 sec

[bwa_aln_core] write to the disk... 0.11 sec

[bwa_aln_core] 71565312 sequences have been processed.

[bwa_aln_core] calculate SA coordinate... 259.11 sec

[bwa_aln_core] write to the disk... 0.11 sec

[bwa_aln_core] 71827456 sequences have been processed.

[bwa_aln_core] calculate SA coordinate... 260.05 sec

[bwa_aln_core] write to the disk... 0.10 sec

[bwa_aln_core] 72089600 sequences have been processed.

[bwa_aln_core] calculate SA coordinate... 262.89 sec

[bwa_aln_core] write to the disk... 0.11 sec

[bwa_aln_core] 72351744 sequences have been processed.

[bwa_aln_core] calculate SA coordinate... 263.64 sec

[bwa_aln_core] write to the disk... 0.11 sec

[bwa_aln_core] 72613888 sequences have been processed.

[bwa_aln_core] calculate SA coordinate... 262.15 sec

[bwa_aln_core] write to the disk... 0.11 sec

[bwa_aln_core] 72876032 sequences have been processed.

[bwa_aln_core] calculate SA coordinate... 262.73 sec

[bwa_aln_core] write to the disk... 0.11 sec

[bwa_aln_core] 73138176 sequences have been processed.

[bwa_aln_core] calculate SA coordinate... 258.71 sec

[bwa_aln_core] write to the disk... 0.10 sec

[bwa_aln_core] 73400320 sequences have been processed.

[bwa_aln_core] calculate SA coordinate... 265.72 sec

[bwa_aln_core] write to the disk... 0.10 sec

[bwa_aln_core] 73662464 sequences have been processed.

[bwa_aln_core] calculate SA coordinate... 261.03 sec

[bwa_aln_core] write to the disk... 0.11 sec

[bwa_aln_core] 73924608 sequences have been processed.

[bwa_aln_core] calculate SA coordinate... 266.49 sec

[bwa_aln_core] write to the disk... 0.11 sec

[bwa_aln_core] 74186752 sequences have been processed.

[bwa_aln_core] calculate SA coordinate... 260.23 sec

[bwa_aln_core] write to the disk... 0.11 sec

[bwa_aln_core] 74448896 sequences have been processed.

[bwa_aln_core] calculate SA coordinate... 269.46 sec

[bwa_aln_core] write to the disk... 0.11 sec

[bwa_aln_core] 74711040 sequences have been processed.

[bwa_aln_core] calculate SA coordinate... 266.23 sec

[bwa_aln_core] write to the disk... 0.11 sec

[bwa_aln_core] 74973184 sequences have been processed.

[bwa_aln_core] calculate SA coordinate... 266.28 sec

[bwa_aln_core] write to the disk... 0.10 sec

[bwa_aln_core] 75235328 sequences have been processed.

[bwa_aln_core] calculate SA coordinate... 253.86 sec

[bwa_aln_core] write to the disk... 0.11 sec

[bwa_aln_core] 75497472 sequences have been processed.

[bwa_aln_core] calculate SA coordinate... 255.40 sec

[bwa_aln_core] write to the disk... 0.10 sec

[bwa_aln_core] 75759616 sequences have been processed.

[bwa_aln_core] calculate SA coordinate... 259.94 sec

[bwa_aln_core] write to the disk... 0.11 sec

[bwa_aln_core] 76021760 sequences have been processed.

[bwa_aln_core] calculate SA coordinate... 276.14 sec

[bwa_aln_core] write to the disk... 0.11 sec

[bwa_aln_core] 76283904 sequences have been processed.

[bwa_aln_core] calculate SA coordinate... 274.41 sec

[bwa_aln_core] write to the disk... 0.11 sec

[bwa_aln_core] 76546048 sequences have been processed.

[bwa_aln_core] calculate SA coordinate... 269.50 sec

[bwa_aln_core] write to the disk... 0.11 sec

[bwa_aln_core] 76808192 sequences have been processed.

[bwa_aln_core] calculate SA coordinate... 274.81 sec

[bwa_aln_core] write to the disk... 0.11 sec

[bwa_aln_core] 77070336 sequences have been processed.

[bwa_aln_core] calculate SA coordinate... 282.69 sec

[bwa_aln_core] write to the disk... 0.11 sec

[bwa_aln_core] 77332480 sequences have been processed.

[bwa_aln_core] calculate SA coordinate... 273.97 sec

[bwa_aln_core] write to the disk... 0.11 sec

[bwa_aln_core] 77594624 sequences have been processed.

[bwa_aln_core] calculate SA coordinate... 278.48 sec

[bwa_aln_core] write to the disk... 0.09 sec

[bwa_aln_core] 77856768 sequences have been processed.

[bwa_aln_core] calculate SA coordinate... 271.83 sec

[bwa_aln_core] write to the disk... 0.10 sec

[bwa_aln_core] 78118912 sequences have been processed.

[bwa_aln_core] calculate SA coordinate... 438.03 sec

[bwa_aln_core] write to the disk... 0.10 sec

[bwa_aln_core] 78381056 sequences have been processed.

[bwa_aln_core] calculate SA coordinate... 280.91 sec

[bwa_aln_core] write to the disk... 0.11 sec

[bwa_aln_core] 78643200 sequences have been processed.

[bwa_aln_core] calculate SA coordinate... 536.04 sec

[bwa_aln_core] write to the disk... 0.10 sec

[bwa_aln_core] 78905344 sequences have been processed.

[bwa_aln_core] calculate SA coordinate... 290.32 sec

[bwa_aln_core] write to the disk... 0.11 sec

[bwa_aln_core] 79167488 sequences have been processed.

[bwa_aln_core] calculate SA coordinate... 286.66 sec

[bwa_aln_core] write to the disk... 0.11 sec

[bwa_aln_core] 79429632 sequences have been processed.

[bwa_aln_core] calculate SA coordinate... 276.72 sec

[bwa_aln_core] write to the disk... 0.11 sec

[bwa_aln_core] 79691776 sequences have been processed.

[bwa_aln_core] calculate SA coordinate... 281.82 sec

[bwa_aln_core] write to the disk... 0.10 sec

[bwa_aln_core] 79953920 sequences have been processed.

[bwa_aln_core] calculate SA coordinate... 283.18 sec

[bwa_aln_core] write to the disk... 0.10 sec

[bwa_aln_core] 80216064 sequences have been processed.

[bwa_aln_core] calculate SA coordinate... 396.74 sec

[bwa_aln_core] write to the disk... 0.11 sec

[bwa_aln_core] 80478208 sequences have been processed.

[bwa_aln_core] calculate SA coordinate... 401.65 sec

[bwa_aln_core] write to the disk... 0.11 sec

[bwa_aln_core] 80740352 sequences have been processed.

[bwa_aln_core] calculate SA coordinate... 661.09 sec

[bwa_aln_core] write to the disk... 0.12 sec

[bwa_aln_core] 81002496 sequences have been processed.

[bwa_aln_core] calculate SA coordinate... 402.69 sec

[bwa_aln_core] write to the disk... 0.11 sec

[bwa_aln_core] 81264640 sequences have been processed.

[bwa_aln_core] calculate SA coordinate... 299.81 sec

[bwa_aln_core] write to the disk... 0.11 sec

[bwa_aln_core] 81526784 sequences have been processed.

[bwa_aln_core] calculate SA coordinate... 297.05 sec

[bwa_aln_core] write to the disk... 0.11 sec

[bwa_aln_core] 81788928 sequences have been processed.

[bwa_aln_core] calculate SA coordinate... 302.19 sec

[bwa_aln_core] write to the disk... 0.11 sec

[bwa_aln_core] 82051072 sequences have been processed.

[bwa_aln_core] calculate SA coordinate... 318.73 sec

[bwa_aln_core] write to the disk... 0.10 sec

[bwa_aln_core] 82313216 sequences have been processed.

[bwa_aln_core] calculate SA coordinate... 352.80 sec

[bwa_aln_core] write to the disk... 0.10 sec

[bwa_aln_core] 82575360 sequences have been processed.

[bwa_aln_core] calculate SA coordinate... 363.12 sec

[bwa_aln_core] write to the disk... 0.11 sec

[bwa_aln_core] 82837504 sequences have been processed.

[bwa_aln_core] calculate SA coordinate... 363.82 sec

[bwa_aln_core] write to the disk... 0.10 sec

[bwa_aln_core] 83099648 sequences have been processed.

[bwa_aln_core] calculate SA coordinate... 393.74 sec

[bwa_aln_core] write to the disk... 0.10 sec

[bwa_aln_core] 83361792 sequences have been processed.

[bwa_aln_core] calculate SA coordinate... 386.04 sec

[bwa_aln_core] write to the disk... 0.11 sec

[bwa_aln_core] 83623936 sequences have been processed.

[bwa_aln_core] calculate SA coordinate... 384.73 sec

[bwa_aln_core] write to the disk... 0.11 sec

[bwa_aln_core] 83886080 sequences have been processed.

[bwa_aln_core] calculate SA coordinate... 415.59 sec

[bwa_aln_core] write to the disk... 0.11 sec

[bwa_aln_core] 84148224 sequences have been processed.

[bwa_aln_core] calculate SA coordinate... 391.77 sec

[bwa_aln_core] write to the disk... 0.10 sec

[bwa_aln_core] 84410368 sequences have been processed.

[bwa_aln_core] calculate SA coordinate... 429.54 sec

[bwa_aln_core] write to the disk... 0.11 sec

[bwa_aln_core] 84672512 sequences have been processed.

[bwa_aln_core] calculate SA coordinate... 423.45 sec

[bwa_aln_core] write to the disk... 0.11 sec

[bwa_aln_core] 84934656 sequences have been processed.

[bwa_aln_core] calculate SA coordinate... 416.64 sec

[bwa_aln_core] write to the disk... 0.11 sec

[bwa_aln_core] 85196800 sequences have been processed.

[bwa_aln_core] calculate SA coordinate... 449.71 sec

[bwa_aln_core] write to the disk... 0.11 sec

[bwa_aln_core] 85458944 sequences have been processed.

[bwa_aln_core] calculate SA coordinate... 451.70 sec

[bwa_aln_core] write to the disk... 0.11 sec

[bwa_aln_core] 85721088 sequences have been processed.

[bwa_aln_core] calculate SA coordinate... 907.71 sec

[bwa_aln_core] write to the disk... 0.11 sec

[bwa_aln_core] 85983232 sequences have been processed.

[bwa_aln_core] calculate SA coordinate... 826.50 sec

[bwa_aln_core] write to the disk... 0.11 sec

[bwa_aln_core] 86245376 sequences have been processed.

[bwa_aln_core] calculate SA coordinate... 681.36 sec

[bwa_aln_core] write to the disk... 0.10 sec

[bwa_aln_core] 86507520 sequences have been processed.

[bwa_aln_core] calculate SA coordinate... 535.22 sec

[bwa_aln_core] write to the disk... 0.11 sec

[bwa_aln_core] 86769664 sequences have been processed.

[bwa_aln_core] calculate SA coordinate... 552.97 sec

[bwa_aln_core] write to the disk... 0.11 sec

[bwa_aln_core] 87031808 sequences have been processed.

[bwa_aln_core] calculate SA coordinate... 726.74 sec

[bwa_aln_core] write to the disk... 0.11 sec

[bwa_aln_core] 87293952 sequences have been processed.

[bwa_aln_core] calculate SA coordinate... 504.87 sec

[bwa_aln_core] write to the disk... 0.11 sec

[bwa_aln_core] 87556096 sequences have been processed.

[bwa_aln_core] calculate SA coordinate... 277.73 sec

[bwa_aln_core] write to the disk... 0.11 sec

[bwa_aln_core] 87818240 sequences have been processed.

[bwa_aln_core] calculate SA coordinate... 273.54 sec

[bwa_aln_core] write to the disk... 0.12 sec

[bwa_aln_core] 88080384 sequences have been processed.

[bwa_aln_core] calculate SA coordinate... 274.37 sec

[bwa_aln_core] write to the disk... 0.11 sec

[bwa_aln_core] 88342528 sequences have been processed.

[bwa_aln_core] calculate SA coordinate... 267.80 sec

[bwa_aln_core] write to the disk... 0.12 sec

[bwa_aln_core] 88604672 sequences have been processed.

[bwa_aln_core] calculate SA coordinate... 271.81 sec

[bwa_aln_core] write to the disk... 0.12 sec

[bwa_aln_core] 88866816 sequences have been processed.

[bwa_aln_core] calculate SA coordinate... 266.36 sec

[bwa_aln_core] write to the disk... 0.12 sec

[bwa_aln_core] 89128960 sequences have been processed.

[bwa_aln_core] calculate SA coordinate... 267.39 sec

[bwa_aln_core] write to the disk... 0.12 sec

[bwa_aln_core] 89391104 sequences have been processed.

[bwa_aln_core] calculate SA coordinate... 269.58 sec

[bwa_aln_core] write to the disk... 0.11 sec

[bwa_aln_core] 89653248 sequences have been processed.

[bwa_aln_core] calculate SA coordinate... 272.68 sec

[bwa_aln_core] write to the disk... 0.12 sec

[bwa_aln_core] 89915392 sequences have been processed.

[bwa_aln_core] calculate SA coordinate... 261.17 sec

[bwa_aln_core] write to the disk... 0.11 sec

[bwa_aln_core] 90177536 sequences have been processed.

[bwa_aln_core] calculate SA coordinate... 265.34 sec

[bwa_aln_core] write to the disk... 0.11 sec

[bwa_aln_core] 90439680 sequences have been processed.

[bwa_aln_core] calculate SA coordinate... 269.37 sec

[bwa_aln_core] write to the disk... 0.11 sec

[bwa_aln_core] 90701824 sequences have been processed.

[bwa_aln_core] calculate SA coordinate... 269.06 sec

[bwa_aln_core] write to the disk... 0.12 sec

[bwa_aln_core] 90963968 sequences have been processed.

[bwa_aln_core] calculate SA coordinate... 266.37 sec

[bwa_aln_core] write to the disk... 0.11 sec

[bwa_aln_core] 91226112 sequences have been processed.

[bwa_aln_core] calculate SA coordinate... 276.33 sec

[bwa_aln_core] write to the disk... 0.12 sec

[bwa_aln_core] 91488256 sequences have been processed.

[bwa_aln_core] calculate SA coordinate... 282.22 sec

[bwa_aln_core] write to the disk... 0.12 sec

[bwa_aln_core] 91750400 sequences have been processed.

[bwa_aln_core] calculate SA coordinate... 282.77 sec

[bwa_aln_core] write to the disk... 0.11 sec

[bwa_aln_core] 92012544 sequences have been processed.

[bwa_aln_core] calculate SA coordinate... 273.57 sec

[bwa_aln_core] write to the disk... 0.11 sec

[bwa_aln_core] 92274688 sequences have been processed.

[bwa_aln_core] calculate SA coordinate... 279.91 sec

[bwa_aln_core] write to the disk... 0.11 sec

[bwa_aln_core] 92536832 sequences have been processed.

[bwa_aln_core] calculate SA coordinate... 280.01 sec

[bwa_aln_core] write to the disk... 0.12 sec

[bwa_aln_core] 92798976 sequences have been processed.

[bwa_aln_core] calculate SA coordinate... 283.05 sec

[bwa_aln_core] write to the disk... 0.12 sec

[bwa_aln_core] 93061120 sequences have been processed.

[bwa_aln_core] calculate SA coordinate... 266.51 sec

[bwa_aln_core] write to the disk... 0.10 sec

[bwa_aln_core] 93323264 sequences have been processed.

[bwa_aln_core] calculate SA coordinate... 264.85 sec

[bwa_aln_core] write to the disk... 0.11 sec

[bwa_aln_core] 93585408 sequences have been processed.

[bwa_aln_core] calculate SA coordinate... 267.58 sec

[bwa_aln_core] write to the disk... 0.10 sec

[bwa_aln_core] 93847552 sequences have been processed.

[bwa_aln_core] calculate SA coordinate... 333.29 sec

[bwa_aln_core] write to the disk... 0.10 sec

[bwa_aln_core] 94109696 sequences have been processed.

[bwa_aln_core] calculate SA coordinate... 316.34 sec

[bwa_aln_core] write to the disk... 0.11 sec

[bwa_aln_core] 94371840 sequences have been processed.

[bwa_aln_core] calculate SA coordinate... 522.11 sec

[bwa_aln_core] write to the disk... 0.11 sec

[bwa_aln_core] 94633984 sequences have been processed.

[bwa_aln_core] calculate SA coordinate... 340.26 sec

[bwa_aln_core] write to the disk... 0.11 sec

[bwa_aln_core] 94896128 sequences have been processed.

[bwa_aln_core] calculate SA coordinate... 307.59 sec

[bwa_aln_core] write to the disk... 0.11 sec

[bwa_aln_core] 95158272 sequences have been processed.

[bwa_aln_core] calculate SA coordinate... 333.92 sec

[bwa_aln_core] write to the disk... 0.12 sec

[bwa_aln_core] 95420416 sequences have been processed.

[bwa_aln_core] calculate SA coordinate... 675.41 sec

[bwa_aln_core] write to the disk... 0.10 sec

[bwa_aln_core] 95682560 sequences have been processed.

[bwa_aln_core] calculate SA coordinate... 328.58 sec

[bwa_aln_core] write to the disk... 0.10 sec

[bwa_aln_core] 95944704 sequences have been processed.

[bwa_aln_core] calculate SA coordinate... 1093.54 sec

[bwa_aln_core] write to the disk... 0.10 sec

[bwa_aln_core] 96206848 sequences have been processed.

[bwa_aln_core] calculate SA coordinate... 269.06 sec

[bwa_aln_core] write to the disk... 0.11 sec

[bwa_aln_core] 96468992 sequences have been processed.

[bwa_aln_core] calculate SA coordinate... 263.07 sec

[bwa_aln_core] write to the disk... 0.10 sec

[bwa_aln_core] 96731136 sequences have been processed.

[bwa_aln_core] calculate SA coordinate... 273.89 sec

[bwa_aln_core] write to the disk... 0.11 sec

[bwa_aln_core] 96993280 sequences have been processed.

[bwa_aln_core] calculate SA coordinate... 274.55 sec

[bwa_aln_core] write to the disk... 0.11 sec

[bwa_aln_core] 97255424 sequences have been processed.

[bwa_aln_core] calculate SA coordinate... 262.46 sec

[bwa_aln_core] write to the disk... 0.10 sec

[bwa_aln_core] 97517568 sequences have been processed.

[bwa_aln_core] calculate SA coordinate... 273.03 sec

[bwa_aln_core] write to the disk... 0.10 sec

[bwa_aln_core] 97779712 sequences have been processed.

[bwa_aln_core] calculate SA coordinate... 264.82 sec

[bwa_aln_core] write to the disk... 0.11 sec

[bwa_aln_core] 98041856 sequences have been processed.

[bwa_aln_core] calculate SA coordinate... 272.40 sec

[bwa_aln_core] write to the disk... 0.10 sec

[bwa_aln_core] 98304000 sequences have been processed.

[bwa_aln_core] calculate SA coordinate... 267.79 sec

[bwa_aln_core] write to the disk... 0.11 sec

[bwa_aln_core] 98566144 sequences have been processed.

[bwa_aln_core] calculate SA coordinate... 268.43 sec

[bwa_aln_core] write to the disk... 0.11 sec

[bwa_aln_core] 98828288 sequences have been processed.

[bwa_aln_core] calculate SA coordinate... 269.89 sec

[bwa_aln_core] write to the disk... 0.10 sec

[bwa_aln_core] 99090432 sequences have been processed.

[bwa_aln_core] calculate SA coordinate... 268.94 sec

[bwa_aln_core] write to the disk... 0.10 sec

[bwa_aln_core] 99352576 sequences have been processed.

[bwa_aln_core] calculate SA coordinate... 281.26 sec

[bwa_aln_core] write to the disk... 0.10 sec

[bwa_aln_core] 99614720 sequences have been processed.

[bwa_aln_core] calculate SA coordinate... 263.20 sec

[bwa_aln_core] write to the disk... 0.10 sec

[bwa_aln_core] 99876864 sequences have been processed.

[bwa_aln_core] calculate SA coordinate... 284.53 sec

[bwa_aln_core] write to the disk... 0.10 sec

[bwa_aln_core] 100139008 sequences have been processed.

[bwa_aln_core] calculate SA coordinate... 275.28 sec

[bwa_aln_core] write to the disk... 0.10 sec

[bwa_aln_core] 100401152 sequences have been processed.

[bwa_aln_core] calculate SA coordinate... 273.14 sec

[bwa_aln_core] write to the disk... 0.10 sec

[bwa_aln_core] 100663296 sequences have been processed.

[bwa_aln_core] calculate SA coordinate... 278.98 sec

[bwa_aln_core] write to the disk... 0.12 sec

[bwa_aln_core] 100925440 sequences have been processed.

[bwa_aln_core] calculate SA coordinate... 288.71 sec

[bwa_aln_core] write to the disk... 0.11 sec

[bwa_aln_core] 101187584 sequences have been processed.

[bwa_aln_core] calculate SA coordinate... 304.40 sec

[bwa_aln_core] write to the disk... 0.10 sec

[bwa_aln_core] 101449728 sequences have been processed.

[bwa_aln_core] calculate SA coordinate... 293.20 sec

[bwa_aln_core] write to the disk... 0.10 sec

[bwa_aln_core] 101711872 sequences have been processed.

[bwa_aln_core] calculate SA coordinate... 288.53 sec

[bwa_aln_core] write to the disk... 0.10 sec

[bwa_aln_core] 101974016 sequences have been processed.

[bwa_aln_core] calculate SA coordinate... 281.74 sec

[bwa_aln_core] write to the disk... 0.10 sec

[bwa_aln_core] 102236160 sequences have been processed.

[bwa_aln_core] calculate SA coordinate... 284.44 sec

[bwa_aln_core] write to the disk... 0.11 sec

[bwa_aln_core] 102498304 sequences have been processed.

[bwa_aln_core] calculate SA coordinate... 283.66 sec

[bwa_aln_core] write to the disk... 0.11 sec

[bwa_aln_core] 102760448 sequences have been processed.

[bwa_aln_core] calculate SA coordinate... 353.12 sec

[bwa_aln_core] write to the disk... 0.12 sec

[bwa_aln_core] 103022592 sequences have been processed.

[bwa_aln_core] calculate SA coordinate... 479.63 sec

[bwa_aln_core] write to the disk... 0.11 sec

[bwa_aln_core] 103284736 sequences have been processed.

[bwa_aln_core] calculate SA coordinate... 780.07 sec

[bwa_aln_core] write to the disk... 0.12 sec

[bwa_aln_core] 103546880 sequences have been processed.

[bwa_aln_core] calculate SA coordinate... 1233.41 sec

[bwa_aln_core] write to the disk... 0.11 sec

[bwa_aln_core] 103809024 sequences have been processed.

[bwa_aln_core] calculate SA coordinate... 375.88 sec

[bwa_aln_core] write to the disk... 0.11 sec

[bwa_aln_core] 104071168 sequences have been processed.

[bwa_aln_core] calculate SA coordinate... 307.38 sec

[bwa_aln_core] write to the disk... 0.12 sec

[bwa_aln_core] 104333312 sequences have been processed.

[bwa_aln_core] calculate SA coordinate... 330.28 sec

[bwa_aln_core] write to the disk... 0.12 sec

[bwa_aln_core] 104595456 sequences have been processed.

[bwa_aln_core] calculate SA coordinate... 347.15 sec

[bwa_aln_core] write to the disk... 0.10 sec

[bwa_aln_core] 104857600 sequences have been processed.

[bwa_aln_core] calculate SA coordinate... 989.44 sec

[bwa_aln_core] write to the disk... 0.11 sec

[bwa_aln_core] 105119744 sequences have been processed.

[bwa_aln_core] calculate SA coordinate... 565.35 sec

[bwa_aln_core] write to the disk... 0.11 sec

[bwa_aln_core] 105381888 sequences have been processed.

[bwa_aln_core] calculate SA coordinate... 306.34 sec

[bwa_aln_core] write to the disk... 0.12 sec

[bwa_aln_core] 105644032 sequences have been processed.

[bwa_aln_core] calculate SA coordinate... 711.86 sec

[bwa_aln_core] write to the disk... 0.11 sec

[bwa_aln_core] 105906176 sequences have been processed.

[bwa_aln_core] calculate SA coordinate... 587.97 sec

[bwa_aln_core] write to the disk... 0.11 sec

[bwa_aln_core] 106168320 sequences have been processed.

[bwa_aln_core] calculate SA coordinate... 423.70 sec

[bwa_aln_core] write to the disk... 0.11 sec

[bwa_aln_core] 106430464 sequences have been processed.

[bwa_aln_core] calculate SA coordinate... 554.99 sec

[bwa_aln_core] write to the disk... 0.12 sec

[bwa_aln_core] 106692608 sequences have been processed.

[bwa_aln_core] calculate SA coordinate... 382.30 sec

[bwa_aln_core] write to the disk... 0.11 sec

[bwa_aln_core] 106954752 sequences have been processed.

[bwa_aln_core] calculate SA coordinate... 1404.65 sec

[bwa_aln_core] write to the disk... 0.10 sec

[bwa_aln_core] 107216896 sequences have been processed.

[bwa_aln_core] calculate SA coordinate... 396.08 sec

[bwa_aln_core] write to the disk... 0.11 sec

[bwa_aln_core] 107479040 sequences have been processed.

[bwa_aln_core] calculate SA coordinate... 472.54 sec

[bwa_aln_core] write to the disk... 0.11 sec

[bwa_aln_core] 107741184 sequences have been processed.

[bwa_aln_core] calculate SA coordinate... 361.34 sec

[bwa_aln_core] write to the disk... 0.11 sec

[bwa_aln_core] 108003328 sequences have been processed.

[bwa_aln_core] calculate SA coordinate... 1224.23 sec

[bwa_aln_core] write to the disk... 0.13 sec

[bwa_aln_core] 108265472 sequences have been processed.

[bwa_aln_core] calculate SA coordinate... 446.15 sec

[bwa_aln_core] write to the disk... 0.11 sec

[bwa_aln_core] 108527616 sequences have been processed.

[bwa_aln_core] calculate SA coordinate... 1285.89 sec

[bwa_aln_core] write to the disk... 0.13 sec

[bwa_aln_core] 108789760 sequences have been processed.

[bwa_aln_core] calculate SA coordinate... 333.63 sec

[bwa_aln_core] write to the disk... 0.11 sec

[bwa_aln_core] 109051904 sequences have been processed.

[bwa_aln_core] calculate SA coordinate... 343.62 sec

[bwa_aln_core] write to the disk... 0.11 sec

[bwa_aln_core] 109314048 sequences have been processed.

[bwa_aln_core] calculate SA coordinate... 354.11 sec

[bwa_aln_core] write to the disk... 0.11 sec

[bwa_aln_core] 109576192 sequences have been processed.

[bwa_aln_core] calculate SA coordinate... 334.61 sec

[bwa_aln_core] write to the disk... 0.12 sec

[bwa_aln_core] 109838336 sequences have been processed.

[bwa_aln_core] calculate SA coordinate... 338.79 sec

[bwa_aln_core] write to the disk... 0.12 sec

[bwa_aln_core] 110100480 sequences have been processed.

[bwa_aln_core] calculate SA coordinate... 342.58 sec

[bwa_aln_core] write to the disk... 0.11 sec

[bwa_aln_core] 110362624 sequences have been processed.

[bwa_aln_core] calculate SA coordinate... 344.99 sec

[bwa_aln_core] write to the disk... 0.10 sec

[bwa_aln_core] 110624768 sequences have been processed.

[bwa_aln_core] calculate SA coordinate... 318.12 sec

[bwa_aln_core] write to the disk... 0.12 sec

[bwa_aln_core] 110886912 sequences have been processed.

[bwa_aln_core] calculate SA coordinate... 340.55 sec

[bwa_aln_core] write to the disk... 0.11 sec

[bwa_aln_core] 111149056 sequences have been processed.

[bwa_aln_core] calculate SA coordinate... 332.84 sec

[bwa_aln_core] write to the disk... 0.12 sec

[bwa_aln_core] 111411200 sequences have been processed.

[bwa_aln_core] calculate SA coordinate... 361.53 sec

[bwa_aln_core] write to the disk... 0.11 sec

[bwa_aln_core] 111673344 sequences have been processed.

[bwa_aln_core] calculate SA coordinate... 362.70 sec

[bwa_aln_core] write to the disk... 0.12 sec

[bwa_aln_core] 111935488 sequences have been processed.

[bwa_aln_core] calculate SA coordinate... 371.35 sec

[bwa_aln_core] write to the disk... 0.11 sec

[bwa_aln_core] 112197632 sequences have been processed.

[bwa_aln_core] calculate SA coordinate... 381.57 sec

[bwa_aln_core] write to the disk... 0.11 sec

[bwa_aln_core] 112459776 sequences have been processed.

[bwa_aln_core] calculate SA coordinate... 385.55 sec

[bwa_aln_core] write to the disk... 0.12 sec

[bwa_aln_core] 112721920 sequences have been processed.

[bwa_aln_core] calculate SA coordinate... 395.48 sec

[bwa_aln_core] write to the disk... 0.12 sec

[bwa_aln_core] 112984064 sequences have been processed.

[bwa_aln_core] calculate SA coordinate... 404.19 sec

[bwa_aln_core] write to the disk... 0.11 sec

[bwa_aln_core] 113246208 sequences have been processed.

[bwa_aln_core] calculate SA coordinate... 1305.25 sec

[bwa_aln_core] write to the disk... 0.11 sec

[bwa_aln_core] 113508352 sequences have been processed.

[bwa_aln_core] calculate SA coordinate... 414.43 sec

[bwa_aln_core] write to the disk... 0.11 sec

[bwa_aln_core] 113770496 sequences have been processed.

[bwa_aln_core] calculate SA coordinate... 439.73 sec

[bwa_aln_core] write to the disk... 0.11 sec

[bwa_aln_core] 114032640 sequences have been processed.

[bwa_aln_core] calculate SA coordinate... 417.01 sec

[bwa_aln_core] write to the disk... 0.11 sec

[bwa_aln_core] 114294784 sequences have been processed.

[bwa_aln_core] calculate SA coordinate... 427.10 sec

[bwa_aln_core] write to the disk... 0.10 sec

[bwa_aln_core] 114556928 sequences have been processed.

[bwa_aln_core] calculate SA coordinate... 453.59 sec

[bwa_aln_core] write to the disk... 0.11 sec

[bwa_aln_core] 114819072 sequences have been processed.

[bwa_aln_core] calculate SA coordinate... 471.73 sec

[bwa_aln_core] write to the disk... 0.12 sec

[bwa_aln_core] 115081216 sequences have been processed.

[bwa_aln_core] calculate SA coordinate... 490.35 sec

[bwa_aln_core] write to the disk... 0.11 sec

[bwa_aln_core] 115343360 sequences have been processed.

[bwa_aln_core] calculate SA coordinate... 480.86 sec

[bwa_aln_core] write to the disk... 0.10 sec

[bwa_aln_core] 115605504 sequences have been processed.

[bwa_aln_core] calculate SA coordinate... 474.58 sec

[bwa_aln_core] write to the disk... 0.11 sec

[bwa_aln_core] 115867648 sequences have been processed.

[bwa_aln_core] calculate SA coordinate... 475.87 sec

[bwa_aln_core] write to the disk... 0.12 sec

[bwa_aln_core] 116129792 sequences have been processed.

[bwa_aln_core] calculate SA coordinate... 536.61 sec

[bwa_aln_core] write to the disk... 0.11 sec

[bwa_aln_core] 116391936 sequences have been processed.

[bwa_aln_core] calculate SA coordinate... 746.09 sec

[bwa_aln_core] write to the disk... 0.11 sec

[bwa_aln_core] 116654080 sequences have been processed.

[bwa_aln_core] calculate SA coordinate... 595.84 sec

[bwa_aln_core] write to the disk... 0.10 sec

[bwa_aln_core] 116916224 sequences have been processed.

[bwa_aln_core] calculate SA coordinate... 352.99 sec

[bwa_aln_core] write to the disk... 0.11 sec

[bwa_aln_core] 117178368 sequences have been processed.

[bwa_aln_core] calculate SA coordinate... 444.80 sec

[bwa_aln_core] write to the disk... 0.10 sec

[bwa_aln_core] 117440512 sequences have been processed.

[bwa_aln_core] calculate SA coordinate... 447.55 sec

[bwa_aln_core] write to the disk... 0.12 sec

[bwa_aln_core] 117702656 sequences have been processed.

[bwa_aln_core] calculate SA coordinate... 331.70 sec

[bwa_aln_core] write to the disk... 0.11 sec

[bwa_aln_core] 117964800 sequences have been processed.

[bwa_aln_core] calculate SA coordinate... 392.82 sec

[bwa_aln_core] write to the disk... 0.10 sec

[bwa_aln_core] 118226944 sequences have been processed.

[bwa_aln_core] calculate SA coordinate... 401.37 sec

[bwa_aln_core] write to the disk... 0.10 sec

[bwa_aln_core] 118489088 sequences have been processed.

[bwa_aln_core] calculate SA coordinate... 431.28 sec

[bwa_aln_core] write to the disk... 0.10 sec

[bwa_aln_core] 118751232 sequences have been processed.

[bwa_aln_core] calculate SA coordinate... 323.11 sec

[bwa_aln_core] write to the disk... 0.10 sec

[bwa_aln_core] 119013376 sequences have been processed.

[bwa_aln_core] calculate SA coordinate... 369.92 sec

[bwa_aln_core] write to the disk... 0.11 sec

[bwa_aln_core] 119275520 sequences have been processed.

[bwa_aln_core] calculate SA coordinate... 345.47 sec

[bwa_aln_core] write to the disk... 0.10 sec

[bwa_aln_core] 119537664 sequences have been processed.

[bwa_aln_core] calculate SA coordinate... 356.52 sec

[bwa_aln_core] write to the disk... 0.11 sec

[bwa_aln_core] 119799808 sequences have been processed.

[bwa_aln_core] calculate SA coordinate... 331.81 sec

[bwa_aln_core] write to the disk... 0.11 sec

[bwa_aln_core] 120061952 sequences have been processed.

[bwa_aln_core] calculate SA coordinate... 346.88 sec

[bwa_aln_core] write to the disk... 0.10 sec

[bwa_aln_core] 120324096 sequences have been processed.

[bwa_aln_core] calculate SA coordinate... 406.81 sec

[bwa_aln_core] write to the disk... 0.10 sec

[bwa_aln_core] 120586240 sequences have been processed.

[bwa_aln_core] calculate SA coordinate... 544.00 sec

[bwa_aln_core] write to the disk... 0.10 sec

[bwa_aln_core] 120848384 sequences have been processed.

[bwa_aln_core] calculate SA coordinate... 314.99 sec

[bwa_aln_core] write to the disk... 0.11 sec

[bwa_aln_core] 121110528 sequences have been processed.

[bwa_aln_core] calculate SA coordinate... 319.18 sec

[bwa_aln_core] write to the disk... 0.10 sec

[bwa_aln_core] 121372672 sequences have been processed.

[bwa_aln_core] calculate SA coordinate... 1055.09 sec

[bwa_aln_core] write to the disk... 0.10 sec

[bwa_aln_core] 121634816 sequences have been processed.

[bwa_aln_core] calculate SA coordinate... 1014.94 sec

[bwa_aln_core] write to the disk... 0.11 sec

[bwa_aln_core] 121896960 sequences have been processed.

[bwa_aln_core] calculate SA coordinate... 328.60 sec

[bwa_aln_core] write to the disk... 0.10 sec

[bwa_aln_core] 122159104 sequences have been processed.

[bwa_aln_core] calculate SA coordinate... 307.15 sec

[bwa_aln_core] write to the disk... 0.11 sec

[bwa_aln_core] 122421248 sequences have been processed.

[bwa_aln_core] calculate SA coordinate... 312.36 sec

[bwa_aln_core] write to the disk... 0.10 sec

[bwa_aln_core] 122683392 sequences have been processed.

[bwa_aln_core] calculate SA coordinate... 324.62 sec

[bwa_aln_core] write to the disk... 0.11 sec

[bwa_aln_core] 122945536 sequences have been processed.

[bwa_aln_core] calculate SA coordinate... 314.83 sec

[bwa_aln_core] write to the disk... 0.10 sec

[bwa_aln_core] 123207680 sequences have been processed.

[bwa_aln_core] calculate SA coordinate... 335.25 sec

[bwa_aln_core] write to the disk... 0.09 sec

[bwa_aln_core] 123469824 sequences have been processed.

[bwa_aln_core] calculate SA coordinate... 333.75 sec

[bwa_aln_core] write to the disk... 0.11 sec

[bwa_aln_core] 123731968 sequences have been processed.

[bwa_aln_core] calculate SA coordinate... 354.39 sec

[bwa_aln_core] write to the disk... 0.11 sec

[bwa_aln_core] 123994112 sequences have been processed.

[bwa_aln_core] calculate SA coordinate... 354.88 sec

[bwa_aln_core] write to the disk... 0.11 sec

[bwa_aln_core] 124256256 sequences have been processed.

[bwa_aln_core] calculate SA coordinate... 355.78 sec

[bwa_aln_core] write to the disk... 0.11 sec

[bwa_aln_core] 124518400 sequences have been processed.

[bwa_aln_core] calculate SA coordinate... 342.26 sec

[bwa_aln_core] write to the disk... 0.11 sec

[bwa_aln_core] 124780544 sequences have been processed.

[bwa_aln_core] calculate SA coordinate... 328.11 sec

[bwa_aln_core] write to the disk... 0.09 sec

[bwa_aln_core] 125042688 sequences have been processed.

[bwa_aln_core] calculate SA coordinate... 331.77 sec

[bwa_aln_core] write to the disk... 0.12 sec

[bwa_aln_core] 125304832 sequences have been processed.

[bwa_aln_core] calculate SA coordinate... 329.29 sec

[bwa_aln_core] write to the disk... 0.11 sec

[bwa_aln_core] 125566976 sequences have been processed.

[bwa_aln_core] calculate SA coordinate... 335.47 sec

[bwa_aln_core] write to the disk... 0.11 sec

[bwa_aln_core] 125829120 sequences have been processed.

[bwa_aln_core] calculate SA coordinate... 343.00 sec

[bwa_aln_core] write to the disk... 0.11 sec

[bwa_aln_core] 126091264 sequences have been processed.

[bwa_aln_core] calculate SA coordinate... 684.27 sec

[bwa_aln_core] write to the disk... 0.10 sec

[bwa_aln_core] 126353408 sequences have been processed.

[bwa_aln_core] calculate SA coordinate... 1362.43 sec

[bwa_aln_core] write to the disk... 0.11 sec

[bwa_aln_core] 126615552 sequences have been processed.

[bwa_aln_core] calculate SA coordinate... 407.89 sec

[bwa_aln_core] write to the disk... 0.11 sec

[bwa_aln_core] 126877696 sequences have been processed.

[bwa_aln_core] calculate SA coordinate... 462.37 sec

[bwa_aln_core] write to the disk... 0.11 sec

[bwa_aln_core] 127139840 sequences have been processed.

[bwa_aln_core] calculate SA coordinate... 887.10 sec

[bwa_aln_core] write to the disk... 0.10 sec

[bwa_aln_core] 127401984 sequences have been processed.

[bwa_aln_core] calculate SA coordinate... 343.27 sec

[bwa_aln_core] write to the disk... 0.11 sec

[bwa_aln_core] 127664128 sequences have been processed.

[bwa_aln_core] calculate SA coordinate... 336.89 sec

[bwa_aln_core] write to the disk... 0.10 sec

[bwa_aln_core] 127926272 sequences have been processed.

[bwa_aln_core] calculate SA coordinate... 356.04 sec

[bwa_aln_core] write to the disk... 0.11 sec

[bwa_aln_core] 128188416 sequences have been processed.

[bwa_aln_core] calculate SA coordinate... 347.52 sec

[bwa_aln_core] write to the disk... 0.11 sec

[bwa_aln_core] 128450560 sequences have been processed.

[bwa_aln_core] calculate SA coordinate... 365.69 sec

[bwa_aln_core] write to the disk... 0.12 sec

[bwa_aln_core] 128712704 sequences have been processed.

[bwa_aln_core] calculate SA coordinate... 411.35 sec

[bwa_aln_core] write to the disk... 0.12 sec

[bwa_aln_core] 128974848 sequences have been processed.

[bwa_aln_core] calculate SA coordinate... 350.24 sec

[bwa_aln_core] write to the disk... 0.11 sec

[bwa_aln_core] 129236992 sequences have been processed.

[bwa_aln_core] calculate SA coordinate... 385.25 sec

[bwa_aln_core] write to the disk... 0.10 sec

[bwa_aln_core] 129499136 sequences have been processed.

[bwa_aln_core] calculate SA coordinate... 346.52 sec

[bwa_aln_core] write to the disk... 0.11 sec

[bwa_aln_core] 129761280 sequences have been processed.

[bwa_aln_core] calculate SA coordinate... 385.75 sec

[bwa_aln_core] write to the disk... 0.10 sec

[bwa_aln_core] 130023424 sequences have been processed.

[bwa_aln_core] calculate SA coordinate... 425.29 sec

[bwa_aln_core] write to the disk... 0.12 sec

[bwa_aln_core] 130285568 sequences have been processed.

[bwa_aln_core] calculate SA coordinate... 755.66 sec

[bwa_aln_core] write to the disk... 0.10 sec

[bwa_aln_core] 130547712 sequences have been processed.

[bwa_aln_core] calculate SA coordinate... 390.31 sec

[bwa_aln_core] write to the disk... 0.21 sec

[bwa_aln_core] 130809856 sequences have been processed.

[bwa_aln_core] calculate SA coordinate... 528.81 sec

[bwa_aln_core] write to the disk... 0.11 sec

[bwa_aln_core] 131072000 sequences have been processed.

[bwa_aln_core] calculate SA coordinate... 347.34 sec

[bwa_aln_core] write to the disk... 0.11 sec

[bwa_aln_core] 131334144 sequences have been processed.

[bwa_aln_core] calculate SA coordinate... 578.51 sec

[bwa_aln_core] write to the disk... 0.12 sec

[bwa_aln_core] 131596288 sequences have been processed.

[bwa_aln_core] calculate SA coordinate... 357.12 sec

[bwa_aln_core] write to the disk... 0.12 sec

[bwa_aln_core] 131858432 sequences have been processed.

[bwa_aln_core] calculate SA coordinate... 539.32 sec

[bwa_aln_core] write to the disk... 0.11 sec

[bwa_aln_core] 132120576 sequences have been processed.

[bwa_aln_core] calculate SA coordinate... 357.20 sec

[bwa_aln_core] write to the disk... 0.11 sec

[bwa_aln_core] 132382720 sequences have been processed.

[bwa_aln_core] calculate SA coordinate... 370.40 sec

[bwa_aln_core] write to the disk... 0.12 sec

[bwa_aln_core] 132644864 sequences have been processed.

[bwa_aln_core] calculate SA coordinate... 375.64 sec

[bwa_aln_core] write to the disk... 0.11 sec

[bwa_aln_core] 132907008 sequences have been processed.

[bwa_aln_core] calculate SA coordinate... 371.38 sec

[bwa_aln_core] write to the disk... 0.12 sec

[bwa_aln_core] 133169152 sequences have been processed.

[bwa_aln_core] calculate SA coordinate... 388.37 sec

[bwa_aln_core] write to the disk... 0.12 sec

[bwa_aln_core] 133431296 sequences have been processed.

[bwa_aln_core] calculate SA coordinate... 366.30 sec

[bwa_aln_core] write to the disk... 0.12 sec

[bwa_aln_core] 133693440 sequences have been processed.

[bwa_aln_core] calculate SA coordinate... 372.53 sec

[bwa_aln_core] write to the disk... 0.11 sec

[bwa_aln_core] 133955584 sequences have been processed.

[bwa_aln_core] calculate SA coordinate... 1206.68 sec

[bwa_aln_core] write to the disk... 0.12 sec

[bwa_aln_core] 134217728 sequences have been processed.

[bwa_aln_core] calculate SA coordinate... 384.43 sec

[bwa_aln_core] write to the disk... 0.12 sec

[bwa_aln_core] 134479872 sequences have been processed.

[bwa_aln_core] calculate SA coordinate... 345.16 sec

[bwa_aln_core] write to the disk... 0.11 sec

[bwa_aln_core] 134742016 sequences have been processed.

[bwa_aln_core] calculate SA coordinate... 368.50 sec

[bwa_aln_core] write to the disk... 0.11 sec

[bwa_aln_core] 135004160 sequences have been processed.

[bwa_aln_core] calculate SA coordinate... 396.55 sec

[bwa_aln_core] write to the disk... 0.12 sec

[bwa_aln_core] 135266304 sequences have been processed.

[bwa_aln_core] calculate SA coordinate... 365.26 sec

[bwa_aln_core] write to the disk... 0.12 sec

[bwa_aln_core] 135528448 sequences have been processed.

[bwa_aln_core] calculate SA coordinate... 1144.98 sec

[bwa_aln_core] write to the disk... 0.10 sec

[bwa_aln_core] 135790592 sequences have been processed.

[bwa_aln_core] calculate SA coordinate... 450.32 sec

[bwa_aln_core] write to the disk... 0.11 sec

[bwa_aln_core] 136052736 sequences have been processed.

[bwa_aln_core] calculate SA coordinate... 479.79 sec

[bwa_aln_core] write to the disk... 0.10 sec

[bwa_aln_core] 136314880 sequences have been processed.

[bwa_aln_core] calculate SA coordinate... 479.15 sec

[bwa_aln_core] write to the disk... 0.10 sec

[bwa_aln_core] 136577024 sequences have been processed.

[bwa_aln_core] calculate SA coordinate... 468.16 sec

[bwa_aln_core] write to the disk... 0.11 sec

[bwa_aln_core] 136839168 sequences have been processed.

[bwa_aln_core] calculate SA coordinate... 621.82 sec

[bwa_aln_core] write to the disk... 0.10 sec

[bwa_aln_core] 137101312 sequences have been processed.

[bwa_aln_core] calculate SA coordinate... 449.46 sec

[bwa_aln_core] write to the disk... 0.10 sec

[bwa_aln_core] 137363456 sequences have been processed.

[bwa_aln_core] calculate SA coordinate... 390.49 sec

[bwa_aln_core] write to the disk... 0.10 sec

[bwa_aln_core] 137625600 sequences have been processed.

[bwa_aln_core] calculate SA coordinate... 388.60 sec

[bwa_aln_core] write to the disk... 0.10 sec

[bwa_aln_core] 137887744 sequences have been processed.

[bwa_aln_core] calculate SA coordinate... 724.12 sec

[bwa_aln_core] write to the disk... 0.11 sec

[bwa_aln_core] 138149888 sequences have been processed.

[bwa_aln_core] calculate SA coordinate... 1019.04 sec

[bwa_aln_core] write to the disk... 0.11 sec

[bwa_aln_core] 138412032 sequences have been processed.

[bwa_aln_core] calculate SA coordinate... 408.15 sec

[bwa_aln_core] write to the disk... 0.11 sec

[bwa_aln_core] 138674176 sequences have been processed.

[bwa_aln_core] calculate SA coordinate... 393.53 sec

[bwa_aln_core] write to the disk... 0.11 sec

[bwa_aln_core] 138936320 sequences have been processed.

[bwa_aln_core] calculate SA coordinate... 431.86 sec

[bwa_aln_core] write to the disk... 0.10 sec

[bwa_aln_core] 139198464 sequences have been processed.

[bwa_aln_core] calculate SA coordinate... 418.74 sec

[bwa_aln_core] write to the disk... 0.10 sec

[bwa_aln_core] 139460608 sequences have been processed.

[bwa_aln_core] calculate SA coordinate... 425.12 sec

[bwa_aln_core] write to the disk... 0.11 sec

[bwa_aln_core] 139722752 sequences have been processed.

[bwa_aln_core] calculate SA coordinate... 432.99 sec

[bwa_aln_core] write to the disk... 0.11 sec

[bwa_aln_core] 139984896 sequences have been processed.

[bwa_aln_core] calculate SA coordinate... 841.60 sec

[bwa_aln_core] write to the disk... 0.11 sec

[bwa_aln_core] 140247040 sequences have been processed.

[bwa_aln_core] calculate SA coordinate... 439.87 sec

[bwa_aln_core] write to the disk... 0.10 sec

[bwa_aln_core] 140509184 sequences have been processed.

[bwa_aln_core] calculate SA coordinate... 463.78 sec

[bwa_aln_core] write to the disk... 0.11 sec

[bwa_aln_core] 140771328 sequences have been processed.

[bwa_aln_core] calculate SA coordinate... 535.16 sec

[bwa_aln_core] write to the disk... 0.09 sec

[bwa_aln_core] 141033472 sequences have been processed.

[bwa_aln_core] calculate SA coordinate... 631.08 sec

[bwa_aln_core] write to the disk... 0.10 sec

[bwa_aln_core] 141295616 sequences have been processed.

[bwa_aln_core] calculate SA coordinate... 504.94 sec

[bwa_aln_core] write to the disk... 0.10 sec

[bwa_aln_core] 141557760 sequences have been processed.

[bwa_aln_core] calculate SA coordinate... 536.90 sec

[bwa_aln_core] write to the disk... 0.11 sec

[bwa_aln_core] 141819904 sequences have been processed.

[bwa_aln_core] calculate SA coordinate... 639.13 sec

[bwa_aln_core] write to the disk... 0.11 sec

[bwa_aln_core] 142082048 sequences have been processed.

[bwa_aln_core] calculate SA coordinate... 565.44 sec

[bwa_aln_core] write to the disk... 0.11 sec

[bwa_aln_core] 142344192 sequences have been processed.

[bwa_aln_core] calculate SA coordinate... 912.42 sec

[bwa_aln_core] write to the disk... 0.11 sec

[bwa_aln_core] 142606336 sequences have been processed.

[bwa_aln_core] calculate SA coordinate... 849.68 sec

[bwa_aln_core] write to the disk... 0.11 sec

[bwa_aln_core] 142868480 sequences have been processed.

[bwa_aln_core] calculate SA coordinate... 759.12 sec

[bwa_aln_core] write to the disk... 0.11 sec

[bwa_aln_core] 143130624 sequences have been processed.

[bwa_aln_core] calculate SA coordinate... 570.15 sec

[bwa_aln_core] write to the disk... 0.11 sec

[bwa_aln_core] 143392768 sequences have been processed.

[bwa_aln_core] calculate SA coordinate... 577.33 sec

[bwa_aln_core] write to the disk... 0.11 sec

[bwa_aln_core] 143654912 sequences have been processed.

[bwa_aln_core] calculate SA coordinate... 576.56 sec

[bwa_aln_core] write to the disk... 0.11 sec

[bwa_aln_core] 143917056 sequences have been processed.

[bwa_aln_core] calculate SA coordinate... 567.77 sec

[bwa_aln_core] write to the disk... 0.10 sec

[bwa_aln_core] 144179200 sequences have been processed.

[bwa_aln_core] calculate SA coordinate... 281.19 sec

[bwa_aln_core] write to the disk... 0.11 sec

[bwa_aln_core] 144441344 sequences have been processed.

[bwa_aln_core] calculate SA coordinate... 277.55 sec

[bwa_aln_core] write to the disk... 0.11 sec

[bwa_aln_core] 144703488 sequences have been processed.

[bwa_aln_core] calculate SA coordinate... 294.14 sec

[bwa_aln_core] write to the disk... 0.11 sec

[bwa_aln_core] 144965632 sequences have been processed.

[bwa_aln_core] calculate SA coordinate... 280.23 sec

[bwa_aln_core] write to the disk... 0.11 sec

[bwa_aln_core] 145227776 sequences have been processed.

[bwa_aln_core] calculate SA coordinate... 275.52 sec

[bwa_aln_core] write to the disk... 0.11 sec

[bwa_aln_core] 145489920 sequences have been processed.

[bwa_aln_core] calculate SA coordinate... 281.20 sec

[bwa_aln_core] write to the disk... 0.12 sec

[bwa_aln_core] 145752064 sequences have been processed.

[bwa_aln_core] calculate SA coordinate... 262.33 sec

[bwa_aln_core] write to the disk... 0.11 sec

[bwa_aln_core] 146014208 sequences have been processed.

[bwa_aln_core] calculate SA coordinate... 296.64 sec

[bwa_aln_core] write to the disk... 0.11 sec

[bwa_aln_core] 146276352 sequences have been processed.

[bwa_aln_core] calculate SA coordinate... 273.81 sec

[bwa_aln_core] write to the disk... 0.10 sec

[bwa_aln_core] 146538496 sequences have been processed.

[bwa_aln_core] calculate SA coordinate... 270.20 sec

[bwa_aln_core] write to the disk... 0.11 sec

[bwa_aln_core] 146800640 sequences have been processed.

[bwa_aln_core] calculate SA coordinate... 293.99 sec

[bwa_aln_core] write to the disk... 0.11 sec

[bwa_aln_core] 147062784 sequences have been processed.

[bwa_aln_core] calculate SA coordinate... 287.11 sec

[bwa_aln_core] write to the disk... 0.10 sec

[bwa_aln_core] 147324928 sequences have been processed.

[bwa_aln_core] calculate SA coordinate... 293.98 sec

[bwa_aln_core] write to the disk... 0.10 sec

[bwa_aln_core] 147587072 sequences have been processed.

[bwa_aln_core] calculate SA coordinate... 273.93 sec

[bwa_aln_core] write to the disk... 0.11 sec

[bwa_aln_core] 147849216 sequences have been processed.

[bwa_aln_core] calculate SA coordinate... 284.75 sec

[bwa_aln_core] write to the disk... 0.11 sec

[bwa_aln_core] 148111360 sequences have been processed.

[bwa_aln_core] calculate SA coordinate... 275.75 sec

[bwa_aln_core] write to the disk... 0.10 sec

[bwa_aln_core] 148373504 sequences have been processed.

[bwa_aln_core] calculate SA coordinate... 268.30 sec

[bwa_aln_core] write to the disk... 0.10 sec

[bwa_aln_core] 148635648 sequences have been processed.

[bwa_aln_core] calculate SA coordinate... 286.74 sec

[bwa_aln_core] write to the disk... 0.11 sec

[bwa_aln_core] 148897792 sequences have been processed.

[bwa_aln_core] calculate SA coordinate... 279.22 sec

[bwa_aln_core] write to the disk... 0.12 sec

[bwa_aln_core] 149159936 sequences have been processed.

[bwa_aln_core] calculate SA coordinate... 274.95 sec

[bwa_aln_core] write to the disk... 0.12 sec

[bwa_aln_core] 149422080 sequences have been processed.

[bwa_aln_core] calculate SA coordinate... 294.11 sec

[bwa_aln_core] write to the disk... 0.12 sec

[bwa_aln_core] 149684224 sequences have been processed.

[bwa_aln_core] calculate SA coordinate... 263.57 sec

[bwa_aln_core] write to the disk... 0.11 sec

[bwa_aln_core] 149946368 sequences have been processed.

[bwa_aln_core] calculate SA coordinate... 271.49 sec

[bwa_aln_core] write to the disk... 0.12 sec

[bwa_aln_core] 150208512 sequences have been processed.

[bwa_aln_core] calculate SA coordinate... 263.93 sec

[bwa_aln_core] write to the disk... 0.11 sec

[bwa_aln_core] 150470656 sequences have been processed.

[bwa_aln_core] calculate SA coordinate... 280.70 sec

[bwa_aln_core] write to the disk... 0.12 sec

[bwa_aln_core] 150732800 sequences have been processed.

[bwa_aln_core] calculate SA coordinate... 271.14 sec

[bwa_aln_core] write to the disk... 0.12 sec

[bwa_aln_core] 150994944 sequences have been processed.

[bwa_aln_core] calculate SA coordinate... 283.01 sec

[bwa_aln_core] write to the disk... 0.11 sec

[bwa_aln_core] 151257088 sequences have been processed.

[bwa_aln_core] calculate SA coordinate... 283.96 sec

[bwa_aln_core] write to the disk... 0.11 sec

[bwa_aln_core] 151519232 sequences have been processed.

[bwa_aln_core] calculate SA coordinate... 278.47 sec

[bwa_aln_core] write to the disk... 0.11 sec

[bwa_aln_core] 151781376 sequences have been processed.

[bwa_aln_core] calculate SA coordinate... 1238.23 sec

[bwa_aln_core] write to the disk... 0.10 sec

[bwa_aln_core] 152043520 sequences have been processed.

[bwa_aln_core] calculate SA coordinate... 389.49 sec

[bwa_aln_core] write to the disk... 0.11 sec

[bwa_aln_core] 152305664 sequences have been processed.

[bwa_aln_core] calculate SA coordinate... 1230.32 sec

[bwa_aln_core] write to the disk... 0.10 sec

[bwa_aln_core] 152567808 sequences have been processed.

[bwa_aln_core] calculate SA coordinate... 1552.56 sec

[bwa_aln_core] write to the disk... 0.11 sec

[bwa_aln_core] 152829952 sequences have been processed.

[bwa_aln_core] calculate SA coordinate... 768.14 sec

[bwa_aln_core] write to the disk... 0.12 sec

[bwa_aln_core] 153092096 sequences have been processed.

[bwa_aln_core] calculate SA coordinate... 268.19 sec

[bwa_aln_core] write to the disk... 0.11 sec

[bwa_aln_core] 153354240 sequences have been processed.

[bwa_aln_core] calculate SA coordinate... 269.03 sec

[bwa_aln_core] write to the disk... 0.11 sec

[bwa_aln_core] 153616384 sequences have been processed.

[bwa_aln_core] calculate SA coordinate... 270.89 sec

[bwa_aln_core] write to the disk... 0.11 sec

[bwa_aln_core] 153878528 sequences have been processed.

[bwa_aln_core] calculate SA coordinate... 512.74 sec

[bwa_aln_core] write to the disk... 0.11 sec

[bwa_aln_core] 154140672 sequences have been processed.

[bwa_aln_core] calculate SA coordinate... 263.73 sec

[bwa_aln_core] write to the disk... 0.11 sec

[bwa_aln_core] 154402816 sequences have been processed.

[bwa_aln_core] calculate SA coordinate... 268.06 sec

[bwa_aln_core] write to the disk... 0.10 sec

[bwa_aln_core] 154664960 sequences have been processed.

[bwa_aln_core] calculate SA coordinate... 271.60 sec

[bwa_aln_core] write to the disk... 0.11 sec

[bwa_aln_core] 154927104 sequences have been processed.

[bwa_aln_core] calculate SA coordinate... 259.22 sec

[bwa_aln_core] write to the disk... 0.10 sec

[bwa_aln_core] 155189248 sequences have been processed.

[bwa_aln_core] calculate SA coordinate... 706.26 sec

[bwa_aln_core] write to the disk... 0.10 sec

[bwa_aln_core] 155451392 sequences have been processed.

[bwa_aln_core] calculate SA coordinate... 376.44 sec

[bwa_aln_core] write to the disk... 0.11 sec

[bwa_aln_core] 155713536 sequences have been processed.

[bwa_aln_core] calculate SA coordinate... 441.16 sec

[bwa_aln_core] write to the disk... 0.11 sec

[bwa_aln_core] 155975680 sequences have been processed.

[bwa_aln_core] calculate SA coordinate... 280.13 sec

[bwa_aln_core] write to the disk... 0.12 sec

[bwa_aln_core] 156237824 sequences have been processed.

[bwa_aln_core] calculate SA coordinate... 261.45 sec

[bwa_aln_core] write to the disk... 0.12 sec

[bwa_aln_core] 156499968 sequences have been processed.

[bwa_aln_core] calculate SA coordinate... 280.29 sec

[bwa_aln_core] write to the disk... 0.12 sec

[bwa_aln_core] 156762112 sequences have been processed.

[bwa_aln_core] calculate SA coordinate... 273.04 sec

[bwa_aln_core] write to the disk... 0.12 sec

[bwa_aln_core] 157024256 sequences have been processed.

[bwa_aln_core] calculate SA coordinate... 422.59 sec

[bwa_aln_core] write to the disk... 0.11 sec

[bwa_aln_core] 157286400 sequences have been processed.

[bwa_aln_core] calculate SA coordinate... 279.39 sec

[bwa_aln_core] write to the disk... 0.12 sec

[bwa_aln_core] 157548544 sequences have been processed.

[bwa_aln_core] calculate SA coordinate... 295.51 sec

[bwa_aln_core] write to the disk... 0.13 sec

[bwa_aln_core] 157810688 sequences have been processed.

[bwa_aln_core] calculate SA coordinate... 296.07 sec

[bwa_aln_core] write to the disk... 0.12 sec

[bwa_aln_core] 158072832 sequences have been processed.

[bwa_aln_core] calculate SA coordinate... 291.50 sec

[bwa_aln_core] write to the disk... 0.11 sec

[bwa_aln_core] 158334976 sequences have been processed.

[bwa_aln_core] calculate SA coordinate... 286.52 sec

[bwa_aln_core] write to the disk... 0.11 sec

[bwa_aln_core] 158597120 sequences have been processed.

[bwa_aln_core] calculate SA coordinate... 302.92 sec

[bwa_aln_core] write to the disk... 0.12 sec

[bwa_aln_core] 158859264 sequences have been processed.

[bwa_aln_core] calculate SA coordinate... 293.93 sec

[bwa_aln_core] write to the disk... 0.12 sec

[bwa_aln_core] 159121408 sequences have been processed.

[bwa_aln_core] calculate SA coordinate... 281.35 sec

[bwa_aln_core] write to the disk... 0.12 sec

[bwa_aln_core] 159383552 sequences have been processed.

[bwa_aln_core] calculate SA coordinate... 274.72 sec

[bwa_aln_core] write to the disk... 0.13 sec

[bwa_aln_core] 159645696 sequences have been processed.

[bwa_aln_core] calculate SA coordinate... 280.58 sec

[bwa_aln_core] write to the disk... 0.12 sec

[bwa_aln_core] 159907840 sequences have been processed.

[bwa_aln_core] calculate SA coordinate... 281.59 sec

[bwa_aln_core] write to the disk... 0.12 sec

[bwa_aln_core] 160169984 sequences have been processed.

[bwa_aln_core] calculate SA coordinate... 279.65 sec

[bwa_aln_core] write to the disk... 0.11 sec

[bwa_aln_core] 160432128 sequences have been processed.

[bwa_aln_core] calculate SA coordinate... 314.25 sec

[bwa_aln_core] write to the disk... 0.12 sec

[bwa_aln_core] 160694272 sequences have been processed.

[bwa_aln_core] calculate SA coordinate... 306.62 sec

[bwa_aln_core] write to the disk... 0.12 sec

[bwa_aln_core] 160956416 sequences have been processed.

[bwa_aln_core] calculate SA coordinate... 313.91 sec

[bwa_aln_core] write to the disk... 0.12 sec

[bwa_aln_core] 161218560 sequences have been processed.

[bwa_aln_core] calculate SA coordinate... 300.91 sec

[bwa_aln_core] write to the disk... 0.12 sec

[bwa_aln_core] 161480704 sequences have been processed.

[bwa_aln_core] calculate SA coordinate... 298.08 sec

[bwa_aln_core] write to the disk... 0.14 sec

[bwa_aln_core] 161742848 sequences have been processed.

[bwa_aln_core] calculate SA coordinate... 299.78 sec

[bwa_aln_core] write to the disk... 0.12 sec

[bwa_aln_core] 162004992 sequences have been processed.

[bwa_aln_core] calculate SA coordinate... 311.78 sec

[bwa_aln_core] write to the disk... 0.13 sec

[bwa_aln_core] 162267136 sequences have been processed.

[bwa_aln_core] calculate SA coordinate... 306.81 sec

[bwa_aln_core] write to the disk... 0.12 sec

[bwa_aln_core] 162529280 sequences have been processed.

[bwa_aln_core] calculate SA coordinate... 322.71 sec

[bwa_aln_core] write to the disk... 0.12 sec

[bwa_aln_core] 162791424 sequences have been processed.

[bwa_aln_core] calculate SA coordinate... 325.22 sec

[bwa_aln_core] write to the disk... 0.12 sec

[bwa_aln_core] 163053568 sequences have been processed.

[bwa_aln_core] calculate SA coordinate... 313.77 sec

[bwa_aln_core] write to the disk... 0.11 sec

[bwa_aln_core] 163315712 sequences have been processed.

[bwa_aln_core] calculate SA coordinate... 324.02 sec

[bwa_aln_core] write to the disk... 0.12 sec

[bwa_aln_core] 163577856 sequences have been processed.

[bwa_aln_core] calculate SA coordinate... 299.03 sec

[bwa_aln_core] write to the disk... 0.11 sec

[bwa_aln_core] 163840000 sequences have been processed.

[bwa_aln_core] calculate SA coordinate... 310.26 sec

[bwa_aln_core] write to the disk... 0.12 sec

[bwa_aln_core] 164102144 sequences have been processed.

[bwa_aln_core] calculate SA coordinate... 310.80 sec

[bwa_aln_core] write to the disk... 0.12 sec

[bwa_aln_core] 164364288 sequences have been processed.

[bwa_aln_core] calculate SA coordinate... 315.47 sec

[bwa_aln_core] write to the disk... 0.12 sec

[bwa_aln_core] 164626432 sequences have been processed.

[bwa_aln_core] calculate SA coordinate... 1414.98 sec

[bwa_aln_core] write to the disk... 0.10 sec

[bwa_aln_core] 164888576 sequences have been processed.

[bwa_aln_core] calculate SA coordinate... 1276.43 sec

[bwa_aln_core] write to the disk... 0.10 sec

[bwa_aln_core] 165150720 sequences have been processed.

[bwa_aln_core] calculate SA coordinate... 1053.07 sec

[bwa_aln_core] write to the disk... 0.11 sec

[bwa_aln_core] 165412864 sequences have been processed.

[bwa_aln_core] calculate SA coordinate... 1136.13 sec

[bwa_aln_core] write to the disk... 0.10 sec

[bwa_aln_core] 165675008 sequences have been processed.

[bwa_aln_core] calculate SA coordinate... 736.79 sec

[bwa_aln_core] write to the disk... 0.11 sec

[bwa_aln_core] 165937152 sequences have been processed.

[bwa_aln_core] calculate SA coordinate... 683.54 sec

[bwa_aln_core] write to the disk... 0.10 sec

[bwa_aln_core] 166199296 sequences have been processed.

[bwa_aln_core] calculate SA coordinate... 329.14 sec

[bwa_aln_core] write to the disk... 0.10 sec

[bwa_aln_core] 166461440 sequences have been processed.

[bwa_aln_core] calculate SA coordinate... 325.50 sec

[bwa_aln_core] write to the disk... 0.10 sec

[bwa_aln_core] 166723584 sequences have been processed.

[bwa_aln_core] calculate SA coordinate... 316.50 sec

[bwa_aln_core] write to the disk... 0.10 sec

[bwa_aln_core] 166985728 sequences have been processed.

[bwa_aln_core] calculate SA coordinate... 325.24 sec

[bwa_aln_core] write to the disk... 0.11 sec

[bwa_aln_core] 167247872 sequences have been processed.

[bwa_aln_core] calculate SA coordinate... 452.09 sec

[bwa_aln_core] write to the disk... 0.10 sec

[bwa_aln_core] 167510016 sequences have been processed.

[bwa_aln_core] calculate SA coordinate... 330.86 sec

[bwa_aln_core] write to the disk... 0.11 sec

[bwa_aln_core] 167772160 sequences have been processed.

[bwa_aln_core] calculate SA coordinate... 325.70 sec

[bwa_aln_core] write to the disk... 0.10 sec

[bwa_aln_core] 168034304 sequences have been processed.

[bwa_aln_core] calculate SA coordinate... 327.06 sec

[bwa_aln_core] write to the disk... 0.11 sec

[bwa_aln_core] 168296448 sequences have been processed.

[bwa_aln_core] calculate SA coordinate... 342.52 sec

[bwa_aln_core] write to the disk... 0.09 sec

[bwa_aln_core] 168558592 sequences have been processed.

[bwa_aln_core] calculate SA coordinate... 329.82 sec

[bwa_aln_core] write to the disk... 0.10 sec

[bwa_aln_core] 168820736 sequences have been processed.

[bwa_aln_core] calculate SA coordinate... 355.01 sec

[bwa_aln_core] write to the disk... 0.10 sec

[bwa_aln_core] 169082880 sequences have been processed.

[bwa_aln_core] calculate SA coordinate... 359.66 sec

[bwa_aln_core] write to the disk... 0.11 sec

[bwa_aln_core] 169345024 sequences have been processed.

[bwa_aln_core] calculate SA coordinate... 363.27 sec

[bwa_aln_core] write to the disk... 0.09 sec

[bwa_aln_core] 169607168 sequences have been processed.

[bwa_aln_core] calculate SA coordinate... 365.41 sec

[bwa_aln_core] write to the disk... 0.11 sec

[bwa_aln_core] 169869312 sequences have been processed.

[bwa_aln_core] calculate SA coordinate... 357.57 sec

[bwa_aln_core] write to the disk... 0.10 sec

[bwa_aln_core] 170131456 sequences have been processed.

[bwa_aln_core] calculate SA coordinate... 365.29 sec

[bwa_aln_core] write to the disk... 0.11 sec

[bwa_aln_core] 170393600 sequences have been processed.

[bwa_aln_core] calculate SA coordinate... 386.42 sec

[bwa_aln_core] write to the disk... 0.11 sec

[bwa_aln_core] 170655744 sequences have been processed.

[bwa_aln_core] calculate SA coordinate... 394.54 sec

[bwa_aln_core] write to the disk... 0.11 sec

[bwa_aln_core] 170917888 sequences have been processed.

[bwa_aln_core] calculate SA coordinate... 394.13 sec

[bwa_aln_core] write to the disk... 0.11 sec

[bwa_aln_core] 171180032 sequences have been processed.

[bwa_aln_core] calculate SA coordinate... 397.40 sec

[bwa_aln_core] write to the disk... 0.10 sec

[bwa_aln_core] 171442176 sequences have been processed.

[bwa_aln_core] calculate SA coordinate... 415.21 sec

[bwa_aln_core] write to the disk... 0.11 sec

[bwa_aln_core] 171704320 sequences have been processed.

[bwa_aln_core] calculate SA coordinate... 434.32 sec

[bwa_aln_core] write to the disk... 0.10 sec

[bwa_aln_core] 171966464 sequences have been processed.

[bwa_aln_core] calculate SA coordinate... 437.77 sec

[bwa_aln_core] write to the disk... 0.10 sec

[bwa_aln_core] 172228608 sequences have been processed.

[bwa_aln_core] calculate SA coordinate... 452.78 sec

[bwa_aln_core] write to the disk... 0.10 sec

[bwa_aln_core] 172490752 sequences have been processed.

[bwa_aln_core] calculate SA coordinate... 477.14 sec

[bwa_aln_core] write to the disk... 0.11 sec

[bwa_aln_core] 172752896 sequences have been processed.

[bwa_aln_core] calculate SA coordinate... 344.38 sec

[bwa_aln_core] write to the disk... 0.06 sec

[bwa_aln_core] 172870680 sequences have been processed.

[main] Version: 0.6.2-r126

[main] CMD: /sapfs/actrec/ngs_tools/bwa-0.6.2/bwa aln -t 50 -f /sapfs/actrec/users/pratik/HPVDetector/siha_wgs/SRR1609142_1.sai /sapfs/actrec/users/pratik/HPVDetector/HPVDetector_v1.0/Human_HPV_index_files/human_HPV /sapfs/actrec/users/pratik/down/siha/SRR1609142_1.fastq

[main] Real time: 9812.800 sec; CPU: 357547.385 sec

[bwa_aln] 17bp reads: max_diff = 2

[bwa_aln] 38bp reads: max_diff = 3

[bwa_aln] 64bp reads: max_diff = 4

[bwa_aln] 93bp reads: max_diff = 5

[bwa_aln] 124bp reads: max_diff = 6

[bwa_aln] 157bp reads: max_diff = 7

[bwa_aln] 190bp reads: max_diff = 8

[bwa_aln] 225bp reads: max_diff = 9

[bwa_aln_core] calculate SA coordinate... 232.52 sec

[bwa_aln_core] write to the disk... 0.10 sec

[bwa_aln_core] 262144 sequences have been processed.

[bwa_aln_core] calculate SA coordinate... 242.08 sec

[bwa_aln_core] write to the disk... 0.11 sec

[bwa_aln_core] 524288 sequences have been processed.

[bwa_aln_core] calculate SA coordinate... 396.34 sec

[bwa_aln_core] write to the disk... 0.11 sec

[bwa_aln_core] 786432 sequences have been processed.

[bwa_aln_core] calculate SA coordinate... 544.09 sec

[bwa_aln_core] write to the disk... 0.09 sec

[bwa_aln_core] 1048576 sequences have been processed.

[bwa_aln_core] calculate SA coordinate... 562.11 sec

[bwa_aln_core] write to the disk... 0.10 sec

[bwa_aln_core] 1310720 sequences have been processed.

[bwa_aln_core] calculate SA coordinate... 450.01 sec

[bwa_aln_core] write to the disk... 0.09 sec

[bwa_aln_core] 1572864 sequences have been processed.

[bwa_aln_core] calculate SA coordinate... 496.07 sec

[bwa_aln_core] write to the disk... 0.10 sec

[bwa_aln_core] 1835008 sequences have been processed.

[bwa_aln_core] calculate SA coordinate... 453.56 sec

[bwa_aln_core] write to the disk... 0.11 sec

[bwa_aln_core] 2097152 sequences have been processed.

[bwa_aln_core] calculate SA coordinate... 546.20 sec

[bwa_aln_core] write to the disk... 0.10 sec

[bwa_aln_core] 2359296 sequences have been processed.

[bwa_aln_core] calculate SA coordinate... 493.47 sec

[bwa_aln_core] write to the disk... 0.11 sec

[bwa_aln_core] 2621440 sequences have been processed.

[bwa_aln_core] calculate SA coordinate... 554.52 sec

[bwa_aln_core] write to the disk... 0.11 sec

[bwa_aln_core] 2883584 sequences have been processed.

[bwa_aln_core] calculate SA coordinate... 555.43 sec

[bwa_aln_core] write to the disk... 0.10 sec

[bwa_aln_core] 3145728 sequences have been processed.

[bwa_aln_core] calculate SA coordinate... 503.82 sec

[bwa_aln_core] write to the disk... 0.10 sec

[bwa_aln_core] 3407872 sequences have been processed.

[bwa_aln_core] calculate SA coordinate... 510.52 sec

[bwa_aln_core] write to the disk... 0.11 sec

[bwa_aln_core] 3670016 sequences have been processed.

[bwa_aln_core] calculate SA coordinate... 495.83 sec

[bwa_aln_core] write to the disk... 0.11 sec

[bwa_aln_core] 3932160 sequences have been processed.

[bwa_aln_core] calculate SA coordinate... 497.20 sec

[bwa_aln_core] write to the disk... 0.09 sec

[bwa_aln_core] 4194304 sequences have been processed.

[bwa_aln_core] calculate SA coordinate... 531.61 sec

[bwa_aln_core] write to the disk... 0.11 sec

[bwa_aln_core] 4456448 sequences have been processed.

[bwa_aln_core] calculate SA coordinate... 492.54 sec

[bwa_aln_core] write to the disk... 0.11 sec

[bwa_aln_core] 4718592 sequences have been processed.

[bwa_aln_core] calculate SA coordinate... 473.60 sec

[bwa_aln_core] write to the disk... 0.11 sec

[bwa_aln_core] 4980736 sequences have been processed.

[bwa_aln_core] calculate SA coordinate... 492.42 sec

[bwa_aln_core] write to the disk... 0.11 sec

[bwa_aln_core] 5242880 sequences have been processed.

[bwa_aln_core] calculate SA coordinate... 475.46 sec

[bwa_aln_core] write to the disk... 0.11 sec

[bwa_aln_core] 5505024 sequences have been processed.

[bwa_aln_core] calculate SA coordinate... 519.54 sec

[bwa_aln_core] write to the disk... 0.12 sec

[bwa_aln_core] 5767168 sequences have been processed.

[bwa_aln_core] calculate SA coordinate... 477.77 sec

[bwa_aln_core] write to the disk... 0.11 sec

[bwa_aln_core] 6029312 sequences have been processed.

[bwa_aln_core] calculate SA coordinate... 469.33 sec

[bwa_aln_core] write to the disk... 0.11 sec

[bwa_aln_core] 6291456 sequences have been processed.

[bwa_aln_core] calculate SA coordinate... 469.65 sec

[bwa_aln_core] write to the disk... 0.11 sec

[bwa_aln_core] 6553600 sequences have been processed.

[bwa_aln_core] calculate SA coordinate... 446.38 sec

[bwa_aln_core] write to the disk... 0.11 sec

[bwa_aln_core] 6815744 sequences have been processed.

[bwa_aln_core] calculate SA coordinate... 482.18 sec

[bwa_aln_core] write to the disk... 0.11 sec

[bwa_aln_core] 7077888 sequences have been processed.

[bwa_aln_core] calculate SA coordinate... 471.09 sec

[bwa_aln_core] write to the disk... 0.11 sec

[bwa_aln_core] 7340032 sequences have been processed.

[bwa_aln_core] calculate SA coordinate... 482.63 sec

[bwa_aln_core] write to the disk... 0.10 sec

[bwa_aln_core] 7602176 sequences have been processed.

[bwa_aln_core] calculate SA coordinate... 492.22 sec

[bwa_aln_core] write to the disk... 0.11 sec

[bwa_aln_core] 7864320 sequences have been processed.

[bwa_aln_core] calculate SA coordinate... 471.26 sec

[bwa_aln_core] write to the disk... 0.11 sec

[bwa_aln_core] 8126464 sequences have been processed.

[bwa_aln_core] calculate SA coordinate... 434.79 sec

[bwa_aln_core] write to the disk... 0.13 sec

[bwa_aln_core] 8388608 sequences have been processed.

[bwa_aln_core] calculate SA coordinate... 520.34 sec

[bwa_aln_core] write to the disk... 0.12 sec

[bwa_aln_core] 8650752 sequences have been processed.

[bwa_aln_core] calculate SA coordinate... 506.14 sec

[bwa_aln_core] write to the disk... 0.11 sec

[bwa_aln_core] 8912896 sequences have been processed.

[bwa_aln_core] calculate SA coordinate... 478.23 sec

[bwa_aln_core] write to the disk... 0.12 sec

[bwa_aln_core] 9175040 sequences have been processed.

[bwa_aln_core] calculate SA coordinate... 454.11 sec

[bwa_aln_core] write to the disk... 0.12 sec

[bwa_aln_core] 9437184 sequences have been processed.

[bwa_aln_core] calculate SA coordinate... 537.67 sec

[bwa_aln_core] write to the disk... 0.10 sec

[bwa_aln_core] 9699328 sequences have been processed.

[bwa_aln_core] calculate SA coordinate... 483.76 sec

[bwa_aln_core] write to the disk... 0.11 sec

[bwa_aln_core] 9961472 sequences have been processed.

[bwa_aln_core] calculate SA coordinate... 478.04 sec

[bwa_aln_core] write to the disk... 0.12 sec

[bwa_aln_core] 10223616 sequences have been processed.

[bwa_aln_core] calculate SA coordinate... 535.93 sec

[bwa_aln_core] write to the disk... 0.12 sec

[bwa_aln_core] 10485760 sequences have been processed.

[bwa_aln_core] calculate SA coordinate... 444.07 sec

[bwa_aln_core] write to the disk... 0.12 sec

[bwa_aln_core] 10747904 sequences have been processed.

[bwa_aln_core] calculate SA coordinate... 499.61 sec

[bwa_aln_core] write to the disk... 0.11 sec

[bwa_aln_core] 11010048 sequences have been processed.

[bwa_aln_core] calculate SA coordinate... 483.17 sec

[bwa_aln_core] write to the disk... 0.11 sec

[bwa_aln_core] 11272192 sequences have been processed.

[bwa_aln_core] calculate SA coordinate... 495.42 sec

[bwa_aln_core] write to the disk... 0.11 sec

[bwa_aln_core] 11534336 sequences have been processed.

[bwa_aln_core] calculate SA coordinate... 446.47 sec

[bwa_aln_core] write to the disk... 0.10 sec

[bwa_aln_core] 11796480 sequences have been processed.

[bwa_aln_core] calculate SA coordinate... 508.91 sec

[bwa_aln_core] write to the disk... 0.11 sec

[bwa_aln_core] 12058624 sequences have been processed.

[bwa_aln_core] calculate SA coordinate... 546.38 sec

[bwa_aln_core] write to the disk... 0.11 sec

[bwa_aln_core] 12320768 sequences have been processed.

[bwa_aln_core] calculate SA coordinate... 552.31 sec

[bwa_aln_core] write to the disk... 0.13 sec

[bwa_aln_core] 12582912 sequences have been processed.

[bwa_aln_core] calculate SA coordinate... 504.41 sec

[bwa_aln_core] write to the disk... 0.11 sec

[bwa_aln_core] 12845056 sequences have been processed.

[bwa_aln_core] calculate SA coordinate... 478.11 sec

[bwa_aln_core] write to the disk... 0.12 sec

[bwa_aln_core] 13107200 sequences have been processed.

[bwa_aln_core] calculate SA coordinate... 498.98 sec

[bwa_aln_core] write to the disk... 0.11 sec

[bwa_aln_core] 13369344 sequences have been processed.

[bwa_aln_core] calculate SA coordinate... 518.58 sec

[bwa_aln_core] write to the disk... 0.13 sec

[bwa_aln_core] 13631488 sequences have been processed.

[bwa_aln_core] calculate SA coordinate... 566.42 sec

[bwa_aln_core] write to the disk... 0.10 sec

[bwa_aln_core] 13893632 sequences have been processed.

[bwa_aln_core] calculate SA coordinate... 553.36 sec

[bwa_aln_core] write to the disk... 0.10 sec

[bwa_aln_core] 14155776 sequences have been processed.

[bwa_aln_core] calculate SA coordinate... 541.40 sec

[bwa_aln_core] write to the disk... 0.11 sec

[bwa_aln_core] 14417920 sequences have been processed.

[bwa_aln_core] calculate SA coordinate... 468.81 sec

[bwa_aln_core] write to the disk... 0.11 sec

[bwa_aln_core] 14680064 sequences have been processed.

[bwa_aln_core] calculate SA coordinate... 540.95 sec

[bwa_aln_core] write to the disk... 0.09 sec

[bwa_aln_core] 14942208 sequences have been processed.

[bwa_aln_core] calculate SA coordinate... 527.94 sec

[bwa_aln_core] write to the disk... 0.09 sec

[bwa_aln_core] 15204352 sequences have been processed.

[bwa_aln_core] calculate SA coordinate... 534.05 sec

[bwa_aln_core] write to the disk... 0.11 sec

[bwa_aln_core] 15466496 sequences have been processed.

[bwa_aln_core] calculate SA coordinate... 509.73 sec

[bwa_aln_core] write to the disk... 0.09 sec

[bwa_aln_core] 15728640 sequences have been processed.

[bwa_aln_core] calculate SA coordinate... 496.47 sec

[bwa_aln_core] write to the disk... 0.11 sec

[bwa_aln_core] 15990784 sequences have been processed.

[bwa_aln_core] calculate SA coordinate... 555.32 sec

[bwa_aln_core] write to the disk... 0.11 sec

[bwa_aln_core] 16252928 sequences have been processed.

[bwa_aln_core] calculate SA coordinate... 525.90 sec

[bwa_aln_core] write to the disk... 0.10 sec

[bwa_aln_core] 16515072 sequences have been processed.

[bwa_aln_core] calculate SA coordinate... 508.56 sec

[bwa_aln_core] write to the disk... 0.10 sec

[bwa_aln_core] 16777216 sequences have been processed.

[bwa_aln_core] calculate SA coordinate... 445.30 sec

[bwa_aln_core] write to the disk... 0.11 sec

[bwa_aln_core] 17039360 sequences have been processed.

[bwa_aln_core] calculate SA coordinate... 444.48 sec

[bwa_aln_core] write to the disk... 0.09 sec

[bwa_aln_core] 17301504 sequences have been processed.

[bwa_aln_core] calculate SA coordinate... 526.15 sec

[bwa_aln_core] write to the disk... 0.11 sec

[bwa_aln_core] 17563648 sequences have been processed.

[bwa_aln_core] calculate SA coordinate... 489.01 sec

[bwa_aln_core] write to the disk... 0.10 sec

[bwa_aln_core] 17825792 sequences have been processed.

[bwa_aln_core] calculate SA coordinate... 497.34 sec

[bwa_aln_core] write to the disk... 0.10 sec

[bwa_aln_core] 18087936 sequences have been processed.

[bwa_aln_core] calculate SA coordinate... 487.42 sec

[bwa_aln_core] write to the disk... 0.10 sec

[bwa_aln_core] 18350080 sequences have been processed.

[bwa_aln_core] calculate SA coordinate... 467.63 sec

[bwa_aln_core] write to the disk... 0.10 sec

[bwa_aln_core] 18612224 sequences have been processed.

[bwa_aln_core] calculate SA coordinate... 505.61 sec

[bwa_aln_core] write to the disk... 0.09 sec

[bwa_aln_core] 18874368 sequences have been processed.

[bwa_aln_core] calculate SA coordinate... 500.20 sec

[bwa_aln_core] write to the disk... 0.10 sec

[bwa_aln_core] 19136512 sequences have been processed.

[bwa_aln_core] calculate SA coordinate... 496.59 sec

[bwa_aln_core] write to the disk... 0.10 sec

[bwa_aln_core] 19398656 sequences have been processed.

[bwa_aln_core] calculate SA coordinate... 502.76 sec

[bwa_aln_core] write to the disk... 0.11 sec

[bwa_aln_core] 19660800 sequences have been processed.

[bwa_aln_core] calculate SA coordinate... 474.77 sec

[bwa_aln_core] write to the disk... 0.11 sec

[bwa_aln_core] 19922944 sequences have been processed.

[bwa_aln_core] calculate SA coordinate... 446.00 sec

[bwa_aln_core] write to the disk... 0.11 sec

[bwa_aln_core] 20185088 sequences have been processed.

[bwa_aln_core] calculate SA coordinate... 481.88 sec

[bwa_aln_core] write to the disk... 0.12 sec

[bwa_aln_core] 20447232 sequences have been processed.

[bwa_aln_core] calculate SA coordinate... 416.16 sec

[bwa_aln_core] write to the disk... 0.11 sec

[bwa_aln_core] 20709376 sequences have been processed.

[bwa_aln_core] calculate SA coordinate... 429.77 sec

[bwa_aln_core] write to the disk... 0.10 sec

[bwa_aln_core] 20971520 sequences have been processed.

[bwa_aln_core] calculate SA coordinate... 464.15 sec

[bwa_aln_core] write to the disk... 0.10 sec

[bwa_aln_core] 21233664 sequences have been processed.

[bwa_aln_core] calculate SA coordinate... 494.86 sec

[bwa_aln_core] write to the disk... 0.10 sec

[bwa_aln_core] 21495808 sequences have been processed.

[bwa_aln_core] calculate SA coordinate... 505.02 sec

[bwa_aln_core] write to the disk... 0.11 sec

[bwa_aln_core] 21757952 sequences have been processed.

[bwa_aln_core] calculate SA coordinate... 519.62 sec

[bwa_aln_core] write to the disk... 0.10 sec

[bwa_aln_core] 22020096 sequences have been processed.

[bwa_aln_core] calculate SA coordinate... 480.60 sec

[bwa_aln_core] write to the disk... 0.11 sec

[bwa_aln_core] 22282240 sequences have been processed.

[bwa_aln_core] calculate SA coordinate... 518.73 sec

[bwa_aln_core] write to the disk... 0.11 sec

[bwa_aln_core] 22544384 sequences have been processed.

[bwa_aln_core] calculate SA coordinate... 484.56 sec

[bwa_aln_core] write to the disk... 0.11 sec

[bwa_aln_core] 22806528 sequences have been processed.

[bwa_aln_core] calculate SA coordinate... 450.45 sec

[bwa_aln_core] write to the disk... 0.10 sec

[bwa_aln_core] 23068672 sequences have been processed.

[bwa_aln_core] calculate SA coordinate... 479.30 sec

[bwa_aln_core] write to the disk... 0.10 sec

[bwa_aln_core] 23330816 sequences have been processed.

[bwa_aln_core] calculate SA coordinate... 480.21 sec

[bwa_aln_core] write to the disk... 0.11 sec

[bwa_aln_core] 23592960 sequences have been processed.

[bwa_aln_core] calculate SA coordinate... 475.10 sec

[bwa_aln_core] write to the disk... 0.10 sec

[bwa_aln_core] 23855104 sequences have been processed.

[bwa_aln_core] calculate SA coordinate... 544.10 sec

[bwa_aln_core] write to the disk... 0.10 sec

[bwa_aln_core] 24117248 sequences have been processed.

[bwa_aln_core] calculate SA coordinate... 526.03 sec

[bwa_aln_core] write to the disk... 0.10 sec

[bwa_aln_core] 24379392 sequences have been processed.

[bwa_aln_core] calculate SA coordinate... 512.15 sec

[bwa_aln_core] write to the disk... 0.11 sec

[bwa_aln_core] 24641536 sequences have been processed.

[bwa_aln_core] calculate SA coordinate... 467.85 sec

[bwa_aln_core] write to the disk... 0.10 sec

[bwa_aln_core] 24903680 sequences have been processed.

[bwa_aln_core] calculate SA coordinate... 469.01 sec

[bwa_aln_core] write to the disk... 0.10 sec

[bwa_aln_core] 25165824 sequences have been processed.

[bwa_aln_core] calculate SA coordinate... 513.77 sec

[bwa_aln_core] write to the disk... 0.10 sec

[bwa_aln_core] 25427968 sequences have been processed.

[bwa_aln_core] calculate SA coordinate... 499.14 sec

[bwa_aln_core] write to the disk... 0.09 sec

[bwa_aln_core] 25690112 sequences have been processed.

[bwa_aln_core] calculate SA coordinate... 477.60 sec

[bwa_aln_core] write to the disk... 0.10 sec

[bwa_aln_core] 25952256 sequences have been processed.

[bwa_aln_core] calculate SA coordinate... 464.33 sec

[bwa_aln_core] write to the disk... 0.10 sec

[bwa_aln_core] 26214400 sequences have been processed.

[bwa_aln_core] calculate SA coordinate... 501.90 sec

[bwa_aln_core] write to the disk... 0.10 sec

[bwa_aln_core] 26476544 sequences have been processed.

[bwa_aln_core] calculate SA coordinate... 486.81 sec

[bwa_aln_core] write to the disk... 0.10 sec

[bwa_aln_core] 26738688 sequences have been processed.

[bwa_aln_core] calculate SA coordinate... 501.30 sec

[bwa_aln_core] write to the disk... 0.11 sec

[bwa_aln_core] 27000832 sequences have been processed.

[bwa_aln_core] calculate SA coordinate... 500.90 sec

[bwa_aln_core] write to the disk... 0.10 sec

[bwa_aln_core] 27262976 sequences have been processed.

[bwa_aln_core] calculate SA coordinate... 583.10 sec

[bwa_aln_core] write to the disk... 0.09 sec

[bwa_aln_core] 27525120 sequences have been processed.

[bwa_aln_core] calculate SA coordinate... 560.25 sec

[bwa_aln_core] write to the disk... 0.09 sec

[bwa_aln_core] 27787264 sequences have been processed.

[bwa_aln_core] calculate SA coordinate... 530.02 sec

[bwa_aln_core] write to the disk... 0.09 sec

[bwa_aln_core] 28049408 sequences have been processed.

[bwa_aln_core] calculate SA coordinate... 504.71 sec

[bwa_aln_core] write to the disk... 0.10 sec

[bwa_aln_core] 28311552 sequences have been processed.

[bwa_aln_core] calculate SA coordinate... 470.43 sec

[bwa_aln_core] write to the disk... 0.12 sec

[bwa_aln_core] 28573696 sequences have been processed.

[bwa_aln_core] calculate SA coordinate... 513.70 sec

[bwa_aln_core] write to the disk... 0.10 sec

[bwa_aln_core] 28835840 sequences have been processed.

[bwa_aln_core] calculate SA coordinate... 441.34 sec

[bwa_aln_core] write to the disk... 0.11 sec

[bwa_aln_core] 29097984 sequences have been processed.

[bwa_aln_core] calculate SA coordinate... 448.87 sec

[bwa_aln_core] write to the disk... 0.10 sec

[bwa_aln_core] 29360128 sequences have been processed.

[bwa_aln_core] calculate SA coordinate... 424.08 sec

[bwa_aln_core] write to the disk... 0.13 sec

[bwa_aln_core] 29622272 sequences have been processed.

[bwa_aln_core] calculate SA coordinate... 481.26 sec

[bwa_aln_core] write to the disk... 0.10 sec

[bwa_aln_core] 29884416 sequences have been processed.

[bwa_aln_core] calculate SA coordinate... 438.99 sec

[bwa_aln_core] write to the disk... 0.11 sec

[bwa_aln_core] 30146560 sequences have been processed.

[bwa_aln_core] calculate SA coordinate... 518.33 sec

[bwa_aln_core] write to the disk... 0.12 sec

[bwa_aln_core] 30408704 sequences have been processed.

[bwa_aln_core] calculate SA coordinate... 477.27 sec

[bwa_aln_core] write to the disk... 0.11 sec

[bwa_aln_core] 30670848 sequences have been processed.

[bwa_aln_core] calculate SA coordinate... 523.95 sec

[bwa_aln_core] write to the disk... 0.11 sec

[bwa_aln_core] 30932992 sequences have been processed.

[bwa_aln_core] calculate SA coordinate... 525.27 sec

[bwa_aln_core] write to the disk... 0.11 sec

[bwa_aln_core] 31195136 sequences have been processed.

[bwa_aln_core] calculate SA coordinate... 439.57 sec

[bwa_aln_core] write to the disk... 0.11 sec

[bwa_aln_core] 31457280 sequences have been processed.

[bwa_aln_core] calculate SA coordinate... 477.74 sec

[bwa_aln_core] write to the disk... 0.11 sec

[bwa_aln_core] 31719424 sequences have been processed.

[bwa_aln_core] calculate SA coordinate... 502.81 sec

[bwa_aln_core] write to the disk... 0.10 sec

[bwa_aln_core] 31981568 sequences have been processed.

[bwa_aln_core] calculate SA coordinate... 481.01 sec

[bwa_aln_core] write to the disk... 0.10 sec

[bwa_aln_core] 32243712 sequences have been processed.

[bwa_aln_core] calculate SA coordinate... 570.96 sec

[bwa_aln_core] write to the disk... 0.10 sec

[bwa_aln_core] 32505856 sequences have been processed.

[bwa_aln_core] calculate SA coordinate... 483.03 sec

[bwa_aln_core] write to the disk... 0.10 sec

[bwa_aln_core] 32768000 sequences have been processed.

[bwa_aln_core] calculate SA coordinate... 494.44 sec

[bwa_aln_core] write to the disk... 0.10 sec

[bwa_aln_core] 33030144 sequences have been processed.

[bwa_aln_core] calculate SA coordinate... 490.53 sec

[bwa_aln_core] write to the disk... 0.11 sec

[bwa_aln_core] 33292288 sequences have been processed.

[bwa_aln_core] calculate SA coordinate... 496.38 sec

[bwa_aln_core] write to the disk... 0.10 sec

[bwa_aln_core] 33554432 sequences have been processed.

[bwa_aln_core] calculate SA coordinate... 481.24 sec

[bwa_aln_core] write to the disk... 0.11 sec

[bwa_aln_core] 33816576 sequences have been processed.

[bwa_aln_core] calculate SA coordinate... 493.19 sec

[bwa_aln_core] write to the disk... 0.10 sec

[bwa_aln_core] 34078720 sequences have been processed.

[bwa_aln_core] calculate SA coordinate... 473.32 sec

[bwa_aln_core] write to the disk... 0.10 sec

[bwa_aln_core] 34340864 sequences have been processed.

[bwa_aln_core] calculate SA coordinate... 528.27 sec

[bwa_aln_core] write to the disk... 0.09 sec

[bwa_aln_core] 34603008 sequences have been processed.

[bwa_aln_core] calculate SA coordinate... 532.80 sec

[bwa_aln_core] write to the disk... 0.11 sec

[bwa_aln_core] 34865152 sequences have been processed.

[bwa_aln_core] calculate SA coordinate... 536.76 sec

[bwa_aln_core] write to the disk... 0.11 sec

[bwa_aln_core] 35127296 sequences have been processed.

[bwa_aln_core] calculate SA coordinate... 550.42 sec

[bwa_aln_core] write to the disk... 0.11 sec

[bwa_aln_core] 35389440 sequences have been processed.

[bwa_aln_core] calculate SA coordinate... 468.30 sec

[bwa_aln_core] write to the disk... 0.11 sec

[bwa_aln_core] 35651584 sequences have been processed.

[bwa_aln_core] calculate SA coordinate... 495.11 sec

[bwa_aln_core] write to the disk... 0.11 sec

[bwa_aln_core] 35913728 sequences have been processed.

[bwa_aln_core] calculate SA coordinate... 500.40 sec

[bwa_aln_core] write to the disk... 0.10 sec

[bwa_aln_core] 36175872 sequences have been processed.

[bwa_aln_core] calculate SA coordinate... 526.65 sec

[bwa_aln_core] write to the disk... 0.11 sec

[bwa_aln_core] 36438016 sequences have been processed.

[bwa_aln_core] calculate SA coordinate... 442.22 sec

[bwa_aln_core] write to the disk... 0.10 sec

[bwa_aln_core] 36700160 sequences have been processed.

[bwa_aln_core] calculate SA coordinate... 482.81 sec

[bwa_aln_core] write to the disk... 0.10 sec

[bwa_aln_core] 36962304 sequences have been processed.

[bwa_aln_core] calculate SA coordinate... 457.76 sec

[bwa_aln_core] write to the disk... 0.10 sec

[bwa_aln_core] 37224448 sequences have been processed.

[bwa_aln_core] calculate SA coordinate... 457.13 sec

[bwa_aln_core] write to the disk... 0.10 sec

[bwa_aln_core] 37486592 sequences have been processed.

[bwa_aln_core] calculate SA coordinate... 560.51 sec

[bwa_aln_core] write to the disk... 0.10 sec

[bwa_aln_core] 37748736 sequences have been processed.

[bwa_aln_core] calculate SA coordinate... 497.14 sec

[bwa_aln_core] write to the disk... 0.10 sec

[bwa_aln_core] 38010880 sequences have been processed.

[bwa_aln_core] calculate SA coordinate... 484.04 sec

[bwa_aln_core] write to the disk... 0.10 sec

[bwa_aln_core] 38273024 sequences have been processed.

[bwa_aln_core] calculate SA coordinate... 581.88 sec

[bwa_aln_core] write to the disk... 0.09 sec

[bwa_aln_core] 38535168 sequences have been processed.

[bwa_aln_core] calculate SA coordinate... 448.55 sec

[bwa_aln_core] write to the disk... 0.11 sec

[bwa_aln_core] 38797312 sequences have been processed.

[bwa_aln_core] calculate SA coordinate... 494.83 sec

[bwa_aln_core] write to the disk... 0.11 sec

[bwa_aln_core] 39059456 sequences have been processed.

[bwa_aln_core] calculate SA coordinate... 474.45 sec

[bwa_aln_core] write to the disk... 0.11 sec

[bwa_aln_core] 39321600 sequences have been processed.

[bwa_aln_core] calculate SA coordinate... 469.16 sec

[bwa_aln_core] write to the disk... 0.10 sec

[bwa_aln_core] 39583744 sequences have been processed.

[bwa_aln_core] calculate SA coordinate... 465.74 sec

[bwa_aln_core] write to the disk... 0.10 sec

[bwa_aln_core] 39845888 sequences have been processed.

[bwa_aln_core] calculate SA coordinate... 471.38 sec

[bwa_aln_core] write to the disk... 0.10 sec

[bwa_aln_core] 40108032 sequences have been processed.

[bwa_aln_core] calculate SA coordinate... 495.84 sec

[bwa_aln_core] write to the disk... 0.09 sec

[bwa_aln_core] 40370176 sequences have been processed.

[bwa_aln_core] calculate SA coordinate... 436.27 sec

[bwa_aln_core] write to the disk... 0.10 sec

[bwa_aln_core] 40632320 sequences have been processed.

[bwa_aln_core] calculate SA coordinate... 485.08 sec

[bwa_aln_core] write to the disk... 0.11 sec

[bwa_aln_core] 40894464 sequences have been processed.

[bwa_aln_core] calculate SA coordinate... 510.42 sec

[bwa_aln_core] write to the disk... 0.10 sec

[bwa_aln_core] 41156608 sequences have been processed.

[bwa_aln_core] calculate SA coordinate... 520.10 sec

[bwa_aln_core] write to the disk... 0.10 sec

[bwa_aln_core] 41418752 sequences have been processed.

[bwa_aln_core] calculate SA coordinate... 516.48 sec

[bwa_aln_core] write to the disk... 0.11 sec

[bwa_aln_core] 41680896 sequences have been processed.

[bwa_aln_core] calculate SA coordinate... 472.70 sec

[bwa_aln_core] write to the disk... 0.12 sec

[bwa_aln_core] 41943040 sequences have been processed.

[bwa_aln_core] calculate SA coordinate... 497.45 sec

[bwa_aln_core] write to the disk... 0.12 sec

[bwa_aln_core] 42205184 sequences have been processed.

[bwa_aln_core] calculate SA coordinate... 464.54 sec

[bwa_aln_core] write to the disk... 0.11 sec

[bwa_aln_core] 42467328 sequences have been processed.

[bwa_aln_core] calculate SA coordinate... 462.81 sec

[bwa_aln_core] write to the disk... 0.11 sec

[bwa_aln_core] 42729472 sequences have been processed.

[bwa_aln_core] calculate SA coordinate... 482.05 sec

[bwa_aln_core] write to the disk... 0.11 sec

[bwa_aln_core] 42991616 sequences have been processed.

[bwa_aln_core] calculate SA coordinate... 467.62 sec

[bwa_aln_core] write to the disk... 0.11 sec

[bwa_aln_core] 43253760 sequences have been processed.

[bwa_aln_core] calculate SA coordinate... 474.04 sec

[bwa_aln_core] write to the disk... 0.11 sec

[bwa_aln_core] 43515904 sequences have been processed.

[bwa_aln_core] calculate SA coordinate... 486.08 sec

[bwa_aln_core] write to the disk... 0.12 sec

[bwa_aln_core] 43778048 sequences have been processed.

[bwa_aln_core] calculate SA coordinate... 459.96 sec

[bwa_aln_core] write to the disk... 0.11 sec

[bwa_aln_core] 44040192 sequences have been processed.

[bwa_aln_core] calculate SA coordinate... 494.83 sec

[bwa_aln_core] write to the disk... 0.11 sec

[bwa_aln_core] 44302336 sequences have been processed.

[bwa_aln_core] calculate SA coordinate... 542.29 sec

[bwa_aln_core] write to the disk... 0.10 sec

[bwa_aln_core] 44564480 sequences have been processed.

[bwa_aln_core] calculate SA coordinate... 473.75 sec

[bwa_aln_core] write to the disk... 0.11 sec

[bwa_aln_core] 44826624 sequences have been processed.

[bwa_aln_core] calculate SA coordinate... 570.30 sec

[bwa_aln_core] write to the disk... 0.11 sec

[bwa_aln_core] 45088768 sequences have been processed.

[bwa_aln_core] calculate SA coordinate... 503.94 sec

[bwa_aln_core] write to the disk... 0.10 sec

[bwa_aln_core] 45350912 sequences have been processed.

[bwa_aln_core] calculate SA coordinate... 442.15 sec

[bwa_aln_core] write to the disk... 0.11 sec

[bwa_aln_core] 45613056 sequences have been processed.

[bwa_aln_core] calculate SA coordinate... 505.78 sec

[bwa_aln_core] write to the disk... 0.10 sec

[bwa_aln_core] 45875200 sequences have been processed.

[bwa_aln_core] calculate SA coordinate... 465.38 sec

[bwa_aln_core] write to the disk... 0.11 sec

[bwa_aln_core] 46137344 sequences have been processed.

[bwa_aln_core] calculate SA coordinate... 485.12 sec

[bwa_aln_core] write to the disk... 0.11 sec

[bwa_aln_core] 46399488 sequences have been processed.

[bwa_aln_core] calculate SA coordinate... 499.73 sec

[bwa_aln_core] write to the disk... 0.11 sec

[bwa_aln_core] 46661632 sequences have been processed.

[bwa_aln_core] calculate SA coordinate... 474.34 sec

[bwa_aln_core] write to the disk... 0.11 sec

[bwa_aln_core] 46923776 sequences have been processed.

[bwa_aln_core] calculate SA coordinate... 487.40 sec

[bwa_aln_core] write to the disk... 0.10 sec

[bwa_aln_core] 47185920 sequences have been processed.

[bwa_aln_core] calculate SA coordinate... 550.49 sec

[bwa_aln_core] write to the disk... 0.11 sec

[bwa_aln_core] 47448064 sequences have been processed.

[bwa_aln_core] calculate SA coordinate... 489.87 sec

[bwa_aln_core] write to the disk... 0.11 sec

[bwa_aln_core] 47710208 sequences have been processed.

[bwa_aln_core] calculate SA coordinate... 506.47 sec

[bwa_aln_core] write to the disk... 0.11 sec

[bwa_aln_core] 47972352 sequences have been processed.

[bwa_aln_core] calculate SA coordinate... 468.69 sec

[bwa_aln_core] write to the disk... 0.11 sec

[bwa_aln_core] 48234496 sequences have been processed.

[bwa_aln_core] calculate SA coordinate... 508.88 sec

[bwa_aln_core] write to the disk... 0.10 sec

[bwa_aln_core] 48496640 sequences have been processed.

[bwa_aln_core] calculate SA coordinate... 489.44 sec

[bwa_aln_core] write to the disk... 0.12 sec

[bwa_aln_core] 48758784 sequences have been processed.

[bwa_aln_core] calculate SA coordinate... 448.17 sec

[bwa_aln_core] write to the disk... 0.11 sec

[bwa_aln_core] 49020928 sequences have been processed.

[bwa_aln_core] calculate SA coordinate... 492.70 sec

[bwa_aln_core] write to the disk... 0.12 sec

[bwa_aln_core] 49283072 sequences have been processed.

[bwa_aln_core] calculate SA coordinate... 461.91 sec

[bwa_aln_core] write to the disk... 0.11 sec

[bwa_aln_core] 49545216 sequences have been processed.

[bwa_aln_core] calculate SA coordinate... 507.36 sec

[bwa_aln_core] write to the disk... 0.11 sec

[bwa_aln_core] 49807360 sequences have been processed.

[bwa_aln_core] calculate SA coordinate... 475.31 sec

[bwa_aln_core] write to the disk... 0.12 sec

[bwa_aln_core] 50069504 sequences have been processed.

[bwa_aln_core] calculate SA coordinate... 520.51 sec

[bwa_aln_core] write to the disk... 0.12 sec

[bwa_aln_core] 50331648 sequences have been processed.

[bwa_aln_core] calculate SA coordinate... 447.60 sec

[bwa_aln_core] write to the disk... 0.11 sec

[bwa_aln_core] 50593792 sequences have been processed.

[bwa_aln_core] calculate SA coordinate... 522.50 sec

[bwa_aln_core] write to the disk... 0.14 sec

[bwa_aln_core] 50855936 sequences have been processed.

[bwa_aln_core] calculate SA coordinate... 460.77 sec

[bwa_aln_core] write to the disk... 0.12 sec

[bwa_aln_core] 51118080 sequences have been processed.

[bwa_aln_core] calculate SA coordinate... 489.52 sec

[bwa_aln_core] write to the disk... 0.12 sec

[bwa_aln_core] 51380224 sequences have been processed.

[bwa_aln_core] calculate SA coordinate... 484.60 sec

[bwa_aln_core] write to the disk... 0.11 sec

[bwa_aln_core] 51642368 sequences have been processed.

[bwa_aln_core] calculate SA coordinate... 459.07 sec

[bwa_aln_core] write to the disk... 0.10 sec

[bwa_aln_core] 51904512 sequences have been processed.

[bwa_aln_core] calculate SA coordinate... 524.13 sec

[bwa_aln_core] write to the disk... 0.11 sec

[bwa_aln_core] 52166656 sequences have been processed.

[bwa_aln_core] calculate SA coordinate... 466.82 sec

[bwa_aln_core] write to the disk... 0.12 sec

[bwa_aln_core] 52428800 sequences have been processed.

[bwa_aln_core] calculate SA coordinate... 484.68 sec

[bwa_aln_core] write to the disk... 0.11 sec

[bwa_aln_core] 52690944 sequences have been processed.

[bwa_aln_core] calculate SA coordinate... 510.30 sec

[bwa_aln_core] write to the disk... 0.12 sec

[bwa_aln_core] 52953088 sequences have been processed.

[bwa_aln_core] calculate SA coordinate... 503.38 sec

[bwa_aln_core] write to the disk... 0.11 sec

[bwa_aln_core] 53215232 sequences have been processed.

[bwa_aln_core] calculate SA coordinate... 510.18 sec

[bwa_aln_core] write to the disk... 0.10 sec

[bwa_aln_core] 53477376 sequences have been processed.

[bwa_aln_core] calculate SA coordinate... 441.71 sec

[bwa_aln_core] write to the disk... 0.11 sec

[bwa_aln_core] 53739520 sequences have been processed.

[bwa_aln_core] calculate SA coordinate... 459.78 sec

[bwa_aln_core] write to the disk... 0.10 sec

[bwa_aln_core] 54001664 sequences have been processed.

[bwa_aln_core] calculate SA coordinate... 465.94 sec

[bwa_aln_core] write to the disk... 0.12 sec

[bwa_aln_core] 54263808 sequences have been processed.

[bwa_aln_core] calculate SA coordinate... 451.03 sec

[bwa_aln_core] write to the disk... 0.10 sec

[bwa_aln_core] 54525952 sequences have been processed.

[bwa_aln_core] calculate SA coordinate... 513.88 sec

[bwa_aln_core] write to the disk... 0.11 sec

[bwa_aln_core] 54788096 sequences have been processed.

[bwa_aln_core] calculate SA coordinate... 516.54 sec

[bwa_aln_core] write to the disk... 0.11 sec

[bwa_aln_core] 55050240 sequences have been processed.

[bwa_aln_core] calculate SA coordinate... 474.27 sec

[bwa_aln_core] write to the disk... 0.10 sec

[bwa_aln_core] 55312384 sequences have been processed.

[bwa_aln_core] calculate SA coordinate... 500.26 sec

[bwa_aln_core] write to the disk... 0.11 sec

[bwa_aln_core] 55574528 sequences have been processed.

[bwa_aln_core] calculate SA coordinate... 545.37 sec

[bwa_aln_core] write to the disk... 0.11 sec

[bwa_aln_core] 55836672 sequences have been processed.

[bwa_aln_core] calculate SA coordinate... 511.00 sec

[bwa_aln_core] write to the disk... 0.11 sec

[bwa_aln_core] 56098816 sequences have been processed.

[bwa_aln_core] calculate SA coordinate... 507.34 sec

[bwa_aln_core] write to the disk... 0.11 sec

[bwa_aln_core] 56360960 sequences have been processed.

[bwa_aln_core] calculate SA coordinate... 467.11 sec

[bwa_aln_core] write to the disk... 0.10 sec

[bwa_aln_core] 56623104 sequences have been processed.

[bwa_aln_core] calculate SA coordinate... 493.88 sec

[bwa_aln_core] write to the disk... 0.10 sec

[bwa_aln_core] 56885248 sequences have been processed.

[bwa_aln_core] calculate SA coordinate... 468.89 sec

[bwa_aln_core] write to the disk... 0.11 sec

[bwa_aln_core] 57147392 sequences have been processed.

[bwa_aln_core] calculate SA coordinate... 481.58 sec

[bwa_aln_core] write to the disk... 0.12 sec

[bwa_aln_core] 57409536 sequences have been processed.

[bwa_aln_core] calculate SA coordinate... 510.88 sec

[bwa_aln_core] write to the disk... 0.11 sec

[bwa_aln_core] 57671680 sequences have been processed.

[bwa_aln_core] calculate SA coordinate... 483.83 sec

[bwa_aln_core] write to the disk... 0.11 sec

[bwa_aln_core] 57933824 sequences have been processed.

[bwa_aln_core] calculate SA coordinate... 491.32 sec

[bwa_aln_core] write to the disk... 0.11 sec

[bwa_aln_core] 58195968 sequences have been processed.

[bwa_aln_core] calculate SA coordinate... 482.99 sec

[bwa_aln_core] write to the disk... 0.11 sec

[bwa_aln_core] 58458112 sequences have been processed.

[bwa_aln_core] calculate SA coordinate... 478.14 sec

[bwa_aln_core] write to the disk... 0.11 sec

[bwa_aln_core] 58720256 sequences have been processed.

[bwa_aln_core] calculate SA coordinate... 476.57 sec

[bwa_aln_core] write to the disk... 0.11 sec

[bwa_aln_core] 58982400 sequences have been processed.

[bwa_aln_core] calculate SA coordinate... 553.54 sec

[bwa_aln_core] write to the disk... 0.11 sec

[bwa_aln_core] 59244544 sequences have been processed.

[bwa_aln_core] calculate SA coordinate... 524.81 sec

[bwa_aln_core] write to the disk... 0.10 sec

[bwa_aln_core] 59506688 sequences have been processed.

[bwa_aln_core] calculate SA coordinate... 485.03 sec

[bwa_aln_core] write to the disk... 0.11 sec

[bwa_aln_core] 59768832 sequences have been processed.

[bwa_aln_core] calculate SA coordinate... 539.16 sec

[bwa_aln_core] write to the disk... 0.11 sec

[bwa_aln_core] 60030976 sequences have been processed.

[bwa_aln_core] calculate SA coordinate... 452.23 sec

[bwa_aln_core] write to the disk... 0.11 sec

[bwa_aln_core] 60293120 sequences have been processed.

[bwa_aln_core] calculate SA coordinate... 481.45 sec

[bwa_aln_core] write to the disk... 0.12 sec

[bwa_aln_core] 60555264 sequences have been processed.

[bwa_aln_core] calculate SA coordinate... 474.34 sec

[bwa_aln_core] write to the disk... 0.10 sec

[bwa_aln_core] 60817408 sequences have been processed.

[bwa_aln_core] calculate SA coordinate... 539.81 sec

[bwa_aln_core] write to the disk... 0.11 sec

[bwa_aln_core] 61079552 sequences have been processed.

[bwa_aln_core] calculate SA coordinate... 474.31 sec

[bwa_aln_core] write to the disk... 0.11 sec

[bwa_aln_core] 61341696 sequences have been processed.

[bwa_aln_core] calculate SA coordinate... 495.46 sec

[bwa_aln_core] write to the disk... 0.12 sec

[bwa_aln_core] 61603840 sequences have been processed.

[bwa_aln_core] calculate SA coordinate... 465.97 sec

[bwa_aln_core] write to the disk... 0.11 sec

[bwa_aln_core] 61865984 sequences have been processed.

[bwa_aln_core] calculate SA coordinate... 539.65 sec

[bwa_aln_core] write to the disk... 0.11 sec

[bwa_aln_core] 62128128 sequences have been processed.

[bwa_aln_core] calculate SA coordinate... 483.98 sec

[bwa_aln_core] write to the disk... 0.11 sec

[bwa_aln_core] 62390272 sequences have been processed.

[bwa_aln_core] calculate SA coordinate... 432.40 sec

[bwa_aln_core] write to the disk... 0.11 sec

[bwa_aln_core] 62652416 sequences have been processed.

[bwa_aln_core] calculate SA coordinate... 490.36 sec

[bwa_aln_core] write to the disk... 0.10 sec

[bwa_aln_core] 62914560 sequences have been processed.

[bwa_aln_core] calculate SA coordinate... 491.37 sec

[bwa_aln_core] write to the disk... 0.11 sec

[bwa_aln_core] 63176704 sequences have been processed.

[bwa_aln_core] calculate SA coordinate... 506.06 sec

[bwa_aln_core] write to the disk... 0.10 sec

[bwa_aln_core] 63438848 sequences have been processed.

[bwa_aln_core] calculate SA coordinate... 452.15 sec

[bwa_aln_core] write to the disk... 0.12 sec

[bwa_aln_core] 63700992 sequences have been processed.

[bwa_aln_core] calculate SA coordinate... 479.95 sec

[bwa_aln_core] write to the disk... 0.11 sec

[bwa_aln_core] 63963136 sequences have been processed.

[bwa_aln_core] calculate SA coordinate... 389.26 sec

[bwa_aln_core] write to the disk... 0.11 sec

[bwa_aln_core] 64225280 sequences have been processed.

[bwa_aln_core] calculate SA coordinate... 521.38 sec

[bwa_aln_core] write to the disk... 0.11 sec

[bwa_aln_core] 64487424 sequences have been processed.

[bwa_aln_core] calculate SA coordinate... 443.41 sec

[bwa_aln_core] write to the disk... 0.12 sec

[bwa_aln_core] 64749568 sequences have been processed.

[bwa_aln_core] calculate SA coordinate... 493.17 sec

[bwa_aln_core] write to the disk... 0.11 sec

[bwa_aln_core] 65011712 sequences have been processed.

[bwa_aln_core] calculate SA coordinate... 533.98 sec

[bwa_aln_core] write to the disk... 0.12 sec

[bwa_aln_core] 65273856 sequences have been processed.

[bwa_aln_core] calculate SA coordinate... 456.33 sec

[bwa_aln_core] write to the disk... 0.11 sec

[bwa_aln_core] 65536000 sequences have been processed.

[bwa_aln_core] calculate SA coordinate... 434.56 sec

[bwa_aln_core] write to the disk... 0.12 sec

[bwa_aln_core] 65798144 sequences have been processed.

[bwa_aln_core] calculate SA coordinate... 462.94 sec

[bwa_aln_core] write to the disk... 0.12 sec

[bwa_aln_core] 66060288 sequences have been processed.

[bwa_aln_core] calculate SA coordinate... 435.48 sec

[bwa_aln_core] write to the disk... 0.13 sec

[bwa_aln_core] 66322432 sequences have been processed.

[bwa_aln_core] calculate SA coordinate... 479.74 sec

[bwa_aln_core] write to the disk... 0.12 sec

[bwa_aln_core] 66584576 sequences have been processed.

[bwa_aln_core] calculate SA coordinate... 513.81 sec

[bwa_aln_core] write to the disk... 0.10 sec

[bwa_aln_core] 66846720 sequences have been processed.

[bwa_aln_core] calculate SA coordinate... 517.19 sec

[bwa_aln_core] write to the disk... 0.11 sec

[bwa_aln_core] 67108864 sequences have been processed.

[bwa_aln_core] calculate SA coordinate... 495.65 sec

[bwa_aln_core] write to the disk... 0.11 sec

[bwa_aln_core] 67371008 sequences have been processed.

[bwa_aln_core] calculate SA coordinate... 547.00 sec

[bwa_aln_core] write to the disk... 0.10 sec

[bwa_aln_core] 67633152 sequences have been processed.

[bwa_aln_core] calculate SA coordinate... 463.92 sec

[bwa_aln_core] write to the disk... 0.10 sec

[bwa_aln_core] 67895296 sequences have been processed.

[bwa_aln_core] calculate SA coordinate... 451.05 sec

[bwa_aln_core] write to the disk... 0.09 sec

[bwa_aln_core] 68157440 sequences have been processed.

[bwa_aln_core] calculate SA coordinate... 493.86 sec

[bwa_aln_core] write to the disk... 0.10 sec

[bwa_aln_core] 68419584 sequences have been processed.

[bwa_aln_core] calculate SA coordinate... 470.20 sec

[bwa_aln_core] write to the disk... 0.12 sec

[bwa_aln_core] 68681728 sequences have been processed.

[bwa_aln_core] calculate SA coordinate... 446.05 sec

[bwa_aln_core] write to the disk... 0.11 sec

[bwa_aln_core] 68943872 sequences have been processed.

[bwa_aln_core] calculate SA coordinate... 491.55 sec

[bwa_aln_core] write to the disk... 0.10 sec

[bwa_aln_core] 69206016 sequences have been processed.

[bwa_aln_core] calculate SA coordinate... 498.37 sec

[bwa_aln_core] write to the disk... 0.11 sec

[bwa_aln_core] 69468160 sequences have been processed.

[bwa_aln_core] calculate SA coordinate... 525.45 sec

[bwa_aln_core] write to the disk... 0.11 sec

[bwa_aln_core] 69730304 sequences have been processed.

[bwa_aln_core] calculate SA coordinate... 455.89 sec

[bwa_aln_core] write to the disk... 0.10 sec

[bwa_aln_core] 69992448 sequences have been processed.

[bwa_aln_core] calculate SA coordinate... 468.83 sec

[bwa_aln_core] write to the disk... 0.12 sec

[bwa_aln_core] 70254592 sequences have been processed.

[bwa_aln_core] calculate SA coordinate... 465.04 sec

[bwa_aln_core] write to the disk... 0.12 sec

[bwa_aln_core] 70516736 sequences have been processed.

[bwa_aln_core] calculate SA coordinate... 468.01 sec

[bwa_aln_core] write to the disk... 0.11 sec

[bwa_aln_core] 70778880 sequences have been processed.

[bwa_aln_core] calculate SA coordinate... 499.47 sec

[bwa_aln_core] write to the disk... 0.11 sec

[bwa_aln_core] 71041024 sequences have been processed.

[bwa_aln_core] calculate SA coordinate... 423.17 sec

[bwa_aln_core] write to the disk... 0.11 sec

[bwa_aln_core] 71303168 sequences have been processed.

[bwa_aln_core] calculate SA coordinate... 513.42 sec

[bwa_aln_core] write to the disk... 0.11 sec

[bwa_aln_core] 71565312 sequences have been processed.

[bwa_aln_core] calculate SA coordinate... 453.40 sec

[bwa_aln_core] write to the disk... 0.09 sec

[bwa_aln_core] 71827456 sequences have been processed.

[bwa_aln_core] calculate SA coordinate... 474.78 sec

[bwa_aln_core] write to the disk... 0.10 sec

[bwa_aln_core] 72089600 sequences have been processed.

[bwa_aln_core] calculate SA coordinate... 465.18 sec

[bwa_aln_core] write to the disk... 0.11 sec

[bwa_aln_core] 72351744 sequences have been processed.

[bwa_aln_core] calculate SA coordinate... 431.42 sec

[bwa_aln_core] write to the disk... 0.10 sec

[bwa_aln_core] 72613888 sequences have been processed.

[bwa_aln_core] calculate SA coordinate... 460.68 sec

[bwa_aln_core] write to the disk... 0.10 sec

[bwa_aln_core] 72876032 sequences have been processed.

[bwa_aln_core] calculate SA coordinate... 437.69 sec

[bwa_aln_core] write to the disk... 0.10 sec

[bwa_aln_core] 73138176 sequences have been processed.

[bwa_aln_core] calculate SA coordinate... 482.63 sec

[bwa_aln_core] write to the disk... 0.12 sec

[bwa_aln_core] 73400320 sequences have been processed.

[bwa_aln_core] calculate SA coordinate... 435.16 sec

[bwa_aln_core] write to the disk... 0.12 sec

[bwa_aln_core] 73662464 sequences have been processed.

[bwa_aln_core] calculate SA coordinate... 517.57 sec

[bwa_aln_core] write to the disk... 0.12 sec

[bwa_aln_core] 73924608 sequences have been processed.

[bwa_aln_core] calculate SA coordinate... 510.17 sec

[bwa_aln_core] write to the disk... 0.12 sec

[bwa_aln_core] 74186752 sequences have been processed.

[bwa_aln_core] calculate SA coordinate... 477.75 sec

[bwa_aln_core] write to the disk... 0.12 sec

[bwa_aln_core] 74448896 sequences have been processed.

[bwa_aln_core] calculate SA coordinate... 509.72 sec

[bwa_aln_core] write to the disk... 0.12 sec

[bwa_aln_core] 74711040 sequences have been processed.

[bwa_aln_core] calculate SA coordinate... 570.77 sec

[bwa_aln_core] write to the disk... 0.11 sec

[bwa_aln_core] 74973184 sequences have been processed.

[bwa_aln_core] calculate SA coordinate... 493.22 sec

[bwa_aln_core] write to the disk... 0.13 sec

[bwa_aln_core] 75235328 sequences have been processed.

[bwa_aln_core] calculate SA coordinate... 486.26 sec

[bwa_aln_core] write to the disk... 0.11 sec

[bwa_aln_core] 75497472 sequences have been processed.

[bwa_aln_core] calculate SA coordinate... 516.41 sec

[bwa_aln_core] write to the disk... 0.11 sec

[bwa_aln_core] 75759616 sequences have been processed.

[bwa_aln_core] calculate SA coordinate... 523.04 sec

[bwa_aln_core] write to the disk... 0.10 sec

[bwa_aln_core] 76021760 sequences have been processed.

[bwa_aln_core] calculate SA coordinate... 465.59 sec

[bwa_aln_core] write to the disk... 0.11 sec

[bwa_aln_core] 76283904 sequences have been processed.

[bwa_aln_core] calculate SA coordinate... 494.55 sec

[bwa_aln_core] write to the disk... 0.10 sec

[bwa_aln_core] 76546048 sequences have been processed.

[bwa_aln_core] calculate SA coordinate... 499.71 sec

[bwa_aln_core] write to the disk... 0.11 sec

[bwa_aln_core] 76808192 sequences have been processed.

[bwa_aln_core] calculate SA coordinate... 502.33 sec

[bwa_aln_core] write to the disk... 0.10 sec

[bwa_aln_core] 77070336 sequences have been processed.

[bwa_aln_core] calculate SA coordinate... 466.85 sec

[bwa_aln_core] write to the disk... 0.11 sec

[bwa_aln_core] 77332480 sequences have been processed.

[bwa_aln_core] calculate SA coordinate... 451.71 sec

[bwa_aln_core] write to the disk... 0.10 sec

[bwa_aln_core] 77594624 sequences have been processed.

[bwa_aln_core] calculate SA coordinate... 531.64 sec

[bwa_aln_core] write to the disk... 0.11 sec

[bwa_aln_core] 77856768 sequences have been processed.

[bwa_aln_core] calculate SA coordinate... 490.41 sec

[bwa_aln_core] write to the disk... 0.09 sec

[bwa_aln_core] 78118912 sequences have been processed.

[bwa_aln_core] calculate SA coordinate... 476.18 sec

[bwa_aln_core] write to the disk... 0.10 sec

[bwa_aln_core] 78381056 sequences have been processed.

[bwa_aln_core] calculate SA coordinate... 471.66 sec

[bwa_aln_core] write to the disk... 0.11 sec

[bwa_aln_core] 78643200 sequences have been processed.

[bwa_aln_core] calculate SA coordinate... 532.95 sec

[bwa_aln_core] write to the disk... 0.10 sec

[bwa_aln_core] 78905344 sequences have been processed.

[bwa_aln_core] calculate SA coordinate... 507.20 sec

[bwa_aln_core] write to the disk... 0.10 sec

[bwa_aln_core] 79167488 sequences have been processed.

[bwa_aln_core] calculate SA coordinate... 541.43 sec

[bwa_aln_core] write to the disk... 0.11 sec

[bwa_aln_core] 79429632 sequences have been processed.

[bwa_aln_core] calculate SA coordinate... 509.03 sec

[bwa_aln_core] write to the disk... 0.12 sec

[bwa_aln_core] 79691776 sequences have been processed.

[bwa_aln_core] calculate SA coordinate... 524.89 sec

[bwa_aln_core] write to the disk... 0.12 sec

[bwa_aln_core] 79953920 sequences have been processed.

[bwa_aln_core] calculate SA coordinate... 449.52 sec

[bwa_aln_core] write to the disk... 0.13 sec

[bwa_aln_core] 80216064 sequences have been processed.

[bwa_aln_core] calculate SA coordinate... 529.15 sec

[bwa_aln_core] write to the disk... 0.11 sec

[bwa_aln_core] 80478208 sequences have been processed.

[bwa_aln_core] calculate SA coordinate... 457.80 sec

[bwa_aln_core] write to the disk... 0.12 sec

[bwa_aln_core] 80740352 sequences have been processed.

[bwa_aln_core] calculate SA coordinate... 476.06 sec

[bwa_aln_core] write to the disk... 0.12 sec

[bwa_aln_core] 81002496 sequences have been processed.

[bwa_aln_core] calculate SA coordinate... 501.64 sec

[bwa_aln_core] write to the disk... 0.11 sec

[bwa_aln_core] 81264640 sequences have been processed.

[bwa_aln_core] calculate SA coordinate... 471.00 sec

[bwa_aln_core] write to the disk... 0.11 sec

[bwa_aln_core] 81526784 sequences have been processed.

[bwa_aln_core] calculate SA coordinate... 519.12 sec

[bwa_aln_core] write to the disk... 0.11 sec

[bwa_aln_core] 81788928 sequences have been processed.

[bwa_aln_core] calculate SA coordinate... 549.58 sec

[bwa_aln_core] write to the disk... 0.11 sec

[bwa_aln_core] 82051072 sequences have been processed.

[bwa_aln_core] calculate SA coordinate... 501.16 sec

[bwa_aln_core] write to the disk... 0.11 sec

[bwa_aln_core] 82313216 sequences have been processed.

[bwa_aln_core] calculate SA coordinate... 479.36 sec

[bwa_aln_core] write to the disk... 0.11 sec

[bwa_aln_core] 82575360 sequences have been processed.

[bwa_aln_core] calculate SA coordinate... 459.82 sec

[bwa_aln_core] write to the disk... 0.10 sec

[bwa_aln_core] 82837504 sequences have been processed.

[bwa_aln_core] calculate SA coordinate... 517.70 sec

[bwa_aln_core] write to the disk... 0.11 sec

[bwa_aln_core] 83099648 sequences have been processed.

[bwa_aln_core] calculate SA coordinate... 497.27 sec

[bwa_aln_core] write to the disk... 0.11 sec

[bwa_aln_core] 83361792 sequences have been processed.

[bwa_aln_core] calculate SA coordinate... 509.20 sec

[bwa_aln_core] write to the disk... 0.10 sec

[bwa_aln_core] 83623936 sequences have been processed.

[bwa_aln_core] calculate SA coordinate... 438.68 sec

[bwa_aln_core] write to the disk... 0.11 sec

[bwa_aln_core] 83886080 sequences have been processed.

[bwa_aln_core] calculate SA coordinate... 468.90 sec

[bwa_aln_core] write to the disk... 0.11 sec

[bwa_aln_core] 84148224 sequences have been processed.

[bwa_aln_core] calculate SA coordinate... 477.68 sec

[bwa_aln_core] write to the disk... 0.12 sec

[bwa_aln_core] 84410368 sequences have been processed.

[bwa_aln_core] calculate SA coordinate... 530.46 sec

[bwa_aln_core] write to the disk... 0.11 sec

[bwa_aln_core] 84672512 sequences have been processed.

[bwa_aln_core] calculate SA coordinate... 472.29 sec

[bwa_aln_core] write to the disk... 0.11 sec

[bwa_aln_core] 84934656 sequences have been processed.

[bwa_aln_core] calculate SA coordinate... 461.46 sec

[bwa_aln_core] write to the disk... 0.10 sec

[bwa_aln_core] 85196800 sequences have been processed.

[bwa_aln_core] calculate SA coordinate... 508.08 sec

[bwa_aln_core] write to the disk... 0.11 sec

[bwa_aln_core] 85458944 sequences have been processed.

[bwa_aln_core] calculate SA coordinate... 458.76 sec

[bwa_aln_core] write to the disk... 0.11 sec

[bwa_aln_core] 85721088 sequences have been processed.

[bwa_aln_core] calculate SA coordinate... 504.19 sec

[bwa_aln_core] write to the disk... 0.10 sec

[bwa_aln_core] 85983232 sequences have been processed.

[bwa_aln_core] calculate SA coordinate... 420.25 sec

[bwa_aln_core] write to the disk... 0.10 sec

[bwa_aln_core] 86245376 sequences have been processed.

[bwa_aln_core] calculate SA coordinate... 505.73 sec

[bwa_aln_core] write to the disk... 0.11 sec

[bwa_aln_core] 86507520 sequences have been processed.

[bwa_aln_core] calculate SA coordinate... 546.51 sec

[bwa_aln_core] write to the disk... 0.09 sec

[bwa_aln_core] 86769664 sequences have been processed.

[bwa_aln_core] calculate SA coordinate... 489.13 sec

[bwa_aln_core] write to the disk... 0.09 sec

[bwa_aln_core] 87031808 sequences have been processed.

[bwa_aln_core] calculate SA coordinate... 518.31 sec

[bwa_aln_core] write to the disk... 0.10 sec

[bwa_aln_core] 87293952 sequences have been processed.

[bwa_aln_core] calculate SA coordinate... 536.88 sec

[bwa_aln_core] write to the disk... 0.09 sec

[bwa_aln_core] 87556096 sequences have been processed.

[bwa_aln_core] calculate SA coordinate... 518.67 sec

[bwa_aln_core] write to the disk... 0.09 sec

[bwa_aln_core] 87818240 sequences have been processed.

[bwa_aln_core] calculate SA coordinate... 521.86 sec

[bwa_aln_core] write to the disk... 0.09 sec

[bwa_aln_core] 88080384 sequences have been processed.

[bwa_aln_core] calculate SA coordinate... 493.77 sec

[bwa_aln_core] write to the disk... 0.09 sec

[bwa_aln_core] 88342528 sequences have been processed.

[bwa_aln_core] calculate SA coordinate... 512.43 sec

[bwa_aln_core] write to the disk... 0.08 sec

[bwa_aln_core] 88604672 sequences have been processed.

[bwa_aln_core] calculate SA coordinate... 501.20 sec

[bwa_aln_core] write to the disk... 0.08 sec

[bwa_aln_core] 88866816 sequences have been processed.

[bwa_aln_core] calculate SA coordinate... 471.02 sec

[bwa_aln_core] write to the disk... 0.08 sec

[bwa_aln_core] 89128960 sequences have been processed.

[bwa_aln_core] calculate SA coordinate... 485.90 sec

[bwa_aln_core] write to the disk... 0.12 sec

[bwa_aln_core] 89391104 sequences have been processed.

[bwa_aln_core] calculate SA coordinate... 470.01 sec

[bwa_aln_core] write to the disk... 0.11 sec

[bwa_aln_core] 89653248 sequences have been processed.

[bwa_aln_core] calculate SA coordinate... 533.50 sec

[bwa_aln_core] write to the disk... 0.11 sec

[bwa_aln_core] 89915392 sequences have been processed.

[bwa_aln_core] calculate SA coordinate... 507.38 sec

[bwa_aln_core] write to the disk... 0.12 sec

[bwa_aln_core] 90177536 sequences have been processed.

[bwa_aln_core] calculate SA coordinate... 498.24 sec

[bwa_aln_core] write to the disk... 0.11 sec

[bwa_aln_core] 90439680 sequences have been processed.

[bwa_aln_core] calculate SA coordinate... 485.26 sec

[bwa_aln_core] write to the disk... 0.12 sec

[bwa_aln_core] 90701824 sequences have been processed.

[bwa_aln_core] calculate SA coordinate... 508.25 sec

[bwa_aln_core] write to the disk... 0.11 sec

[bwa_aln_core] 90963968 sequences have been processed.

[bwa_aln_core] calculate SA coordinate... 509.11 sec

[bwa_aln_core] write to the disk... 0.12 sec

[bwa_aln_core] 91226112 sequences have been processed.

[bwa_aln_core] calculate SA coordinate... 507.83 sec

[bwa_aln_core] write to the disk... 0.12 sec

[bwa_aln_core] 91488256 sequences have been processed.

[bwa_aln_core] calculate SA coordinate... 486.66 sec

[bwa_aln_core] write to the disk... 0.12 sec

[bwa_aln_core] 91750400 sequences have been processed.

[bwa_aln_core] calculate SA coordinate... 507.54 sec

[bwa_aln_core] write to the disk... 0.11 sec

[bwa_aln_core] 92012544 sequences have been processed.

[bwa_aln_core] calculate SA coordinate... 460.81 sec

[bwa_aln_core] write to the disk... 0.12 sec

[bwa_aln_core] 92274688 sequences have been processed.

[bwa_aln_core] calculate SA coordinate... 465.00 sec

[bwa_aln_core] write to the disk... 0.11 sec

[bwa_aln_core] 92536832 sequences have been processed.

[bwa_aln_core] calculate SA coordinate... 446.78 sec

[bwa_aln_core] write to the disk... 0.11 sec

[bwa_aln_core] 92798976 sequences have been processed.

[bwa_aln_core] calculate SA coordinate... 432.53 sec

[bwa_aln_core] write to the disk... 0.11 sec

[bwa_aln_core] 93061120 sequences have been processed.

[bwa_aln_core] calculate SA coordinate... 474.97 sec

[bwa_aln_core] write to the disk... 0.11 sec

[bwa_aln_core] 93323264 sequences have been processed.

[bwa_aln_core] calculate SA coordinate... 550.93 sec

[bwa_aln_core] write to the disk... 0.12 sec

[bwa_aln_core] 93585408 sequences have been processed.

[bwa_aln_core] calculate SA coordinate... 525.20 sec

[bwa_aln_core] write to the disk... 0.12 sec

[bwa_aln_core] 93847552 sequences have been processed.

[bwa_aln_core] calculate SA coordinate... 485.99 sec

[bwa_aln_core] write to the disk... 0.12 sec

[bwa_aln_core] 94109696 sequences have been processed.

[bwa_aln_core] calculate SA coordinate... 529.58 sec

[bwa_aln_core] write to the disk... 0.11 sec

[bwa_aln_core] 94371840 sequences have been processed.

[bwa_aln_core] calculate SA coordinate... 554.19 sec

[bwa_aln_core] write to the disk... 0.12 sec

[bwa_aln_core] 94633984 sequences have been processed.

[bwa_aln_core] calculate SA coordinate... 518.54 sec

[bwa_aln_core] write to the disk... 0.11 sec

[bwa_aln_core] 94896128 sequences have been processed.

[bwa_aln_core] calculate SA coordinate... 495.21 sec

[bwa_aln_core] write to the disk... 0.11 sec

[bwa_aln_core] 95158272 sequences have been processed.

[bwa_aln_core] calculate SA coordinate... 495.18 sec

[bwa_aln_core] write to the disk... 0.11 sec

[bwa_aln_core] 95420416 sequences have been processed.

[bwa_aln_core] calculate SA coordinate... 443.88 sec

[bwa_aln_core] write to the disk... 0.11 sec

[bwa_aln_core] 95682560 sequences have been processed.

[bwa_aln_core] calculate SA coordinate... 531.42 sec

[bwa_aln_core] write to the disk... 0.12 sec

[bwa_aln_core] 95944704 sequences have been processed.

[bwa_aln_core] calculate SA coordinate... 505.74 sec

[bwa_aln_core] write to the disk... 0.11 sec

[bwa_aln_core] 96206848 sequences have been processed.

[bwa_aln_core] calculate SA coordinate... 516.96 sec

[bwa_aln_core] write to the disk... 0.11 sec

[bwa_aln_core] 96468992 sequences have been processed.

[bwa_aln_core] calculate SA coordinate... 465.36 sec

[bwa_aln_core] write to the disk... 0.11 sec

[bwa_aln_core] 96731136 sequences have been processed.

[bwa_aln_core] calculate SA coordinate... 556.25 sec

[bwa_aln_core] write to the disk... 0.11 sec

[bwa_aln_core] 96993280 sequences have been processed.

[bwa_aln_core] calculate SA coordinate... 529.77 sec

[bwa_aln_core] write to the disk... 0.11 sec

[bwa_aln_core] 97255424 sequences have been processed.

[bwa_aln_core] calculate SA coordinate... 509.95 sec

[bwa_aln_core] write to the disk... 0.12 sec

[bwa_aln_core] 97517568 sequences have been processed.

[bwa_aln_core] calculate SA coordinate... 464.04 sec

[bwa_aln_core] write to the disk... 0.12 sec

[bwa_aln_core] 97779712 sequences have been processed.

[bwa_aln_core] calculate SA coordinate... 507.71 sec

[bwa_aln_core] write to the disk... 0.11 sec

[bwa_aln_core] 98041856 sequences have been processed.

[bwa_aln_core] calculate SA coordinate... 523.69 sec

[bwa_aln_core] write to the disk... 0.11 sec

[bwa_aln_core] 98304000 sequences have been processed.

[bwa_aln_core] calculate SA coordinate... 512.12 sec

[bwa_aln_core] write to the disk... 0.10 sec

[bwa_aln_core] 98566144 sequences have been processed.

[bwa_aln_core] calculate SA coordinate... 472.72 sec

[bwa_aln_core] write to the disk... 0.11 sec

[bwa_aln_core] 98828288 sequences have been processed.

[bwa_aln_core] calculate SA coordinate... 457.09 sec

[bwa_aln_core] write to the disk... 0.10 sec

[bwa_aln_core] 99090432 sequences have been processed.

[bwa_aln_core] calculate SA coordinate... 547.36 sec

[bwa_aln_core] write to the disk... 0.11 sec

[bwa_aln_core] 99352576 sequences have been processed.

[bwa_aln_core] calculate SA coordinate... 450.74 sec

[bwa_aln_core] write to the disk... 0.10 sec

[bwa_aln_core] 99614720 sequences have been processed.

[bwa_aln_core] calculate SA coordinate... 510.62 sec

[bwa_aln_core] write to the disk... 0.11 sec

[bwa_aln_core] 99876864 sequences have been processed.

[bwa_aln_core] calculate SA coordinate... 521.10 sec

[bwa_aln_core] write to the disk... 0.10 sec

[bwa_aln_core] 100139008 sequences have been processed.

[bwa_aln_core] calculate SA coordinate... 513.17 sec

[bwa_aln_core] write to the disk... 0.11 sec

[bwa_aln_core] 100401152 sequences have been processed.

[bwa_aln_core] calculate SA coordinate... 447.78 sec

[bwa_aln_core] write to the disk... 0.10 sec

[bwa_aln_core] 100663296 sequences have been processed.

[bwa_aln_core] calculate SA coordinate... 484.94 sec

[bwa_aln_core] write to the disk... 0.10 sec

[bwa_aln_core] 100925440 sequences have been processed.

[bwa_aln_core] calculate SA coordinate... 488.98 sec

[bwa_aln_core] write to the disk... 0.10 sec

[bwa_aln_core] 101187584 sequences have been processed.

[bwa_aln_core] calculate SA coordinate... 544.95 sec

[bwa_aln_core] write to the disk... 0.10 sec

[bwa_aln_core] 101449728 sequences have been processed.

[bwa_aln_core] calculate SA coordinate... 497.45 sec

[bwa_aln_core] write to the disk... 0.12 sec

[bwa_aln_core] 101711872 sequences have been processed.

[bwa_aln_core] calculate SA coordinate... 527.83 sec

[bwa_aln_core] write to the disk... 0.11 sec

[bwa_aln_core] 101974016 sequences have been processed.

[bwa_aln_core] calculate SA coordinate... 461.76 sec

[bwa_aln_core] write to the disk... 0.11 sec

[bwa_aln_core] 102236160 sequences have been processed.

[bwa_aln_core] calculate SA coordinate... 504.09 sec

[bwa_aln_core] write to the disk... 0.11 sec

[bwa_aln_core] 102498304 sequences have been processed.

[bwa_aln_core] calculate SA coordinate... 510.79 sec

[bwa_aln_core] write to the disk... 0.12 sec

[bwa_aln_core] 102760448 sequences have been processed.

[bwa_aln_core] calculate SA coordinate... 479.28 sec

[bwa_aln_core] write to the disk... 0.12 sec

[bwa_aln_core] 103022592 sequences have been processed.

[bwa_aln_core] calculate SA coordinate... 490.06 sec

[bwa_aln_core] write to the disk... 0.11 sec

[bwa_aln_core] 103284736 sequences have been processed.

[bwa_aln_core] calculate SA coordinate... 444.28 sec

[bwa_aln_core] write to the disk... 0.11 sec

[bwa_aln_core] 103546880 sequences have been processed.

[bwa_aln_core] calculate SA coordinate... 485.99 sec

[bwa_aln_core] write to the disk... 0.12 sec

[bwa_aln_core] 103809024 sequences have been processed.

[bwa_aln_core] calculate SA coordinate... 496.69 sec

[bwa_aln_core] write to the disk... 0.12 sec

[bwa_aln_core] 104071168 sequences have been processed.

[bwa_aln_core] calculate SA coordinate... 464.28 sec

[bwa_aln_core] write to the disk... 0.12 sec

[bwa_aln_core] 104333312 sequences have been processed.

[bwa_aln_core] calculate SA coordinate... 532.76 sec

[bwa_aln_core] write to the disk... 0.11 sec

[bwa_aln_core] 104595456 sequences have been processed.

[bwa_aln_core] calculate SA coordinate... 537.59 sec

[bwa_aln_core] write to the disk... 0.11 sec

[bwa_aln_core] 104857600 sequences have been processed.

[bwa_aln_core] calculate SA coordinate... 524.46 sec

[bwa_aln_core] write to the disk... 0.11 sec

[bwa_aln_core] 105119744 sequences have been processed.

[bwa_aln_core] calculate SA coordinate... 556.88 sec

[bwa_aln_core] write to the disk... 0.12 sec

[bwa_aln_core] 105381888 sequences have been processed.

[bwa_aln_core] calculate SA coordinate... 554.70 sec

[bwa_aln_core] write to the disk... 0.12 sec

[bwa_aln_core] 105644032 sequences have been processed.

[bwa_aln_core] calculate SA coordinate... 453.49 sec

[bwa_aln_core] write to the disk... 0.12 sec

[bwa_aln_core] 105906176 sequences have been processed.

[bwa_aln_core] calculate SA coordinate... 447.42 sec

[bwa_aln_core] write to the disk... 0.11 sec

[bwa_aln_core] 106168320 sequences have been processed.

[bwa_aln_core] calculate SA coordinate... 496.79 sec

[bwa_aln_core] write to the disk... 0.10 sec

[bwa_aln_core] 106430464 sequences have been processed.

[bwa_aln_core] calculate SA coordinate... 511.24 sec

[bwa_aln_core] write to the disk... 0.11 sec

[bwa_aln_core] 106692608 sequences have been processed.

[bwa_aln_core] calculate SA coordinate... 470.40 sec

[bwa_aln_core] write to the disk... 0.12 sec

[bwa_aln_core] 106954752 sequences have been processed.

[bwa_aln_core] calculate SA coordinate... 481.05 sec

[bwa_aln_core] write to the disk... 0.12 sec

[bwa_aln_core] 107216896 sequences have been processed.

[bwa_aln_core] calculate SA coordinate... 505.62 sec

[bwa_aln_core] write to the disk... 0.12 sec

[bwa_aln_core] 107479040 sequences have been processed.

[bwa_aln_core] calculate SA coordinate... 452.43 sec

[bwa_aln_core] write to the disk... 0.12 sec

[bwa_aln_core] 107741184 sequences have been processed.

[bwa_aln_core] calculate SA coordinate... 444.26 sec

[bwa_aln_core] write to the disk... 0.12 sec

[bwa_aln_core] 108003328 sequences have been processed.

[bwa_aln_core] calculate SA coordinate... 534.40 sec

[bwa_aln_core] write to the disk... 0.12 sec

[bwa_aln_core] 108265472 sequences have been processed.

[bwa_aln_core] calculate SA coordinate... 541.34 sec

[bwa_aln_core] write to the disk... 0.12 sec

[bwa_aln_core] 108527616 sequences have been processed.

[bwa_aln_core] calculate SA coordinate... 516.57 sec

[bwa_aln_core] write to the disk... 0.12 sec

[bwa_aln_core] 108789760 sequences have been processed.

[bwa_aln_core] calculate SA coordinate... 455.75 sec

[bwa_aln_core] write to the disk... 0.12 sec

[bwa_aln_core] 109051904 sequences have been processed.

[bwa_aln_core] calculate SA coordinate... 485.52 sec

[bwa_aln_core] write to the disk... 0.12 sec

[bwa_aln_core] 109314048 sequences have been processed.

[bwa_aln_core] calculate SA coordinate... 469.55 sec

[bwa_aln_core] write to the disk... 0.13 sec

[bwa_aln_core] 109576192 sequences have been processed.

[bwa_aln_core] calculate SA coordinate... 510.76 sec

[bwa_aln_core] write to the disk... 0.11 sec

[bwa_aln_core] 109838336 sequences have been processed.

[bwa_aln_core] calculate SA coordinate... 462.39 sec

[bwa_aln_core] write to the disk... 0.12 sec

[bwa_aln_core] 110100480 sequences have been processed.

[bwa_aln_core] calculate SA coordinate... 453.96 sec

[bwa_aln_core] write to the disk... 0.12 sec

[bwa_aln_core] 110362624 sequences have been processed.

[bwa_aln_core] calculate SA coordinate... 545.20 sec

[bwa_aln_core] write to the disk... 0.11 sec

[bwa_aln_core] 110624768 sequences have been processed.

[bwa_aln_core] calculate SA coordinate... 512.31 sec

[bwa_aln_core] write to the disk... 0.11 sec

[bwa_aln_core] 110886912 sequences have been processed.

[bwa_aln_core] calculate SA coordinate... 452.19 sec

[bwa_aln_core] write to the disk... 0.10 sec

[bwa_aln_core] 111149056 sequences have been processed.

[bwa_aln_core] calculate SA coordinate... 455.23 sec

[bwa_aln_core] write to the disk... 0.12 sec

[bwa_aln_core] 111411200 sequences have been processed.

[bwa_aln_core] calculate SA coordinate... 514.44 sec

[bwa_aln_core] write to the disk... 0.13 sec

[bwa_aln_core] 111673344 sequences have been processed.

[bwa_aln_core] calculate SA coordinate... 452.17 sec

[bwa_aln_core] write to the disk... 0.12 sec

[bwa_aln_core] 111935488 sequences have been processed.

[bwa_aln_core] calculate SA coordinate... 560.10 sec

[bwa_aln_core] write to the disk... 0.10 sec

[bwa_aln_core] 112197632 sequences have been processed.

[bwa_aln_core] calculate SA coordinate... 458.76 sec

[bwa_aln_core] write to the disk... 0.11 sec

[bwa_aln_core] 112459776 sequences have been processed.

[bwa_aln_core] calculate SA coordinate... 540.10 sec

[bwa_aln_core] write to the disk... 0.11 sec

[bwa_aln_core] 112721920 sequences have been processed.

[bwa_aln_core] calculate SA coordinate... 600.92 sec

[bwa_aln_core] write to the disk... 0.13 sec

[bwa_aln_core] 112984064 sequences have been processed.

[bwa_aln_core] calculate SA coordinate... 513.24 sec

[bwa_aln_core] write to the disk... 0.11 sec

[bwa_aln_core] 113246208 sequences have been processed.

[bwa_aln_core] calculate SA coordinate... 509.28 sec

[bwa_aln_core] write to the disk... 0.11 sec

[bwa_aln_core] 113508352 sequences have been processed.

[bwa_aln_core] calculate SA coordinate... 493.66 sec

[bwa_aln_core] write to the disk... 0.11 sec

[bwa_aln_core] 113770496 sequences have been processed.

[bwa_aln_core] calculate SA coordinate... 546.87 sec

[bwa_aln_core] write to the disk... 0.12 sec

[bwa_aln_core] 114032640 sequences have been processed.

[bwa_aln_core] calculate SA coordinate... 470.16 sec

[bwa_aln_core] write to the disk... 0.12 sec

[bwa_aln_core] 114294784 sequences have been processed.

[bwa_aln_core] calculate SA coordinate... 505.13 sec

[bwa_aln_core] write to the disk... 0.12 sec

[bwa_aln_core] 114556928 sequences have been processed.

[bwa_aln_core] calculate SA coordinate... 408.63 sec

[bwa_aln_core] write to the disk... 0.12 sec

[bwa_aln_core] 114819072 sequences have been processed.

[bwa_aln_core] calculate SA coordinate... 475.30 sec

[bwa_aln_core] write to the disk... 0.12 sec

[bwa_aln_core] 115081216 sequences have been processed.

[bwa_aln_core] calculate SA coordinate... 494.00 sec

[bwa_aln_core] write to the disk... 0.11 sec

[bwa_aln_core] 115343360 sequences have been processed.

[bwa_aln_core] calculate SA coordinate... 503.07 sec

[bwa_aln_core] write to the disk... 0.11 sec

[bwa_aln_core] 115605504 sequences have been processed.

[bwa_aln_core] calculate SA coordinate... 505.19 sec

[bwa_aln_core] write to the disk... 0.11 sec

[bwa_aln_core] 115867648 sequences have been processed.

[bwa_aln_core] calculate SA coordinate... 507.64 sec

[bwa_aln_core] write to the disk... 0.13 sec

[bwa_aln_core] 116129792 sequences have been processed.

[bwa_aln_core] calculate SA coordinate... 497.45 sec

[bwa_aln_core] write to the disk... 0.13 sec

[bwa_aln_core] 116391936 sequences have been processed.

[bwa_aln_core] calculate SA coordinate... 503.18 sec

[bwa_aln_core] write to the disk... 0.13 sec

[bwa_aln_core] 116654080 sequences have been processed.

[bwa_aln_core] calculate SA coordinate... 593.55 sec

[bwa_aln_core] write to the disk... 0.12 sec

[bwa_aln_core] 116916224 sequences have been processed.

[bwa_aln_core] calculate SA coordinate... 533.32 sec

[bwa_aln_core] write to the disk... 0.12 sec

[bwa_aln_core] 117178368 sequences have been processed.

[bwa_aln_core] calculate SA coordinate... 526.55 sec

[bwa_aln_core] write to the disk... 0.10 sec

[bwa_aln_core] 117440512 sequences have been processed.

[bwa_aln_core] calculate SA coordinate... 540.92 sec

[bwa_aln_core] write to the disk... 0.11 sec

[bwa_aln_core] 117702656 sequences have been processed.

[bwa_aln_core] calculate SA coordinate... 424.80 sec

[bwa_aln_core] write to the disk... 0.11 sec

[bwa_aln_core] 117964800 sequences have been processed.

[bwa_aln_core] calculate SA coordinate... 516.88 sec

[bwa_aln_core] write to the disk... 0.11 sec

[bwa_aln_core] 118226944 sequences have been processed.

[bwa_aln_core] calculate SA coordinate... 500.10 sec

[bwa_aln_core] write to the disk... 0.11 sec

[bwa_aln_core] 118489088 sequences have been processed.

[bwa_aln_core] calculate SA coordinate... 456.91 sec

[bwa_aln_core] write to the disk... 0.11 sec

[bwa_aln_core] 118751232 sequences have been processed.

[bwa_aln_core] calculate SA coordinate... 476.66 sec

[bwa_aln_core] write to the disk... 0.11 sec

[bwa_aln_core] 119013376 sequences have been processed.

[bwa_aln_core] calculate SA coordinate... 423.47 sec

[bwa_aln_core] write to the disk... 0.12 sec

[bwa_aln_core] 119275520 sequences have been processed.

[bwa_aln_core] calculate SA coordinate... 484.74 sec

[bwa_aln_core] write to the disk... 0.15 sec

[bwa_aln_core] 119537664 sequences have been processed.

[bwa_aln_core] calculate SA coordinate... 451.18 sec

[bwa_aln_core] write to the disk... 0.12 sec

[bwa_aln_core] 119799808 sequences have been processed.

[bwa_aln_core] calculate SA coordinate... 493.15 sec

[bwa_aln_core] write to the disk... 0.12 sec

[bwa_aln_core] 120061952 sequences have been processed.

[bwa_aln_core] calculate SA coordinate... 496.04 sec

[bwa_aln_core] write to the disk... 0.10 sec

[bwa_aln_core] 120324096 sequences have been processed.

[bwa_aln_core] calculate SA coordinate... 507.22 sec

[bwa_aln_core] write to the disk... 0.12 sec

[bwa_aln_core] 120586240 sequences have been processed.

[bwa_aln_core] calculate SA coordinate... 525.09 sec

[bwa_aln_core] write to the disk... 0.11 sec

[bwa_aln_core] 120848384 sequences have been processed.

[bwa_aln_core] calculate SA coordinate... 480.68 sec

[bwa_aln_core] write to the disk... 0.12 sec

[bwa_aln_core] 121110528 sequences have been processed.

[bwa_aln_core] calculate SA coordinate... 467.58 sec

[bwa_aln_core] write to the disk... 0.11 sec

[bwa_aln_core] 121372672 sequences have been processed.

[bwa_aln_core] calculate SA coordinate... 466.46 sec

[bwa_aln_core] write to the disk... 0.12 sec

[bwa_aln_core] 121634816 sequences have been processed.

[bwa_aln_core] calculate SA coordinate... 471.74 sec

[bwa_aln_core] write to the disk... 0.11 sec

[bwa_aln_core] 121896960 sequences have been processed.

[bwa_aln_core] calculate SA coordinate... 522.54 sec

[bwa_aln_core] write to the disk... 0.12 sec

[bwa_aln_core] 122159104 sequences have been processed.

[bwa_aln_core] calculate SA coordinate... 470.79 sec

[bwa_aln_core] write to the disk... 0.12 sec

[bwa_aln_core] 122421248 sequences have been processed.

[bwa_aln_core] calculate SA coordinate... 467.07 sec

[bwa_aln_core] write to the disk... 0.11 sec

[bwa_aln_core] 122683392 sequences have been processed.

[bwa_aln_core] calculate SA coordinate... 440.64 sec

[bwa_aln_core] write to the disk... 0.12 sec

[bwa_aln_core] 122945536 sequences have been processed.

[bwa_aln_core] calculate SA coordinate... 496.52 sec

[bwa_aln_core] write to the disk... 0.12 sec

[bwa_aln_core] 123207680 sequences have been processed.

[bwa_aln_core] calculate SA coordinate... 535.48 sec

[bwa_aln_core] write to the disk... 0.11 sec

[bwa_aln_core] 123469824 sequences have been processed.

[bwa_aln_core] calculate SA coordinate... 425.93 sec

[bwa_aln_core] write to the disk... 0.10 sec

[bwa_aln_core] 123731968 sequences have been processed.

[bwa_aln_core] calculate SA coordinate... 476.63 sec

[bwa_aln_core] write to the disk... 0.11 sec

[bwa_aln_core] 123994112 sequences have been processed.

[bwa_aln_core] calculate SA coordinate... 443.43 sec

[bwa_aln_core] write to the disk... 0.11 sec

[bwa_aln_core] 124256256 sequences have been processed.

[bwa_aln_core] calculate SA coordinate... 459.47 sec

[bwa_aln_core] write to the disk... 0.10 sec

[bwa_aln_core] 124518400 sequences have been processed.

[bwa_aln_core] calculate SA coordinate... 561.14 sec

[bwa_aln_core] write to the disk... 0.10 sec

[bwa_aln_core] 124780544 sequences have been processed.

[bwa_aln_core] calculate SA coordinate... 479.49 sec

[bwa_aln_core] write to the disk... 0.11 sec

[bwa_aln_core] 125042688 sequences have been processed.

[bwa_aln_core] calculate SA coordinate... 517.11 sec

[bwa_aln_core] write to the disk... 0.11 sec

[bwa_aln_core] 125304832 sequences have been processed.

[bwa_aln_core] calculate SA coordinate... 499.43 sec

[bwa_aln_core] write to the disk... 0.10 sec

[bwa_aln_core] 125566976 sequences have been processed.

[bwa_aln_core] calculate SA coordinate... 477.00 sec

[bwa_aln_core] write to the disk... 0.11 sec

[bwa_aln_core] 125829120 sequences have been processed.

[bwa_aln_core] calculate SA coordinate... 500.22 sec

[bwa_aln_core] write to the disk... 0.10 sec

[bwa_aln_core] 126091264 sequences have been processed.

[bwa_aln_core] calculate SA coordinate... 480.00 sec

[bwa_aln_core] write to the disk... 0.11 sec

[bwa_aln_core] 126353408 sequences have been processed.

[bwa_aln_core] calculate SA coordinate... 466.85 sec

[bwa_aln_core] write to the disk... 0.10 sec

[bwa_aln_core] 126615552 sequences have been processed.

[bwa_aln_core] calculate SA coordinate... 534.24 sec

[bwa_aln_core] write to the disk... 0.12 sec

[bwa_aln_core] 126877696 sequences have been processed.

[bwa_aln_core] calculate SA coordinate... 512.87 sec

[bwa_aln_core] write to the disk... 0.11 sec

[bwa_aln_core] 127139840 sequences have been processed.

[bwa_aln_core] calculate SA coordinate... 491.37 sec

[bwa_aln_core] write to the disk... 0.11 sec

[bwa_aln_core] 127401984 sequences have been processed.

[bwa_aln_core] calculate SA coordinate... 470.40 sec

[bwa_aln_core] write to the disk... 0.11 sec

[bwa_aln_core] 127664128 sequences have been processed.

[bwa_aln_core] calculate SA coordinate... 535.18 sec

[bwa_aln_core] write to the disk... 0.12 sec

[bwa_aln_core] 127926272 sequences have been processed.

[bwa_aln_core] calculate SA coordinate... 483.54 sec

[bwa_aln_core] write to the disk... 0.11 sec

[bwa_aln_core] 128188416 sequences have been processed.

[bwa_aln_core] calculate SA coordinate... 485.05 sec

[bwa_aln_core] write to the disk... 0.12 sec

[bwa_aln_core] 128450560 sequences have been processed.

[bwa_aln_core] calculate SA coordinate... 507.11 sec

[bwa_aln_core] write to the disk... 0.12 sec

[bwa_aln_core] 128712704 sequences have been processed.

[bwa_aln_core] calculate SA coordinate... 452.41 sec

[bwa_aln_core] write to the disk... 0.12 sec

[bwa_aln_core] 128974848 sequences have been processed.

[bwa_aln_core] calculate SA coordinate... 450.05 sec

[bwa_aln_core] write to the disk... 0.11 sec

[bwa_aln_core] 129236992 sequences have been processed.

[bwa_aln_core] calculate SA coordinate... 454.76 sec

[bwa_aln_core] write to the disk... 0.12 sec

[bwa_aln_core] 129499136 sequences have been processed.

[bwa_aln_core] calculate SA coordinate... 511.78 sec

[bwa_aln_core] write to the disk... 0.12 sec

[bwa_aln_core] 129761280 sequences have been processed.

[bwa_aln_core] calculate SA coordinate... 500.45 sec

[bwa_aln_core] write to the disk... 0.12 sec

[bwa_aln_core] 130023424 sequences have been processed.

[bwa_aln_core] calculate SA coordinate... 499.89 sec

[bwa_aln_core] write to the disk... 0.12 sec

[bwa_aln_core] 130285568 sequences have been processed.

[bwa_aln_core] calculate SA coordinate... 516.33 sec

[bwa_aln_core] write to the disk... 0.13 sec

[bwa_aln_core] 130547712 sequences have been processed.

[bwa_aln_core] calculate SA coordinate... 526.50 sec

[bwa_aln_core] write to the disk... 0.13 sec

[bwa_aln_core] 130809856 sequences have been processed.

[bwa_aln_core] calculate SA coordinate... 497.19 sec

[bwa_aln_core] write to the disk... 0.12 sec

[bwa_aln_core] 131072000 sequences have been processed.

[bwa_aln_core] calculate SA coordinate... 498.35 sec

[bwa_aln_core] write to the disk... 0.10 sec

[bwa_aln_core] 131334144 sequences have been processed.

[bwa_aln_core] calculate SA coordinate... 474.60 sec

[bwa_aln_core] write to the disk... 0.12 sec

[bwa_aln_core] 131596288 sequences have been processed.

[bwa_aln_core] calculate SA coordinate... 487.73 sec

[bwa_aln_core] write to the disk... 0.10 sec

[bwa_aln_core] 131858432 sequences have been processed.

[bwa_aln_core] calculate SA coordinate... 462.80 sec

[bwa_aln_core] write to the disk... 0.11 sec

[bwa_aln_core] 132120576 sequences have been processed.

[bwa_aln_core] calculate SA coordinate... 516.49 sec

[bwa_aln_core] write to the disk... 0.13 sec

[bwa_aln_core] 132382720 sequences have been processed.

[bwa_aln_core] calculate SA coordinate... 468.60 sec

[bwa_aln_core] write to the disk... 0.11 sec

[bwa_aln_core] 132644864 sequences have been processed.

[bwa_aln_core] calculate SA coordinate... 534.74 sec

[bwa_aln_core] write to the disk... 0.11 sec

[bwa_aln_core] 132907008 sequences have been processed.

[bwa_aln_core] calculate SA coordinate... 485.77 sec

[bwa_aln_core] write to the disk... 0.11 sec

[bwa_aln_core] 133169152 sequences have been processed.

[bwa_aln_core] calculate SA coordinate... 497.87 sec

[bwa_aln_core] write to the disk... 0.12 sec

[bwa_aln_core] 133431296 sequences have been processed.

[bwa_aln_core] calculate SA coordinate... 511.51 sec

[bwa_aln_core] write to the disk... 0.11 sec

[bwa_aln_core] 133693440 sequences have been processed.

[bwa_aln_core] calculate SA coordinate... 486.82 sec

[bwa_aln_core] write to the disk... 0.10 sec

[bwa_aln_core] 133955584 sequences have been processed.

[bwa_aln_core] calculate SA coordinate... 457.85 sec

[bwa_aln_core] write to the disk... 0.10 sec

[bwa_aln_core] 134217728 sequences have been processed.

[bwa_aln_core] calculate SA coordinate... 518.18 sec

[bwa_aln_core] write to the disk... 0.11 sec

[bwa_aln_core] 134479872 sequences have been processed.

[bwa_aln_core] calculate SA coordinate... 513.39 sec

[bwa_aln_core] write to the disk... 0.10 sec

[bwa_aln_core] 134742016 sequences have been processed.

[bwa_aln_core] calculate SA coordinate... 462.24 sec

[bwa_aln_core] write to the disk... 0.11 sec

[bwa_aln_core] 135004160 sequences have been processed.

[bwa_aln_core] calculate SA coordinate... 503.95 sec

[bwa_aln_core] write to the disk... 0.11 sec

[bwa_aln_core] 135266304 sequences have been processed.

[bwa_aln_core] calculate SA coordinate... 450.71 sec

[bwa_aln_core] write to the disk... 0.10 sec

[bwa_aln_core] 135528448 sequences have been processed.

[bwa_aln_core] calculate SA coordinate... 507.16 sec

[bwa_aln_core] write to the disk... 0.11 sec

[bwa_aln_core] 135790592 sequences have been processed.

[bwa_aln_core] calculate SA coordinate... 463.32 sec

[bwa_aln_core] write to the disk... 0.11 sec

[bwa_aln_core] 136052736 sequences have been processed.

[bwa_aln_core] calculate SA coordinate... 441.15 sec

[bwa_aln_core] write to the disk... 0.11 sec

[bwa_aln_core] 136314880 sequences have been processed.

[bwa_aln_core] calculate SA coordinate... 429.87 sec

[bwa_aln_core] write to the disk... 0.11 sec

[bwa_aln_core] 136577024 sequences have been processed.

[bwa_aln_core] calculate SA coordinate... 415.28 sec

[bwa_aln_core] write to the disk... 0.13 sec

[bwa_aln_core] 136839168 sequences have been processed.

[bwa_aln_core] calculate SA coordinate... 495.26 sec

[bwa_aln_core] write to the disk... 0.10 sec

[bwa_aln_core] 137101312 sequences have been processed.

[bwa_aln_core] calculate SA coordinate... 509.69 sec

[bwa_aln_core] write to the disk... 0.11 sec

[bwa_aln_core] 137363456 sequences have been processed.

[bwa_aln_core] calculate SA coordinate... 502.27 sec

[bwa_aln_core] write to the disk... 0.10 sec

[bwa_aln_core] 137625600 sequences have been processed.

[bwa_aln_core] calculate SA coordinate... 487.45 sec

[bwa_aln_core] write to the disk... 0.12 sec

[bwa_aln_core] 137887744 sequences have been processed.

[bwa_aln_core] calculate SA coordinate... 529.73 sec

[bwa_aln_core] write to the disk... 0.12 sec

[bwa_aln_core] 138149888 sequences have been processed.

[bwa_aln_core] calculate SA coordinate... 511.85 sec

[bwa_aln_core] write to the disk... 0.12 sec

[bwa_aln_core] 138412032 sequences have been processed.

[bwa_aln_core] calculate SA coordinate... 494.41 sec

[bwa_aln_core] write to the disk... 0.11 sec

[bwa_aln_core] 138674176 sequences have been processed.

[bwa_aln_core] calculate SA coordinate... 503.48 sec

[bwa_aln_core] write to the disk... 0.11 sec

[bwa_aln_core] 138936320 sequences have been processed.

[bwa_aln_core] calculate SA coordinate... 567.76 sec

[bwa_aln_core] write to the disk... 0.10 sec

[bwa_aln_core] 139198464 sequences have been processed.

[bwa_aln_core] calculate SA coordinate... 457.63 sec

[bwa_aln_core] write to the disk... 0.12 sec

[bwa_aln_core] 139460608 sequences have been processed.

[bwa_aln_core] calculate SA coordinate... 399.62 sec

[bwa_aln_core] write to the disk... 0.11 sec

[bwa_aln_core] 139722752 sequences have been processed.

[bwa_aln_core] calculate SA coordinate... 441.46 sec

[bwa_aln_core] write to the disk... 0.11 sec

[bwa_aln_core] 139984896 sequences have been processed.

[bwa_aln_core] calculate SA coordinate... 465.71 sec

[bwa_aln_core] write to the disk... 0.12 sec

[bwa_aln_core] 140247040 sequences have been processed.

[bwa_aln_core] calculate SA coordinate... 466.18 sec

[bwa_aln_core] write to the disk... 0.11 sec

[bwa_aln_core] 140509184 sequences have been processed.

[bwa_aln_core] calculate SA coordinate... 489.98 sec

[bwa_aln_core] write to the disk... 0.11 sec

[bwa_aln_core] 140771328 sequences have been processed.

[bwa_aln_core] calculate SA coordinate... 416.15 sec

[bwa_aln_core] write to the disk... 0.11 sec

[bwa_aln_core] 141033472 sequences have been processed.

[bwa_aln_core] calculate SA coordinate... 416.13 sec

[bwa_aln_core] write to the disk... 0.11 sec

[bwa_aln_core] 141295616 sequences have been processed.

[bwa_aln_core] calculate SA coordinate... 475.96 sec

[bwa_aln_core] write to the disk... 0.12 sec

[bwa_aln_core] 141557760 sequences have been processed.

[bwa_aln_core] calculate SA coordinate... 485.96 sec

[bwa_aln_core] write to the disk... 0.12 sec

[bwa_aln_core] 141819904 sequences have been processed.

[bwa_aln_core] calculate SA coordinate... 572.10 sec

[bwa_aln_core] write to the disk... 0.11 sec

[bwa_aln_core] 142082048 sequences have been processed.

[bwa_aln_core] calculate SA coordinate... 480.87 sec

[bwa_aln_core] write to the disk... 0.11 sec

[bwa_aln_core] 142344192 sequences have been processed.

[bwa_aln_core] calculate SA coordinate... 463.90 sec

[bwa_aln_core] write to the disk... 0.12 sec

[bwa_aln_core] 142606336 sequences have been processed.

[bwa_aln_core] calculate SA coordinate... 514.68 sec

[bwa_aln_core] write to the disk... 0.12 sec

[bwa_aln_core] 142868480 sequences have been processed.

[bwa_aln_core] calculate SA coordinate... 502.04 sec

[bwa_aln_core] write to the disk... 0.12 sec

[bwa_aln_core] 143130624 sequences have been processed.

[bwa_aln_core] calculate SA coordinate... 537.23 sec

[bwa_aln_core] write to the disk... 0.11 sec

[bwa_aln_core] 143392768 sequences have been processed.

[bwa_aln_core] calculate SA coordinate... 451.55 sec

[bwa_aln_core] write to the disk... 0.12 sec

[bwa_aln_core] 143654912 sequences have been processed.

[bwa_aln_core] calculate SA coordinate... 511.07 sec

[bwa_aln_core] write to the disk... 0.12 sec

[bwa_aln_core] 143917056 sequences have been processed.

[bwa_aln_core] calculate SA coordinate... 505.51 sec

[bwa_aln_core] write to the disk... 0.12 sec

[bwa_aln_core] 144179200 sequences have been processed.

[bwa_aln_core] calculate SA coordinate... 471.67 sec

[bwa_aln_core] write to the disk... 0.12 sec

[bwa_aln_core] 144441344 sequences have been processed.

[bwa_aln_core] calculate SA coordinate... 487.04 sec

[bwa_aln_core] write to the disk... 0.12 sec

[bwa_aln_core] 144703488 sequences have been processed.

[bwa_aln_core] calculate SA coordinate... 505.67 sec

[bwa_aln_core] write to the disk... 0.12 sec

[bwa_aln_core] 144965632 sequences have been processed.

[bwa_aln_core] calculate SA coordinate... 496.55 sec

[bwa_aln_core] write to the disk... 0.11 sec

[bwa_aln_core] 145227776 sequences have been processed.

[bwa_aln_core] calculate SA coordinate... 459.87 sec

[bwa_aln_core] write to the disk... 0.10 sec

[bwa_aln_core] 145489920 sequences have been processed.

[bwa_aln_core] calculate SA coordinate... 513.47 sec

[bwa_aln_core] write to the disk... 0.12 sec

[bwa_aln_core] 145752064 sequences have been processed.

[bwa_aln_core] calculate SA coordinate... 514.66 sec

[bwa_aln_core] write to the disk... 0.11 sec

[bwa_aln_core] 146014208 sequences have been processed.

[bwa_aln_core] calculate SA coordinate... 487.66 sec

[bwa_aln_core] write to the disk... 0.11 sec

[bwa_aln_core] 146276352 sequences have been processed.

[bwa_aln_core] calculate SA coordinate... 467.53 sec

[bwa_aln_core] write to the disk... 0.12 sec

[bwa_aln_core] 146538496 sequences have been processed.

[bwa_aln_core] calculate SA coordinate... 500.62 sec

[bwa_aln_core] write to the disk... 0.12 sec

[bwa_aln_core] 146800640 sequences have been processed.

[bwa_aln_core] calculate SA coordinate... 549.45 sec

[bwa_aln_core] write to the disk... 0.10 sec

[bwa_aln_core] 147062784 sequences have been processed.

[bwa_aln_core] calculate SA coordinate... 574.46 sec

[bwa_aln_core] write to the disk... 0.11 sec

[bwa_aln_core] 147324928 sequences have been processed.

[bwa_aln_core] calculate SA coordinate... 496.86 sec

[bwa_aln_core] write to the disk... 0.11 sec

[bwa_aln_core] 147587072 sequences have been processed.

[bwa_aln_core] calculate SA coordinate... 470.31 sec

[bwa_aln_core] write to the disk... 0.11 sec

[bwa_aln_core] 147849216 sequences have been processed.

[bwa_aln_core] calculate SA coordinate... 487.08 sec

[bwa_aln_core] write to the disk... 0.11 sec

[bwa_aln_core] 148111360 sequences have been processed.

[bwa_aln_core] calculate SA coordinate... 448.12 sec

[bwa_aln_core] write to the disk... 0.11 sec

[bwa_aln_core] 148373504 sequences have been processed.

[bwa_aln_core] calculate SA coordinate... 487.79 sec

[bwa_aln_core] write to the disk... 0.10 sec

[bwa_aln_core] 148635648 sequences have been processed.

[bwa_aln_core] calculate SA coordinate... 475.23 sec

[bwa_aln_core] write to the disk... 0.11 sec

[bwa_aln_core] 148897792 sequences have been processed.

[bwa_aln_core] calculate SA coordinate... 476.54 sec

[bwa_aln_core] write to the disk... 0.12 sec

[bwa_aln_core] 149159936 sequences have been processed.

[bwa_aln_core] calculate SA coordinate... 494.40 sec

[bwa_aln_core] write to the disk... 0.11 sec

[bwa_aln_core] 149422080 sequences have been processed.

[bwa_aln_core] calculate SA coordinate... 524.85 sec

[bwa_aln_core] write to the disk... 0.11 sec

[bwa_aln_core] 149684224 sequences have been processed.

[bwa_aln_core] calculate SA coordinate... 524.90 sec

[bwa_aln_core] write to the disk... 0.12 sec

[bwa_aln_core] 149946368 sequences have been processed.

[bwa_aln_core] calculate SA coordinate... 486.48 sec

[bwa_aln_core] write to the disk... 0.11 sec

[bwa_aln_core] 150208512 sequences have been processed.

[bwa_aln_core] calculate SA coordinate... 512.89 sec

[bwa_aln_core] write to the disk... 0.10 sec

[bwa_aln_core] 150470656 sequences have been processed.

[bwa_aln_core] calculate SA coordinate... 489.57 sec

[bwa_aln_core] write to the disk... 0.11 sec

[bwa_aln_core] 150732800 sequences have been processed.

[bwa_aln_core] calculate SA coordinate... 461.80 sec

[bwa_aln_core] write to the disk... 0.11 sec

[bwa_aln_core] 150994944 sequences have been processed.

[bwa_aln_core] calculate SA coordinate... 513.12 sec

[bwa_aln_core] write to the disk... 0.12 sec

[bwa_aln_core] 151257088 sequences have been processed.

[bwa_aln_core] calculate SA coordinate... 454.64 sec

[bwa_aln_core] write to the disk... 0.10 sec

[bwa_aln_core] 151519232 sequences have been processed.

[bwa_aln_core] calculate SA coordinate... 498.75 sec

[bwa_aln_core] write to the disk... 0.12 sec

[bwa_aln_core] 151781376 sequences have been processed.

[bwa_aln_core] calculate SA coordinate... 474.36 sec

[bwa_aln_core] write to the disk... 0.12 sec

[bwa_aln_core] 152043520 sequences have been processed.

[bwa_aln_core] calculate SA coordinate... 446.48 sec

[bwa_aln_core] write to the disk... 0.12 sec

[bwa_aln_core] 152305664 sequences have been processed.

[bwa_aln_core] calculate SA coordinate... 468.64 sec

[bwa_aln_core] write to the disk... 0.11 sec

[bwa_aln_core] 152567808 sequences have been processed.

[bwa_aln_core] calculate SA coordinate... 506.35 sec

[bwa_aln_core] write to the disk... 0.10 sec

[bwa_aln_core] 152829952 sequences have been processed.

[bwa_aln_core] calculate SA coordinate... 480.98 sec

[bwa_aln_core] write to the disk... 0.11 sec

[bwa_aln_core] 153092096 sequences have been processed.

[bwa_aln_core] calculate SA coordinate... 466.79 sec

[bwa_aln_core] write to the disk... 0.11 sec

[bwa_aln_core] 153354240 sequences have been processed.

[bwa_aln_core] calculate SA coordinate... 534.57 sec

[bwa_aln_core] write to the disk... 0.11 sec

[bwa_aln_core] 153616384 sequences have been processed.

[bwa_aln_core] calculate SA coordinate... 433.35 sec

[bwa_aln_core] write to the disk... 0.11 sec

[bwa_aln_core] 153878528 sequences have been processed.

[bwa_aln_core] calculate SA coordinate... 503.78 sec

[bwa_aln_core] write to the disk... 0.12 sec

[bwa_aln_core] 154140672 sequences have been processed.

[bwa_aln_core] calculate SA coordinate... 459.84 sec

[bwa_aln_core] write to the disk... 0.11 sec

[bwa_aln_core] 154402816 sequences have been processed.

[bwa_aln_core] calculate SA coordinate... 456.26 sec

[bwa_aln_core] write to the disk... 0.12 sec

[bwa_aln_core] 154664960 sequences have been processed.

[bwa_aln_core] calculate SA coordinate... 466.99 sec

[bwa_aln_core] write to the disk... 0.11 sec

[bwa_aln_core] 154927104 sequences have been processed.

[bwa_aln_core] calculate SA coordinate... 512.57 sec

[bwa_aln_core] write to the disk... 0.11 sec

[bwa_aln_core] 155189248 sequences have been processed.

[bwa_aln_core] calculate SA coordinate... 472.70 sec

[bwa_aln_core] write to the disk... 0.11 sec

[bwa_aln_core] 155451392 sequences have been processed.

[bwa_aln_core] calculate SA coordinate... 502.06 sec

[bwa_aln_core] write to the disk... 0.12 sec

[bwa_aln_core] 155713536 sequences have been processed.

[bwa_aln_core] calculate SA coordinate... 459.30 sec

[bwa_aln_core] write to the disk... 0.10 sec

[bwa_aln_core] 155975680 sequences have been processed.

[bwa_aln_core] calculate SA coordinate... 474.87 sec

[bwa_aln_core] write to the disk... 0.11 sec

[bwa_aln_core] 156237824 sequences have been processed.

[bwa_aln_core] calculate SA coordinate... 488.05 sec

[bwa_aln_core] write to the disk... 0.12 sec

[bwa_aln_core] 156499968 sequences have been processed.

[bwa_aln_core] calculate SA coordinate... 457.89 sec

[bwa_aln_core] write to the disk... 0.11 sec

[bwa_aln_core] 156762112 sequences have been processed.

[bwa_aln_core] calculate SA coordinate... 449.01 sec

[bwa_aln_core] write to the disk... 0.11 sec

[bwa_aln_core] 157024256 sequences have been processed.

[bwa_aln_core] calculate SA coordinate... 491.23 sec

[bwa_aln_core] write to the disk... 0.11 sec

[bwa_aln_core] 157286400 sequences have been processed.

[bwa_aln_core] calculate SA coordinate... 487.94 sec

[bwa_aln_core] write to the disk... 0.12 sec

[bwa_aln_core] 157548544 sequences have been processed.

[bwa_aln_core] calculate SA coordinate... 447.90 sec

[bwa_aln_core] write to the disk... 0.11 sec

[bwa_aln_core] 157810688 sequences have been processed.

[bwa_aln_core] calculate SA coordinate... 490.70 sec

[bwa_aln_core] write to the disk... 0.12 sec

[bwa_aln_core] 158072832 sequences have been processed.

[bwa_aln_core] calculate SA coordinate... 464.18 sec

[bwa_aln_core] write to the disk... 0.12 sec

[bwa_aln_core] 158334976 sequences have been processed.

[bwa_aln_core] calculate SA coordinate... 505.31 sec

[bwa_aln_core] write to the disk... 0.12 sec

[bwa_aln_core] 158597120 sequences have been processed.

[bwa_aln_core] calculate SA coordinate... 483.21 sec

[bwa_aln_core] write to the disk... 0.10 sec

[bwa_aln_core] 158859264 sequences have been processed.

[bwa_aln_core] calculate SA coordinate... 466.78 sec

[bwa_aln_core] write to the disk... 0.12 sec

[bwa_aln_core] 159121408 sequences have been processed.

[bwa_aln_core] calculate SA coordinate... 457.09 sec

[bwa_aln_core] write to the disk... 0.12 sec

[bwa_aln_core] 159383552 sequences have been processed.

[bwa_aln_core] calculate SA coordinate... 493.70 sec

[bwa_aln_core] write to the disk... 0.11 sec

[bwa_aln_core] 159645696 sequences have been processed.

[bwa_aln_core] calculate SA coordinate... 564.27 sec

[bwa_aln_core] write to the disk... 0.11 sec

[bwa_aln_core] 159907840 sequences have been processed.

[bwa_aln_core] calculate SA coordinate... 608.39 sec

[bwa_aln_core] write to the disk... 0.11 sec

[bwa_aln_core] 160169984 sequences have been processed.

[bwa_aln_core] calculate SA coordinate... 455.43 sec

[bwa_aln_core] write to the disk... 0.10 sec

[bwa_aln_core] 160432128 sequences have been processed.

[bwa_aln_core] calculate SA coordinate... 476.78 sec

[bwa_aln_core] write to the disk... 0.11 sec

[bwa_aln_core] 160694272 sequences have been processed.

[bwa_aln_core] calculate SA coordinate... 465.46 sec

[bwa_aln_core] write to the disk... 0.12 sec

[bwa_aln_core] 160956416 sequences have been processed.

[bwa_aln_core] calculate SA coordinate... 468.47 sec

[bwa_aln_core] write to the disk... 0.12 sec

[bwa_aln_core] 161218560 sequences have been processed.

[bwa_aln_core] calculate SA coordinate... 465.19 sec

[bwa_aln_core] write to the disk... 0.11 sec

[bwa_aln_core] 161480704 sequences have been processed.

[bwa_aln_core] calculate SA coordinate... 484.42 sec

[bwa_aln_core] write to the disk... 0.11 sec

[bwa_aln_core] 161742848 sequences have been processed.

[bwa_aln_core] calculate SA coordinate... 493.09 sec

[bwa_aln_core] write to the disk... 0.11 sec

[bwa_aln_core] 162004992 sequences have been processed.

[bwa_aln_core] calculate SA coordinate... 527.11 sec

[bwa_aln_core] write to the disk... 0.12 sec

[bwa_aln_core] 162267136 sequences have been processed.

[bwa_aln_core] calculate SA coordinate... 489.85 sec

[bwa_aln_core] write to the disk... 0.11 sec

[bwa_aln_core] 162529280 sequences have been processed.

[bwa_aln_core] calculate SA coordinate... 490.56 sec

[bwa_aln_core] write to the disk... 0.13 sec

[bwa_aln_core] 162791424 sequences have been processed.

[bwa_aln_core] calculate SA coordinate... 480.24 sec

[bwa_aln_core] write to the disk... 0.11 sec

[bwa_aln_core] 163053568 sequences have been processed.

[bwa_aln_core] calculate SA coordinate... 478.04 sec

[bwa_aln_core] write to the disk... 0.13 sec

[bwa_aln_core] 163315712 sequences have been processed.

[bwa_aln_core] calculate SA coordinate... 489.60 sec

[bwa_aln_core] write to the disk... 0.12 sec

[bwa_aln_core] 163577856 sequences have been processed.

[bwa_aln_core] calculate SA coordinate... 514.48 sec

[bwa_aln_core] write to the disk... 0.11 sec

[bwa_aln_core] 163840000 sequences have been processed.

[bwa_aln_core] calculate SA coordinate... 473.33 sec

[bwa_aln_core] write to the disk... 0.11 sec

[bwa_aln_core] 164102144 sequences have been processed.

[bwa_aln_core] calculate SA coordinate... 502.54 sec

[bwa_aln_core] write to the disk... 0.11 sec

[bwa_aln_core] 164364288 sequences have been processed.

[bwa_aln_core] calculate SA coordinate... 482.00 sec

[bwa_aln_core] write to the disk... 0.12 sec

[bwa_aln_core] 164626432 sequences have been processed.

[bwa_aln_core] calculate SA coordinate... 497.38 sec

[bwa_aln_core] write to the disk... 0.11 sec

[bwa_aln_core] 164888576 sequences have been processed.

[bwa_aln_core] calculate SA coordinate... 474.76 sec

[bwa_aln_core] write to the disk... 0.11 sec

[bwa_aln_core] 165150720 sequences have been processed.

[bwa_aln_core] calculate SA coordinate... 496.95 sec

[bwa_aln_core] write to the disk... 0.10 sec

[bwa_aln_core] 165412864 sequences have been processed.

[bwa_aln_core] calculate SA coordinate... 467.72 sec

[bwa_aln_core] write to the disk... 0.10 sec

[bwa_aln_core] 165675008 sequences have been processed.

[bwa_aln_core] calculate SA coordinate... 544.50 sec

[bwa_aln_core] write to the disk... 0.12 sec

[bwa_aln_core] 165937152 sequences have been processed.

[bwa_aln_core] calculate SA coordinate... 556.00 sec

[bwa_aln_core] write to the disk... 0.11 sec

[bwa_aln_core] 166199296 sequences have been processed.

[bwa_aln_core] calculate SA coordinate... 490.06 sec

[bwa_aln_core] write to the disk... 0.11 sec

[bwa_aln_core] 166461440 sequences have been processed.

[bwa_aln_core] calculate SA coordinate... 516.63 sec

[bwa_aln_core] write to the disk... 0.10 sec

[bwa_aln_core] 166723584 sequences have been processed.

[bwa_aln_core] calculate SA coordinate... 517.46 sec

[bwa_aln_core] write to the disk... 0.11 sec

[bwa_aln_core] 166985728 sequences have been processed.

[bwa_aln_core] calculate SA coordinate... 487.50 sec

[bwa_aln_core] write to the disk... 0.12 sec

[bwa_aln_core] 167247872 sequences have been processed.

[bwa_aln_core] calculate SA coordinate... 453.21 sec

[bwa_aln_core] write to the disk... 0.12 sec

[bwa_aln_core] 167510016 sequences have been processed.

[bwa_aln_core] calculate SA coordinate... 482.73 sec

[bwa_aln_core] write to the disk... 0.12 sec

[bwa_aln_core] 167772160 sequences have been processed.

[bwa_aln_core] calculate SA coordinate... 495.09 sec

[bwa_aln_core] write to the disk... 0.11 sec

[bwa_aln_core] 168034304 sequences have been processed.

[bwa_aln_core] calculate SA coordinate... 476.37 sec

[bwa_aln_core] write to the disk... 0.11 sec

[bwa_aln_core] 168296448 sequences have been processed.

[bwa_aln_core] calculate SA coordinate... 472.24 sec

[bwa_aln_core] write to the disk... 0.10 sec

[bwa_aln_core] 168558592 sequences have been processed.

[bwa_aln_core] calculate SA coordinate... 481.62 sec

[bwa_aln_core] write to the disk... 0.11 sec

[bwa_aln_core] 168820736 sequences have been processed.

[bwa_aln_core] calculate SA coordinate... 445.67 sec

[bwa_aln_core] write to the disk... 0.12 sec

[bwa_aln_core] 169082880 sequences have been processed.

[bwa_aln_core] calculate SA coordinate... 504.00 sec

[bwa_aln_core] write to the disk... 0.12 sec

[bwa_aln_core] 169345024 sequences have been processed.

[bwa_aln_core] calculate SA coordinate... 490.27 sec

[bwa_aln_core] write to the disk... 0.13 sec

[bwa_aln_core] 169607168 sequences have been processed.

[bwa_aln_core] calculate SA coordinate... 451.67 sec

[bwa_aln_core] write to the disk... 0.12 sec

[bwa_aln_core] 169869312 sequences have been processed.

[bwa_aln_core] calculate SA coordinate... 491.96 sec

[bwa_aln_core] write to the disk... 0.12 sec

[bwa_aln_core] 170131456 sequences have been processed.

[bwa_aln_core] calculate SA coordinate... 517.88 sec

[bwa_aln_core] write to the disk... 0.12 sec

[bwa_aln_core] 170393600 sequences have been processed.

[bwa_aln_core] calculate SA coordinate... 523.31 sec

[bwa_aln_core] write to the disk... 0.12 sec

[bwa_aln_core] 170655744 sequences have been processed.

[bwa_aln_core] calculate SA coordinate... 417.26 sec

[bwa_aln_core] write to the disk... 0.13 sec

[bwa_aln_core] 170917888 sequences have been processed.

[bwa_aln_core] calculate SA coordinate... 467.01 sec

[bwa_aln_core] write to the disk... 0.12 sec

[bwa_aln_core] 171180032 sequences have been processed.

[bwa_aln_core] calculate SA coordinate... 458.12 sec

[bwa_aln_core] write to the disk... 0.11 sec

[bwa_aln_core] 171442176 sequences have been processed.

[bwa_aln_core] calculate SA coordinate... 512.14 sec

[bwa_aln_core] write to the disk... 0.11 sec

[bwa_aln_core] 171704320 sequences have been processed.

[bwa_aln_core] calculate SA coordinate... 504.19 sec

[bwa_aln_core] write to the disk... 0.12 sec

[bwa_aln_core] 171966464 sequences have been processed.

[bwa_aln_core] calculate SA coordinate... 527.38 sec

[bwa_aln_core] write to the disk... 0.12 sec

[bwa_aln_core] 172228608 sequences have been processed.

[bwa_aln_core] calculate SA coordinate... 464.72 sec

[bwa_aln_core] write to the disk... 0.11 sec

[bwa_aln_core] 172490752 sequences have been processed.

[bwa_aln_core] calculate SA coordinate... 507.89 sec

[bwa_aln_core] write to the disk... 0.12 sec

[bwa_aln_core] 172752896 sequences have been processed.

[bwa_aln_core] calculate SA coordinate... 226.24 sec

[bwa_aln_core] write to the disk... 0.05 sec

[bwa_aln_core] 172870680 sequences have been processed.

[main] Version: 0.6.2-r126

[main] CMD: /sapfs/actrec/ngs_tools/bwa-0.6.2/bwa aln -t 50 -f /sapfs/actrec/users/pratik/HPVDetector/siha_wgs/SRR1609142_2.sai /sapfs/actrec/users/pratik/HPVDetector/HPVDetector_v1.0/Human_HPV_index_files/human_HPV /sapfs/actrec/users/pratik/down/siha/SRR1609142_2.fastq

[main] Real time: 12208.871 sec; CPU: 324230.522 sec

[bwa_sai2sam_pe_core] convert to sequence coordinate...

[infer_isize] (25, 50, 75) percentile: (453, 463, 473)

[infer_isize] low and high boundaries: 413 and 513 for estimating avg and std

[infer_isize] inferred external isize from 205111 pairs: 463.286 +/- 13.972

[infer_isize] skewness: 0.003; kurtosis: -0.131; ap_prior: 1.73e-05

[infer_isize] inferred maximum insert size: 561 (6.96 sigma)

[bwa_sai2sam_pe_core] time elapses: 38.09 sec

[bwa_sai2sam_pe_core] changing coordinates of 6233 alignments.

[bwa_sai2sam_pe_core] align unmapped mate...

[bwa_paired_sw] 10137 out of 10724 Q17 singletons are mated.

[bwa_paired_sw] 926 out of 1547 Q17 discordant pairs are fixed.

[bwa_sai2sam_pe_core] time elapses: 5.07 sec

[bwa_sai2sam_pe_core] refine gapped alignments... 1.07 sec

[bwa_sai2sam_pe_core] print alignments... 2.26 sec

[bwa_sai2sam_pe_core] 262144 sequences have been processed.

[bwa_sai2sam_pe_core] convert to sequence coordinate...

[infer_isize] (25, 50, 75) percentile: (453, 463, 473)

[infer_isize] low and high boundaries: 413 and 513 for estimating avg and std

[infer_isize] inferred external isize from 205374 pairs: 463.287 +/- 13.971

[infer_isize] skewness: 0.004; kurtosis: -0.131; ap_prior: 1.67e-05

[infer_isize] inferred maximum insert size: 561 (6.96 sigma)

[bwa_sai2sam_pe_core] time elapses: 34.21 sec

[bwa_sai2sam_pe_core] changing coordinates of 6073 alignments.

[bwa_sai2sam_pe_core] align unmapped mate...

[bwa_paired_sw] 10520 out of 11057 Q17 singletons are mated.

[bwa_paired_sw] 892 out of 1488 Q17 discordant pairs are fixed.

[bwa_sai2sam_pe_core] time elapses: 4.80 sec

[bwa_sai2sam_pe_core] refine gapped alignments... 1.03 sec

[bwa_sai2sam_pe_core] print alignments... 2.16 sec

[bwa_sai2sam_pe_core] 524288 sequences have been processed.

[bwa_sai2sam_pe_core] convert to sequence coordinate...

[infer_isize] (25, 50, 75) percentile: (453, 463, 473)

[infer_isize] low and high boundaries: 413 and 513 for estimating avg and std

[infer_isize] inferred external isize from 204904 pairs: 463.278 +/- 13.945

[infer_isize] skewness: 0.007; kurtosis: -0.138; ap_prior: 1.72e-05

[infer_isize] inferred maximum insert size: 560 (6.96 sigma)

[bwa_sai2sam_pe_core] time elapses: 33.65 sec

[bwa_sai2sam_pe_core] changing coordinates of 5868 alignments.

[bwa_sai2sam_pe_core] align unmapped mate...

[bwa_paired_sw] 10858 out of 11422 Q17 singletons are mated.

[bwa_paired_sw] 924 out of 1495 Q17 discordant pairs are fixed.

[bwa_sai2sam_pe_core] time elapses: 4.81 sec

[bwa_sai2sam_pe_core] refine gapped alignments... 0.97 sec

[bwa_sai2sam_pe_core] print alignments... 2.09 sec

[bwa_sai2sam_pe_core] 786432 sequences have been processed.

[bwa_sai2sam_pe_core] convert to sequence coordinate...

[infer_isize] (25, 50, 75) percentile: (453, 463, 473)

[infer_isize] low and high boundaries: 413 and 513 for estimating avg and std

[infer_isize] inferred external isize from 205868 pairs: 463.308 +/- 13.933

[infer_isize] skewness: 0.002; kurtosis: -0.144; ap_prior: 1.87e-05

[infer_isize] inferred maximum insert size: 560 (6.96 sigma)

[bwa_sai2sam_pe_core] time elapses: 33.23 sec

[bwa_sai2sam_pe_core] changing coordinates of 5926 alignments.

[bwa_sai2sam_pe_core] align unmapped mate...

[bwa_paired_sw] 10104 out of 10699 Q17 singletons are mated.

[bwa_paired_sw] 915 out of 1548 Q17 discordant pairs are fixed.

[bwa_sai2sam_pe_core] time elapses: 4.62 sec

[bwa_sai2sam_pe_core] refine gapped alignments... 0.97 sec

[bwa_sai2sam_pe_core] print alignments... 2.23 sec

[bwa_sai2sam_pe_core] 1048576 sequences have been processed.

[bwa_sai2sam_pe_core] convert to sequence coordinate...

[infer_isize] (25, 50, 75) percentile: (453, 463, 473)

[infer_isize] low and high boundaries: 413 and 513 for estimating avg and std

[infer_isize] inferred external isize from 205250 pairs: 463.317 +/- 13.916

[infer_isize] skewness: 0.007; kurtosis: -0.138; ap_prior: 1.79e-05

[infer_isize] inferred maximum insert size: 560 (6.96 sigma)

[bwa_sai2sam_pe_core] time elapses: 33.98 sec

[bwa_sai2sam_pe_core] changing coordinates of 6178 alignments.

[bwa_sai2sam_pe_core] align unmapped mate...

[bwa_paired_sw] 10061 out of 10667 Q17 singletons are mated.

[bwa_paired_sw] 886 out of 1511 Q17 discordant pairs are fixed.

[bwa_sai2sam_pe_core] time elapses: 4.60 sec

[bwa_sai2sam_pe_core] refine gapped alignments... 0.98 sec

[bwa_sai2sam_pe_core] print alignments... 2.20 sec

[bwa_sai2sam_pe_core] 1310720 sequences have been processed.

[bwa_sai2sam_pe_core] convert to sequence coordinate...

[infer_isize] (25, 50, 75) percentile: (453, 463, 473)

[infer_isize] low and high boundaries: 413 and 513 for estimating avg and std

[infer_isize] inferred external isize from 205191 pairs: 463.257 +/- 13.932

[infer_isize] skewness: 0.007; kurtosis: -0.141; ap_prior: 1.72e-05

[infer_isize] inferred maximum insert size: 560 (6.96 sigma)

[bwa_sai2sam_pe_core] time elapses: 33.66 sec

[bwa_sai2sam_pe_core] changing coordinates of 5781 alignments.

[bwa_sai2sam_pe_core] align unmapped mate...

[bwa_paired_sw] 10555 out of 11114 Q17 singletons are mated.

[bwa_paired_sw] 883 out of 1439 Q17 discordant pairs are fixed.

[bwa_sai2sam_pe_core] time elapses: 4.70 sec

[bwa_sai2sam_pe_core] refine gapped alignments... 0.99 sec

[bwa_sai2sam_pe_core] print alignments... 2.23 sec

[bwa_sai2sam_pe_core] 1572864 sequences have been processed.

[bwa_sai2sam_pe_core] convert to sequence coordinate...

[infer_isize] (25, 50, 75) percentile: (453, 463, 473)

[infer_isize] low and high boundaries: 413 and 513 for estimating avg and std

[infer_isize] inferred external isize from 204823 pairs: 463.238 +/- 13.937

[infer_isize] skewness: 0.007; kurtosis: -0.152; ap_prior: 1.90e-05

[infer_isize] inferred maximum insert size: 560 (6.96 sigma)

[bwa_sai2sam_pe_core] time elapses: 34.41 sec

[bwa_sai2sam_pe_core] changing coordinates of 5918 alignments.

[bwa_sai2sam_pe_core] align unmapped mate...

[bwa_paired_sw] 10558 out of 11141 Q17 singletons are mated.

[bwa_paired_sw] 900 out of 1505 Q17 discordant pairs are fixed.

[bwa_sai2sam_pe_core] time elapses: 4.81 sec

[bwa_sai2sam_pe_core] refine gapped alignments... 1.04 sec

[bwa_sai2sam_pe_core] print alignments... 2.33 sec

[bwa_sai2sam_pe_core] 1835008 sequences have been processed.

[bwa_sai2sam_pe_core] convert to sequence coordinate...

[infer_isize] (25, 50, 75) percentile: (453, 463, 473)

[infer_isize] low and high boundaries: 413 and 513 for estimating avg and std

[infer_isize] inferred external isize from 205364 pairs: 463.363 +/- 13.968

[infer_isize] skewness: -0.002; kurtosis: -0.148; ap_prior: 1.66e-05

[infer_isize] inferred maximum insert size: 561 (6.96 sigma)

[bwa_sai2sam_pe_core] time elapses: 35.52 sec

[bwa_sai2sam_pe_core] changing coordinates of 6094 alignments.

[bwa_sai2sam_pe_core] align unmapped mate...

[bwa_paired_sw] 10334 out of 10880 Q17 singletons are mated.

[bwa_paired_sw] 891 out of 1518 Q17 discordant pairs are fixed.

[bwa_sai2sam_pe_core] time elapses: 4.77 sec

[bwa_sai2sam_pe_core] refine gapped alignments... 1.05 sec

[bwa_sai2sam_pe_core] print alignments... 2.33 sec

[bwa_sai2sam_pe_core] 2097152 sequences have been processed.

[bwa_sai2sam_pe_core] convert to sequence coordinate...

[infer_isize] (25, 50, 75) percentile: (453, 463, 473)

[infer_isize] low and high boundaries: 413 and 513 for estimating avg and std

[infer_isize] inferred external isize from 204337 pairs: 463.230 +/- 13.971

[infer_isize] skewness: 0.002; kurtosis: -0.123; ap_prior: 1.77e-05

[infer_isize] inferred maximum insert size: 560 (6.96 sigma)

[bwa_sai2sam_pe_core] time elapses: 34.47 sec

[bwa_sai2sam_pe_core] changing coordinates of 5960 alignments.

[bwa_sai2sam_pe_core] align unmapped mate...

[bwa_paired_sw] 10784 out of 11294 Q17 singletons are mated.

[bwa_paired_sw] 896 out of 1503 Q17 discordant pairs are fixed.

[bwa_sai2sam_pe_core] time elapses: 4.86 sec

[bwa_sai2sam_pe_core] refine gapped alignments... 1.07 sec

[bwa_sai2sam_pe_core] print alignments... 2.35 sec

[bwa_sai2sam_pe_core] 2359296 sequences have been processed.

[bwa_sai2sam_pe_core] convert to sequence coordinate...

[infer_isize] (25, 50, 75) percentile: (453, 463, 473)

[infer_isize] low and high boundaries: 413 and 513 for estimating avg and std

[infer_isize] inferred external isize from 205646 pairs: 463.229 +/- 13.957

[infer_isize] skewness: -0.002; kurtosis: -0.125; ap_prior: 1.83e-05

[infer_isize] inferred maximum insert size: 560 (6.96 sigma)

[bwa_sai2sam_pe_core] time elapses: 35.71 sec

[bwa_sai2sam_pe_core] changing coordinates of 5967 alignments.

[bwa_sai2sam_pe_core] align unmapped mate...

[bwa_paired_sw] 10075 out of 10635 Q17 singletons are mated.

[bwa_paired_sw] 863 out of 1439 Q17 discordant pairs are fixed.

[bwa_sai2sam_pe_core] time elapses: 4.72 sec

[bwa_sai2sam_pe_core] refine gapped alignments... 1.09 sec

[bwa_sai2sam_pe_core] print alignments... 2.32 sec

[bwa_sai2sam_pe_core] 2621440 sequences have been processed.

[bwa_sai2sam_pe_core] convert to sequence coordinate...

[infer_isize] (25, 50, 75) percentile: (453, 463, 473)

[infer_isize] low and high boundaries: 413 and 513 for estimating avg and std

[infer_isize] inferred external isize from 205181 pairs: 463.303 +/- 13.935

[infer_isize] skewness: 0.001; kurtosis: -0.147; ap_prior: 1.73e-05

[infer_isize] inferred maximum insert size: 560 (6.96 sigma)

[bwa_sai2sam_pe_core] time elapses: 37.75 sec

[bwa_sai2sam_pe_core] changing coordinates of 6030 alignments.

[bwa_sai2sam_pe_core] align unmapped mate...

[bwa_paired_sw] 10072 out of 10707 Q17 singletons are mated.

[bwa_paired_sw] 838 out of 1485 Q17 discordant pairs are fixed.

[bwa_sai2sam_pe_core] time elapses: 4.68 sec

[bwa_sai2sam_pe_core] refine gapped alignments... 1.09 sec

[bwa_sai2sam_pe_core] print alignments... 2.23 sec

[bwa_sai2sam_pe_core] 2883584 sequences have been processed.

[bwa_sai2sam_pe_core] convert to sequence coordinate...

[infer_isize] (25, 50, 75) percentile: (453, 463, 473)

[infer_isize] low and high boundaries: 413 and 513 for estimating avg and std

[infer_isize] inferred external isize from 205348 pairs: 463.281 +/- 13.968

[infer_isize] skewness: -0.008; kurtosis: -0.148; ap_prior: 1.67e-05

[infer_isize] inferred maximum insert size: 560 (6.96 sigma)

[bwa_sai2sam_pe_core] time elapses: 40.69 sec

[bwa_sai2sam_pe_core] changing coordinates of 6104 alignments.

[bwa_sai2sam_pe_core] align unmapped mate...

[bwa_paired_sw] 10300 out of 10887 Q17 singletons are mated.

[bwa_paired_sw] 925 out of 1546 Q17 discordant pairs are fixed.

[bwa_sai2sam_pe_core] time elapses: 4.68 sec

[bwa_sai2sam_pe_core] refine gapped alignments... 1.06 sec

[bwa_sai2sam_pe_core] print alignments... 2.20 sec

[bwa_sai2sam_pe_core] 3145728 sequences have been processed.

[bwa_sai2sam_pe_core] convert to sequence coordinate...

[infer_isize] (25, 50, 75) percentile: (453, 463, 473)

[infer_isize] low and high boundaries: 413 and 513 for estimating avg and std

[infer_isize] inferred external isize from 205382 pairs: 463.284 +/- 13.949

[infer_isize] skewness: 0.005; kurtosis: -0.161; ap_prior: 1.82e-05

[infer_isize] inferred maximum insert size: 560 (6.96 sigma)

[bwa_sai2sam_pe_core] time elapses: 33.44 sec

[bwa_sai2sam_pe_core] changing coordinates of 6056 alignments.

[bwa_sai2sam_pe_core] align unmapped mate...

[bwa_paired_sw] 9946 out of 10564 Q17 singletons are mated.

[bwa_paired_sw] 904 out of 1499 Q17 discordant pairs are fixed.

[bwa_sai2sam_pe_core] time elapses: 4.48 sec

[bwa_sai2sam_pe_core] refine gapped alignments... 0.93 sec

[bwa_sai2sam_pe_core] print alignments... 2.08 sec

[bwa_sai2sam_pe_core] 3407872 sequences have been processed.

[bwa_sai2sam_pe_core] convert to sequence coordinate...

[infer_isize] (25, 50, 75) percentile: (453, 463, 473)

[infer_isize] low and high boundaries: 413 and 513 for estimating avg and std

[infer_isize] inferred external isize from 204968 pairs: 463.282 +/- 13.953

[infer_isize] skewness: 0.014; kurtosis: -0.144; ap_prior: 1.76e-05

[infer_isize] inferred maximum insert size: 560 (6.96 sigma)

[bwa_sai2sam_pe_core] time elapses: 32.20 sec

[bwa_sai2sam_pe_core] changing coordinates of 5925 alignments.

[bwa_sai2sam_pe_core] align unmapped mate...

[bwa_paired_sw] 10581 out of 11134 Q17 singletons are mated.

[bwa_paired_sw] 957 out of 1543 Q17 discordant pairs are fixed.

[bwa_sai2sam_pe_core] time elapses: 4.65 sec

[bwa_sai2sam_pe_core] refine gapped alignments... 0.96 sec

[bwa_sai2sam_pe_core] print alignments... 2.11 sec

[bwa_sai2sam_pe_core] 3670016 sequences have been processed.

[bwa_sai2sam_pe_core] convert to sequence coordinate...

[infer_isize] (25, 50, 75) percentile: (453, 463, 473)

[infer_isize] low and high boundaries: 413 and 513 for estimating avg and std

[infer_isize] inferred external isize from 205256 pairs: 463.260 +/- 13.933

[infer_isize] skewness: -0.006; kurtosis: -0.129; ap_prior: 1.93e-05

[infer_isize] inferred maximum insert size: 560 (6.96 sigma)

[bwa_sai2sam_pe_core] time elapses: 32.76 sec

[bwa_sai2sam_pe_core] changing coordinates of 6026 alignments.

[bwa_sai2sam_pe_core] align unmapped mate...

[bwa_paired_sw] 10129 out of 10719 Q17 singletons are mated.

[bwa_paired_sw] 860 out of 1430 Q17 discordant pairs are fixed.

[bwa_sai2sam_pe_core] time elapses: 4.50 sec

[bwa_sai2sam_pe_core] refine gapped alignments... 0.98 sec

[bwa_sai2sam_pe_core] print alignments... 2.08 sec

[bwa_sai2sam_pe_core] 3932160 sequences have been processed.

[bwa_sai2sam_pe_core] convert to sequence coordinate...

[infer_isize] (25, 50, 75) percentile: (453, 463, 473)

[infer_isize] low and high boundaries: 413 and 513 for estimating avg and std

[infer_isize] inferred external isize from 205613 pairs: 463.264 +/- 13.958

[infer_isize] skewness: 0.002; kurtosis: -0.134; ap_prior: 1.65e-05

[infer_isize] inferred maximum insert size: 560 (6.96 sigma)

[bwa_sai2sam_pe_core] time elapses: 34.82 sec

[bwa_sai2sam_pe_core] changing coordinates of 6108 alignments.

[bwa_sai2sam_pe_core] align unmapped mate...

[bwa_paired_sw] 9910 out of 10487 Q17 singletons are mated.

[bwa_paired_sw] 923 out of 1527 Q17 discordant pairs are fixed.

[bwa_sai2sam_pe_core] time elapses: 4.62 sec

[bwa_sai2sam_pe_core] refine gapped alignments... 1.04 sec

[bwa_sai2sam_pe_core] print alignments... 2.14 sec

[bwa_sai2sam_pe_core] 4194304 sequences have been processed.

[bwa_sai2sam_pe_core] convert to sequence coordinate...

[infer_isize] (25, 50, 75) percentile: (453, 463, 473)

[infer_isize] low and high boundaries: 413 and 513 for estimating avg and std

[infer_isize] inferred external isize from 205260 pairs: 463.244 +/- 13.943

[infer_isize] skewness: -0.002; kurtosis: -0.141; ap_prior: 1.69e-05

[infer_isize] inferred maximum insert size: 560 (6.96 sigma)

[bwa_sai2sam_pe_core] time elapses: 34.56 sec

[bwa_sai2sam_pe_core] changing coordinates of 5969 alignments.

[bwa_sai2sam_pe_core] align unmapped mate...

[bwa_paired_sw] 10343 out of 10880 Q17 singletons are mated.

[bwa_paired_sw] 850 out of 1450 Q17 discordant pairs are fixed.

[bwa_sai2sam_pe_core] time elapses: 4.69 sec

[bwa_sai2sam_pe_core] refine gapped alignments... 1.04 sec

[bwa_sai2sam_pe_core] print alignments... 2.09 sec

[bwa_sai2sam_pe_core] 4456448 sequences have been processed.

[bwa_sai2sam_pe_core] convert to sequence coordinate...

[infer_isize] (25, 50, 75) percentile: (453, 463, 473)

[infer_isize] low and high boundaries: 413 and 513 for estimating avg and std

[infer_isize] inferred external isize from 205103 pairs: 463.233 +/- 13.963

[infer_isize] skewness: 0.003; kurtosis: -0.132; ap_prior: 1.70e-05

[infer_isize] inferred maximum insert size: 560 (6.96 sigma)

[bwa_sai2sam_pe_core] time elapses: 34.09 sec

[bwa_sai2sam_pe_core] changing coordinates of 6158 alignments.

[bwa_sai2sam_pe_core] align unmapped mate...

[bwa_paired_sw] 10138 out of 10668 Q17 singletons are mated.

[bwa_paired_sw] 856 out of 1467 Q17 discordant pairs are fixed.

[bwa_sai2sam_pe_core] time elapses: 4.64 sec

[bwa_sai2sam_pe_core] refine gapped alignments... 0.98 sec

[bwa_sai2sam_pe_core] print alignments... 2.22 sec

[bwa_sai2sam_pe_core] 4718592 sequences have been processed.

[bwa_sai2sam_pe_core] convert to sequence coordinate...

[infer_isize] (25, 50, 75) percentile: (453, 463, 473)

[infer_isize] low and high boundaries: 413 and 513 for estimating avg and std

[infer_isize] inferred external isize from 205471 pairs: 463.217 +/- 13.929

[infer_isize] skewness: -0.002; kurtosis: -0.145; ap_prior: 1.71e-05

[infer_isize] inferred maximum insert size: 560 (6.96 sigma)

[bwa_sai2sam_pe_core] time elapses: 35.41 sec

[bwa_sai2sam_pe_core] changing coordinates of 6103 alignments.

[bwa_sai2sam_pe_core] align unmapped mate...

[bwa_paired_sw] 9921 out of 10478 Q17 singletons are mated.

[bwa_paired_sw] 917 out of 1525 Q17 discordant pairs are fixed.

[bwa_sai2sam_pe_core] time elapses: 4.63 sec

[bwa_sai2sam_pe_core] refine gapped alignments... 1.04 sec

[bwa_sai2sam_pe_core] print alignments... 2.24 sec

[bwa_sai2sam_pe_core] 4980736 sequences have been processed.

[bwa_sai2sam_pe_core] convert to sequence coordinate...

[infer_isize] (25, 50, 75) percentile: (453, 463, 473)

[infer_isize] low and high boundaries: 413 and 513 for estimating avg and std

[infer_isize] inferred external isize from 205582 pairs: 463.209 +/- 13.932

[infer_isize] skewness: 0.001; kurtosis: -0.131; ap_prior: 1.75e-05

[infer_isize] inferred maximum insert size: 560 (6.96 sigma)

[bwa_sai2sam_pe_core] time elapses: 34.74 sec

[bwa_sai2sam_pe_core] changing coordinates of 6097 alignments.

[bwa_sai2sam_pe_core] align unmapped mate...

[bwa_paired_sw] 9987 out of 10509 Q17 singletons are mated.

[bwa_paired_sw] 873 out of 1489 Q17 discordant pairs are fixed.

[bwa_sai2sam_pe_core] time elapses: 4.67 sec

[bwa_sai2sam_pe_core] refine gapped alignments... 1.06 sec

[bwa_sai2sam_pe_core] print alignments... 2.28 sec

[bwa_sai2sam_pe_core] 5242880 sequences have been processed.

[bwa_sai2sam_pe_core] convert to sequence coordinate...

[infer_isize] (25, 50, 75) percentile: (453, 463, 473)

[infer_isize] low and high boundaries: 413 and 513 for estimating avg and std

[infer_isize] inferred external isize from 205423 pairs: 463.297 +/- 13.952

[infer_isize] skewness: 0.000; kurtosis: -0.140; ap_prior: 1.88e-05

[infer_isize] inferred maximum insert size: 560 (6.96 sigma)

[bwa_sai2sam_pe_core] time elapses: 35.69 sec

[bwa_sai2sam_pe_core] changing coordinates of 6106 alignments.

[bwa_sai2sam_pe_core] align unmapped mate...

[bwa_paired_sw] 9876 out of 10435 Q17 singletons are mated.

[bwa_paired_sw] 855 out of 1515 Q17 discordant pairs are fixed.

[bwa_sai2sam_pe_core] time elapses: 4.61 sec

[bwa_sai2sam_pe_core] refine gapped alignments... 1.09 sec

[bwa_sai2sam_pe_core] print alignments... 2.32 sec

[bwa_sai2sam_pe_core] 5505024 sequences have been processed.

[bwa_sai2sam_pe_core] convert to sequence coordinate...

[infer_isize] (25, 50, 75) percentile: (453, 463, 473)

[infer_isize] low and high boundaries: 413 and 513 for estimating avg and std

[infer_isize] inferred external isize from 205600 pairs: 463.224 +/- 13.935

[infer_isize] skewness: 0.009; kurtosis: -0.148; ap_prior: 1.77e-05

[infer_isize] inferred maximum insert size: 560 (6.96 sigma)

[bwa_sai2sam_pe_core] time elapses: 42.56 sec

[bwa_sai2sam_pe_core] changing coordinates of 5955 alignments.

[bwa_sai2sam_pe_core] align unmapped mate...

[bwa_paired_sw] 9892 out of 10462 Q17 singletons are mated.

[bwa_paired_sw] 891 out of 1491 Q17 discordant pairs are fixed.

[bwa_sai2sam_pe_core] time elapses: 4.56 sec

[bwa_sai2sam_pe_core] refine gapped alignments... 1.07 sec

[bwa_sai2sam_pe_core] print alignments... 2.29 sec

[bwa_sai2sam_pe_core] 5767168 sequences have been processed.

[bwa_sai2sam_pe_core] convert to sequence coordinate...

[infer_isize] (25, 50, 75) percentile: (453, 463, 473)

[infer_isize] low and high boundaries: 413 and 513 for estimating avg and std

[infer_isize] inferred external isize from 205625 pairs: 463.233 +/- 13.931

[infer_isize] skewness: 0.002; kurtosis: -0.135; ap_prior: 1.67e-05

[infer_isize] inferred maximum insert size: 560 (6.96 sigma)

[bwa_sai2sam_pe_core] time elapses: 32.16 sec

[bwa_sai2sam_pe_core] changing coordinates of 6022 alignments.

[bwa_sai2sam_pe_core] align unmapped mate...

[bwa_paired_sw] 10007 out of 10569 Q17 singletons are mated.

[bwa_paired_sw] 830 out of 1438 Q17 discordant pairs are fixed.

[bwa_sai2sam_pe_core] time elapses: 4.56 sec

[bwa_sai2sam_pe_core] refine gapped alignments... 1.06 sec

[bwa_sai2sam_pe_core] print alignments... 2.14 sec

[bwa_sai2sam_pe_core] 6029312 sequences have been processed.

[bwa_sai2sam_pe_core] convert to sequence coordinate...

[infer_isize] (25, 50, 75) percentile: (453, 463, 473)

[infer_isize] low and high boundaries: 413 and 513 for estimating avg and std

[infer_isize] inferred external isize from 205761 pairs: 463.233 +/- 13.941

[infer_isize] skewness: 0.006; kurtosis: -0.150; ap_prior: 1.82e-05

[infer_isize] inferred maximum insert size: 560 (6.96 sigma)

[bwa_sai2sam_pe_core] time elapses: 31.71 sec

[bwa_sai2sam_pe_core] changing coordinates of 6058 alignments.

[bwa_sai2sam_pe_core] align unmapped mate...

[bwa_paired_sw] 9890 out of 10445 Q17 singletons are mated.

[bwa_paired_sw] 904 out of 1505 Q17 discordant pairs are fixed.

[bwa_sai2sam_pe_core] time elapses: 4.57 sec

[bwa_sai2sam_pe_core] refine gapped alignments... 1.04 sec

[bwa_sai2sam_pe_core] print alignments... 2.29 sec

[bwa_sai2sam_pe_core] 6291456 sequences have been processed.

[bwa_sai2sam_pe_core] convert to sequence coordinate...

[infer_isize] (25, 50, 75) percentile: (453, 463, 473)

[infer_isize] low and high boundaries: 413 and 513 for estimating avg and std

[infer_isize] inferred external isize from 205693 pairs: 463.199 +/- 13.941

[infer_isize] skewness: 0.000; kurtosis: -0.107; ap_prior: 1.77e-05

[infer_isize] inferred maximum insert size: 560 (6.96 sigma)

[bwa_sai2sam_pe_core] time elapses: 37.01 sec

[bwa_sai2sam_pe_core] changing coordinates of 6000 alignments.

[bwa_sai2sam_pe_core] align unmapped mate...

[bwa_paired_sw] 9770 out of 10355 Q17 singletons are mated.

[bwa_paired_sw] 900 out of 1535 Q17 discordant pairs are fixed.

[bwa_sai2sam_pe_core] time elapses: 4.47 sec

[bwa_sai2sam_pe_core] refine gapped alignments... 1.05 sec

[bwa_sai2sam_pe_core] print alignments... 2.11 sec

[bwa_sai2sam_pe_core] 6553600 sequences have been processed.

[bwa_sai2sam_pe_core] convert to sequence coordinate...

[infer_isize] (25, 50, 75) percentile: (453, 463, 473)

[infer_isize] low and high boundaries: 413 and 513 for estimating avg and std

[infer_isize] inferred external isize from 206042 pairs: 463.272 +/- 13.943

[infer_isize] skewness: 0.010; kurtosis: -0.136; ap_prior: 1.77e-05

[infer_isize] inferred maximum insert size: 560 (6.96 sigma)

[bwa_sai2sam_pe_core] time elapses: 31.46 sec

[bwa_sai2sam_pe_core] changing coordinates of 5957 alignments.

[bwa_sai2sam_pe_core] align unmapped mate...

[bwa_paired_sw] 9622 out of 10202 Q17 singletons are mated.

[bwa_paired_sw] 895 out of 1503 Q17 discordant pairs are fixed.

[bwa_sai2sam_pe_core] time elapses: 4.46 sec

[bwa_sai2sam_pe_core] refine gapped alignments... 1.02 sec

[bwa_sai2sam_pe_core] print alignments... 2.18 sec

[bwa_sai2sam_pe_core] 6815744 sequences have been processed.

[bwa_sai2sam_pe_core] convert to sequence coordinate...

[infer_isize] (25, 50, 75) percentile: (453, 463, 473)

[infer_isize] low and high boundaries: 413 and 513 for estimating avg and std

[infer_isize] inferred external isize from 205956 pairs: 463.260 +/- 13.929

[infer_isize] skewness: 0.002; kurtosis: -0.149; ap_prior: 1.90e-05

[infer_isize] inferred maximum insert size: 560 (6.96 sigma)

[bwa_sai2sam_pe_core] time elapses: 31.49 sec

[bwa_sai2sam_pe_core] changing coordinates of 6049 alignments.

[bwa_sai2sam_pe_core] align unmapped mate...

[bwa_paired_sw] 9671 out of 10219 Q17 singletons are mated.

[bwa_paired_sw] 899 out of 1535 Q17 discordant pairs are fixed.

[bwa_sai2sam_pe_core] time elapses: 4.52 sec

[bwa_sai2sam_pe_core] refine gapped alignments... 0.99 sec

[bwa_sai2sam_pe_core] print alignments... 2.13 sec

[bwa_sai2sam_pe_core] 7077888 sequences have been processed.

[bwa_sai2sam_pe_core] convert to sequence coordinate...

[infer_isize] (25, 50, 75) percentile: (453, 463, 473)

[infer_isize] low and high boundaries: 413 and 513 for estimating avg and std

[infer_isize] inferred external isize from 205459 pairs: 463.232 +/- 13.956

[infer_isize] skewness: 0.008; kurtosis: -0.141; ap_prior: 1.81e-05

[infer_isize] inferred maximum insert size: 560 (6.96 sigma)

[bwa_sai2sam_pe_core] time elapses: 31.71 sec

[bwa_sai2sam_pe_core] changing coordinates of 6001 alignments.

[bwa_sai2sam_pe_core] align unmapped mate...

[bwa_paired_sw] 9696 out of 10288 Q17 singletons are mated.

[bwa_paired_sw] 940 out of 1556 Q17 discordant pairs are fixed.

[bwa_sai2sam_pe_core] time elapses: 4.54 sec

[bwa_sai2sam_pe_core] refine gapped alignments... 0.96 sec

[bwa_sai2sam_pe_core] print alignments... 2.06 sec

[bwa_sai2sam_pe_core] 7340032 sequences have been processed.

[bwa_sai2sam_pe_core] convert to sequence coordinate...

[infer_isize] (25, 50, 75) percentile: (453, 463, 473)

[infer_isize] low and high boundaries: 413 and 513 for estimating avg and std

[infer_isize] inferred external isize from 205626 pairs: 463.287 +/- 13.919

[infer_isize] skewness: 0.005; kurtosis: -0.140; ap_prior: 1.75e-05

[infer_isize] inferred maximum insert size: 560 (6.96 sigma)

[bwa_sai2sam_pe_core] time elapses: 32.68 sec

[bwa_sai2sam_pe_core] changing coordinates of 6066 alignments.

[bwa_sai2sam_pe_core] align unmapped mate...

[bwa_paired_sw] 9842 out of 10363 Q17 singletons are mated.

[bwa_paired_sw] 891 out of 1479 Q17 discordant pairs are fixed.

[bwa_sai2sam_pe_core] time elapses: 4.54 sec

[bwa_sai2sam_pe_core] refine gapped alignments... 0.99 sec

[bwa_sai2sam_pe_core] print alignments... 2.09 sec

[bwa_sai2sam_pe_core] 7602176 sequences have been processed.

[bwa_sai2sam_pe_core] convert to sequence coordinate...

[infer_isize] (25, 50, 75) percentile: (453, 463, 473)

[infer_isize] low and high boundaries: 413 and 513 for estimating avg and std

[infer_isize] inferred external isize from 205998 pairs: 463.211 +/- 13.927

[infer_isize] skewness: -0.001; kurtosis: -0.139; ap_prior: 1.70e-05

[infer_isize] inferred maximum insert size: 560 (6.96 sigma)

[bwa_sai2sam_pe_core] time elapses: 31.74 sec

[bwa_sai2sam_pe_core] changing coordinates of 5939 alignments.

[bwa_sai2sam_pe_core] align unmapped mate...

[bwa_paired_sw] 9494 out of 10077 Q17 singletons are mated.

[bwa_paired_sw] 935 out of 1522 Q17 discordant pairs are fixed.

[bwa_sai2sam_pe_core] time elapses: 4.50 sec

[bwa_sai2sam_pe_core] refine gapped alignments... 0.98 sec

[bwa_sai2sam_pe_core] print alignments... 2.17 sec

[bwa_sai2sam_pe_core] 7864320 sequences have been processed.

[bwa_sai2sam_pe_core] convert to sequence coordinate...

[infer_isize] (25, 50, 75) percentile: (453, 463, 473)

[infer_isize] low and high boundaries: 413 and 513 for estimating avg and std

[infer_isize] inferred external isize from 205730 pairs: 463.276 +/- 13.917

[infer_isize] skewness: 0.007; kurtosis: -0.128; ap_prior: 1.67e-05

[infer_isize] inferred maximum insert size: 560 (6.96 sigma)

[bwa_sai2sam_pe_core] time elapses: 33.08 sec

[bwa_sai2sam_pe_core] changing coordinates of 5840 alignments.

[bwa_sai2sam_pe_core] align unmapped mate...

[bwa_paired_sw] 9893 out of 10455 Q17 singletons are mated.

[bwa_paired_sw] 835 out of 1415 Q17 discordant pairs are fixed.

[bwa_sai2sam_pe_core] time elapses: 4.52 sec

[bwa_sai2sam_pe_core] refine gapped alignments... 0.98 sec

[bwa_sai2sam_pe_core] print alignments... 2.30 sec

[bwa_sai2sam_pe_core] 8126464 sequences have been processed.

[bwa_sai2sam_pe_core] convert to sequence coordinate...

[infer_isize] (25, 50, 75) percentile: (453, 463, 473)

[infer_isize] low and high boundaries: 413 and 513 for estimating avg and std

[infer_isize] inferred external isize from 205750 pairs: 463.237 +/- 13.961

[infer_isize] skewness: 0.002; kurtosis: -0.126; ap_prior: 1.75e-05

[infer_isize] inferred maximum insert size: 560 (6.96 sigma)

[bwa_sai2sam_pe_core] time elapses: 34.06 sec

[bwa_sai2sam_pe_core] changing coordinates of 6019 alignments.

[bwa_sai2sam_pe_core] align unmapped mate...

[bwa_paired_sw] 9602 out of 10151 Q17 singletons are mated.

[bwa_paired_sw] 863 out of 1461 Q17 discordant pairs are fixed.

[bwa_sai2sam_pe_core] time elapses: 4.50 sec

[bwa_sai2sam_pe_core] refine gapped alignments... 1.00 sec

[bwa_sai2sam_pe_core] print alignments... 2.20 sec

[bwa_sai2sam_pe_core] 8388608 sequences have been processed.

[bwa_sai2sam_pe_core] convert to sequence coordinate...

[infer_isize] (25, 50, 75) percentile: (453, 463, 473)

[infer_isize] low and high boundaries: 413 and 513 for estimating avg and std

[infer_isize] inferred external isize from 204899 pairs: 463.198 +/- 13.933

[infer_isize] skewness: 0.002; kurtosis: -0.125; ap_prior: 1.77e-05

[infer_isize] inferred maximum insert size: 560 (6.96 sigma)

[bwa_sai2sam_pe_core] time elapses: 33.93 sec

[bwa_sai2sam_pe_core] changing coordinates of 6152 alignments.

[bwa_sai2sam_pe_core] align unmapped mate...

[bwa_paired_sw] 10264 out of 10787 Q17 singletons are mated.

[bwa_paired_sw] 900 out of 1495 Q17 discordant pairs are fixed.

[bwa_sai2sam_pe_core] time elapses: 4.69 sec

[bwa_sai2sam_pe_core] refine gapped alignments... 1.04 sec

[bwa_sai2sam_pe_core] print alignments... 2.30 sec

[bwa_sai2sam_pe_core] 8650752 sequences have been processed.

[bwa_sai2sam_pe_core] convert to sequence coordinate...

[infer_isize] (25, 50, 75) percentile: (453, 463, 473)

[infer_isize] low and high boundaries: 413 and 513 for estimating avg and std

[infer_isize] inferred external isize from 205694 pairs: 463.254 +/- 13.967

[infer_isize] skewness: 0.009; kurtosis: -0.141; ap_prior: 1.77e-05

[infer_isize] inferred maximum insert size: 560 (6.96 sigma)

[bwa_sai2sam_pe_core] time elapses: 36.13 sec

[bwa_sai2sam_pe_core] changing coordinates of 6067 alignments.

[bwa_sai2sam_pe_core] align unmapped mate...

[bwa_paired_sw] 9757 out of 10309 Q17 singletons are mated.

[bwa_paired_sw] 899 out of 1501 Q17 discordant pairs are fixed.

[bwa_sai2sam_pe_core] time elapses: 4.64 sec

[bwa_sai2sam_pe_core] refine gapped alignments... 1.07 sec

[bwa_sai2sam_pe_core] print alignments... 2.30 sec

[bwa_sai2sam_pe_core] 8912896 sequences have been processed.

[bwa_sai2sam_pe_core] convert to sequence coordinate...

[infer_isize] (25, 50, 75) percentile: (453, 463, 473)

[infer_isize] low and high boundaries: 413 and 513 for estimating avg and std

[infer_isize] inferred external isize from 205937 pairs: 463.164 +/- 13.925

[infer_isize] skewness: -0.002; kurtosis: -0.127; ap_prior: 1.84e-05

[infer_isize] inferred maximum insert size: 560 (6.96 sigma)

[bwa_sai2sam_pe_core] time elapses: 36.29 sec

[bwa_sai2sam_pe_core] changing coordinates of 5956 alignments.

[bwa_sai2sam_pe_core] align unmapped mate...

[bwa_paired_sw] 9638 out of 10194 Q17 singletons are mated.

[bwa_paired_sw] 853 out of 1466 Q17 discordant pairs are fixed.

[bwa_sai2sam_pe_core] time elapses: 4.61 sec

[bwa_sai2sam_pe_core] refine gapped alignments... 1.11 sec

[bwa_sai2sam_pe_core] print alignments... 2.29 sec

[bwa_sai2sam_pe_core] 9175040 sequences have been processed.

[bwa_sai2sam_pe_core] convert to sequence coordinate...

[infer_isize] (25, 50, 75) percentile: (453, 463, 473)

[infer_isize] low and high boundaries: 413 and 513 for estimating avg and std

[infer_isize] inferred external isize from 205286 pairs: 463.241 +/- 13.952

[infer_isize] skewness: -0.001; kurtosis: -0.136; ap_prior: 1.70e-05

[infer_isize] inferred maximum insert size: 560 (6.96 sigma)

[bwa_sai2sam_pe_core] time elapses: 55.23 sec

[bwa_sai2sam_pe_core] changing coordinates of 6074 alignments.

[bwa_sai2sam_pe_core] align unmapped mate...

[bwa_paired_sw] 9933 out of 10513 Q17 singletons are mated.

[bwa_paired_sw] 919 out of 1534 Q17 discordant pairs are fixed.

[bwa_sai2sam_pe_core] time elapses: 4.72 sec

[bwa_sai2sam_pe_core] refine gapped alignments... 1.10 sec

[bwa_sai2sam_pe_core] print alignments... 2.25 sec

[bwa_sai2sam_pe_core] 9437184 sequences have been processed.

[bwa_sai2sam_pe_core] convert to sequence coordinate...

[infer_isize] (25, 50, 75) percentile: (453, 463, 473)

[infer_isize] low and high boundaries: 413 and 513 for estimating avg and std

[infer_isize] inferred external isize from 205470 pairs: 463.239 +/- 13.962

[infer_isize] skewness: -0.007; kurtosis: -0.124; ap_prior: 1.76e-05

[infer_isize] inferred maximum insert size: 560 (6.96 sigma)

[bwa_sai2sam_pe_core] time elapses: 33.30 sec

[bwa_sai2sam_pe_core] changing coordinates of 6105 alignments.

[bwa_sai2sam_pe_core] align unmapped mate...

[bwa_paired_sw] 10057 out of 10550 Q17 singletons are mated.

[bwa_paired_sw] 905 out of 1517 Q17 discordant pairs are fixed.

[bwa_sai2sam_pe_core] time elapses: 4.61 sec

[bwa_sai2sam_pe_core] refine gapped alignments... 1.03 sec

[bwa_sai2sam_pe_core] print alignments... 2.13 sec

[bwa_sai2sam_pe_core] 9699328 sequences have been processed.

[bwa_sai2sam_pe_core] convert to sequence coordinate...

[infer_isize] (25, 50, 75) percentile: (453, 463, 473)

[infer_isize] low and high boundaries: 413 and 513 for estimating avg and std

[infer_isize] inferred external isize from 205662 pairs: 463.264 +/- 13.936

[infer_isize] skewness: 0.004; kurtosis: -0.145; ap_prior: 1.67e-05

[infer_isize] inferred maximum insert size: 560 (6.96 sigma)

[bwa_sai2sam_pe_core] time elapses: 33.51 sec

[bwa_sai2sam_pe_core] changing coordinates of 6067 alignments.

[bwa_sai2sam_pe_core] align unmapped mate...

[bwa_paired_sw] 9662 out of 10199 Q17 singletons are mated.

[bwa_paired_sw] 901 out of 1492 Q17 discordant pairs are fixed.

[bwa_sai2sam_pe_core] time elapses: 4.45 sec

[bwa_sai2sam_pe_core] refine gapped alignments... 1.01 sec

[bwa_sai2sam_pe_core] print alignments... 2.15 sec

[bwa_sai2sam_pe_core] 9961472 sequences have been processed.

[bwa_sai2sam_pe_core] convert to sequence coordinate...

[infer_isize] (25, 50, 75) percentile: (453, 463, 473)

[infer_isize] low and high boundaries: 413 and 513 for estimating avg and std

[infer_isize] inferred external isize from 205283 pairs: 463.239 +/- 13.923

[infer_isize] skewness: -0.000; kurtosis: -0.142; ap_prior: 1.65e-05

[infer_isize] inferred maximum insert size: 560 (6.96 sigma)

[bwa_sai2sam_pe_core] time elapses: 32.76 sec

[bwa_sai2sam_pe_core] changing coordinates of 6033 alignments.

[bwa_sai2sam_pe_core] align unmapped mate...

[bwa_paired_sw] 9724 out of 10255 Q17 singletons are mated.

[bwa_paired_sw] 862 out of 1473 Q17 discordant pairs are fixed.

[bwa_sai2sam_pe_core] time elapses: 4.45 sec

[bwa_sai2sam_pe_core] refine gapped alignments... 0.96 sec

[bwa_sai2sam_pe_core] print alignments... 2.05 sec

[bwa_sai2sam_pe_core] 10223616 sequences have been processed.

[bwa_sai2sam_pe_core] convert to sequence coordinate...

[infer_isize] (25, 50, 75) percentile: (453, 463, 473)

[infer_isize] low and high boundaries: 413 and 513 for estimating avg and std

[infer_isize] inferred external isize from 205299 pairs: 463.225 +/- 13.972

[infer_isize] skewness: 0.000; kurtosis: -0.150; ap_prior: 1.88e-05

[infer_isize] inferred maximum insert size: 560 (6.96 sigma)

[bwa_sai2sam_pe_core] time elapses: 34.40 sec

[bwa_sai2sam_pe_core] changing coordinates of 6195 alignments.

[bwa_sai2sam_pe_core] align unmapped mate...

[bwa_paired_sw] 9967 out of 10506 Q17 singletons are mated.

[bwa_paired_sw] 891 out of 1514 Q17 discordant pairs are fixed.

[bwa_sai2sam_pe_core] time elapses: 4.34 sec

[bwa_sai2sam_pe_core] refine gapped alignments... 0.92 sec

[bwa_sai2sam_pe_core] print alignments... 2.06 sec

[bwa_sai2sam_pe_core] 10485760 sequences have been processed.

[bwa_sai2sam_pe_core] convert to sequence coordinate...

[infer_isize] (25, 50, 75) percentile: (453, 463, 473)

[infer_isize] low and high boundaries: 413 and 513 for estimating avg and std

[infer_isize] inferred external isize from 204888 pairs: 463.242 +/- 13.967

[infer_isize] skewness: 0.000; kurtosis: -0.135; ap_prior: 1.92e-05

[infer_isize] inferred maximum insert size: 560 (6.96 sigma)

[bwa_sai2sam_pe_core] time elapses: 29.56 sec

[bwa_sai2sam_pe_core] changing coordinates of 6033 alignments.

[bwa_sai2sam_pe_core] align unmapped mate...

[bwa_paired_sw] 10473 out of 10958 Q17 singletons are mated.

[bwa_paired_sw] 906 out of 1452 Q17 discordant pairs are fixed.

[bwa_sai2sam_pe_core] time elapses: 4.47 sec

[bwa_sai2sam_pe_core] refine gapped alignments... 0.90 sec

[bwa_sai2sam_pe_core] print alignments... 2.05 sec

[bwa_sai2sam_pe_core] 10747904 sequences have been processed.

[bwa_sai2sam_pe_core] convert to sequence coordinate...

[infer_isize] (25, 50, 75) percentile: (453, 463, 473)

[infer_isize] low and high boundaries: 413 and 513 for estimating avg and std

[infer_isize] inferred external isize from 205361 pairs: 463.168 +/- 13.988

[infer_isize] skewness: 0.003; kurtosis: -0.142; ap_prior: 2.00e-05

[infer_isize] inferred maximum insert size: 561 (6.96 sigma)

[bwa_sai2sam_pe_core] time elapses: 29.14 sec

[bwa_sai2sam_pe_core] changing coordinates of 6109 alignments.

[bwa_sai2sam_pe_core] align unmapped mate...

[bwa_paired_sw] 9842 out of 10349 Q17 singletons are mated.

[bwa_paired_sw] 918 out of 1491 Q17 discordant pairs are fixed.

[bwa_sai2sam_pe_core] time elapses: 4.31 sec

[bwa_sai2sam_pe_core] refine gapped alignments... 0.92 sec

[bwa_sai2sam_pe_core] print alignments... 2.05 sec

[bwa_sai2sam_pe_core] 11010048 sequences have been processed.

[bwa_sai2sam_pe_core] convert to sequence coordinate...

[infer_isize] (25, 50, 75) percentile: (453, 463, 473)

[infer_isize] low and high boundaries: 413 and 513 for estimating avg and std

[infer_isize] inferred external isize from 205740 pairs: 463.319 +/- 13.969

[infer_isize] skewness: -0.002; kurtosis: -0.162; ap_prior: 1.95e-05

[infer_isize] inferred maximum insert size: 561 (6.96 sigma)

[bwa_sai2sam_pe_core] time elapses: 29.67 sec

[bwa_sai2sam_pe_core] changing coordinates of 6080 alignments.

[bwa_sai2sam_pe_core] align unmapped mate...

[bwa_paired_sw] 9548 out of 10088 Q17 singletons are mated.

[bwa_paired_sw] 869 out of 1487 Q17 discordant pairs are fixed.

[bwa_sai2sam_pe_core] time elapses: 4.25 sec

[bwa_sai2sam_pe_core] refine gapped alignments... 0.91 sec

[bwa_sai2sam_pe_core] print alignments... 2.07 sec

[bwa_sai2sam_pe_core] 11272192 sequences have been processed.

[bwa_sai2sam_pe_core] convert to sequence coordinate...

[infer_isize] (25, 50, 75) percentile: (453, 463, 473)

[infer_isize] low and high boundaries: 413 and 513 for estimating avg and std

[infer_isize] inferred external isize from 205336 pairs: 463.247 +/- 13.932

[infer_isize] skewness: -0.003; kurtosis: -0.144; ap_prior: 1.75e-05

[infer_isize] inferred maximum insert size: 560 (6.96 sigma)

[bwa_sai2sam_pe_core] time elapses: 30.83 sec

[bwa_sai2sam_pe_core] changing coordinates of 5973 alignments.

[bwa_sai2sam_pe_core] align unmapped mate...

[bwa_paired_sw] 9865 out of 10363 Q17 singletons are mated.

[bwa_paired_sw] 888 out of 1510 Q17 discordant pairs are fixed.

[bwa_sai2sam_pe_core] time elapses: 4.48 sec

[bwa_sai2sam_pe_core] refine gapped alignments... 0.95 sec

[bwa_sai2sam_pe_core] print alignments... 2.15 sec

[bwa_sai2sam_pe_core] 11534336 sequences have been processed.

[bwa_sai2sam_pe_core] convert to sequence coordinate...

[infer_isize] (25, 50, 75) percentile: (453, 463, 473)

[infer_isize] low and high boundaries: 413 and 513 for estimating avg and std

[infer_isize] inferred external isize from 205157 pairs: 463.239 +/- 13.929

[infer_isize] skewness: -0.001; kurtosis: -0.150; ap_prior: 1.90e-05

[infer_isize] inferred maximum insert size: 560 (6.96 sigma)

[bwa_sai2sam_pe_core] time elapses: 32.33 sec

[bwa_sai2sam_pe_core] changing coordinates of 5994 alignments.

[bwa_sai2sam_pe_core] align unmapped mate...

[bwa_paired_sw] 10005 out of 10537 Q17 singletons are mated.

[bwa_paired_sw] 906 out of 1511 Q17 discordant pairs are fixed.

[bwa_sai2sam_pe_core] time elapses: 4.62 sec

[bwa_sai2sam_pe_core] refine gapped alignments... 1.04 sec

[bwa_sai2sam_pe_core] print alignments... 2.22 sec

[bwa_sai2sam_pe_core] 11796480 sequences have been processed.

[bwa_sai2sam_pe_core] convert to sequence coordinate...

[infer_isize] (25, 50, 75) percentile: (453, 463, 473)

[infer_isize] low and high boundaries: 413 and 513 for estimating avg and std

[infer_isize] inferred external isize from 205739 pairs: 463.210 +/- 13.916

[infer_isize] skewness: 0.008; kurtosis: -0.150; ap_prior: 1.72e-05

[infer_isize] inferred maximum insert size: 560 (6.96 sigma)

[bwa_sai2sam_pe_core] time elapses: 33.03 sec

[bwa_sai2sam_pe_core] changing coordinates of 6039 alignments.

[bwa_sai2sam_pe_core] align unmapped mate...

[bwa_paired_sw] 9553 out of 10095 Q17 singletons are mated.

[bwa_paired_sw] 912 out of 1519 Q17 discordant pairs are fixed.

[bwa_sai2sam_pe_core] time elapses: 4.50 sec

[bwa_sai2sam_pe_core] refine gapped alignments... 1.08 sec

[bwa_sai2sam_pe_core] print alignments... 2.23 sec

[bwa_sai2sam_pe_core] 12058624 sequences have been processed.

[bwa_sai2sam_pe_core] convert to sequence coordinate...

[infer_isize] (25, 50, 75) percentile: (453, 463, 473)

[infer_isize] low and high boundaries: 413 and 513 for estimating avg and std

[infer_isize] inferred external isize from 205263 pairs: 463.178 +/- 13.964

[infer_isize] skewness: -0.000; kurtosis: -0.139; ap_prior: 1.86e-05

[infer_isize] inferred maximum insert size: 560 (6.96 sigma)

[bwa_sai2sam_pe_core] time elapses: 35.50 sec

[bwa_sai2sam_pe_core] changing coordinates of 6149 alignments.

[bwa_sai2sam_pe_core] align unmapped mate...

[bwa_paired_sw] 9801 out of 10346 Q17 singletons are mated.

[bwa_paired_sw] 865 out of 1455 Q17 discordant pairs are fixed.

[bwa_sai2sam_pe_core] time elapses: 4.53 sec

[bwa_sai2sam_pe_core] refine gapped alignments... 1.05 sec

[bwa_sai2sam_pe_core] print alignments... 2.30 sec

[bwa_sai2sam_pe_core] 12320768 sequences have been processed.

[bwa_sai2sam_pe_core] convert to sequence coordinate...

[infer_isize] (25, 50, 75) percentile: (453, 463, 473)

[infer_isize] low and high boundaries: 413 and 513 for estimating avg and std

[infer_isize] inferred external isize from 205087 pairs: 463.253 +/- 13.966

[infer_isize] skewness: 0.004; kurtosis: -0.127; ap_prior: 1.70e-05

[infer_isize] inferred maximum insert size: 560 (6.96 sigma)

[bwa_sai2sam_pe_core] time elapses: 34.10 sec

[bwa_sai2sam_pe_core] changing coordinates of 6048 alignments.

[bwa_sai2sam_pe_core] align unmapped mate...

[bwa_paired_sw] 10150 out of 10689 Q17 singletons are mated.

[bwa_paired_sw] 870 out of 1478 Q17 discordant pairs are fixed.

[bwa_sai2sam_pe_core] time elapses: 4.66 sec

[bwa_sai2sam_pe_core] refine gapped alignments... 1.06 sec

[bwa_sai2sam_pe_core] print alignments... 2.15 sec

[bwa_sai2sam_pe_core] 12582912 sequences have been processed.

[bwa_sai2sam_pe_core] convert to sequence coordinate...

[infer_isize] (25, 50, 75) percentile: (453, 463, 473)

[infer_isize] low and high boundaries: 413 and 513 for estimating avg and std

[infer_isize] inferred external isize from 205267 pairs: 463.225 +/- 13.934

[infer_isize] skewness: 0.005; kurtosis: -0.141; ap_prior: 1.83e-05

[infer_isize] inferred maximum insert size: 560 (6.96 sigma)

[bwa_sai2sam_pe_core] time elapses: 63.52 sec

[bwa_sai2sam_pe_core] changing coordinates of 6083 alignments.

[bwa_sai2sam_pe_core] align unmapped mate...

[bwa_paired_sw] 10277 out of 10885 Q17 singletons are mated.

[bwa_paired_sw] 892 out of 1527 Q17 discordant pairs are fixed.

[bwa_sai2sam_pe_core] time elapses: 4.71 sec

[bwa_sai2sam_pe_core] refine gapped alignments... 1.06 sec

[bwa_sai2sam_pe_core] print alignments... 2.19 sec

[bwa_sai2sam_pe_core] 12845056 sequences have been processed.

[bwa_sai2sam_pe_core] convert to sequence coordinate...

[infer_isize] (25, 50, 75) percentile: (453, 463, 473)

[infer_isize] low and high boundaries: 413 and 513 for estimating avg and std

[infer_isize] inferred external isize from 205012 pairs: 463.244 +/- 13.957

[infer_isize] skewness: -0.002; kurtosis: -0.130; ap_prior: 1.56e-05

[infer_isize] inferred maximum insert size: 560 (6.96 sigma)

[bwa_sai2sam_pe_core] time elapses: 32.59 sec

[bwa_sai2sam_pe_core] changing coordinates of 5984 alignments.

[bwa_sai2sam_pe_core] align unmapped mate...

[bwa_paired_sw] 10032 out of 10606 Q17 singletons are mated.

[bwa_paired_sw] 874 out of 1476 Q17 discordant pairs are fixed.

[bwa_sai2sam_pe_core] time elapses: 4.40 sec

[bwa_sai2sam_pe_core] refine gapped alignments... 1.01 sec

[bwa_sai2sam_pe_core] print alignments... 2.17 sec

[bwa_sai2sam_pe_core] 13107200 sequences have been processed.

[bwa_sai2sam_pe_core] convert to sequence coordinate...

[infer_isize] (25, 50, 75) percentile: (453, 463, 473)

[infer_isize] low and high boundaries: 413 and 513 for estimating avg and std

[infer_isize] inferred external isize from 204842 pairs: 463.186 +/- 13.912

[infer_isize] skewness: 0.002; kurtosis: -0.125; ap_prior: 1.70e-05

[infer_isize] inferred maximum insert size: 560 (6.96 sigma)

[bwa_sai2sam_pe_core] time elapses: 32.65 sec

[bwa_sai2sam_pe_core] changing coordinates of 5987 alignments.

[bwa_sai2sam_pe_core] align unmapped mate...

[bwa_paired_sw] 10145 out of 10690 Q17 singletons are mated.

[bwa_paired_sw] 855 out of 1425 Q17 discordant pairs are fixed.

[bwa_sai2sam_pe_core] time elapses: 4.59 sec

[bwa_sai2sam_pe_core] refine gapped alignments... 0.98 sec

[bwa_sai2sam_pe_core] print alignments... 2.14 sec

[bwa_sai2sam_pe_core] 13369344 sequences have been processed.

[bwa_sai2sam_pe_core] convert to sequence coordinate...

[infer_isize] (25, 50, 75) percentile: (453, 463, 473)

[infer_isize] low and high boundaries: 413 and 513 for estimating avg and std

[infer_isize] inferred external isize from 205317 pairs: 463.227 +/- 13.929

[infer_isize] skewness: -0.003; kurtosis: -0.149; ap_prior: 1.65e-05

[infer_isize] inferred maximum insert size: 560 (6.96 sigma)

[bwa_sai2sam_pe_core] time elapses: 32.80 sec

[bwa_sai2sam_pe_core] changing coordinates of 5929 alignments.

[bwa_sai2sam_pe_core] align unmapped mate...

[bwa_paired_sw] 9850 out of 10405 Q17 singletons are mated.

[bwa_paired_sw] 904 out of 1501 Q17 discordant pairs are fixed.

[bwa_sai2sam_pe_core] time elapses: 4.57 sec

[bwa_sai2sam_pe_core] refine gapped alignments... 0.99 sec

[bwa_sai2sam_pe_core] print alignments... 2.13 sec

[bwa_sai2sam_pe_core] 13631488 sequences have been processed.

[bwa_sai2sam_pe_core] convert to sequence coordinate...

[infer_isize] (25, 50, 75) percentile: (453, 463, 473)

[infer_isize] low and high boundaries: 413 and 513 for estimating avg and std

[infer_isize] inferred external isize from 204870 pairs: 463.221 +/- 13.950

[infer_isize] skewness: 0.005; kurtosis: -0.132; ap_prior: 1.79e-05

[infer_isize] inferred maximum insert size: 560 (6.96 sigma)

[bwa_sai2sam_pe_core] time elapses: 33.17 sec

[bwa_sai2sam_pe_core] changing coordinates of 6065 alignments.

[bwa_sai2sam_pe_core] align unmapped mate...

[bwa_paired_sw] 10266 out of 10813 Q17 singletons are mated.

[bwa_paired_sw] 857 out of 1458 Q17 discordant pairs are fixed.

[bwa_sai2sam_pe_core] time elapses: 4.67 sec

[bwa_sai2sam_pe_core] refine gapped alignments... 0.98 sec

[bwa_sai2sam_pe_core] print alignments... 2.13 sec

[bwa_sai2sam_pe_core] 13893632 sequences have been processed.

[bwa_sai2sam_pe_core] convert to sequence coordinate...

[infer_isize] (25, 50, 75) percentile: (453, 463, 473)

[infer_isize] low and high boundaries: 413 and 513 for estimating avg and std

[infer_isize] inferred external isize from 205419 pairs: 463.213 +/- 13.952

[infer_isize] skewness: -0.006; kurtosis: -0.140; ap_prior: 1.83e-05

[infer_isize] inferred maximum insert size: 560 (6.96 sigma)

[bwa_sai2sam_pe_core] time elapses: 34.17 sec

[bwa_sai2sam_pe_core] changing coordinates of 6174 alignments.

[bwa_sai2sam_pe_core] align unmapped mate...

[bwa_paired_sw] 9987 out of 10564 Q17 singletons are mated.

[bwa_paired_sw] 896 out of 1519 Q17 discordant pairs are fixed.

[bwa_sai2sam_pe_core] time elapses: 4.63 sec

[bwa_sai2sam_pe_core] refine gapped alignments... 0.97 sec

[bwa_sai2sam_pe_core] print alignments... 2.10 sec

[bwa_sai2sam_pe_core] 14155776 sequences have been processed.

[bwa_sai2sam_pe_core] convert to sequence coordinate...

[infer_isize] (25, 50, 75) percentile: (453, 463, 473)

[infer_isize] low and high boundaries: 413 and 513 for estimating avg and std

[infer_isize] inferred external isize from 204636 pairs: 463.174 +/- 13.917

[infer_isize] skewness: 0.000; kurtosis: -0.131; ap_prior: 1.67e-05

[infer_isize] inferred maximum insert size: 560 (6.96 sigma)

[bwa_sai2sam_pe_core] time elapses: 33.73 sec

[bwa_sai2sam_pe_core] changing coordinates of 6146 alignments.

[bwa_sai2sam_pe_core] align unmapped mate...

[bwa_paired_sw] 10267 out of 10832 Q17 singletons are mated.

[bwa_paired_sw] 903 out of 1497 Q17 discordant pairs are fixed.

[bwa_sai2sam_pe_core] time elapses: 4.69 sec

[bwa_sai2sam_pe_core] refine gapped alignments... 0.99 sec

[bwa_sai2sam_pe_core] print alignments... 2.12 sec

[bwa_sai2sam_pe_core] 14417920 sequences have been processed.

[bwa_sai2sam_pe_core] convert to sequence coordinate...

[infer_isize] (25, 50, 75) percentile: (453, 463, 473)

[infer_isize] low and high boundaries: 413 and 513 for estimating avg and std

[infer_isize] inferred external isize from 205281 pairs: 463.150 +/- 13.915

[infer_isize] skewness: 0.003; kurtosis: -0.135; ap_prior: 1.76e-05

[infer_isize] inferred maximum insert size: 560 (6.96 sigma)

[bwa_sai2sam_pe_core] time elapses: 33.53 sec

[bwa_sai2sam_pe_core] changing coordinates of 5905 alignments.

[bwa_sai2sam_pe_core] align unmapped mate...

[bwa_paired_sw] 10062 out of 10617 Q17 singletons are mated.

[bwa_paired_sw] 903 out of 1520 Q17 discordant pairs are fixed.

[bwa_sai2sam_pe_core] time elapses: 4.66 sec

[bwa_sai2sam_pe_core] refine gapped alignments... 0.97 sec

[bwa_sai2sam_pe_core] print alignments... 2.16 sec

[bwa_sai2sam_pe_core] 14680064 sequences have been processed.

[bwa_sai2sam_pe_core] convert to sequence coordinate...

[infer_isize] (25, 50, 75) percentile: (453, 463, 473)

[infer_isize] low and high boundaries: 413 and 513 for estimating avg and std

[infer_isize] inferred external isize from 204901 pairs: 463.183 +/- 13.914

[infer_isize] skewness: -0.004; kurtosis: -0.132; ap_prior: 1.89e-05

[infer_isize] inferred maximum insert size: 560 (6.96 sigma)

[bwa_sai2sam_pe_core] time elapses: 34.38 sec

[bwa_sai2sam_pe_core] changing coordinates of 6062 alignments.

[bwa_sai2sam_pe_core] align unmapped mate...

[bwa_paired_sw] 9932 out of 10466 Q17 singletons are mated.

[bwa_paired_sw] 905 out of 1537 Q17 discordant pairs are fixed.

[bwa_sai2sam_pe_core] time elapses: 4.64 sec

[bwa_sai2sam_pe_core] refine gapped alignments... 1.08 sec

[bwa_sai2sam_pe_core] print alignments... 2.30 sec

[bwa_sai2sam_pe_core] 14942208 sequences have been processed.

[bwa_sai2sam_pe_core] convert to sequence coordinate...

[infer_isize] (25, 50, 75) percentile: (453, 463, 473)

[infer_isize] low and high boundaries: 413 and 513 for estimating avg and std

[infer_isize] inferred external isize from 205104 pairs: 463.155 +/- 13.980

[infer_isize] skewness: 0.005; kurtosis: -0.139; ap_prior: 1.81e-05

[infer_isize] inferred maximum insert size: 560 (6.96 sigma)

[bwa_sai2sam_pe_core] time elapses: 33.95 sec

[bwa_sai2sam_pe_core] changing coordinates of 5919 alignments.

[bwa_sai2sam_pe_core] align unmapped mate...

[bwa_paired_sw] 10329 out of 10858 Q17 singletons are mated.

[bwa_paired_sw] 866 out of 1478 Q17 discordant pairs are fixed.

[bwa_sai2sam_pe_core] time elapses: 4.76 sec

[bwa_sai2sam_pe_core] refine gapped alignments... 1.07 sec

[bwa_sai2sam_pe_core] print alignments... 2.31 sec

[bwa_sai2sam_pe_core] 15204352 sequences have been processed.

[bwa_sai2sam_pe_core] convert to sequence coordinate...

[infer_isize] (25, 50, 75) percentile: (453, 463, 473)

[infer_isize] low and high boundaries: 413 and 513 for estimating avg and std

[infer_isize] inferred external isize from 205001 pairs: 463.187 +/- 13.948

[infer_isize] skewness: -0.003; kurtosis: -0.143; ap_prior: 1.68e-05

[infer_isize] inferred maximum insert size: 560 (6.96 sigma)

[bwa_sai2sam_pe_core] time elapses: 36.56 sec

[bwa_sai2sam_pe_core] changing coordinates of 6123 alignments.

[bwa_sai2sam_pe_core] align unmapped mate...

[bwa_paired_sw] 10066 out of 10638 Q17 singletons are mated.

[bwa_paired_sw] 870 out of 1479 Q17 discordant pairs are fixed.

[bwa_sai2sam_pe_core] time elapses: 4.70 sec

[bwa_sai2sam_pe_core] refine gapped alignments... 1.11 sec

[bwa_sai2sam_pe_core] print alignments... 2.32 sec

[bwa_sai2sam_pe_core] 15466496 sequences have been processed.

[bwa_sai2sam_pe_core] convert to sequence coordinate...

[infer_isize] (25, 50, 75) percentile: (453, 463, 473)

[infer_isize] low and high boundaries: 413 and 513 for estimating avg and std

[infer_isize] inferred external isize from 204945 pairs: 463.158 +/- 13.940

[infer_isize] skewness: 0.002; kurtosis: -0.131; ap_prior: 1.95e-05

[infer_isize] inferred maximum insert size: 560 (6.96 sigma)

[bwa_sai2sam_pe_core] time elapses: 36.48 sec

[bwa_sai2sam_pe_core] changing coordinates of 6075 alignments.

[bwa_sai2sam_pe_core] align unmapped mate...

[bwa_paired_sw] 10246 out of 10731 Q17 singletons are mated.

[bwa_paired_sw] 924 out of 1492 Q17 discordant pairs are fixed.

[bwa_sai2sam_pe_core] time elapses: 4.83 sec

[bwa_sai2sam_pe_core] refine gapped alignments... 1.13 sec

[bwa_sai2sam_pe_core] print alignments... 2.37 sec

[bwa_sai2sam_pe_core] 15728640 sequences have been processed.

[bwa_sai2sam_pe_core] convert to sequence coordinate...

[infer_isize] (25, 50, 75) percentile: (453, 463, 473)

[infer_isize] low and high boundaries: 413 and 513 for estimating avg and std

[infer_isize] inferred external isize from 204731 pairs: 463.168 +/- 13.941

[infer_isize] skewness: -0.001; kurtosis: -0.154; ap_prior: 1.69e-05

[infer_isize] inferred maximum insert size: 560 (6.96 sigma)

[bwa_sai2sam_pe_core] time elapses: 36.92 sec

[bwa_sai2sam_pe_core] changing coordinates of 6102 alignments.

[bwa_sai2sam_pe_core] align unmapped mate...

[bwa_paired_sw] 10362 out of 10903 Q17 singletons are mated.

[bwa_paired_sw] 887 out of 1501 Q17 discordant pairs are fixed.

[bwa_sai2sam_pe_core] time elapses: 4.84 sec

[bwa_sai2sam_pe_core] refine gapped alignments... 1.14 sec

[bwa_sai2sam_pe_core] print alignments... 2.30 sec

[bwa_sai2sam_pe_core] 15990784 sequences have been processed.

[bwa_sai2sam_pe_core] convert to sequence coordinate...

[infer_isize] (25, 50, 75) percentile: (453, 463, 473)

[infer_isize] low and high boundaries: 413 and 513 for estimating avg and std

[infer_isize] inferred external isize from 205123 pairs: 463.160 +/- 13.911

[infer_isize] skewness: -0.003; kurtosis: -0.127; ap_prior: 1.71e-05

[infer_isize] inferred maximum insert size: 560 (6.96 sigma)

[bwa_sai2sam_pe_core] time elapses: 36.42 sec

[bwa_sai2sam_pe_core] changing coordinates of 5926 alignments.

[bwa_sai2sam_pe_core] align unmapped mate...

[bwa_paired_sw] 10347 out of 10837 Q17 singletons are mated.

[bwa_paired_sw] 865 out of 1482 Q17 discordant pairs are fixed.

[bwa_sai2sam_pe_core] time elapses: 4.68 sec

[bwa_sai2sam_pe_core] refine gapped alignments... 1.11 sec

[bwa_sai2sam_pe_core] print alignments... 2.26 sec

[bwa_sai2sam_pe_core] 16252928 sequences have been processed.

[bwa_sai2sam_pe_core] convert to sequence coordinate...

[infer_isize] (25, 50, 75) percentile: (453, 463, 473)

[infer_isize] low and high boundaries: 413 and 513 for estimating avg and std

[infer_isize] inferred external isize from 204876 pairs: 463.187 +/- 13.955

[infer_isize] skewness: 0.006; kurtosis: -0.146; ap_prior: 1.78e-05

[infer_isize] inferred maximum insert size: 560 (6.96 sigma)

[bwa_sai2sam_pe_core] time elapses: 31.31 sec

[bwa_sai2sam_pe_core] changing coordinates of 5834 alignments.

[bwa_sai2sam_pe_core] align unmapped mate...

[bwa_paired_sw] 10360 out of 10879 Q17 singletons are mated.

[bwa_paired_sw] 885 out of 1491 Q17 discordant pairs are fixed.

[bwa_sai2sam_pe_core] time elapses: 4.46 sec

[bwa_sai2sam_pe_core] refine gapped alignments... 0.94 sec

[bwa_sai2sam_pe_core] print alignments... 2.08 sec

[bwa_sai2sam_pe_core] 16515072 sequences have been processed.

[bwa_sai2sam_pe_core] convert to sequence coordinate...

[infer_isize] (25, 50, 75) percentile: (453, 463, 473)

[infer_isize] low and high boundaries: 413 and 513 for estimating avg and std

[infer_isize] inferred external isize from 204959 pairs: 463.103 +/- 13.943

[infer_isize] skewness: 0.009; kurtosis: -0.143; ap_prior: 1.79e-05

[infer_isize] inferred maximum insert size: 560 (6.96 sigma)

[bwa_sai2sam_pe_core] time elapses: 30.26 sec

[bwa_sai2sam_pe_core] changing coordinates of 6032 alignments.

[bwa_sai2sam_pe_core] align unmapped mate...

[bwa_paired_sw] 10319 out of 10867 Q17 singletons are mated.

[bwa_paired_sw] 892 out of 1500 Q17 discordant pairs are fixed.

[bwa_sai2sam_pe_core] time elapses: 4.40 sec

[bwa_sai2sam_pe_core] refine gapped alignments... 0.89 sec

[bwa_sai2sam_pe_core] print alignments... 2.09 sec

[bwa_sai2sam_pe_core] 16777216 sequences have been processed.

[bwa_sai2sam_pe_core] convert to sequence coordinate...

[infer_isize] (25, 50, 75) percentile: (453, 463, 473)

[infer_isize] low and high boundaries: 413 and 513 for estimating avg and std

[infer_isize] inferred external isize from 204307 pairs: 463.125 +/- 13.935

[infer_isize] skewness: 0.005; kurtosis: -0.148; ap_prior: 1.69e-05

[infer_isize] inferred maximum insert size: 560 (6.96 sigma)

[bwa_sai2sam_pe_core] time elapses: 30.40 sec

[bwa_sai2sam_pe_core] changing coordinates of 6228 alignments.

[bwa_sai2sam_pe_core] align unmapped mate...

[bwa_paired_sw] 10372 out of 10929 Q17 singletons are mated.

[bwa_paired_sw] 877 out of 1462 Q17 discordant pairs are fixed.

[bwa_sai2sam_pe_core] time elapses: 4.40 sec

[bwa_sai2sam_pe_core] refine gapped alignments... 0.91 sec

[bwa_sai2sam_pe_core] print alignments... 2.09 sec

[bwa_sai2sam_pe_core] 17039360 sequences have been processed.

[bwa_sai2sam_pe_core] convert to sequence coordinate...

[infer_isize] (25, 50, 75) percentile: (453, 463, 473)

[infer_isize] low and high boundaries: 413 and 513 for estimating avg and std

[infer_isize] inferred external isize from 204077 pairs: 463.133 +/- 13.941

[infer_isize] skewness: 0.003; kurtosis: -0.129; ap_prior: 1.81e-05

[infer_isize] inferred maximum insert size: 560 (6.96 sigma)

[bwa_sai2sam_pe_core] time elapses: 25.60 sec

[bwa_sai2sam_pe_core] changing coordinates of 6059 alignments.

[bwa_sai2sam_pe_core] align unmapped mate...

[bwa_paired_sw] 10755 out of 11323 Q17 singletons are mated.

[bwa_paired_sw] 870 out of 1483 Q17 discordant pairs are fixed.

[bwa_sai2sam_pe_core] time elapses: 4.53 sec

[bwa_sai2sam_pe_core] refine gapped alignments... 0.91 sec

[bwa_sai2sam_pe_core] print alignments... 2.09 sec

[bwa_sai2sam_pe_core] 17301504 sequences have been processed.

[bwa_sai2sam_pe_core] convert to sequence coordinate...

[infer_isize] (25, 50, 75) percentile: (453, 463, 473)

[infer_isize] low and high boundaries: 413 and 513 for estimating avg and std

[infer_isize] inferred external isize from 203608 pairs: 463.158 +/- 13.920

[infer_isize] skewness: 0.008; kurtosis: -0.135; ap_prior: 1.82e-05

[infer_isize] inferred maximum insert size: 560 (6.96 sigma)

[bwa_sai2sam_pe_core] time elapses: 25.23 sec

[bwa_sai2sam_pe_core] changing coordinates of 6016 alignments.

[bwa_sai2sam_pe_core] align unmapped mate...

[bwa_paired_sw] 11344 out of 11846 Q17 singletons are mated.

[bwa_paired_sw] 846 out of 1454 Q17 discordant pairs are fixed.

[bwa_sai2sam_pe_core] time elapses: 4.66 sec

[bwa_sai2sam_pe_core] refine gapped alignments... 0.92 sec

[bwa_sai2sam_pe_core] print alignments... 2.08 sec

[bwa_sai2sam_pe_core] 17563648 sequences have been processed.

[bwa_sai2sam_pe_core] convert to sequence coordinate...

[infer_isize] (25, 50, 75) percentile: (453, 463, 473)

[infer_isize] low and high boundaries: 413 and 513 for estimating avg and std

[infer_isize] inferred external isize from 204173 pairs: 463.104 +/- 13.961

[infer_isize] skewness: -0.004; kurtosis: -0.140; ap_prior: 1.68e-05

[infer_isize] inferred maximum insert size: 560 (6.96 sigma)

[bwa_sai2sam_pe_core] time elapses: 25.24 sec

[bwa_sai2sam_pe_core] changing coordinates of 5977 alignments.

[bwa_sai2sam_pe_core] align unmapped mate...

[bwa_paired_sw] 10932 out of 11449 Q17 singletons are mated.

[bwa_paired_sw] 904 out of 1485 Q17 discordant pairs are fixed.

[bwa_sai2sam_pe_core] time elapses: 4.56 sec

[bwa_sai2sam_pe_core] refine gapped alignments... 0.92 sec

[bwa_sai2sam_pe_core] print alignments... 2.07 sec

[bwa_sai2sam_pe_core] 17825792 sequences have been processed.

[bwa_sai2sam_pe_core] convert to sequence coordinate...

[infer_isize] (25, 50, 75) percentile: (453, 463, 473)

[infer_isize] low and high boundaries: 413 and 513 for estimating avg and std

[infer_isize] inferred external isize from 204122 pairs: 463.139 +/- 13.949

[infer_isize] skewness: 0.005; kurtosis: -0.124; ap_prior: 1.89e-05

[infer_isize] inferred maximum insert size: 560 (6.96 sigma)

[bwa_sai2sam_pe_core] time elapses: 26.50 sec

[bwa_sai2sam_pe_core] changing coordinates of 6029 alignments.

[bwa_sai2sam_pe_core] align unmapped mate...

[bwa_paired_sw] 10885 out of 11463 Q17 singletons are mated.

[bwa_paired_sw] 885 out of 1464 Q17 discordant pairs are fixed.

[bwa_sai2sam_pe_core] time elapses: 4.54 sec

[bwa_sai2sam_pe_core] refine gapped alignments... 0.91 sec

[bwa_sai2sam_pe_core] print alignments... 2.10 sec

[bwa_sai2sam_pe_core] 18087936 sequences have been processed.

[bwa_sai2sam_pe_core] convert to sequence coordinate...

[infer_isize] (25, 50, 75) percentile: (453, 463, 473)

[infer_isize] low and high boundaries: 413 and 513 for estimating avg and std

[infer_isize] inferred external isize from 204428 pairs: 463.168 +/- 13.946

[infer_isize] skewness: -0.005; kurtosis: -0.131; ap_prior: 1.84e-05

[infer_isize] inferred maximum insert size: 560 (6.96 sigma)

[bwa_sai2sam_pe_core] time elapses: 33.36 sec

[bwa_sai2sam_pe_core] changing coordinates of 5907 alignments.

[bwa_sai2sam_pe_core] align unmapped mate...

[bwa_paired_sw] 10807 out of 11362 Q17 singletons are mated.

[bwa_paired_sw] 871 out of 1493 Q17 discordant pairs are fixed.

[bwa_sai2sam_pe_core] time elapses: 4.85 sec

[bwa_sai2sam_pe_core] refine gapped alignments... 0.99 sec

[bwa_sai2sam_pe_core] print alignments... 2.17 sec

[bwa_sai2sam_pe_core] 18350080 sequences have been processed.

[bwa_sai2sam_pe_core] convert to sequence coordinate...

[infer_isize] (25, 50, 75) percentile: (453, 463, 473)

[infer_isize] low and high boundaries: 413 and 513 for estimating avg and std

[infer_isize] inferred external isize from 204099 pairs: 463.155 +/- 13.927

[infer_isize] skewness: 0.000; kurtosis: -0.127; ap_prior: 1.96e-05

[infer_isize] inferred maximum insert size: 560 (6.96 sigma)

[bwa_sai2sam_pe_core] time elapses: 34.05 sec

[bwa_sai2sam_pe_core] changing coordinates of 6034 alignments.

[bwa_sai2sam_pe_core] align unmapped mate...

[bwa_paired_sw] 10810 out of 11393 Q17 singletons are mated.

[bwa_paired_sw] 895 out of 1480 Q17 discordant pairs are fixed.

[bwa_sai2sam_pe_core] time elapses: 4.84 sec

[bwa_sai2sam_pe_core] refine gapped alignments... 1.07 sec

[bwa_sai2sam_pe_core] print alignments... 2.26 sec

[bwa_sai2sam_pe_core] 18612224 sequences have been processed.

[bwa_sai2sam_pe_core] convert to sequence coordinate...

[infer_isize] (25, 50, 75) percentile: (453, 463, 473)

[infer_isize] low and high boundaries: 413 and 513 for estimating avg and std

[infer_isize] inferred external isize from 204043 pairs: 463.104 +/- 13.928

[infer_isize] skewness: -0.004; kurtosis: -0.137; ap_prior: 1.70e-05

[infer_isize] inferred maximum insert size: 560 (6.96 sigma)

[bwa_sai2sam_pe_core] time elapses: 35.79 sec

[bwa_sai2sam_pe_core] changing coordinates of 5952 alignments.

[bwa_sai2sam_pe_core] align unmapped mate...

[bwa_paired_sw] 11060 out of 11608 Q17 singletons are mated.

[bwa_paired_sw] 903 out of 1517 Q17 discordant pairs are fixed.

[bwa_sai2sam_pe_core] time elapses: 4.96 sec

[bwa_sai2sam_pe_core] refine gapped alignments... 1.10 sec

[bwa_sai2sam_pe_core] print alignments... 2.35 sec

[bwa_sai2sam_pe_core] 18874368 sequences have been processed.

[bwa_sai2sam_pe_core] convert to sequence coordinate...

[infer_isize] (25, 50, 75) percentile: (453, 463, 473)

[infer_isize] low and high boundaries: 413 and 513 for estimating avg and std

[infer_isize] inferred external isize from 203830 pairs: 463.141 +/- 13.944

[infer_isize] skewness: 0.001; kurtosis: -0.136; ap_prior: 1.59e-05

[infer_isize] inferred maximum insert size: 560 (6.96 sigma)

[bwa_sai2sam_pe_core] time elapses: 35.62 sec

[bwa_sai2sam_pe_core] changing coordinates of 6055 alignments.

[bwa_sai2sam_pe_core] align unmapped mate...

[bwa_paired_sw] 11111 out of 11626 Q17 singletons are mated.

[bwa_paired_sw] 893 out of 1451 Q17 discordant pairs are fixed.

[bwa_sai2sam_pe_core] time elapses: 5.04 sec

[bwa_sai2sam_pe_core] refine gapped alignments... 1.12 sec

[bwa_sai2sam_pe_core] print alignments... 2.29 sec

[bwa_sai2sam_pe_core] 19136512 sequences have been processed.

[bwa_sai2sam_pe_core] convert to sequence coordinate...

[infer_isize] (25, 50, 75) percentile: (453, 463, 473)

[infer_isize] low and high boundaries: 413 and 513 for estimating avg and std

[infer_isize] inferred external isize from 203938 pairs: 463.165 +/- 13.955

[infer_isize] skewness: -0.002; kurtosis: -0.143; ap_prior: 1.72e-05

[infer_isize] inferred maximum insert size: 560 (6.96 sigma)

[bwa_sai2sam_pe_core] time elapses: 36.91 sec

[bwa_sai2sam_pe_core] changing coordinates of 5915 alignments.

[bwa_sai2sam_pe_core] align unmapped mate...

[bwa_paired_sw] 11032 out of 11543 Q17 singletons are mated.

[bwa_paired_sw] 871 out of 1437 Q17 discordant pairs are fixed.

[bwa_sai2sam_pe_core] time elapses: 4.97 sec

[bwa_sai2sam_pe_core] refine gapped alignments... 1.12 sec

[bwa_sai2sam_pe_core] print alignments... 2.22 sec

[bwa_sai2sam_pe_core] 19398656 sequences have been processed.

[bwa_sai2sam_pe_core] convert to sequence coordinate...

[infer_isize] (25, 50, 75) percentile: (453, 463, 473)

[infer_isize] low and high boundaries: 413 and 513 for estimating avg and std

[infer_isize] inferred external isize from 203658 pairs: 463.144 +/- 13.932

[infer_isize] skewness: 0.004; kurtosis: -0.140; ap_prior: 1.70e-05

[infer_isize] inferred maximum insert size: 560 (6.96 sigma)

[bwa_sai2sam_pe_core] time elapses: 35.30 sec

[bwa_sai2sam_pe_core] changing coordinates of 6049 alignments.

[bwa_sai2sam_pe_core] align unmapped mate...

[bwa_paired_sw] 11000 out of 11553 Q17 singletons are mated.

[bwa_paired_sw] 900 out of 1501 Q17 discordant pairs are fixed.

[bwa_sai2sam_pe_core] time elapses: 4.91 sec

[bwa_sai2sam_pe_core] refine gapped alignments... 1.01 sec

[bwa_sai2sam_pe_core] print alignments... 2.16 sec

[bwa_sai2sam_pe_core] 19660800 sequences have been processed.

[bwa_sai2sam_pe_core] convert to sequence coordinate...

[infer_isize] (25, 50, 75) percentile: (453, 463, 473)

[infer_isize] low and high boundaries: 413 and 513 for estimating avg and std

[infer_isize] inferred external isize from 203013 pairs: 463.155 +/- 13.968

[infer_isize] skewness: 0.004; kurtosis: -0.116; ap_prior: 1.65e-05

[infer_isize] inferred maximum insert size: 560 (6.96 sigma)

[bwa_sai2sam_pe_core] time elapses: 31.08 sec

[bwa_sai2sam_pe_core] changing coordinates of 5942 alignments.

[bwa_sai2sam_pe_core] align unmapped mate...

[bwa_paired_sw] 11755 out of 12354 Q17 singletons are mated.

[bwa_paired_sw] 910 out of 1517 Q17 discordant pairs are fixed.

[bwa_sai2sam_pe_core] time elapses: 4.90 sec

[bwa_sai2sam_pe_core] refine gapped alignments... 0.94 sec

[bwa_sai2sam_pe_core] print alignments... 2.09 sec

[bwa_sai2sam_pe_core] 19922944 sequences have been processed.

[bwa_sai2sam_pe_core] convert to sequence coordinate...

[infer_isize] (25, 50, 75) percentile: (453, 463, 473)

[infer_isize] low and high boundaries: 413 and 513 for estimating avg and std

[infer_isize] inferred external isize from 203176 pairs: 463.076 +/- 13.958

[infer_isize] skewness: 0.009; kurtosis: -0.129; ap_prior: 1.71e-05

[infer_isize] inferred maximum insert size: 560 (6.96 sigma)

[bwa_sai2sam_pe_core] time elapses: 32.43 sec

[bwa_sai2sam_pe_core] changing coordinates of 5927 alignments.

[bwa_sai2sam_pe_core] align unmapped mate...

[bwa_paired_sw] 11579 out of 12125 Q17 singletons are mated.

[bwa_paired_sw] 849 out of 1386 Q17 discordant pairs are fixed.

[bwa_sai2sam_pe_core] time elapses: 4.99 sec

[bwa_sai2sam_pe_core] refine gapped alignments... 1.01 sec

[bwa_sai2sam_pe_core] print alignments... 2.10 sec

[bwa_sai2sam_pe_core] 20185088 sequences have been processed.

[bwa_sai2sam_pe_core] convert to sequence coordinate...

[infer_isize] (25, 50, 75) percentile: (453, 463, 473)

[infer_isize] low and high boundaries: 413 and 513 for estimating avg and std

[infer_isize] inferred external isize from 203282 pairs: 463.122 +/- 13.938

[infer_isize] skewness: -0.004; kurtosis: -0.138; ap_prior: 1.73e-05

[infer_isize] inferred maximum insert size: 560 (6.96 sigma)

[bwa_sai2sam_pe_core] time elapses: 33.06 sec

[bwa_sai2sam_pe_core] changing coordinates of 5913 alignments.

[bwa_sai2sam_pe_core] align unmapped mate...

[bwa_paired_sw] 11848 out of 12378 Q17 singletons are mated.

[bwa_paired_sw] 884 out of 1498 Q17 discordant pairs are fixed.

[bwa_sai2sam_pe_core] time elapses: 5.08 sec

[bwa_sai2sam_pe_core] refine gapped alignments... 1.02 sec

[bwa_sai2sam_pe_core] print alignments... 2.09 sec

[bwa_sai2sam_pe_core] 20447232 sequences have been processed.

[bwa_sai2sam_pe_core] convert to sequence coordinate...

[infer_isize] (25, 50, 75) percentile: (453, 463, 473)

[infer_isize] low and high boundaries: 413 and 513 for estimating avg and std

[infer_isize] inferred external isize from 203580 pairs: 463.101 +/- 13.952

[infer_isize] skewness: -0.000; kurtosis: -0.137; ap_prior: 1.81e-05

[infer_isize] inferred maximum insert size: 560 (6.96 sigma)

[bwa_sai2sam_pe_core] time elapses: 33.00 sec

[bwa_sai2sam_pe_core] changing coordinates of 6049 alignments.

[bwa_sai2sam_pe_core] align unmapped mate...

[bwa_paired_sw] 11565 out of 12125 Q17 singletons are mated.

[bwa_paired_sw] 860 out of 1441 Q17 discordant pairs are fixed.

[bwa_sai2sam_pe_core] time elapses: 4.99 sec

[bwa_sai2sam_pe_core] refine gapped alignments... 1.01 sec

[bwa_sai2sam_pe_core] print alignments... 2.12 sec

[bwa_sai2sam_pe_core] 20709376 sequences have been processed.

[bwa_sai2sam_pe_core] convert to sequence coordinate...

[infer_isize] (25, 50, 75) percentile: (453, 463, 473)

[infer_isize] low and high boundaries: 413 and 513 for estimating avg and std

[infer_isize] inferred external isize from 202870 pairs: 463.041 +/- 13.936

[infer_isize] skewness: 0.010; kurtosis: -0.146; ap_prior: 1.62e-05

[infer_isize] inferred maximum insert size: 560 (6.96 sigma)

[bwa_sai2sam_pe_core] time elapses: 34.01 sec

[bwa_sai2sam_pe_core] changing coordinates of 5810 alignments.

[bwa_sai2sam_pe_core] align unmapped mate...

[bwa_paired_sw] 11938 out of 12463 Q17 singletons are mated.

[bwa_paired_sw] 879 out of 1524 Q17 discordant pairs are fixed.

[bwa_sai2sam_pe_core] time elapses: 5.10 sec

[bwa_sai2sam_pe_core] refine gapped alignments... 1.00 sec

[bwa_sai2sam_pe_core] print alignments... 2.08 sec

[bwa_sai2sam_pe_core] 20971520 sequences have been processed.

[bwa_sai2sam_pe_core] convert to sequence coordinate...

[infer_isize] (25, 50, 75) percentile: (453, 463, 473)

[infer_isize] low and high boundaries: 413 and 513 for estimating avg and std

[infer_isize] inferred external isize from 202854 pairs: 463.098 +/- 13.926

[infer_isize] skewness: 0.008; kurtosis: -0.141; ap_prior: 1.75e-05

[infer_isize] inferred maximum insert size: 560 (6.96 sigma)

[bwa_sai2sam_pe_core] time elapses: 33.68 sec

[bwa_sai2sam_pe_core] changing coordinates of 6011 alignments.

[bwa_sai2sam_pe_core] align unmapped mate...

[bwa_paired_sw] 11940 out of 12442 Q17 singletons are mated.

[bwa_paired_sw] 926 out of 1509 Q17 discordant pairs are fixed.

[bwa_sai2sam_pe_core] time elapses: 5.10 sec

[bwa_sai2sam_pe_core] refine gapped alignments... 1.03 sec

[bwa_sai2sam_pe_core] print alignments... 2.17 sec

[bwa_sai2sam_pe_core] 21233664 sequences have been processed.

[bwa_sai2sam_pe_core] convert to sequence coordinate...

[infer_isize] (25, 50, 75) percentile: (453, 463, 473)

[infer_isize] low and high boundaries: 413 and 513 for estimating avg and std

[infer_isize] inferred external isize from 202972 pairs: 463.104 +/- 13.970

[infer_isize] skewness: 0.004; kurtosis: -0.135; ap_prior: 1.75e-05

[infer_isize] inferred maximum insert size: 560 (6.96 sigma)

[bwa_sai2sam_pe_core] time elapses: 33.52 sec

[bwa_sai2sam_pe_core] changing coordinates of 5863 alignments.

[bwa_sai2sam_pe_core] align unmapped mate...

[bwa_paired_sw] 12094 out of 12682 Q17 singletons are mated.

[bwa_paired_sw] 862 out of 1402 Q17 discordant pairs are fixed.

[bwa_sai2sam_pe_core] time elapses: 5.13 sec

[bwa_sai2sam_pe_core] refine gapped alignments... 1.03 sec

[bwa_sai2sam_pe_core] print alignments... 2.22 sec

[bwa_sai2sam_pe_core] 21495808 sequences have been processed.

[bwa_sai2sam_pe_core] convert to sequence coordinate...

[infer_isize] (25, 50, 75) percentile: (453, 463, 473)

[infer_isize] low and high boundaries: 413 and 513 for estimating avg and std

[infer_isize] inferred external isize from 202653 pairs: 463.064 +/- 13.938

[infer_isize] skewness: 0.011; kurtosis: -0.148; ap_prior: 1.55e-05

[infer_isize] inferred maximum insert size: 560 (6.96 sigma)

[bwa_sai2sam_pe_core] time elapses: 33.16 sec

[bwa_sai2sam_pe_core] changing coordinates of 5774 alignments.

[bwa_sai2sam_pe_core] align unmapped mate...

[bwa_paired_sw] 12172 out of 12710 Q17 singletons are mated.

[bwa_paired_sw] 842 out of 1415 Q17 discordant pairs are fixed.

[bwa_sai2sam_pe_core] time elapses: 5.16 sec

[bwa_sai2sam_pe_core] refine gapped alignments... 1.09 sec

[bwa_sai2sam_pe_core] print alignments... 2.27 sec

[bwa_sai2sam_pe_core] 21757952 sequences have been processed.

[bwa_sai2sam_pe_core] convert to sequence coordinate...

[infer_isize] (25, 50, 75) percentile: (453, 463, 473)

[infer_isize] low and high boundaries: 413 and 513 for estimating avg and std

[infer_isize] inferred external isize from 202485 pairs: 463.014 +/- 13.950

[infer_isize] skewness: 0.000; kurtosis: -0.128; ap_prior: 1.97e-05

[infer_isize] inferred maximum insert size: 560 (6.96 sigma)

[bwa_sai2sam_pe_core] time elapses: 35.34 sec

[bwa_sai2sam_pe_core] changing coordinates of 5881 alignments.

[bwa_sai2sam_pe_core] align unmapped mate...

[bwa_paired_sw] 11983 out of 12535 Q17 singletons are mated.

[bwa_paired_sw] 904 out of 1512 Q17 discordant pairs are fixed.

[bwa_sai2sam_pe_core] time elapses: 5.23 sec

[bwa_sai2sam_pe_core] refine gapped alignments... 1.11 sec

[bwa_sai2sam_pe_core] print alignments... 2.33 sec

[bwa_sai2sam_pe_core] 22020096 sequences have been processed.

[bwa_sai2sam_pe_core] convert to sequence coordinate...

[infer_isize] (25, 50, 75) percentile: (453, 463, 473)

[infer_isize] low and high boundaries: 413 and 513 for estimating avg and std

[infer_isize] inferred external isize from 201778 pairs: 463.081 +/- 13.936

[infer_isize] skewness: -0.002; kurtosis: -0.145; ap_prior: 1.85e-05

[infer_isize] inferred maximum insert size: 560 (6.96 sigma)

[bwa_sai2sam_pe_core] time elapses: 34.06 sec

[bwa_sai2sam_pe_core] changing coordinates of 6098 alignments.

[bwa_sai2sam_pe_core] align unmapped mate...

[bwa_paired_sw] 12618 out of 13216 Q17 singletons are mated.

[bwa_paired_sw] 873 out of 1483 Q17 discordant pairs are fixed.

[bwa_sai2sam_pe_core] time elapses: 5.10 sec

[bwa_sai2sam_pe_core] refine gapped alignments... 1.07 sec

[bwa_sai2sam_pe_core] print alignments... 2.17 sec

[bwa_sai2sam_pe_core] 22282240 sequences have been processed.

[bwa_sai2sam_pe_core] convert to sequence coordinate...

[infer_isize] (25, 50, 75) percentile: (453, 463, 473)

[infer_isize] low and high boundaries: 413 and 513 for estimating avg and std

[infer_isize] inferred external isize from 202240 pairs: 463.012 +/- 13.958

[infer_isize] skewness: 0.008; kurtosis: -0.117; ap_prior: 1.67e-05

[infer_isize] inferred maximum insert size: 560 (6.96 sigma)

[bwa_sai2sam_pe_core] time elapses: 19.64 sec

[bwa_sai2sam_pe_core] changing coordinates of 5992 alignments.

[bwa_sai2sam_pe_core] align unmapped mate...

[bwa_paired_sw] 12239 out of 12794 Q17 singletons are mated.

[bwa_paired_sw] 907 out of 1497 Q17 discordant pairs are fixed.

[bwa_sai2sam_pe_core] time elapses: 4.96 sec

[bwa_sai2sam_pe_core] refine gapped alignments... 1.04 sec

[bwa_sai2sam_pe_core] print alignments... 2.18 sec

[bwa_sai2sam_pe_core] 22544384 sequences have been processed.

[bwa_sai2sam_pe_core] convert to sequence coordinate...

[infer_isize] (25, 50, 75) percentile: (453, 463, 473)

[infer_isize] low and high boundaries: 413 and 513 for estimating avg and std

[infer_isize] inferred external isize from 202266 pairs: 463.085 +/- 13.974

[infer_isize] skewness: 0.008; kurtosis: -0.129; ap_prior: 1.85e-05

[infer_isize] inferred maximum insert size: 560 (6.96 sigma)

[bwa_sai2sam_pe_core] time elapses: 19.89 sec

[bwa_sai2sam_pe_core] changing coordinates of 5936 alignments.

[bwa_sai2sam_pe_core] align unmapped mate...

[bwa_paired_sw] 12540 out of 13097 Q17 singletons are mated.

[bwa_paired_sw] 890 out of 1475 Q17 discordant pairs are fixed.

[bwa_sai2sam_pe_core] time elapses: 5.04 sec

[bwa_sai2sam_pe_core] refine gapped alignments... 0.94 sec

[bwa_sai2sam_pe_core] print alignments... 2.05 sec

[bwa_sai2sam_pe_core] 22806528 sequences have been processed.

[bwa_sai2sam_pe_core] convert to sequence coordinate...

[infer_isize] (25, 50, 75) percentile: (453, 463, 473)

[infer_isize] low and high boundaries: 413 and 513 for estimating avg and std

[infer_isize] inferred external isize from 201877 pairs: 463.085 +/- 13.911

[infer_isize] skewness: -0.003; kurtosis: -0.123; ap_prior: 1.79e-05

[infer_isize] inferred maximum insert size: 560 (6.96 sigma)

[bwa_sai2sam_pe_core] time elapses: 22.02 sec

[bwa_sai2sam_pe_core] changing coordinates of 5990 alignments.

[bwa_sai2sam_pe_core] align unmapped mate...

[bwa_paired_sw] 12999 out of 13532 Q17 singletons are mated.

[bwa_paired_sw] 868 out of 1450 Q17 discordant pairs are fixed.

[bwa_sai2sam_pe_core] time elapses: 5.14 sec

[bwa_sai2sam_pe_core] refine gapped alignments... 0.95 sec

[bwa_sai2sam_pe_core] print alignments... 2.05 sec

[bwa_sai2sam_pe_core] 23068672 sequences have been processed.

[bwa_sai2sam_pe_core] convert to sequence coordinate...

[infer_isize] (25, 50, 75) percentile: (453, 463, 473)

[infer_isize] low and high boundaries: 413 and 513 for estimating avg and std

[infer_isize] inferred external isize from 201547 pairs: 463.054 +/- 13.928

[infer_isize] skewness: 0.007; kurtosis: -0.142; ap_prior: 1.68e-05

[infer_isize] inferred maximum insert size: 560 (6.96 sigma)

[bwa_sai2sam_pe_core] time elapses: 23.93 sec

[bwa_sai2sam_pe_core] changing coordinates of 5852 alignments.

[bwa_sai2sam_pe_core] align unmapped mate...

[bwa_paired_sw] 13092 out of 13667 Q17 singletons are mated.

[bwa_paired_sw] 886 out of 1456 Q17 discordant pairs are fixed.

[bwa_sai2sam_pe_core] time elapses: 5.23 sec

[bwa_sai2sam_pe_core] refine gapped alignments... 0.99 sec

[bwa_sai2sam_pe_core] print alignments... 2.12 sec

[bwa_sai2sam_pe_core] 23330816 sequences have been processed.

[bwa_sai2sam_pe_core] convert to sequence coordinate...

[infer_isize] (25, 50, 75) percentile: (453, 463, 473)

[infer_isize] low and high boundaries: 413 and 513 for estimating avg and std

[infer_isize] inferred external isize from 200671 pairs: 463.038 +/- 13.939

[infer_isize] skewness: 0.008; kurtosis: -0.136; ap_prior: 1.69e-05

[infer_isize] inferred maximum insert size: 560 (6.96 sigma)

[bwa_sai2sam_pe_core] time elapses: 23.53 sec

[bwa_sai2sam_pe_core] changing coordinates of 5872 alignments.

[bwa_sai2sam_pe_core] align unmapped mate...

[bwa_paired_sw] 13821 out of 14328 Q17 singletons are mated.

[bwa_paired_sw] 832 out of 1427 Q17 discordant pairs are fixed.

[bwa_sai2sam_pe_core] time elapses: 5.39 sec

[bwa_sai2sam_pe_core] refine gapped alignments... 0.99 sec

[bwa_sai2sam_pe_core] print alignments... 2.07 sec

[bwa_sai2sam_pe_core] 23592960 sequences have been processed.

[bwa_sai2sam_pe_core] convert to sequence coordinate...

[infer_isize] (25, 50, 75) percentile: (453, 463, 473)

[infer_isize] low and high boundaries: 413 and 513 for estimating avg and std

[infer_isize] inferred external isize from 200750 pairs: 463.020 +/- 13.954

[infer_isize] skewness: 0.016; kurtosis: -0.146; ap_prior: 1.78e-05

[infer_isize] inferred maximum insert size: 560 (6.96 sigma)

[bwa_sai2sam_pe_core] time elapses: 23.88 sec

[bwa_sai2sam_pe_core] changing coordinates of 5853 alignments.

[bwa_sai2sam_pe_core] align unmapped mate...

[bwa_paired_sw] 13766 out of 14333 Q17 singletons are mated.

[bwa_paired_sw] 963 out of 1539 Q17 discordant pairs are fixed.

[bwa_sai2sam_pe_core] time elapses: 5.40 sec

[bwa_sai2sam_pe_core] refine gapped alignments... 0.99 sec

[bwa_sai2sam_pe_core] print alignments... 2.08 sec

[bwa_sai2sam_pe_core] 23855104 sequences have been processed.

[bwa_sai2sam_pe_core] convert to sequence coordinate...

[infer_isize] (25, 50, 75) percentile: (453, 463, 473)

[infer_isize] low and high boundaries: 413 and 513 for estimating avg and std

[infer_isize] inferred external isize from 200853 pairs: 463.040 +/- 13.930

[infer_isize] skewness: 0.012; kurtosis: -0.146; ap_prior: 1.73e-05

[infer_isize] inferred maximum insert size: 560 (6.96 sigma)

[bwa_sai2sam_pe_core] time elapses: 20.10 sec

[bwa_sai2sam_pe_core] changing coordinates of 5884 alignments.

[bwa_sai2sam_pe_core] align unmapped mate...

[bwa_paired_sw] 13868 out of 14416 Q17 singletons are mated.

[bwa_paired_sw] 870 out of 1457 Q17 discordant pairs are fixed.

[bwa_sai2sam_pe_core] time elapses: 5.39 sec

[bwa_sai2sam_pe_core] refine gapped alignments... 0.99 sec

[bwa_sai2sam_pe_core] print alignments... 2.10 sec

[bwa_sai2sam_pe_core] 24117248 sequences have been processed.

[bwa_sai2sam_pe_core] convert to sequence coordinate...

[infer_isize] (25, 50, 75) percentile: (453, 463, 473)

[infer_isize] low and high boundaries: 413 and 513 for estimating avg and std

[infer_isize] inferred external isize from 199899 pairs: 462.999 +/- 13.965

[infer_isize] skewness: 0.005; kurtosis: -0.130; ap_prior: 1.70e-05

[infer_isize] inferred maximum insert size: 560 (6.96 sigma)

[bwa_sai2sam_pe_core] time elapses: 19.81 sec

[bwa_sai2sam_pe_core] changing coordinates of 5900 alignments.

[bwa_sai2sam_pe_core] align unmapped mate...

[bwa_paired_sw] 14666 out of 15190 Q17 singletons are mated.

[bwa_paired_sw] 869 out of 1378 Q17 discordant pairs are fixed.

[bwa_sai2sam_pe_core] time elapses: 5.56 sec

[bwa_sai2sam_pe_core] refine gapped alignments... 1.00 sec

[bwa_sai2sam_pe_core] print alignments... 2.06 sec

[bwa_sai2sam_pe_core] 24379392 sequences have been processed.

[bwa_sai2sam_pe_core] convert to sequence coordinate...

[infer_isize] (25, 50, 75) percentile: (453, 463, 473)

[infer_isize] low and high boundaries: 413 and 513 for estimating avg and std

[infer_isize] inferred external isize from 199288 pairs: 463.049 +/- 13.920

[infer_isize] skewness: 0.006; kurtosis: -0.112; ap_prior: 1.73e-05

[infer_isize] inferred maximum insert size: 560 (6.96 sigma)

[bwa_sai2sam_pe_core] time elapses: 19.62 sec

[bwa_sai2sam_pe_core] changing coordinates of 5852 alignments.

[bwa_sai2sam_pe_core] align unmapped mate...

[bwa_paired_sw] 15086 out of 15664 Q17 singletons are mated.

[bwa_paired_sw] 865 out of 1412 Q17 discordant pairs are fixed.

[bwa_sai2sam_pe_core] time elapses: 5.71 sec

[bwa_sai2sam_pe_core] refine gapped alignments... 0.99 sec

[bwa_sai2sam_pe_core] print alignments... 2.05 sec

[bwa_sai2sam_pe_core] 24641536 sequences have been processed.

[bwa_sai2sam_pe_core] convert to sequence coordinate...

[infer_isize] (25, 50, 75) percentile: (453, 463, 473)

[infer_isize] low and high boundaries: 413 and 513 for estimating avg and std

[infer_isize] inferred external isize from 199383 pairs: 463.020 +/- 13.936

[infer_isize] skewness: 0.003; kurtosis: -0.130; ap_prior: 1.77e-05

[infer_isize] inferred maximum insert size: 560 (6.96 sigma)

[bwa_sai2sam_pe_core] time elapses: 19.59 sec

[bwa_sai2sam_pe_core] changing coordinates of 5837 alignments.

[bwa_sai2sam_pe_core] align unmapped mate...

[bwa_paired_sw] 15201 out of 15710 Q17 singletons are mated.

[bwa_paired_sw] 860 out of 1395 Q17 discordant pairs are fixed.

[bwa_sai2sam_pe_core] time elapses: 5.70 sec

[bwa_sai2sam_pe_core] refine gapped alignments... 0.99 sec

[bwa_sai2sam_pe_core] print alignments... 2.04 sec

[bwa_sai2sam_pe_core] 24903680 sequences have been processed.

[bwa_sai2sam_pe_core] convert to sequence coordinate...

[infer_isize] (25, 50, 75) percentile: (453, 463, 473)

[infer_isize] low and high boundaries: 413 and 513 for estimating avg and std

[infer_isize] inferred external isize from 198758 pairs: 463.031 +/- 13.955

[infer_isize] skewness: 0.012; kurtosis: -0.133; ap_prior: 1.62e-05

[infer_isize] inferred maximum insert size: 560 (6.96 sigma)

[bwa_sai2sam_pe_core] time elapses: 22.91 sec

[bwa_sai2sam_pe_core] changing coordinates of 5801 alignments.

[bwa_sai2sam_pe_core] align unmapped mate...

[bwa_paired_sw] 15303 out of 15877 Q17 singletons are mated.

[bwa_paired_sw] 850 out of 1440 Q17 discordant pairs are fixed.

[bwa_sai2sam_pe_core] time elapses: 5.80 sec

[bwa_sai2sam_pe_core] refine gapped alignments... 1.03 sec

[bwa_sai2sam_pe_core] print alignments... 2.07 sec

[bwa_sai2sam_pe_core] 25165824 sequences have been processed.

[bwa_sai2sam_pe_core] convert to sequence coordinate...

[infer_isize] (25, 50, 75) percentile: (453, 463, 473)

[infer_isize] low and high boundaries: 413 and 513 for estimating avg and std

[infer_isize] inferred external isize from 198421 pairs: 463.040 +/- 13.947

[infer_isize] skewness: 0.012; kurtosis: -0.129; ap_prior: 1.77e-05

[infer_isize] inferred maximum insert size: 560 (6.96 sigma)

[bwa_sai2sam_pe_core] time elapses: 23.24 sec

[bwa_sai2sam_pe_core] changing coordinates of 5744 alignments.

[bwa_sai2sam_pe_core] align unmapped mate...

[bwa_paired_sw] 15713 out of 16330 Q17 singletons are mated.

[bwa_paired_sw] 876 out of 1480 Q17 discordant pairs are fixed.

[bwa_sai2sam_pe_core] time elapses: 5.95 sec

[bwa_sai2sam_pe_core] refine gapped alignments... 1.08 sec

[bwa_sai2sam_pe_core] print alignments... 2.14 sec

[bwa_sai2sam_pe_core] 25427968 sequences have been processed.

[bwa_sai2sam_pe_core] convert to sequence coordinate...

[infer_isize] (25, 50, 75) percentile: (453, 463, 473)

[infer_isize] low and high boundaries: 413 and 513 for estimating avg and std

[infer_isize] inferred external isize from 197518 pairs: 462.963 +/- 13.938

[infer_isize] skewness: 0.008; kurtosis: -0.124; ap_prior: 1.80e-05

[infer_isize] inferred maximum insert size: 560 (6.96 sigma)

[bwa_sai2sam_pe_core] time elapses: 23.60 sec

[bwa_sai2sam_pe_core] changing coordinates of 5777 alignments.

[bwa_sai2sam_pe_core] align unmapped mate...

[bwa_paired_sw] 16585 out of 17130 Q17 singletons are mated.

[bwa_paired_sw] 933 out of 1464 Q17 discordant pairs are fixed.

[bwa_sai2sam_pe_core] time elapses: 6.20 sec

[bwa_sai2sam_pe_core] refine gapped alignments... 1.09 sec

[bwa_sai2sam_pe_core] print alignments... 2.11 sec

[bwa_sai2sam_pe_core] 25690112 sequences have been processed.

[bwa_sai2sam_pe_core] convert to sequence coordinate...

[infer_isize] (25, 50, 75) percentile: (453, 463, 473)

[infer_isize] low and high boundaries: 413 and 513 for estimating avg and std

[infer_isize] inferred external isize from 197499 pairs: 462.999 +/- 13.950

[infer_isize] skewness: 0.007; kurtosis: -0.133; ap_prior: 1.72e-05

[infer_isize] inferred maximum insert size: 560 (6.96 sigma)

[bwa_sai2sam_pe_core] time elapses: 23.22 sec

[bwa_sai2sam_pe_core] changing coordinates of 5821 alignments.

[bwa_sai2sam_pe_core] align unmapped mate...

[bwa_paired_sw] 16846 out of 17394 Q17 singletons are mated.

[bwa_paired_sw] 909 out of 1469 Q17 discordant pairs are fixed.

[bwa_sai2sam_pe_core] time elapses: 6.20 sec

[bwa_sai2sam_pe_core] refine gapped alignments... 1.10 sec

[bwa_sai2sam_pe_core] print alignments... 2.06 sec

[bwa_sai2sam_pe_core] 25952256 sequences have been processed.

[bwa_sai2sam_pe_core] convert to sequence coordinate...

[infer_isize] (25, 50, 75) percentile: (453, 463, 473)

[infer_isize] low and high boundaries: 413 and 513 for estimating avg and std

[infer_isize] inferred external isize from 196163 pairs: 462.975 +/- 13.953

[infer_isize] skewness: 0.009; kurtosis: -0.137; ap_prior: 1.76e-05

[infer_isize] inferred maximum insert size: 560 (6.96 sigma)

[bwa_sai2sam_pe_core] time elapses: 20.92 sec

[bwa_sai2sam_pe_core] changing coordinates of 5712 alignments.

[bwa_sai2sam_pe_core] align unmapped mate...

[bwa_paired_sw] 18127 out of 18747 Q17 singletons are mated.

[bwa_paired_sw] 892 out of 1435 Q17 discordant pairs are fixed.

[bwa_sai2sam_pe_core] time elapses: 6.56 sec

[bwa_sai2sam_pe_core] refine gapped alignments... 1.11 sec

[bwa_sai2sam_pe_core] print alignments... 2.06 sec

[bwa_sai2sam_pe_core] 26214400 sequences have been processed.

[bwa_sai2sam_pe_core] convert to sequence coordinate...

[infer_isize] (25, 50, 75) percentile: (453, 463, 473)

[infer_isize] low and high boundaries: 413 and 513 for estimating avg and std

[infer_isize] inferred external isize from 195664 pairs: 462.906 +/- 13.927

[infer_isize] skewness: 0.013; kurtosis: -0.147; ap_prior: 1.58e-05

[infer_isize] inferred maximum insert size: 560 (6.96 sigma)

[bwa_sai2sam_pe_core] time elapses: 23.45 sec

[bwa_sai2sam_pe_core] changing coordinates of 5766 alignments.

[bwa_sai2sam_pe_core] align unmapped mate...

[bwa_paired_sw] 18433 out of 19021 Q17 singletons are mated.

[bwa_paired_sw] 942 out of 1426 Q17 discordant pairs are fixed.

[bwa_sai2sam_pe_core] time elapses: 6.71 sec

[bwa_sai2sam_pe_core] refine gapped alignments... 1.16 sec

[bwa_sai2sam_pe_core] print alignments... 2.12 sec

[bwa_sai2sam_pe_core] 26476544 sequences have been processed.

[bwa_sai2sam_pe_core] convert to sequence coordinate...

[infer_isize] (25, 50, 75) percentile: (453, 463, 473)

[infer_isize] low and high boundaries: 413 and 513 for estimating avg and std

[infer_isize] inferred external isize from 194755 pairs: 462.955 +/- 13.950

[infer_isize] skewness: 0.007; kurtosis: -0.147; ap_prior: 1.71e-05

[infer_isize] inferred maximum insert size: 560 (6.96 sigma)

[bwa_sai2sam_pe_core] time elapses: 23.47 sec

[bwa_sai2sam_pe_core] changing coordinates of 5530 alignments.

[bwa_sai2sam_pe_core] align unmapped mate...

[bwa_paired_sw] 19388 out of 19942 Q17 singletons are mated.

[bwa_paired_sw] 875 out of 1380 Q17 discordant pairs are fixed.

[bwa_sai2sam_pe_core] time elapses: 6.94 sec

[bwa_sai2sam_pe_core] refine gapped alignments... 1.18 sec

[bwa_sai2sam_pe_core] print alignments... 2.12 sec

[bwa_sai2sam_pe_core] 26738688 sequences have been processed.

[bwa_sai2sam_pe_core] convert to sequence coordinate...

[infer_isize] (25, 50, 75) percentile: (453, 463, 473)

[infer_isize] low and high boundaries: 413 and 513 for estimating avg and std

[infer_isize] inferred external isize from 194421 pairs: 462.956 +/- 13.910

[infer_isize] skewness: 0.002; kurtosis: -0.137; ap_prior: 1.77e-05

[infer_isize] inferred maximum insert size: 560 (6.96 sigma)

[bwa_sai2sam_pe_core] time elapses: 23.25 sec

[bwa_sai2sam_pe_core] changing coordinates of 5826 alignments.

[bwa_sai2sam_pe_core] align unmapped mate...

[bwa_paired_sw] 19891 out of 20484 Q17 singletons are mated.

[bwa_paired_sw] 890 out of 1382 Q17 discordant pairs are fixed.

[bwa_sai2sam_pe_core] time elapses: 7.16 sec

[bwa_sai2sam_pe_core] refine gapped alignments... 1.22 sec

[bwa_sai2sam_pe_core] print alignments... 2.16 sec

[bwa_sai2sam_pe_core] 27000832 sequences have been processed.

[bwa_sai2sam_pe_core] convert to sequence coordinate...

[infer_isize] (25, 50, 75) percentile: (453, 463, 473)

[infer_isize] low and high boundaries: 413 and 513 for estimating avg and std

[infer_isize] inferred external isize from 193611 pairs: 462.928 +/- 13.949

[infer_isize] skewness: 0.008; kurtosis: -0.132; ap_prior: 1.57e-05

[infer_isize] inferred maximum insert size: 560 (6.96 sigma)

[bwa_sai2sam_pe_core] time elapses: 22.96 sec

[bwa_sai2sam_pe_core] changing coordinates of 5670 alignments.

[bwa_sai2sam_pe_core] align unmapped mate...

[bwa_paired_sw] 20282 out of 20821 Q17 singletons are mated.

[bwa_paired_sw] 942 out of 1460 Q17 discordant pairs are fixed.

[bwa_sai2sam_pe_core] time elapses: 7.15 sec

[bwa_sai2sam_pe_core] refine gapped alignments... 1.21 sec

[bwa_sai2sam_pe_core] print alignments... 2.13 sec

[bwa_sai2sam_pe_core] 27262976 sequences have been processed.

[bwa_sai2sam_pe_core] convert to sequence coordinate...

[infer_isize] (25, 50, 75) percentile: (453, 463, 473)

[infer_isize] low and high boundaries: 413 and 513 for estimating avg and std

[infer_isize] inferred external isize from 192423 pairs: 462.924 +/- 13.946

[infer_isize] skewness: -0.000; kurtosis: -0.128; ap_prior: 1.68e-05

[infer_isize] inferred maximum insert size: 560 (6.96 sigma)

[bwa_sai2sam_pe_core] time elapses: 18.96 sec

[bwa_sai2sam_pe_core] changing coordinates of 5643 alignments.

[bwa_sai2sam_pe_core] align unmapped mate...

[bwa_paired_sw] 21389 out of 21992 Q17 singletons are mated.

[bwa_paired_sw] 873 out of 1369 Q17 discordant pairs are fixed.

[bwa_sai2sam_pe_core] time elapses: 7.41 sec

[bwa_sai2sam_pe_core] refine gapped alignments... 1.19 sec

[bwa_sai2sam_pe_core] print alignments... 2.08 sec

[bwa_sai2sam_pe_core] 27525120 sequences have been processed.

[bwa_sai2sam_pe_core] convert to sequence coordinate...

[infer_isize] (25, 50, 75) percentile: (453, 463, 473)

[infer_isize] low and high boundaries: 413 and 513 for estimating avg and std

[infer_isize] inferred external isize from 191250 pairs: 462.891 +/- 13.969

[infer_isize] skewness: 0.008; kurtosis: -0.129; ap_prior: 1.66e-05

[infer_isize] inferred maximum insert size: 560 (6.96 sigma)

[bwa_sai2sam_pe_core] time elapses: 18.87 sec

[bwa_sai2sam_pe_core] changing coordinates of 5406 alignments.

[bwa_sai2sam_pe_core] align unmapped mate...

[bwa_paired_sw] 22613 out of 23195 Q17 singletons are mated.

[bwa_paired_sw] 938 out of 1480 Q17 discordant pairs are fixed.

[bwa_sai2sam_pe_core] time elapses: 7.79 sec

[bwa_sai2sam_pe_core] refine gapped alignments... 1.25 sec

[bwa_sai2sam_pe_core] print alignments... 2.07 sec

[bwa_sai2sam_pe_core] 27787264 sequences have been processed.

[bwa_sai2sam_pe_core] convert to sequence coordinate...

[infer_isize] (25, 50, 75) percentile: (453, 463, 473)

[infer_isize] low and high boundaries: 413 and 513 for estimating avg and std

[infer_isize] inferred external isize from 190189 pairs: 462.928 +/- 13.917

[infer_isize] skewness: 0.004; kurtosis: -0.147; ap_prior: 1.72e-05

[infer_isize] inferred maximum insert size: 560 (6.96 sigma)

[bwa_sai2sam_pe_core] time elapses: 19.21 sec

[bwa_sai2sam_pe_core] changing coordinates of 5787 alignments.

[bwa_sai2sam_pe_core] align unmapped mate...

[bwa_paired_sw] 23351 out of 23916 Q17 singletons are mated.

[bwa_paired_sw] 878 out of 1389 Q17 discordant pairs are fixed.

[bwa_sai2sam_pe_core] time elapses: 7.93 sec

[bwa_sai2sam_pe_core] refine gapped alignments... 1.29 sec

[bwa_sai2sam_pe_core] print alignments... 2.04 sec

[bwa_sai2sam_pe_core] 28049408 sequences have been processed.

[bwa_sai2sam_pe_core] convert to sequence coordinate...

[infer_isize] (25, 50, 75) percentile: (453, 463, 473)

[infer_isize] low and high boundaries: 413 and 513 for estimating avg and std

[infer_isize] inferred external isize from 189105 pairs: 462.908 +/- 13.906

[infer_isize] skewness: 0.001; kurtosis: -0.143; ap_prior: 1.56e-05

[infer_isize] inferred maximum insert size: 560 (6.96 sigma)

[bwa_sai2sam_pe_core] time elapses: 22.89 sec

[bwa_sai2sam_pe_core] changing coordinates of 5498 alignments.

[bwa_sai2sam_pe_core] align unmapped mate...

[bwa_paired_sw] 24393 out of 24992 Q17 singletons are mated.

[bwa_paired_sw] 876 out of 1351 Q17 discordant pairs are fixed.

[bwa_sai2sam_pe_core] time elapses: 8.23 sec

[bwa_sai2sam_pe_core] refine gapped alignments... 1.34 sec

[bwa_sai2sam_pe_core] print alignments... 2.05 sec

[bwa_sai2sam_pe_core] 28311552 sequences have been processed.

[bwa_sai2sam_pe_core] convert to sequence coordinate...

[infer_isize] (25, 50, 75) percentile: (453, 463, 472)

[infer_isize] low and high boundaries: 415 and 510 for estimating avg and std

[infer_isize] inferred external isize from 188061 pairs: 462.832 +/- 13.877

[infer_isize] skewness: 0.007; kurtosis: -0.166; ap_prior: 1.77e-05

[infer_isize] inferred maximum insert size: 559 (6.96 sigma)

[bwa_sai2sam_pe_core] time elapses: 25.18 sec

[bwa_sai2sam_pe_core] changing coordinates of 5469 alignments.

[bwa_sai2sam_pe_core] align unmapped mate...

[bwa_paired_sw] 25454 out of 26057 Q17 singletons are mated.

[bwa_paired_sw] 912 out of 1385 Q17 discordant pairs are fixed.

[bwa_sai2sam_pe_core] time elapses: 8.59 sec

[bwa_sai2sam_pe_core] refine gapped alignments... 1.41 sec

[bwa_sai2sam_pe_core] print alignments... 2.14 sec

[bwa_sai2sam_pe_core] 28573696 sequences have been processed.

[bwa_sai2sam_pe_core] convert to sequence coordinate...

[infer_isize] (25, 50, 75) percentile: (453, 463, 473)

[infer_isize] low and high boundaries: 413 and 513 for estimating avg and std

[infer_isize] inferred external isize from 187402 pairs: 462.856 +/- 13.935

[infer_isize] skewness: 0.003; kurtosis: -0.143; ap_prior: 1.71e-05

[infer_isize] inferred maximum insert size: 560 (6.96 sigma)

[bwa_sai2sam_pe_core] time elapses: 23.39 sec

[bwa_sai2sam_pe_core] changing coordinates of 5661 alignments.

[bwa_sai2sam_pe_core] align unmapped mate...

[bwa_paired_sw] 26214 out of 26818 Q17 singletons are mated.

[bwa_paired_sw] 840 out of 1346 Q17 discordant pairs are fixed.

[bwa_sai2sam_pe_core] time elapses: 8.83 sec

[bwa_sai2sam_pe_core] refine gapped alignments... 1.43 sec

[bwa_sai2sam_pe_core] print alignments... 2.09 sec

[bwa_sai2sam_pe_core] 28835840 sequences have been processed.

[bwa_sai2sam_pe_core] convert to sequence coordinate...

[infer_isize] (25, 50, 75) percentile: (453, 463, 473)

[infer_isize] low and high boundaries: 413 and 513 for estimating avg and std

[infer_isize] inferred external isize from 185974 pairs: 462.852 +/- 13.943

[infer_isize] skewness: 0.005; kurtosis: -0.144; ap_prior: 1.65e-05

[infer_isize] inferred maximum insert size: 560 (6.96 sigma)

[bwa_sai2sam_pe_core] time elapses: 22.97 sec

[bwa_sai2sam_pe_core] changing coordinates of 5575 alignments.

[bwa_sai2sam_pe_core] align unmapped mate...

[bwa_paired_sw] 27460 out of 28050 Q17 singletons are mated.

[bwa_paired_sw] 910 out of 1396 Q17 discordant pairs are fixed.

[bwa_sai2sam_pe_core] time elapses: 9.08 sec

[bwa_sai2sam_pe_core] refine gapped alignments... 1.39 sec

[bwa_sai2sam_pe_core] print alignments... 2.10 sec

[bwa_sai2sam_pe_core] 29097984 sequences have been processed.

[bwa_sai2sam_pe_core] convert to sequence coordinate...

[infer_isize] (25, 50, 75) percentile: (453, 463, 473)

[infer_isize] low and high boundaries: 413 and 513 for estimating avg and std

[infer_isize] inferred external isize from 187088 pairs: 462.936 +/- 13.947

[infer_isize] skewness: -0.000; kurtosis: -0.138; ap_prior: 1.63e-05

[infer_isize] inferred maximum insert size: 560 (6.96 sigma)

[bwa_sai2sam_pe_core] time elapses: 18.87 sec

[bwa_sai2sam_pe_core] changing coordinates of 5397 alignments.

[bwa_sai2sam_pe_core] align unmapped mate...

[bwa_paired_sw] 26551 out of 27146 Q17 singletons are mated.

[bwa_paired_sw] 900 out of 1335 Q17 discordant pairs are fixed.

[bwa_sai2sam_pe_core] time elapses: 8.80 sec

[bwa_sai2sam_pe_core] refine gapped alignments... 1.32 sec

[bwa_sai2sam_pe_core] print alignments... 2.10 sec

[bwa_sai2sam_pe_core] 29360128 sequences have been processed.

[bwa_sai2sam_pe_core] convert to sequence coordinate...

[infer_isize] (25, 50, 75) percentile: (453, 463, 473)

[infer_isize] low and high boundaries: 413 and 513 for estimating avg and std

[infer_isize] inferred external isize from 204861 pairs: 463.299 +/- 13.930

[infer_isize] skewness: 0.002; kurtosis: -0.140; ap_prior: 1.73e-05

[infer_isize] inferred maximum insert size: 560 (6.96 sigma)

[bwa_sai2sam_pe_core] time elapses: 22.73 sec

[bwa_sai2sam_pe_core] changing coordinates of 5978 alignments.

[bwa_sai2sam_pe_core] align unmapped mate...

[bwa_paired_sw] 10868 out of 11455 Q17 singletons are mated.

[bwa_paired_sw] 867 out of 1461 Q17 discordant pairs are fixed.

[bwa_sai2sam_pe_core] time elapses: 4.57 sec

[bwa_sai2sam_pe_core] refine gapped alignments... 0.89 sec

[bwa_sai2sam_pe_core] print alignments... 2.05 sec

[bwa_sai2sam_pe_core] 29622272 sequences have been processed.

[bwa_sai2sam_pe_core] convert to sequence coordinate...

[infer_isize] (25, 50, 75) percentile: (453, 463, 473)

[infer_isize] low and high boundaries: 413 and 513 for estimating avg and std

[infer_isize] inferred external isize from 205509 pairs: 463.305 +/- 13.937

[infer_isize] skewness: 0.002; kurtosis: -0.131; ap_prior: 1.75e-05

[infer_isize] inferred maximum insert size: 560 (6.96 sigma)

[bwa_sai2sam_pe_core] time elapses: 22.36 sec

[bwa_sai2sam_pe_core] changing coordinates of 6017 alignments.

[bwa_sai2sam_pe_core] align unmapped mate...

[bwa_paired_sw] 10618 out of 11198 Q17 singletons are mated.

[bwa_paired_sw] 951 out of 1551 Q17 discordant pairs are fixed.

[bwa_sai2sam_pe_core] time elapses: 4.55 sec

[bwa_sai2sam_pe_core] refine gapped alignments... 0.89 sec

[bwa_sai2sam_pe_core] print alignments... 2.06 sec

[bwa_sai2sam_pe_core] 29884416 sequences have been processed.

[bwa_sai2sam_pe_core] convert to sequence coordinate...

[infer_isize] (25, 50, 75) percentile: (453, 463, 473)

[infer_isize] low and high boundaries: 413 and 513 for estimating avg and std

[infer_isize] inferred external isize from 205413 pairs: 463.360 +/- 13.969

[infer_isize] skewness: 0.002; kurtosis: -0.131; ap_prior: 1.91e-05

[infer_isize] inferred maximum insert size: 561 (6.96 sigma)

[bwa_sai2sam_pe_core] time elapses: 24.68 sec

[bwa_sai2sam_pe_core] changing coordinates of 6034 alignments.

[bwa_sai2sam_pe_core] align unmapped mate...

[bwa_paired_sw] 10344 out of 10921 Q17 singletons are mated.

[bwa_paired_sw] 886 out of 1493 Q17 discordant pairs are fixed.

[bwa_sai2sam_pe_core] time elapses: 4.50 sec

[bwa_sai2sam_pe_core] refine gapped alignments... 0.93 sec

[bwa_sai2sam_pe_core] print alignments... 2.16 sec

[bwa_sai2sam_pe_core] 30146560 sequences have been processed.

[bwa_sai2sam_pe_core] convert to sequence coordinate...

[infer_isize] (25, 50, 75) percentile: (453, 463, 473)

[infer_isize] low and high boundaries: 413 and 513 for estimating avg and std

[infer_isize] inferred external isize from 205169 pairs: 463.344 +/- 13.937

[infer_isize] skewness: 0.003; kurtosis: -0.133; ap_prior: 1.66e-05

[infer_isize] inferred maximum insert size: 560 (6.96 sigma)

[bwa_sai2sam_pe_core] time elapses: 28.36 sec

[bwa_sai2sam_pe_core] changing coordinates of 6060 alignments.

[bwa_sai2sam_pe_core] align unmapped mate...

[bwa_paired_sw] 10514 out of 11069 Q17 singletons are mated.

[bwa_paired_sw] 899 out of 1469 Q17 discordant pairs are fixed.

[bwa_sai2sam_pe_core] time elapses: 4.53 sec

[bwa_sai2sam_pe_core] refine gapped alignments... 0.95 sec

[bwa_sai2sam_pe_core] print alignments... 2.21 sec

[bwa_sai2sam_pe_core] 30408704 sequences have been processed.

[bwa_sai2sam_pe_core] convert to sequence coordinate...

[infer_isize] (25, 50, 75) percentile: (453, 463, 473)

[infer_isize] low and high boundaries: 413 and 513 for estimating avg and std

[infer_isize] inferred external isize from 205725 pairs: 463.349 +/- 13.926

[infer_isize] skewness: -0.001; kurtosis: -0.135; ap_prior: 1.83e-05

[infer_isize] inferred maximum insert size: 560 (6.96 sigma)

[bwa_sai2sam_pe_core] time elapses: 28.88 sec

[bwa_sai2sam_pe_core] changing coordinates of 6038 alignments.

[bwa_sai2sam_pe_core] align unmapped mate...

[bwa_paired_sw] 10245 out of 10843 Q17 singletons are mated.

[bwa_paired_sw] 862 out of 1445 Q17 discordant pairs are fixed.

[bwa_sai2sam_pe_core] time elapses: 4.52 sec

[bwa_sai2sam_pe_core] refine gapped alignments... 0.95 sec

[bwa_sai2sam_pe_core] print alignments... 2.15 sec

[bwa_sai2sam_pe_core] 30670848 sequences have been processed.

[bwa_sai2sam_pe_core] convert to sequence coordinate...

[infer_isize] (25, 50, 75) percentile: (453, 463, 473)

[infer_isize] low and high boundaries: 413 and 513 for estimating avg and std

[infer_isize] inferred external isize from 205381 pairs: 463.325 +/- 13.930

[infer_isize] skewness: 0.007; kurtosis: -0.148; ap_prior: 1.80e-05

[infer_isize] inferred maximum insert size: 560 (6.96 sigma)

[bwa_sai2sam_pe_core] time elapses: 29.24 sec

[bwa_sai2sam_pe_core] changing coordinates of 6015 alignments.

[bwa_sai2sam_pe_core] align unmapped mate...

[bwa_paired_sw] 10674 out of 11237 Q17 singletons are mated.

[bwa_paired_sw] 868 out of 1447 Q17 discordant pairs are fixed.

[bwa_sai2sam_pe_core] time elapses: 4.58 sec

[bwa_sai2sam_pe_core] refine gapped alignments... 0.96 sec

[bwa_sai2sam_pe_core] print alignments... 2.07 sec

[bwa_sai2sam_pe_core] 30932992 sequences have been processed.

[bwa_sai2sam_pe_core] convert to sequence coordinate...

[infer_isize] (25, 50, 75) percentile: (453, 463, 473)

[infer_isize] low and high boundaries: 413 and 513 for estimating avg and std

[infer_isize] inferred external isize from 204771 pairs: 463.323 +/- 13.949

[infer_isize] skewness: 0.002; kurtosis: -0.141; ap_prior: 1.61e-05

[infer_isize] inferred maximum insert size: 560 (6.96 sigma)

[bwa_sai2sam_pe_core] time elapses: 25.08 sec

[bwa_sai2sam_pe_core] changing coordinates of 6147 alignments.

[bwa_sai2sam_pe_core] align unmapped mate...

[bwa_paired_sw] 10894 out of 11432 Q17 singletons are mated.

[bwa_paired_sw] 890 out of 1432 Q17 discordant pairs are fixed.

[bwa_sai2sam_pe_core] time elapses: 4.64 sec

[bwa_sai2sam_pe_core] refine gapped alignments... 0.94 sec

[bwa_sai2sam_pe_core] print alignments... 2.16 sec

[bwa_sai2sam_pe_core] 31195136 sequences have been processed.

[bwa_sai2sam_pe_core] convert to sequence coordinate...

[infer_isize] (25, 50, 75) percentile: (453, 463, 473)

[infer_isize] low and high boundaries: 413 and 513 for estimating avg and std

[infer_isize] inferred external isize from 205291 pairs: 463.301 +/- 13.963

[infer_isize] skewness: 0.006; kurtosis: -0.137; ap_prior: 1.94e-05

[infer_isize] inferred maximum insert size: 560 (6.96 sigma)

[bwa_sai2sam_pe_core] time elapses: 24.83 sec

[bwa_sai2sam_pe_core] changing coordinates of 5876 alignments.

[bwa_sai2sam_pe_core] align unmapped mate...

[bwa_paired_sw] 10518 out of 11066 Q17 singletons are mated.

[bwa_paired_sw] 884 out of 1488 Q17 discordant pairs are fixed.

[bwa_sai2sam_pe_core] time elapses: 4.54 sec

[bwa_sai2sam_pe_core] refine gapped alignments... 0.92 sec

[bwa_sai2sam_pe_core] print alignments... 2.15 sec

[bwa_sai2sam_pe_core] 31457280 sequences have been processed.

[bwa_sai2sam_pe_core] convert to sequence coordinate...

[infer_isize] (25, 50, 75) percentile: (453, 463, 473)

[infer_isize] low and high boundaries: 413 and 513 for estimating avg and std

[infer_isize] inferred external isize from 205004 pairs: 463.289 +/- 13.981

[infer_isize] skewness: 0.005; kurtosis: -0.124; ap_prior: 1.70e-05

[infer_isize] inferred maximum insert size: 561 (6.96 sigma)

[bwa_sai2sam_pe_core] time elapses: 25.41 sec

[bwa_sai2sam_pe_core] changing coordinates of 5905 alignments.

[bwa_sai2sam_pe_core] align unmapped mate...

[bwa_paired_sw] 10688 out of 11253 Q17 singletons are mated.

[bwa_paired_sw] 925 out of 1491 Q17 discordant pairs are fixed.

[bwa_sai2sam_pe_core] time elapses: 4.61 sec

[bwa_sai2sam_pe_core] refine gapped alignments... 0.92 sec

[bwa_sai2sam_pe_core] print alignments... 2.09 sec

[bwa_sai2sam_pe_core] 31719424 sequences have been processed.

[bwa_sai2sam_pe_core] convert to sequence coordinate...

[infer_isize] (25, 50, 75) percentile: (453, 463, 473)

[infer_isize] low and high boundaries: 413 and 513 for estimating avg and std

[infer_isize] inferred external isize from 205329 pairs: 463.334 +/- 13.963

[infer_isize] skewness: 0.013; kurtosis: -0.137; ap_prior: 1.70e-05

[infer_isize] inferred maximum insert size: 561 (6.96 sigma)

[bwa_sai2sam_pe_core] time elapses: 25.69 sec

[bwa_sai2sam_pe_core] changing coordinates of 6006 alignments.

[bwa_sai2sam_pe_core] align unmapped mate...

[bwa_paired_sw] 10771 out of 11355 Q17 singletons are mated.

[bwa_paired_sw] 836 out of 1424 Q17 discordant pairs are fixed.

[bwa_sai2sam_pe_core] time elapses: 4.57 sec

[bwa_sai2sam_pe_core] refine gapped alignments... 0.92 sec

[bwa_sai2sam_pe_core] print alignments... 2.07 sec

[bwa_sai2sam_pe_core] 31981568 sequences have been processed.

[bwa_sai2sam_pe_core] convert to sequence coordinate...

[infer_isize] (25, 50, 75) percentile: (453, 463, 473)

[infer_isize] low and high boundaries: 413 and 513 for estimating avg and std

[infer_isize] inferred external isize from 205185 pairs: 463.271 +/- 13.928

[infer_isize] skewness: -0.002; kurtosis: -0.138; ap_prior: 1.97e-05

[infer_isize] inferred maximum insert size: 560 (6.96 sigma)

[bwa_sai2sam_pe_core] time elapses: 25.59 sec

[bwa_sai2sam_pe_core] changing coordinates of 6066 alignments.

[bwa_sai2sam_pe_core] align unmapped mate...

[bwa_paired_sw] 10373 out of 10956 Q17 singletons are mated.

[bwa_paired_sw] 930 out of 1513 Q17 discordant pairs are fixed.

[bwa_sai2sam_pe_core] time elapses: 4.51 sec

[bwa_sai2sam_pe_core] refine gapped alignments... 0.91 sec

[bwa_sai2sam_pe_core] print alignments... 2.07 sec

[bwa_sai2sam_pe_core] 32243712 sequences have been processed.

[bwa_sai2sam_pe_core] convert to sequence coordinate...

[infer_isize] (25, 50, 75) percentile: (453, 463, 473)

[infer_isize] low and high boundaries: 413 and 513 for estimating avg and std

[infer_isize] inferred external isize from 205330 pairs: 463.303 +/- 13.969

[infer_isize] skewness: 0.000; kurtosis: -0.128; ap_prior: 1.73e-05

[infer_isize] inferred maximum insert size: 561 (6.96 sigma)

[bwa_sai2sam_pe_core] time elapses: 25.32 sec

[bwa_sai2sam_pe_core] changing coordinates of 6076 alignments.

[bwa_sai2sam_pe_core] align unmapped mate...

[bwa_paired_sw] 10769 out of 11353 Q17 singletons are mated.

[bwa_paired_sw] 863 out of 1414 Q17 discordant pairs are fixed.

[bwa_sai2sam_pe_core] time elapses: 4.58 sec

[bwa_sai2sam_pe_core] refine gapped alignments... 0.92 sec

[bwa_sai2sam_pe_core] print alignments... 2.06 sec

[bwa_sai2sam_pe_core] 32505856 sequences have been processed.

[bwa_sai2sam_pe_core] convert to sequence coordinate...

[infer_isize] (25, 50, 75) percentile: (453, 463, 473)

[infer_isize] low and high boundaries: 413 and 513 for estimating avg and std

[infer_isize] inferred external isize from 205597 pairs: 463.349 +/- 13.891

[infer_isize] skewness: 0.006; kurtosis: -0.133; ap_prior: 1.77e-05

[infer_isize] inferred maximum insert size: 560 (6.96 sigma)

[bwa_sai2sam_pe_core] time elapses: 25.40 sec

[bwa_sai2sam_pe_core] changing coordinates of 6068 alignments.

[bwa_sai2sam_pe_core] align unmapped mate...

[bwa_paired_sw] 10387 out of 10955 Q17 singletons are mated.

[bwa_paired_sw] 907 out of 1494 Q17 discordant pairs are fixed.

[bwa_sai2sam_pe_core] time elapses: 4.45 sec

[bwa_sai2sam_pe_core] refine gapped alignments... 0.90 sec

[bwa_sai2sam_pe_core] print alignments... 2.06 sec

[bwa_sai2sam_pe_core] 32768000 sequences have been processed.

[bwa_sai2sam_pe_core] convert to sequence coordinate...

[infer_isize] (25, 50, 75) percentile: (453, 463, 473)

[infer_isize] low and high boundaries: 413 and 513 for estimating avg and std

[infer_isize] inferred external isize from 205469 pairs: 463.300 +/- 13.965

[infer_isize] skewness: 0.008; kurtosis: -0.139; ap_prior: 1.69e-05

[infer_isize] inferred maximum insert size: 560 (6.96 sigma)

[bwa_sai2sam_pe_core] time elapses: 23.36 sec

[bwa_sai2sam_pe_core] changing coordinates of 5810 alignments.

[bwa_sai2sam_pe_core] align unmapped mate...

[bwa_paired_sw] 10677 out of 11209 Q17 singletons are mated.

[bwa_paired_sw] 879 out of 1420 Q17 discordant pairs are fixed.

[bwa_sai2sam_pe_core] time elapses: 4.50 sec

[bwa_sai2sam_pe_core] refine gapped alignments... 0.90 sec

[bwa_sai2sam_pe_core] print alignments... 2.05 sec

[bwa_sai2sam_pe_core] 33030144 sequences have been processed.

[bwa_sai2sam_pe_core] convert to sequence coordinate...

[infer_isize] (25, 50, 75) percentile: (453, 463, 473)

[infer_isize] low and high boundaries: 413 and 513 for estimating avg and std

[infer_isize] inferred external isize from 205323 pairs: 463.327 +/- 13.946

[infer_isize] skewness: -0.001; kurtosis: -0.127; ap_prior: 1.79e-05

[infer_isize] inferred maximum insert size: 560 (6.96 sigma)

[bwa_sai2sam_pe_core] time elapses: 25.83 sec

[bwa_sai2sam_pe_core] changing coordinates of 6088 alignments.

[bwa_sai2sam_pe_core] align unmapped mate...

[bwa_paired_sw] 10404 out of 10944 Q17 singletons are mated.

[bwa_paired_sw] 870 out of 1461 Q17 discordant pairs are fixed.

[bwa_sai2sam_pe_core] time elapses: 4.50 sec

[bwa_sai2sam_pe_core] refine gapped alignments... 0.96 sec

[bwa_sai2sam_pe_core] print alignments... 2.15 sec

[bwa_sai2sam_pe_core] 33292288 sequences have been processed.

[bwa_sai2sam_pe_core] convert to sequence coordinate...

[infer_isize] (25, 50, 75) percentile: (453, 463, 473)

[infer_isize] low and high boundaries: 413 and 513 for estimating avg and std

[infer_isize] inferred external isize from 205056 pairs: 463.308 +/- 13.988

[infer_isize] skewness: -0.006; kurtosis: -0.137; ap_prior: 1.73e-05

[infer_isize] inferred maximum insert size: 561 (6.96 sigma)

[bwa_sai2sam_pe_core] time elapses: 26.07 sec

[bwa_sai2sam_pe_core] changing coordinates of 6075 alignments.

[bwa_sai2sam_pe_core] align unmapped mate...

[bwa_paired_sw] 10398 out of 10984 Q17 singletons are mated.

[bwa_paired_sw] 899 out of 1469 Q17 discordant pairs are fixed.

[bwa_sai2sam_pe_core] time elapses: 4.50 sec

[bwa_sai2sam_pe_core] refine gapped alignments... 0.97 sec

[bwa_sai2sam_pe_core] print alignments... 2.19 sec

[bwa_sai2sam_pe_core] 33554432 sequences have been processed.

[bwa_sai2sam_pe_core] convert to sequence coordinate...

[infer_isize] (25, 50, 75) percentile: (453, 463, 473)

[infer_isize] low and high boundaries: 413 and 513 for estimating avg and std

[infer_isize] inferred external isize from 205227 pairs: 463.288 +/- 13.919

[infer_isize] skewness: 0.005; kurtosis: -0.144; ap_prior: 1.69e-05

[infer_isize] inferred maximum insert size: 560 (6.96 sigma)

[bwa_sai2sam_pe_core] time elapses: 27.68 sec

[bwa_sai2sam_pe_core] changing coordinates of 5897 alignments.

[bwa_sai2sam_pe_core] align unmapped mate...

[bwa_paired_sw] 10582 out of 11146 Q17 singletons are mated.

[bwa_paired_sw] 872 out of 1472 Q17 discordant pairs are fixed.

[bwa_sai2sam_pe_core] time elapses: 4.55 sec

[bwa_sai2sam_pe_core] refine gapped alignments... 0.95 sec

[bwa_sai2sam_pe_core] print alignments... 2.09 sec

[bwa_sai2sam_pe_core] 33816576 sequences have been processed.

[bwa_sai2sam_pe_core] convert to sequence coordinate...

[infer_isize] (25, 50, 75) percentile: (453, 463, 473)

[infer_isize] low and high boundaries: 413 and 513 for estimating avg and std

[infer_isize] inferred external isize from 205335 pairs: 463.341 +/- 13.906

[infer_isize] skewness: 0.006; kurtosis: -0.133; ap_prior: 1.71e-05

[infer_isize] inferred maximum insert size: 560 (6.96 sigma)

[bwa_sai2sam_pe_core] time elapses: 24.61 sec

[bwa_sai2sam_pe_core] changing coordinates of 5999 alignments.

[bwa_sai2sam_pe_core] align unmapped mate...

[bwa_paired_sw] 10185 out of 10717 Q17 singletons are mated.

[bwa_paired_sw] 867 out of 1484 Q17 discordant pairs are fixed.

[bwa_sai2sam_pe_core] time elapses: 4.43 sec

[bwa_sai2sam_pe_core] refine gapped alignments... 0.95 sec

[bwa_sai2sam_pe_core] print alignments... 2.17 sec

[bwa_sai2sam_pe_core] 34078720 sequences have been processed.

[bwa_sai2sam_pe_core] convert to sequence coordinate...

[infer_isize] (25, 50, 75) percentile: (453, 463, 473)

[infer_isize] low and high boundaries: 413 and 513 for estimating avg and std

[infer_isize] inferred external isize from 205508 pairs: 463.314 +/- 13.957

[infer_isize] skewness: 0.001; kurtosis: -0.127; ap_prior: 1.84e-05

[infer_isize] inferred maximum insert size: 560 (6.96 sigma)

[bwa_sai2sam_pe_core] time elapses: 24.60 sec

[bwa_sai2sam_pe_core] changing coordinates of 6067 alignments.

[bwa_sai2sam_pe_core] align unmapped mate...

[bwa_paired_sw] 10475 out of 11014 Q17 singletons are mated.

[bwa_paired_sw] 880 out of 1461 Q17 discordant pairs are fixed.

[bwa_sai2sam_pe_core] time elapses: 4.53 sec

[bwa_sai2sam_pe_core] refine gapped alignments... 0.98 sec

[bwa_sai2sam_pe_core] print alignments... 2.23 sec

[bwa_sai2sam_pe_core] 34340864 sequences have been processed.

[bwa_sai2sam_pe_core] convert to sequence coordinate...

[infer_isize] (25, 50, 75) percentile: (453, 463, 473)

[infer_isize] low and high boundaries: 413 and 513 for estimating avg and std

[infer_isize] inferred external isize from 204922 pairs: 463.328 +/- 13.916

[infer_isize] skewness: 0.003; kurtosis: -0.140; ap_prior: 1.68e-05

[infer_isize] inferred maximum insert size: 560 (6.96 sigma)

[bwa_sai2sam_pe_core] time elapses: 26.87 sec

[bwa_sai2sam_pe_core] changing coordinates of 6055 alignments.

[bwa_sai2sam_pe_core] align unmapped mate...

[bwa_paired_sw] 10588 out of 11132 Q17 singletons are mated.

[bwa_paired_sw] 841 out of 1436 Q17 discordant pairs are fixed.

[bwa_sai2sam_pe_core] time elapses: 4.55 sec

[bwa_sai2sam_pe_core] refine gapped alignments... 0.96 sec

[bwa_sai2sam_pe_core] print alignments... 2.18 sec

[bwa_sai2sam_pe_core] 34603008 sequences have been processed.

[bwa_sai2sam_pe_core] convert to sequence coordinate...

[infer_isize] (25, 50, 75) percentile: (453, 463, 473)

[infer_isize] low and high boundaries: 413 and 513 for estimating avg and std

[infer_isize] inferred external isize from 205920 pairs: 463.287 +/- 13.962

[infer_isize] skewness: -0.002; kurtosis: -0.138; ap_prior: 1.67e-05

[infer_isize] inferred maximum insert size: 560 (6.96 sigma)

[bwa_sai2sam_pe_core] time elapses: 27.71 sec

[bwa_sai2sam_pe_core] changing coordinates of 5987 alignments.

[bwa_sai2sam_pe_core] align unmapped mate...

[bwa_paired_sw] 10249 out of 10820 Q17 singletons are mated.

[bwa_paired_sw] 891 out of 1473 Q17 discordant pairs are fixed.

[bwa_sai2sam_pe_core] time elapses: 4.49 sec

[bwa_sai2sam_pe_core] refine gapped alignments... 0.95 sec

[bwa_sai2sam_pe_core] print alignments... 2.11 sec

[bwa_sai2sam_pe_core] 34865152 sequences have been processed.

[bwa_sai2sam_pe_core] convert to sequence coordinate...

[infer_isize] (25, 50, 75) percentile: (453, 463, 473)

[infer_isize] low and high boundaries: 413 and 513 for estimating avg and std

[infer_isize] inferred external isize from 204897 pairs: 463.357 +/- 13.928

[infer_isize] skewness: 0.006; kurtosis: -0.139; ap_prior: 1.61e-05

[infer_isize] inferred maximum insert size: 560 (6.96 sigma)

[bwa_sai2sam_pe_core] time elapses: 28.31 sec

[bwa_sai2sam_pe_core] changing coordinates of 5999 alignments.

[bwa_sai2sam_pe_core] align unmapped mate...

[bwa_paired_sw] 10977 out of 11535 Q17 singletons are mated.

[bwa_paired_sw] 845 out of 1410 Q17 discordant pairs are fixed.

[bwa_sai2sam_pe_core] time elapses: 4.63 sec

[bwa_sai2sam_pe_core] refine gapped alignments... 0.93 sec

[bwa_sai2sam_pe_core] print alignments... 2.11 sec

[bwa_sai2sam_pe_core] 35127296 sequences have been processed.

[bwa_sai2sam_pe_core] convert to sequence coordinate...

[infer_isize] (25, 50, 75) percentile: (453, 463, 473)

[infer_isize] low and high boundaries: 413 and 513 for estimating avg and std

[infer_isize] inferred external isize from 205592 pairs: 463.363 +/- 13.981

[infer_isize] skewness: -0.008; kurtosis: -0.129; ap_prior: 1.67e-05

[infer_isize] inferred maximum insert size: 561 (6.96 sigma)

[bwa_sai2sam_pe_core] time elapses: 28.82 sec

[bwa_sai2sam_pe_core] changing coordinates of 5965 alignments.

[bwa_sai2sam_pe_core] align unmapped mate...

[bwa_paired_sw] 10372 out of 10966 Q17 singletons are mated.

[bwa_paired_sw] 888 out of 1515 Q17 discordant pairs are fixed.

[bwa_sai2sam_pe_core] time elapses: 4.52 sec

[bwa_sai2sam_pe_core] refine gapped alignments... 0.91 sec

[bwa_sai2sam_pe_core] print alignments... 2.10 sec

[bwa_sai2sam_pe_core] 35389440 sequences have been processed.

[bwa_sai2sam_pe_core] convert to sequence coordinate...

[infer_isize] (25, 50, 75) percentile: (453, 463, 473)

[infer_isize] low and high boundaries: 413 and 513 for estimating avg and std

[infer_isize] inferred external isize from 205788 pairs: 463.237 +/- 13.958

[infer_isize] skewness: 0.008; kurtosis: -0.148; ap_prior: 1.56e-05

[infer_isize] inferred maximum insert size: 560 (6.96 sigma)

[bwa_sai2sam_pe_core] time elapses: 28.91 sec

[bwa_sai2sam_pe_core] changing coordinates of 6027 alignments.

[bwa_sai2sam_pe_core] align unmapped mate...

[bwa_paired_sw] 10317 out of 10850 Q17 singletons are mated.

[bwa_paired_sw] 873 out of 1452 Q17 discordant pairs are fixed.

[bwa_sai2sam_pe_core] time elapses: 4.45 sec

[bwa_sai2sam_pe_core] refine gapped alignments... 0.93 sec

[bwa_sai2sam_pe_core] print alignments... 2.07 sec

[bwa_sai2sam_pe_core] 35651584 sequences have been processed.

[bwa_sai2sam_pe_core] convert to sequence coordinate...

[infer_isize] (25, 50, 75) percentile: (453, 463, 473)

[infer_isize] low and high boundaries: 413 and 513 for estimating avg and std

[infer_isize] inferred external isize from 205443 pairs: 463.263 +/- 13.964

[infer_isize] skewness: 0.008; kurtosis: -0.159; ap_prior: 1.83e-05

[infer_isize] inferred maximum insert size: 560 (6.96 sigma)

[bwa_sai2sam_pe_core] time elapses: 28.44 sec

[bwa_sai2sam_pe_core] changing coordinates of 6003 alignments.

[bwa_sai2sam_pe_core] align unmapped mate...

[bwa_paired_sw] 10312 out of 10837 Q17 singletons are mated.

[bwa_paired_sw] 943 out of 1588 Q17 discordant pairs are fixed.

[bwa_sai2sam_pe_core] time elapses: 4.44 sec

[bwa_sai2sam_pe_core] refine gapped alignments... 0.91 sec

[bwa_sai2sam_pe_core] print alignments... 2.08 sec

[bwa_sai2sam_pe_core] 35913728 sequences have been processed.

[bwa_sai2sam_pe_core] convert to sequence coordinate...

[infer_isize] (25, 50, 75) percentile: (453, 463, 473)

[infer_isize] low and high boundaries: 413 and 513 for estimating avg and std

[infer_isize] inferred external isize from 204941 pairs: 463.300 +/- 13.932

[infer_isize] skewness: 0.002; kurtosis: -0.144; ap_prior: 1.72e-05

[infer_isize] inferred maximum insert size: 560 (6.96 sigma)

[bwa_sai2sam_pe_core] time elapses: 26.25 sec

[bwa_sai2sam_pe_core] changing coordinates of 6148 alignments.

[bwa_sai2sam_pe_core] align unmapped mate...

[bwa_paired_sw] 10583 out of 11088 Q17 singletons are mated.

[bwa_paired_sw] 930 out of 1540 Q17 discordant pairs are fixed.

[bwa_sai2sam_pe_core] time elapses: 4.52 sec

[bwa_sai2sam_pe_core] refine gapped alignments... 0.92 sec

[bwa_sai2sam_pe_core] print alignments... 2.05 sec

[bwa_sai2sam_pe_core] 36175872 sequences have been processed.

[bwa_sai2sam_pe_core] convert to sequence coordinate...

[infer_isize] (25, 50, 75) percentile: (453, 463, 473)

[infer_isize] low and high boundaries: 413 and 513 for estimating avg and std

[infer_isize] inferred external isize from 205350 pairs: 463.246 +/- 13.957

[infer_isize] skewness: 0.011; kurtosis: -0.139; ap_prior: 1.74e-05

[infer_isize] inferred maximum insert size: 560 (6.96 sigma)

[bwa_sai2sam_pe_core] time elapses: 26.59 sec

[bwa_sai2sam_pe_core] changing coordinates of 5963 alignments.

[bwa_sai2sam_pe_core] align unmapped mate...

[bwa_paired_sw] 10485 out of 11046 Q17 singletons are mated.

[bwa_paired_sw] 889 out of 1489 Q17 discordant pairs are fixed.

[bwa_sai2sam_pe_core] time elapses: 4.48 sec

[bwa_sai2sam_pe_core] refine gapped alignments... 0.91 sec

[bwa_sai2sam_pe_core] print alignments... 2.05 sec

[bwa_sai2sam_pe_core] 36438016 sequences have been processed.

[bwa_sai2sam_pe_core] convert to sequence coordinate...

[infer_isize] (25, 50, 75) percentile: (453, 463, 473)

[infer_isize] low and high boundaries: 413 and 513 for estimating avg and std

[infer_isize] inferred external isize from 205389 pairs: 463.344 +/- 13.933

[infer_isize] skewness: 0.000; kurtosis: -0.133; ap_prior: 1.79e-05

[infer_isize] inferred maximum insert size: 560 (6.96 sigma)

[bwa_sai2sam_pe_core] time elapses: 28.86 sec

[bwa_sai2sam_pe_core] changing coordinates of 6030 alignments.

[bwa_sai2sam_pe_core] align unmapped mate...

[bwa_paired_sw] 10438 out of 10986 Q17 singletons are mated.

[bwa_paired_sw] 862 out of 1436 Q17 discordant pairs are fixed.

[bwa_sai2sam_pe_core] time elapses: 4.50 sec

[bwa_sai2sam_pe_core] refine gapped alignments... 0.95 sec

[bwa_sai2sam_pe_core] print alignments... 2.18 sec

[bwa_sai2sam_pe_core] 36700160 sequences have been processed.

[bwa_sai2sam_pe_core] convert to sequence coordinate...

[infer_isize] (25, 50, 75) percentile: (453, 463, 473)

[infer_isize] low and high boundaries: 413 and 513 for estimating avg and std

[infer_isize] inferred external isize from 205432 pairs: 463.260 +/- 13.933

[infer_isize] skewness: 0.012; kurtosis: -0.135; ap_prior: 1.76e-05

[infer_isize] inferred maximum insert size: 560 (6.96 sigma)

[bwa_sai2sam_pe_core] time elapses: 27.40 sec

[bwa_sai2sam_pe_core] changing coordinates of 6037 alignments.

[bwa_sai2sam_pe_core] align unmapped mate...

[bwa_paired_sw] 10252 out of 10792 Q17 singletons are mated.

[bwa_paired_sw] 872 out of 1460 Q17 discordant pairs are fixed.

[bwa_sai2sam_pe_core] time elapses: 4.59 sec

[bwa_sai2sam_pe_core] refine gapped alignments... 0.98 sec

[bwa_sai2sam_pe_core] print alignments... 2.27 sec

[bwa_sai2sam_pe_core] 36962304 sequences have been processed.

[bwa_sai2sam_pe_core] convert to sequence coordinate...

[infer_isize] (25, 50, 75) percentile: (453, 463, 473)

[infer_isize] low and high boundaries: 413 and 513 for estimating avg and std

[infer_isize] inferred external isize from 205149 pairs: 463.311 +/- 13.937

[infer_isize] skewness: 0.012; kurtosis: -0.153; ap_prior: 1.76e-05

[infer_isize] inferred maximum insert size: 560 (6.96 sigma)

[bwa_sai2sam_pe_core] time elapses: 32.24 sec

[bwa_sai2sam_pe_core] changing coordinates of 6003 alignments.

[bwa_sai2sam_pe_core] align unmapped mate...

[bwa_paired_sw] 10432 out of 10986 Q17 singletons are mated.

[bwa_paired_sw] 877 out of 1431 Q17 discordant pairs are fixed.

[bwa_sai2sam_pe_core] time elapses: 4.73 sec

[bwa_sai2sam_pe_core] refine gapped alignments... 1.02 sec

[bwa_sai2sam_pe_core] print alignments... 2.09 sec

[bwa_sai2sam_pe_core] 37224448 sequences have been processed.

[bwa_sai2sam_pe_core] convert to sequence coordinate...

[infer_isize] (25, 50, 75) percentile: (453, 463, 473)

[infer_isize] low and high boundaries: 413 and 513 for estimating avg and std

[infer_isize] inferred external isize from 205341 pairs: 463.300 +/- 13.950

[infer_isize] skewness: -0.002; kurtosis: -0.134; ap_prior: 1.77e-05

[infer_isize] inferred maximum insert size: 560 (6.96 sigma)

[bwa_sai2sam_pe_core] time elapses: 31.79 sec

[bwa_sai2sam_pe_core] changing coordinates of 5989 alignments.

[bwa_sai2sam_pe_core] align unmapped mate...

[bwa_paired_sw] 10267 out of 10826 Q17 singletons are mated.

[bwa_paired_sw] 877 out of 1446 Q17 discordant pairs are fixed.

[bwa_sai2sam_pe_core] time elapses: 4.45 sec

[bwa_sai2sam_pe_core] refine gapped alignments... 0.95 sec

[bwa_sai2sam_pe_core] print alignments... 2.15 sec

[bwa_sai2sam_pe_core] 37486592 sequences have been processed.

[bwa_sai2sam_pe_core] convert to sequence coordinate...

[infer_isize] (25, 50, 75) percentile: (453, 463, 473)

[infer_isize] low and high boundaries: 413 and 513 for estimating avg and std

[infer_isize] inferred external isize from 205526 pairs: 463.297 +/- 13.976

[infer_isize] skewness: -0.003; kurtosis: -0.125; ap_prior: 1.71e-05

[infer_isize] inferred maximum insert size: 561 (6.96 sigma)

[bwa_sai2sam_pe_core] time elapses: 29.53 sec

[bwa_sai2sam_pe_core] changing coordinates of 6178 alignments.

[bwa_sai2sam_pe_core] align unmapped mate...

[bwa_paired_sw] 10164 out of 10668 Q17 singletons are mated.

[bwa_paired_sw] 861 out of 1469 Q17 discordant pairs are fixed.

[bwa_sai2sam_pe_core] time elapses: 4.42 sec

[bwa_sai2sam_pe_core] refine gapped alignments... 0.94 sec

[bwa_sai2sam_pe_core] print alignments... 2.08 sec

[bwa_sai2sam_pe_core] 37748736 sequences have been processed.

[bwa_sai2sam_pe_core] convert to sequence coordinate...

[infer_isize] (25, 50, 75) percentile: (453, 463, 473)

[infer_isize] low and high boundaries: 413 and 513 for estimating avg and std

[infer_isize] inferred external isize from 205230 pairs: 463.360 +/- 13.929

[infer_isize] skewness: 0.003; kurtosis: -0.146; ap_prior: 1.54e-05

[infer_isize] inferred maximum insert size: 560 (6.96 sigma)

[bwa_sai2sam_pe_core] time elapses: 27.76 sec

[bwa_sai2sam_pe_core] changing coordinates of 5950 alignments.

[bwa_sai2sam_pe_core] align unmapped mate...

[bwa_paired_sw] 10559 out of 11101 Q17 singletons are mated.

[bwa_paired_sw] 846 out of 1429 Q17 discordant pairs are fixed.

[bwa_sai2sam_pe_core] time elapses: 4.53 sec

[bwa_sai2sam_pe_core] refine gapped alignments... 0.93 sec

[bwa_sai2sam_pe_core] print alignments... 2.08 sec

[bwa_sai2sam_pe_core] 38010880 sequences have been processed.

[bwa_sai2sam_pe_core] convert to sequence coordinate...

[infer_isize] (25, 50, 75) percentile: (453, 463, 473)

[infer_isize] low and high boundaries: 413 and 513 for estimating avg and std

[infer_isize] inferred external isize from 204839 pairs: 463.305 +/- 13.928

[infer_isize] skewness: 0.000; kurtosis: -0.145; ap_prior: 1.72e-05

[infer_isize] inferred maximum insert size: 560 (6.96 sigma)

[bwa_sai2sam_pe_core] time elapses: 27.75 sec

[bwa_sai2sam_pe_core] changing coordinates of 6042 alignments.

[bwa_sai2sam_pe_core] align unmapped mate...

[bwa_paired_sw] 10894 out of 11501 Q17 singletons are mated.

[bwa_paired_sw] 940 out of 1523 Q17 discordant pairs are fixed.

[bwa_sai2sam_pe_core] time elapses: 4.67 sec

[bwa_sai2sam_pe_core] refine gapped alignments... 0.93 sec

[bwa_sai2sam_pe_core] print alignments... 2.08 sec

[bwa_sai2sam_pe_core] 38273024 sequences have been processed.

[bwa_sai2sam_pe_core] convert to sequence coordinate...

[infer_isize] (25, 50, 75) percentile: (453, 463, 473)

[infer_isize] low and high boundaries: 413 and 513 for estimating avg and std

[infer_isize] inferred external isize from 204992 pairs: 463.306 +/- 13.939

[infer_isize] skewness: -0.001; kurtosis: -0.127; ap_prior: 1.58e-05

[infer_isize] inferred maximum insert size: 560 (6.96 sigma)

[bwa_sai2sam_pe_core] time elapses: 29.01 sec

[bwa_sai2sam_pe_core] changing coordinates of 5956 alignments.

[bwa_sai2sam_pe_core] align unmapped mate...

[bwa_paired_sw] 10602 out of 11124 Q17 singletons are mated.

[bwa_paired_sw] 929 out of 1528 Q17 discordant pairs are fixed.

[bwa_sai2sam_pe_core] time elapses: 4.57 sec

[bwa_sai2sam_pe_core] refine gapped alignments... 0.94 sec

[bwa_sai2sam_pe_core] print alignments... 2.09 sec

[bwa_sai2sam_pe_core] 38535168 sequences have been processed.

[bwa_sai2sam_pe_core] convert to sequence coordinate...

[infer_isize] (25, 50, 75) percentile: (453, 463, 473)

[infer_isize] low and high boundaries: 413 and 513 for estimating avg and std

[infer_isize] inferred external isize from 205028 pairs: 463.299 +/- 13.937

[infer_isize] skewness: -0.001; kurtosis: -0.153; ap_prior: 1.57e-05

[infer_isize] inferred maximum insert size: 560 (6.96 sigma)

[bwa_sai2sam_pe_core] time elapses: 28.32 sec

[bwa_sai2sam_pe_core] changing coordinates of 5929 alignments.

[bwa_sai2sam_pe_core] align unmapped mate...

[bwa_paired_sw] 10706 out of 11245 Q17 singletons are mated.

[bwa_paired_sw] 906 out of 1507 Q17 discordant pairs are fixed.

[bwa_sai2sam_pe_core] time elapses: 4.62 sec

[bwa_sai2sam_pe_core] refine gapped alignments... 0.93 sec

[bwa_sai2sam_pe_core] print alignments... 2.08 sec

[bwa_sai2sam_pe_core] 38797312 sequences have been processed.

[bwa_sai2sam_pe_core] convert to sequence coordinate...

[infer_isize] (25, 50, 75) percentile: (453, 463, 473)

[infer_isize] low and high boundaries: 413 and 513 for estimating avg and std

[infer_isize] inferred external isize from 205080 pairs: 463.296 +/- 13.958

[infer_isize] skewness: 0.010; kurtosis: -0.148; ap_prior: 1.67e-05

[infer_isize] inferred maximum insert size: 560 (6.96 sigma)

[bwa_sai2sam_pe_core] time elapses: 28.81 sec

[bwa_sai2sam_pe_core] changing coordinates of 5963 alignments.

[bwa_sai2sam_pe_core] align unmapped mate...

[bwa_paired_sw] 10493 out of 11044 Q17 singletons are mated.

[bwa_paired_sw] 863 out of 1455 Q17 discordant pairs are fixed.

[bwa_sai2sam_pe_core] time elapses: 4.66 sec

[bwa_sai2sam_pe_core] refine gapped alignments... 0.96 sec

[bwa_sai2sam_pe_core] print alignments... 2.10 sec

[bwa_sai2sam_pe_core] 39059456 sequences have been processed.

[bwa_sai2sam_pe_core] convert to sequence coordinate...

[infer_isize] (25, 50, 75) percentile: (453, 463, 473)

[infer_isize] low and high boundaries: 413 and 513 for estimating avg and std

[infer_isize] inferred external isize from 205111 pairs: 463.308 +/- 13.937

[infer_isize] skewness: 0.001; kurtosis: -0.134; ap_prior: 1.71e-05

[infer_isize] inferred maximum insert size: 560 (6.96 sigma)

[bwa_sai2sam_pe_core] time elapses: 31.32 sec

[bwa_sai2sam_pe_core] changing coordinates of 6141 alignments.

[bwa_sai2sam_pe_core] align unmapped mate...

[bwa_paired_sw] 10575 out of 11092 Q17 singletons are mated.

[bwa_paired_sw] 906 out of 1468 Q17 discordant pairs are fixed.

[bwa_sai2sam_pe_core] time elapses: 4.75 sec

[bwa_sai2sam_pe_core] refine gapped alignments... 0.99 sec

[bwa_sai2sam_pe_core] print alignments... 2.18 sec

[bwa_sai2sam_pe_core] 39321600 sequences have been processed.

[bwa_sai2sam_pe_core] convert to sequence coordinate...

[infer_isize] (25, 50, 75) percentile: (453, 463, 473)

[infer_isize] low and high boundaries: 413 and 513 for estimating avg and std

[infer_isize] inferred external isize from 205091 pairs: 463.251 +/- 13.942

[infer_isize] skewness: 0.000; kurtosis: -0.141; ap_prior: 1.79e-05

[infer_isize] inferred maximum insert size: 560 (6.96 sigma)

[bwa_sai2sam_pe_core] time elapses: 31.34 sec

[bwa_sai2sam_pe_core] changing coordinates of 6032 alignments.

[bwa_sai2sam_pe_core] align unmapped mate...

[bwa_paired_sw] 10591 out of 11104 Q17 singletons are mated.

[bwa_paired_sw] 836 out of 1447 Q17 discordant pairs are fixed.

[bwa_sai2sam_pe_core] time elapses: 4.79 sec

[bwa_sai2sam_pe_core] refine gapped alignments... 1.01 sec

[bwa_sai2sam_pe_core] print alignments... 2.16 sec

[bwa_sai2sam_pe_core] 39583744 sequences have been processed.

[bwa_sai2sam_pe_core] convert to sequence coordinate...

[infer_isize] (25, 50, 75) percentile: (453, 463, 473)

[infer_isize] low and high boundaries: 413 and 513 for estimating avg and std

[infer_isize] inferred external isize from 205248 pairs: 463.313 +/- 13.959

[infer_isize] skewness: -0.003; kurtosis: -0.133; ap_prior: 1.69e-05

[infer_isize] inferred maximum insert size: 560 (6.96 sigma)

[bwa_sai2sam_pe_core] time elapses: 31.96 sec

[bwa_sai2sam_pe_core] changing coordinates of 6118 alignments.

[bwa_sai2sam_pe_core] align unmapped mate...

[bwa_paired_sw] 10706 out of 11246 Q17 singletons are mated.

[bwa_paired_sw] 910 out of 1472 Q17 discordant pairs are fixed.

[bwa_sai2sam_pe_core] time elapses: 4.79 sec

[bwa_sai2sam_pe_core] refine gapped alignments... 1.01 sec

[bwa_sai2sam_pe_core] print alignments... 2.17 sec

[bwa_sai2sam_pe_core] 39845888 sequences have been processed.

[bwa_sai2sam_pe_core] convert to sequence coordinate...

[infer_isize] (25, 50, 75) percentile: (453, 463, 473)

[infer_isize] low and high boundaries: 413 and 513 for estimating avg and std

[infer_isize] inferred external isize from 205217 pairs: 463.308 +/- 13.961

[infer_isize] skewness: 0.005; kurtosis: -0.146; ap_prior: 1.86e-05

[infer_isize] inferred maximum insert size: 560 (6.96 sigma)

[bwa_sai2sam_pe_core] time elapses: 31.50 sec

[bwa_sai2sam_pe_core] changing coordinates of 5938 alignments.

[bwa_sai2sam_pe_core] align unmapped mate...

[bwa_paired_sw] 10701 out of 11240 Q17 singletons are mated.

[bwa_paired_sw] 915 out of 1499 Q17 discordant pairs are fixed.

[bwa_sai2sam_pe_core] time elapses: 4.62 sec

[bwa_sai2sam_pe_core] refine gapped alignments... 0.98 sec

[bwa_sai2sam_pe_core] print alignments... 2.10 sec

[bwa_sai2sam_pe_core] 40108032 sequences have been processed.

[bwa_sai2sam_pe_core] convert to sequence coordinate...

[infer_isize] (25, 50, 75) percentile: (453, 463, 473)

[infer_isize] low and high boundaries: 413 and 513 for estimating avg and std

[infer_isize] inferred external isize from 205075 pairs: 463.282 +/- 13.907

[infer_isize] skewness: 0.001; kurtosis: -0.126; ap_prior: 1.73e-05

[infer_isize] inferred maximum insert size: 560 (6.96 sigma)

[bwa_sai2sam_pe_core] time elapses: 29.09 sec

[bwa_sai2sam_pe_core] changing coordinates of 6105 alignments.

[bwa_sai2sam_pe_core] align unmapped mate...

[bwa_paired_sw] 10655 out of 11180 Q17 singletons are mated.

[bwa_paired_sw] 878 out of 1505 Q17 discordant pairs are fixed.

[bwa_sai2sam_pe_core] time elapses: 4.68 sec

[bwa_sai2sam_pe_core] refine gapped alignments... 1.01 sec

[bwa_sai2sam_pe_core] print alignments... 2.22 sec

[bwa_sai2sam_pe_core] 40370176 sequences have been processed.

[bwa_sai2sam_pe_core] convert to sequence coordinate...

[infer_isize] (25, 50, 75) percentile: (453, 463, 473)

[infer_isize] low and high boundaries: 413 and 513 for estimating avg and std

[infer_isize] inferred external isize from 205031 pairs: 463.263 +/- 13.981

[infer_isize] skewness: 0.005; kurtosis: -0.123; ap_prior: 1.58e-05

[infer_isize] inferred maximum insert size: 561 (6.96 sigma)

[bwa_sai2sam_pe_core] time elapses: 30.59 sec

[bwa_sai2sam_pe_core] changing coordinates of 5932 alignments.

[bwa_sai2sam_pe_core] align unmapped mate...

[bwa_paired_sw] 10591 out of 11127 Q17 singletons are mated.

[bwa_paired_sw] 863 out of 1431 Q17 discordant pairs are fixed.

[bwa_sai2sam_pe_core] time elapses: 4.53 sec

[bwa_sai2sam_pe_core] refine gapped alignments... 1.02 sec

[bwa_sai2sam_pe_core] print alignments... 2.22 sec

[bwa_sai2sam_pe_core] 40632320 sequences have been processed.

[bwa_sai2sam_pe_core] convert to sequence coordinate...

[infer_isize] (25, 50, 75) percentile: (453, 463, 473)

[infer_isize] low and high boundaries: 413 and 513 for estimating avg and std

[infer_isize] inferred external isize from 205048 pairs: 463.222 +/- 13.964

[infer_isize] skewness: 0.005; kurtosis: -0.144; ap_prior: 1.85e-05

[infer_isize] inferred maximum insert size: 560 (6.96 sigma)

[bwa_sai2sam_pe_core] time elapses: 28.25 sec

[bwa_sai2sam_pe_core] changing coordinates of 5952 alignments.

[bwa_sai2sam_pe_core] align unmapped mate...

[bwa_paired_sw] 10819 out of 11331 Q17 singletons are mated.

[bwa_paired_sw] 879 out of 1494 Q17 discordant pairs are fixed.

[bwa_sai2sam_pe_core] time elapses: 4.64 sec

[bwa_sai2sam_pe_core] refine gapped alignments... 1.00 sec

[bwa_sai2sam_pe_core] print alignments... 2.16 sec

[bwa_sai2sam_pe_core] 40894464 sequences have been processed.

[bwa_sai2sam_pe_core] convert to sequence coordinate...

[infer_isize] (25, 50, 75) percentile: (453, 463, 473)

[infer_isize] low and high boundaries: 413 and 513 for estimating avg and std

[infer_isize] inferred external isize from 204799 pairs: 463.281 +/- 13.953

[infer_isize] skewness: 0.003; kurtosis: -0.148; ap_prior: 1.81e-05

[infer_isize] inferred maximum insert size: 560 (6.96 sigma)

[bwa_sai2sam_pe_core] time elapses: 30.07 sec

[bwa_sai2sam_pe_core] changing coordinates of 5941 alignments.

[bwa_sai2sam_pe_core] align unmapped mate...

[bwa_paired_sw] 10597 out of 11131 Q17 singletons are mated.

[bwa_paired_sw] 903 out of 1522 Q17 discordant pairs are fixed.

[bwa_sai2sam_pe_core] time elapses: 4.57 sec

[bwa_sai2sam_pe_core] refine gapped alignments... 0.94 sec

[bwa_sai2sam_pe_core] print alignments... 2.08 sec

[bwa_sai2sam_pe_core] 41156608 sequences have been processed.

[bwa_sai2sam_pe_core] convert to sequence coordinate...

[infer_isize] (25, 50, 75) percentile: (453, 463, 473)

[infer_isize] low and high boundaries: 413 and 513 for estimating avg and std

[infer_isize] inferred external isize from 204936 pairs: 463.276 +/- 13.953

[infer_isize] skewness: -0.002; kurtosis: -0.155; ap_prior: 1.80e-05

[infer_isize] inferred maximum insert size: 560 (6.96 sigma)

[bwa_sai2sam_pe_core] time elapses: 26.43 sec

[bwa_sai2sam_pe_core] changing coordinates of 5905 alignments.

[bwa_sai2sam_pe_core] align unmapped mate...

[bwa_paired_sw] 10703 out of 11215 Q17 singletons are mated.

[bwa_paired_sw] 884 out of 1486 Q17 discordant pairs are fixed.

[bwa_sai2sam_pe_core] time elapses: 4.59 sec

[bwa_sai2sam_pe_core] refine gapped alignments... 0.96 sec

[bwa_sai2sam_pe_core] print alignments... 2.08 sec

[bwa_sai2sam_pe_core] 41418752 sequences have been processed.

[bwa_sai2sam_pe_core] convert to sequence coordinate...

[infer_isize] (25, 50, 75) percentile: (453, 463, 473)

[infer_isize] low and high boundaries: 413 and 513 for estimating avg and std

[infer_isize] inferred external isize from 205187 pairs: 463.203 +/- 13.914

[infer_isize] skewness: -0.002; kurtosis: -0.147; ap_prior: 1.76e-05

[infer_isize] inferred maximum insert size: 560 (6.96 sigma)

[bwa_sai2sam_pe_core] time elapses: 26.82 sec

[bwa_sai2sam_pe_core] changing coordinates of 5919 alignments.

[bwa_sai2sam_pe_core] align unmapped mate...

[bwa_paired_sw] 10597 out of 11149 Q17 singletons are mated.

[bwa_paired_sw] 887 out of 1442 Q17 discordant pairs are fixed.

[bwa_sai2sam_pe_core] time elapses: 4.53 sec

[bwa_sai2sam_pe_core] refine gapped alignments... 0.95 sec

[bwa_sai2sam_pe_core] print alignments... 2.09 sec

[bwa_sai2sam_pe_core] 41680896 sequences have been processed.

[bwa_sai2sam_pe_core] convert to sequence coordinate...

[infer_isize] (25, 50, 75) percentile: (453, 463, 473)

[infer_isize] low and high boundaries: 413 and 513 for estimating avg and std

[infer_isize] inferred external isize from 204761 pairs: 463.326 +/- 13.953

[infer_isize] skewness: -0.003; kurtosis: -0.129; ap_prior: 1.62e-05

[infer_isize] inferred maximum insert size: 560 (6.96 sigma)

[bwa_sai2sam_pe_core] time elapses: 23.84 sec

[bwa_sai2sam_pe_core] changing coordinates of 6017 alignments.

[bwa_sai2sam_pe_core] align unmapped mate...

[bwa_paired_sw] 10900 out of 11433 Q17 singletons are mated.

[bwa_paired_sw] 909 out of 1502 Q17 discordant pairs are fixed.

[bwa_sai2sam_pe_core] time elapses: 4.61 sec

[bwa_sai2sam_pe_core] refine gapped alignments... 0.94 sec

[bwa_sai2sam_pe_core] print alignments... 2.08 sec

[bwa_sai2sam_pe_core] 41943040 sequences have been processed.

[bwa_sai2sam_pe_core] convert to sequence coordinate...

[infer_isize] (25, 50, 75) percentile: (453, 463, 473)

[infer_isize] low and high boundaries: 413 and 513 for estimating avg and std

[infer_isize] inferred external isize from 204827 pairs: 463.288 +/- 13.952

[infer_isize] skewness: -0.006; kurtosis: -0.139; ap_prior: 1.81e-05

[infer_isize] inferred maximum insert size: 560 (6.96 sigma)

[bwa_sai2sam_pe_core] time elapses: 23.81 sec

[bwa_sai2sam_pe_core] changing coordinates of 6090 alignments.

[bwa_sai2sam_pe_core] align unmapped mate...

[bwa_paired_sw] 10873 out of 11398 Q17 singletons are mated.

[bwa_paired_sw] 867 out of 1454 Q17 discordant pairs are fixed.

[bwa_sai2sam_pe_core] time elapses: 4.59 sec

[bwa_sai2sam_pe_core] refine gapped alignments... 0.92 sec

[bwa_sai2sam_pe_core] print alignments... 2.09 sec

[bwa_sai2sam_pe_core] 42205184 sequences have been processed.

[bwa_sai2sam_pe_core] convert to sequence coordinate...

[infer_isize] (25, 50, 75) percentile: (453, 463, 473)

[infer_isize] low and high boundaries: 413 and 513 for estimating avg and std

[infer_isize] inferred external isize from 204896 pairs: 463.270 +/- 13.949

[infer_isize] skewness: 0.003; kurtosis: -0.143; ap_prior: 1.83e-05

[infer_isize] inferred maximum insert size: 560 (6.96 sigma)

[bwa_sai2sam_pe_core] time elapses: 23.47 sec

[bwa_sai2sam_pe_core] changing coordinates of 6003 alignments.

[bwa_sai2sam_pe_core] align unmapped mate...

[bwa_paired_sw] 10766 out of 11300 Q17 singletons are mated.

[bwa_paired_sw] 922 out of 1523 Q17 discordant pairs are fixed.

[bwa_sai2sam_pe_core] time elapses: 4.57 sec

[bwa_sai2sam_pe_core] refine gapped alignments... 0.94 sec

[bwa_sai2sam_pe_core] print alignments... 2.10 sec

[bwa_sai2sam_pe_core] 42467328 sequences have been processed.

[bwa_sai2sam_pe_core] convert to sequence coordinate...

[infer_isize] (25, 50, 75) percentile: (453, 463, 473)

[infer_isize] low and high boundaries: 413 and 513 for estimating avg and std

[infer_isize] inferred external isize from 204732 pairs: 463.275 +/- 13.946

[infer_isize] skewness: -0.002; kurtosis: -0.148; ap_prior: 1.77e-05

[infer_isize] inferred maximum insert size: 560 (6.96 sigma)

[bwa_sai2sam_pe_core] time elapses: 23.64 sec

[bwa_sai2sam_pe_core] changing coordinates of 5951 alignments.

[bwa_sai2sam_pe_core] align unmapped mate...

[bwa_paired_sw] 11023 out of 11567 Q17 singletons are mated.

[bwa_paired_sw] 908 out of 1475 Q17 discordant pairs are fixed.

[bwa_sai2sam_pe_core] time elapses: 4.64 sec

[bwa_sai2sam_pe_core] refine gapped alignments... 0.91 sec

[bwa_sai2sam_pe_core] print alignments... 2.07 sec

[bwa_sai2sam_pe_core] 42729472 sequences have been processed.

[bwa_sai2sam_pe_core] convert to sequence coordinate...

[infer_isize] (25, 50, 75) percentile: (453, 463, 473)

[infer_isize] low and high boundaries: 413 and 513 for estimating avg and std

[infer_isize] inferred external isize from 204402 pairs: 463.246 +/- 14.000

[infer_isize] skewness: 0.005; kurtosis: -0.127; ap_prior: 1.66e-05

[infer_isize] inferred maximum insert size: 561 (6.96 sigma)

[bwa_sai2sam_pe_core] time elapses: 23.94 sec

[bwa_sai2sam_pe_core] changing coordinates of 6087 alignments.

[bwa_sai2sam_pe_core] align unmapped mate...

[bwa_paired_sw] 10894 out of 11406 Q17 singletons are mated.

[bwa_paired_sw] 870 out of 1438 Q17 discordant pairs are fixed.

[bwa_sai2sam_pe_core] time elapses: 4.61 sec

[bwa_sai2sam_pe_core] refine gapped alignments... 0.94 sec

[bwa_sai2sam_pe_core] print alignments... 2.17 sec

[bwa_sai2sam_pe_core] 42991616 sequences have been processed.

[bwa_sai2sam_pe_core] convert to sequence coordinate...

[infer_isize] (25, 50, 75) percentile: (453, 463, 473)

[infer_isize] low and high boundaries: 413 and 513 for estimating avg and std

[infer_isize] inferred external isize from 204295 pairs: 463.216 +/- 13.952

[infer_isize] skewness: -0.003; kurtosis: -0.131; ap_prior: 1.65e-05

[infer_isize] inferred maximum insert size: 560 (6.96 sigma)

[bwa_sai2sam_pe_core] time elapses: 24.94 sec

[bwa_sai2sam_pe_core] changing coordinates of 6145 alignments.

[bwa_sai2sam_pe_core] align unmapped mate...

[bwa_paired_sw] 11055 out of 11586 Q17 singletons are mated.

[bwa_paired_sw] 865 out of 1439 Q17 discordant pairs are fixed.

[bwa_sai2sam_pe_core] time elapses: 4.67 sec

[bwa_sai2sam_pe_core] refine gapped alignments... 0.99 sec

[bwa_sai2sam_pe_core] print alignments... 2.17 sec

[bwa_sai2sam_pe_core] 43253760 sequences have been processed.

[bwa_sai2sam_pe_core] convert to sequence coordinate...

[infer_isize] (25, 50, 75) percentile: (453, 463, 473)

[infer_isize] low and high boundaries: 413 and 513 for estimating avg and std

[infer_isize] inferred external isize from 204876 pairs: 463.259 +/- 13.929

[infer_isize] skewness: 0.006; kurtosis: -0.139; ap_prior: 1.76e-05

[infer_isize] inferred maximum insert size: 560 (6.96 sigma)

[bwa_sai2sam_pe_core] time elapses: 24.42 sec

[bwa_sai2sam_pe_core] changing coordinates of 5897 alignments.

[bwa_sai2sam_pe_core] align unmapped mate...

[bwa_paired_sw] 11009 out of 11537 Q17 singletons are mated.

[bwa_paired_sw] 889 out of 1470 Q17 discordant pairs are fixed.

[bwa_sai2sam_pe_core] time elapses: 4.74 sec

[bwa_sai2sam_pe_core] refine gapped alignments... 0.97 sec

[bwa_sai2sam_pe_core] print alignments... 2.26 sec

[bwa_sai2sam_pe_core] 43515904 sequences have been processed.

[bwa_sai2sam_pe_core] convert to sequence coordinate...

[infer_isize] (25, 50, 75) percentile: (453, 463, 473)

[infer_isize] low and high boundaries: 413 and 513 for estimating avg and std

[infer_isize] inferred external isize from 204686 pairs: 463.246 +/- 13.956

[infer_isize] skewness: 0.005; kurtosis: -0.142; ap_prior: 1.65e-05

[infer_isize] inferred maximum insert size: 560 (6.96 sigma)

[bwa_sai2sam_pe_core] time elapses: 33.72 sec

[bwa_sai2sam_pe_core] changing coordinates of 5920 alignments.

[bwa_sai2sam_pe_core] align unmapped mate...

[bwa_paired_sw] 11117 out of 11673 Q17 singletons are mated.

[bwa_paired_sw] 901 out of 1516 Q17 discordant pairs are fixed.

[bwa_sai2sam_pe_core] time elapses: 4.89 sec

[bwa_sai2sam_pe_core] refine gapped alignments... 1.08 sec

[bwa_sai2sam_pe_core] print alignments... 2.24 sec

[bwa_sai2sam_pe_core] 43778048 sequences have been processed.

[bwa_sai2sam_pe_core] convert to sequence coordinate...

[infer_isize] (25, 50, 75) percentile: (453, 463, 473)

[infer_isize] low and high boundaries: 413 and 513 for estimating avg and std

[infer_isize] inferred external isize from 204412 pairs: 463.225 +/- 13.970

[infer_isize] skewness: 0.009; kurtosis: -0.146; ap_prior: 1.87e-05

[infer_isize] inferred maximum insert size: 560 (6.96 sigma)

[bwa_sai2sam_pe_core] time elapses: 33.93 sec

[bwa_sai2sam_pe_core] changing coordinates of 5897 alignments.

[bwa_sai2sam_pe_core] align unmapped mate...

[bwa_paired_sw] 11062 out of 11590 Q17 singletons are mated.

[bwa_paired_sw] 902 out of 1458 Q17 discordant pairs are fixed.

[bwa_sai2sam_pe_core] time elapses: 4.91 sec

[bwa_sai2sam_pe_core] refine gapped alignments... 1.08 sec

[bwa_sai2sam_pe_core] print alignments... 2.24 sec

[bwa_sai2sam_pe_core] 44040192 sequences have been processed.

[bwa_sai2sam_pe_core] convert to sequence coordinate...

[infer_isize] (25, 50, 75) percentile: (453, 463, 473)

[infer_isize] low and high boundaries: 413 and 513 for estimating avg and std

[infer_isize] inferred external isize from 204617 pairs: 463.258 +/- 13.937

[infer_isize] skewness: -0.001; kurtosis: -0.145; ap_prior: 1.64e-05

[infer_isize] inferred maximum insert size: 560 (6.96 sigma)

[bwa_sai2sam_pe_core] time elapses: 31.02 sec

[bwa_sai2sam_pe_core] changing coordinates of 5928 alignments.

[bwa_sai2sam_pe_core] align unmapped mate...

[bwa_paired_sw] 11185 out of 11738 Q17 singletons are mated.

[bwa_paired_sw] 880 out of 1461 Q17 discordant pairs are fixed.

[bwa_sai2sam_pe_core] time elapses: 4.71 sec

[bwa_sai2sam_pe_core] refine gapped alignments... 0.98 sec

[bwa_sai2sam_pe_core] print alignments... 2.11 sec

[bwa_sai2sam_pe_core] 44302336 sequences have been processed.

[bwa_sai2sam_pe_core] convert to sequence coordinate...

[infer_isize] (25, 50, 75) percentile: (453, 463, 473)

[infer_isize] low and high boundaries: 413 and 513 for estimating avg and std

[infer_isize] inferred external isize from 204317 pairs: 463.187 +/- 13.942

[infer_isize] skewness: -0.001; kurtosis: -0.144; ap_prior: 1.60e-05

[infer_isize] inferred maximum insert size: 560 (6.96 sigma)

[bwa_sai2sam_pe_core] time elapses: 28.45 sec

[bwa_sai2sam_pe_core] changing coordinates of 5901 alignments.

[bwa_sai2sam_pe_core] align unmapped mate...

[bwa_paired_sw] 11285 out of 11819 Q17 singletons are mated.

[bwa_paired_sw] 892 out of 1493 Q17 discordant pairs are fixed.

[bwa_sai2sam_pe_core] time elapses: 4.75 sec

[bwa_sai2sam_pe_core] refine gapped alignments... 0.98 sec

[bwa_sai2sam_pe_core] print alignments... 2.13 sec

[bwa_sai2sam_pe_core] 44564480 sequences have been processed.

[bwa_sai2sam_pe_core] convert to sequence coordinate...

[infer_isize] (25, 50, 75) percentile: (453, 463, 473)

[infer_isize] low and high boundaries: 413 and 513 for estimating avg and std

[infer_isize] inferred external isize from 204193 pairs: 463.207 +/- 13.992

[infer_isize] skewness: 0.001; kurtosis: -0.149; ap_prior: 1.76e-05

[infer_isize] inferred maximum insert size: 561 (6.96 sigma)

[bwa_sai2sam_pe_core] time elapses: 29.82 sec

[bwa_sai2sam_pe_core] changing coordinates of 6035 alignments.

[bwa_sai2sam_pe_core] align unmapped mate...

[bwa_paired_sw] 11146 out of 11691 Q17 singletons are mated.

[bwa_paired_sw] 910 out of 1471 Q17 discordant pairs are fixed.

[bwa_sai2sam_pe_core] time elapses: 4.76 sec

[bwa_sai2sam_pe_core] refine gapped alignments... 0.95 sec

[bwa_sai2sam_pe_core] print alignments... 2.10 sec

[bwa_sai2sam_pe_core] 44826624 sequences have been processed.

[bwa_sai2sam_pe_core] convert to sequence coordinate...

[infer_isize] (25, 50, 75) percentile: (453, 463, 473)

[infer_isize] low and high boundaries: 413 and 513 for estimating avg and std

[infer_isize] inferred external isize from 204116 pairs: 463.214 +/- 13.954

[infer_isize] skewness: 0.004; kurtosis: -0.114; ap_prior: 1.70e-05

[infer_isize] inferred maximum insert size: 560 (6.96 sigma)

[bwa_sai2sam_pe_core] time elapses: 22.02 sec

[bwa_sai2sam_pe_core] changing coordinates of 6024 alignments.

[bwa_sai2sam_pe_core] align unmapped mate...

[bwa_paired_sw] 11505 out of 12064 Q17 singletons are mated.

[bwa_paired_sw] 875 out of 1446 Q17 discordant pairs are fixed.

[bwa_sai2sam_pe_core] time elapses: 4.74 sec

[bwa_sai2sam_pe_core] refine gapped alignments... 0.95 sec

[bwa_sai2sam_pe_core] print alignments... 2.14 sec

[bwa_sai2sam_pe_core] 45088768 sequences have been processed.

[bwa_sai2sam_pe_core] convert to sequence coordinate...

[infer_isize] (25, 50, 75) percentile: (453, 463, 473)

[infer_isize] low and high boundaries: 413 and 513 for estimating avg and std

[infer_isize] inferred external isize from 204157 pairs: 463.262 +/- 13.936

[infer_isize] skewness: -0.002; kurtosis: -0.142; ap_prior: 1.78e-05

[infer_isize] inferred maximum insert size: 560 (6.96 sigma)

[bwa_sai2sam_pe_core] time elapses: 22.00 sec

[bwa_sai2sam_pe_core] changing coordinates of 5887 alignments.

[bwa_sai2sam_pe_core] align unmapped mate...

[bwa_paired_sw] 11645 out of 12217 Q17 singletons are mated.

[bwa_paired_sw] 852 out of 1452 Q17 discordant pairs are fixed.

[bwa_sai2sam_pe_core] time elapses: 4.79 sec

[bwa_sai2sam_pe_core] refine gapped alignments... 0.93 sec

[bwa_sai2sam_pe_core] print alignments... 2.09 sec

[bwa_sai2sam_pe_core] 45350912 sequences have been processed.

[bwa_sai2sam_pe_core] convert to sequence coordinate...

[infer_isize] (25, 50, 75) percentile: (453, 463, 473)

[infer_isize] low and high boundaries: 413 and 513 for estimating avg and std

[infer_isize] inferred external isize from 203810 pairs: 463.180 +/- 13.971

[infer_isize] skewness: -0.005; kurtosis: -0.114; ap_prior: 1.59e-05

[infer_isize] inferred maximum insert size: 560 (6.96 sigma)

[bwa_sai2sam_pe_core] time elapses: 21.96 sec

[bwa_sai2sam_pe_core] changing coordinates of 5970 alignments.

[bwa_sai2sam_pe_core] align unmapped mate...

[bwa_paired_sw] 11550 out of 12109 Q17 singletons are mated.

[bwa_paired_sw] 851 out of 1408 Q17 discordant pairs are fixed.

[bwa_sai2sam_pe_core] time elapses: 4.73 sec

[bwa_sai2sam_pe_core] refine gapped alignments... 0.95 sec

[bwa_sai2sam_pe_core] print alignments... 2.09 sec

[bwa_sai2sam_pe_core] 45613056 sequences have been processed.

[bwa_sai2sam_pe_core] convert to sequence coordinate...

[infer_isize] (25, 50, 75) percentile: (453, 463, 473)

[infer_isize] low and high boundaries: 413 and 513 for estimating avg and std

[infer_isize] inferred external isize from 203912 pairs: 463.235 +/- 13.956

[infer_isize] skewness: 0.009; kurtosis: -0.154; ap_prior: 1.93e-05

[infer_isize] inferred maximum insert size: 560 (6.96 sigma)

[bwa_sai2sam_pe_core] time elapses: 22.32 sec

[bwa_sai2sam_pe_core] changing coordinates of 6025 alignments.

[bwa_sai2sam_pe_core] align unmapped mate...

[bwa_paired_sw] 11509 out of 12074 Q17 singletons are mated.

[bwa_paired_sw] 924 out of 1497 Q17 discordant pairs are fixed.

[bwa_sai2sam_pe_core] time elapses: 4.77 sec

[bwa_sai2sam_pe_core] refine gapped alignments... 0.93 sec

[bwa_sai2sam_pe_core] print alignments... 2.11 sec

[bwa_sai2sam_pe_core] 45875200 sequences have been processed.

[bwa_sai2sam_pe_core] convert to sequence coordinate...

[infer_isize] (25, 50, 75) percentile: (453, 463, 473)

[infer_isize] low and high boundaries: 413 and 513 for estimating avg and std

[infer_isize] inferred external isize from 203624 pairs: 463.263 +/- 13.931

[infer_isize] skewness: 0.004; kurtosis: -0.118; ap_prior: 1.72e-05

[infer_isize] inferred maximum insert size: 560 (6.96 sigma)

[bwa_sai2sam_pe_core] time elapses: 22.28 sec

[bwa_sai2sam_pe_core] changing coordinates of 5938 alignments.

[bwa_sai2sam_pe_core] align unmapped mate...

[bwa_paired_sw] 11826 out of 12345 Q17 singletons are mated.

[bwa_paired_sw] 869 out of 1451 Q17 discordant pairs are fixed.

[bwa_sai2sam_pe_core] time elapses: 4.84 sec

[bwa_sai2sam_pe_core] refine gapped alignments... 0.94 sec

[bwa_sai2sam_pe_core] print alignments... 2.09 sec

[bwa_sai2sam_pe_core] 46137344 sequences have been processed.

[bwa_sai2sam_pe_core] convert to sequence coordinate...

[infer_isize] (25, 50, 75) percentile: (453, 463, 473)

[infer_isize] low and high boundaries: 413 and 513 for estimating avg and std

[infer_isize] inferred external isize from 203576 pairs: 463.179 +/- 13.979

[infer_isize] skewness: 0.013; kurtosis: -0.136; ap_prior: 1.67e-05

[infer_isize] inferred maximum insert size: 560 (6.96 sigma)

[bwa_sai2sam_pe_core] time elapses: 22.67 sec

[bwa_sai2sam_pe_core] changing coordinates of 5966 alignments.

[bwa_sai2sam_pe_core] align unmapped mate...

[bwa_paired_sw] 11752 out of 12306 Q17 singletons are mated.

[bwa_paired_sw] 847 out of 1469 Q17 discordant pairs are fixed.

[bwa_sai2sam_pe_core] time elapses: 4.86 sec

[bwa_sai2sam_pe_core] refine gapped alignments... 0.93 sec

[bwa_sai2sam_pe_core] print alignments... 2.08 sec

[bwa_sai2sam_pe_core] 46399488 sequences have been processed.

[bwa_sai2sam_pe_core] convert to sequence coordinate...

[infer_isize] (25, 50, 75) percentile: (453, 463, 473)

[infer_isize] low and high boundaries: 413 and 513 for estimating avg and std

[infer_isize] inferred external isize from 203483 pairs: 463.261 +/- 13.937

[infer_isize] skewness: 0.001; kurtosis: -0.143; ap_prior: 1.59e-05

[infer_isize] inferred maximum insert size: 560 (6.96 sigma)

[bwa_sai2sam_pe_core] time elapses: 22.13 sec

[bwa_sai2sam_pe_core] changing coordinates of 6015 alignments.

[bwa_sai2sam_pe_core] align unmapped mate...

[bwa_paired_sw] 12047 out of 12617 Q17 singletons are mated.

[bwa_paired_sw] 894 out of 1458 Q17 discordant pairs are fixed.

[bwa_sai2sam_pe_core] time elapses: 4.94 sec

[bwa_sai2sam_pe_core] refine gapped alignments... 0.94 sec

[bwa_sai2sam_pe_core] print alignments... 2.09 sec

[bwa_sai2sam_pe_core] 46661632 sequences have been processed.

[bwa_sai2sam_pe_core] convert to sequence coordinate...

[infer_isize] (25, 50, 75) percentile: (453, 463, 473)

[infer_isize] low and high boundaries: 413 and 513 for estimating avg and std

[infer_isize] inferred external isize from 203089 pairs: 463.194 +/- 13.938

[infer_isize] skewness: 0.007; kurtosis: -0.140; ap_prior: 1.61e-05

[infer_isize] inferred maximum insert size: 560 (6.96 sigma)

[bwa_sai2sam_pe_core] time elapses: 22.36 sec

[bwa_sai2sam_pe_core] changing coordinates of 5955 alignments.

[bwa_sai2sam_pe_core] align unmapped mate...

[bwa_paired_sw] 12155 out of 12734 Q17 singletons are mated.

[bwa_paired_sw] 921 out of 1500 Q17 discordant pairs are fixed.

[bwa_sai2sam_pe_core] time elapses: 4.99 sec

[bwa_sai2sam_pe_core] refine gapped alignments... 0.96 sec

[bwa_sai2sam_pe_core] print alignments... 2.11 sec

[bwa_sai2sam_pe_core] 46923776 sequences have been processed.

[bwa_sai2sam_pe_core] convert to sequence coordinate...

[infer_isize] (25, 50, 75) percentile: (453, 463, 473)

[infer_isize] low and high boundaries: 413 and 513 for estimating avg and std

[infer_isize] inferred external isize from 203329 pairs: 463.234 +/- 13.945

[infer_isize] skewness: 0.001; kurtosis: -0.138; ap_prior: 1.61e-05

[infer_isize] inferred maximum insert size: 560 (6.96 sigma)

[bwa_sai2sam_pe_core] time elapses: 25.51 sec

[bwa_sai2sam_pe_core] changing coordinates of 5933 alignments.

[bwa_sai2sam_pe_core] align unmapped mate...

[bwa_paired_sw] 12124 out of 12655 Q17 singletons are mated.

[bwa_paired_sw] 794 out of 1380 Q17 discordant pairs are fixed.

[bwa_sai2sam_pe_core] time elapses: 4.92 sec

[bwa_sai2sam_pe_core] refine gapped alignments... 0.99 sec

[bwa_sai2sam_pe_core] print alignments... 2.06 sec

[bwa_sai2sam_pe_core] 47185920 sequences have been processed.

[bwa_sai2sam_pe_core] convert to sequence coordinate...

[infer_isize] (25, 50, 75) percentile: (453, 463, 473)

[infer_isize] low and high boundaries: 413 and 513 for estimating avg and std

[infer_isize] inferred external isize from 203595 pairs: 463.209 +/- 13.986

[infer_isize] skewness: 0.007; kurtosis: -0.139; ap_prior: 1.76e-05

[infer_isize] inferred maximum insert size: 561 (6.96 sigma)

[bwa_sai2sam_pe_core] time elapses: 25.73 sec

[bwa_sai2sam_pe_core] changing coordinates of 5974 alignments.

[bwa_sai2sam_pe_core] align unmapped mate...

[bwa_paired_sw] 12003 out of 12607 Q17 singletons are mated.

[bwa_paired_sw] 900 out of 1488 Q17 discordant pairs are fixed.

[bwa_sai2sam_pe_core] time elapses: 4.92 sec

[bwa_sai2sam_pe_core] refine gapped alignments... 0.97 sec

[bwa_sai2sam_pe_core] print alignments... 2.13 sec

[bwa_sai2sam_pe_core] 47448064 sequences have been processed.

[bwa_sai2sam_pe_core] convert to sequence coordinate...

[infer_isize] (25, 50, 75) percentile: (453, 463, 473)

[infer_isize] low and high boundaries: 413 and 513 for estimating avg and std

[infer_isize] inferred external isize from 203012 pairs: 463.200 +/- 13.898

[infer_isize] skewness: 0.010; kurtosis: -0.139; ap_prior: 1.63e-05

[infer_isize] inferred maximum insert size: 560 (6.96 sigma)

[bwa_sai2sam_pe_core] time elapses: 32.11 sec

[bwa_sai2sam_pe_core] changing coordinates of 6022 alignments.

[bwa_sai2sam_pe_core] align unmapped mate...

[bwa_paired_sw] 12525 out of 13109 Q17 singletons are mated.

[bwa_paired_sw] 888 out of 1440 Q17 discordant pairs are fixed.

[bwa_sai2sam_pe_core] time elapses: 5.35 sec

[bwa_sai2sam_pe_core] refine gapped alignments... 1.05 sec

[bwa_sai2sam_pe_core] print alignments... 2.22 sec

[bwa_sai2sam_pe_core] 47710208 sequences have been processed.

[bwa_sai2sam_pe_core] convert to sequence coordinate...

[infer_isize] (25, 50, 75) percentile: (453, 463, 473)

[infer_isize] low and high boundaries: 413 and 513 for estimating avg and std

[infer_isize] inferred external isize from 203337 pairs: 463.220 +/- 13.962

[infer_isize] skewness: 0.006; kurtosis: -0.139; ap_prior: 1.62e-05

[infer_isize] inferred maximum insert size: 560 (6.96 sigma)

[bwa_sai2sam_pe_core] time elapses: 32.01 sec

[bwa_sai2sam_pe_core] changing coordinates of 5799 alignments.

[bwa_sai2sam_pe_core] align unmapped mate...

[bwa_paired_sw] 12177 out of 12751 Q17 singletons are mated.

[bwa_paired_sw] 834 out of 1396 Q17 discordant pairs are fixed.

[bwa_sai2sam_pe_core] time elapses: 5.23 sec

[bwa_sai2sam_pe_core] refine gapped alignments... 1.06 sec

[bwa_sai2sam_pe_core] print alignments... 2.19 sec

[bwa_sai2sam_pe_core] 47972352 sequences have been processed.

[bwa_sai2sam_pe_core] convert to sequence coordinate...

[infer_isize] (25, 50, 75) percentile: (453, 463, 473)

[infer_isize] low and high boundaries: 413 and 513 for estimating avg and std

[infer_isize] inferred external isize from 202842 pairs: 463.177 +/- 13.946

[infer_isize] skewness: -0.002; kurtosis: -0.146; ap_prior: 1.71e-05

[infer_isize] inferred maximum insert size: 560 (6.96 sigma)

[bwa_sai2sam_pe_core] time elapses: 32.12 sec

[bwa_sai2sam_pe_core] changing coordinates of 5986 alignments.

[bwa_sai2sam_pe_core] align unmapped mate...

[bwa_paired_sw] 12529 out of 13118 Q17 singletons are mated.

[bwa_paired_sw] 904 out of 1441 Q17 discordant pairs are fixed.

[bwa_sai2sam_pe_core] time elapses: 5.17 sec

[bwa_sai2sam_pe_core] refine gapped alignments... 1.00 sec

[bwa_sai2sam_pe_core] print alignments... 2.11 sec

[bwa_sai2sam_pe_core] 48234496 sequences have been processed.

[bwa_sai2sam_pe_core] convert to sequence coordinate...

[infer_isize] (25, 50, 75) percentile: (453, 463, 473)

[infer_isize] low and high boundaries: 413 and 513 for estimating avg and std

[infer_isize] inferred external isize from 202094 pairs: 463.154 +/- 13.936

[infer_isize] skewness: -0.001; kurtosis: -0.130; ap_prior: 1.68e-05

[infer_isize] inferred maximum insert size: 560 (6.96 sigma)

[bwa_sai2sam_pe_core] time elapses: 30.24 sec

[bwa_sai2sam_pe_core] changing coordinates of 5992 alignments.

[bwa_sai2sam_pe_core] align unmapped mate...

[bwa_paired_sw] 12928 out of 13463 Q17 singletons are mated.

[bwa_paired_sw] 853 out of 1419 Q17 discordant pairs are fixed.

[bwa_sai2sam_pe_core] time elapses: 5.12 sec

[bwa_sai2sam_pe_core] refine gapped alignments... 0.94 sec

[bwa_sai2sam_pe_core] print alignments... 2.09 sec

[bwa_sai2sam_pe_core] 48496640 sequences have been processed.

[bwa_sai2sam_pe_core] convert to sequence coordinate...

[infer_isize] (25, 50, 75) percentile: (453, 463, 473)

[infer_isize] low and high boundaries: 413 and 513 for estimating avg and std

[infer_isize] inferred external isize from 202456 pairs: 463.214 +/- 13.955

[infer_isize] skewness: 0.006; kurtosis: -0.119; ap_prior: 1.80e-05

[infer_isize] inferred maximum insert size: 560 (6.96 sigma)

[bwa_sai2sam_pe_core] time elapses: 29.66 sec

[bwa_sai2sam_pe_core] changing coordinates of 6065 alignments.

[bwa_sai2sam_pe_core] align unmapped mate...

[bwa_paired_sw] 12740 out of 13272 Q17 singletons are mated.

[bwa_paired_sw] 876 out of 1439 Q17 discordant pairs are fixed.

[bwa_sai2sam_pe_core] time elapses: 5.21 sec

[bwa_sai2sam_pe_core] refine gapped alignments... 0.99 sec

[bwa_sai2sam_pe_core] print alignments... 2.09 sec

[bwa_sai2sam_pe_core] 48758784 sequences have been processed.

[bwa_sai2sam_pe_core] convert to sequence coordinate...

[infer_isize] (25, 50, 75) percentile: (453, 463, 473)

[infer_isize] low and high boundaries: 413 and 513 for estimating avg and std

[infer_isize] inferred external isize from 202709 pairs: 463.125 +/- 13.911

[infer_isize] skewness: 0.010; kurtosis: -0.136; ap_prior: 1.53e-05

[infer_isize] inferred maximum insert size: 560 (6.96 sigma)

[bwa_sai2sam_pe_core] time elapses: 29.05 sec

[bwa_sai2sam_pe_core] changing coordinates of 5831 alignments.

[bwa_sai2sam_pe_core] align unmapped mate...

[bwa_paired_sw] 12850 out of 13442 Q17 singletons are mated.

[bwa_paired_sw] 853 out of 1397 Q17 discordant pairs are fixed.

[bwa_sai2sam_pe_core] time elapses: 5.22 sec

[bwa_sai2sam_pe_core] refine gapped alignments... 0.98 sec

[bwa_sai2sam_pe_core] print alignments... 2.09 sec

[bwa_sai2sam_pe_core] 49020928 sequences have been processed.

[bwa_sai2sam_pe_core] convert to sequence coordinate...

[infer_isize] (25, 50, 75) percentile: (453, 463, 473)

[infer_isize] low and high boundaries: 413 and 513 for estimating avg and std

[infer_isize] inferred external isize from 202450 pairs: 463.155 +/- 13.919

[infer_isize] skewness: 0.010; kurtosis: -0.135; ap_prior: 1.72e-05

[infer_isize] inferred maximum insert size: 560 (6.96 sigma)

[bwa_sai2sam_pe_core] time elapses: 30.89 sec

[bwa_sai2sam_pe_core] changing coordinates of 6073 alignments.

[bwa_sai2sam_pe_core] align unmapped mate...

[bwa_paired_sw] 12798 out of 13326 Q17 singletons are mated.

[bwa_paired_sw] 873 out of 1426 Q17 discordant pairs are fixed.

[bwa_sai2sam_pe_core] time elapses: 5.28 sec

[bwa_sai2sam_pe_core] refine gapped alignments... 1.03 sec

[bwa_sai2sam_pe_core] print alignments... 2.08 sec

[bwa_sai2sam_pe_core] 49283072 sequences have been processed.

[bwa_sai2sam_pe_core] convert to sequence coordinate...

[infer_isize] (25, 50, 75) percentile: (453, 463, 473)

[infer_isize] low and high boundaries: 413 and 513 for estimating avg and std

[infer_isize] inferred external isize from 201943 pairs: 463.142 +/- 13.954

[infer_isize] skewness: -0.001; kurtosis: -0.127; ap_prior: 1.79e-05

[infer_isize] inferred maximum insert size: 560 (6.96 sigma)

[bwa_sai2sam_pe_core] time elapses: 30.13 sec

[bwa_sai2sam_pe_core] changing coordinates of 5914 alignments.

[bwa_sai2sam_pe_core] align unmapped mate...

[bwa_paired_sw] 13372 out of 13902 Q17 singletons are mated.

[bwa_paired_sw] 869 out of 1432 Q17 discordant pairs are fixed.

[bwa_sai2sam_pe_core] time elapses: 5.37 sec

[bwa_sai2sam_pe_core] refine gapped alignments... 1.00 sec

[bwa_sai2sam_pe_core] print alignments... 2.06 sec

[bwa_sai2sam_pe_core] 49545216 sequences have been processed.

[bwa_sai2sam_pe_core] convert to sequence coordinate...

[infer_isize] (25, 50, 75) percentile: (453, 463, 473)

[infer_isize] low and high boundaries: 413 and 513 for estimating avg and std

[infer_isize] inferred external isize from 201957 pairs: 463.156 +/- 13.968

[infer_isize] skewness: -0.000; kurtosis: -0.128; ap_prior: 1.81e-05

[infer_isize] inferred maximum insert size: 560 (6.96 sigma)

[bwa_sai2sam_pe_core] time elapses: 29.82 sec

[bwa_sai2sam_pe_core] changing coordinates of 6066 alignments.

[bwa_sai2sam_pe_core] align unmapped mate...

[bwa_paired_sw] 13222 out of 13755 Q17 singletons are mated.

[bwa_paired_sw] 937 out of 1493 Q17 discordant pairs are fixed.

[bwa_sai2sam_pe_core] time elapses: 5.35 sec

[bwa_sai2sam_pe_core] refine gapped alignments... 1.02 sec

[bwa_sai2sam_pe_core] print alignments... 2.09 sec

[bwa_sai2sam_pe_core] 49807360 sequences have been processed.

[bwa_sai2sam_pe_core] convert to sequence coordinate...

[infer_isize] (25, 50, 75) percentile: (453, 463, 473)

[infer_isize] low and high boundaries: 413 and 513 for estimating avg and std

[infer_isize] inferred external isize from 201715 pairs: 463.165 +/- 13.909

[infer_isize] skewness: 0.011; kurtosis: -0.142; ap_prior: 1.74e-05

[infer_isize] inferred maximum insert size: 560 (6.96 sigma)

[bwa_sai2sam_pe_core] time elapses: 26.43 sec

[bwa_sai2sam_pe_core] changing coordinates of 6062 alignments.

[bwa_sai2sam_pe_core] align unmapped mate...

[bwa_paired_sw] 13236 out of 13734 Q17 singletons are mated.

[bwa_paired_sw] 859 out of 1414 Q17 discordant pairs are fixed.

[bwa_sai2sam_pe_core] time elapses: 5.18 sec

[bwa_sai2sam_pe_core] refine gapped alignments... 0.97 sec

[bwa_sai2sam_pe_core] print alignments... 2.10 sec

[bwa_sai2sam_pe_core] 50069504 sequences have been processed.

[bwa_sai2sam_pe_core] convert to sequence coordinate...

[infer_isize] (25, 50, 75) percentile: (453, 463, 473)

[infer_isize] low and high boundaries: 413 and 513 for estimating avg and std

[infer_isize] inferred external isize from 201593 pairs: 463.196 +/- 13.934

[infer_isize] skewness: 0.004; kurtosis: -0.130; ap_prior: 1.74e-05

[infer_isize] inferred maximum insert size: 560 (6.96 sigma)

[bwa_sai2sam_pe_core] time elapses: 22.34 sec

[bwa_sai2sam_pe_core] changing coordinates of 5937 alignments.

[bwa_sai2sam_pe_core] align unmapped mate...

[bwa_paired_sw] 13552 out of 14090 Q17 singletons are mated.

[bwa_paired_sw] 876 out of 1436 Q17 discordant pairs are fixed.

[bwa_sai2sam_pe_core] time elapses: 5.29 sec

[bwa_sai2sam_pe_core] refine gapped alignments... 0.98 sec

[bwa_sai2sam_pe_core] print alignments... 2.06 sec

[bwa_sai2sam_pe_core] 50331648 sequences have been processed.

[bwa_sai2sam_pe_core] convert to sequence coordinate...

[infer_isize] (25, 50, 75) percentile: (453, 463, 473)

[infer_isize] low and high boundaries: 413 and 513 for estimating avg and std

[infer_isize] inferred external isize from 201960 pairs: 463.163 +/- 13.948

[infer_isize] skewness: 0.006; kurtosis: -0.134; ap_prior: 1.75e-05

[infer_isize] inferred maximum insert size: 560 (6.96 sigma)

[bwa_sai2sam_pe_core] time elapses: 22.84 sec

[bwa_sai2sam_pe_core] changing coordinates of 5844 alignments.

[bwa_sai2sam_pe_core] align unmapped mate...

[bwa_paired_sw] 13665 out of 14214 Q17 singletons are mated.

[bwa_paired_sw] 871 out of 1411 Q17 discordant pairs are fixed.

[bwa_sai2sam_pe_core] time elapses: 5.32 sec

[bwa_sai2sam_pe_core] refine gapped alignments... 0.99 sec

[bwa_sai2sam_pe_core] print alignments... 2.06 sec

[bwa_sai2sam_pe_core] 50593792 sequences have been processed.

[bwa_sai2sam_pe_core] convert to sequence coordinate...

[infer_isize] (25, 50, 75) percentile: (453, 463, 473)

[infer_isize] low and high boundaries: 413 and 513 for estimating avg and std

[infer_isize] inferred external isize from 201033 pairs: 463.153 +/- 13.940

[infer_isize] skewness: 0.012; kurtosis: -0.128; ap_prior: 1.49e-05

[infer_isize] inferred maximum insert size: 560 (6.96 sigma)

[bwa_sai2sam_pe_core] time elapses: 22.68 sec

[bwa_sai2sam_pe_core] changing coordinates of 5884 alignments.

[bwa_sai2sam_pe_core] align unmapped mate...

[bwa_paired_sw] 14224 out of 14806 Q17 singletons are mated.

[bwa_paired_sw] 838 out of 1418 Q17 discordant pairs are fixed.

[bwa_sai2sam_pe_core] time elapses: 5.46 sec

[bwa_sai2sam_pe_core] refine gapped alignments... 0.99 sec

[bwa_sai2sam_pe_core] print alignments... 2.12 sec

[bwa_sai2sam_pe_core] 50855936 sequences have been processed.

[bwa_sai2sam_pe_core] convert to sequence coordinate...

[infer_isize] (25, 50, 75) percentile: (453, 463, 473)

[infer_isize] low and high boundaries: 413 and 513 for estimating avg and std

[infer_isize] inferred external isize from 200733 pairs: 463.162 +/- 13.927

[infer_isize] skewness: 0.003; kurtosis: -0.140; ap_prior: 1.69e-05

[infer_isize] inferred maximum insert size: 560 (6.96 sigma)

[bwa_sai2sam_pe_core] time elapses: 26.64 sec

[bwa_sai2sam_pe_core] changing coordinates of 5890 alignments.

[bwa_sai2sam_pe_core] align unmapped mate...

[bwa_paired_sw] 14488 out of 15055 Q17 singletons are mated.

[bwa_paired_sw] 851 out of 1416 Q17 discordant pairs are fixed.

[bwa_sai2sam_pe_core] time elapses: 5.56 sec

[bwa_sai2sam_pe_core] refine gapped alignments... 1.05 sec

[bwa_sai2sam_pe_core] print alignments... 2.16 sec

[bwa_sai2sam_pe_core] 51118080 sequences have been processed.

[bwa_sai2sam_pe_core] convert to sequence coordinate...

[infer_isize] (25, 50, 75) percentile: (453, 463, 473)

[infer_isize] low and high boundaries: 413 and 513 for estimating avg and std

[infer_isize] inferred external isize from 200992 pairs: 463.152 +/- 13.971

[infer_isize] skewness: 0.003; kurtosis: -0.126; ap_prior: 1.81e-05

[infer_isize] inferred maximum insert size: 560 (6.96 sigma)

[bwa_sai2sam_pe_core] time elapses: 32.54 sec

[bwa_sai2sam_pe_core] changing coordinates of 5749 alignments.

[bwa_sai2sam_pe_core] align unmapped mate...

[bwa_paired_sw] 14355 out of 14909 Q17 singletons are mated.

[bwa_paired_sw] 875 out of 1433 Q17 discordant pairs are fixed.

[bwa_sai2sam_pe_core] time elapses: 5.70 sec

[bwa_sai2sam_pe_core] refine gapped alignments... 1.09 sec

[bwa_sai2sam_pe_core] print alignments... 2.12 sec

[bwa_sai2sam_pe_core] 51380224 sequences have been processed.

[bwa_sai2sam_pe_core] convert to sequence coordinate...

[infer_isize] (25, 50, 75) percentile: (453, 463, 473)

[infer_isize] low and high boundaries: 413 and 513 for estimating avg and std

[infer_isize] inferred external isize from 200277 pairs: 463.123 +/- 13.965

[infer_isize] skewness: 0.005; kurtosis: -0.133; ap_prior: 1.66e-05

[infer_isize] inferred maximum insert size: 560 (6.96 sigma)

[bwa_sai2sam_pe_core] time elapses: 31.42 sec

[bwa_sai2sam_pe_core] changing coordinates of 5840 alignments.

[bwa_sai2sam_pe_core] align unmapped mate...

[bwa_paired_sw] 14729 out of 15302 Q17 singletons are mated.

[bwa_paired_sw] 852 out of 1447 Q17 discordant pairs are fixed.

[bwa_sai2sam_pe_core] time elapses: 5.86 sec

[bwa_sai2sam_pe_core] refine gapped alignments... 1.08 sec

[bwa_sai2sam_pe_core] print alignments... 2.10 sec

[bwa_sai2sam_pe_core] 51642368 sequences have been processed.

[bwa_sai2sam_pe_core] convert to sequence coordinate...

[infer_isize] (25, 50, 75) percentile: (453, 463, 473)

[infer_isize] low and high boundaries: 413 and 513 for estimating avg and std

[infer_isize] inferred external isize from 200098 pairs: 463.142 +/- 13.918

[infer_isize] skewness: 0.008; kurtosis: -0.117; ap_prior: 1.69e-05

[infer_isize] inferred maximum insert size: 560 (6.96 sigma)

[bwa_sai2sam_pe_core] time elapses: 30.59 sec

[bwa_sai2sam_pe_core] changing coordinates of 5924 alignments.

[bwa_sai2sam_pe_core] align unmapped mate...

[bwa_paired_sw] 15108 out of 15667 Q17 singletons are mated.

[bwa_paired_sw] 897 out of 1458 Q17 discordant pairs are fixed.

[bwa_sai2sam_pe_core] time elapses: 5.71 sec

[bwa_sai2sam_pe_core] refine gapped alignments... 1.03 sec

[bwa_sai2sam_pe_core] print alignments... 2.09 sec

[bwa_sai2sam_pe_core] 51904512 sequences have been processed.

[bwa_sai2sam_pe_core] convert to sequence coordinate...

[infer_isize] (25, 50, 75) percentile: (453, 463, 473)

[infer_isize] low and high boundaries: 413 and 513 for estimating avg and std

[infer_isize] inferred external isize from 199734 pairs: 463.155 +/- 13.977

[infer_isize] skewness: -0.001; kurtosis: -0.142; ap_prior: 1.66e-05

[infer_isize] inferred maximum insert size: 560 (6.96 sigma)

[bwa_sai2sam_pe_core] time elapses: 28.55 sec

[bwa_sai2sam_pe_core] changing coordinates of 5870 alignments.

[bwa_sai2sam_pe_core] align unmapped mate...

[bwa_paired_sw] 15316 out of 15891 Q17 singletons are mated.

[bwa_paired_sw] 848 out of 1367 Q17 discordant pairs are fixed.

[bwa_sai2sam_pe_core] time elapses: 5.74 sec

[bwa_sai2sam_pe_core] refine gapped alignments... 1.03 sec

[bwa_sai2sam_pe_core] print alignments... 2.09 sec

[bwa_sai2sam_pe_core] 52166656 sequences have been processed.

[bwa_sai2sam_pe_core] convert to sequence coordinate...

[infer_isize] (25, 50, 75) percentile: (453, 463, 473)

[infer_isize] low and high boundaries: 413 and 513 for estimating avg and std

[infer_isize] inferred external isize from 199401 pairs: 463.045 +/- 13.949

[infer_isize] skewness: -0.006; kurtosis: -0.127; ap_prior: 1.70e-05

[infer_isize] inferred maximum insert size: 560 (6.96 sigma)

[bwa_sai2sam_pe_core] time elapses: 28.06 sec

[bwa_sai2sam_pe_core] changing coordinates of 5796 alignments.

[bwa_sai2sam_pe_core] align unmapped mate...

[bwa_paired_sw] 15530 out of 16037 Q17 singletons are mated.

[bwa_paired_sw] 810 out of 1362 Q17 discordant pairs are fixed.

[bwa_sai2sam_pe_core] time elapses: 5.81 sec

[bwa_sai2sam_pe_core] refine gapped alignments... 1.07 sec

[bwa_sai2sam_pe_core] print alignments... 2.10 sec

[bwa_sai2sam_pe_core] 52428800 sequences have been processed.

[bwa_sai2sam_pe_core] convert to sequence coordinate...

[infer_isize] (25, 50, 75) percentile: (453, 463, 473)

[infer_isize] low and high boundaries: 413 and 513 for estimating avg and std

[infer_isize] inferred external isize from 199234 pairs: 463.095 +/- 13.987

[infer_isize] skewness: 0.006; kurtosis: -0.130; ap_prior: 1.67e-05

[infer_isize] inferred maximum insert size: 560 (6.96 sigma)

[bwa_sai2sam_pe_core] time elapses: 28.06 sec

[bwa_sai2sam_pe_core] changing coordinates of 5875 alignments.

[bwa_sai2sam_pe_core] align unmapped mate...

[bwa_paired_sw] 15826 out of 16402 Q17 singletons are mated.

[bwa_paired_sw] 845 out of 1424 Q17 discordant pairs are fixed.

[bwa_sai2sam_pe_core] time elapses: 5.89 sec

[bwa_sai2sam_pe_core] refine gapped alignments... 1.06 sec

[bwa_sai2sam_pe_core] print alignments... 2.08 sec

[bwa_sai2sam_pe_core] 52690944 sequences have been processed.

[bwa_sai2sam_pe_core] convert to sequence coordinate...

[infer_isize] (25, 50, 75) percentile: (453, 463, 473)

[infer_isize] low and high boundaries: 413 and 513 for estimating avg and std

[infer_isize] inferred external isize from 198594 pairs: 463.083 +/- 13.900

[infer_isize] skewness: -0.001; kurtosis: -0.113; ap_prior: 1.75e-05

[infer_isize] inferred maximum insert size: 560 (6.96 sigma)

[bwa_sai2sam_pe_core] time elapses: 24.97 sec

[bwa_sai2sam_pe_core] changing coordinates of 5868 alignments.

[bwa_sai2sam_pe_core] align unmapped mate...

[bwa_paired_sw] 16239 out of 16806 Q17 singletons are mated.

[bwa_paired_sw] 865 out of 1409 Q17 discordant pairs are fixed.

[bwa_sai2sam_pe_core] time elapses: 6.02 sec

[bwa_sai2sam_pe_core] refine gapped alignments... 1.07 sec

[bwa_sai2sam_pe_core] print alignments... 2.09 sec

[bwa_sai2sam_pe_core] 52953088 sequences have been processed.

[bwa_sai2sam_pe_core] convert to sequence coordinate...

[infer_isize] (25, 50, 75) percentile: (453, 463, 473)

[infer_isize] low and high boundaries: 413 and 513 for estimating avg and std

[infer_isize] inferred external isize from 197877 pairs: 463.067 +/- 13.955

[infer_isize] skewness: 0.009; kurtosis: -0.139; ap_prior: 1.55e-05

[infer_isize] inferred maximum insert size: 560 (6.96 sigma)

[bwa_sai2sam_pe_core] time elapses: 25.12 sec

[bwa_sai2sam_pe_core] changing coordinates of 5910 alignments.

[bwa_sai2sam_pe_core] align unmapped mate...

[bwa_paired_sw] 16843 out of 17358 Q17 singletons are mated.

[bwa_paired_sw] 897 out of 1378 Q17 discordant pairs are fixed.

[bwa_sai2sam_pe_core] time elapses: 6.17 sec

[bwa_sai2sam_pe_core] refine gapped alignments... 1.07 sec

[bwa_sai2sam_pe_core] print alignments... 2.09 sec

[bwa_sai2sam_pe_core] 53215232 sequences have been processed.

[bwa_sai2sam_pe_core] convert to sequence coordinate...

[infer_isize] (25, 50, 75) percentile: (453, 463, 473)

[infer_isize] low and high boundaries: 413 and 513 for estimating avg and std

[infer_isize] inferred external isize from 197687 pairs: 463.052 +/- 13.945

[infer_isize] skewness: 0.003; kurtosis: -0.122; ap_prior: 1.75e-05

[infer_isize] inferred maximum insert size: 560 (6.96 sigma)

[bwa_sai2sam_pe_core] time elapses: 24.67 sec

[bwa_sai2sam_pe_core] changing coordinates of 5805 alignments.

[bwa_sai2sam_pe_core] align unmapped mate...

[bwa_paired_sw] 17217 out of 17787 Q17 singletons are mated.

[bwa_paired_sw] 827 out of 1346 Q17 discordant pairs are fixed.

[bwa_sai2sam_pe_core] time elapses: 6.26 sec

[bwa_sai2sam_pe_core] refine gapped alignments... 1.10 sec

[bwa_sai2sam_pe_core] print alignments... 2.09 sec

[bwa_sai2sam_pe_core] 53477376 sequences have been processed.

[bwa_sai2sam_pe_core] convert to sequence coordinate...

[infer_isize] (25, 50, 75) percentile: (453, 463, 473)

[infer_isize] low and high boundaries: 413 and 513 for estimating avg and std

[infer_isize] inferred external isize from 196768 pairs: 463.041 +/- 13.974

[infer_isize] skewness: 0.010; kurtosis: -0.138; ap_prior: 1.64e-05

[infer_isize] inferred maximum insert size: 560 (6.96 sigma)

[bwa_sai2sam_pe_core] time elapses: 29.57 sec

[bwa_sai2sam_pe_core] changing coordinates of 5720 alignments.

[bwa_sai2sam_pe_core] align unmapped mate...

[bwa_paired_sw] 17996 out of 18588 Q17 singletons are mated.

[bwa_paired_sw] 831 out of 1387 Q17 discordant pairs are fixed.

[bwa_sai2sam_pe_core] time elapses: 6.65 sec

[bwa_sai2sam_pe_core] refine gapped alignments... 1.15 sec

[bwa_sai2sam_pe_core] print alignments... 2.07 sec

[bwa_sai2sam_pe_core] 53739520 sequences have been processed.

[bwa_sai2sam_pe_core] convert to sequence coordinate...

[infer_isize] (25, 50, 75) percentile: (453, 463, 473)

[infer_isize] low and high boundaries: 413 and 513 for estimating avg and std

[infer_isize] inferred external isize from 197052 pairs: 463.110 +/- 13.959

[infer_isize] skewness: 0.006; kurtosis: -0.126; ap_prior: 1.55e-05

[infer_isize] inferred maximum insert size: 560 (6.96 sigma)

[bwa_sai2sam_pe_core] time elapses: 29.38 sec

[bwa_sai2sam_pe_core] changing coordinates of 5581 alignments.

[bwa_sai2sam_pe_core] align unmapped mate...

[bwa_paired_sw] 18352 out of 18860 Q17 singletons are mated.

[bwa_paired_sw] 833 out of 1373 Q17 discordant pairs are fixed.

[bwa_sai2sam_pe_core] time elapses: 6.55 sec

[bwa_sai2sam_pe_core] refine gapped alignments... 1.13 sec

[bwa_sai2sam_pe_core] print alignments... 2.17 sec

[bwa_sai2sam_pe_core] 54001664 sequences have been processed.

[bwa_sai2sam_pe_core] convert to sequence coordinate...

[infer_isize] (25, 50, 75) percentile: (453, 463, 473)

[infer_isize] low and high boundaries: 413 and 513 for estimating avg and std

[infer_isize] inferred external isize from 195830 pairs: 463.030 +/- 13.967

[infer_isize] skewness: 0.007; kurtosis: -0.124; ap_prior: 1.74e-05

[infer_isize] inferred maximum insert size: 560 (6.96 sigma)

[bwa_sai2sam_pe_core] time elapses: 32.14 sec

[bwa_sai2sam_pe_core] changing coordinates of 5774 alignments.

[bwa_sai2sam_pe_core] align unmapped mate...

[bwa_paired_sw] 19027 out of 19589 Q17 singletons are mated.

[bwa_paired_sw] 891 out of 1429 Q17 discordant pairs are fixed.

[bwa_sai2sam_pe_core] time elapses: 7.11 sec

[bwa_sai2sam_pe_core] refine gapped alignments... 1.32 sec

[bwa_sai2sam_pe_core] print alignments... 2.30 sec

[bwa_sai2sam_pe_core] 54263808 sequences have been processed.

[bwa_sai2sam_pe_core] convert to sequence coordinate...

[infer_isize] (25, 50, 75) percentile: (453, 463, 473)

[infer_isize] low and high boundaries: 413 and 513 for estimating avg and std

[infer_isize] inferred external isize from 194439 pairs: 463.038 +/- 13.962

[infer_isize] skewness: 0.003; kurtosis: -0.132; ap_prior: 1.83e-05

[infer_isize] inferred maximum insert size: 560 (6.96 sigma)

[bwa_sai2sam_pe_core] time elapses: 36.96 sec

[bwa_sai2sam_pe_core] changing coordinates of 5805 alignments.

[bwa_sai2sam_pe_core] align unmapped mate...

[bwa_paired_sw] 20173 out of 20754 Q17 singletons are mated.

[bwa_paired_sw] 945 out of 1467 Q17 discordant pairs are fixed.

[bwa_sai2sam_pe_core] time elapses: 7.32 sec

[bwa_sai2sam_pe_core] refine gapped alignments... 1.31 sec

[bwa_sai2sam_pe_core] print alignments... 2.20 sec

[bwa_sai2sam_pe_core] 54525952 sequences have been processed.

[bwa_sai2sam_pe_core] convert to sequence coordinate...

[infer_isize] (25, 50, 75) percentile: (453, 463, 473)

[infer_isize] low and high boundaries: 413 and 513 for estimating avg and std

[infer_isize] inferred external isize from 193986 pairs: 463.099 +/- 13.902

[infer_isize] skewness: 0.000; kurtosis: -0.121; ap_prior: 1.76e-05

[infer_isize] inferred maximum insert size: 560 (6.96 sigma)

[bwa_sai2sam_pe_core] time elapses: 32.04 sec

[bwa_sai2sam_pe_core] changing coordinates of 5723 alignments.

[bwa_sai2sam_pe_core] align unmapped mate...

[bwa_paired_sw] 20712 out of 21286 Q17 singletons are mated.

[bwa_paired_sw] 863 out of 1378 Q17 discordant pairs are fixed.

[bwa_sai2sam_pe_core] time elapses: 7.36 sec

[bwa_sai2sam_pe_core] refine gapped alignments... 1.25 sec

[bwa_sai2sam_pe_core] print alignments... 2.08 sec

[bwa_sai2sam_pe_core] 54788096 sequences have been processed.

[bwa_sai2sam_pe_core] convert to sequence coordinate...

[infer_isize] (25, 50, 75) percentile: (453, 463, 473)

[infer_isize] low and high boundaries: 413 and 513 for estimating avg and std

[infer_isize] inferred external isize from 193524 pairs: 462.987 +/- 13.938

[infer_isize] skewness: 0.006; kurtosis: -0.140; ap_prior: 1.66e-05

[infer_isize] inferred maximum insert size: 560 (6.96 sigma)

[bwa_sai2sam_pe_core] time elapses: 31.11 sec

[bwa_sai2sam_pe_core] changing coordinates of 5601 alignments.

[bwa_sai2sam_pe_core] align unmapped mate...

[bwa_paired_sw] 21293 out of 21901 Q17 singletons are mated.

[bwa_paired_sw] 892 out of 1402 Q17 discordant pairs are fixed.

[bwa_sai2sam_pe_core] time elapses: 7.42 sec

[bwa_sai2sam_pe_core] refine gapped alignments... 1.25 sec

[bwa_sai2sam_pe_core] print alignments... 2.11 sec

[bwa_sai2sam_pe_core] 55050240 sequences have been processed.

[bwa_sai2sam_pe_core] convert to sequence coordinate...

[infer_isize] (25, 50, 75) percentile: (453, 463, 473)

[infer_isize] low and high boundaries: 413 and 513 for estimating avg and std

[infer_isize] inferred external isize from 192864 pairs: 462.998 +/- 13.934

[infer_isize] skewness: 0.006; kurtosis: -0.152; ap_prior: 1.54e-05

[infer_isize] inferred maximum insert size: 560 (6.96 sigma)

[bwa_sai2sam_pe_core] time elapses: 26.31 sec

[bwa_sai2sam_pe_core] changing coordinates of 5572 alignments.

[bwa_sai2sam_pe_core] align unmapped mate...

[bwa_paired_sw] 21757 out of 22312 Q17 singletons are mated.

[bwa_paired_sw] 884 out of 1392 Q17 discordant pairs are fixed.

[bwa_sai2sam_pe_core] time elapses: 7.52 sec

[bwa_sai2sam_pe_core] refine gapped alignments... 1.27 sec

[bwa_sai2sam_pe_core] print alignments... 2.10 sec

[bwa_sai2sam_pe_core] 55312384 sequences have been processed.

[bwa_sai2sam_pe_core] convert to sequence coordinate...

[infer_isize] (25, 50, 75) percentile: (453, 463, 473)

[infer_isize] low and high boundaries: 413 and 513 for estimating avg and std

[infer_isize] inferred external isize from 192455 pairs: 463.041 +/- 13.925

[infer_isize] skewness: 0.000; kurtosis: -0.146; ap_prior: 1.65e-05

[infer_isize] inferred maximum insert size: 560 (6.96 sigma)

[bwa_sai2sam_pe_core] time elapses: 25.87 sec

[bwa_sai2sam_pe_core] changing coordinates of 5539 alignments.

[bwa_sai2sam_pe_core] align unmapped mate...

[bwa_paired_sw] 22229 out of 22795 Q17 singletons are mated.

[bwa_paired_sw] 851 out of 1372 Q17 discordant pairs are fixed.

[bwa_sai2sam_pe_core] time elapses: 7.64 sec

[bwa_sai2sam_pe_core] refine gapped alignments... 1.29 sec

[bwa_sai2sam_pe_core] print alignments... 2.09 sec

[bwa_sai2sam_pe_core] 55574528 sequences have been processed.

[bwa_sai2sam_pe_core] convert to sequence coordinate...

[infer_isize] (25, 50, 75) percentile: (453, 463, 473)

[infer_isize] low and high boundaries: 413 and 513 for estimating avg and std

[infer_isize] inferred external isize from 191325 pairs: 462.985 +/- 13.923

[infer_isize] skewness: 0.011; kurtosis: -0.137; ap_prior: 1.81e-05

[infer_isize] inferred maximum insert size: 560 (6.96 sigma)

[bwa_sai2sam_pe_core] time elapses: 24.50 sec

[bwa_sai2sam_pe_core] changing coordinates of 5623 alignments.

[bwa_sai2sam_pe_core] align unmapped mate...

[bwa_paired_sw] 23401 out of 23950 Q17 singletons are mated.

[bwa_paired_sw] 891 out of 1377 Q17 discordant pairs are fixed.

[bwa_sai2sam_pe_core] time elapses: 7.95 sec

[bwa_sai2sam_pe_core] refine gapped alignments... 1.30 sec

[bwa_sai2sam_pe_core] print alignments... 2.07 sec

[bwa_sai2sam_pe_core] 55836672 sequences have been processed.

[bwa_sai2sam_pe_core] convert to sequence coordinate...

[infer_isize] (25, 50, 75) percentile: (453, 463, 473)

[infer_isize] low and high boundaries: 413 and 513 for estimating avg and std

[infer_isize] inferred external isize from 190533 pairs: 462.971 +/- 13.932

[infer_isize] skewness: -0.003; kurtosis: -0.132; ap_prior: 1.68e-05

[infer_isize] inferred maximum insert size: 560 (6.96 sigma)

[bwa_sai2sam_pe_core] time elapses: 24.28 sec

[bwa_sai2sam_pe_core] changing coordinates of 5745 alignments.

[bwa_sai2sam_pe_core] align unmapped mate...

[bwa_paired_sw] 23801 out of 24403 Q17 singletons are mated.

[bwa_paired_sw] 903 out of 1383 Q17 discordant pairs are fixed.

[bwa_sai2sam_pe_core] time elapses: 8.05 sec

[bwa_sai2sam_pe_core] refine gapped alignments... 1.35 sec

[bwa_sai2sam_pe_core] print alignments... 2.09 sec

[bwa_sai2sam_pe_core] 56098816 sequences have been processed.

[bwa_sai2sam_pe_core] convert to sequence coordinate...

[infer_isize] (25, 50, 75) percentile: (453, 463, 473)

[infer_isize] low and high boundaries: 413 and 513 for estimating avg and std

[infer_isize] inferred external isize from 190172 pairs: 462.927 +/- 13.927

[infer_isize] skewness: 0.006; kurtosis: -0.118; ap_prior: 1.65e-05

[infer_isize] inferred maximum insert size: 560 (6.96 sigma)

[bwa_sai2sam_pe_core] time elapses: 19.11 sec

[bwa_sai2sam_pe_core] changing coordinates of 5691 alignments.

[bwa_sai2sam_pe_core] align unmapped mate...

[bwa_paired_sw] 24305 out of 24896 Q17 singletons are mated.

[bwa_paired_sw] 835 out of 1332 Q17 discordant pairs are fixed.

[bwa_sai2sam_pe_core] time elapses: 8.16 sec

[bwa_sai2sam_pe_core] refine gapped alignments... 1.38 sec

[bwa_sai2sam_pe_core] print alignments... 2.10 sec

[bwa_sai2sam_pe_core] 56360960 sequences have been processed.

[bwa_sai2sam_pe_core] convert to sequence coordinate...

[infer_isize] (25, 50, 75) percentile: (453, 463, 473)

[infer_isize] low and high boundaries: 413 and 513 for estimating avg and std

[infer_isize] inferred external isize from 189181 pairs: 462.969 +/- 13.940

[infer_isize] skewness: 0.006; kurtosis: -0.133; ap_prior: 1.69e-05

[infer_isize] inferred maximum insert size: 560 (6.96 sigma)

[bwa_sai2sam_pe_core] time elapses: 22.93 sec

[bwa_sai2sam_pe_core] changing coordinates of 5445 alignments.

[bwa_sai2sam_pe_core] align unmapped mate...

[bwa_paired_sw] 25479 out of 26048 Q17 singletons are mated.

[bwa_paired_sw] 911 out of 1403 Q17 discordant pairs are fixed.

[bwa_sai2sam_pe_core] time elapses: 8.53 sec

[bwa_sai2sam_pe_core] refine gapped alignments... 1.43 sec

[bwa_sai2sam_pe_core] print alignments... 2.07 sec

[bwa_sai2sam_pe_core] 56623104 sequences have been processed.

[bwa_sai2sam_pe_core] convert to sequence coordinate...

[infer_isize] (25, 50, 75) percentile: (453, 463, 473)

[infer_isize] low and high boundaries: 413 and 513 for estimating avg and std

[infer_isize] inferred external isize from 187812 pairs: 463.025 +/- 13.923

[infer_isize] skewness: 0.007; kurtosis: -0.146; ap_prior: 1.77e-05

[infer_isize] inferred maximum insert size: 560 (6.96 sigma)

[bwa_sai2sam_pe_core] time elapses: 23.37 sec

[bwa_sai2sam_pe_core] changing coordinates of 5296 alignments.

[bwa_sai2sam_pe_core] align unmapped mate...

[bwa_paired_sw] 26775 out of 27368 Q17 singletons are mated.

[bwa_paired_sw] 859 out of 1363 Q17 discordant pairs are fixed.

[bwa_sai2sam_pe_core] time elapses: 8.87 sec

[bwa_sai2sam_pe_core] refine gapped alignments... 1.44 sec

[bwa_sai2sam_pe_core] print alignments... 2.06 sec

[bwa_sai2sam_pe_core] 56885248 sequences have been processed.

[bwa_sai2sam_pe_core] convert to sequence coordinate...

[infer_isize] (25, 50, 75) percentile: (453, 463, 473)

[infer_isize] low and high boundaries: 413 and 513 for estimating avg and std

[infer_isize] inferred external isize from 186381 pairs: 462.911 +/- 13.959

[infer_isize] skewness: 0.012; kurtosis: -0.132; ap_prior: 1.50e-05

[infer_isize] inferred maximum insert size: 560 (6.96 sigma)

[bwa_sai2sam_pe_core] time elapses: 23.26 sec

[bwa_sai2sam_pe_core] changing coordinates of 5336 alignments.

[bwa_sai2sam_pe_core] align unmapped mate...

[bwa_paired_sw] 28072 out of 28679 Q17 singletons are mated.

[bwa_paired_sw] 909 out of 1362 Q17 discordant pairs are fixed.

[bwa_sai2sam_pe_core] time elapses: 9.23 sec

[bwa_sai2sam_pe_core] refine gapped alignments... 1.47 sec

[bwa_sai2sam_pe_core] print alignments... 2.09 sec

[bwa_sai2sam_pe_core] 57147392 sequences have been processed.

[bwa_sai2sam_pe_core] convert to sequence coordinate...

[infer_isize] (25, 50, 75) percentile: (453, 463, 473)

[infer_isize] low and high boundaries: 413 and 513 for estimating avg and std

[infer_isize] inferred external isize from 186314 pairs: 462.842 +/- 13.949

[infer_isize] skewness: 0.011; kurtosis: -0.134; ap_prior: 1.72e-05

[infer_isize] inferred maximum insert size: 560 (6.96 sigma)

[bwa_sai2sam_pe_core] time elapses: 29.92 sec

[bwa_sai2sam_pe_core] changing coordinates of 5495 alignments.

[bwa_sai2sam_pe_core] align unmapped mate...

[bwa_paired_sw] 27699 out of 28345 Q17 singletons are mated.

[bwa_paired_sw] 889 out of 1314 Q17 discordant pairs are fixed.

[bwa_sai2sam_pe_core] time elapses: 9.39 sec

[bwa_sai2sam_pe_core] refine gapped alignments... 1.65 sec

[bwa_sai2sam_pe_core] print alignments... 2.16 sec

[bwa_sai2sam_pe_core] 57409536 sequences have been processed.

[bwa_sai2sam_pe_core] convert to sequence coordinate...

[infer_isize] (25, 50, 75) percentile: (453, 463, 473)

[infer_isize] low and high boundaries: 413 and 513 for estimating avg and std

[infer_isize] inferred external isize from 186427 pairs: 462.873 +/- 13.944

[infer_isize] skewness: 0.012; kurtosis: -0.131; ap_prior: 1.69e-05

[infer_isize] inferred maximum insert size: 560 (6.96 sigma)

[bwa_sai2sam_pe_core] time elapses: 29.04 sec

[bwa_sai2sam_pe_core] changing coordinates of 5422 alignments.

[bwa_sai2sam_pe_core] align unmapped mate...

[bwa_paired_sw] 27908 out of 28520 Q17 singletons are mated.

[bwa_paired_sw] 919 out of 1412 Q17 discordant pairs are fixed.

[bwa_sai2sam_pe_core] time elapses: 9.36 sec

[bwa_sai2sam_pe_core] refine gapped alignments... 1.59 sec

[bwa_sai2sam_pe_core] print alignments... 2.14 sec

[bwa_sai2sam_pe_core] 57671680 sequences have been processed.

[bwa_sai2sam_pe_core] convert to sequence coordinate...

[infer_isize] (25, 50, 75) percentile: (453, 463, 473)

[infer_isize] low and high boundaries: 413 and 513 for estimating avg and std

[infer_isize] inferred external isize from 189214 pairs: 462.990 +/- 13.963

[infer_isize] skewness: 0.007; kurtosis: -0.129; ap_prior: 1.59e-05

[infer_isize] inferred maximum insert size: 560 (6.96 sigma)

[bwa_sai2sam_pe_core] time elapses: 28.32 sec

[bwa_sai2sam_pe_core] changing coordinates of 5519 alignments.

[bwa_sai2sam_pe_core] align unmapped mate...

[bwa_paired_sw] 24561 out of 25173 Q17 singletons are mated.

[bwa_paired_sw] 886 out of 1411 Q17 discordant pairs are fixed.

[bwa_sai2sam_pe_core] time elapses: 8.41 sec

[bwa_sai2sam_pe_core] refine gapped alignments... 1.35 sec

[bwa_sai2sam_pe_core] print alignments... 2.05 sec

[bwa_sai2sam_pe_core] 57933824 sequences have been processed.

[bwa_sai2sam_pe_core] convert to sequence coordinate...

[infer_isize] (25, 50, 75) percentile: (453, 463, 473)

[infer_isize] low and high boundaries: 413 and 513 for estimating avg and std

[infer_isize] inferred external isize from 206094 pairs: 463.320 +/- 13.935

[infer_isize] skewness: -0.010; kurtosis: -0.138; ap_prior: 1.73e-05

[infer_isize] inferred maximum insert size: 560 (6.96 sigma)

[bwa_sai2sam_pe_core] time elapses: 31.57 sec

[bwa_sai2sam_pe_core] changing coordinates of 6090 alignments.

[bwa_sai2sam_pe_core] align unmapped mate...

[bwa_paired_sw] 9436 out of 10018 Q17 singletons are mated.

[bwa_paired_sw] 837 out of 1461 Q17 discordant pairs are fixed.

[bwa_sai2sam_pe_core] time elapses: 4.32 sec

[bwa_sai2sam_pe_core] refine gapped alignments... 0.91 sec

[bwa_sai2sam_pe_core] print alignments... 2.12 sec

[bwa_sai2sam_pe_core] 58195968 sequences have been processed.

[bwa_sai2sam_pe_core] convert to sequence coordinate...

[infer_isize] (25, 50, 75) percentile: (453, 463, 473)

[infer_isize] low and high boundaries: 413 and 513 for estimating avg and std

[infer_isize] inferred external isize from 206674 pairs: 463.247 +/- 13.951

[infer_isize] skewness: 0.007; kurtosis: -0.146; ap_prior: 1.77e-05

[infer_isize] inferred maximum insert size: 560 (6.96 sigma)

[bwa_sai2sam_pe_core] time elapses: 30.58 sec

[bwa_sai2sam_pe_core] changing coordinates of 5962 alignments.

[bwa_sai2sam_pe_core] align unmapped mate...

[bwa_paired_sw] 9417 out of 9972 Q17 singletons are mated.

[bwa_paired_sw] 875 out of 1504 Q17 discordant pairs are fixed.

[bwa_sai2sam_pe_core] time elapses: 4.34 sec

[bwa_sai2sam_pe_core] refine gapped alignments... 0.91 sec

[bwa_sai2sam_pe_core] print alignments... 2.09 sec

[bwa_sai2sam_pe_core] 58458112 sequences have been processed.

[bwa_sai2sam_pe_core] convert to sequence coordinate...

[infer_isize] (25, 50, 75) percentile: (453, 463, 473)

[infer_isize] low and high boundaries: 413 and 513 for estimating avg and std

[infer_isize] inferred external isize from 206798 pairs: 463.353 +/- 13.966

[infer_isize] skewness: -0.001; kurtosis: -0.140; ap_prior: 1.76e-05

[infer_isize] inferred maximum insert size: 561 (6.96 sigma)

[bwa_sai2sam_pe_core] time elapses: 30.81 sec

[bwa_sai2sam_pe_core] changing coordinates of 6121 alignments.

[bwa_sai2sam_pe_core] align unmapped mate...

[bwa_paired_sw] 9046 out of 9559 Q17 singletons are mated.

[bwa_paired_sw] 843 out of 1441 Q17 discordant pairs are fixed.

[bwa_sai2sam_pe_core] time elapses: 4.20 sec

[bwa_sai2sam_pe_core] refine gapped alignments... 0.92 sec

[bwa_sai2sam_pe_core] print alignments... 2.08 sec

[bwa_sai2sam_pe_core] 58720256 sequences have been processed.

[bwa_sai2sam_pe_core] convert to sequence coordinate...

[infer_isize] (25, 50, 75) percentile: (453, 463, 473)

[infer_isize] low and high boundaries: 413 and 513 for estimating avg and std

[infer_isize] inferred external isize from 206425 pairs: 463.373 +/- 13.949

[infer_isize] skewness: 0.000; kurtosis: -0.129; ap_prior: 1.82e-05

[infer_isize] inferred maximum insert size: 560 (6.96 sigma)

[bwa_sai2sam_pe_core] time elapses: 31.43 sec

[bwa_sai2sam_pe_core] changing coordinates of 6198 alignments.

[bwa_sai2sam_pe_core] align unmapped mate...

[bwa_paired_sw] 9210 out of 9781 Q17 singletons are mated.

[bwa_paired_sw] 931 out of 1553 Q17 discordant pairs are fixed.

[bwa_sai2sam_pe_core] time elapses: 4.34 sec

[bwa_sai2sam_pe_core] refine gapped alignments... 0.92 sec

[bwa_sai2sam_pe_core] print alignments... 2.11 sec

[bwa_sai2sam_pe_core] 58982400 sequences have been processed.

[bwa_sai2sam_pe_core] convert to sequence coordinate...

[infer_isize] (25, 50, 75) percentile: (453, 463, 473)

[infer_isize] low and high boundaries: 413 and 513 for estimating avg and std

[infer_isize] inferred external isize from 207033 pairs: 463.348 +/- 13.951

[infer_isize] skewness: 0.007; kurtosis: -0.158; ap_prior: 1.76e-05

[infer_isize] inferred maximum insert size: 560 (6.96 sigma)

[bwa_sai2sam_pe_core] time elapses: 30.70 sec

[bwa_sai2sam_pe_core] changing coordinates of 6029 alignments.

[bwa_sai2sam_pe_core] align unmapped mate...

[bwa_paired_sw] 8830 out of 9406 Q17 singletons are mated.

[bwa_paired_sw] 924 out of 1507 Q17 discordant pairs are fixed.

[bwa_sai2sam_pe_core] time elapses: 4.04 sec

[bwa_sai2sam_pe_core] refine gapped alignments... 0.88 sec

[bwa_sai2sam_pe_core] print alignments... 2.10 sec

[bwa_sai2sam_pe_core] 59244544 sequences have been processed.

[bwa_sai2sam_pe_core] convert to sequence coordinate...

[infer_isize] (25, 50, 75) percentile: (453, 463, 473)

[infer_isize] low and high boundaries: 413 and 513 for estimating avg and std

[infer_isize] inferred external isize from 207058 pairs: 463.309 +/- 13.920

[infer_isize] skewness: 0.008; kurtosis: -0.145; ap_prior: 1.64e-05

[infer_isize] inferred maximum insert size: 560 (6.96 sigma)

[bwa_sai2sam_pe_core] time elapses: 24.01 sec

[bwa_sai2sam_pe_core] changing coordinates of 5983 alignments.

[bwa_sai2sam_pe_core] align unmapped mate...

[bwa_paired_sw] 9022 out of 9579 Q17 singletons are mated.

[bwa_paired_sw] 902 out of 1540 Q17 discordant pairs are fixed.

[bwa_sai2sam_pe_core] time elapses: 4.11 sec

[bwa_sai2sam_pe_core] refine gapped alignments... 0.87 sec

[bwa_sai2sam_pe_core] print alignments... 2.06 sec

[bwa_sai2sam_pe_core] 59506688 sequences have been processed.

[bwa_sai2sam_pe_core] convert to sequence coordinate...

[infer_isize] (25, 50, 75) percentile: (453, 463, 473)

[infer_isize] low and high boundaries: 413 and 513 for estimating avg and std

[infer_isize] inferred external isize from 206983 pairs: 463.307 +/- 13.925

[infer_isize] skewness: -0.004; kurtosis: -0.129; ap_prior: 1.86e-05

[infer_isize] inferred maximum insert size: 560 (6.96 sigma)

[bwa_sai2sam_pe_core] time elapses: 24.15 sec

[bwa_sai2sam_pe_core] changing coordinates of 6028 alignments.

[bwa_sai2sam_pe_core] align unmapped mate...

[bwa_paired_sw] 9021 out of 9590 Q17 singletons are mated.

[bwa_paired_sw] 911 out of 1476 Q17 discordant pairs are fixed.

[bwa_sai2sam_pe_core] time elapses: 4.10 sec

[bwa_sai2sam_pe_core] refine gapped alignments... 0.87 sec

[bwa_sai2sam_pe_core] print alignments... 2.05 sec

[bwa_sai2sam_pe_core] 59768832 sequences have been processed.

[bwa_sai2sam_pe_core] convert to sequence coordinate...

[infer_isize] (25, 50, 75) percentile: (453, 463, 473)

[infer_isize] low and high boundaries: 413 and 513 for estimating avg and std

[infer_isize] inferred external isize from 207221 pairs: 463.361 +/- 13.961

[infer_isize] skewness: 0.001; kurtosis: -0.132; ap_prior: 1.73e-05

[infer_isize] inferred maximum insert size: 561 (6.96 sigma)

[bwa_sai2sam_pe_core] time elapses: 24.14 sec

[bwa_sai2sam_pe_core] changing coordinates of 5945 alignments.

[bwa_sai2sam_pe_core] align unmapped mate...

[bwa_paired_sw] 8755 out of 9306 Q17 singletons are mated.

[bwa_paired_sw] 893 out of 1505 Q17 discordant pairs are fixed.

[bwa_sai2sam_pe_core] time elapses: 4.01 sec

[bwa_sai2sam_pe_core] refine gapped alignments... 0.88 sec

[bwa_sai2sam_pe_core] print alignments... 2.09 sec

[bwa_sai2sam_pe_core] 60030976 sequences have been processed.

[bwa_sai2sam_pe_core] convert to sequence coordinate...

[infer_isize] (25, 50, 75) percentile: (453, 463, 473)

[infer_isize] low and high boundaries: 413 and 513 for estimating avg and std

[infer_isize] inferred external isize from 207095 pairs: 463.362 +/- 13.948

[infer_isize] skewness: 0.003; kurtosis: -0.143; ap_prior: 1.78e-05

[infer_isize] inferred maximum insert size: 560 (6.96 sigma)

[bwa_sai2sam_pe_core] time elapses: 31.69 sec

[bwa_sai2sam_pe_core] changing coordinates of 6097 alignments.

[bwa_sai2sam_pe_core] align unmapped mate...

[bwa_paired_sw] 8579 out of 9134 Q17 singletons are mated.

[bwa_paired_sw] 931 out of 1556 Q17 discordant pairs are fixed.

[bwa_sai2sam_pe_core] time elapses: 4.13 sec

[bwa_sai2sam_pe_core] refine gapped alignments... 0.92 sec

[bwa_sai2sam_pe_core] print alignments... 2.06 sec

[bwa_sai2sam_pe_core] 60293120 sequences have been processed.

[bwa_sai2sam_pe_core] convert to sequence coordinate...

[infer_isize] (25, 50, 75) percentile: (453, 463, 473)

[infer_isize] low and high boundaries: 413 and 513 for estimating avg and std

[infer_isize] inferred external isize from 207118 pairs: 463.321 +/- 13.943

[infer_isize] skewness: 0.003; kurtosis: -0.133; ap_prior: 1.87e-05

[infer_isize] inferred maximum insert size: 560 (6.96 sigma)

[bwa_sai2sam_pe_core] time elapses: 30.30 sec

[bwa_sai2sam_pe_core] changing coordinates of 6124 alignments.

[bwa_sai2sam_pe_core] align unmapped mate...

[bwa_paired_sw] 8853 out of 9402 Q17 singletons are mated.

[bwa_paired_sw] 923 out of 1535 Q17 discordant pairs are fixed.

[bwa_sai2sam_pe_core] time elapses: 4.20 sec

[bwa_sai2sam_pe_core] refine gapped alignments... 0.92 sec

[bwa_sai2sam_pe_core] print alignments... 2.05 sec

[bwa_sai2sam_pe_core] 60555264 sequences have been processed.

[bwa_sai2sam_pe_core] convert to sequence coordinate...

[infer_isize] (25, 50, 75) percentile: (453, 463, 473)

[infer_isize] low and high boundaries: 413 and 513 for estimating avg and std

[infer_isize] inferred external isize from 207118 pairs: 463.293 +/- 13.962

[infer_isize] skewness: -0.003; kurtosis: -0.145; ap_prior: 1.69e-05

[infer_isize] inferred maximum insert size: 560 (6.96 sigma)

[bwa_sai2sam_pe_core] time elapses: 30.42 sec

[bwa_sai2sam_pe_core] changing coordinates of 6118 alignments.

[bwa_sai2sam_pe_core] align unmapped mate...

[bwa_paired_sw] 8677 out of 9228 Q17 singletons are mated.

[bwa_paired_sw] 894 out of 1491 Q17 discordant pairs are fixed.

[bwa_sai2sam_pe_core] time elapses: 4.11 sec

[bwa_sai2sam_pe_core] refine gapped alignments... 0.93 sec

[bwa_sai2sam_pe_core] print alignments... 2.11 sec

[bwa_sai2sam_pe_core] 60817408 sequences have been processed.

[bwa_sai2sam_pe_core] convert to sequence coordinate...

[infer_isize] (25, 50, 75) percentile: (453, 463, 473)

[infer_isize] low and high boundaries: 413 and 513 for estimating avg and std

[infer_isize] inferred external isize from 206856 pairs: 463.352 +/- 13.949

[infer_isize] skewness: -0.006; kurtosis: -0.137; ap_prior: 1.70e-05

[infer_isize] inferred maximum insert size: 560 (6.96 sigma)

[bwa_sai2sam_pe_core] time elapses: 31.97 sec

[bwa_sai2sam_pe_core] changing coordinates of 6135 alignments.

[bwa_sai2sam_pe_core] align unmapped mate...

[bwa_paired_sw] 8877 out of 9414 Q17 singletons are mated.

[bwa_paired_sw] 927 out of 1543 Q17 discordant pairs are fixed.

[bwa_sai2sam_pe_core] time elapses: 4.33 sec

[bwa_sai2sam_pe_core] refine gapped alignments... 0.97 sec

[bwa_sai2sam_pe_core] print alignments... 2.10 sec

[bwa_sai2sam_pe_core] 61079552 sequences have been processed.

[bwa_sai2sam_pe_core] convert to sequence coordinate...

[infer_isize] (25, 50, 75) percentile: (453, 463, 473)

[infer_isize] low and high boundaries: 413 and 513 for estimating avg and std

[infer_isize] inferred external isize from 206590 pairs: 463.292 +/- 13.977

[infer_isize] skewness: 0.001; kurtosis: -0.128; ap_prior: 1.77e-05

[infer_isize] inferred maximum insert size: 561 (6.96 sigma)

[bwa_sai2sam_pe_core] time elapses: 31.40 sec

[bwa_sai2sam_pe_core] changing coordinates of 6216 alignments.

[bwa_sai2sam_pe_core] align unmapped mate...

[bwa_paired_sw] 9221 out of 9758 Q17 singletons are mated.

[bwa_paired_sw] 905 out of 1512 Q17 discordant pairs are fixed.

[bwa_sai2sam_pe_core] time elapses: 4.40 sec

[bwa_sai2sam_pe_core] refine gapped alignments... 0.96 sec

[bwa_sai2sam_pe_core] print alignments... 2.06 sec

[bwa_sai2sam_pe_core] 61341696 sequences have been processed.

[bwa_sai2sam_pe_core] convert to sequence coordinate...

[infer_isize] (25, 50, 75) percentile: (453, 463, 473)

[infer_isize] low and high boundaries: 413 and 513 for estimating avg and std

[infer_isize] inferred external isize from 206621 pairs: 463.312 +/- 13.927

[infer_isize] skewness: 0.000; kurtosis: -0.157; ap_prior: 1.81e-05

[infer_isize] inferred maximum insert size: 560 (6.96 sigma)

[bwa_sai2sam_pe_core] time elapses: 31.97 sec

[bwa_sai2sam_pe_core] changing coordinates of 6123 alignments.

[bwa_sai2sam_pe_core] align unmapped mate...

[bwa_paired_sw] 8996 out of 9536 Q17 singletons are mated.

[bwa_paired_sw] 962 out of 1650 Q17 discordant pairs are fixed.

[bwa_sai2sam_pe_core] time elapses: 4.41 sec

[bwa_sai2sam_pe_core] refine gapped alignments... 0.94 sec

[bwa_sai2sam_pe_core] print alignments... 2.06 sec

[bwa_sai2sam_pe_core] 61603840 sequences have been processed.

[bwa_sai2sam_pe_core] convert to sequence coordinate...

[infer_isize] (25, 50, 75) percentile: (453, 463, 473)

[infer_isize] low and high boundaries: 413 and 513 for estimating avg and std

[infer_isize] inferred external isize from 206858 pairs: 463.332 +/- 13.926

[infer_isize] skewness: -0.002; kurtosis: -0.132; ap_prior: 1.77e-05

[infer_isize] inferred maximum insert size: 560 (6.96 sigma)

[bwa_sai2sam_pe_core] time elapses: 31.50 sec

[bwa_sai2sam_pe_core] changing coordinates of 6005 alignments.

[bwa_sai2sam_pe_core] align unmapped mate...

[bwa_paired_sw] 8443 out of 9018 Q17 singletons are mated.

[bwa_paired_sw] 943 out of 1600 Q17 discordant pairs are fixed.

[bwa_sai2sam_pe_core] time elapses: 4.25 sec

[bwa_sai2sam_pe_core] refine gapped alignments... 0.94 sec

[bwa_sai2sam_pe_core] print alignments... 2.09 sec

[bwa_sai2sam_pe_core] 61865984 sequences have been processed.

[bwa_sai2sam_pe_core] convert to sequence coordinate...

[infer_isize] (25, 50, 75) percentile: (453, 463, 473)

[infer_isize] low and high boundaries: 413 and 513 for estimating avg and std

[infer_isize] inferred external isize from 206844 pairs: 463.244 +/- 13.935

[infer_isize] skewness: 0.005; kurtosis: -0.137; ap_prior: 1.71e-05

[infer_isize] inferred maximum insert size: 560 (6.96 sigma)

[bwa_sai2sam_pe_core] time elapses: 31.14 sec

[bwa_sai2sam_pe_core] changing coordinates of 6172 alignments.

[bwa_sai2sam_pe_core] align unmapped mate...

[bwa_paired_sw] 8780 out of 9349 Q17 singletons are mated.

[bwa_paired_sw] 891 out of 1488 Q17 discordant pairs are fixed.

[bwa_sai2sam_pe_core] time elapses: 4.20 sec

[bwa_sai2sam_pe_core] refine gapped alignments... 0.92 sec

[bwa_sai2sam_pe_core] print alignments... 2.09 sec

[bwa_sai2sam_pe_core] 62128128 sequences have been processed.

[bwa_sai2sam_pe_core] convert to sequence coordinate...

[infer_isize] (25, 50, 75) percentile: (453, 463, 473)

[infer_isize] low and high boundaries: 413 and 513 for estimating avg and std

[infer_isize] inferred external isize from 206872 pairs: 463.285 +/- 13.974

[infer_isize] skewness: 0.002; kurtosis: -0.150; ap_prior: 1.78e-05

[infer_isize] inferred maximum insert size: 561 (6.96 sigma)

[bwa_sai2sam_pe_core] time elapses: 30.77 sec

[bwa_sai2sam_pe_core] changing coordinates of 6139 alignments.

[bwa_sai2sam_pe_core] align unmapped mate...

[bwa_paired_sw] 8966 out of 9500 Q17 singletons are mated.

[bwa_paired_sw] 912 out of 1535 Q17 discordant pairs are fixed.

[bwa_sai2sam_pe_core] time elapses: 4.27 sec

[bwa_sai2sam_pe_core] refine gapped alignments... 0.92 sec

[bwa_sai2sam_pe_core] print alignments... 2.06 sec

[bwa_sai2sam_pe_core] 62390272 sequences have been processed.

[bwa_sai2sam_pe_core] convert to sequence coordinate...

[infer_isize] (25, 50, 75) percentile: (453, 463, 473)

[infer_isize] low and high boundaries: 413 and 513 for estimating avg and std

[infer_isize] inferred external isize from 206919 pairs: 463.341 +/- 13.963

[infer_isize] skewness: 0.014; kurtosis: -0.147; ap_prior: 1.78e-05

[infer_isize] inferred maximum insert size: 561 (6.96 sigma)

[bwa_sai2sam_pe_core] time elapses: 31.56 sec

[bwa_sai2sam_pe_core] changing coordinates of 6125 alignments.

[bwa_sai2sam_pe_core] align unmapped mate...

[bwa_paired_sw] 8645 out of 9240 Q17 singletons are mated.

[bwa_paired_sw] 895 out of 1542 Q17 discordant pairs are fixed.

[bwa_sai2sam_pe_core] time elapses: 4.01 sec

[bwa_sai2sam_pe_core] refine gapped alignments... 0.88 sec

[bwa_sai2sam_pe_core] print alignments... 2.11 sec

[bwa_sai2sam_pe_core] 62652416 sequences have been processed.

[bwa_sai2sam_pe_core] convert to sequence coordinate...

[infer_isize] (25, 50, 75) percentile: (453, 463, 473)

[infer_isize] low and high boundaries: 413 and 513 for estimating avg and std

[infer_isize] inferred external isize from 207301 pairs: 463.294 +/- 13.933

[infer_isize] skewness: -0.002; kurtosis: -0.118; ap_prior: 1.69e-05

[infer_isize] inferred maximum insert size: 560 (6.96 sigma)

[bwa_sai2sam_pe_core] time elapses: 29.78 sec

[bwa_sai2sam_pe_core] changing coordinates of 6097 alignments.

[bwa_sai2sam_pe_core] align unmapped mate...

[bwa_paired_sw] 8691 out of 9200 Q17 singletons are mated.

[bwa_paired_sw] 918 out of 1551 Q17 discordant pairs are fixed.

[bwa_sai2sam_pe_core] time elapses: 4.00 sec

[bwa_sai2sam_pe_core] refine gapped alignments... 0.89 sec

[bwa_sai2sam_pe_core] print alignments... 2.06 sec

[bwa_sai2sam_pe_core] 62914560 sequences have been processed.

[bwa_sai2sam_pe_core] convert to sequence coordinate...

[infer_isize] (25, 50, 75) percentile: (453, 463, 473)

[infer_isize] low and high boundaries: 413 and 513 for estimating avg and std

[infer_isize] inferred external isize from 206465 pairs: 463.260 +/- 13.963

[infer_isize] skewness: 0.004; kurtosis: -0.141; ap_prior: 1.82e-05

[infer_isize] inferred maximum insert size: 560 (6.96 sigma)

[bwa_sai2sam_pe_core] time elapses: 29.75 sec

[bwa_sai2sam_pe_core] changing coordinates of 6093 alignments.

[bwa_sai2sam_pe_core] align unmapped mate...

[bwa_paired_sw] 8891 out of 9450 Q17 singletons are mated.

[bwa_paired_sw] 913 out of 1573 Q17 discordant pairs are fixed.

[bwa_sai2sam_pe_core] time elapses: 4.18 sec

[bwa_sai2sam_pe_core] refine gapped alignments... 0.89 sec

[bwa_sai2sam_pe_core] print alignments... 2.08 sec

[bwa_sai2sam_pe_core] 63176704 sequences have been processed.

[bwa_sai2sam_pe_core] convert to sequence coordinate...

[infer_isize] (25, 50, 75) percentile: (453, 463, 473)

[infer_isize] low and high boundaries: 413 and 513 for estimating avg and std

[infer_isize] inferred external isize from 207369 pairs: 463.348 +/- 13.950

[infer_isize] skewness: 0.002; kurtosis: -0.137; ap_prior: 1.78e-05

[infer_isize] inferred maximum insert size: 560 (6.96 sigma)

[bwa_sai2sam_pe_core] time elapses: 32.93 sec

[bwa_sai2sam_pe_core] changing coordinates of 6152 alignments.

[bwa_sai2sam_pe_core] align unmapped mate...

[bwa_paired_sw] 8388 out of 8970 Q17 singletons are mated.

[bwa_paired_sw] 848 out of 1492 Q17 discordant pairs are fixed.

[bwa_sai2sam_pe_core] time elapses: 3.99 sec

[bwa_sai2sam_pe_core] refine gapped alignments... 1.05 sec

[bwa_sai2sam_pe_core] print alignments... 2.22 sec

[bwa_sai2sam_pe_core] 63438848 sequences have been processed.

[bwa_sai2sam_pe_core] convert to sequence coordinate...

[infer_isize] (25, 50, 75) percentile: (453, 463, 473)

[infer_isize] low and high boundaries: 413 and 513 for estimating avg and std

[infer_isize] inferred external isize from 207115 pairs: 463.225 +/- 13.972

[infer_isize] skewness: 0.005; kurtosis: -0.119; ap_prior: 1.71e-05

[infer_isize] inferred maximum insert size: 560 (6.96 sigma)

[bwa_sai2sam_pe_core] time elapses: 30.09 sec

[bwa_sai2sam_pe_core] changing coordinates of 6118 alignments.

[bwa_sai2sam_pe_core] align unmapped mate...

[bwa_paired_sw] 8655 out of 9180 Q17 singletons are mated.

[bwa_paired_sw] 894 out of 1524 Q17 discordant pairs are fixed.

[bwa_sai2sam_pe_core] time elapses: 4.07 sec

[bwa_sai2sam_pe_core] refine gapped alignments... 0.95 sec

[bwa_sai2sam_pe_core] print alignments... 2.11 sec

[bwa_sai2sam_pe_core] 63700992 sequences have been processed.

[bwa_sai2sam_pe_core] convert to sequence coordinate...

[infer_isize] (25, 50, 75) percentile: (453, 463, 473)

[infer_isize] low and high boundaries: 413 and 513 for estimating avg and std

[infer_isize] inferred external isize from 206990 pairs: 463.291 +/- 13.939

[infer_isize] skewness: -0.005; kurtosis: -0.125; ap_prior: 1.72e-05

[infer_isize] inferred maximum insert size: 560 (6.96 sigma)

[bwa_sai2sam_pe_core] time elapses: 29.96 sec

[bwa_sai2sam_pe_core] changing coordinates of 6167 alignments.

[bwa_sai2sam_pe_core] align unmapped mate...

[bwa_paired_sw] 8746 out of 9307 Q17 singletons are mated.

[bwa_paired_sw] 862 out of 1489 Q17 discordant pairs are fixed.

[bwa_sai2sam_pe_core] time elapses: 4.00 sec

[bwa_sai2sam_pe_core] refine gapped alignments... 0.92 sec

[bwa_sai2sam_pe_core] print alignments... 2.08 sec

[bwa_sai2sam_pe_core] 63963136 sequences have been processed.

[bwa_sai2sam_pe_core] convert to sequence coordinate...

[infer_isize] (25, 50, 75) percentile: (453, 463, 473)

[infer_isize] low and high boundaries: 413 and 513 for estimating avg and std

[infer_isize] inferred external isize from 207204 pairs: 463.298 +/- 13.963

[infer_isize] skewness: -0.001; kurtosis: -0.129; ap_prior: 1.81e-05

[infer_isize] inferred maximum insert size: 560 (6.96 sigma)

[bwa_sai2sam_pe_core] time elapses: 30.16 sec

[bwa_sai2sam_pe_core] changing coordinates of 6017 alignments.

[bwa_sai2sam_pe_core] align unmapped mate...

[bwa_paired_sw] 8480 out of 9035 Q17 singletons are mated.

[bwa_paired_sw] 932 out of 1511 Q17 discordant pairs are fixed.

[bwa_sai2sam_pe_core] time elapses: 3.96 sec

[bwa_sai2sam_pe_core] refine gapped alignments... 0.91 sec

[bwa_sai2sam_pe_core] print alignments... 2.12 sec

[bwa_sai2sam_pe_core] 64225280 sequences have been processed.

[bwa_sai2sam_pe_core] convert to sequence coordinate...

[infer_isize] (25, 50, 75) percentile: (453, 463, 473)

[infer_isize] low and high boundaries: 413 and 513 for estimating avg and std

[infer_isize] inferred external isize from 207285 pairs: 463.233 +/- 13.960

[infer_isize] skewness: 0.000; kurtosis: -0.133; ap_prior: 1.74e-05

[infer_isize] inferred maximum insert size: 560 (6.96 sigma)

[bwa_sai2sam_pe_core] time elapses: 29.99 sec

[bwa_sai2sam_pe_core] changing coordinates of 6158 alignments.

[bwa_sai2sam_pe_core] align unmapped mate...

[bwa_paired_sw] 8463 out of 8938 Q17 singletons are mated.

[bwa_paired_sw] 873 out of 1516 Q17 discordant pairs are fixed.

[bwa_sai2sam_pe_core] time elapses: 3.92 sec

[bwa_sai2sam_pe_core] refine gapped alignments... 0.92 sec

[bwa_sai2sam_pe_core] print alignments... 2.10 sec

[bwa_sai2sam_pe_core] 64487424 sequences have been processed.

[bwa_sai2sam_pe_core] convert to sequence coordinate...

[infer_isize] (25, 50, 75) percentile: (453, 463, 473)

[infer_isize] low and high boundaries: 413 and 513 for estimating avg and std

[infer_isize] inferred external isize from 207040 pairs: 463.297 +/- 13.963

[infer_isize] skewness: 0.003; kurtosis: -0.134; ap_prior: 1.74e-05

[infer_isize] inferred maximum insert size: 560 (6.96 sigma)

[bwa_sai2sam_pe_core] time elapses: 30.55 sec

[bwa_sai2sam_pe_core] changing coordinates of 6052 alignments.

[bwa_sai2sam_pe_core] align unmapped mate...

[bwa_paired_sw] 8629 out of 9167 Q17 singletons are mated.

[bwa_paired_sw] 952 out of 1572 Q17 discordant pairs are fixed.

[bwa_sai2sam_pe_core] time elapses: 4.02 sec

[bwa_sai2sam_pe_core] refine gapped alignments... 0.91 sec

[bwa_sai2sam_pe_core] print alignments... 2.09 sec

[bwa_sai2sam_pe_core] 64749568 sequences have been processed.

[bwa_sai2sam_pe_core] convert to sequence coordinate...

[infer_isize] (25, 50, 75) percentile: (453, 463, 473)

[infer_isize] low and high boundaries: 413 and 513 for estimating avg and std

[infer_isize] inferred external isize from 207115 pairs: 463.320 +/- 13.927

[infer_isize] skewness: 0.001; kurtosis: -0.126; ap_prior: 1.85e-05

[infer_isize] inferred maximum insert size: 560 (6.96 sigma)

[bwa_sai2sam_pe_core] time elapses: 29.62 sec

[bwa_sai2sam_pe_core] changing coordinates of 6016 alignments.

[bwa_sai2sam_pe_core] align unmapped mate...

[bwa_paired_sw] 8800 out of 9364 Q17 singletons are mated.

[bwa_paired_sw] 867 out of 1517 Q17 discordant pairs are fixed.

[bwa_sai2sam_pe_core] time elapses: 4.06 sec

[bwa_sai2sam_pe_core] refine gapped alignments... 0.93 sec

[bwa_sai2sam_pe_core] print alignments... 2.09 sec

[bwa_sai2sam_pe_core] 65011712 sequences have been processed.

[bwa_sai2sam_pe_core] convert to sequence coordinate...

[infer_isize] (25, 50, 75) percentile: (453, 463, 473)

[infer_isize] low and high boundaries: 413 and 513 for estimating avg and std

[infer_isize] inferred external isize from 206782 pairs: 463.273 +/- 13.920

[infer_isize] skewness: 0.007; kurtosis: -0.142; ap_prior: 1.75e-05

[infer_isize] inferred maximum insert size: 560 (6.96 sigma)

[bwa_sai2sam_pe_core] time elapses: 30.52 sec

[bwa_sai2sam_pe_core] changing coordinates of 6044 alignments.

[bwa_sai2sam_pe_core] align unmapped mate...

[bwa_paired_sw] 8641 out of 9207 Q17 singletons are mated.

[bwa_paired_sw] 931 out of 1550 Q17 discordant pairs are fixed.

[bwa_sai2sam_pe_core] time elapses: 4.20 sec

[bwa_sai2sam_pe_core] refine gapped alignments... 0.96 sec

[bwa_sai2sam_pe_core] print alignments... 2.20 sec

[bwa_sai2sam_pe_core] 65273856 sequences have been processed.

[bwa_sai2sam_pe_core] convert to sequence coordinate...

[infer_isize] (25, 50, 75) percentile: (453, 463, 473)

[infer_isize] low and high boundaries: 413 and 513 for estimating avg and std

[infer_isize] inferred external isize from 206865 pairs: 463.289 +/- 13.940

[infer_isize] skewness: -0.005; kurtosis: -0.142; ap_prior: 1.75e-05

[infer_isize] inferred maximum insert size: 560 (6.96 sigma)

[bwa_sai2sam_pe_core] time elapses: 32.50 sec

[bwa_sai2sam_pe_core] changing coordinates of 6039 alignments.

[bwa_sai2sam_pe_core] align unmapped mate...

[bwa_paired_sw] 8733 out of 9317 Q17 singletons are mated.

[bwa_paired_sw] 888 out of 1499 Q17 discordant pairs are fixed.

[bwa_sai2sam_pe_core] time elapses: 4.24 sec

[bwa_sai2sam_pe_core] refine gapped alignments... 1.01 sec

[bwa_sai2sam_pe_core] print alignments... 2.17 sec

[bwa_sai2sam_pe_core] 65536000 sequences have been processed.

[bwa_sai2sam_pe_core] convert to sequence coordinate...

[infer_isize] (25, 50, 75) percentile: (453, 463, 473)

[infer_isize] low and high boundaries: 413 and 513 for estimating avg and std

[infer_isize] inferred external isize from 207137 pairs: 463.240 +/- 13.950

[infer_isize] skewness: -0.002; kurtosis: -0.122; ap_prior: 1.78e-05

[infer_isize] inferred maximum insert size: 560 (6.96 sigma)

[bwa_sai2sam_pe_core] time elapses: 32.06 sec

[bwa_sai2sam_pe_core] changing coordinates of 5978 alignments.

[bwa_sai2sam_pe_core] align unmapped mate...

[bwa_paired_sw] 8451 out of 9000 Q17 singletons are mated.

[bwa_paired_sw] 926 out of 1534 Q17 discordant pairs are fixed.

[bwa_sai2sam_pe_core] time elapses: 4.18 sec

[bwa_sai2sam_pe_core] refine gapped alignments... 1.05 sec

[bwa_sai2sam_pe_core] print alignments... 2.22 sec

[bwa_sai2sam_pe_core] 65798144 sequences have been processed.

[bwa_sai2sam_pe_core] convert to sequence coordinate...

[infer_isize] (25, 50, 75) percentile: (453, 463, 473)

[infer_isize] low and high boundaries: 413 and 513 for estimating avg and std

[infer_isize] inferred external isize from 206931 pairs: 463.306 +/- 13.936

[infer_isize] skewness: 0.003; kurtosis: -0.124; ap_prior: 1.76e-05

[infer_isize] inferred maximum insert size: 560 (6.96 sigma)

[bwa_sai2sam_pe_core] time elapses: 33.38 sec

[bwa_sai2sam_pe_core] changing coordinates of 6202 alignments.

[bwa_sai2sam_pe_core] align unmapped mate...

[bwa_paired_sw] 8564 out of 9089 Q17 singletons are mated.

[bwa_paired_sw] 872 out of 1485 Q17 discordant pairs are fixed.

[bwa_sai2sam_pe_core] time elapses: 4.17 sec

[bwa_sai2sam_pe_core] refine gapped alignments... 1.03 sec

[bwa_sai2sam_pe_core] print alignments... 2.17 sec

[bwa_sai2sam_pe_core] 66060288 sequences have been processed.

[bwa_sai2sam_pe_core] convert to sequence coordinate...

[infer_isize] (25, 50, 75) percentile: (453, 463, 473)

[infer_isize] low and high boundaries: 413 and 513 for estimating avg and std

[infer_isize] inferred external isize from 207068 pairs: 463.305 +/- 13.939

[infer_isize] skewness: 0.003; kurtosis: -0.120; ap_prior: 1.72e-05

[infer_isize] inferred maximum insert size: 560 (6.96 sigma)

[bwa_sai2sam_pe_core] time elapses: 32.00 sec

[bwa_sai2sam_pe_core] changing coordinates of 5993 alignments.

[bwa_sai2sam_pe_core] align unmapped mate...

[bwa_paired_sw] 8399 out of 8922 Q17 singletons are mated.

[bwa_paired_sw] 888 out of 1504 Q17 discordant pairs are fixed.

[bwa_sai2sam_pe_core] time elapses: 4.14 sec

[bwa_sai2sam_pe_core] refine gapped alignments... 0.99 sec

[bwa_sai2sam_pe_core] print alignments... 2.17 sec

[bwa_sai2sam_pe_core] 66322432 sequences have been processed.

[bwa_sai2sam_pe_core] convert to sequence coordinate...

[infer_isize] (25, 50, 75) percentile: (453, 463, 473)

[infer_isize] low and high boundaries: 413 and 513 for estimating avg and std

[infer_isize] inferred external isize from 207178 pairs: 463.306 +/- 13.966

[infer_isize] skewness: 0.003; kurtosis: -0.132; ap_prior: 1.77e-05

[infer_isize] inferred maximum insert size: 561 (6.96 sigma)

[bwa_sai2sam_pe_core] time elapses: 32.01 sec

[bwa_sai2sam_pe_core] changing coordinates of 6260 alignments.

[bwa_sai2sam_pe_core] align unmapped mate...

[bwa_paired_sw] 8645 out of 9176 Q17 singletons are mated.

[bwa_paired_sw] 916 out of 1494 Q17 discordant pairs are fixed.

[bwa_sai2sam_pe_core] time elapses: 4.22 sec

[bwa_sai2sam_pe_core] refine gapped alignments... 0.98 sec

[bwa_sai2sam_pe_core] print alignments... 2.13 sec

[bwa_sai2sam_pe_core] 66584576 sequences have been processed.

[bwa_sai2sam_pe_core] convert to sequence coordinate...

[infer_isize] (25, 50, 75) percentile: (453, 463, 473)

[infer_isize] low and high boundaries: 413 and 513 for estimating avg and std

[infer_isize] inferred external isize from 207243 pairs: 463.290 +/- 13.940

[infer_isize] skewness: -0.002; kurtosis: -0.139; ap_prior: 1.73e-05

[infer_isize] inferred maximum insert size: 560 (6.96 sigma)

[bwa_sai2sam_pe_core] time elapses: 34.58 sec

[bwa_sai2sam_pe_core] changing coordinates of 6047 alignments.

[bwa_sai2sam_pe_core] align unmapped mate...

[bwa_paired_sw] 8550 out of 9071 Q17 singletons are mated.

[bwa_paired_sw] 903 out of 1486 Q17 discordant pairs are fixed.

[bwa_sai2sam_pe_core] time elapses: 4.21 sec

[bwa_sai2sam_pe_core] refine gapped alignments... 0.99 sec

[bwa_sai2sam_pe_core] print alignments... 2.21 sec

[bwa_sai2sam_pe_core] 66846720 sequences have been processed.

[bwa_sai2sam_pe_core] convert to sequence coordinate...

[infer_isize] (25, 50, 75) percentile: (453, 463, 473)

[infer_isize] low and high boundaries: 413 and 513 for estimating avg and std

[infer_isize] inferred external isize from 206926 pairs: 463.289 +/- 13.909

[infer_isize] skewness: -0.002; kurtosis: -0.150; ap_prior: 1.70e-05

[infer_isize] inferred maximum insert size: 560 (6.96 sigma)

[bwa_sai2sam_pe_core] time elapses: 34.47 sec

[bwa_sai2sam_pe_core] changing coordinates of 6143 alignments.

[bwa_sai2sam_pe_core] align unmapped mate...

[bwa_paired_sw] 8488 out of 9019 Q17 singletons are mated.

[bwa_paired_sw] 892 out of 1507 Q17 discordant pairs are fixed.

[bwa_sai2sam_pe_core] time elapses: 4.22 sec

[bwa_sai2sam_pe_core] refine gapped alignments... 0.97 sec

[bwa_sai2sam_pe_core] print alignments... 2.11 sec

[bwa_sai2sam_pe_core] 67108864 sequences have been processed.

[bwa_sai2sam_pe_core] convert to sequence coordinate...

[infer_isize] (25, 50, 75) percentile: (453, 463, 473)

[infer_isize] low and high boundaries: 413 and 513 for estimating avg and std

[infer_isize] inferred external isize from 206869 pairs: 463.303 +/- 13.938

[infer_isize] skewness: 0.004; kurtosis: -0.134; ap_prior: 1.78e-05

[infer_isize] inferred maximum insert size: 560 (6.96 sigma)

[bwa_sai2sam_pe_core] time elapses: 32.99 sec

[bwa_sai2sam_pe_core] changing coordinates of 6164 alignments.

[bwa_sai2sam_pe_core] align unmapped mate...

[bwa_paired_sw] 8621 out of 9154 Q17 singletons are mated.

[bwa_paired_sw] 910 out of 1526 Q17 discordant pairs are fixed.

[bwa_sai2sam_pe_core] time elapses: 4.24 sec

[bwa_sai2sam_pe_core] refine gapped alignments... 0.97 sec

[bwa_sai2sam_pe_core] print alignments... 2.13 sec

[bwa_sai2sam_pe_core] 67371008 sequences have been processed.

[bwa_sai2sam_pe_core] convert to sequence coordinate...

[infer_isize] (25, 50, 75) percentile: (453, 463, 473)

[infer_isize] low and high boundaries: 413 and 513 for estimating avg and std

[infer_isize] inferred external isize from 207248 pairs: 463.289 +/- 13.938

[infer_isize] skewness: -0.006; kurtosis: -0.146; ap_prior: 1.79e-05

[infer_isize] inferred maximum insert size: 560 (6.96 sigma)

[bwa_sai2sam_pe_core] time elapses: 33.05 sec

[bwa_sai2sam_pe_core] changing coordinates of 6182 alignments.

[bwa_sai2sam_pe_core] align unmapped mate...

[bwa_paired_sw] 8431 out of 9012 Q17 singletons are mated.

[bwa_paired_sw] 877 out of 1537 Q17 discordant pairs are fixed.

[bwa_sai2sam_pe_core] time elapses: 4.19 sec

[bwa_sai2sam_pe_core] refine gapped alignments... 0.96 sec

[bwa_sai2sam_pe_core] print alignments... 2.11 sec

[bwa_sai2sam_pe_core] 67633152 sequences have been processed.

[bwa_sai2sam_pe_core] convert to sequence coordinate...

[infer_isize] (25, 50, 75) percentile: (453, 463, 473)

[infer_isize] low and high boundaries: 413 and 513 for estimating avg and std

[infer_isize] inferred external isize from 207470 pairs: 463.313 +/- 13.946

[infer_isize] skewness: -0.001; kurtosis: -0.140; ap_prior: 1.68e-05

[infer_isize] inferred maximum insert size: 560 (6.96 sigma)

[bwa_sai2sam_pe_core] time elapses: 34.98 sec

[bwa_sai2sam_pe_core] changing coordinates of 6119 alignments.

[bwa_sai2sam_pe_core] align unmapped mate...

[bwa_paired_sw] 8513 out of 9047 Q17 singletons are mated.

[bwa_paired_sw] 881 out of 1484 Q17 discordant pairs are fixed.

[bwa_sai2sam_pe_core] time elapses: 4.29 sec

[bwa_sai2sam_pe_core] refine gapped alignments... 1.03 sec

[bwa_sai2sam_pe_core] print alignments... 2.20 sec

[bwa_sai2sam_pe_core] 67895296 sequences have been processed.

[bwa_sai2sam_pe_core] convert to sequence coordinate...

[infer_isize] (25, 50, 75) percentile: (453, 463, 473)

[infer_isize] low and high boundaries: 413 and 513 for estimating avg and std

[infer_isize] inferred external isize from 207228 pairs: 463.302 +/- 13.954

[infer_isize] skewness: -0.003; kurtosis: -0.138; ap_prior: 1.78e-05

[infer_isize] inferred maximum insert size: 560 (6.96 sigma)

[bwa_sai2sam_pe_core] time elapses: 35.05 sec

[bwa_sai2sam_pe_core] changing coordinates of 6128 alignments.

[bwa_sai2sam_pe_core] align unmapped mate...

[bwa_paired_sw] 8421 out of 8957 Q17 singletons are mated.

[bwa_paired_sw] 875 out of 1510 Q17 discordant pairs are fixed.

[bwa_sai2sam_pe_core] time elapses: 4.30 sec

[bwa_sai2sam_pe_core] refine gapped alignments... 1.01 sec

[bwa_sai2sam_pe_core] print alignments... 2.20 sec

[bwa_sai2sam_pe_core] 68157440 sequences have been processed.

[bwa_sai2sam_pe_core] convert to sequence coordinate...

[infer_isize] (25, 50, 75) percentile: (453, 463, 473)

[infer_isize] low and high boundaries: 413 and 513 for estimating avg and std

[infer_isize] inferred external isize from 207087 pairs: 463.297 +/- 13.981

[infer_isize] skewness: 0.018; kurtosis: -0.133; ap_prior: 1.89e-05

[infer_isize] inferred maximum insert size: 561 (6.96 sigma)

[bwa_sai2sam_pe_core] time elapses: 36.07 sec

[bwa_sai2sam_pe_core] changing coordinates of 6218 alignments.

[bwa_sai2sam_pe_core] align unmapped mate...

[bwa_paired_sw] 8490 out of 8990 Q17 singletons are mated.

[bwa_paired_sw] 870 out of 1484 Q17 discordant pairs are fixed.

[bwa_sai2sam_pe_core] time elapses: 4.26 sec

[bwa_sai2sam_pe_core] refine gapped alignments... 1.04 sec

[bwa_sai2sam_pe_core] print alignments... 2.21 sec

[bwa_sai2sam_pe_core] 68419584 sequences have been processed.

[bwa_sai2sam_pe_core] convert to sequence coordinate...

[infer_isize] (25, 50, 75) percentile: (453, 463, 473)

[infer_isize] low and high boundaries: 413 and 513 for estimating avg and std

[infer_isize] inferred external isize from 207045 pairs: 463.242 +/- 13.920

[infer_isize] skewness: 0.005; kurtosis: -0.119; ap_prior: 1.90e-05

[infer_isize] inferred maximum insert size: 560 (6.96 sigma)

[bwa_sai2sam_pe_core] time elapses: 33.22 sec

[bwa_sai2sam_pe_core] changing coordinates of 6059 alignments.

[bwa_sai2sam_pe_core] align unmapped mate...

[bwa_paired_sw] 8580 out of 9084 Q17 singletons are mated.

[bwa_paired_sw] 928 out of 1567 Q17 discordant pairs are fixed.

[bwa_sai2sam_pe_core] time elapses: 4.25 sec

[bwa_sai2sam_pe_core] refine gapped alignments... 1.02 sec

[bwa_sai2sam_pe_core] print alignments... 2.28 sec

[bwa_sai2sam_pe_core] 68681728 sequences have been processed.

[bwa_sai2sam_pe_core] convert to sequence coordinate...

[infer_isize] (25, 50, 75) percentile: (453, 463, 473)

[infer_isize] low and high boundaries: 413 and 513 for estimating avg and std

[infer_isize] inferred external isize from 206899 pairs: 463.281 +/- 13.982

[infer_isize] skewness: -0.003; kurtosis: -0.125; ap_prior: 1.78e-05

[infer_isize] inferred maximum insert size: 561 (6.96 sigma)

[bwa_sai2sam_pe_core] time elapses: 34.06 sec

[bwa_sai2sam_pe_core] changing coordinates of 6061 alignments.

[bwa_sai2sam_pe_core] align unmapped mate...

[bwa_paired_sw] 8529 out of 9058 Q17 singletons are mated.

[bwa_paired_sw] 856 out of 1470 Q17 discordant pairs are fixed.

[bwa_sai2sam_pe_core] time elapses: 4.23 sec

[bwa_sai2sam_pe_core] refine gapped alignments... 1.08 sec

[bwa_sai2sam_pe_core] print alignments... 2.39 sec

[bwa_sai2sam_pe_core] 68943872 sequences have been processed.

[bwa_sai2sam_pe_core] convert to sequence coordinate...

[infer_isize] (25, 50, 75) percentile: (453, 463, 473)

[infer_isize] low and high boundaries: 413 and 513 for estimating avg and std

[infer_isize] inferred external isize from 207403 pairs: 463.286 +/- 13.961

[infer_isize] skewness: 0.005; kurtosis: -0.131; ap_prior: 1.74e-05

[infer_isize] inferred maximum insert size: 560 (6.96 sigma)

[bwa_sai2sam_pe_core] time elapses: 34.76 sec

[bwa_sai2sam_pe_core] changing coordinates of 5969 alignments.

[bwa_sai2sam_pe_core] align unmapped mate...

[bwa_paired_sw] 8317 out of 8866 Q17 singletons are mated.

[bwa_paired_sw] 896 out of 1498 Q17 discordant pairs are fixed.

[bwa_sai2sam_pe_core] time elapses: 4.21 sec

[bwa_sai2sam_pe_core] refine gapped alignments... 1.06 sec

[bwa_sai2sam_pe_core] print alignments... 2.40 sec

[bwa_sai2sam_pe_core] 69206016 sequences have been processed.

[bwa_sai2sam_pe_core] convert to sequence coordinate...

[infer_isize] (25, 50, 75) percentile: (453, 463, 473)

[infer_isize] low and high boundaries: 413 and 513 for estimating avg and std

[infer_isize] inferred external isize from 207306 pairs: 463.292 +/- 13.929

[infer_isize] skewness: 0.006; kurtosis: -0.138; ap_prior: 1.76e-05

[infer_isize] inferred maximum insert size: 560 (6.96 sigma)

[bwa_sai2sam_pe_core] time elapses: 33.51 sec

[bwa_sai2sam_pe_core] changing coordinates of 6176 alignments.

[bwa_sai2sam_pe_core] align unmapped mate...

[bwa_paired_sw] 8488 out of 9004 Q17 singletons are mated.

[bwa_paired_sw] 913 out of 1513 Q17 discordant pairs are fixed.

[bwa_sai2sam_pe_core] time elapses: 4.20 sec

[bwa_sai2sam_pe_core] refine gapped alignments... 1.05 sec

[bwa_sai2sam_pe_core] print alignments... 2.29 sec

[bwa_sai2sam_pe_core] 69468160 sequences have been processed.

[bwa_sai2sam_pe_core] convert to sequence coordinate...

[infer_isize] (25, 50, 75) percentile: (453, 463, 473)

[infer_isize] low and high boundaries: 413 and 513 for estimating avg and std

[infer_isize] inferred external isize from 207107 pairs: 463.318 +/- 13.982

[infer_isize] skewness: -0.004; kurtosis: -0.135; ap_prior: 1.82e-05

[infer_isize] inferred maximum insert size: 561 (6.96 sigma)

[bwa_sai2sam_pe_core] time elapses: 60.48 sec

[bwa_sai2sam_pe_core] changing coordinates of 6210 alignments.

[bwa_sai2sam_pe_core] align unmapped mate...

[bwa_paired_sw] 8372 out of 8859 Q17 singletons are mated.

[bwa_paired_sw] 927 out of 1556 Q17 discordant pairs are fixed.

[bwa_sai2sam_pe_core] time elapses: 20.86 sec

[bwa_sai2sam_pe_core] refine gapped alignments... 4.94 sec

[bwa_sai2sam_pe_core] print alignments... 11.79 sec

[bwa_sai2sam_pe_core] 69730304 sequences have been processed.

[bwa_sai2sam_pe_core] convert to sequence coordinate...

[infer_isize] (25, 50, 75) percentile: (453, 463, 473)

[infer_isize] low and high boundaries: 413 and 513 for estimating avg and std

[infer_isize] inferred external isize from 207315 pairs: 463.300 +/- 13.934

[infer_isize] skewness: -0.005; kurtosis: -0.131; ap_prior: 1.81e-05

[infer_isize] inferred maximum insert size: 560 (6.96 sigma)

[bwa_sai2sam_pe_core] time elapses: 321.07 sec

[bwa_sai2sam_pe_core] changing coordinates of 5989 alignments.

[bwa_sai2sam_pe_core] align unmapped mate...

[bwa_paired_sw] 8351 out of 8892 Q17 singletons are mated.

[bwa_paired_sw] 881 out of 1531 Q17 discordant pairs are fixed.

[bwa_sai2sam_pe_core] time elapses: 19.51 sec

[bwa_sai2sam_pe_core] refine gapped alignments... 5.02 sec

[bwa_sai2sam_pe_core] print alignments... 13.08 sec

[bwa_sai2sam_pe_core] 69992448 sequences have been processed.

[bwa_sai2sam_pe_core] convert to sequence coordinate...

[infer_isize] (25, 50, 75) percentile: (453, 463, 473)

[infer_isize] low and high boundaries: 413 and 513 for estimating avg and std

[infer_isize] inferred external isize from 207076 pairs: 463.285 +/- 13.938

[infer_isize] skewness: 0.007; kurtosis: -0.123; ap_prior: 1.66e-05

[infer_isize] inferred maximum insert size: 560 (6.96 sigma)

[bwa_sai2sam_pe_core] time elapses: 119.85 sec

[bwa_sai2sam_pe_core] changing coordinates of 6071 alignments.

[bwa_sai2sam_pe_core] align unmapped mate...

[bwa_paired_sw] 8526 out of 9115 Q17 singletons are mated.

[bwa_paired_sw] 897 out of 1492 Q17 discordant pairs are fixed.

[bwa_sai2sam_pe_core] time elapses: 4.15 sec

[bwa_sai2sam_pe_core] refine gapped alignments... 1.05 sec

[bwa_sai2sam_pe_core] print alignments... 2.21 sec

[bwa_sai2sam_pe_core] 70254592 sequences have been processed.

[bwa_sai2sam_pe_core] convert to sequence coordinate...

[infer_isize] (25, 50, 75) percentile: (453, 463, 473)

[infer_isize] low and high boundaries: 413 and 513 for estimating avg and std

[infer_isize] inferred external isize from 207142 pairs: 463.235 +/- 13.947

[infer_isize] skewness: 0.004; kurtosis: -0.134; ap_prior: 1.67e-05

[infer_isize] inferred maximum insert size: 560 (6.96 sigma)

[bwa_sai2sam_pe_core] time elapses: 115.78 sec

[bwa_sai2sam_pe_core] changing coordinates of 6042 alignments.

[bwa_sai2sam_pe_core] align unmapped mate...

[bwa_paired_sw] 8795 out of 9312 Q17 singletons are mated.

[bwa_paired_sw] 898 out of 1491 Q17 discordant pairs are fixed.

[bwa_sai2sam_pe_core] time elapses: 21.25 sec

[bwa_sai2sam_pe_core] refine gapped alignments... 4.34 sec

[bwa_sai2sam_pe_core] print alignments... 10.46 sec

[bwa_sai2sam_pe_core] 70516736 sequences have been processed.

[bwa_sai2sam_pe_core] convert to sequence coordinate...

[infer_isize] (25, 50, 75) percentile: (453, 463, 473)

[infer_isize] low and high boundaries: 413 and 513 for estimating avg and std

[infer_isize] inferred external isize from 206757 pairs: 463.297 +/- 13.962

[infer_isize] skewness: -0.001; kurtosis: -0.133; ap_prior: 1.85e-05

[infer_isize] inferred maximum insert size: 560 (6.96 sigma)

[bwa_sai2sam_pe_core] time elapses: 147.42 sec

[bwa_sai2sam_pe_core] changing coordinates of 6086 alignments.

[bwa_sai2sam_pe_core] align unmapped mate...

[bwa_paired_sw] 8866 out of 9380 Q17 singletons are mated.

[bwa_paired_sw] 927 out of 1575 Q17 discordant pairs are fixed.

[bwa_sai2sam_pe_core] time elapses: 19.93 sec

[bwa_sai2sam_pe_core] refine gapped alignments... 3.84 sec

[bwa_sai2sam_pe_core] print alignments... 10.63 sec

[bwa_sai2sam_pe_core] 70778880 sequences have been processed.

[bwa_sai2sam_pe_core] convert to sequence coordinate...

[infer_isize] (25, 50, 75) percentile: (453, 463, 473)

[infer_isize] low and high boundaries: 413 and 513 for estimating avg and std

[infer_isize] inferred external isize from 206753 pairs: 463.283 +/- 13.972

[infer_isize] skewness: 0.003; kurtosis: -0.135; ap_prior: 1.76e-05

[infer_isize] inferred maximum insert size: 561 (6.96 sigma)

[bwa_sai2sam_pe_core] time elapses: 139.98 sec

[bwa_sai2sam_pe_core] changing coordinates of 6141 alignments.

[bwa_sai2sam_pe_core] align unmapped mate...

[bwa_paired_sw] 8726 out of 9293 Q17 singletons are mated.

[bwa_paired_sw] 879 out of 1529 Q17 discordant pairs are fixed.

[bwa_sai2sam_pe_core] time elapses: 21.92 sec

[bwa_sai2sam_pe_core] refine gapped alignments... 5.19 sec

[bwa_sai2sam_pe_core] print alignments... 11.80 sec

[bwa_sai2sam_pe_core] 71041024 sequences have been processed.

[bwa_sai2sam_pe_core] convert to sequence coordinate...

[infer_isize] (25, 50, 75) percentile: (453, 463, 473)

[infer_isize] low and high boundaries: 413 and 513 for estimating avg and std

[infer_isize] inferred external isize from 206764 pairs: 463.246 +/- 13.920

[infer_isize] skewness: 0.005; kurtosis: -0.145; ap_prior: 1.67e-05

[infer_isize] inferred maximum insert size: 560 (6.96 sigma)

[bwa_sai2sam_pe_core] time elapses: 46.07 sec

[bwa_sai2sam_pe_core] changing coordinates of 6030 alignments.

[bwa_sai2sam_pe_core] align unmapped mate...

[bwa_paired_sw] 8753 out of 9324 Q17 singletons are mated.

[bwa_paired_sw] 924 out of 1514 Q17 discordant pairs are fixed.

[bwa_sai2sam_pe_core] time elapses: 4.26 sec

[bwa_sai2sam_pe_core] refine gapped alignments... 0.99 sec

[bwa_sai2sam_pe_core] print alignments... 2.34 sec

[bwa_sai2sam_pe_core] 71303168 sequences have been processed.

[bwa_sai2sam_pe_core] convert to sequence coordinate...

[infer_isize] (25, 50, 75) percentile: (453, 463, 473)

[infer_isize] low and high boundaries: 413 and 513 for estimating avg and std

[infer_isize] inferred external isize from 206826 pairs: 463.217 +/- 13.922

[infer_isize] skewness: 0.007; kurtosis: -0.137; ap_prior: 1.79e-05

[infer_isize] inferred maximum insert size: 560 (6.96 sigma)

[bwa_sai2sam_pe_core] time elapses: 136.80 sec

[bwa_sai2sam_pe_core] changing coordinates of 6221 alignments.

[bwa_sai2sam_pe_core] align unmapped mate...

[bwa_paired_sw] 8827 out of 9371 Q17 singletons are mated.

[bwa_paired_sw] 907 out of 1535 Q17 discordant pairs are fixed.

[bwa_sai2sam_pe_core] time elapses: 21.85 sec

[bwa_sai2sam_pe_core] refine gapped alignments... 5.07 sec

[bwa_sai2sam_pe_core] print alignments... 13.17 sec

[bwa_sai2sam_pe_core] 71565312 sequences have been processed.

[bwa_sai2sam_pe_core] convert to sequence coordinate...

[infer_isize] (25, 50, 75) percentile: (453, 463, 473)

[infer_isize] low and high boundaries: 413 and 513 for estimating avg and std

[infer_isize] inferred external isize from 206592 pairs: 463.214 +/- 13.982

[infer_isize] skewness: -0.001; kurtosis: -0.152; ap_prior: 1.77e-05

[infer_isize] inferred maximum insert size: 561 (6.96 sigma)

[bwa_sai2sam_pe_core] time elapses: 150.95 sec

[bwa_sai2sam_pe_core] changing coordinates of 6044 alignments.

[bwa_sai2sam_pe_core] align unmapped mate...

[bwa_paired_sw] 8810 out of 9352 Q17 singletons are mated.

[bwa_paired_sw] 921 out of 1557 Q17 discordant pairs are fixed.

[bwa_sai2sam_pe_core] time elapses: 21.82 sec

[bwa_sai2sam_pe_core] refine gapped alignments... 5.84 sec

[bwa_sai2sam_pe_core] print alignments... 12.65 sec

[bwa_sai2sam_pe_core] 71827456 sequences have been processed.

[bwa_sai2sam_pe_core] convert to sequence coordinate...

[infer_isize] (25, 50, 75) percentile: (453, 463, 473)

[infer_isize] low and high boundaries: 413 and 513 for estimating avg and std

[infer_isize] inferred external isize from 206652 pairs: 463.233 +/- 13.933

[infer_isize] skewness: 0.010; kurtosis: -0.134; ap_prior: 1.65e-05

[infer_isize] inferred maximum insert size: 560 (6.96 sigma)

[bwa_sai2sam_pe_core] time elapses: 104.75 sec

[bwa_sai2sam_pe_core] changing coordinates of 6076 alignments.

[bwa_sai2sam_pe_core] align unmapped mate...

[bwa_paired_sw] 9002 out of 9531 Q17 singletons are mated.

[bwa_paired_sw] 925 out of 1567 Q17 discordant pairs are fixed.

[bwa_sai2sam_pe_core] time elapses: 21.73 sec

[bwa_sai2sam_pe_core] refine gapped alignments... 4.46 sec

[bwa_sai2sam_pe_core] print alignments... 11.79 sec

[bwa_sai2sam_pe_core] 72089600 sequences have been processed.

[bwa_sai2sam_pe_core] convert to sequence coordinate...

[infer_isize] (25, 50, 75) percentile: (453, 463, 473)

[infer_isize] low and high boundaries: 413 and 513 for estimating avg and std

[infer_isize] inferred external isize from 206757 pairs: 463.228 +/- 13.955

[infer_isize] skewness: 0.010; kurtosis: -0.142; ap_prior: 1.74e-05

[infer_isize] inferred maximum insert size: 560 (6.96 sigma)

[bwa_sai2sam_pe_core] time elapses: 71.85 sec

[bwa_sai2sam_pe_core] changing coordinates of 6094 alignments.

[bwa_sai2sam_pe_core] align unmapped mate...

[bwa_paired_sw] 8901 out of 9424 Q17 singletons are mated.

[bwa_paired_sw] 915 out of 1493 Q17 discordant pairs are fixed.

[bwa_sai2sam_pe_core] time elapses: 4.08 sec

[bwa_sai2sam_pe_core] refine gapped alignments... 0.90 sec

[bwa_sai2sam_pe_core] print alignments... 2.07 sec

[bwa_sai2sam_pe_core] 72351744 sequences have been processed.

[bwa_sai2sam_pe_core] convert to sequence coordinate...

[infer_isize] (25, 50, 75) percentile: (453, 463, 473)

[infer_isize] low and high boundaries: 413 and 513 for estimating avg and std

[infer_isize] inferred external isize from 206969 pairs: 463.217 +/- 13.969

[infer_isize] skewness: 0.004; kurtosis: -0.162; ap_prior: 1.86e-05

[infer_isize] inferred maximum insert size: 560 (6.96 sigma)

[bwa_sai2sam_pe_core] time elapses: 24.38 sec

[bwa_sai2sam_pe_core] changing coordinates of 6110 alignments.

[bwa_sai2sam_pe_core] align unmapped mate...

[bwa_paired_sw] 8812 out of 9348 Q17 singletons are mated.

[bwa_paired_sw] 895 out of 1517 Q17 discordant pairs are fixed.

[bwa_sai2sam_pe_core] time elapses: 4.02 sec

[bwa_sai2sam_pe_core] refine gapped alignments... 0.86 sec

[bwa_sai2sam_pe_core] print alignments... 2.06 sec

[bwa_sai2sam_pe_core] 72613888 sequences have been processed.

[bwa_sai2sam_pe_core] convert to sequence coordinate...

[infer_isize] (25, 50, 75) percentile: (453, 463, 473)

[infer_isize] low and high boundaries: 413 and 513 for estimating avg and std

[infer_isize] inferred external isize from 206903 pairs: 463.240 +/- 13.931

[infer_isize] skewness: 0.006; kurtosis: -0.137; ap_prior: 1.66e-05

[infer_isize] inferred maximum insert size: 560 (6.96 sigma)

[bwa_sai2sam_pe_core] time elapses: 23.46 sec

[bwa_sai2sam_pe_core] changing coordinates of 6015 alignments.

[bwa_sai2sam_pe_core] align unmapped mate...

[bwa_paired_sw] 8640 out of 9174 Q17 singletons are mated.

[bwa_paired_sw] 875 out of 1500 Q17 discordant pairs are fixed.

[bwa_sai2sam_pe_core] time elapses: 4.00 sec

[bwa_sai2sam_pe_core] refine gapped alignments... 0.88 sec

[bwa_sai2sam_pe_core] print alignments... 2.05 sec

[bwa_sai2sam_pe_core] 72876032 sequences have been processed.

[bwa_sai2sam_pe_core] convert to sequence coordinate...

[infer_isize] (25, 50, 75) percentile: (453, 463, 473)

[infer_isize] low and high boundaries: 413 and 513 for estimating avg and std

[infer_isize] inferred external isize from 207092 pairs: 463.211 +/- 13.972

[infer_isize] skewness: 0.003; kurtosis: -0.137; ap_prior: 1.80e-05

[infer_isize] inferred maximum insert size: 560 (6.96 sigma)

[bwa_sai2sam_pe_core] time elapses: 23.55 sec

[bwa_sai2sam_pe_core] changing coordinates of 5999 alignments.

[bwa_sai2sam_pe_core] align unmapped mate...

[bwa_paired_sw] 8629 out of 9172 Q17 singletons are mated.

[bwa_paired_sw] 897 out of 1494 Q17 discordant pairs are fixed.

[bwa_sai2sam_pe_core] time elapses: 3.95 sec

[bwa_sai2sam_pe_core] refine gapped alignments... 0.86 sec

[bwa_sai2sam_pe_core] print alignments... 2.02 sec

[bwa_sai2sam_pe_core] 73138176 sequences have been processed.

[bwa_sai2sam_pe_core] convert to sequence coordinate...

[infer_isize] (25, 50, 75) percentile: (453, 463, 473)

[infer_isize] low and high boundaries: 413 and 513 for estimating avg and std

[infer_isize] inferred external isize from 206679 pairs: 463.213 +/- 13.957

[infer_isize] skewness: 0.002; kurtosis: -0.135; ap_prior: 1.77e-05

[infer_isize] inferred maximum insert size: 560 (6.96 sigma)

[bwa_sai2sam_pe_core] time elapses: 26.17 sec

[bwa_sai2sam_pe_core] changing coordinates of 6100 alignments.

[bwa_sai2sam_pe_core] align unmapped mate...

[bwa_paired_sw] 8757 out of 9295 Q17 singletons are mated.

[bwa_paired_sw] 896 out of 1497 Q17 discordant pairs are fixed.

[bwa_sai2sam_pe_core] time elapses: 3.96 sec

[bwa_sai2sam_pe_core] refine gapped alignments... 0.84 sec

[bwa_sai2sam_pe_core] print alignments... 2.03 sec

[bwa_sai2sam_pe_core] 73400320 sequences have been processed.

[bwa_sai2sam_pe_core] convert to sequence coordinate...

[infer_isize] (25, 50, 75) percentile: (453, 463, 473)

[infer_isize] low and high boundaries: 413 and 513 for estimating avg and std

[infer_isize] inferred external isize from 206692 pairs: 463.170 +/- 13.965

[infer_isize] skewness: 0.003; kurtosis: -0.156; ap_prior: 1.72e-05

[infer_isize] inferred maximum insert size: 560 (6.96 sigma)

[bwa_sai2sam_pe_core] time elapses: 23.05 sec

[bwa_sai2sam_pe_core] changing coordinates of 6091 alignments.

[bwa_sai2sam_pe_core] align unmapped mate...

[bwa_paired_sw] 8732 out of 9270 Q17 singletons are mated.

[bwa_paired_sw] 921 out of 1515 Q17 discordant pairs are fixed.

[bwa_sai2sam_pe_core] time elapses: 4.03 sec

[bwa_sai2sam_pe_core] refine gapped alignments... 0.90 sec

[bwa_sai2sam_pe_core] print alignments... 2.08 sec

[bwa_sai2sam_pe_core] 73662464 sequences have been processed.

[bwa_sai2sam_pe_core] convert to sequence coordinate...

[infer_isize] (25, 50, 75) percentile: (453, 463, 473)

[infer_isize] low and high boundaries: 413 and 513 for estimating avg and std

[infer_isize] inferred external isize from 206802 pairs: 463.265 +/- 13.938

[infer_isize] skewness: -0.005; kurtosis: -0.142; ap_prior: 1.78e-05

[infer_isize] inferred maximum insert size: 560 (6.96 sigma)

[bwa_sai2sam_pe_core] time elapses: 22.17 sec

[bwa_sai2sam_pe_core] changing coordinates of 6019 alignments.

[bwa_sai2sam_pe_core] align unmapped mate...

[bwa_paired_sw] 8824 out of 9364 Q17 singletons are mated.

[bwa_paired_sw] 880 out of 1522 Q17 discordant pairs are fixed.

[bwa_sai2sam_pe_core] time elapses: 4.01 sec

[bwa_sai2sam_pe_core] refine gapped alignments... 0.87 sec

[bwa_sai2sam_pe_core] print alignments... 2.06 sec

[bwa_sai2sam_pe_core] 73924608 sequences have been processed.

[bwa_sai2sam_pe_core] convert to sequence coordinate...

[infer_isize] (25, 50, 75) percentile: (453, 463, 473)

[infer_isize] low and high boundaries: 413 and 513 for estimating avg and std

[infer_isize] inferred external isize from 206291 pairs: 463.217 +/- 13.939

[infer_isize] skewness: -0.002; kurtosis: -0.128; ap_prior: 1.78e-05

[infer_isize] inferred maximum insert size: 560 (6.96 sigma)

[bwa_sai2sam_pe_core] time elapses: 21.62 sec

[bwa_sai2sam_pe_core] changing coordinates of 6083 alignments.

[bwa_sai2sam_pe_core] align unmapped mate...

[bwa_paired_sw] 9080 out of 9631 Q17 singletons are mated.

[bwa_paired_sw] 866 out of 1510 Q17 discordant pairs are fixed.

[bwa_sai2sam_pe_core] time elapses: 4.06 sec

[bwa_sai2sam_pe_core] refine gapped alignments... 0.85 sec

[bwa_sai2sam_pe_core] print alignments... 2.05 sec

[bwa_sai2sam_pe_core] 74186752 sequences have been processed.

[bwa_sai2sam_pe_core] convert to sequence coordinate...

[infer_isize] (25, 50, 75) percentile: (453, 463, 473)

[infer_isize] low and high boundaries: 413 and 513 for estimating avg and std

[infer_isize] inferred external isize from 206061 pairs: 463.244 +/- 13.962

[infer_isize] skewness: 0.012; kurtosis: -0.145; ap_prior: 1.73e-05

[infer_isize] inferred maximum insert size: 560 (6.96 sigma)

[bwa_sai2sam_pe_core] time elapses: 20.45 sec

[bwa_sai2sam_pe_core] changing coordinates of 6107 alignments.

[bwa_sai2sam_pe_core] align unmapped mate...

[bwa_paired_sw] 8974 out of 9501 Q17 singletons are mated.

[bwa_paired_sw] 870 out of 1467 Q17 discordant pairs are fixed.

[bwa_sai2sam_pe_core] time elapses: 4.02 sec

[bwa_sai2sam_pe_core] refine gapped alignments... 0.87 sec

[bwa_sai2sam_pe_core] print alignments... 2.08 sec

[bwa_sai2sam_pe_core] 74448896 sequences have been processed.

[bwa_sai2sam_pe_core] convert to sequence coordinate...

[infer_isize] (25, 50, 75) percentile: (453, 463, 473)

[infer_isize] low and high boundaries: 413 and 513 for estimating avg and std

[infer_isize] inferred external isize from 206571 pairs: 463.266 +/- 13.942

[infer_isize] skewness: 0.001; kurtosis: -0.130; ap_prior: 1.77e-05

[infer_isize] inferred maximum insert size: 560 (6.96 sigma)

[bwa_sai2sam_pe_core] time elapses: 20.61 sec

[bwa_sai2sam_pe_core] changing coordinates of 6051 alignments.

[bwa_sai2sam_pe_core] align unmapped mate...

[bwa_paired_sw] 9002 out of 9502 Q17 singletons are mated.

[bwa_paired_sw] 830 out of 1405 Q17 discordant pairs are fixed.

[bwa_sai2sam_pe_core] time elapses: 4.03 sec

[bwa_sai2sam_pe_core] refine gapped alignments... 0.85 sec

[bwa_sai2sam_pe_core] print alignments... 2.05 sec

[bwa_sai2sam_pe_core] 74711040 sequences have been processed.

[bwa_sai2sam_pe_core] convert to sequence coordinate...

[infer_isize] (25, 50, 75) percentile: (453, 463, 473)

[infer_isize] low and high boundaries: 413 and 513 for estimating avg and std

[infer_isize] inferred external isize from 206262 pairs: 463.228 +/- 13.956

[infer_isize] skewness: 0.009; kurtosis: -0.143; ap_prior: 1.72e-05

[infer_isize] inferred maximum insert size: 560 (6.96 sigma)

[bwa_sai2sam_pe_core] time elapses: 20.61 sec

[bwa_sai2sam_pe_core] changing coordinates of 6015 alignments.

[bwa_sai2sam_pe_core] align unmapped mate...

[bwa_paired_sw] 9236 out of 9770 Q17 singletons are mated.

[bwa_paired_sw] 900 out of 1517 Q17 discordant pairs are fixed.

[bwa_sai2sam_pe_core] time elapses: 4.15 sec

[bwa_sai2sam_pe_core] refine gapped alignments... 1.11 sec

[bwa_sai2sam_pe_core] print alignments... 2.26 sec

[bwa_sai2sam_pe_core] 74973184 sequences have been processed.

[bwa_sai2sam_pe_core] convert to sequence coordinate...

[infer_isize] (25, 50, 75) percentile: (453, 463, 473)

[infer_isize] low and high boundaries: 413 and 513 for estimating avg and std

[infer_isize] inferred external isize from 206435 pairs: 463.270 +/- 13.930

[infer_isize] skewness: 0.002; kurtosis: -0.116; ap_prior: 1.89e-05

[infer_isize] inferred maximum insert size: 560 (6.96 sigma)

[bwa_sai2sam_pe_core] time elapses: 26.28 sec

[bwa_sai2sam_pe_core] changing coordinates of 6134 alignments.

[bwa_sai2sam_pe_core] align unmapped mate...

[bwa_paired_sw] 9021 out of 9540 Q17 singletons are mated.

[bwa_paired_sw] 920 out of 1534 Q17 discordant pairs are fixed.

[bwa_sai2sam_pe_core] time elapses: 3.95 sec

[bwa_sai2sam_pe_core] refine gapped alignments... 0.88 sec

[bwa_sai2sam_pe_core] print alignments... 2.07 sec

[bwa_sai2sam_pe_core] 75235328 sequences have been processed.

[bwa_sai2sam_pe_core] convert to sequence coordinate...

[infer_isize] (25, 50, 75) percentile: (453, 463, 473)

[infer_isize] low and high boundaries: 413 and 513 for estimating avg and std

[infer_isize] inferred external isize from 206565 pairs: 463.182 +/- 13.951

[infer_isize] skewness: -0.014; kurtosis: -0.136; ap_prior: 1.65e-05

[infer_isize] inferred maximum insert size: 560 (6.96 sigma)

[bwa_sai2sam_pe_core] time elapses: 26.06 sec

[bwa_sai2sam_pe_core] changing coordinates of 6145 alignments.

[bwa_sai2sam_pe_core] align unmapped mate...

[bwa_paired_sw] 9011 out of 9577 Q17 singletons are mated.

[bwa_paired_sw] 825 out of 1460 Q17 discordant pairs are fixed.

[bwa_sai2sam_pe_core] time elapses: 3.92 sec

[bwa_sai2sam_pe_core] refine gapped alignments... 0.91 sec

[bwa_sai2sam_pe_core] print alignments... 2.06 sec

[bwa_sai2sam_pe_core] 75497472 sequences have been processed.

[bwa_sai2sam_pe_core] convert to sequence coordinate...

[infer_isize] (25, 50, 75) percentile: (453, 463, 473)

[infer_isize] low and high boundaries: 413 and 513 for estimating avg and std

[infer_isize] inferred external isize from 206252 pairs: 463.209 +/- 13.946

[infer_isize] skewness: 0.005; kurtosis: -0.135; ap_prior: 1.70e-05

[infer_isize] inferred maximum insert size: 560 (6.96 sigma)

[bwa_sai2sam_pe_core] time elapses: 33.71 sec

[bwa_sai2sam_pe_core] changing coordinates of 5972 alignments.

[bwa_sai2sam_pe_core] align unmapped mate...

[bwa_paired_sw] 9031 out of 9604 Q17 singletons are mated.

[bwa_paired_sw] 881 out of 1491 Q17 discordant pairs are fixed.

[bwa_sai2sam_pe_core] time elapses: 4.08 sec

[bwa_sai2sam_pe_core] refine gapped alignments... 0.94 sec

[bwa_sai2sam_pe_core] print alignments... 2.11 sec

[bwa_sai2sam_pe_core] 75759616 sequences have been processed.

[bwa_sai2sam_pe_core] convert to sequence coordinate...

[infer_isize] (25, 50, 75) percentile: (453, 463, 473)

[infer_isize] low and high boundaries: 413 and 513 for estimating avg and std

[infer_isize] inferred external isize from 206117 pairs: 463.210 +/- 13.918

[infer_isize] skewness: 0.005; kurtosis: -0.129; ap_prior: 1.76e-05

[infer_isize] inferred maximum insert size: 560 (6.96 sigma)

[bwa_sai2sam_pe_core] time elapses: 35.38 sec

[bwa_sai2sam_pe_core] changing coordinates of 6103 alignments.

[bwa_sai2sam_pe_core] align unmapped mate...

[bwa_paired_sw] 9193 out of 9753 Q17 singletons are mated.

[bwa_paired_sw] 896 out of 1516 Q17 discordant pairs are fixed.

[bwa_sai2sam_pe_core] time elapses: 4.03 sec

[bwa_sai2sam_pe_core] refine gapped alignments... 0.91 sec

[bwa_sai2sam_pe_core] print alignments... 2.13 sec

[bwa_sai2sam_pe_core] 76021760 sequences have been processed.

[bwa_sai2sam_pe_core] convert to sequence coordinate...

[infer_isize] (25, 50, 75) percentile: (453, 463, 473)

[infer_isize] low and high boundaries: 413 and 513 for estimating avg and std

[infer_isize] inferred external isize from 206468 pairs: 463.189 +/- 13.964

[infer_isize] skewness: -0.002; kurtosis: -0.133; ap_prior: 1.86e-05

[infer_isize] inferred maximum insert size: 560 (6.96 sigma)

[bwa_sai2sam_pe_core] time elapses: 38.35 sec

[bwa_sai2sam_pe_core] changing coordinates of 6165 alignments.

[bwa_sai2sam_pe_core] align unmapped mate...

[bwa_paired_sw] 9099 out of 9649 Q17 singletons are mated.

[bwa_paired_sw] 906 out of 1476 Q17 discordant pairs are fixed.

[bwa_sai2sam_pe_core] time elapses: 3.98 sec

[bwa_sai2sam_pe_core] refine gapped alignments... 0.93 sec

[bwa_sai2sam_pe_core] print alignments... 2.07 sec

[bwa_sai2sam_pe_core] 76283904 sequences have been processed.

[bwa_sai2sam_pe_core] convert to sequence coordinate...

[infer_isize] (25, 50, 75) percentile: (453, 463, 473)

[infer_isize] low and high boundaries: 413 and 513 for estimating avg and std

[infer_isize] inferred external isize from 205892 pairs: 463.193 +/- 13.951

[infer_isize] skewness: 0.003; kurtosis: -0.150; ap_prior: 1.67e-05

[infer_isize] inferred maximum insert size: 560 (6.96 sigma)

[bwa_sai2sam_pe_core] time elapses: 54.07 sec

[bwa_sai2sam_pe_core] changing coordinates of 6088 alignments.

[bwa_sai2sam_pe_core] align unmapped mate...

[bwa_paired_sw] 9317 out of 9836 Q17 singletons are mated.

[bwa_paired_sw] 893 out of 1541 Q17 discordant pairs are fixed.

[bwa_sai2sam_pe_core] time elapses: 4.04 sec

[bwa_sai2sam_pe_core] refine gapped alignments... 0.91 sec

[bwa_sai2sam_pe_core] print alignments... 2.12 sec

[bwa_sai2sam_pe_core] 76546048 sequences have been processed.

[bwa_sai2sam_pe_core] convert to sequence coordinate...

[infer_isize] (25, 50, 75) percentile: (453, 463, 473)

[infer_isize] low and high boundaries: 413 and 513 for estimating avg and std

[infer_isize] inferred external isize from 205756 pairs: 463.139 +/- 13.928

[infer_isize] skewness: 0.012; kurtosis: -0.126; ap_prior: 1.75e-05

[infer_isize] inferred maximum insert size: 560 (6.96 sigma)

[bwa_sai2sam_pe_core] time elapses: 33.24 sec

[bwa_sai2sam_pe_core] changing coordinates of 6144 alignments.

[bwa_sai2sam_pe_core] align unmapped mate...

[bwa_paired_sw] 9281 out of 9854 Q17 singletons are mated.

[bwa_paired_sw] 885 out of 1482 Q17 discordant pairs are fixed.

[bwa_sai2sam_pe_core] time elapses: 4.02 sec

[bwa_sai2sam_pe_core] refine gapped alignments... 0.90 sec

[bwa_sai2sam_pe_core] print alignments... 2.08 sec

[bwa_sai2sam_pe_core] 76808192 sequences have been processed.

[bwa_sai2sam_pe_core] convert to sequence coordinate...

[infer_isize] (25, 50, 75) percentile: (453, 463, 473)

[infer_isize] low and high boundaries: 413 and 513 for estimating avg and std

[infer_isize] inferred external isize from 206156 pairs: 463.208 +/- 13.951

[infer_isize] skewness: 0.004; kurtosis: -0.139; ap_prior: 1.90e-05

[infer_isize] inferred maximum insert size: 560 (6.96 sigma)

[bwa_sai2sam_pe_core] time elapses: 23.65 sec

[bwa_sai2sam_pe_core] changing coordinates of 6049 alignments.

[bwa_sai2sam_pe_core] align unmapped mate...

[bwa_paired_sw] 9279 out of 9821 Q17 singletons are mated.

[bwa_paired_sw] 950 out of 1560 Q17 discordant pairs are fixed.

[bwa_sai2sam_pe_core] time elapses: 4.05 sec

[bwa_sai2sam_pe_core] refine gapped alignments... 0.91 sec

[bwa_sai2sam_pe_core] print alignments... 2.07 sec

[bwa_sai2sam_pe_core] 77070336 sequences have been processed.

[bwa_sai2sam_pe_core] convert to sequence coordinate...

[infer_isize] (25, 50, 75) percentile: (453, 463, 473)

[infer_isize] low and high boundaries: 413 and 513 for estimating avg and std

[infer_isize] inferred external isize from 205676 pairs: 463.218 +/- 13.954

[infer_isize] skewness: 0.013; kurtosis: -0.127; ap_prior: 1.48e-05

[infer_isize] inferred maximum insert size: 560 (6.96 sigma)

[bwa_sai2sam_pe_core] time elapses: 23.07 sec

[bwa_sai2sam_pe_core] changing coordinates of 6047 alignments.

[bwa_sai2sam_pe_core] align unmapped mate...

[bwa_paired_sw] 9520 out of 10062 Q17 singletons are mated.

[bwa_paired_sw] 848 out of 1424 Q17 discordant pairs are fixed.

[bwa_sai2sam_pe_core] time elapses: 4.04 sec

[bwa_sai2sam_pe_core] refine gapped alignments... 0.91 sec

[bwa_sai2sam_pe_core] print alignments... 2.07 sec

[bwa_sai2sam_pe_core] 77332480 sequences have been processed.

[bwa_sai2sam_pe_core] convert to sequence coordinate...

[infer_isize] (25, 50, 75) percentile: (453, 463, 473)

[infer_isize] low and high boundaries: 413 and 513 for estimating avg and std

[infer_isize] inferred external isize from 206162 pairs: 463.194 +/- 13.932

[infer_isize] skewness: 0.011; kurtosis: -0.125; ap_prior: 1.76e-05

[infer_isize] inferred maximum insert size: 560 (6.96 sigma)

[bwa_sai2sam_pe_core] time elapses: 22.91 sec

[bwa_sai2sam_pe_core] changing coordinates of 6103 alignments.

[bwa_sai2sam_pe_core] align unmapped mate...

[bwa_paired_sw] 9337 out of 9892 Q17 singletons are mated.

[bwa_paired_sw] 939 out of 1558 Q17 discordant pairs are fixed.

[bwa_sai2sam_pe_core] time elapses: 4.05 sec

[bwa_sai2sam_pe_core] refine gapped alignments... 0.90 sec

[bwa_sai2sam_pe_core] print alignments... 2.10 sec

[bwa_sai2sam_pe_core] 77594624 sequences have been processed.

[bwa_sai2sam_pe_core] convert to sequence coordinate...

[infer_isize] (25, 50, 75) percentile: (453, 463, 473)

[infer_isize] low and high boundaries: 413 and 513 for estimating avg and std

[infer_isize] inferred external isize from 205621 pairs: 463.214 +/- 13.937

[infer_isize] skewness: 0.004; kurtosis: -0.140; ap_prior: 1.69e-05

[infer_isize] inferred maximum insert size: 560 (6.96 sigma)

[bwa_sai2sam_pe_core] time elapses: 23.34 sec

[bwa_sai2sam_pe_core] changing coordinates of 6106 alignments.

[bwa_sai2sam_pe_core] align unmapped mate...

[bwa_paired_sw] 9500 out of 10037 Q17 singletons are mated.

[bwa_paired_sw] 880 out of 1441 Q17 discordant pairs are fixed.

[bwa_sai2sam_pe_core] time elapses: 4.04 sec

[bwa_sai2sam_pe_core] refine gapped alignments... 0.92 sec

[bwa_sai2sam_pe_core] print alignments... 2.08 sec

[bwa_sai2sam_pe_core] 77856768 sequences have been processed.

[bwa_sai2sam_pe_core] convert to sequence coordinate...

[infer_isize] (25, 50, 75) percentile: (453, 463, 473)

[infer_isize] low and high boundaries: 413 and 513 for estimating avg and std

[infer_isize] inferred external isize from 205602 pairs: 463.199 +/- 13.944

[infer_isize] skewness: 0.001; kurtosis: -0.130; ap_prior: 1.66e-05

[infer_isize] inferred maximum insert size: 560 (6.96 sigma)

[bwa_sai2sam_pe_core] time elapses: 22.94 sec

[bwa_sai2sam_pe_core] changing coordinates of 6017 alignments.

[bwa_sai2sam_pe_core] align unmapped mate...

[bwa_paired_sw] 9626 out of 10220 Q17 singletons are mated.

[bwa_paired_sw] 850 out of 1415 Q17 discordant pairs are fixed.

[bwa_sai2sam_pe_core] time elapses: 4.08 sec

[bwa_sai2sam_pe_core] refine gapped alignments... 0.92 sec

[bwa_sai2sam_pe_core] print alignments... 2.07 sec

[bwa_sai2sam_pe_core] 78118912 sequences have been processed.

[bwa_sai2sam_pe_core] convert to sequence coordinate...

[infer_isize] (25, 50, 75) percentile: (453, 463, 473)

[infer_isize] low and high boundaries: 413 and 513 for estimating avg and std

[infer_isize] inferred external isize from 205086 pairs: 463.170 +/- 13.899

[infer_isize] skewness: 0.007; kurtosis: -0.134; ap_prior: 1.59e-05

[infer_isize] inferred maximum insert size: 560 (6.96 sigma)

[bwa_sai2sam_pe_core] time elapses: 23.10 sec

[bwa_sai2sam_pe_core] changing coordinates of 6112 alignments.

[bwa_sai2sam_pe_core] align unmapped mate...

[bwa_paired_sw] 9661 out of 10210 Q17 singletons are mated.

[bwa_paired_sw] 883 out of 1474 Q17 discordant pairs are fixed.

[bwa_sai2sam_pe_core] time elapses: 4.18 sec

[bwa_sai2sam_pe_core] refine gapped alignments... 0.95 sec

[bwa_sai2sam_pe_core] print alignments... 2.17 sec

[bwa_sai2sam_pe_core] 78381056 sequences have been processed.

[bwa_sai2sam_pe_core] convert to sequence coordinate...

[infer_isize] (25, 50, 75) percentile: (453, 463, 473)

[infer_isize] low and high boundaries: 413 and 513 for estimating avg and std

[infer_isize] inferred external isize from 205667 pairs: 463.172 +/- 13.930

[infer_isize] skewness: 0.005; kurtosis: -0.144; ap_prior: 1.71e-05

[infer_isize] inferred maximum insert size: 560 (6.96 sigma)

[bwa_sai2sam_pe_core] time elapses: 30.86 sec

[bwa_sai2sam_pe_core] changing coordinates of 6074 alignments.

[bwa_sai2sam_pe_core] align unmapped mate...

[bwa_paired_sw] 9715 out of 10253 Q17 singletons are mated.

[bwa_paired_sw] 841 out of 1436 Q17 discordant pairs are fixed.

[bwa_sai2sam_pe_core] time elapses: 4.20 sec

[bwa_sai2sam_pe_core] refine gapped alignments... 0.96 sec

[bwa_sai2sam_pe_core] print alignments... 2.18 sec

[bwa_sai2sam_pe_core] 78643200 sequences have been processed.

[bwa_sai2sam_pe_core] convert to sequence coordinate...

[infer_isize] (25, 50, 75) percentile: (453, 463, 473)

[infer_isize] low and high boundaries: 413 and 513 for estimating avg and std

[infer_isize] inferred external isize from 206103 pairs: 463.204 +/- 13.919

[infer_isize] skewness: 0.014; kurtosis: -0.131; ap_prior: 1.63e-05

[infer_isize] inferred maximum insert size: 560 (6.96 sigma)

[bwa_sai2sam_pe_core] time elapses: 35.91 sec

[bwa_sai2sam_pe_core] changing coordinates of 5831 alignments.

[bwa_sai2sam_pe_core] align unmapped mate...

[bwa_paired_sw] 9757 out of 10298 Q17 singletons are mated.

[bwa_paired_sw] 876 out of 1494 Q17 discordant pairs are fixed.

[bwa_sai2sam_pe_core] time elapses: 4.17 sec

[bwa_sai2sam_pe_core] refine gapped alignments... 0.96 sec

[bwa_sai2sam_pe_core] print alignments... 2.16 sec

[bwa_sai2sam_pe_core] 78905344 sequences have been processed.

[bwa_sai2sam_pe_core] convert to sequence coordinate...

[infer_isize] (25, 50, 75) percentile: (453, 463, 473)

[infer_isize] low and high boundaries: 413 and 513 for estimating avg and std

[infer_isize] inferred external isize from 205233 pairs: 463.192 +/- 13.928

[infer_isize] skewness: 0.001; kurtosis: -0.145; ap_prior: 1.77e-05

[infer_isize] inferred maximum insert size: 560 (6.96 sigma)

[bwa_sai2sam_pe_core] time elapses: 34.63 sec

[bwa_sai2sam_pe_core] changing coordinates of 6131 alignments.

[bwa_sai2sam_pe_core] align unmapped mate...

[bwa_paired_sw] 9862 out of 10395 Q17 singletons are mated.

[bwa_paired_sw] 854 out of 1465 Q17 discordant pairs are fixed.

[bwa_sai2sam_pe_core] time elapses: 4.41 sec

[bwa_sai2sam_pe_core] refine gapped alignments... 1.00 sec

[bwa_sai2sam_pe_core] print alignments... 2.10 sec

[bwa_sai2sam_pe_core] 79167488 sequences have been processed.

[bwa_sai2sam_pe_core] convert to sequence coordinate...

[infer_isize] (25, 50, 75) percentile: (453, 463, 473)

[infer_isize] low and high boundaries: 413 and 513 for estimating avg and std

[infer_isize] inferred external isize from 205500 pairs: 463.153 +/- 13.975

[infer_isize] skewness: -0.008; kurtosis: -0.126; ap_prior: 1.66e-05

[infer_isize] inferred maximum insert size: 560 (6.96 sigma)

[bwa_sai2sam_pe_core] time elapses: 92.44 sec

[bwa_sai2sam_pe_core] changing coordinates of 6091 alignments.

[bwa_sai2sam_pe_core] align unmapped mate...

[bwa_paired_sw] 9859 out of 10409 Q17 singletons are mated.

[bwa_paired_sw] 858 out of 1392 Q17 discordant pairs are fixed.

[bwa_sai2sam_pe_core] time elapses: 4.16 sec

[bwa_sai2sam_pe_core] refine gapped alignments... 0.95 sec

[bwa_sai2sam_pe_core] print alignments... 2.18 sec

[bwa_sai2sam_pe_core] 79429632 sequences have been processed.

[bwa_sai2sam_pe_core] convert to sequence coordinate...

[infer_isize] (25, 50, 75) percentile: (453, 463, 473)

[infer_isize] low and high boundaries: 413 and 513 for estimating avg and std

[infer_isize] inferred external isize from 205197 pairs: 463.234 +/- 13.947

[infer_isize] skewness: 0.002; kurtosis: -0.119; ap_prior: 1.72e-05

[infer_isize] inferred maximum insert size: 560 (6.96 sigma)

[bwa_sai2sam_pe_core] time elapses: 29.64 sec

[bwa_sai2sam_pe_core] changing coordinates of 5893 alignments.

[bwa_sai2sam_pe_core] align unmapped mate...

[bwa_paired_sw] 9968 out of 10527 Q17 singletons are mated.

[bwa_paired_sw] 866 out of 1465 Q17 discordant pairs are fixed.

[bwa_sai2sam_pe_core] time elapses: 4.11 sec

[bwa_sai2sam_pe_core] refine gapped alignments... 0.95 sec

[bwa_sai2sam_pe_core] print alignments... 2.14 sec

[bwa_sai2sam_pe_core] 79691776 sequences have been processed.

[bwa_sai2sam_pe_core] convert to sequence coordinate...

[infer_isize] (25, 50, 75) percentile: (453, 463, 473)

[infer_isize] low and high boundaries: 413 and 513 for estimating avg and std

[infer_isize] inferred external isize from 205328 pairs: 463.182 +/- 13.947

[infer_isize] skewness: -0.002; kurtosis: -0.142; ap_prior: 1.75e-05

[infer_isize] inferred maximum insert size: 560 (6.96 sigma)

[bwa_sai2sam_pe_core] time elapses: 24.83 sec

[bwa_sai2sam_pe_core] changing coordinates of 5833 alignments.

[bwa_sai2sam_pe_core] align unmapped mate...

[bwa_paired_sw] 10000 out of 10523 Q17 singletons are mated.

[bwa_paired_sw] 897 out of 1481 Q17 discordant pairs are fixed.

[bwa_sai2sam_pe_core] time elapses: 4.13 sec

[bwa_sai2sam_pe_core] refine gapped alignments... 0.90 sec

[bwa_sai2sam_pe_core] print alignments... 2.10 sec

[bwa_sai2sam_pe_core] 79953920 sequences have been processed.

[bwa_sai2sam_pe_core] convert to sequence coordinate...

[infer_isize] (25, 50, 75) percentile: (453, 463, 473)

[infer_isize] low and high boundaries: 413 and 513 for estimating avg and std

[infer_isize] inferred external isize from 204817 pairs: 463.172 +/- 13.946

[infer_isize] skewness: 0.003; kurtosis: -0.134; ap_prior: 1.78e-05

[infer_isize] inferred maximum insert size: 560 (6.96 sigma)

[bwa_sai2sam_pe_core] time elapses: 24.01 sec

[bwa_sai2sam_pe_core] changing coordinates of 6110 alignments.

[bwa_sai2sam_pe_core] align unmapped mate...

[bwa_paired_sw] 10101 out of 10661 Q17 singletons are mated.

[bwa_paired_sw] 907 out of 1507 Q17 discordant pairs are fixed.

[bwa_sai2sam_pe_core] time elapses: 4.16 sec

[bwa_sai2sam_pe_core] refine gapped alignments... 0.91 sec

[bwa_sai2sam_pe_core] print alignments... 2.11 sec

[bwa_sai2sam_pe_core] 80216064 sequences have been processed.

[bwa_sai2sam_pe_core] convert to sequence coordinate...

[infer_isize] (25, 50, 75) percentile: (453, 463, 473)

[infer_isize] low and high boundaries: 413 and 513 for estimating avg and std

[infer_isize] inferred external isize from 204873 pairs: 463.187 +/- 13.919

[infer_isize] skewness: 0.007; kurtosis: -0.128; ap_prior: 1.90e-05

[infer_isize] inferred maximum insert size: 560 (6.96 sigma)

[bwa_sai2sam_pe_core] time elapses: 20.71 sec

[bwa_sai2sam_pe_core] changing coordinates of 6068 alignments.

[bwa_sai2sam_pe_core] align unmapped mate...

[bwa_paired_sw] 10385 out of 10977 Q17 singletons are mated.

[bwa_paired_sw] 852 out of 1446 Q17 discordant pairs are fixed.

[bwa_sai2sam_pe_core] time elapses: 4.21 sec

[bwa_sai2sam_pe_core] refine gapped alignments... 0.91 sec

[bwa_sai2sam_pe_core] print alignments... 2.12 sec

[bwa_sai2sam_pe_core] 80478208 sequences have been processed.

[bwa_sai2sam_pe_core] convert to sequence coordinate...

[infer_isize] (25, 50, 75) percentile: (453, 463, 473)

[infer_isize] low and high boundaries: 413 and 513 for estimating avg and std

[infer_isize] inferred external isize from 204593 pairs: 463.153 +/- 13.958

[infer_isize] skewness: -0.003; kurtosis: -0.126; ap_prior: 1.60e-05

[infer_isize] inferred maximum insert size: 560 (6.96 sigma)

[bwa_sai2sam_pe_core] time elapses: 20.59 sec

[bwa_sai2sam_pe_core] changing coordinates of 5908 alignments.

[bwa_sai2sam_pe_core] align unmapped mate...

[bwa_paired_sw] 10511 out of 11079 Q17 singletons are mated.

[bwa_paired_sw] 865 out of 1441 Q17 discordant pairs are fixed.

[bwa_sai2sam_pe_core] time elapses: 4.24 sec

[bwa_sai2sam_pe_core] refine gapped alignments... 0.92 sec

[bwa_sai2sam_pe_core] print alignments... 2.11 sec

[bwa_sai2sam_pe_core] 80740352 sequences have been processed.

[bwa_sai2sam_pe_core] convert to sequence coordinate...

[infer_isize] (25, 50, 75) percentile: (453, 463, 473)

[infer_isize] low and high boundaries: 413 and 513 for estimating avg and std

[infer_isize] inferred external isize from 204609 pairs: 463.151 +/- 13.938

[infer_isize] skewness: -0.002; kurtosis: -0.137; ap_prior: 1.79e-05

[infer_isize] inferred maximum insert size: 560 (6.96 sigma)

[bwa_sai2sam_pe_core] time elapses: 20.93 sec

[bwa_sai2sam_pe_core] changing coordinates of 6004 alignments.

[bwa_sai2sam_pe_core] align unmapped mate...

[bwa_paired_sw] 10417 out of 10947 Q17 singletons are mated.

[bwa_paired_sw] 874 out of 1503 Q17 discordant pairs are fixed.

[bwa_sai2sam_pe_core] time elapses: 4.25 sec

[bwa_sai2sam_pe_core] refine gapped alignments... 1.61 sec

[bwa_sai2sam_pe_core] print alignments... 10.94 sec

[bwa_sai2sam_pe_core] 81002496 sequences have been processed.

[bwa_sai2sam_pe_core] convert to sequence coordinate...

[infer_isize] (25, 50, 75) percentile: (453, 463, 473)

[infer_isize] low and high boundaries: 413 and 513 for estimating avg and std

[infer_isize] inferred external isize from 204052 pairs: 463.178 +/- 13.925

[infer_isize] skewness: 0.005; kurtosis: -0.150; ap_prior: 1.70e-05

[infer_isize] inferred maximum insert size: 560 (6.96 sigma)

[bwa_sai2sam_pe_core] time elapses: 92.70 sec

[bwa_sai2sam_pe_core] changing coordinates of 5965 alignments.

[bwa_sai2sam_pe_core] align unmapped mate...

[bwa_paired_sw] 11124 out of 11689 Q17 singletons are mated.

[bwa_paired_sw] 862 out of 1466 Q17 discordant pairs are fixed.

[bwa_sai2sam_pe_core] time elapses: 23.07 sec

[bwa_sai2sam_pe_core] refine gapped alignments... 5.01 sec

[bwa_sai2sam_pe_core] print alignments... 11.38 sec

[bwa_sai2sam_pe_core] 81264640 sequences have been processed.

[bwa_sai2sam_pe_core] convert to sequence coordinate...

[infer_isize] (25, 50, 75) percentile: (453, 463, 473)

[infer_isize] low and high boundaries: 413 and 513 for estimating avg and std

[infer_isize] inferred external isize from 204165 pairs: 463.193 +/- 13.918

[infer_isize] skewness: 0.005; kurtosis: -0.138; ap_prior: 1.74e-05

[infer_isize] inferred maximum insert size: 560 (6.96 sigma)

[bwa_sai2sam_pe_core] time elapses: 91.20 sec

[bwa_sai2sam_pe_core] changing coordinates of 6005 alignments.

[bwa_sai2sam_pe_core] align unmapped mate...

[bwa_paired_sw] 10721 out of 11279 Q17 singletons are mated.

[bwa_paired_sw] 883 out of 1462 Q17 discordant pairs are fixed.

[bwa_sai2sam_pe_core] time elapses: 21.70 sec

[bwa_sai2sam_pe_core] refine gapped alignments... 4.58 sec

[bwa_sai2sam_pe_core] print alignments... 11.07 sec

[bwa_sai2sam_pe_core] 81526784 sequences have been processed.

[bwa_sai2sam_pe_core] convert to sequence coordinate...

[infer_isize] (25, 50, 75) percentile: (453, 463, 473)

[infer_isize] low and high boundaries: 413 and 513 for estimating avg and std

[infer_isize] inferred external isize from 204092 pairs: 463.105 +/- 13.948

[infer_isize] skewness: 0.010; kurtosis: -0.134; ap_prior: 1.74e-05

[infer_isize] inferred maximum insert size: 560 (6.96 sigma)

[bwa_sai2sam_pe_core] time elapses: 93.70 sec

[bwa_sai2sam_pe_core] changing coordinates of 5999 alignments.

[bwa_sai2sam_pe_core] align unmapped mate...

[bwa_paired_sw] 10969 out of 11523 Q17 singletons are mated.

[bwa_paired_sw] 885 out of 1464 Q17 discordant pairs are fixed.

[bwa_sai2sam_pe_core] time elapses: 20.92 sec

[bwa_sai2sam_pe_core] refine gapped alignments... 3.92 sec

[bwa_sai2sam_pe_core] print alignments... 10.10 sec

[bwa_sai2sam_pe_core] 81788928 sequences have been processed.

[bwa_sai2sam_pe_core] convert to sequence coordinate...

[infer_isize] (25, 50, 75) percentile: (453, 463, 473)

[infer_isize] low and high boundaries: 413 and 513 for estimating avg and std

[infer_isize] inferred external isize from 203729 pairs: 463.132 +/- 13.899

[infer_isize] skewness: 0.008; kurtosis: -0.139; ap_prior: 1.75e-05

[infer_isize] inferred maximum insert size: 560 (6.96 sigma)

[bwa_sai2sam_pe_core] time elapses: 93.12 sec

[bwa_sai2sam_pe_core] changing coordinates of 5963 alignments.

[bwa_sai2sam_pe_core] align unmapped mate...

[bwa_paired_sw] 11130 out of 11695 Q17 singletons are mated.

[bwa_paired_sw] 899 out of 1498 Q17 discordant pairs are fixed.

[bwa_sai2sam_pe_core] time elapses: 22.23 sec

[bwa_sai2sam_pe_core] refine gapped alignments... 5.26 sec

[bwa_sai2sam_pe_core] print alignments... 10.23 sec

[bwa_sai2sam_pe_core] 82051072 sequences have been processed.

[bwa_sai2sam_pe_core] convert to sequence coordinate...

[infer_isize] (25, 50, 75) percentile: (453, 463, 473)

[infer_isize] low and high boundaries: 413 and 513 for estimating avg and std

[infer_isize] inferred external isize from 203562 pairs: 463.101 +/- 13.952

[infer_isize] skewness: 0.000; kurtosis: -0.145; ap_prior: 1.56e-05

[infer_isize] inferred maximum insert size: 560 (6.96 sigma)

[bwa_sai2sam_pe_core] time elapses: 31.16 sec

[bwa_sai2sam_pe_core] changing coordinates of 6021 alignments.

[bwa_sai2sam_pe_core] align unmapped mate...

[bwa_paired_sw] 11339 out of 11895 Q17 singletons are mated.

[bwa_paired_sw] 875 out of 1425 Q17 discordant pairs are fixed.

[bwa_sai2sam_pe_core] time elapses: 4.48 sec

[bwa_sai2sam_pe_core] refine gapped alignments... 0.96 sec

[bwa_sai2sam_pe_core] print alignments... 2.08 sec

[bwa_sai2sam_pe_core] 82313216 sequences have been processed.

[bwa_sai2sam_pe_core] convert to sequence coordinate...

[infer_isize] (25, 50, 75) percentile: (453, 463, 473)

[infer_isize] low and high boundaries: 413 and 513 for estimating avg and std

[infer_isize] inferred external isize from 203230 pairs: 463.084 +/- 13.975

[infer_isize] skewness: -0.001; kurtosis: -0.119; ap_prior: 1.55e-05

[infer_isize] inferred maximum insert size: 560 (6.96 sigma)

[bwa_sai2sam_pe_core] time elapses: 29.78 sec

[bwa_sai2sam_pe_core] changing coordinates of 5910 alignments.

[bwa_sai2sam_pe_core] align unmapped mate...

[bwa_paired_sw] 11900 out of 12448 Q17 singletons are mated.

[bwa_paired_sw] 845 out of 1413 Q17 discordant pairs are fixed.

[bwa_sai2sam_pe_core] time elapses: 4.70 sec

[bwa_sai2sam_pe_core] refine gapped alignments... 1.00 sec

[bwa_sai2sam_pe_core] print alignments... 2.10 sec

[bwa_sai2sam_pe_core] 82575360 sequences have been processed.

[bwa_sai2sam_pe_core] convert to sequence coordinate...

[infer_isize] (25, 50, 75) percentile: (453, 463, 473)

[infer_isize] low and high boundaries: 413 and 513 for estimating avg and std

[infer_isize] inferred external isize from 203141 pairs: 463.040 +/- 13.911

[infer_isize] skewness: 0.003; kurtosis: -0.122; ap_prior: 1.65e-05

[infer_isize] inferred maximum insert size: 560 (6.96 sigma)

[bwa_sai2sam_pe_core] time elapses: 54.34 sec

[bwa_sai2sam_pe_core] changing coordinates of 6022 alignments.

[bwa_sai2sam_pe_core] align unmapped mate...

[bwa_paired_sw] 11995 out of 12543 Q17 singletons are mated.

[bwa_paired_sw] 875 out of 1435 Q17 discordant pairs are fixed.

[bwa_sai2sam_pe_core] time elapses: 4.67 sec

[bwa_sai2sam_pe_core] refine gapped alignments... 1.00 sec

[bwa_sai2sam_pe_core] print alignments... 2.17 sec

[bwa_sai2sam_pe_core] 82837504 sequences have been processed.

[bwa_sai2sam_pe_core] convert to sequence coordinate...

[infer_isize] (25, 50, 75) percentile: (453, 463, 473)

[infer_isize] low and high boundaries: 413 and 513 for estimating avg and std

[infer_isize] inferred external isize from 202408 pairs: 463.151 +/- 13.931

[infer_isize] skewness: 0.005; kurtosis: -0.141; ap_prior: 1.84e-05

[infer_isize] inferred maximum insert size: 560 (6.96 sigma)

[bwa_sai2sam_pe_core] time elapses: 20.63 sec

[bwa_sai2sam_pe_core] changing coordinates of 6044 alignments.

[bwa_sai2sam_pe_core] align unmapped mate...

[bwa_paired_sw] 12397 out of 12985 Q17 singletons are mated.

[bwa_paired_sw] 938 out of 1510 Q17 discordant pairs are fixed.

[bwa_sai2sam_pe_core] time elapses: 4.80 sec

[bwa_sai2sam_pe_core] refine gapped alignments... 0.97 sec

[bwa_sai2sam_pe_core] print alignments... 2.11 sec

[bwa_sai2sam_pe_core] 83099648 sequences have been processed.

[bwa_sai2sam_pe_core] convert to sequence coordinate...

[infer_isize] (25, 50, 75) percentile: (453, 463, 473)

[infer_isize] low and high boundaries: 413 and 513 for estimating avg and std

[infer_isize] inferred external isize from 202108 pairs: 463.080 +/- 13.976

[infer_isize] skewness: 0.009; kurtosis: -0.119; ap_prior: 1.62e-05

[infer_isize] inferred maximum insert size: 560 (6.96 sigma)

[bwa_sai2sam_pe_core] time elapses: 20.09 sec

[bwa_sai2sam_pe_core] changing coordinates of 5959 alignments.

[bwa_sai2sam_pe_core] align unmapped mate...

[bwa_paired_sw] 12823 out of 13391 Q17 singletons are mated.

[bwa_paired_sw] 865 out of 1408 Q17 discordant pairs are fixed.

[bwa_sai2sam_pe_core] time elapses: 4.85 sec

[bwa_sai2sam_pe_core] refine gapped alignments... 0.99 sec

[bwa_sai2sam_pe_core] print alignments... 2.11 sec

[bwa_sai2sam_pe_core] 83361792 sequences have been processed.

[bwa_sai2sam_pe_core] convert to sequence coordinate...

[infer_isize] (25, 50, 75) percentile: (453, 463, 473)

[infer_isize] low and high boundaries: 413 and 513 for estimating avg and std

[infer_isize] inferred external isize from 202006 pairs: 463.026 +/- 13.918

[infer_isize] skewness: 0.003; kurtosis: -0.119; ap_prior: 1.67e-05

[infer_isize] inferred maximum insert size: 560 (6.96 sigma)

[bwa_sai2sam_pe_core] time elapses: 19.78 sec

[bwa_sai2sam_pe_core] changing coordinates of 6065 alignments.

[bwa_sai2sam_pe_core] align unmapped mate...

[bwa_paired_sw] 13005 out of 13545 Q17 singletons are mated.

[bwa_paired_sw] 903 out of 1495 Q17 discordant pairs are fixed.

[bwa_sai2sam_pe_core] time elapses: 4.93 sec

[bwa_sai2sam_pe_core] refine gapped alignments... 1.00 sec

[bwa_sai2sam_pe_core] print alignments... 2.09 sec

[bwa_sai2sam_pe_core] 83623936 sequences have been processed.

[bwa_sai2sam_pe_core] convert to sequence coordinate...

[infer_isize] (25, 50, 75) percentile: (453, 463, 473)

[infer_isize] low and high boundaries: 413 and 513 for estimating avg and std

[infer_isize] inferred external isize from 201289 pairs: 463.136 +/- 13.951

[infer_isize] skewness: 0.010; kurtosis: -0.140; ap_prior: 2.04e-05

[infer_isize] inferred maximum insert size: 560 (6.96 sigma)

[bwa_sai2sam_pe_core] time elapses: 20.57 sec

[bwa_sai2sam_pe_core] changing coordinates of 5850 alignments.

[bwa_sai2sam_pe_core] align unmapped mate...

[bwa_paired_sw] 13311 out of 13867 Q17 singletons are mated.

[bwa_paired_sw] 920 out of 1469 Q17 discordant pairs are fixed.

[bwa_sai2sam_pe_core] time elapses: 5.03 sec

[bwa_sai2sam_pe_core] refine gapped alignments... 1.01 sec

[bwa_sai2sam_pe_core] print alignments... 2.09 sec

[bwa_sai2sam_pe_core] 83886080 sequences have been processed.

[bwa_sai2sam_pe_core] convert to sequence coordinate...

[infer_isize] (25, 50, 75) percentile: (453, 463, 473)

[infer_isize] low and high boundaries: 413 and 513 for estimating avg and std

[infer_isize] inferred external isize from 201142 pairs: 463.068 +/- 13.967

[infer_isize] skewness: -0.007; kurtosis: -0.134; ap_prior: 1.67e-05

[infer_isize] inferred maximum insert size: 560 (6.96 sigma)

[bwa_sai2sam_pe_core] time elapses: 20.53 sec

[bwa_sai2sam_pe_core] changing coordinates of 6000 alignments.

[bwa_sai2sam_pe_core] align unmapped mate...

[bwa_paired_sw] 13971 out of 14549 Q17 singletons are mated.

[bwa_paired_sw] 876 out of 1435 Q17 discordant pairs are fixed.

[bwa_sai2sam_pe_core] time elapses: 5.17 sec

[bwa_sai2sam_pe_core] refine gapped alignments... 1.03 sec

[bwa_sai2sam_pe_core] print alignments... 2.10 sec

[bwa_sai2sam_pe_core] 84148224 sequences have been processed.

[bwa_sai2sam_pe_core] convert to sequence coordinate...

[infer_isize] (25, 50, 75) percentile: (453, 463, 473)

[infer_isize] low and high boundaries: 413 and 513 for estimating avg and std

[infer_isize] inferred external isize from 200230 pairs: 463.054 +/- 13.923

[infer_isize] skewness: 0.002; kurtosis: -0.135; ap_prior: 1.65e-05

[infer_isize] inferred maximum insert size: 560 (6.96 sigma)

[bwa_sai2sam_pe_core] time elapses: 20.26 sec

[bwa_sai2sam_pe_core] changing coordinates of 5963 alignments.

[bwa_sai2sam_pe_core] align unmapped mate...

[bwa_paired_sw] 14494 out of 15081 Q17 singletons are mated.

[bwa_paired_sw] 887 out of 1466 Q17 discordant pairs are fixed.

[bwa_sai2sam_pe_core] time elapses: 5.35 sec

[bwa_sai2sam_pe_core] refine gapped alignments... 1.03 sec

[bwa_sai2sam_pe_core] print alignments... 2.10 sec

[bwa_sai2sam_pe_core] 84410368 sequences have been processed.

[bwa_sai2sam_pe_core] convert to sequence coordinate...

[infer_isize] (25, 50, 75) percentile: (453, 463, 473)

[infer_isize] low and high boundaries: 413 and 513 for estimating avg and std

[infer_isize] inferred external isize from 199721 pairs: 463.059 +/- 13.922

[infer_isize] skewness: 0.002; kurtosis: -0.150; ap_prior: 1.62e-05

[infer_isize] inferred maximum insert size: 560 (6.96 sigma)

[bwa_sai2sam_pe_core] time elapses: 20.64 sec

[bwa_sai2sam_pe_core] changing coordinates of 5827 alignments.

[bwa_sai2sam_pe_core] align unmapped mate...

[bwa_paired_sw] 15251 out of 15808 Q17 singletons are mated.

[bwa_paired_sw] 866 out of 1415 Q17 discordant pairs are fixed.

[bwa_sai2sam_pe_core] time elapses: 5.50 sec

[bwa_sai2sam_pe_core] refine gapped alignments... 1.06 sec

[bwa_sai2sam_pe_core] print alignments... 2.10 sec

[bwa_sai2sam_pe_core] 84672512 sequences have been processed.

[bwa_sai2sam_pe_core] convert to sequence coordinate...

[infer_isize] (25, 50, 75) percentile: (453, 463, 473)

[infer_isize] low and high boundaries: 413 and 513 for estimating avg and std

[infer_isize] inferred external isize from 198767 pairs: 463.027 +/- 13.922

[infer_isize] skewness: 0.000; kurtosis: -0.130; ap_prior: 1.61e-05

[infer_isize] inferred maximum insert size: 560 (6.96 sigma)

[bwa_sai2sam_pe_core] time elapses: 20.33 sec

[bwa_sai2sam_pe_core] changing coordinates of 5818 alignments.

[bwa_sai2sam_pe_core] align unmapped mate...

[bwa_paired_sw] 15665 out of 16214 Q17 singletons are mated.

[bwa_paired_sw] 857 out of 1390 Q17 discordant pairs are fixed.

[bwa_sai2sam_pe_core] time elapses: 5.61 sec

[bwa_sai2sam_pe_core] refine gapped alignments... 1.09 sec

[bwa_sai2sam_pe_core] print alignments... 2.12 sec

[bwa_sai2sam_pe_core] 84934656 sequences have been processed.

[bwa_sai2sam_pe_core] convert to sequence coordinate...

[infer_isize] (25, 50, 75) percentile: (453, 463, 473)

[infer_isize] low and high boundaries: 413 and 513 for estimating avg and std

[infer_isize] inferred external isize from 198239 pairs: 463.068 +/- 13.915

[infer_isize] skewness: 0.004; kurtosis: -0.151; ap_prior: 1.67e-05

[infer_isize] inferred maximum insert size: 560 (6.96 sigma)

[bwa_sai2sam_pe_core] time elapses: 20.75 sec

[bwa_sai2sam_pe_core] changing coordinates of 5865 alignments.

[bwa_sai2sam_pe_core] align unmapped mate...

[bwa_paired_sw] 16146 out of 16651 Q17 singletons are mated.

[bwa_paired_sw] 913 out of 1467 Q17 discordant pairs are fixed.

[bwa_sai2sam_pe_core] time elapses: 5.77 sec

[bwa_sai2sam_pe_core] refine gapped alignments... 1.09 sec

[bwa_sai2sam_pe_core] print alignments... 2.11 sec

[bwa_sai2sam_pe_core] 85196800 sequences have been processed.

[bwa_sai2sam_pe_core] convert to sequence coordinate...

[infer_isize] (25, 50, 75) percentile: (453, 463, 473)

[infer_isize] low and high boundaries: 413 and 513 for estimating avg and std

[infer_isize] inferred external isize from 197899 pairs: 463.000 +/- 13.955

[infer_isize] skewness: 0.004; kurtosis: -0.122; ap_prior: 1.75e-05

[infer_isize] inferred maximum insert size: 560 (6.96 sigma)

[bwa_sai2sam_pe_core] time elapses: 20.67 sec

[bwa_sai2sam_pe_core] changing coordinates of 5858 alignments.

[bwa_sai2sam_pe_core] align unmapped mate...

[bwa_paired_sw] 16566 out of 17120 Q17 singletons are mated.

[bwa_paired_sw] 883 out of 1405 Q17 discordant pairs are fixed.

[bwa_sai2sam_pe_core] time elapses: 5.89 sec

[bwa_sai2sam_pe_core] refine gapped alignments... 1.14 sec

[bwa_sai2sam_pe_core] print alignments... 2.06 sec

[bwa_sai2sam_pe_core] 85458944 sequences have been processed.

[bwa_sai2sam_pe_core] convert to sequence coordinate...

[infer_isize] (25, 50, 75) percentile: (453, 463, 473)

[infer_isize] low and high boundaries: 413 and 513 for estimating avg and std

[infer_isize] inferred external isize from 197253 pairs: 463.072 +/- 13.975

[infer_isize] skewness: 0.002; kurtosis: -0.148; ap_prior: 1.70e-05

[infer_isize] inferred maximum insert size: 560 (6.96 sigma)

[bwa_sai2sam_pe_core] time elapses: 23.29 sec

[bwa_sai2sam_pe_core] changing coordinates of 5736 alignments.

[bwa_sai2sam_pe_core] align unmapped mate...

[bwa_paired_sw] 17600 out of 18142 Q17 singletons are mated.

[bwa_paired_sw] 895 out of 1423 Q17 discordant pairs are fixed.

[bwa_sai2sam_pe_core] time elapses: 6.16 sec

[bwa_sai2sam_pe_core] refine gapped alignments... 1.16 sec

[bwa_sai2sam_pe_core] print alignments... 2.12 sec

[bwa_sai2sam_pe_core] 85721088 sequences have been processed.

[bwa_sai2sam_pe_core] convert to sequence coordinate...

[infer_isize] (25, 50, 75) percentile: (453, 463, 473)

[infer_isize] low and high boundaries: 413 and 513 for estimating avg and std

[infer_isize] inferred external isize from 195935 pairs: 463.025 +/- 13.923

[infer_isize] skewness: 0.000; kurtosis: -0.134; ap_prior: 1.87e-05

[infer_isize] inferred maximum insert size: 560 (6.96 sigma)

[bwa_sai2sam_pe_core] time elapses: 34.01 sec

[bwa_sai2sam_pe_core] changing coordinates of 5782 alignments.

[bwa_sai2sam_pe_core] align unmapped mate...

[bwa_paired_sw] 18657 out of 19220 Q17 singletons are mated.

[bwa_paired_sw] 849 out of 1361 Q17 discordant pairs are fixed.

[bwa_sai2sam_pe_core] time elapses: 6.73 sec

[bwa_sai2sam_pe_core] refine gapped alignments... 1.27 sec

[bwa_sai2sam_pe_core] print alignments... 2.14 sec

[bwa_sai2sam_pe_core] 85983232 sequences have been processed.

[bwa_sai2sam_pe_core] convert to sequence coordinate...

[infer_isize] (25, 50, 75) percentile: (453, 463, 473)

[infer_isize] low and high boundaries: 413 and 513 for estimating avg and std

[infer_isize] inferred external isize from 195508 pairs: 462.963 +/- 13.976

[infer_isize] skewness: -0.003; kurtosis: -0.120; ap_prior: 1.73e-05

[infer_isize] inferred maximum insert size: 560 (6.96 sigma)

[bwa_sai2sam_pe_core] time elapses: 32.40 sec

[bwa_sai2sam_pe_core] changing coordinates of 5722 alignments.

[bwa_sai2sam_pe_core] align unmapped mate...

[bwa_paired_sw] 19239 out of 19762 Q17 singletons are mated.

[bwa_paired_sw] 916 out of 1407 Q17 discordant pairs are fixed.

[bwa_sai2sam_pe_core] time elapses: 6.65 sec

[bwa_sai2sam_pe_core] refine gapped alignments... 1.24 sec

[bwa_sai2sam_pe_core] print alignments... 2.13 sec

[bwa_sai2sam_pe_core] 86245376 sequences have been processed.

[bwa_sai2sam_pe_core] convert to sequence coordinate...

[infer_isize] (25, 50, 75) percentile: (453, 463, 473)

[infer_isize] low and high boundaries: 413 and 513 for estimating avg and std

[infer_isize] inferred external isize from 194193 pairs: 462.991 +/- 13.990

[infer_isize] skewness: -0.005; kurtosis: -0.122; ap_prior: 1.58e-05

[infer_isize] inferred maximum insert size: 560 (6.96 sigma)

[bwa_sai2sam_pe_core] time elapses: 28.96 sec

[bwa_sai2sam_pe_core] changing coordinates of 5679 alignments.

[bwa_sai2sam_pe_core] align unmapped mate...

[bwa_paired_sw] 20068 out of 20625 Q17 singletons are mated.

[bwa_paired_sw] 896 out of 1409 Q17 discordant pairs are fixed.

[bwa_sai2sam_pe_core] time elapses: 6.83 sec

[bwa_sai2sam_pe_core] refine gapped alignments... 1.25 sec

[bwa_sai2sam_pe_core] print alignments... 2.11 sec

[bwa_sai2sam_pe_core] 86507520 sequences have been processed.

[bwa_sai2sam_pe_core] convert to sequence coordinate...

[infer_isize] (25, 50, 75) percentile: (453, 463, 473)

[infer_isize] low and high boundaries: 413 and 513 for estimating avg and std

[infer_isize] inferred external isize from 191672 pairs: 462.925 +/- 13.974

[infer_isize] skewness: 0.001; kurtosis: -0.137; ap_prior: 1.78e-05

[infer_isize] inferred maximum insert size: 560 (6.96 sigma)

[bwa_sai2sam_pe_core] time elapses: 19.50 sec

[bwa_sai2sam_pe_core] changing coordinates of 5658 alignments.

[bwa_sai2sam_pe_core] align unmapped mate...

[bwa_paired_sw] 22618 out of 23196 Q17 singletons are mated.

[bwa_paired_sw] 870 out of 1399 Q17 discordant pairs are fixed.

[bwa_sai2sam_pe_core] time elapses: 7.51 sec

[bwa_sai2sam_pe_core] refine gapped alignments... 1.25 sec

[bwa_sai2sam_pe_core] print alignments... 2.11 sec

[bwa_sai2sam_pe_core] 86769664 sequences have been processed.

[bwa_sai2sam_pe_core] convert to sequence coordinate...

[infer_isize] (25, 50, 75) percentile: (453, 463, 473)

[infer_isize] low and high boundaries: 413 and 513 for estimating avg and std

[infer_isize] inferred external isize from 192403 pairs: 462.990 +/- 13.949

[infer_isize] skewness: 0.001; kurtosis: -0.135; ap_prior: 1.50e-05

[infer_isize] inferred maximum insert size: 560 (6.96 sigma)

[bwa_sai2sam_pe_core] time elapses: 49.99 sec

[bwa_sai2sam_pe_core] changing coordinates of 5596 alignments.

[bwa_sai2sam_pe_core] align unmapped mate...

[bwa_paired_sw] 21920 out of 22526 Q17 singletons are mated.

[bwa_paired_sw] 861 out of 1346 Q17 discordant pairs are fixed.

[bwa_sai2sam_pe_core] time elapses: 36.15 sec

[bwa_sai2sam_pe_core] refine gapped alignments... 6.70 sec

[bwa_sai2sam_pe_core] print alignments... 11.15 sec

[bwa_sai2sam_pe_core] 87031808 sequences have been processed.

[bwa_sai2sam_pe_core] convert to sequence coordinate...

[infer_isize] (25, 50, 75) percentile: (453, 463, 473)

[infer_isize] low and high boundaries: 413 and 513 for estimating avg and std

[infer_isize] inferred external isize from 191837 pairs: 462.925 +/- 13.960

[infer_isize] skewness: 0.008; kurtosis: -0.122; ap_prior: 1.61e-05

[infer_isize] inferred maximum insert size: 560 (6.96 sigma)

[bwa_sai2sam_pe_core] time elapses: 87.23 sec

[bwa_sai2sam_pe_core] changing coordinates of 5679 alignments.

[bwa_sai2sam_pe_core] align unmapped mate...

[bwa_paired_sw] 22424 out of 22980 Q17 singletons are mated.

[bwa_paired_sw] 915 out of 1391 Q17 discordant pairs are fixed.

[bwa_sai2sam_pe_core] time elapses: 36.41 sec

[bwa_sai2sam_pe_core] refine gapped alignments... 6.58 sec

[bwa_sai2sam_pe_core] print alignments... 11.32 sec

[bwa_sai2sam_pe_core] 87293952 sequences have been processed.

[bwa_sai2sam_pe_core] convert to sequence coordinate...

[infer_isize] (25, 50, 75) percentile: (453, 463, 473)

[infer_isize] low and high boundaries: 413 and 513 for estimating avg and std

[infer_isize] inferred external isize from 191858 pairs: 462.905 +/- 13.935

[infer_isize] skewness: 0.015; kurtosis: -0.132; ap_prior: 1.74e-05

[infer_isize] inferred maximum insert size: 560 (6.96 sigma)

[bwa_sai2sam_pe_core] time elapses: 107.63 sec

[bwa_sai2sam_pe_core] changing coordinates of 5629 alignments.

[bwa_sai2sam_pe_core] align unmapped mate...

[bwa_paired_sw] 22266 out of 22807 Q17 singletons are mated.

[bwa_paired_sw] 898 out of 1419 Q17 discordant pairs are fixed.

[bwa_sai2sam_pe_core] time elapses: 36.86 sec

[bwa_sai2sam_pe_core] refine gapped alignments... 6.33 sec

[bwa_sai2sam_pe_core] print alignments... 11.08 sec

[bwa_sai2sam_pe_core] 87556096 sequences have been processed.

[bwa_sai2sam_pe_core] convert to sequence coordinate...

[infer_isize] (25, 50, 75) percentile: (453, 463, 473)

[infer_isize] low and high boundaries: 413 and 513 for estimating avg and std

[infer_isize] inferred external isize from 205140 pairs: 463.233 +/- 13.977

[infer_isize] skewness: -0.007; kurtosis: -0.145; ap_prior: 1.74e-05

[infer_isize] inferred maximum insert size: 561 (6.96 sigma)

[bwa_sai2sam_pe_core] time elapses: 114.02 sec

[bwa_sai2sam_pe_core] changing coordinates of 6019 alignments.

[bwa_sai2sam_pe_core] align unmapped mate...

[bwa_paired_sw] 10325 out of 10921 Q17 singletons are mated.

[bwa_paired_sw] 860 out of 1514 Q17 discordant pairs are fixed.

[bwa_sai2sam_pe_core] time elapses: 21.24 sec

[bwa_sai2sam_pe_core] refine gapped alignments... 4.39 sec

[bwa_sai2sam_pe_core] print alignments... 5.29 sec

[bwa_sai2sam_pe_core] 87818240 sequences have been processed.

[bwa_sai2sam_pe_core] convert to sequence coordinate...

[infer_isize] (25, 50, 75) percentile: (453, 463, 473)

[infer_isize] low and high boundaries: 413 and 513 for estimating avg and std

[infer_isize] inferred external isize from 204411 pairs: 463.301 +/- 13.918

[infer_isize] skewness: 0.002; kurtosis: -0.140; ap_prior: 1.72e-05

[infer_isize] inferred maximum insert size: 560 (6.96 sigma)

[bwa_sai2sam_pe_core] time elapses: 25.68 sec

[bwa_sai2sam_pe_core] changing coordinates of 5913 alignments.

[bwa_sai2sam_pe_core] align unmapped mate...

[bwa_paired_sw] 10951 out of 11529 Q17 singletons are mated.

[bwa_paired_sw] 871 out of 1463 Q17 discordant pairs are fixed.

[bwa_sai2sam_pe_core] time elapses: 4.38 sec

[bwa_sai2sam_pe_core] refine gapped alignments... 0.91 sec

[bwa_sai2sam_pe_core] print alignments... 2.11 sec

[bwa_sai2sam_pe_core] 88080384 sequences have been processed.

[bwa_sai2sam_pe_core] convert to sequence coordinate...

[infer_isize] (25, 50, 75) percentile: (453, 463, 473)

[infer_isize] low and high boundaries: 413 and 513 for estimating avg and std

[infer_isize] inferred external isize from 204555 pairs: 463.233 +/- 13.939

[infer_isize] skewness: 0.003; kurtosis: -0.149; ap_prior: 1.63e-05

[infer_isize] inferred maximum insert size: 560 (6.96 sigma)

[bwa_sai2sam_pe_core] time elapses: 25.84 sec

[bwa_sai2sam_pe_core] changing coordinates of 6043 alignments.

[bwa_sai2sam_pe_core] align unmapped mate...

[bwa_paired_sw] 10762 out of 11345 Q17 singletons are mated.

[bwa_paired_sw] 887 out of 1497 Q17 discordant pairs are fixed.

[bwa_sai2sam_pe_core] time elapses: 4.37 sec

[bwa_sai2sam_pe_core] refine gapped alignments... 0.89 sec

[bwa_sai2sam_pe_core] print alignments... 2.10 sec

[bwa_sai2sam_pe_core] 88342528 sequences have been processed.

[bwa_sai2sam_pe_core] convert to sequence coordinate...

[infer_isize] (25, 50, 75) percentile: (453, 463, 473)

[infer_isize] low and high boundaries: 413 and 513 for estimating avg and std

[infer_isize] inferred external isize from 205160 pairs: 463.249 +/- 13.941

[infer_isize] skewness: -0.002; kurtosis: -0.137; ap_prior: 1.67e-05

[infer_isize] inferred maximum insert size: 560 (6.96 sigma)

[bwa_sai2sam_pe_core] time elapses: 22.40 sec

[bwa_sai2sam_pe_core] changing coordinates of 5792 alignments.

[bwa_sai2sam_pe_core] align unmapped mate...

[bwa_paired_sw] 10357 out of 10956 Q17 singletons are mated.

[bwa_paired_sw] 898 out of 1469 Q17 discordant pairs are fixed.

[bwa_sai2sam_pe_core] time elapses: 4.23 sec

[bwa_sai2sam_pe_core] refine gapped alignments... 0.91 sec

[bwa_sai2sam_pe_core] print alignments... 2.11 sec

[bwa_sai2sam_pe_core] 88604672 sequences have been processed.

[bwa_sai2sam_pe_core] convert to sequence coordinate...

[infer_isize] (25, 50, 75) percentile: (453, 463, 473)

[infer_isize] low and high boundaries: 413 and 513 for estimating avg and std

[infer_isize] inferred external isize from 205233 pairs: 463.250 +/- 13.942

[infer_isize] skewness: 0.003; kurtosis: -0.143; ap_prior: 1.60e-05

[infer_isize] inferred maximum insert size: 560 (6.96 sigma)

[bwa_sai2sam_pe_core] time elapses: 28.05 sec

[bwa_sai2sam_pe_core] changing coordinates of 6130 alignments.

[bwa_sai2sam_pe_core] align unmapped mate...

[bwa_paired_sw] 10138 out of 10702 Q17 singletons are mated.

[bwa_paired_sw] 814 out of 1399 Q17 discordant pairs are fixed.

[bwa_sai2sam_pe_core] time elapses: 4.13 sec

[bwa_sai2sam_pe_core] refine gapped alignments... 0.90 sec

[bwa_sai2sam_pe_core] print alignments... 2.10 sec

[bwa_sai2sam_pe_core] 88866816 sequences have been processed.

[bwa_sai2sam_pe_core] convert to sequence coordinate...

[infer_isize] (25, 50, 75) percentile: (453, 463, 473)

[infer_isize] low and high boundaries: 413 and 513 for estimating avg and std

[infer_isize] inferred external isize from 204844 pairs: 463.238 +/- 13.940

[infer_isize] skewness: 0.010; kurtosis: -0.141; ap_prior: 1.69e-05

[infer_isize] inferred maximum insert size: 560 (6.96 sigma)

[bwa_sai2sam_pe_core] time elapses: 22.54 sec

[bwa_sai2sam_pe_core] changing coordinates of 6054 alignments.

[bwa_sai2sam_pe_core] align unmapped mate...

[bwa_paired_sw] 10380 out of 10968 Q17 singletons are mated.

[bwa_paired_sw] 908 out of 1528 Q17 discordant pairs are fixed.

[bwa_sai2sam_pe_core] time elapses: 4.23 sec

[bwa_sai2sam_pe_core] refine gapped alignments... 0.88 sec

[bwa_sai2sam_pe_core] print alignments... 2.13 sec

[bwa_sai2sam_pe_core] 89128960 sequences have been processed.

[bwa_sai2sam_pe_core] convert to sequence coordinate...

[infer_isize] (25, 50, 75) percentile: (453, 463, 473)

[infer_isize] low and high boundaries: 413 and 513 for estimating avg and std

[infer_isize] inferred external isize from 205351 pairs: 463.263 +/- 13.959

[infer_isize] skewness: -0.006; kurtosis: -0.113; ap_prior: 1.87e-05

[infer_isize] inferred maximum insert size: 560 (6.96 sigma)

[bwa_sai2sam_pe_core] time elapses: 20.45 sec

[bwa_sai2sam_pe_core] changing coordinates of 5949 alignments.

[bwa_sai2sam_pe_core] align unmapped mate...

[bwa_paired_sw] 10195 out of 10718 Q17 singletons are mated.

[bwa_paired_sw] 897 out of 1514 Q17 discordant pairs are fixed.

[bwa_sai2sam_pe_core] time elapses: 4.18 sec

[bwa_sai2sam_pe_core] refine gapped alignments... 0.87 sec

[bwa_sai2sam_pe_core] print alignments... 2.09 sec

[bwa_sai2sam_pe_core] 89391104 sequences have been processed.

[bwa_sai2sam_pe_core] convert to sequence coordinate...

[infer_isize] (25, 50, 75) percentile: (453, 463, 473)

[infer_isize] low and high boundaries: 413 and 513 for estimating avg and std

[infer_isize] inferred external isize from 205302 pairs: 463.250 +/- 13.935

[infer_isize] skewness: -0.006; kurtosis: -0.113; ap_prior: 1.85e-05

[infer_isize] inferred maximum insert size: 560 (6.96 sigma)

[bwa_sai2sam_pe_core] time elapses: 20.70 sec

[bwa_sai2sam_pe_core] changing coordinates of 6167 alignments.

[bwa_sai2sam_pe_core] align unmapped mate...

[bwa_paired_sw] 10193 out of 10778 Q17 singletons are mated.

[bwa_paired_sw] 899 out of 1515 Q17 discordant pairs are fixed.

[bwa_sai2sam_pe_core] time elapses: 4.19 sec

[bwa_sai2sam_pe_core] refine gapped alignments... 0.91 sec

[bwa_sai2sam_pe_core] print alignments... 2.08 sec

[bwa_sai2sam_pe_core] 89653248 sequences have been processed.

[bwa_sai2sam_pe_core] convert to sequence coordinate...

[infer_isize] (25, 50, 75) percentile: (453, 463, 473)

[infer_isize] low and high boundaries: 413 and 513 for estimating avg and std

[infer_isize] inferred external isize from 204836 pairs: 463.311 +/- 13.966

[infer_isize] skewness: 0.001; kurtosis: -0.141; ap_prior: 1.78e-05

[infer_isize] inferred maximum insert size: 561 (6.96 sigma)

[bwa_sai2sam_pe_core] time elapses: 20.32 sec

[bwa_sai2sam_pe_core] changing coordinates of 5909 alignments.

[bwa_sai2sam_pe_core] align unmapped mate...

[bwa_paired_sw] 10493 out of 11005 Q17 singletons are mated.

[bwa_paired_sw] 929 out of 1508 Q17 discordant pairs are fixed.

[bwa_sai2sam_pe_core] time elapses: 4.27 sec

[bwa_sai2sam_pe_core] refine gapped alignments... 0.89 sec

[bwa_sai2sam_pe_core] print alignments... 2.06 sec

[bwa_sai2sam_pe_core] 89915392 sequences have been processed.

[bwa_sai2sam_pe_core] convert to sequence coordinate...

[infer_isize] (25, 50, 75) percentile: (453, 463, 473)

[infer_isize] low and high boundaries: 413 and 513 for estimating avg and std

[infer_isize] inferred external isize from 205097 pairs: 463.278 +/- 13.897

[infer_isize] skewness: 0.010; kurtosis: -0.127; ap_prior: 1.64e-05

[infer_isize] inferred maximum insert size: 560 (6.96 sigma)

[bwa_sai2sam_pe_core] time elapses: 20.49 sec

[bwa_sai2sam_pe_core] changing coordinates of 6116 alignments.

[bwa_sai2sam_pe_core] align unmapped mate...

[bwa_paired_sw] 10061 out of 10627 Q17 singletons are mated.

[bwa_paired_sw] 894 out of 1511 Q17 discordant pairs are fixed.

[bwa_sai2sam_pe_core] time elapses: 4.15 sec

[bwa_sai2sam_pe_core] refine gapped alignments... 0.91 sec

[bwa_sai2sam_pe_core] print alignments... 2.05 sec

[bwa_sai2sam_pe_core] 90177536 sequences have been processed.

[bwa_sai2sam_pe_core] convert to sequence coordinate...

[infer_isize] (25, 50, 75) percentile: (453, 463, 473)

[infer_isize] low and high boundaries: 413 and 513 for estimating avg and std

[infer_isize] inferred external isize from 205406 pairs: 463.214 +/- 13.919

[infer_isize] skewness: -0.002; kurtosis: -0.131; ap_prior: 1.71e-05

[infer_isize] inferred maximum insert size: 560 (6.96 sigma)

[bwa_sai2sam_pe_core] time elapses: 23.42 sec

[bwa_sai2sam_pe_core] changing coordinates of 6021 alignments.

[bwa_sai2sam_pe_core] align unmapped mate...

[bwa_paired_sw] 10117 out of 10666 Q17 singletons are mated.

[bwa_paired_sw] 909 out of 1487 Q17 discordant pairs are fixed.

[bwa_sai2sam_pe_core] time elapses: 4.23 sec

[bwa_sai2sam_pe_core] refine gapped alignments... 0.92 sec

[bwa_sai2sam_pe_core] print alignments... 2.13 sec

[bwa_sai2sam_pe_core] 90439680 sequences have been processed.

[bwa_sai2sam_pe_core] convert to sequence coordinate...

[infer_isize] (25, 50, 75) percentile: (453, 463, 473)

[infer_isize] low and high boundaries: 413 and 513 for estimating avg and std

[infer_isize] inferred external isize from 205366 pairs: 463.278 +/- 13.933

[infer_isize] skewness: 0.008; kurtosis: -0.147; ap_prior: 1.72e-05

[infer_isize] inferred maximum insert size: 560 (6.96 sigma)

[bwa_sai2sam_pe_core] time elapses: 22.38 sec

[bwa_sai2sam_pe_core] changing coordinates of 6026 alignments.

[bwa_sai2sam_pe_core] align unmapped mate...

[bwa_paired_sw] 10036 out of 10602 Q17 singletons are mated.

[bwa_paired_sw] 867 out of 1496 Q17 discordant pairs are fixed.

[bwa_sai2sam_pe_core] time elapses: 4.13 sec

[bwa_sai2sam_pe_core] refine gapped alignments... 0.92 sec

[bwa_sai2sam_pe_core] print alignments... 2.10 sec

[bwa_sai2sam_pe_core] 90701824 sequences have been processed.

[bwa_sai2sam_pe_core] convert to sequence coordinate...

[infer_isize] (25, 50, 75) percentile: (453, 463, 473)

[infer_isize] low and high boundaries: 413 and 513 for estimating avg and std

[infer_isize] inferred external isize from 205023 pairs: 463.263 +/- 13.926

[infer_isize] skewness: 0.001; kurtosis: -0.137; ap_prior: 1.66e-05

[infer_isize] inferred maximum insert size: 560 (6.96 sigma)

[bwa_sai2sam_pe_core] time elapses: 22.73 sec

[bwa_sai2sam_pe_core] changing coordinates of 6019 alignments.

[bwa_sai2sam_pe_core] align unmapped mate...

[bwa_paired_sw] 10128 out of 10690 Q17 singletons are mated.

[bwa_paired_sw] 859 out of 1467 Q17 discordant pairs are fixed.

[bwa_sai2sam_pe_core] time elapses: 4.14 sec

[bwa_sai2sam_pe_core] refine gapped alignments... 0.90 sec

[bwa_sai2sam_pe_core] print alignments... 2.10 sec

[bwa_sai2sam_pe_core] 90963968 sequences have been processed.

[bwa_sai2sam_pe_core] convert to sequence coordinate...

[infer_isize] (25, 50, 75) percentile: (453, 463, 473)

[infer_isize] low and high boundaries: 413 and 513 for estimating avg and std

[infer_isize] inferred external isize from 205428 pairs: 463.279 +/- 13.920

[infer_isize] skewness: 0.004; kurtosis: -0.125; ap_prior: 1.81e-05

[infer_isize] inferred maximum insert size: 560 (6.96 sigma)

[bwa_sai2sam_pe_core] time elapses: 22.44 sec

[bwa_sai2sam_pe_core] changing coordinates of 5891 alignments.

[bwa_sai2sam_pe_core] align unmapped mate...

[bwa_paired_sw] 10032 out of 10553 Q17 singletons are mated.

[bwa_paired_sw] 940 out of 1561 Q17 discordant pairs are fixed.

[bwa_sai2sam_pe_core] time elapses: 4.16 sec

[bwa_sai2sam_pe_core] refine gapped alignments... 0.90 sec

[bwa_sai2sam_pe_core] print alignments... 2.10 sec

[bwa_sai2sam_pe_core] 91226112 sequences have been processed.

[bwa_sai2sam_pe_core] convert to sequence coordinate...

[infer_isize] (25, 50, 75) percentile: (453, 463, 473)

[infer_isize] low and high boundaries: 413 and 513 for estimating avg and std

[infer_isize] inferred external isize from 205044 pairs: 463.258 +/- 13.961

[infer_isize] skewness: 0.001; kurtosis: -0.138; ap_prior: 1.75e-05

[infer_isize] inferred maximum insert size: 560 (6.96 sigma)

[bwa_sai2sam_pe_core] time elapses: 22.54 sec

[bwa_sai2sam_pe_core] changing coordinates of 6036 alignments.

[bwa_sai2sam_pe_core] align unmapped mate...

[bwa_paired_sw] 10081 out of 10656 Q17 singletons are mated.

[bwa_paired_sw] 858 out of 1447 Q17 discordant pairs are fixed.

[bwa_sai2sam_pe_core] time elapses: 4.12 sec

[bwa_sai2sam_pe_core] refine gapped alignments... 0.88 sec

[bwa_sai2sam_pe_core] print alignments... 2.11 sec

[bwa_sai2sam_pe_core] 91488256 sequences have been processed.

[bwa_sai2sam_pe_core] convert to sequence coordinate...

[infer_isize] (25, 50, 75) percentile: (453, 463, 473)

[infer_isize] low and high boundaries: 413 and 513 for estimating avg and std

[infer_isize] inferred external isize from 205115 pairs: 463.223 +/- 13.955

[infer_isize] skewness: 0.001; kurtosis: -0.112; ap_prior: 1.81e-05

[infer_isize] inferred maximum insert size: 560 (6.96 sigma)

[bwa_sai2sam_pe_core] time elapses: 24.89 sec

[bwa_sai2sam_pe_core] changing coordinates of 6188 alignments.

[bwa_sai2sam_pe_core] align unmapped mate...

[bwa_paired_sw] 10186 out of 10768 Q17 singletons are mated.

[bwa_paired_sw] 893 out of 1549 Q17 discordant pairs are fixed.

[bwa_sai2sam_pe_core] time elapses: 4.21 sec

[bwa_sai2sam_pe_core] refine gapped alignments... 0.90 sec

[bwa_sai2sam_pe_core] print alignments... 2.09 sec

[bwa_sai2sam_pe_core] 91750400 sequences have been processed.

[bwa_sai2sam_pe_core] convert to sequence coordinate...

[infer_isize] (25, 50, 75) percentile: (453, 463, 473)

[infer_isize] low and high boundaries: 413 and 513 for estimating avg and std

[infer_isize] inferred external isize from 205029 pairs: 463.269 +/- 13.933

[infer_isize] skewness: 0.000; kurtosis: -0.151; ap_prior: 1.74e-05

[infer_isize] inferred maximum insert size: 560 (6.96 sigma)

[bwa_sai2sam_pe_core] time elapses: 25.51 sec

[bwa_sai2sam_pe_core] changing coordinates of 5816 alignments.

[bwa_sai2sam_pe_core] align unmapped mate...

[bwa_paired_sw] 10302 out of 10855 Q17 singletons are mated.

[bwa_paired_sw] 886 out of 1534 Q17 discordant pairs are fixed.

[bwa_sai2sam_pe_core] time elapses: 4.21 sec

[bwa_sai2sam_pe_core] refine gapped alignments... 0.90 sec

[bwa_sai2sam_pe_core] print alignments... 2.08 sec

[bwa_sai2sam_pe_core] 92012544 sequences have been processed.

[bwa_sai2sam_pe_core] convert to sequence coordinate...

[infer_isize] (25, 50, 75) percentile: (453, 463, 473)

[infer_isize] low and high boundaries: 413 and 513 for estimating avg and std

[infer_isize] inferred external isize from 204806 pairs: 463.289 +/- 13.947

[infer_isize] skewness: 0.002; kurtosis: -0.139; ap_prior: 1.88e-05

[infer_isize] inferred maximum insert size: 560 (6.96 sigma)

[bwa_sai2sam_pe_core] time elapses: 28.10 sec

[bwa_sai2sam_pe_core] changing coordinates of 6068 alignments.

[bwa_sai2sam_pe_core] align unmapped mate...

[bwa_paired_sw] 9935 out of 10517 Q17 singletons are mated.

[bwa_paired_sw] 935 out of 1577 Q17 discordant pairs are fixed.

[bwa_sai2sam_pe_core] time elapses: 4.16 sec

[bwa_sai2sam_pe_core] refine gapped alignments... 0.88 sec

[bwa_sai2sam_pe_core] print alignments... 2.09 sec

[bwa_sai2sam_pe_core] 92274688 sequences have been processed.

[bwa_sai2sam_pe_core] convert to sequence coordinate...

[infer_isize] (25, 50, 75) percentile: (453, 463, 473)

[infer_isize] low and high boundaries: 413 and 513 for estimating avg and std

[infer_isize] inferred external isize from 205282 pairs: 463.213 +/- 13.996

[infer_isize] skewness: -0.005; kurtosis: -0.136; ap_prior: 1.68e-05

[infer_isize] inferred maximum insert size: 561 (6.96 sigma)

[bwa_sai2sam_pe_core] time elapses: 21.60 sec

[bwa_sai2sam_pe_core] changing coordinates of 5987 alignments.

[bwa_sai2sam_pe_core] align unmapped mate...

[bwa_paired_sw] 10038 out of 10570 Q17 singletons are mated.

[bwa_paired_sw] 818 out of 1422 Q17 discordant pairs are fixed.

[bwa_sai2sam_pe_core] time elapses: 4.08 sec

[bwa_sai2sam_pe_core] refine gapped alignments... 0.90 sec

[bwa_sai2sam_pe_core] print alignments... 2.11 sec

[bwa_sai2sam_pe_core] 92536832 sequences have been processed.

[bwa_sai2sam_pe_core] convert to sequence coordinate...

[infer_isize] (25, 50, 75) percentile: (453, 463, 473)

[infer_isize] low and high boundaries: 413 and 513 for estimating avg and std

[infer_isize] inferred external isize from 205036 pairs: 463.227 +/- 13.936

[infer_isize] skewness: -0.002; kurtosis: -0.137; ap_prior: 1.69e-05

[infer_isize] inferred maximum insert size: 560 (6.96 sigma)

[bwa_sai2sam_pe_core] time elapses: 21.82 sec

[bwa_sai2sam_pe_core] changing coordinates of 5918 alignments.

[bwa_sai2sam_pe_core] align unmapped mate...

[bwa_paired_sw] 10273 out of 10818 Q17 singletons are mated.

[bwa_paired_sw] 874 out of 1471 Q17 discordant pairs are fixed.

[bwa_sai2sam_pe_core] time elapses: 4.19 sec

[bwa_sai2sam_pe_core] refine gapped alignments... 0.88 sec

[bwa_sai2sam_pe_core] print alignments... 2.07 sec

[bwa_sai2sam_pe_core] 92798976 sequences have been processed.

[bwa_sai2sam_pe_core] convert to sequence coordinate...

[infer_isize] (25, 50, 75) percentile: (453, 463, 473)

[infer_isize] low and high boundaries: 413 and 513 for estimating avg and std

[infer_isize] inferred external isize from 204792 pairs: 463.256 +/- 13.944

[infer_isize] skewness: 0.003; kurtosis: -0.150; ap_prior: 1.71e-05

[infer_isize] inferred maximum insert size: 560 (6.96 sigma)

[bwa_sai2sam_pe_core] time elapses: 21.73 sec

[bwa_sai2sam_pe_core] changing coordinates of 6038 alignments.

[bwa_sai2sam_pe_core] align unmapped mate...

[bwa_paired_sw] 10435 out of 10961 Q17 singletons are mated.

[bwa_paired_sw] 898 out of 1475 Q17 discordant pairs are fixed.

[bwa_sai2sam_pe_core] time elapses: 4.23 sec

[bwa_sai2sam_pe_core] refine gapped alignments... 0.89 sec

[bwa_sai2sam_pe_core] print alignments... 2.06 sec

[bwa_sai2sam_pe_core] 93061120 sequences have been processed.

[bwa_sai2sam_pe_core] convert to sequence coordinate...

[infer_isize] (25, 50, 75) percentile: (453, 463, 473)

[infer_isize] low and high boundaries: 413 and 513 for estimating avg and std

[infer_isize] inferred external isize from 205431 pairs: 463.208 +/- 13.904

[infer_isize] skewness: -0.001; kurtosis: -0.132; ap_prior: 1.86e-05

[infer_isize] inferred maximum insert size: 560 (6.96 sigma)

[bwa_sai2sam_pe_core] time elapses: 21.83 sec

[bwa_sai2sam_pe_core] changing coordinates of 5914 alignments.

[bwa_sai2sam_pe_core] align unmapped mate...

[bwa_paired_sw] 9994 out of 10563 Q17 singletons are mated.

[bwa_paired_sw] 879 out of 1511 Q17 discordant pairs are fixed.

[bwa_sai2sam_pe_core] time elapses: 4.14 sec

[bwa_sai2sam_pe_core] refine gapped alignments... 0.91 sec

[bwa_sai2sam_pe_core] print alignments... 2.09 sec

[bwa_sai2sam_pe_core] 93323264 sequences have been processed.

[bwa_sai2sam_pe_core] convert to sequence coordinate...

[infer_isize] (25, 50, 75) percentile: (453, 463, 473)

[infer_isize] low and high boundaries: 413 and 513 for estimating avg and std

[infer_isize] inferred external isize from 205537 pairs: 463.250 +/- 13.906

[infer_isize] skewness: -0.007; kurtosis: -0.136; ap_prior: 1.59e-05

[infer_isize] inferred maximum insert size: 560 (6.96 sigma)

[bwa_sai2sam_pe_core] time elapses: 45.90 sec

[bwa_sai2sam_pe_core] changing coordinates of 5899 alignments.

[bwa_sai2sam_pe_core] align unmapped mate...

[bwa_paired_sw] 10076 out of 10621 Q17 singletons are mated.

[bwa_paired_sw] 816 out of 1421 Q17 discordant pairs are fixed.

[bwa_sai2sam_pe_core] time elapses: 4.25 sec

[bwa_sai2sam_pe_core] refine gapped alignments... 1.01 sec

[bwa_sai2sam_pe_core] print alignments... 2.19 sec

[bwa_sai2sam_pe_core] 93585408 sequences have been processed.

[bwa_sai2sam_pe_core] convert to sequence coordinate...

[infer_isize] (25, 50, 75) percentile: (453, 463, 473)

[infer_isize] low and high boundaries: 413 and 513 for estimating avg and std

[infer_isize] inferred external isize from 204926 pairs: 463.230 +/- 13.925

[infer_isize] skewness: 0.009; kurtosis: -0.131; ap_prior: 1.73e-05

[infer_isize] inferred maximum insert size: 560 (6.96 sigma)

[bwa_sai2sam_pe_core] time elapses: 33.91 sec

[bwa_sai2sam_pe_core] changing coordinates of 5964 alignments.

[bwa_sai2sam_pe_core] align unmapped mate...

[bwa_paired_sw] 10278 out of 10815 Q17 singletons are mated.

[bwa_paired_sw] 900 out of 1528 Q17 discordant pairs are fixed.

[bwa_sai2sam_pe_core] time elapses: 4.29 sec

[bwa_sai2sam_pe_core] refine gapped alignments... 1.01 sec

[bwa_sai2sam_pe_core] print alignments... 2.24 sec

[bwa_sai2sam_pe_core] 93847552 sequences have been processed.

[bwa_sai2sam_pe_core] convert to sequence coordinate...

[infer_isize] (25, 50, 75) percentile: (453, 463, 473)

[infer_isize] low and high boundaries: 413 and 513 for estimating avg and std

[infer_isize] inferred external isize from 205271 pairs: 463.268 +/- 13.921

[infer_isize] skewness: 0.001; kurtosis: -0.136; ap_prior: 1.66e-05

[infer_isize] inferred maximum insert size: 560 (6.96 sigma)

[bwa_sai2sam_pe_core] time elapses: 32.39 sec

[bwa_sai2sam_pe_core] changing coordinates of 5989 alignments.

[bwa_sai2sam_pe_core] align unmapped mate...

[bwa_paired_sw] 10109 out of 10634 Q17 singletons are mated.

[bwa_paired_sw] 878 out of 1506 Q17 discordant pairs are fixed.

[bwa_sai2sam_pe_core] time elapses: 4.15 sec

[bwa_sai2sam_pe_core] refine gapped alignments... 0.90 sec

[bwa_sai2sam_pe_core] print alignments... 2.09 sec

[bwa_sai2sam_pe_core] 94109696 sequences have been processed.

[bwa_sai2sam_pe_core] convert to sequence coordinate...

[infer_isize] (25, 50, 75) percentile: (453, 463, 473)

[infer_isize] low and high boundaries: 413 and 513 for estimating avg and std

[infer_isize] inferred external isize from 205506 pairs: 463.272 +/- 13.946

[infer_isize] skewness: 0.006; kurtosis: -0.121; ap_prior: 1.69e-05

[infer_isize] inferred maximum insert size: 560 (6.96 sigma)

[bwa_sai2sam_pe_core] time elapses: 29.21 sec

[bwa_sai2sam_pe_core] changing coordinates of 5922 alignments.

[bwa_sai2sam_pe_core] align unmapped mate...

[bwa_paired_sw] 10036 out of 10553 Q17 singletons are mated.

[bwa_paired_sw] 910 out of 1526 Q17 discordant pairs are fixed.

[bwa_sai2sam_pe_core] time elapses: 4.15 sec

[bwa_sai2sam_pe_core] refine gapped alignments... 0.90 sec

[bwa_sai2sam_pe_core] print alignments... 2.11 sec

[bwa_sai2sam_pe_core] 94371840 sequences have been processed.

[bwa_sai2sam_pe_core] convert to sequence coordinate...

[infer_isize] (25, 50, 75) percentile: (453, 463, 473)

[infer_isize] low and high boundaries: 413 and 513 for estimating avg and std

[infer_isize] inferred external isize from 205308 pairs: 463.178 +/- 13.873

[infer_isize] skewness: 0.006; kurtosis: -0.135; ap_prior: 1.81e-05

[infer_isize] inferred maximum insert size: 560 (6.96 sigma)

[bwa_sai2sam_pe_core] time elapses: 21.67 sec

[bwa_sai2sam_pe_core] changing coordinates of 5968 alignments.

[bwa_sai2sam_pe_core] align unmapped mate...

[bwa_paired_sw] 9924 out of 10452 Q17 singletons are mated.

[bwa_paired_sw] 914 out of 1527 Q17 discordant pairs are fixed.

[bwa_sai2sam_pe_core] time elapses: 4.11 sec

[bwa_sai2sam_pe_core] refine gapped alignments... 0.88 sec

[bwa_sai2sam_pe_core] print alignments... 2.11 sec

[bwa_sai2sam_pe_core] 94633984 sequences have been processed.

[bwa_sai2sam_pe_core] convert to sequence coordinate...

[infer_isize] (25, 50, 75) percentile: (453, 463, 473)

[infer_isize] low and high boundaries: 413 and 513 for estimating avg and std

[infer_isize] inferred external isize from 205354 pairs: 463.271 +/- 13.917

[infer_isize] skewness: 0.004; kurtosis: -0.129; ap_prior: 1.67e-05

[infer_isize] inferred maximum insert size: 560 (6.96 sigma)

[bwa_sai2sam_pe_core] time elapses: 21.86 sec

[bwa_sai2sam_pe_core] changing coordinates of 5904 alignments.

[bwa_sai2sam_pe_core] align unmapped mate...

[bwa_paired_sw] 10163 out of 10698 Q17 singletons are mated.

[bwa_paired_sw] 889 out of 1539 Q17 discordant pairs are fixed.

[bwa_sai2sam_pe_core] time elapses: 4.18 sec

[bwa_sai2sam_pe_core] refine gapped alignments... 0.89 sec

[bwa_sai2sam_pe_core] print alignments... 2.11 sec

[bwa_sai2sam_pe_core] 94896128 sequences have been processed.

[bwa_sai2sam_pe_core] convert to sequence coordinate...

[infer_isize] (25, 50, 75) percentile: (453, 463, 473)

[infer_isize] low and high boundaries: 413 and 513 for estimating avg and std

[infer_isize] inferred external isize from 205866 pairs: 463.290 +/- 13.965

[infer_isize] skewness: 0.006; kurtosis: -0.146; ap_prior: 1.97e-05

[infer_isize] inferred maximum insert size: 560 (6.96 sigma)

[bwa_sai2sam_pe_core] time elapses: 22.06 sec

[bwa_sai2sam_pe_core] changing coordinates of 6072 alignments.

[bwa_sai2sam_pe_core] align unmapped mate...

[bwa_paired_sw] 9822 out of 10345 Q17 singletons are mated.

[bwa_paired_sw] 891 out of 1464 Q17 discordant pairs are fixed.

[bwa_sai2sam_pe_core] time elapses: 4.05 sec

[bwa_sai2sam_pe_core] refine gapped alignments... 0.90 sec

[bwa_sai2sam_pe_core] print alignments... 2.11 sec

[bwa_sai2sam_pe_core] 95158272 sequences have been processed.

[bwa_sai2sam_pe_core] convert to sequence coordinate...

[infer_isize] (25, 50, 75) percentile: (453, 463, 473)

[infer_isize] low and high boundaries: 413 and 513 for estimating avg and std

[infer_isize] inferred external isize from 205554 pairs: 463.257 +/- 13.939

[infer_isize] skewness: 0.006; kurtosis: -0.146; ap_prior: 1.86e-05

[infer_isize] inferred maximum insert size: 560 (6.96 sigma)

[bwa_sai2sam_pe_core] time elapses: 21.65 sec

[bwa_sai2sam_pe_core] changing coordinates of 5980 alignments.

[bwa_sai2sam_pe_core] align unmapped mate...

[bwa_paired_sw] 9932 out of 10459 Q17 singletons are mated.

[bwa_paired_sw] 822 out of 1436 Q17 discordant pairs are fixed.

[bwa_sai2sam_pe_core] time elapses: 4.07 sec

[bwa_sai2sam_pe_core] refine gapped alignments... 0.91 sec

[bwa_sai2sam_pe_core] print alignments... 2.09 sec

[bwa_sai2sam_pe_core] 95420416 sequences have been processed.

[bwa_sai2sam_pe_core] convert to sequence coordinate...

[infer_isize] (25, 50, 75) percentile: (453, 463, 473)

[infer_isize] low and high boundaries: 413 and 513 for estimating avg and std

[infer_isize] inferred external isize from 205793 pairs: 463.254 +/- 13.926

[infer_isize] skewness: 0.002; kurtosis: -0.141; ap_prior: 1.75e-05

[infer_isize] inferred maximum insert size: 560 (6.96 sigma)

[bwa_sai2sam_pe_core] time elapses: 30.20 sec

[bwa_sai2sam_pe_core] changing coordinates of 5893 alignments.

[bwa_sai2sam_pe_core] align unmapped mate...

[bwa_paired_sw] 9844 out of 10335 Q17 singletons are mated.

[bwa_paired_sw] 880 out of 1476 Q17 discordant pairs are fixed.

[bwa_sai2sam_pe_core] time elapses: 4.07 sec

[bwa_sai2sam_pe_core] refine gapped alignments... 0.91 sec

[bwa_sai2sam_pe_core] print alignments... 2.07 sec

[bwa_sai2sam_pe_core] 95682560 sequences have been processed.

[bwa_sai2sam_pe_core] convert to sequence coordinate...

[infer_isize] (25, 50, 75) percentile: (453, 463, 473)

[infer_isize] low and high boundaries: 413 and 513 for estimating avg and std

[infer_isize] inferred external isize from 205384 pairs: 463.224 +/- 13.922

[infer_isize] skewness: -0.003; kurtosis: -0.129; ap_prior: 1.75e-05

[infer_isize] inferred maximum insert size: 560 (6.96 sigma)

[bwa_sai2sam_pe_core] time elapses: 23.30 sec

[bwa_sai2sam_pe_core] changing coordinates of 6020 alignments.

[bwa_sai2sam_pe_core] align unmapped mate...

[bwa_paired_sw] 10028 out of 10547 Q17 singletons are mated.

[bwa_paired_sw] 872 out of 1503 Q17 discordant pairs are fixed.

[bwa_sai2sam_pe_core] time elapses: 4.14 sec

[bwa_sai2sam_pe_core] refine gapped alignments... 0.91 sec

[bwa_sai2sam_pe_core] print alignments... 2.08 sec

[bwa_sai2sam_pe_core] 95944704 sequences have been processed.

[bwa_sai2sam_pe_core] convert to sequence coordinate...

[infer_isize] (25, 50, 75) percentile: (453, 463, 473)

[infer_isize] low and high boundaries: 413 and 513 for estimating avg and std

[infer_isize] inferred external isize from 205376 pairs: 463.233 +/- 13.954

[infer_isize] skewness: 0.002; kurtosis: -0.143; ap_prior: 1.68e-05

[infer_isize] inferred maximum insert size: 560 (6.96 sigma)

[bwa_sai2sam_pe_core] time elapses: 23.50 sec

[bwa_sai2sam_pe_core] changing coordinates of 6032 alignments.

[bwa_sai2sam_pe_core] align unmapped mate...

[bwa_paired_sw] 9853 out of 10387 Q17 singletons are mated.

[bwa_paired_sw] 888 out of 1455 Q17 discordant pairs are fixed.

[bwa_sai2sam_pe_core] time elapses: 4.07 sec

[bwa_sai2sam_pe_core] refine gapped alignments... 0.90 sec

[bwa_sai2sam_pe_core] print alignments... 2.09 sec

[bwa_sai2sam_pe_core] 96206848 sequences have been processed.

[bwa_sai2sam_pe_core] convert to sequence coordinate...

[infer_isize] (25, 50, 75) percentile: (453, 463, 473)

[infer_isize] low and high boundaries: 413 and 513 for estimating avg and std

[infer_isize] inferred external isize from 205064 pairs: 463.206 +/- 13.938

[infer_isize] skewness: -0.002; kurtosis: -0.117; ap_prior: 1.69e-05

[infer_isize] inferred maximum insert size: 560 (6.96 sigma)

[bwa_sai2sam_pe_core] time elapses: 27.50 sec

[bwa_sai2sam_pe_core] changing coordinates of 6053 alignments.

[bwa_sai2sam_pe_core] align unmapped mate...

[bwa_paired_sw] 10134 out of 10640 Q17 singletons are mated.

[bwa_paired_sw] 897 out of 1484 Q17 discordant pairs are fixed.

[bwa_sai2sam_pe_core] time elapses: 4.20 sec

[bwa_sai2sam_pe_core] refine gapped alignments... 0.93 sec

[bwa_sai2sam_pe_core] print alignments... 2.15 sec

[bwa_sai2sam_pe_core] 96468992 sequences have been processed.

[bwa_sai2sam_pe_core] convert to sequence coordinate...

[infer_isize] (25, 50, 75) percentile: (453, 463, 473)

[infer_isize] low and high boundaries: 413 and 513 for estimating avg and std

[infer_isize] inferred external isize from 204398 pairs: 463.155 +/- 13.919

[infer_isize] skewness: 0.005; kurtosis: -0.145; ap_prior: 1.65e-05

[infer_isize] inferred maximum insert size: 560 (6.96 sigma)

[bwa_sai2sam_pe_core] time elapses: 27.46 sec

[bwa_sai2sam_pe_core] changing coordinates of 6164 alignments.

[bwa_sai2sam_pe_core] align unmapped mate...

[bwa_paired_sw] 10379 out of 10913 Q17 singletons are mated.

[bwa_paired_sw] 866 out of 1478 Q17 discordant pairs are fixed.

[bwa_sai2sam_pe_core] time elapses: 4.53 sec

[bwa_sai2sam_pe_core] refine gapped alignments... 0.98 sec

[bwa_sai2sam_pe_core] print alignments... 2.11 sec

[bwa_sai2sam_pe_core] 96731136 sequences have been processed.

[bwa_sai2sam_pe_core] convert to sequence coordinate...

[infer_isize] (25, 50, 75) percentile: (453, 463, 473)

[infer_isize] low and high boundaries: 413 and 513 for estimating avg and std

[infer_isize] inferred external isize from 204990 pairs: 463.224 +/- 13.942

[infer_isize] skewness: 0.013; kurtosis: -0.138; ap_prior: 1.76e-05

[infer_isize] inferred maximum insert size: 560 (6.96 sigma)

[bwa_sai2sam_pe_core] time elapses: 26.67 sec

[bwa_sai2sam_pe_core] changing coordinates of 6100 alignments.

[bwa_sai2sam_pe_core] align unmapped mate...

[bwa_paired_sw] 10007 out of 10561 Q17 singletons are mated.

[bwa_paired_sw] 905 out of 1514 Q17 discordant pairs are fixed.

[bwa_sai2sam_pe_core] time elapses: 4.17 sec

[bwa_sai2sam_pe_core] refine gapped alignments... 0.92 sec

[bwa_sai2sam_pe_core] print alignments... 2.11 sec

[bwa_sai2sam_pe_core] 96993280 sequences have been processed.

[bwa_sai2sam_pe_core] convert to sequence coordinate...

[infer_isize] (25, 50, 75) percentile: (453, 463, 473)

[infer_isize] low and high boundaries: 413 and 513 for estimating avg and std

[infer_isize] inferred external isize from 205178 pairs: 463.237 +/- 13.935

[infer_isize] skewness: 0.009; kurtosis: -0.147; ap_prior: 1.84e-05

[infer_isize] inferred maximum insert size: 560 (6.96 sigma)

[bwa_sai2sam_pe_core] time elapses: 22.54 sec

[bwa_sai2sam_pe_core] changing coordinates of 5810 alignments.

[bwa_sai2sam_pe_core] align unmapped mate...

[bwa_paired_sw] 10321 out of 10875 Q17 singletons are mated.

[bwa_paired_sw] 885 out of 1479 Q17 discordant pairs are fixed.

[bwa_sai2sam_pe_core] time elapses: 4.19 sec

[bwa_sai2sam_pe_core] refine gapped alignments... 0.89 sec

[bwa_sai2sam_pe_core] print alignments... 2.10 sec

[bwa_sai2sam_pe_core] 97255424 sequences have been processed.

[bwa_sai2sam_pe_core] convert to sequence coordinate...

[infer_isize] (25, 50, 75) percentile: (453, 463, 473)

[infer_isize] low and high boundaries: 413 and 513 for estimating avg and std

[infer_isize] inferred external isize from 205476 pairs: 463.255 +/- 13.969

[infer_isize] skewness: -0.003; kurtosis: -0.125; ap_prior: 1.75e-05

[infer_isize] inferred maximum insert size: 560 (6.96 sigma)

[bwa_sai2sam_pe_core] time elapses: 21.94 sec

[bwa_sai2sam_pe_core] changing coordinates of 5980 alignments.

[bwa_sai2sam_pe_core] align unmapped mate...

[bwa_paired_sw] 9737 out of 10270 Q17 singletons are mated.

[bwa_paired_sw] 929 out of 1541 Q17 discordant pairs are fixed.

[bwa_sai2sam_pe_core] time elapses: 4.07 sec

[bwa_sai2sam_pe_core] refine gapped alignments... 0.89 sec

[bwa_sai2sam_pe_core] print alignments... 2.12 sec

[bwa_sai2sam_pe_core] 97517568 sequences have been processed.

[bwa_sai2sam_pe_core] convert to sequence coordinate...

[infer_isize] (25, 50, 75) percentile: (453, 463, 473)

[infer_isize] low and high boundaries: 413 and 513 for estimating avg and std

[infer_isize] inferred external isize from 205375 pairs: 463.169 +/- 13.938

[infer_isize] skewness: -0.003; kurtosis: -0.135; ap_prior: 1.93e-05

[infer_isize] inferred maximum insert size: 560 (6.96 sigma)

[bwa_sai2sam_pe_core] time elapses: 22.08 sec

[bwa_sai2sam_pe_core] changing coordinates of 5978 alignments.

[bwa_sai2sam_pe_core] align unmapped mate...

[bwa_paired_sw] 9781 out of 10323 Q17 singletons are mated.

[bwa_paired_sw] 878 out of 1522 Q17 discordant pairs are fixed.

[bwa_sai2sam_pe_core] time elapses: 4.07 sec

[bwa_sai2sam_pe_core] refine gapped alignments... 0.91 sec

[bwa_sai2sam_pe_core] print alignments... 2.10 sec

[bwa_sai2sam_pe_core] 97779712 sequences have been processed.

[bwa_sai2sam_pe_core] convert to sequence coordinate...

[infer_isize] (25, 50, 75) percentile: (453, 463, 473)

[infer_isize] low and high boundaries: 413 and 513 for estimating avg and std

[infer_isize] inferred external isize from 204571 pairs: 463.176 +/- 13.921

[infer_isize] skewness: 0.001; kurtosis: -0.124; ap_prior: 1.69e-05

[infer_isize] inferred maximum insert size: 560 (6.96 sigma)

[bwa_sai2sam_pe_core] time elapses: 24.79 sec

[bwa_sai2sam_pe_core] changing coordinates of 6048 alignments.

[bwa_sai2sam_pe_core] align unmapped mate...

[bwa_paired_sw] 9972 out of 10487 Q17 singletons are mated.

[bwa_paired_sw] 860 out of 1477 Q17 discordant pairs are fixed.

[bwa_sai2sam_pe_core] time elapses: 4.10 sec

[bwa_sai2sam_pe_core] refine gapped alignments... 0.89 sec

[bwa_sai2sam_pe_core] print alignments... 2.07 sec

[bwa_sai2sam_pe_core] 98041856 sequences have been processed.

[bwa_sai2sam_pe_core] convert to sequence coordinate...

[infer_isize] (25, 50, 75) percentile: (453, 463, 473)

[infer_isize] low and high boundaries: 413 and 513 for estimating avg and std

[infer_isize] inferred external isize from 204900 pairs: 463.233 +/- 13.928

[infer_isize] skewness: 0.003; kurtosis: -0.149; ap_prior: 1.63e-05

[infer_isize] inferred maximum insert size: 560 (6.96 sigma)

[bwa_sai2sam_pe_core] time elapses: 25.02 sec

[bwa_sai2sam_pe_core] changing coordinates of 5995 alignments.

[bwa_sai2sam_pe_core] align unmapped mate...

[bwa_paired_sw] 10050 out of 10613 Q17 singletons are mated.

[bwa_paired_sw] 858 out of 1455 Q17 discordant pairs are fixed.

[bwa_sai2sam_pe_core] time elapses: 4.11 sec

[bwa_sai2sam_pe_core] refine gapped alignments... 0.90 sec

[bwa_sai2sam_pe_core] print alignments... 2.10 sec

[bwa_sai2sam_pe_core] 98304000 sequences have been processed.

[bwa_sai2sam_pe_core] convert to sequence coordinate...

[infer_isize] (25, 50, 75) percentile: (453, 463, 473)

[infer_isize] low and high boundaries: 413 and 513 for estimating avg and std

[infer_isize] inferred external isize from 205385 pairs: 463.185 +/- 13.955

[infer_isize] skewness: -0.006; kurtosis: -0.136; ap_prior: 1.57e-05

[infer_isize] inferred maximum insert size: 560 (6.96 sigma)

[bwa_sai2sam_pe_core] time elapses: 21.86 sec

[bwa_sai2sam_pe_core] changing coordinates of 6128 alignments.

[bwa_sai2sam_pe_core] align unmapped mate...

[bwa_paired_sw] 9922 out of 10482 Q17 singletons are mated.

[bwa_paired_sw] 888 out of 1500 Q17 discordant pairs are fixed.

[bwa_sai2sam_pe_core] time elapses: 4.12 sec

[bwa_sai2sam_pe_core] refine gapped alignments... 0.91 sec

[bwa_sai2sam_pe_core] print alignments... 2.10 sec

[bwa_sai2sam_pe_core] 98566144 sequences have been processed.

[bwa_sai2sam_pe_core] convert to sequence coordinate...

[infer_isize] (25, 50, 75) percentile: (453, 463, 473)

[infer_isize] low and high boundaries: 413 and 513 for estimating avg and std

[infer_isize] inferred external isize from 205558 pairs: 463.219 +/- 13.954

[infer_isize] skewness: 0.007; kurtosis: -0.140; ap_prior: 1.88e-05

[infer_isize] inferred maximum insert size: 560 (6.96 sigma)

[bwa_sai2sam_pe_core] time elapses: 20.29 sec

[bwa_sai2sam_pe_core] changing coordinates of 6084 alignments.

[bwa_sai2sam_pe_core] align unmapped mate...

[bwa_paired_sw] 9933 out of 10462 Q17 singletons are mated.

[bwa_paired_sw] 954 out of 1569 Q17 discordant pairs are fixed.

[bwa_sai2sam_pe_core] time elapses: 4.14 sec

[bwa_sai2sam_pe_core] refine gapped alignments... 0.91 sec

[bwa_sai2sam_pe_core] print alignments... 2.08 sec

[bwa_sai2sam_pe_core] 98828288 sequences have been processed.

[bwa_sai2sam_pe_core] convert to sequence coordinate...

[infer_isize] (25, 50, 75) percentile: (453, 463, 473)

[infer_isize] low and high boundaries: 413 and 513 for estimating avg and std

[infer_isize] inferred external isize from 205053 pairs: 463.255 +/- 13.919

[infer_isize] skewness: 0.004; kurtosis: -0.130; ap_prior: 1.64e-05

[infer_isize] inferred maximum insert size: 560 (6.96 sigma)

[bwa_sai2sam_pe_core] time elapses: 20.56 sec

[bwa_sai2sam_pe_core] changing coordinates of 6006 alignments.

[bwa_sai2sam_pe_core] align unmapped mate...

[bwa_paired_sw] 10141 out of 10655 Q17 singletons are mated.

[bwa_paired_sw] 857 out of 1461 Q17 discordant pairs are fixed.

[bwa_sai2sam_pe_core] time elapses: 4.15 sec

[bwa_sai2sam_pe_core] refine gapped alignments... 0.91 sec

[bwa_sai2sam_pe_core] print alignments... 2.07 sec

[bwa_sai2sam_pe_core] 99090432 sequences have been processed.

[bwa_sai2sam_pe_core] convert to sequence coordinate...

[infer_isize] (25, 50, 75) percentile: (453, 463, 473)

[infer_isize] low and high boundaries: 413 and 513 for estimating avg and std

[infer_isize] inferred external isize from 204652 pairs: 463.242 +/- 13.944

[infer_isize] skewness: 0.010; kurtosis: -0.122; ap_prior: 1.72e-05

[infer_isize] inferred maximum insert size: 560 (6.96 sigma)

[bwa_sai2sam_pe_core] time elapses: 20.70 sec

[bwa_sai2sam_pe_core] changing coordinates of 6097 alignments.

[bwa_sai2sam_pe_core] align unmapped mate...

[bwa_paired_sw] 10071 out of 10630 Q17 singletons are mated.

[bwa_paired_sw] 819 out of 1428 Q17 discordant pairs are fixed.

[bwa_sai2sam_pe_core] time elapses: 4.14 sec

[bwa_sai2sam_pe_core] refine gapped alignments... 0.90 sec

[bwa_sai2sam_pe_core] print alignments... 2.05 sec

[bwa_sai2sam_pe_core] 99352576 sequences have been processed.

[bwa_sai2sam_pe_core] convert to sequence coordinate...

[infer_isize] (25, 50, 75) percentile: (453, 463, 473)

[infer_isize] low and high boundaries: 413 and 513 for estimating avg and std

[infer_isize] inferred external isize from 205155 pairs: 463.191 +/- 13.970

[infer_isize] skewness: -0.002; kurtosis: -0.137; ap_prior: 1.74e-05

[infer_isize] inferred maximum insert size: 560 (6.96 sigma)

[bwa_sai2sam_pe_core] time elapses: 20.71 sec

[bwa_sai2sam_pe_core] changing coordinates of 5971 alignments.

[bwa_sai2sam_pe_core] align unmapped mate...

[bwa_paired_sw] 9982 out of 10510 Q17 singletons are mated.

[bwa_paired_sw] 852 out of 1454 Q17 discordant pairs are fixed.

[bwa_sai2sam_pe_core] time elapses: 4.11 sec

[bwa_sai2sam_pe_core] refine gapped alignments... 0.90 sec

[bwa_sai2sam_pe_core] print alignments... 2.08 sec

[bwa_sai2sam_pe_core] 99614720 sequences have been processed.

[bwa_sai2sam_pe_core] convert to sequence coordinate...

[infer_isize] (25, 50, 75) percentile: (453, 463, 473)

[infer_isize] low and high boundaries: 413 and 513 for estimating avg and std

[infer_isize] inferred external isize from 205261 pairs: 463.228 +/- 13.935

[infer_isize] skewness: 0.006; kurtosis: -0.134; ap_prior: 1.74e-05

[infer_isize] inferred maximum insert size: 560 (6.96 sigma)

[bwa_sai2sam_pe_core] time elapses: 23.21 sec

[bwa_sai2sam_pe_core] changing coordinates of 6043 alignments.

[bwa_sai2sam_pe_core] align unmapped mate...

[bwa_paired_sw] 9879 out of 10404 Q17 singletons are mated.

[bwa_paired_sw] 881 out of 1492 Q17 discordant pairs are fixed.

[bwa_sai2sam_pe_core] time elapses: 4.13 sec

[bwa_sai2sam_pe_core] refine gapped alignments... 1.05 sec

[bwa_sai2sam_pe_core] print alignments... 2.20 sec

[bwa_sai2sam_pe_core] 99876864 sequences have been processed.

[bwa_sai2sam_pe_core] convert to sequence coordinate...

[infer_isize] (25, 50, 75) percentile: (453, 463, 473)

[infer_isize] low and high boundaries: 413 and 513 for estimating avg and std

[infer_isize] inferred external isize from 204541 pairs: 463.186 +/- 13.945

[infer_isize] skewness: -0.001; kurtosis: -0.125; ap_prior: 1.91e-05

[infer_isize] inferred maximum insert size: 560 (6.96 sigma)

[bwa_sai2sam_pe_core] time elapses: 33.18 sec

[bwa_sai2sam_pe_core] changing coordinates of 6088 alignments.

[bwa_sai2sam_pe_core] align unmapped mate...

[bwa_paired_sw] 10502 out of 11076 Q17 singletons are mated.

[bwa_paired_sw] 903 out of 1550 Q17 discordant pairs are fixed.

[bwa_sai2sam_pe_core] time elapses: 4.74 sec

[bwa_sai2sam_pe_core] refine gapped alignments... 1.05 sec

[bwa_sai2sam_pe_core] print alignments... 2.20 sec

[bwa_sai2sam_pe_core] 100139008 sequences have been processed.

[bwa_sai2sam_pe_core] convert to sequence coordinate...

[infer_isize] (25, 50, 75) percentile: (453, 463, 473)

[infer_isize] low and high boundaries: 413 and 513 for estimating avg and std

[infer_isize] inferred external isize from 204243 pairs: 463.134 +/- 13.966

[infer_isize] skewness: 0.005; kurtosis: -0.120; ap_prior: 1.79e-05

[infer_isize] inferred maximum insert size: 560 (6.96 sigma)

[bwa_sai2sam_pe_core] time elapses: 32.50 sec

[bwa_sai2sam_pe_core] changing coordinates of 6037 alignments.

[bwa_sai2sam_pe_core] align unmapped mate...

[bwa_paired_sw] 10947 out of 11509 Q17 singletons are mated.

[bwa_paired_sw] 880 out of 1492 Q17 discordant pairs are fixed.

[bwa_sai2sam_pe_core] time elapses: 4.87 sec

[bwa_sai2sam_pe_core] refine gapped alignments... 1.08 sec

[bwa_sai2sam_pe_core] print alignments... 2.19 sec

[bwa_sai2sam_pe_core] 100401152 sequences have been processed.

[bwa_sai2sam_pe_core] convert to sequence coordinate...

[infer_isize] (25, 50, 75) percentile: (453, 463, 473)

[infer_isize] low and high boundaries: 413 and 513 for estimating avg and std

[infer_isize] inferred external isize from 204448 pairs: 463.209 +/- 13.941

[infer_isize] skewness: -0.003; kurtosis: -0.121; ap_prior: 1.81e-05

[infer_isize] inferred maximum insert size: 560 (6.96 sigma)

[bwa_sai2sam_pe_core] time elapses: 31.22 sec

[bwa_sai2sam_pe_core] changing coordinates of 6053 alignments.

[bwa_sai2sam_pe_core] align unmapped mate...

[bwa_paired_sw] 10648 out of 11189 Q17 singletons are mated.

[bwa_paired_sw] 858 out of 1444 Q17 discordant pairs are fixed.

[bwa_sai2sam_pe_core] time elapses: 4.78 sec

[bwa_sai2sam_pe_core] refine gapped alignments... 1.08 sec

[bwa_sai2sam_pe_core] print alignments... 2.24 sec

[bwa_sai2sam_pe_core] 100663296 sequences have been processed.

[bwa_sai2sam_pe_core] convert to sequence coordinate...

[infer_isize] (25, 50, 75) percentile: (453, 463, 473)

[infer_isize] low and high boundaries: 413 and 513 for estimating avg and std

[infer_isize] inferred external isize from 204509 pairs: 463.169 +/- 13.936

[infer_isize] skewness: 0.005; kurtosis: -0.133; ap_prior: 1.92e-05

[infer_isize] inferred maximum insert size: 560 (6.96 sigma)

[bwa_sai2sam_pe_core] time elapses: 31.89 sec

[bwa_sai2sam_pe_core] changing coordinates of 6179 alignments.

[bwa_sai2sam_pe_core] align unmapped mate...

[bwa_paired_sw] 10240 out of 10804 Q17 singletons are mated.

[bwa_paired_sw] 851 out of 1473 Q17 discordant pairs are fixed.

[bwa_sai2sam_pe_core] time elapses: 4.44 sec

[bwa_sai2sam_pe_core] refine gapped alignments... 0.92 sec

[bwa_sai2sam_pe_core] print alignments... 2.12 sec

[bwa_sai2sam_pe_core] 100925440 sequences have been processed.

[bwa_sai2sam_pe_core] convert to sequence coordinate...

[infer_isize] (25, 50, 75) percentile: (453, 463, 473)

[infer_isize] low and high boundaries: 413 and 513 for estimating avg and std

[infer_isize] inferred external isize from 204489 pairs: 463.179 +/- 13.908

[infer_isize] skewness: 0.007; kurtosis: -0.133; ap_prior: 1.75e-05

[infer_isize] inferred maximum insert size: 560 (6.96 sigma)

[bwa_sai2sam_pe_core] time elapses: 26.84 sec

[bwa_sai2sam_pe_core] changing coordinates of 6011 alignments.

[bwa_sai2sam_pe_core] align unmapped mate...

[bwa_paired_sw] 10467 out of 11017 Q17 singletons are mated.

[bwa_paired_sw] 873 out of 1512 Q17 discordant pairs are fixed.

[bwa_sai2sam_pe_core] time elapses: 4.51 sec

[bwa_sai2sam_pe_core] refine gapped alignments... 0.92 sec

[bwa_sai2sam_pe_core] print alignments... 2.08 sec

[bwa_sai2sam_pe_core] 101187584 sequences have been processed.

[bwa_sai2sam_pe_core] convert to sequence coordinate...

[infer_isize] (25, 50, 75) percentile: (453, 463, 473)

[infer_isize] low and high boundaries: 413 and 513 for estimating avg and std

[infer_isize] inferred external isize from 204936 pairs: 463.128 +/- 13.984

[infer_isize] skewness: 0.007; kurtosis: -0.140; ap_prior: 1.68e-05

[infer_isize] inferred maximum insert size: 560 (6.96 sigma)

[bwa_sai2sam_pe_core] time elapses: 27.04 sec

[bwa_sai2sam_pe_core] changing coordinates of 5953 alignments.

[bwa_sai2sam_pe_core] align unmapped mate...

[bwa_paired_sw] 10270 out of 10830 Q17 singletons are mated.

[bwa_paired_sw] 904 out of 1510 Q17 discordant pairs are fixed.

[bwa_sai2sam_pe_core] time elapses: 4.49 sec

[bwa_sai2sam_pe_core] refine gapped alignments... 0.92 sec

[bwa_sai2sam_pe_core] print alignments... 2.10 sec

[bwa_sai2sam_pe_core] 101449728 sequences have been processed.

[bwa_sai2sam_pe_core] convert to sequence coordinate...

[infer_isize] (25, 50, 75) percentile: (453, 463, 473)

[infer_isize] low and high boundaries: 413 and 513 for estimating avg and std

[infer_isize] inferred external isize from 204922 pairs: 463.184 +/- 13.921

[infer_isize] skewness: 0.006; kurtosis: -0.136; ap_prior: 1.81e-05

[infer_isize] inferred maximum insert size: 560 (6.96 sigma)

[bwa_sai2sam_pe_core] time elapses: 27.63 sec

[bwa_sai2sam_pe_core] changing coordinates of 5998 alignments.

[bwa_sai2sam_pe_core] align unmapped mate...

[bwa_paired_sw] 10000 out of 10551 Q17 singletons are mated.

[bwa_paired_sw] 918 out of 1529 Q17 discordant pairs are fixed.

[bwa_sai2sam_pe_core] time elapses: 4.37 sec

[bwa_sai2sam_pe_core] refine gapped alignments... 0.89 sec

[bwa_sai2sam_pe_core] print alignments... 2.10 sec

[bwa_sai2sam_pe_core] 101711872 sequences have been processed.

[bwa_sai2sam_pe_core] convert to sequence coordinate...

[infer_isize] (25, 50, 75) percentile: (453, 463, 473)

[infer_isize] low and high boundaries: 413 and 513 for estimating avg and std

[infer_isize] inferred external isize from 204654 pairs: 463.118 +/- 13.944

[infer_isize] skewness: -0.002; kurtosis: -0.120; ap_prior: 1.73e-05

[infer_isize] inferred maximum insert size: 560 (6.96 sigma)

[bwa_sai2sam_pe_core] time elapses: 23.37 sec

[bwa_sai2sam_pe_core] changing coordinates of 5967 alignments.

[bwa_sai2sam_pe_core] align unmapped mate...

[bwa_paired_sw] 10269 out of 10801 Q17 singletons are mated.

[bwa_paired_sw] 889 out of 1465 Q17 discordant pairs are fixed.

[bwa_sai2sam_pe_core] time elapses: 4.38 sec

[bwa_sai2sam_pe_core] refine gapped alignments... 0.90 sec

[bwa_sai2sam_pe_core] print alignments... 2.09 sec

[bwa_sai2sam_pe_core] 101974016 sequences have been processed.

[bwa_sai2sam_pe_core] convert to sequence coordinate...

[infer_isize] (25, 50, 75) percentile: (453, 463, 473)

[infer_isize] low and high boundaries: 413 and 513 for estimating avg and std

[infer_isize] inferred external isize from 204406 pairs: 463.106 +/- 13.942

[infer_isize] skewness: 0.001; kurtosis: -0.124; ap_prior: 1.79e-05

[infer_isize] inferred maximum insert size: 560 (6.96 sigma)

[bwa_sai2sam_pe_core] time elapses: 26.08 sec

[bwa_sai2sam_pe_core] changing coordinates of 5982 alignments.

[bwa_sai2sam_pe_core] align unmapped mate...

[bwa_paired_sw] 10490 out of 11051 Q17 singletons are mated.

[bwa_paired_sw] 922 out of 1539 Q17 discordant pairs are fixed.

[bwa_sai2sam_pe_core] time elapses: 4.52 sec

[bwa_sai2sam_pe_core] refine gapped alignments... 0.91 sec

[bwa_sai2sam_pe_core] print alignments... 2.07 sec

[bwa_sai2sam_pe_core] 102236160 sequences have been processed.

[bwa_sai2sam_pe_core] convert to sequence coordinate...

[infer_isize] (25, 50, 75) percentile: (453, 463, 473)

[infer_isize] low and high boundaries: 413 and 513 for estimating avg and std

[infer_isize] inferred external isize from 204294 pairs: 463.190 +/- 13.922

[infer_isize] skewness: 0.002; kurtosis: -0.159; ap_prior: 1.71e-05

[infer_isize] inferred maximum insert size: 560 (6.96 sigma)

[bwa_sai2sam_pe_core] time elapses: 24.77 sec

[bwa_sai2sam_pe_core] changing coordinates of 5976 alignments.

[bwa_sai2sam_pe_core] align unmapped mate...

[bwa_paired_sw] 10635 out of 11198 Q17 singletons are mated.

[bwa_paired_sw] 857 out of 1505 Q17 discordant pairs are fixed.

[bwa_sai2sam_pe_core] time elapses: 4.52 sec

[bwa_sai2sam_pe_core] refine gapped alignments... 0.88 sec

[bwa_sai2sam_pe_core] print alignments... 2.07 sec

[bwa_sai2sam_pe_core] 102498304 sequences have been processed.

[bwa_sai2sam_pe_core] convert to sequence coordinate...

[infer_isize] (25, 50, 75) percentile: (453, 463, 473)

[infer_isize] low and high boundaries: 413 and 513 for estimating avg and std

[infer_isize] inferred external isize from 204347 pairs: 463.092 +/- 13.931

[infer_isize] skewness: -0.002; kurtosis: -0.127; ap_prior: 1.68e-05

[infer_isize] inferred maximum insert size: 560 (6.96 sigma)

[bwa_sai2sam_pe_core] time elapses: 23.31 sec

[bwa_sai2sam_pe_core] changing coordinates of 6064 alignments.

[bwa_sai2sam_pe_core] align unmapped mate...

[bwa_paired_sw] 10535 out of 11079 Q17 singletons are mated.

[bwa_paired_sw] 900 out of 1482 Q17 discordant pairs are fixed.

[bwa_sai2sam_pe_core] time elapses: 4.50 sec

[bwa_sai2sam_pe_core] refine gapped alignments... 0.89 sec

[bwa_sai2sam_pe_core] print alignments... 2.08 sec

[bwa_sai2sam_pe_core] 102760448 sequences have been processed.

[bwa_sai2sam_pe_core] convert to sequence coordinate...

[infer_isize] (25, 50, 75) percentile: (453, 463, 473)

[infer_isize] low and high boundaries: 413 and 513 for estimating avg and std

[infer_isize] inferred external isize from 204563 pairs: 463.165 +/- 13.969

[infer_isize] skewness: 0.008; kurtosis: -0.144; ap_prior: 1.62e-05

[infer_isize] inferred maximum insert size: 560 (6.96 sigma)

[bwa_sai2sam_pe_core] time elapses: 22.68 sec

[bwa_sai2sam_pe_core] changing coordinates of 5968 alignments.

[bwa_sai2sam_pe_core] align unmapped mate...

[bwa_paired_sw] 10614 out of 11195 Q17 singletons are mated.

[bwa_paired_sw] 802 out of 1354 Q17 discordant pairs are fixed.

[bwa_sai2sam_pe_core] time elapses: 4.45 sec

[bwa_sai2sam_pe_core] refine gapped alignments... 0.89 sec

[bwa_sai2sam_pe_core] print alignments... 2.08 sec

[bwa_sai2sam_pe_core] 103022592 sequences have been processed.

[bwa_sai2sam_pe_core] convert to sequence coordinate...

[infer_isize] (25, 50, 75) percentile: (453, 463, 473)

[infer_isize] low and high boundaries: 413 and 513 for estimating avg and std

[infer_isize] inferred external isize from 204455 pairs: 463.194 +/- 13.941

[infer_isize] skewness: 0.006; kurtosis: -0.142; ap_prior: 1.65e-05

[infer_isize] inferred maximum insert size: 560 (6.96 sigma)

[bwa_sai2sam_pe_core] time elapses: 22.96 sec

[bwa_sai2sam_pe_core] changing coordinates of 5996 alignments.

[bwa_sai2sam_pe_core] align unmapped mate...

[bwa_paired_sw] 10431 out of 11013 Q17 singletons are mated.

[bwa_paired_sw] 954 out of 1509 Q17 discordant pairs are fixed.

[bwa_sai2sam_pe_core] time elapses: 4.48 sec

[bwa_sai2sam_pe_core] refine gapped alignments... 0.92 sec

[bwa_sai2sam_pe_core] print alignments... 2.05 sec

[bwa_sai2sam_pe_core] 103284736 sequences have been processed.

[bwa_sai2sam_pe_core] convert to sequence coordinate...

[infer_isize] (25, 50, 75) percentile: (453, 463, 473)

[infer_isize] low and high boundaries: 413 and 513 for estimating avg and std

[infer_isize] inferred external isize from 204462 pairs: 463.114 +/- 13.954

[infer_isize] skewness: 0.002; kurtosis: -0.136; ap_prior: 1.78e-05

[infer_isize] inferred maximum insert size: 560 (6.96 sigma)

[bwa_sai2sam_pe_core] time elapses: 23.06 sec

[bwa_sai2sam_pe_core] changing coordinates of 5924 alignments.

[bwa_sai2sam_pe_core] align unmapped mate...

[bwa_paired_sw] 10457 out of 11004 Q17 singletons are mated.

[bwa_paired_sw] 906 out of 1472 Q17 discordant pairs are fixed.

[bwa_sai2sam_pe_core] time elapses: 4.48 sec

[bwa_sai2sam_pe_core] refine gapped alignments... 0.89 sec

[bwa_sai2sam_pe_core] print alignments... 2.09 sec

[bwa_sai2sam_pe_core] 103546880 sequences have been processed.

[bwa_sai2sam_pe_core] convert to sequence coordinate...

[infer_isize] (25, 50, 75) percentile: (453, 463, 473)

[infer_isize] low and high boundaries: 413 and 513 for estimating avg and std

[infer_isize] inferred external isize from 203959 pairs: 463.118 +/- 13.951

[infer_isize] skewness: -0.005; kurtosis: -0.130; ap_prior: 1.74e-05

[infer_isize] inferred maximum insert size: 560 (6.96 sigma)

[bwa_sai2sam_pe_core] time elapses: 24.27 sec

[bwa_sai2sam_pe_core] changing coordinates of 5931 alignments.

[bwa_sai2sam_pe_core] align unmapped mate...

[bwa_paired_sw] 10891 out of 11446 Q17 singletons are mated.

[bwa_paired_sw] 845 out of 1471 Q17 discordant pairs are fixed.

[bwa_sai2sam_pe_core] time elapses: 4.62 sec

[bwa_sai2sam_pe_core] refine gapped alignments... 0.93 sec

[bwa_sai2sam_pe_core] print alignments... 2.10 sec

[bwa_sai2sam_pe_core] 103809024 sequences have been processed.

[bwa_sai2sam_pe_core] convert to sequence coordinate...

[infer_isize] (25, 50, 75) percentile: (453, 463, 473)

[infer_isize] low and high boundaries: 413 and 513 for estimating avg and std

[infer_isize] inferred external isize from 203585 pairs: 463.085 +/- 13.917

[infer_isize] skewness: 0.007; kurtosis: -0.138; ap_prior: 1.87e-05

[infer_isize] inferred maximum insert size: 560 (6.96 sigma)

[bwa_sai2sam_pe_core] time elapses: 23.89 sec

[bwa_sai2sam_pe_core] changing coordinates of 6112 alignments.

[bwa_sai2sam_pe_core] align unmapped mate...

[bwa_paired_sw] 10946 out of 11521 Q17 singletons are mated.

[bwa_paired_sw] 939 out of 1583 Q17 discordant pairs are fixed.

[bwa_sai2sam_pe_core] time elapses: 4.75 sec

[bwa_sai2sam_pe_core] refine gapped alignments... 0.96 sec

[bwa_sai2sam_pe_core] print alignments... 2.17 sec

[bwa_sai2sam_pe_core] 104071168 sequences have been processed.

[bwa_sai2sam_pe_core] convert to sequence coordinate...

[infer_isize] (25, 50, 75) percentile: (453, 463, 473)

[infer_isize] low and high boundaries: 413 and 513 for estimating avg and std

[infer_isize] inferred external isize from 203974 pairs: 463.073 +/- 13.977

[infer_isize] skewness: 0.009; kurtosis: -0.132; ap_prior: 1.74e-05

[infer_isize] inferred maximum insert size: 560 (6.96 sigma)

[bwa_sai2sam_pe_core] time elapses: 32.13 sec

[bwa_sai2sam_pe_core] changing coordinates of 5965 alignments.

[bwa_sai2sam_pe_core] align unmapped mate...

[bwa_paired_sw] 10794 out of 11367 Q17 singletons are mated.

[bwa_paired_sw] 899 out of 1467 Q17 discordant pairs are fixed.

[bwa_sai2sam_pe_core] time elapses: 4.84 sec

[bwa_sai2sam_pe_core] refine gapped alignments... 1.01 sec

[bwa_sai2sam_pe_core] print alignments... 2.14 sec

[bwa_sai2sam_pe_core] 104333312 sequences have been processed.

[bwa_sai2sam_pe_core] convert to sequence coordinate...

[infer_isize] (25, 50, 75) percentile: (453, 463, 473)

[infer_isize] low and high boundaries: 413 and 513 for estimating avg and std

[infer_isize] inferred external isize from 203490 pairs: 463.104 +/- 13.930

[infer_isize] skewness: -0.011; kurtosis: -0.139; ap_prior: 1.70e-05

[infer_isize] inferred maximum insert size: 560 (6.96 sigma)

[bwa_sai2sam_pe_core] time elapses: 26.78 sec

[bwa_sai2sam_pe_core] changing coordinates of 6015 alignments.

[bwa_sai2sam_pe_core] align unmapped mate...

[bwa_paired_sw] 11089 out of 11629 Q17 singletons are mated.

[bwa_paired_sw] 879 out of 1508 Q17 discordant pairs are fixed.

[bwa_sai2sam_pe_core] time elapses: 4.75 sec

[bwa_sai2sam_pe_core] refine gapped alignments... 0.95 sec

[bwa_sai2sam_pe_core] print alignments... 2.14 sec

[bwa_sai2sam_pe_core] 104595456 sequences have been processed.

[bwa_sai2sam_pe_core] convert to sequence coordinate...

[infer_isize] (25, 50, 75) percentile: (453, 463, 473)

[infer_isize] low and high boundaries: 413 and 513 for estimating avg and std

[infer_isize] inferred external isize from 202571 pairs: 463.101 +/- 13.943

[infer_isize] skewness: 0.012; kurtosis: -0.143; ap_prior: 1.77e-05

[infer_isize] inferred maximum insert size: 560 (6.96 sigma)

[bwa_sai2sam_pe_core] time elapses: 40.48 sec

[bwa_sai2sam_pe_core] changing coordinates of 6026 alignments.

[bwa_sai2sam_pe_core] align unmapped mate...

[bwa_paired_sw] 12400 out of 12972 Q17 singletons are mated.

[bwa_paired_sw] 907 out of 1493 Q17 discordant pairs are fixed.

[bwa_sai2sam_pe_core] time elapses: 5.23 sec

[bwa_sai2sam_pe_core] refine gapped alignments... 0.96 sec

[bwa_sai2sam_pe_core] print alignments... 2.10 sec

[bwa_sai2sam_pe_core] 104857600 sequences have been processed.

[bwa_sai2sam_pe_core] convert to sequence coordinate...

[infer_isize] (25, 50, 75) percentile: (453, 463, 473)

[infer_isize] low and high boundaries: 413 and 513 for estimating avg and std

[infer_isize] inferred external isize from 203739 pairs: 463.088 +/- 13.919

[infer_isize] skewness: 0.007; kurtosis: -0.135; ap_prior: 1.89e-05

[infer_isize] inferred maximum insert size: 560 (6.96 sigma)

[bwa_sai2sam_pe_core] time elapses: 30.10 sec

[bwa_sai2sam_pe_core] changing coordinates of 6019 alignments.

[bwa_sai2sam_pe_core] align unmapped mate...

[bwa_paired_sw] 11262 out of 11815 Q17 singletons are mated.

[bwa_paired_sw] 941 out of 1515 Q17 discordant pairs are fixed.

[bwa_sai2sam_pe_core] time elapses: 4.70 sec

[bwa_sai2sam_pe_core] refine gapped alignments... 0.90 sec

[bwa_sai2sam_pe_core] print alignments... 2.10 sec

[bwa_sai2sam_pe_core] 105119744 sequences have been processed.

[bwa_sai2sam_pe_core] convert to sequence coordinate...

[infer_isize] (25, 50, 75) percentile: (453, 463, 473)

[infer_isize] low and high boundaries: 413 and 513 for estimating avg and std

[infer_isize] inferred external isize from 203594 pairs: 463.172 +/- 13.936

[infer_isize] skewness: -0.002; kurtosis: -0.122; ap_prior: 1.78e-05

[infer_isize] inferred maximum insert size: 560 (6.96 sigma)

[bwa_sai2sam_pe_core] time elapses: 21.74 sec

[bwa_sai2sam_pe_core] changing coordinates of 6176 alignments.

[bwa_sai2sam_pe_core] align unmapped mate...

[bwa_paired_sw] 11156 out of 11769 Q17 singletons are mated.

[bwa_paired_sw] 859 out of 1476 Q17 discordant pairs are fixed.

[bwa_sai2sam_pe_core] time elapses: 4.66 sec

[bwa_sai2sam_pe_core] refine gapped alignments... 0.90 sec

[bwa_sai2sam_pe_core] print alignments... 2.10 sec

[bwa_sai2sam_pe_core] 105381888 sequences have been processed.

[bwa_sai2sam_pe_core] convert to sequence coordinate...

[infer_isize] (25, 50, 75) percentile: (453, 463, 473)

[infer_isize] low and high boundaries: 413 and 513 for estimating avg and std

[infer_isize] inferred external isize from 203334 pairs: 463.121 +/- 13.919

[infer_isize] skewness: 0.005; kurtosis: -0.124; ap_prior: 1.68e-05

[infer_isize] inferred maximum insert size: 560 (6.96 sigma)

[bwa_sai2sam_pe_core] time elapses: 22.24 sec

[bwa_sai2sam_pe_core] changing coordinates of 5848 alignments.

[bwa_sai2sam_pe_core] align unmapped mate...

[bwa_paired_sw] 11347 out of 11902 Q17 singletons are mated.

[bwa_paired_sw] 941 out of 1555 Q17 discordant pairs are fixed.

[bwa_sai2sam_pe_core] time elapses: 4.73 sec

[bwa_sai2sam_pe_core] refine gapped alignments... 0.89 sec

[bwa_sai2sam_pe_core] print alignments... 2.08 sec

[bwa_sai2sam_pe_core] 105644032 sequences have been processed.

[bwa_sai2sam_pe_core] convert to sequence coordinate...

[infer_isize] (25, 50, 75) percentile: (453, 463, 473)

[infer_isize] low and high boundaries: 413 and 513 for estimating avg and std

[infer_isize] inferred external isize from 203747 pairs: 463.119 +/- 13.930

[infer_isize] skewness: 0.004; kurtosis: -0.130; ap_prior: 1.69e-05

[infer_isize] inferred maximum insert size: 560 (6.96 sigma)

[bwa_sai2sam_pe_core] time elapses: 21.93 sec

[bwa_sai2sam_pe_core] changing coordinates of 6056 alignments.

[bwa_sai2sam_pe_core] align unmapped mate...

[bwa_paired_sw] 11236 out of 11770 Q17 singletons are mated.

[bwa_paired_sw] 834 out of 1434 Q17 discordant pairs are fixed.

[bwa_sai2sam_pe_core] time elapses: 4.63 sec

[bwa_sai2sam_pe_core] refine gapped alignments... 0.91 sec

[bwa_sai2sam_pe_core] print alignments... 2.08 sec

[bwa_sai2sam_pe_core] 105906176 sequences have been processed.

[bwa_sai2sam_pe_core] convert to sequence coordinate...

[infer_isize] (25, 50, 75) percentile: (453, 463, 473)

[infer_isize] low and high boundaries: 413 and 513 for estimating avg and std

[infer_isize] inferred external isize from 203227 pairs: 463.113 +/- 13.930

[infer_isize] skewness: 0.011; kurtosis: -0.133; ap_prior: 1.74e-05

[infer_isize] inferred maximum insert size: 560 (6.96 sigma)

[bwa_sai2sam_pe_core] time elapses: 21.96 sec

[bwa_sai2sam_pe_core] changing coordinates of 5923 alignments.

[bwa_sai2sam_pe_core] align unmapped mate...

[bwa_paired_sw] 11473 out of 12028 Q17 singletons are mated.

[bwa_paired_sw] 906 out of 1528 Q17 discordant pairs are fixed.

[bwa_sai2sam_pe_core] time elapses: 16.57 sec

[bwa_sai2sam_pe_core] refine gapped alignments... 4.03 sec

[bwa_sai2sam_pe_core] print alignments... 11.57 sec

[bwa_sai2sam_pe_core] 106168320 sequences have been processed.

[bwa_sai2sam_pe_core] convert to sequence coordinate...

[infer_isize] (25, 50, 75) percentile: (453, 463, 473)

[infer_isize] low and high boundaries: 413 and 513 for estimating avg and std

[infer_isize] inferred external isize from 203519 pairs: 463.077 +/- 13.950

[infer_isize] skewness: 0.007; kurtosis: -0.141; ap_prior: 1.66e-05

[infer_isize] inferred maximum insert size: 560 (6.96 sigma)

[bwa_sai2sam_pe_core] time elapses: 114.56 sec

[bwa_sai2sam_pe_core] changing coordinates of 5860 alignments.

[bwa_sai2sam_pe_core] align unmapped mate...

[bwa_paired_sw] 11415 out of 11961 Q17 singletons are mated.

[bwa_paired_sw] 878 out of 1457 Q17 discordant pairs are fixed.

[bwa_sai2sam_pe_core] time elapses: 23.34 sec

[bwa_sai2sam_pe_core] refine gapped alignments... 4.60 sec

[bwa_sai2sam_pe_core] print alignments... 10.49 sec

[bwa_sai2sam_pe_core] 106430464 sequences have been processed.

[bwa_sai2sam_pe_core] convert to sequence coordinate...

[infer_isize] (25, 50, 75) percentile: (453, 463, 473)

[infer_isize] low and high boundaries: 413 and 513 for estimating avg and std

[infer_isize] inferred external isize from 203128 pairs: 463.103 +/- 13.974

[infer_isize] skewness: -0.005; kurtosis: -0.134; ap_prior: 1.79e-05

[infer_isize] inferred maximum insert size: 560 (6.96 sigma)

[bwa_sai2sam_pe_core] time elapses: 118.53 sec

[bwa_sai2sam_pe_core] changing coordinates of 5939 alignments.

[bwa_sai2sam_pe_core] align unmapped mate...

[bwa_paired_sw] 11512 out of 12072 Q17 singletons are mated.

[bwa_paired_sw] 882 out of 1503 Q17 discordant pairs are fixed.

[bwa_sai2sam_pe_core] time elapses: 23.32 sec

[bwa_sai2sam_pe_core] refine gapped alignments... 4.58 sec

[bwa_sai2sam_pe_core] print alignments... 10.63 sec

[bwa_sai2sam_pe_core] 106692608 sequences have been processed.

[bwa_sai2sam_pe_core] convert to sequence coordinate...

[infer_isize] (25, 50, 75) percentile: (453, 463, 473)

[infer_isize] low and high boundaries: 413 and 513 for estimating avg and std

[infer_isize] inferred external isize from 203126 pairs: 463.066 +/- 13.965

[infer_isize] skewness: -0.004; kurtosis: -0.131; ap_prior: 1.81e-05

[infer_isize] inferred maximum insert size: 560 (6.96 sigma)

[bwa_sai2sam_pe_core] time elapses: 118.22 sec

[bwa_sai2sam_pe_core] changing coordinates of 6019 alignments.

[bwa_sai2sam_pe_core] align unmapped mate...

[bwa_paired_sw] 11399 out of 11908 Q17 singletons are mated.

[bwa_paired_sw] 904 out of 1518 Q17 discordant pairs are fixed.

[bwa_sai2sam_pe_core] time elapses: 23.75 sec

[bwa_sai2sam_pe_core] refine gapped alignments... 5.07 sec

[bwa_sai2sam_pe_core] print alignments... 11.68 sec

[bwa_sai2sam_pe_core] 106954752 sequences have been processed.

[bwa_sai2sam_pe_core] convert to sequence coordinate...

[infer_isize] (25, 50, 75) percentile: (453, 463, 473)

[infer_isize] low and high boundaries: 413 and 513 for estimating avg and std

[infer_isize] inferred external isize from 202518 pairs: 463.033 +/- 13.955

[infer_isize] skewness: 0.004; kurtosis: -0.112; ap_prior: 1.79e-05

[infer_isize] inferred maximum insert size: 560 (6.96 sigma)

[bwa_sai2sam_pe_core] time elapses: 70.17 sec

[bwa_sai2sam_pe_core] changing coordinates of 5953 alignments.

[bwa_sai2sam_pe_core] align unmapped mate...

[bwa_paired_sw] 12077 out of 12660 Q17 singletons are mated.

[bwa_paired_sw] 930 out of 1529 Q17 discordant pairs are fixed.

[bwa_sai2sam_pe_core] time elapses: 5.13 sec

[bwa_sai2sam_pe_core] refine gapped alignments... 1.00 sec

[bwa_sai2sam_pe_core] print alignments... 2.10 sec

[bwa_sai2sam_pe_core] 107216896 sequences have been processed.

[bwa_sai2sam_pe_core] convert to sequence coordinate...

[infer_isize] (25, 50, 75) percentile: (453, 463, 473)

[infer_isize] low and high boundaries: 413 and 513 for estimating avg and std

[infer_isize] inferred external isize from 202503 pairs: 463.093 +/- 13.906

[infer_isize] skewness: 0.005; kurtosis: -0.116; ap_prior: 1.67e-05

[infer_isize] inferred maximum insert size: 560 (6.96 sigma)

[bwa_sai2sam_pe_core] time elapses: 30.71 sec

[bwa_sai2sam_pe_core] changing coordinates of 5982 alignments.

[bwa_sai2sam_pe_core] align unmapped mate...

[bwa_paired_sw] 11801 out of 12367 Q17 singletons are mated.

[bwa_paired_sw] 890 out of 1490 Q17 discordant pairs are fixed.

[bwa_sai2sam_pe_core] time elapses: 5.01 sec

[bwa_sai2sam_pe_core] refine gapped alignments... 1.01 sec

[bwa_sai2sam_pe_core] print alignments... 2.11 sec

[bwa_sai2sam_pe_core] 107479040 sequences have been processed.

[bwa_sai2sam_pe_core] convert to sequence coordinate...

[infer_isize] (25, 50, 75) percentile: (453, 463, 473)

[infer_isize] low and high boundaries: 413 and 513 for estimating avg and std

[infer_isize] inferred external isize from 202806 pairs: 463.060 +/- 13.968

[infer_isize] skewness: 0.004; kurtosis: -0.132; ap_prior: 1.72e-05

[infer_isize] inferred maximum insert size: 560 (6.96 sigma)

[bwa_sai2sam_pe_core] time elapses: 31.59 sec

[bwa_sai2sam_pe_core] changing coordinates of 5919 alignments.

[bwa_sai2sam_pe_core] align unmapped mate...

[bwa_paired_sw] 12016 out of 12575 Q17 singletons are mated.

[bwa_paired_sw] 874 out of 1415 Q17 discordant pairs are fixed.

[bwa_sai2sam_pe_core] time elapses: 4.86 sec

[bwa_sai2sam_pe_core] refine gapped alignments... 0.92 sec

[bwa_sai2sam_pe_core] print alignments... 2.10 sec

[bwa_sai2sam_pe_core] 107741184 sequences have been processed.

[bwa_sai2sam_pe_core] convert to sequence coordinate...

[infer_isize] (25, 50, 75) percentile: (453, 463, 473)

[infer_isize] low and high boundaries: 413 and 513 for estimating avg and std

[infer_isize] inferred external isize from 202800 pairs: 463.101 +/- 13.941

[infer_isize] skewness: 0.009; kurtosis: -0.141; ap_prior: 1.68e-05

[infer_isize] inferred maximum insert size: 560 (6.96 sigma)

[bwa_sai2sam_pe_core] time elapses: 27.08 sec

[bwa_sai2sam_pe_core] changing coordinates of 5927 alignments.

[bwa_sai2sam_pe_core] align unmapped mate...

[bwa_paired_sw] 11976 out of 12492 Q17 singletons are mated.

[bwa_paired_sw] 932 out of 1506 Q17 discordant pairs are fixed.

[bwa_sai2sam_pe_core] time elapses: 4.91 sec

[bwa_sai2sam_pe_core] refine gapped alignments... 0.92 sec

[bwa_sai2sam_pe_core] print alignments... 2.05 sec

[bwa_sai2sam_pe_core] 108003328 sequences have been processed.

[bwa_sai2sam_pe_core] convert to sequence coordinate...

[infer_isize] (25, 50, 75) percentile: (453, 463, 473)

[infer_isize] low and high boundaries: 413 and 513 for estimating avg and std

[infer_isize] inferred external isize from 202113 pairs: 463.045 +/- 13.976

[infer_isize] skewness: 0.008; kurtosis: -0.133; ap_prior: 1.59e-05

[infer_isize] inferred maximum insert size: 560 (6.96 sigma)

[bwa_sai2sam_pe_core] time elapses: 29.35 sec

[bwa_sai2sam_pe_core] changing coordinates of 5948 alignments.

[bwa_sai2sam_pe_core] align unmapped mate...

[bwa_paired_sw] 11994 out of 12565 Q17 singletons are mated.

[bwa_paired_sw] 890 out of 1445 Q17 discordant pairs are fixed.

[bwa_sai2sam_pe_core] time elapses: 4.94 sec

[bwa_sai2sam_pe_core] refine gapped alignments... 0.97 sec

[bwa_sai2sam_pe_core] print alignments... 2.13 sec

[bwa_sai2sam_pe_core] 108265472 sequences have been processed.

[bwa_sai2sam_pe_core] convert to sequence coordinate...

[infer_isize] (25, 50, 75) percentile: (453, 463, 473)

[infer_isize] low and high boundaries: 413 and 513 for estimating avg and std

[infer_isize] inferred external isize from 202801 pairs: 463.054 +/- 13.933

[infer_isize] skewness: 0.012; kurtosis: -0.142; ap_prior: 1.82e-05

[infer_isize] inferred maximum insert size: 560 (6.96 sigma)

[bwa_sai2sam_pe_core] time elapses: 29.30 sec

[bwa_sai2sam_pe_core] changing coordinates of 5924 alignments.

[bwa_sai2sam_pe_core] align unmapped mate...

[bwa_paired_sw] 11831 out of 12371 Q17 singletons are mated.

[bwa_paired_sw] 929 out of 1507 Q17 discordant pairs are fixed.

[bwa_sai2sam_pe_core] time elapses: 4.91 sec

[bwa_sai2sam_pe_core] refine gapped alignments... 0.97 sec

[bwa_sai2sam_pe_core] print alignments... 2.13 sec

[bwa_sai2sam_pe_core] 108527616 sequences have been processed.

[bwa_sai2sam_pe_core] convert to sequence coordinate...

[infer_isize] (25, 50, 75) percentile: (453, 463, 473)

[infer_isize] low and high boundaries: 413 and 513 for estimating avg and std

[infer_isize] inferred external isize from 201484 pairs: 463.064 +/- 13.941

[infer_isize] skewness: -0.001; kurtosis: -0.126; ap_prior: 1.66e-05

[infer_isize] inferred maximum insert size: 560 (6.96 sigma)

[bwa_sai2sam_pe_core] time elapses: 29.41 sec

[bwa_sai2sam_pe_core] changing coordinates of 6088 alignments.

[bwa_sai2sam_pe_core] align unmapped mate...

[bwa_paired_sw] 12766 out of 13361 Q17 singletons are mated.

[bwa_paired_sw] 837 out of 1374 Q17 discordant pairs are fixed.

[bwa_sai2sam_pe_core] time elapses: 5.12 sec

[bwa_sai2sam_pe_core] refine gapped alignments... 0.97 sec

[bwa_sai2sam_pe_core] print alignments... 2.11 sec

[bwa_sai2sam_pe_core] 108789760 sequences have been processed.

[bwa_sai2sam_pe_core] convert to sequence coordinate...

[infer_isize] (25, 50, 75) percentile: (453, 463, 473)

[infer_isize] low and high boundaries: 413 and 513 for estimating avg and std

[infer_isize] inferred external isize from 202409 pairs: 463.058 +/- 13.956

[infer_isize] skewness: 0.000; kurtosis: -0.134; ap_prior: 1.69e-05

[infer_isize] inferred maximum insert size: 560 (6.96 sigma)

[bwa_sai2sam_pe_core] time elapses: 29.03 sec

[bwa_sai2sam_pe_core] changing coordinates of 5898 alignments.

[bwa_sai2sam_pe_core] align unmapped mate...

[bwa_paired_sw] 12125 out of 12692 Q17 singletons are mated.

[bwa_paired_sw] 912 out of 1477 Q17 discordant pairs are fixed.

[bwa_sai2sam_pe_core] time elapses: 4.91 sec

[bwa_sai2sam_pe_core] refine gapped alignments... 0.96 sec

[bwa_sai2sam_pe_core] print alignments... 2.11 sec

[bwa_sai2sam_pe_core] 109051904 sequences have been processed.

[bwa_sai2sam_pe_core] convert to sequence coordinate...

[infer_isize] (25, 50, 75) percentile: (453, 463, 473)

[infer_isize] low and high boundaries: 413 and 513 for estimating avg and std

[infer_isize] inferred external isize from 202335 pairs: 463.021 +/- 13.920

[infer_isize] skewness: 0.007; kurtosis: -0.123; ap_prior: 1.71e-05

[infer_isize] inferred maximum insert size: 560 (6.96 sigma)

[bwa_sai2sam_pe_core] time elapses: 22.64 sec

[bwa_sai2sam_pe_core] changing coordinates of 5838 alignments.

[bwa_sai2sam_pe_core] align unmapped mate...

[bwa_paired_sw] 12269 out of 12797 Q17 singletons are mated.

[bwa_paired_sw] 915 out of 1503 Q17 discordant pairs are fixed.

[bwa_sai2sam_pe_core] time elapses: 4.96 sec

[bwa_sai2sam_pe_core] refine gapped alignments... 0.91 sec

[bwa_sai2sam_pe_core] print alignments... 2.06 sec

[bwa_sai2sam_pe_core] 109314048 sequences have been processed.

[bwa_sai2sam_pe_core] convert to sequence coordinate...

[infer_isize] (25, 50, 75) percentile: (453, 463, 473)

[infer_isize] low and high boundaries: 413 and 513 for estimating avg and std

[infer_isize] inferred external isize from 201436 pairs: 463.035 +/- 13.944

[infer_isize] skewness: 0.006; kurtosis: -0.146; ap_prior: 1.78e-05

[infer_isize] inferred maximum insert size: 560 (6.96 sigma)

[bwa_sai2sam_pe_core] time elapses: 23.60 sec

[bwa_sai2sam_pe_core] changing coordinates of 5866 alignments.

[bwa_sai2sam_pe_core] align unmapped mate...

[bwa_paired_sw] 12946 out of 13476 Q17 singletons are mated.

[bwa_paired_sw] 884 out of 1470 Q17 discordant pairs are fixed.

[bwa_sai2sam_pe_core] time elapses: 5.16 sec

[bwa_sai2sam_pe_core] refine gapped alignments... 0.94 sec

[bwa_sai2sam_pe_core] print alignments... 2.05 sec

[bwa_sai2sam_pe_core] 109576192 sequences have been processed.

[bwa_sai2sam_pe_core] convert to sequence coordinate...

[infer_isize] (25, 50, 75) percentile: (453, 463, 473)

[infer_isize] low and high boundaries: 413 and 513 for estimating avg and std

[infer_isize] inferred external isize from 201445 pairs: 463.061 +/- 13.923

[infer_isize] skewness: 0.010; kurtosis: -0.138; ap_prior: 1.70e-05

[infer_isize] inferred maximum insert size: 560 (6.96 sigma)

[bwa_sai2sam_pe_core] time elapses: 25.19 sec

[bwa_sai2sam_pe_core] changing coordinates of 5921 alignments.

[bwa_sai2sam_pe_core] align unmapped mate...

[bwa_paired_sw] 12965 out of 13505 Q17 singletons are mated.

[bwa_paired_sw] 865 out of 1439 Q17 discordant pairs are fixed.

[bwa_sai2sam_pe_core] time elapses: 5.19 sec

[bwa_sai2sam_pe_core] refine gapped alignments... 0.94 sec

[bwa_sai2sam_pe_core] print alignments... 2.09 sec

[bwa_sai2sam_pe_core] 109838336 sequences have been processed.

[bwa_sai2sam_pe_core] convert to sequence coordinate...

[infer_isize] (25, 50, 75) percentile: (453, 463, 473)

[infer_isize] low and high boundaries: 413 and 513 for estimating avg and std

[infer_isize] inferred external isize from 202857 pairs: 463.057 +/- 13.969

[infer_isize] skewness: 0.007; kurtosis: -0.137; ap_prior: 1.84e-05

[infer_isize] inferred maximum insert size: 560 (6.96 sigma)

[bwa_sai2sam_pe_core] time elapses: 30.48 sec

[bwa_sai2sam_pe_core] changing coordinates of 6030 alignments.

[bwa_sai2sam_pe_core] align unmapped mate...

[bwa_paired_sw] 12030 out of 12560 Q17 singletons are mated.

[bwa_paired_sw] 921 out of 1490 Q17 discordant pairs are fixed.

[bwa_sai2sam_pe_core] time elapses: 5.00 sec

[bwa_sai2sam_pe_core] refine gapped alignments... 0.97 sec

[bwa_sai2sam_pe_core] print alignments... 2.12 sec

[bwa_sai2sam_pe_core] 110100480 sequences have been processed.

[bwa_sai2sam_pe_core] convert to sequence coordinate...

[infer_isize] (25, 50, 75) percentile: (453, 463, 473)

[infer_isize] low and high boundaries: 413 and 513 for estimating avg and std

[infer_isize] inferred external isize from 202202 pairs: 462.994 +/- 13.961

[infer_isize] skewness: 0.008; kurtosis: -0.127; ap_prior: 1.82e-05

[infer_isize] inferred maximum insert size: 560 (6.96 sigma)

[bwa_sai2sam_pe_core] time elapses: 31.13 sec

[bwa_sai2sam_pe_core] changing coordinates of 6046 alignments.

[bwa_sai2sam_pe_core] align unmapped mate...

[bwa_paired_sw] 12359 out of 12940 Q17 singletons are mated.

[bwa_paired_sw] 853 out of 1448 Q17 discordant pairs are fixed.

[bwa_sai2sam_pe_core] time elapses: 5.08 sec

[bwa_sai2sam_pe_core] refine gapped alignments... 0.99 sec

[bwa_sai2sam_pe_core] print alignments... 2.09 sec

[bwa_sai2sam_pe_core] 110362624 sequences have been processed.

[bwa_sai2sam_pe_core] convert to sequence coordinate...

[infer_isize] (25, 50, 75) percentile: (453, 463, 473)

[infer_isize] low and high boundaries: 413 and 513 for estimating avg and std

[infer_isize] inferred external isize from 201633 pairs: 463.063 +/- 13.977

[infer_isize] skewness: 0.009; kurtosis: -0.136; ap_prior: 1.61e-05

[infer_isize] inferred maximum insert size: 560 (6.96 sigma)

[bwa_sai2sam_pe_core] time elapses: 30.17 sec

[bwa_sai2sam_pe_core] changing coordinates of 5880 alignments.

[bwa_sai2sam_pe_core] align unmapped mate...

[bwa_paired_sw] 12986 out of 13507 Q17 singletons are mated.

[bwa_paired_sw] 881 out of 1428 Q17 discordant pairs are fixed.

[bwa_sai2sam_pe_core] time elapses: 5.28 sec

[bwa_sai2sam_pe_core] refine gapped alignments... 0.99 sec

[bwa_sai2sam_pe_core] print alignments... 2.11 sec

[bwa_sai2sam_pe_core] 110624768 sequences have been processed.

[bwa_sai2sam_pe_core] convert to sequence coordinate...

[infer_isize] (25, 50, 75) percentile: (453, 463, 473)

[infer_isize] low and high boundaries: 413 and 513 for estimating avg and std

[infer_isize] inferred external isize from 201993 pairs: 463.043 +/- 13.924

[infer_isize] skewness: 0.011; kurtosis: -0.135; ap_prior: 1.82e-05

[infer_isize] inferred maximum insert size: 560 (6.96 sigma)

[bwa_sai2sam_pe_core] time elapses: 28.90 sec

[bwa_sai2sam_pe_core] changing coordinates of 5764 alignments.

[bwa_sai2sam_pe_core] align unmapped mate...

[bwa_paired_sw] 12583 out of 13101 Q17 singletons are mated.

[bwa_paired_sw] 901 out of 1475 Q17 discordant pairs are fixed.

[bwa_sai2sam_pe_core] time elapses: 5.09 sec

[bwa_sai2sam_pe_core] refine gapped alignments... 0.97 sec

[bwa_sai2sam_pe_core] print alignments... 2.08 sec

[bwa_sai2sam_pe_core] 110886912 sequences have been processed.

[bwa_sai2sam_pe_core] convert to sequence coordinate...

[infer_isize] (25, 50, 75) percentile: (453, 463, 473)

[infer_isize] low and high boundaries: 413 and 513 for estimating avg and std

[infer_isize] inferred external isize from 200566 pairs: 462.969 +/- 13.926

[infer_isize] skewness: 0.015; kurtosis: -0.146; ap_prior: 1.82e-05

[infer_isize] inferred maximum insert size: 560 (6.96 sigma)

[bwa_sai2sam_pe_core] time elapses: 29.24 sec

[bwa_sai2sam_pe_core] changing coordinates of 6006 alignments.

[bwa_sai2sam_pe_core] align unmapped mate...

[bwa_paired_sw] 13491 out of 14040 Q17 singletons are mated.

[bwa_paired_sw] 917 out of 1529 Q17 discordant pairs are fixed.

[bwa_sai2sam_pe_core] time elapses: 5.36 sec

[bwa_sai2sam_pe_core] refine gapped alignments... 0.94 sec

[bwa_sai2sam_pe_core] print alignments... 2.08 sec

[bwa_sai2sam_pe_core] 111149056 sequences have been processed.

[bwa_sai2sam_pe_core] convert to sequence coordinate...

[infer_isize] (25, 50, 75) percentile: (453, 463, 473)

[infer_isize] low and high boundaries: 413 and 513 for estimating avg and std

[infer_isize] inferred external isize from 201154 pairs: 463.007 +/- 13.934

[infer_isize] skewness: 0.007; kurtosis: -0.151; ap_prior: 1.90e-05

[infer_isize] inferred maximum insert size: 560 (6.96 sigma)

[bwa_sai2sam_pe_core] time elapses: 29.32 sec

[bwa_sai2sam_pe_core] changing coordinates of 5917 alignments.

[bwa_sai2sam_pe_core] align unmapped mate...

[bwa_paired_sw] 13310 out of 13894 Q17 singletons are mated.

[bwa_paired_sw] 955 out of 1532 Q17 discordant pairs are fixed.

[bwa_sai2sam_pe_core] time elapses: 5.33 sec

[bwa_sai2sam_pe_core] refine gapped alignments... 0.97 sec

[bwa_sai2sam_pe_core] print alignments... 2.05 sec

[bwa_sai2sam_pe_core] 111411200 sequences have been processed.

[bwa_sai2sam_pe_core] convert to sequence coordinate...

[infer_isize] (25, 50, 75) percentile: (453, 463, 473)

[infer_isize] low and high boundaries: 413 and 513 for estimating avg and std

[infer_isize] inferred external isize from 198681 pairs: 462.940 +/- 13.946

[infer_isize] skewness: 0.003; kurtosis: -0.144; ap_prior: 1.76e-05

[infer_isize] inferred maximum insert size: 560 (6.96 sigma)

[bwa_sai2sam_pe_core] time elapses: 29.46 sec

[bwa_sai2sam_pe_core] changing coordinates of 5700 alignments.

[bwa_sai2sam_pe_core] align unmapped mate...

[bwa_paired_sw] 15279 out of 15891 Q17 singletons are mated.

[bwa_paired_sw] 849 out of 1385 Q17 discordant pairs are fixed.

[bwa_sai2sam_pe_core] time elapses: 5.83 sec

[bwa_sai2sam_pe_core] refine gapped alignments... 0.96 sec

[bwa_sai2sam_pe_core] print alignments... 2.10 sec

[bwa_sai2sam_pe_core] 111673344 sequences have been processed.

[bwa_sai2sam_pe_core] convert to sequence coordinate...

[infer_isize] (25, 50, 75) percentile: (453, 463, 473)

[infer_isize] low and high boundaries: 413 and 513 for estimating avg and std

[infer_isize] inferred external isize from 199671 pairs: 462.971 +/- 13.967

[infer_isize] skewness: 0.002; kurtosis: -0.134; ap_prior: 1.89e-05

[infer_isize] inferred maximum insert size: 560 (6.96 sigma)

[bwa_sai2sam_pe_core] time elapses: 28.99 sec

[bwa_sai2sam_pe_core] changing coordinates of 5782 alignments.

[bwa_sai2sam_pe_core] align unmapped mate...

[bwa_paired_sw] 14447 out of 15003 Q17 singletons are mated.

[bwa_paired_sw] 885 out of 1451 Q17 discordant pairs are fixed.

[bwa_sai2sam_pe_core] time elapses: 5.59 sec

[bwa_sai2sam_pe_core] refine gapped alignments... 0.99 sec

[bwa_sai2sam_pe_core] print alignments... 2.10 sec

[bwa_sai2sam_pe_core] 111935488 sequences have been processed.

[bwa_sai2sam_pe_core] convert to sequence coordinate...

[infer_isize] (25, 50, 75) percentile: (453, 463, 473)

[infer_isize] low and high boundaries: 413 and 513 for estimating avg and std

[infer_isize] inferred external isize from 200746 pairs: 463.019 +/- 13.971

[infer_isize] skewness: 0.004; kurtosis: -0.137; ap_prior: 1.86e-05

[infer_isize] inferred maximum insert size: 560 (6.96 sigma)

[bwa_sai2sam_pe_core] time elapses: 29.53 sec

[bwa_sai2sam_pe_core] changing coordinates of 5799 alignments.

[bwa_sai2sam_pe_core] align unmapped mate...

[bwa_paired_sw] 13569 out of 14112 Q17 singletons are mated.

[bwa_paired_sw] 896 out of 1454 Q17 discordant pairs are fixed.

[bwa_sai2sam_pe_core] time elapses: 5.37 sec

[bwa_sai2sam_pe_core] refine gapped alignments... 1.01 sec

[bwa_sai2sam_pe_core] print alignments... 2.15 sec

[bwa_sai2sam_pe_core] 112197632 sequences have been processed.

[bwa_sai2sam_pe_core] convert to sequence coordinate...

[infer_isize] (25, 50, 75) percentile: (453, 463, 473)

[infer_isize] low and high boundaries: 413 and 513 for estimating avg and std

[infer_isize] inferred external isize from 200430 pairs: 462.978 +/- 13.925

[infer_isize] skewness: 0.006; kurtosis: -0.138; ap_prior: 1.69e-05

[infer_isize] inferred maximum insert size: 560 (6.96 sigma)

[bwa_sai2sam_pe_core] time elapses: 27.82 sec

[bwa_sai2sam_pe_core] changing coordinates of 5670 alignments.

[bwa_sai2sam_pe_core] align unmapped mate...

[bwa_paired_sw] 14043 out of 14593 Q17 singletons are mated.

[bwa_paired_sw] 881 out of 1444 Q17 discordant pairs are fixed.

[bwa_sai2sam_pe_core] time elapses: 5.48 sec

[bwa_sai2sam_pe_core] refine gapped alignments... 1.01 sec

[bwa_sai2sam_pe_core] print alignments... 2.08 sec

[bwa_sai2sam_pe_core] 112459776 sequences have been processed.

[bwa_sai2sam_pe_core] convert to sequence coordinate...

[infer_isize] (25, 50, 75) percentile: (453, 463, 473)

[infer_isize] low and high boundaries: 413 and 513 for estimating avg and std

[infer_isize] inferred external isize from 199984 pairs: 462.952 +/- 13.944

[infer_isize] skewness: 0.004; kurtosis: -0.152; ap_prior: 1.55e-05

[infer_isize] inferred maximum insert size: 560 (6.96 sigma)

[bwa_sai2sam_pe_core] time elapses: 27.45 sec

[bwa_sai2sam_pe_core] changing coordinates of 5851 alignments.

[bwa_sai2sam_pe_core] align unmapped mate...

[bwa_paired_sw] 14259 out of 14801 Q17 singletons are mated.

[bwa_paired_sw] 914 out of 1476 Q17 discordant pairs are fixed.

[bwa_sai2sam_pe_core] time elapses: 5.56 sec

[bwa_sai2sam_pe_core] refine gapped alignments... 1.02 sec

[bwa_sai2sam_pe_core] print alignments... 2.13 sec

[bwa_sai2sam_pe_core] 112721920 sequences have been processed.

[bwa_sai2sam_pe_core] convert to sequence coordinate...

[infer_isize] (25, 50, 75) percentile: (453, 463, 473)

[infer_isize] low and high boundaries: 413 and 513 for estimating avg and std

[infer_isize] inferred external isize from 199387 pairs: 462.990 +/- 13.976

[infer_isize] skewness: -0.000; kurtosis: -0.142; ap_prior: 1.79e-05

[infer_isize] inferred maximum insert size: 560 (6.96 sigma)

[bwa_sai2sam_pe_core] time elapses: 28.05 sec

[bwa_sai2sam_pe_core] changing coordinates of 5947 alignments.

[bwa_sai2sam_pe_core] align unmapped mate...

[bwa_paired_sw] 14592 out of 15193 Q17 singletons are mated.

[bwa_paired_sw] 932 out of 1470 Q17 discordant pairs are fixed.

[bwa_sai2sam_pe_core] time elapses: 5.66 sec

[bwa_sai2sam_pe_core] refine gapped alignments... 1.05 sec

[bwa_sai2sam_pe_core] print alignments... 2.13 sec

[bwa_sai2sam_pe_core] 112984064 sequences have been processed.

[bwa_sai2sam_pe_core] convert to sequence coordinate...

[infer_isize] (25, 50, 75) percentile: (453, 463, 473)

[infer_isize] low and high boundaries: 413 and 513 for estimating avg and std

[infer_isize] inferred external isize from 198352 pairs: 462.915 +/- 13.962

[infer_isize] skewness: 0.007; kurtosis: -0.144; ap_prior: 1.87e-05

[infer_isize] inferred maximum insert size: 560 (6.96 sigma)

[bwa_sai2sam_pe_core] time elapses: 26.56 sec

[bwa_sai2sam_pe_core] changing coordinates of 5756 alignments.

[bwa_sai2sam_pe_core] align unmapped mate...

[bwa_paired_sw] 15383 out of 15981 Q17 singletons are mated.

[bwa_paired_sw] 926 out of 1473 Q17 discordant pairs are fixed.

[bwa_sai2sam_pe_core] time elapses: 5.87 sec

[bwa_sai2sam_pe_core] refine gapped alignments... 1.06 sec

[bwa_sai2sam_pe_core] print alignments... 2.07 sec

[bwa_sai2sam_pe_core] 113246208 sequences have been processed.

[bwa_sai2sam_pe_core] convert to sequence coordinate...

[infer_isize] (25, 50, 75) percentile: (453, 463, 473)

[infer_isize] low and high boundaries: 413 and 513 for estimating avg and std

[infer_isize] inferred external isize from 197764 pairs: 462.933 +/- 13.946

[infer_isize] skewness: 0.004; kurtosis: -0.118; ap_prior: 1.59e-05

[infer_isize] inferred maximum insert size: 560 (6.96 sigma)

[bwa_sai2sam_pe_core] time elapses: 29.05 sec

[bwa_sai2sam_pe_core] changing coordinates of 5825 alignments.

[bwa_sai2sam_pe_core] align unmapped mate...

[bwa_paired_sw] 16020 out of 16559 Q17 singletons are mated.

[bwa_paired_sw] 868 out of 1399 Q17 discordant pairs are fixed.

[bwa_sai2sam_pe_core] time elapses: 5.97 sec

[bwa_sai2sam_pe_core] refine gapped alignments... 1.04 sec

[bwa_sai2sam_pe_core] print alignments... 2.13 sec

[bwa_sai2sam_pe_core] 113508352 sequences have been processed.

[bwa_sai2sam_pe_core] convert to sequence coordinate...

[infer_isize] (25, 50, 75) percentile: (453, 463, 473)

[infer_isize] low and high boundaries: 413 and 513 for estimating avg and std

[infer_isize] inferred external isize from 197694 pairs: 462.937 +/- 13.971

[infer_isize] skewness: 0.012; kurtosis: -0.131; ap_prior: 1.67e-05

[infer_isize] inferred maximum insert size: 560 (6.96 sigma)

[bwa_sai2sam_pe_core] time elapses: 24.26 sec

[bwa_sai2sam_pe_core] changing coordinates of 5753 alignments.

[bwa_sai2sam_pe_core] align unmapped mate...

[bwa_paired_sw] 16488 out of 17060 Q17 singletons are mated.

[bwa_paired_sw] 866 out of 1394 Q17 discordant pairs are fixed.

[bwa_sai2sam_pe_core] time elapses: 6.10 sec

[bwa_sai2sam_pe_core] refine gapped alignments... 1.05 sec

[bwa_sai2sam_pe_core] print alignments... 2.09 sec

[bwa_sai2sam_pe_core] 113770496 sequences have been processed.

[bwa_sai2sam_pe_core] convert to sequence coordinate...

[infer_isize] (25, 50, 75) percentile: (453, 463, 473)

[infer_isize] low and high boundaries: 413 and 513 for estimating avg and std

[infer_isize] inferred external isize from 196590 pairs: 462.874 +/- 13.936

[infer_isize] skewness: 0.007; kurtosis: -0.122; ap_prior: 1.58e-05

[infer_isize] inferred maximum insert size: 560 (6.96 sigma)

[bwa_sai2sam_pe_core] time elapses: 24.12 sec

[bwa_sai2sam_pe_core] changing coordinates of 5746 alignments.

[bwa_sai2sam_pe_core] align unmapped mate...

[bwa_paired_sw] 17200 out of 17857 Q17 singletons are mated.

[bwa_paired_sw] 879 out of 1388 Q17 discordant pairs are fixed.

[bwa_sai2sam_pe_core] time elapses: 6.29 sec

[bwa_sai2sam_pe_core] refine gapped alignments... 1.07 sec

[bwa_sai2sam_pe_core] print alignments... 2.05 sec

[bwa_sai2sam_pe_core] 114032640 sequences have been processed.

[bwa_sai2sam_pe_core] convert to sequence coordinate...

[infer_isize] (25, 50, 75) percentile: (453, 463, 473)

[infer_isize] low and high boundaries: 413 and 513 for estimating avg and std

[infer_isize] inferred external isize from 196510 pairs: 462.897 +/- 13.917

[infer_isize] skewness: 0.012; kurtosis: -0.126; ap_prior: 1.68e-05

[infer_isize] inferred maximum insert size: 560 (6.96 sigma)

[bwa_sai2sam_pe_core] time elapses: 23.71 sec

[bwa_sai2sam_pe_core] changing coordinates of 5868 alignments.

[bwa_sai2sam_pe_core] align unmapped mate...

[bwa_paired_sw] 17256 out of 17825 Q17 singletons are mated.

[bwa_paired_sw] 881 out of 1397 Q17 discordant pairs are fixed.

[bwa_sai2sam_pe_core] time elapses: 6.30 sec

[bwa_sai2sam_pe_core] refine gapped alignments... 1.10 sec

[bwa_sai2sam_pe_core] print alignments... 2.07 sec

[bwa_sai2sam_pe_core] 114294784 sequences have been processed.

[bwa_sai2sam_pe_core] convert to sequence coordinate...

[infer_isize] (25, 50, 75) percentile: (453, 463, 473)

[infer_isize] low and high boundaries: 413 and 513 for estimating avg and std

[infer_isize] inferred external isize from 196506 pairs: 462.887 +/- 13.948

[infer_isize] skewness: 0.003; kurtosis: -0.129; ap_prior: 1.66e-05

[infer_isize] inferred maximum insert size: 560 (6.96 sigma)

[bwa_sai2sam_pe_core] time elapses: 24.10 sec

[bwa_sai2sam_pe_core] changing coordinates of 5814 alignments.

[bwa_sai2sam_pe_core] align unmapped mate...

[bwa_paired_sw] 17351 out of 17924 Q17 singletons are mated.

[bwa_paired_sw] 860 out of 1381 Q17 discordant pairs are fixed.

[bwa_sai2sam_pe_core] time elapses: 6.33 sec

[bwa_sai2sam_pe_core] refine gapped alignments... 1.06 sec

[bwa_sai2sam_pe_core] print alignments... 2.06 sec

[bwa_sai2sam_pe_core] 114556928 sequences have been processed.

[bwa_sai2sam_pe_core] convert to sequence coordinate...

[infer_isize] (25, 50, 75) percentile: (453, 463, 473)

[infer_isize] low and high boundaries: 413 and 513 for estimating avg and std

[infer_isize] inferred external isize from 195654 pairs: 462.942 +/- 13.911

[infer_isize] skewness: 0.006; kurtosis: -0.128; ap_prior: 1.68e-05

[infer_isize] inferred maximum insert size: 560 (6.96 sigma)

[bwa_sai2sam_pe_core] time elapses: 26.60 sec

[bwa_sai2sam_pe_core] changing coordinates of 5548 alignments.

[bwa_sai2sam_pe_core] align unmapped mate...

[bwa_paired_sw] 18188 out of 18787 Q17 singletons are mated.

[bwa_paired_sw] 895 out of 1407 Q17 discordant pairs are fixed.

[bwa_sai2sam_pe_core] time elapses: 6.57 sec

[bwa_sai2sam_pe_core] refine gapped alignments... 1.11 sec

[bwa_sai2sam_pe_core] print alignments... 2.07 sec

[bwa_sai2sam_pe_core] 114819072 sequences have been processed.

[bwa_sai2sam_pe_core] convert to sequence coordinate...

[infer_isize] (25, 50, 75) percentile: (453, 463, 472)

[infer_isize] low and high boundaries: 415 and 510 for estimating avg and std

[infer_isize] inferred external isize from 195170 pairs: 462.856 +/- 13.914

[infer_isize] skewness: 0.009; kurtosis: -0.161; ap_prior: 1.58e-05

[infer_isize] inferred maximum insert size: 560 (6.96 sigma)

[bwa_sai2sam_pe_core] time elapses: 28.62 sec

[bwa_sai2sam_pe_core] changing coordinates of 5662 alignments.

[bwa_sai2sam_pe_core] align unmapped mate...

[bwa_paired_sw] 18449 out of 19064 Q17 singletons are mated.

[bwa_paired_sw] 873 out of 1371 Q17 discordant pairs are fixed.

[bwa_sai2sam_pe_core] time elapses: 6.68 sec

[bwa_sai2sam_pe_core] refine gapped alignments... 1.16 sec

[bwa_sai2sam_pe_core] print alignments... 2.15 sec

[bwa_sai2sam_pe_core] 115081216 sequences have been processed.

[bwa_sai2sam_pe_core] convert to sequence coordinate...

[infer_isize] (25, 50, 75) percentile: (453, 463, 472)

[infer_isize] low and high boundaries: 415 and 510 for estimating avg and std

[infer_isize] inferred external isize from 193809 pairs: 462.828 +/- 13.867

[infer_isize] skewness: 0.014; kurtosis: -0.164; ap_prior: 1.63e-05

[infer_isize] inferred maximum insert size: 559 (6.96 sigma)

[bwa_sai2sam_pe_core] time elapses: 24.65 sec

[bwa_sai2sam_pe_core] changing coordinates of 5618 alignments.

[bwa_sai2sam_pe_core] align unmapped mate...

[bwa_paired_sw] 19449 out of 20046 Q17 singletons are mated.

[bwa_paired_sw] 825 out of 1384 Q17 discordant pairs are fixed.

[bwa_sai2sam_pe_core] time elapses: 6.96 sec

[bwa_sai2sam_pe_core] refine gapped alignments... 1.20 sec

[bwa_sai2sam_pe_core] print alignments... 2.13 sec

[bwa_sai2sam_pe_core] 115343360 sequences have been processed.

[bwa_sai2sam_pe_core] convert to sequence coordinate...

[infer_isize] (25, 50, 75) percentile: (453, 462, 472)

[infer_isize] low and high boundaries: 415 and 510 for estimating avg and std

[infer_isize] inferred external isize from 193723 pairs: 462.816 +/- 13.902

[infer_isize] skewness: 0.025; kurtosis: -0.167; ap_prior: 1.73e-05

[infer_isize] inferred maximum insert size: 560 (6.96 sigma)

[bwa_sai2sam_pe_core] time elapses: 24.98 sec

[bwa_sai2sam_pe_core] changing coordinates of 5549 alignments.

[bwa_sai2sam_pe_core] align unmapped mate...

[bwa_paired_sw] 19939 out of 20502 Q17 singletons are mated.

[bwa_paired_sw] 890 out of 1413 Q17 discordant pairs are fixed.

[bwa_sai2sam_pe_core] time elapses: 7.13 sec

[bwa_sai2sam_pe_core] refine gapped alignments... 1.20 sec

[bwa_sai2sam_pe_core] print alignments... 2.12 sec

[bwa_sai2sam_pe_core] 115605504 sequences have been processed.

[bwa_sai2sam_pe_core] convert to sequence coordinate...

[infer_isize] (25, 50, 75) percentile: (453, 463, 472)

[infer_isize] low and high boundaries: 415 and 510 for estimating avg and std

[infer_isize] inferred external isize from 192248 pairs: 462.805 +/- 13.873

[infer_isize] skewness: 0.013; kurtosis: -0.171; ap_prior: 1.65e-05

[infer_isize] inferred maximum insert size: 559 (6.96 sigma)

[bwa_sai2sam_pe_core] time elapses: 22.84 sec

[bwa_sai2sam_pe_core] changing coordinates of 5620 alignments.

[bwa_sai2sam_pe_core] align unmapped mate...

[bwa_paired_sw] 20933 out of 21565 Q17 singletons are mated.

[bwa_paired_sw] 908 out of 1442 Q17 discordant pairs are fixed.

[bwa_sai2sam_pe_core] time elapses: 7.38 sec

[bwa_sai2sam_pe_core] refine gapped alignments... 1.22 sec

[bwa_sai2sam_pe_core] print alignments... 2.14 sec

[bwa_sai2sam_pe_core] 115867648 sequences have been processed.

[bwa_sai2sam_pe_core] convert to sequence coordinate...

[infer_isize] (25, 50, 75) percentile: (453, 462, 472)

[infer_isize] low and high boundaries: 415 and 510 for estimating avg and std

[infer_isize] inferred external isize from 192236 pairs: 462.772 +/- 13.877

[infer_isize] skewness: 0.016; kurtosis: -0.164; ap_prior: 1.72e-05

[infer_isize] inferred maximum insert size: 559 (6.96 sigma)

[bwa_sai2sam_pe_core] time elapses: 23.10 sec

[bwa_sai2sam_pe_core] changing coordinates of 5614 alignments.

[bwa_sai2sam_pe_core] align unmapped mate...

[bwa_paired_sw] 20695 out of 21356 Q17 singletons are mated.

[bwa_paired_sw] 876 out of 1379 Q17 discordant pairs are fixed.

[bwa_sai2sam_pe_core] time elapses: 7.30 sec

[bwa_sai2sam_pe_core] refine gapped alignments... 1.22 sec

[bwa_sai2sam_pe_core] print alignments... 2.11 sec

[bwa_sai2sam_pe_core] 116129792 sequences have been processed.

[bwa_sai2sam_pe_core] convert to sequence coordinate...

[infer_isize] (25, 50, 75) percentile: (453, 463, 473)

[infer_isize] low and high boundaries: 413 and 513 for estimating avg and std

[infer_isize] inferred external isize from 191520 pairs: 462.846 +/- 13.954

[infer_isize] skewness: 0.005; kurtosis: -0.119; ap_prior: 1.70e-05

[infer_isize] inferred maximum insert size: 560 (6.96 sigma)

[bwa_sai2sam_pe_core] time elapses: 23.16 sec

[bwa_sai2sam_pe_core] changing coordinates of 5676 alignments.

[bwa_sai2sam_pe_core] align unmapped mate...

[bwa_paired_sw] 21747 out of 22349 Q17 singletons are mated.

[bwa_paired_sw] 884 out of 1416 Q17 discordant pairs are fixed.

[bwa_sai2sam_pe_core] time elapses: 7.58 sec

[bwa_sai2sam_pe_core] refine gapped alignments... 1.26 sec

[bwa_sai2sam_pe_core] print alignments... 2.11 sec

[bwa_sai2sam_pe_core] 116391936 sequences have been processed.

[bwa_sai2sam_pe_core] convert to sequence coordinate...

[infer_isize] (25, 50, 75) percentile: (453, 463, 473)

[infer_isize] low and high boundaries: 413 and 513 for estimating avg and std

[infer_isize] inferred external isize from 197619 pairs: 463.009 +/- 13.924

[infer_isize] skewness: 0.002; kurtosis: -0.144; ap_prior: 1.70e-05

[infer_isize] inferred maximum insert size: 560 (6.96 sigma)

[bwa_sai2sam_pe_core] time elapses: 23.58 sec

[bwa_sai2sam_pe_core] changing coordinates of 5755 alignments.

[bwa_sai2sam_pe_core] align unmapped mate...

[bwa_paired_sw] 16707 out of 17296 Q17 singletons are mated.

[bwa_paired_sw] 890 out of 1418 Q17 discordant pairs are fixed.

[bwa_sai2sam_pe_core] time elapses: 6.17 sec

[bwa_sai2sam_pe_core] refine gapped alignments... 1.09 sec

[bwa_sai2sam_pe_core] print alignments... 2.12 sec

[bwa_sai2sam_pe_core] 116654080 sequences have been processed.

[bwa_sai2sam_pe_core] convert to sequence coordinate...

[infer_isize] (25, 50, 75) percentile: (453, 463, 473)

[infer_isize] low and high boundaries: 413 and 513 for estimating avg and std

[infer_isize] inferred external isize from 204338 pairs: 463.253 +/- 13.922

[infer_isize] skewness: 0.006; kurtosis: -0.119; ap_prior: 1.83e-05

[infer_isize] inferred maximum insert size: 560 (6.96 sigma)

[bwa_sai2sam_pe_core] time elapses: 24.06 sec

[bwa_sai2sam_pe_core] changing coordinates of 5989 alignments.

[bwa_sai2sam_pe_core] align unmapped mate...

[bwa_paired_sw] 11291 out of 11858 Q17 singletons are mated.

[bwa_paired_sw] 885 out of 1441 Q17 discordant pairs are fixed.

[bwa_sai2sam_pe_core] time elapses: 4.72 sec

[bwa_sai2sam_pe_core] refine gapped alignments... 0.96 sec

[bwa_sai2sam_pe_core] print alignments... 2.08 sec

[bwa_sai2sam_pe_core] 116916224 sequences have been processed.

[bwa_sai2sam_pe_core] convert to sequence coordinate...

[infer_isize] (25, 50, 75) percentile: (453, 463, 473)

[infer_isize] low and high boundaries: 413 and 513 for estimating avg and std

[infer_isize] inferred external isize from 204101 pairs: 463.326 +/- 13.959

[infer_isize] skewness: 0.003; kurtosis: -0.139; ap_prior: 1.71e-05

[infer_isize] inferred maximum insert size: 560 (6.96 sigma)

[bwa_sai2sam_pe_core] time elapses: 29.60 sec

[bwa_sai2sam_pe_core] changing coordinates of 6097 alignments.

[bwa_sai2sam_pe_core] align unmapped mate...

[bwa_paired_sw] 11419 out of 11960 Q17 singletons are mated.

[bwa_paired_sw] 870 out of 1461 Q17 discordant pairs are fixed.

[bwa_sai2sam_pe_core] time elapses: 4.76 sec

[bwa_sai2sam_pe_core] refine gapped alignments... 0.93 sec

[bwa_sai2sam_pe_core] print alignments... 2.09 sec

[bwa_sai2sam_pe_core] 117178368 sequences have been processed.

[bwa_sai2sam_pe_core] convert to sequence coordinate...

[infer_isize] (25, 50, 75) percentile: (453, 463, 473)

[infer_isize] low and high boundaries: 413 and 513 for estimating avg and std

[infer_isize] inferred external isize from 204789 pairs: 463.297 +/- 13.955

[infer_isize] skewness: 0.006; kurtosis: -0.142; ap_prior: 1.81e-05

[infer_isize] inferred maximum insert size: 560 (6.96 sigma)

[bwa_sai2sam_pe_core] time elapses: 29.07 sec

[bwa_sai2sam_pe_core] changing coordinates of 5805 alignments.

[bwa_sai2sam_pe_core] align unmapped mate...

[bwa_paired_sw] 10924 out of 11517 Q17 singletons are mated.

[bwa_paired_sw] 895 out of 1442 Q17 discordant pairs are fixed.

[bwa_sai2sam_pe_core] time elapses: 4.60 sec

[bwa_sai2sam_pe_core] refine gapped alignments... 0.92 sec

[bwa_sai2sam_pe_core] print alignments... 2.09 sec

[bwa_sai2sam_pe_core] 117440512 sequences have been processed.

[bwa_sai2sam_pe_core] convert to sequence coordinate...

[infer_isize] (25, 50, 75) percentile: (453, 463, 473)

[infer_isize] low and high boundaries: 413 and 513 for estimating avg and std

[infer_isize] inferred external isize from 204834 pairs: 463.309 +/- 13.930

[infer_isize] skewness: -0.001; kurtosis: -0.135; ap_prior: 1.79e-05

[infer_isize] inferred maximum insert size: 560 (6.96 sigma)

[bwa_sai2sam_pe_core] time elapses: 24.14 sec

[bwa_sai2sam_pe_core] changing coordinates of 5990 alignments.

[bwa_sai2sam_pe_core] align unmapped mate...

[bwa_paired_sw] 11078 out of 11618 Q17 singletons are mated.

[bwa_paired_sw] 833 out of 1408 Q17 discordant pairs are fixed.

[bwa_sai2sam_pe_core] time elapses: 4.64 sec

[bwa_sai2sam_pe_core] refine gapped alignments... 0.92 sec

[bwa_sai2sam_pe_core] print alignments... 2.06 sec

[bwa_sai2sam_pe_core] 117702656 sequences have been processed.

[bwa_sai2sam_pe_core] convert to sequence coordinate...

[infer_isize] (25, 50, 75) percentile: (453, 463, 473)

[infer_isize] low and high boundaries: 413 and 513 for estimating avg and std

[infer_isize] inferred external isize from 205086 pairs: 463.298 +/- 13.927

[infer_isize] skewness: -0.000; kurtosis: -0.142; ap_prior: 1.60e-05

[infer_isize] inferred maximum insert size: 560 (6.96 sigma)

[bwa_sai2sam_pe_core] time elapses: 27.40 sec

[bwa_sai2sam_pe_core] changing coordinates of 5960 alignments.

[bwa_sai2sam_pe_core] align unmapped mate...

[bwa_paired_sw] 10895 out of 11453 Q17 singletons are mated.

[bwa_paired_sw] 872 out of 1426 Q17 discordant pairs are fixed.

[bwa_sai2sam_pe_core] time elapses: 4.61 sec

[bwa_sai2sam_pe_core] refine gapped alignments... 0.93 sec

[bwa_sai2sam_pe_core] print alignments... 2.07 sec

[bwa_sai2sam_pe_core] 117964800 sequences have been processed.

[bwa_sai2sam_pe_core] convert to sequence coordinate...

[infer_isize] (25, 50, 75) percentile: (453, 463, 473)

[infer_isize] low and high boundaries: 413 and 513 for estimating avg and std

[infer_isize] inferred external isize from 204526 pairs: 463.285 +/- 13.929

[infer_isize] skewness: 0.000; kurtosis: -0.123; ap_prior: 1.86e-05

[infer_isize] inferred maximum insert size: 560 (6.96 sigma)

[bwa_sai2sam_pe_core] time elapses: 27.58 sec

[bwa_sai2sam_pe_core] changing coordinates of 5907 alignments.

[bwa_sai2sam_pe_core] align unmapped mate...

[bwa_paired_sw] 11337 out of 11828 Q17 singletons are mated.

[bwa_paired_sw] 869 out of 1420 Q17 discordant pairs are fixed.

[bwa_sai2sam_pe_core] time elapses: 4.71 sec

[bwa_sai2sam_pe_core] refine gapped alignments... 0.92 sec

[bwa_sai2sam_pe_core] print alignments... 2.10 sec

[bwa_sai2sam_pe_core] 118226944 sequences have been processed.

[bwa_sai2sam_pe_core] convert to sequence coordinate...

[infer_isize] (25, 50, 75) percentile: (453, 463, 473)

[infer_isize] low and high boundaries: 413 and 513 for estimating avg and std

[infer_isize] inferred external isize from 204103 pairs: 463.348 +/- 13.948

[infer_isize] skewness: 0.002; kurtosis: -0.136; ap_prior: 1.84e-05

[infer_isize] inferred maximum insert size: 560 (6.96 sigma)

[bwa_sai2sam_pe_core] time elapses: 28.53 sec

[bwa_sai2sam_pe_core] changing coordinates of 6158 alignments.

[bwa_sai2sam_pe_core] align unmapped mate...

[bwa_paired_sw] 11324 out of 11860 Q17 singletons are mated.

[bwa_paired_sw] 937 out of 1501 Q17 discordant pairs are fixed.

[bwa_sai2sam_pe_core] time elapses: 4.75 sec

[bwa_sai2sam_pe_core] refine gapped alignments... 0.95 sec

[bwa_sai2sam_pe_core] print alignments... 2.12 sec

[bwa_sai2sam_pe_core] 118489088 sequences have been processed.

[bwa_sai2sam_pe_core] convert to sequence coordinate...

[infer_isize] (25, 50, 75) percentile: (453, 463, 473)

[infer_isize] low and high boundaries: 413 and 513 for estimating avg and std

[infer_isize] inferred external isize from 204274 pairs: 463.285 +/- 13.951

[infer_isize] skewness: -0.001; kurtosis: -0.135; ap_prior: 1.59e-05

[infer_isize] inferred maximum insert size: 560 (6.96 sigma)

[bwa_sai2sam_pe_core] time elapses: 28.68 sec

[bwa_sai2sam_pe_core] changing coordinates of 5840 alignments.

[bwa_sai2sam_pe_core] align unmapped mate...

[bwa_paired_sw] 11201 out of 11728 Q17 singletons are mated.

[bwa_paired_sw] 894 out of 1476 Q17 discordant pairs are fixed.

[bwa_sai2sam_pe_core] time elapses: 4.72 sec

[bwa_sai2sam_pe_core] refine gapped alignments... 0.96 sec

[bwa_sai2sam_pe_core] print alignments... 2.14 sec

[bwa_sai2sam_pe_core] 118751232 sequences have been processed.

[bwa_sai2sam_pe_core] convert to sequence coordinate...

[infer_isize] (25, 50, 75) percentile: (453, 463, 473)

[infer_isize] low and high boundaries: 413 and 513 for estimating avg and std

[infer_isize] inferred external isize from 204549 pairs: 463.279 +/- 13.933

[infer_isize] skewness: -0.005; kurtosis: -0.134; ap_prior: 1.64e-05

[infer_isize] inferred maximum insert size: 560 (6.96 sigma)

[bwa_sai2sam_pe_core] time elapses: 28.90 sec

[bwa_sai2sam_pe_core] changing coordinates of 6069 alignments.

[bwa_sai2sam_pe_core] align unmapped mate...

[bwa_paired_sw] 11168 out of 11716 Q17 singletons are mated.

[bwa_paired_sw] 889 out of 1466 Q17 discordant pairs are fixed.

[bwa_sai2sam_pe_core] time elapses: 4.73 sec

[bwa_sai2sam_pe_core] refine gapped alignments... 0.97 sec

[bwa_sai2sam_pe_core] print alignments... 2.20 sec

[bwa_sai2sam_pe_core] 119013376 sequences have been processed.

[bwa_sai2sam_pe_core] convert to sequence coordinate...

[infer_isize] (25, 50, 75) percentile: (453, 463, 473)

[infer_isize] low and high boundaries: 413 and 513 for estimating avg and std

[infer_isize] inferred external isize from 204652 pairs: 463.283 +/- 13.975

[infer_isize] skewness: 0.000; kurtosis: -0.139; ap_prior: 1.79e-05

[infer_isize] inferred maximum insert size: 561 (6.96 sigma)

[bwa_sai2sam_pe_core] time elapses: 31.65 sec

[bwa_sai2sam_pe_core] changing coordinates of 5888 alignments.

[bwa_sai2sam_pe_core] align unmapped mate...

[bwa_paired_sw] 11186 out of 11733 Q17 singletons are mated.

[bwa_paired_sw] 906 out of 1466 Q17 discordant pairs are fixed.

[bwa_sai2sam_pe_core] time elapses: 4.93 sec

[bwa_sai2sam_pe_core] refine gapped alignments... 1.01 sec

[bwa_sai2sam_pe_core] print alignments... 2.20 sec

[bwa_sai2sam_pe_core] 119275520 sequences have been processed.

[bwa_sai2sam_pe_core] convert to sequence coordinate...

[infer_isize] (25, 50, 75) percentile: (453, 463, 473)

[infer_isize] low and high boundaries: 413 and 513 for estimating avg and std

[infer_isize] inferred external isize from 204487 pairs: 463.305 +/- 13.946

[infer_isize] skewness: 0.008; kurtosis: -0.137; ap_prior: 1.62e-05

[infer_isize] inferred maximum insert size: 560 (6.96 sigma)

[bwa_sai2sam_pe_core] time elapses: 31.90 sec

[bwa_sai2sam_pe_core] changing coordinates of 5966 alignments.

[bwa_sai2sam_pe_core] align unmapped mate...

[bwa_paired_sw] 11548 out of 12088 Q17 singletons are mated.

[bwa_paired_sw] 839 out of 1372 Q17 discordant pairs are fixed.

[bwa_sai2sam_pe_core] time elapses: 4.96 sec

[bwa_sai2sam_pe_core] refine gapped alignments... 1.00 sec

[bwa_sai2sam_pe_core] print alignments... 2.17 sec

[bwa_sai2sam_pe_core] 119537664 sequences have been processed.

[bwa_sai2sam_pe_core] convert to sequence coordinate...

[infer_isize] (25, 50, 75) percentile: (453, 463, 473)

[infer_isize] low and high boundaries: 413 and 513 for estimating avg and std

[infer_isize] inferred external isize from 203574 pairs: 463.314 +/- 13.924

[infer_isize] skewness: 0.004; kurtosis: -0.140; ap_prior: 1.86e-05

[infer_isize] inferred maximum insert size: 560 (6.96 sigma)

[bwa_sai2sam_pe_core] time elapses: 32.13 sec

[bwa_sai2sam_pe_core] changing coordinates of 5898 alignments.

[bwa_sai2sam_pe_core] align unmapped mate...

[bwa_paired_sw] 11852 out of 12389 Q17 singletons are mated.

[bwa_paired_sw] 909 out of 1495 Q17 discordant pairs are fixed.

[bwa_sai2sam_pe_core] time elapses: 5.03 sec

[bwa_sai2sam_pe_core] refine gapped alignments... 0.98 sec

[bwa_sai2sam_pe_core] print alignments... 2.16 sec

[bwa_sai2sam_pe_core] 119799808 sequences have been processed.

[bwa_sai2sam_pe_core] convert to sequence coordinate...

[infer_isize] (25, 50, 75) percentile: (453, 463, 473)

[infer_isize] low and high boundaries: 413 and 513 for estimating avg and std

[infer_isize] inferred external isize from 204796 pairs: 463.297 +/- 13.939

[infer_isize] skewness: -0.002; kurtosis: -0.130; ap_prior: 1.79e-05

[infer_isize] inferred maximum insert size: 560 (6.96 sigma)

[bwa_sai2sam_pe_core] time elapses: 31.08 sec

[bwa_sai2sam_pe_core] changing coordinates of 6014 alignments.

[bwa_sai2sam_pe_core] align unmapped mate...

[bwa_paired_sw] 11002 out of 11553 Q17 singletons are mated.

[bwa_paired_sw] 915 out of 1527 Q17 discordant pairs are fixed.

[bwa_sai2sam_pe_core] time elapses: 4.66 sec

[bwa_sai2sam_pe_core] refine gapped alignments... 0.96 sec

[bwa_sai2sam_pe_core] print alignments... 2.12 sec

[bwa_sai2sam_pe_core] 120061952 sequences have been processed.

[bwa_sai2sam_pe_core] convert to sequence coordinate...

[infer_isize] (25, 50, 75) percentile: (453, 463, 473)

[infer_isize] low and high boundaries: 413 and 513 for estimating avg and std

[infer_isize] inferred external isize from 204244 pairs: 463.293 +/- 13.939

[infer_isize] skewness: -0.002; kurtosis: -0.140; ap_prior: 1.70e-05

[infer_isize] inferred maximum insert size: 560 (6.96 sigma)

[bwa_sai2sam_pe_core] time elapses: 25.39 sec

[bwa_sai2sam_pe_core] changing coordinates of 6019 alignments.

[bwa_sai2sam_pe_core] align unmapped mate...

[bwa_paired_sw] 11477 out of 12035 Q17 singletons are mated.

[bwa_paired_sw] 866 out of 1450 Q17 discordant pairs are fixed.

[bwa_sai2sam_pe_core] time elapses: 4.77 sec

[bwa_sai2sam_pe_core] refine gapped alignments... 0.92 sec

[bwa_sai2sam_pe_core] print alignments... 2.07 sec

[bwa_sai2sam_pe_core] 120324096 sequences have been processed.

[bwa_sai2sam_pe_core] convert to sequence coordinate...

[infer_isize] (25, 50, 75) percentile: (453, 463, 473)

[infer_isize] low and high boundaries: 413 and 513 for estimating avg and std

[infer_isize] inferred external isize from 204185 pairs: 463.255 +/- 13.943

[infer_isize] skewness: 0.008; kurtosis: -0.153; ap_prior: 1.71e-05

[infer_isize] inferred maximum insert size: 560 (6.96 sigma)

[bwa_sai2sam_pe_core] time elapses: 28.46 sec

[bwa_sai2sam_pe_core] changing coordinates of 6075 alignments.

[bwa_sai2sam_pe_core] align unmapped mate...

[bwa_paired_sw] 11486 out of 12036 Q17 singletons are mated.

[bwa_paired_sw] 901 out of 1510 Q17 discordant pairs are fixed.

[bwa_sai2sam_pe_core] time elapses: 4.80 sec

[bwa_sai2sam_pe_core] refine gapped alignments... 0.95 sec

[bwa_sai2sam_pe_core] print alignments... 2.05 sec

[bwa_sai2sam_pe_core] 120586240 sequences have been processed.

[bwa_sai2sam_pe_core] convert to sequence coordinate...

[infer_isize] (25, 50, 75) percentile: (453, 463, 473)

[infer_isize] low and high boundaries: 413 and 513 for estimating avg and std

[infer_isize] inferred external isize from 204459 pairs: 463.293 +/- 13.944

[infer_isize] skewness: 0.010; kurtosis: -0.141; ap_prior: 1.78e-05

[infer_isize] inferred maximum insert size: 560 (6.96 sigma)

[bwa_sai2sam_pe_core] time elapses: 28.74 sec

[bwa_sai2sam_pe_core] changing coordinates of 6011 alignments.

[bwa_sai2sam_pe_core] align unmapped mate...

[bwa_paired_sw] 11201 out of 11766 Q17 singletons are mated.

[bwa_paired_sw] 856 out of 1468 Q17 discordant pairs are fixed.

[bwa_sai2sam_pe_core] time elapses: 4.70 sec

[bwa_sai2sam_pe_core] refine gapped alignments... 0.92 sec

[bwa_sai2sam_pe_core] print alignments... 2.08 sec

[bwa_sai2sam_pe_core] 120848384 sequences have been processed.

[bwa_sai2sam_pe_core] convert to sequence coordinate...

[infer_isize] (25, 50, 75) percentile: (453, 463, 473)

[infer_isize] low and high boundaries: 413 and 513 for estimating avg and std

[infer_isize] inferred external isize from 203993 pairs: 463.273 +/- 13.930

[infer_isize] skewness: 0.001; kurtosis: -0.123; ap_prior: 1.74e-05

[infer_isize] inferred maximum insert size: 560 (6.96 sigma)

[bwa_sai2sam_pe_core] time elapses: 28.64 sec

[bwa_sai2sam_pe_core] changing coordinates of 6115 alignments.

[bwa_sai2sam_pe_core] align unmapped mate...

[bwa_paired_sw] 11425 out of 11927 Q17 singletons are mated.

[bwa_paired_sw] 894 out of 1500 Q17 discordant pairs are fixed.

[bwa_sai2sam_pe_core] time elapses: 4.77 sec

[bwa_sai2sam_pe_core] refine gapped alignments... 0.94 sec

[bwa_sai2sam_pe_core] print alignments... 2.06 sec

[bwa_sai2sam_pe_core] 121110528 sequences have been processed.

[bwa_sai2sam_pe_core] convert to sequence coordinate...

[infer_isize] (25, 50, 75) percentile: (453, 463, 473)

[infer_isize] low and high boundaries: 413 and 513 for estimating avg and std

[infer_isize] inferred external isize from 204122 pairs: 463.244 +/- 13.905

[infer_isize] skewness: 0.006; kurtosis: -0.133; ap_prior: 1.79e-05

[infer_isize] inferred maximum insert size: 560 (6.96 sigma)

[bwa_sai2sam_pe_core] time elapses: 29.16 sec

[bwa_sai2sam_pe_core] changing coordinates of 6024 alignments.

[bwa_sai2sam_pe_core] align unmapped mate...

[bwa_paired_sw] 11158 out of 11710 Q17 singletons are mated.

[bwa_paired_sw] 864 out of 1440 Q17 discordant pairs are fixed.

[bwa_sai2sam_pe_core] time elapses: 4.70 sec

[bwa_sai2sam_pe_core] refine gapped alignments... 0.95 sec

[bwa_sai2sam_pe_core] print alignments... 2.07 sec

[bwa_sai2sam_pe_core] 121372672 sequences have been processed.

[bwa_sai2sam_pe_core] convert to sequence coordinate...

[infer_isize] (25, 50, 75) percentile: (453, 463, 473)

[infer_isize] low and high boundaries: 413 and 513 for estimating avg and std

[infer_isize] inferred external isize from 203915 pairs: 463.251 +/- 13.938

[infer_isize] skewness: 0.002; kurtosis: -0.137; ap_prior: 1.72e-05

[infer_isize] inferred maximum insert size: 560 (6.96 sigma)

[bwa_sai2sam_pe_core] time elapses: 28.82 sec

[bwa_sai2sam_pe_core] changing coordinates of 5920 alignments.

[bwa_sai2sam_pe_core] align unmapped mate...

[bwa_paired_sw] 11321 out of 11925 Q17 singletons are mated.

[bwa_paired_sw] 888 out of 1464 Q17 discordant pairs are fixed.

[bwa_sai2sam_pe_core] time elapses: 4.74 sec

[bwa_sai2sam_pe_core] refine gapped alignments... 0.92 sec

[bwa_sai2sam_pe_core] print alignments... 2.13 sec

[bwa_sai2sam_pe_core] 121634816 sequences have been processed.

[bwa_sai2sam_pe_core] convert to sequence coordinate...

[infer_isize] (25, 50, 75) percentile: (453, 463, 473)

[infer_isize] low and high boundaries: 413 and 513 for estimating avg and std

[infer_isize] inferred external isize from 203747 pairs: 463.217 +/- 13.906

[infer_isize] skewness: 0.001; kurtosis: -0.132; ap_prior: 1.66e-05

[infer_isize] inferred maximum insert size: 560 (6.96 sigma)

[bwa_sai2sam_pe_core] time elapses: 29.07 sec

[bwa_sai2sam_pe_core] changing coordinates of 6057 alignments.

[bwa_sai2sam_pe_core] align unmapped mate...

[bwa_paired_sw] 11666 out of 12246 Q17 singletons are mated.

[bwa_paired_sw] 883 out of 1453 Q17 discordant pairs are fixed.

[bwa_sai2sam_pe_core] time elapses: 4.82 sec

[bwa_sai2sam_pe_core] refine gapped alignments... 0.98 sec

[bwa_sai2sam_pe_core] print alignments... 2.16 sec

[bwa_sai2sam_pe_core] 121896960 sequences have been processed.

[bwa_sai2sam_pe_core] convert to sequence coordinate...

[infer_isize] (25, 50, 75) percentile: (453, 463, 473)

[infer_isize] low and high boundaries: 413 and 513 for estimating avg and std

[infer_isize] inferred external isize from 204074 pairs: 463.256 +/- 13.935

[infer_isize] skewness: 0.005; kurtosis: -0.137; ap_prior: 1.72e-05

[infer_isize] inferred maximum insert size: 560 (6.96 sigma)

[bwa_sai2sam_pe_core] time elapses: 29.02 sec

[bwa_sai2sam_pe_core] changing coordinates of 5976 alignments.

[bwa_sai2sam_pe_core] align unmapped mate...

[bwa_paired_sw] 11664 out of 12229 Q17 singletons are mated.

[bwa_paired_sw] 907 out of 1478 Q17 discordant pairs are fixed.

[bwa_sai2sam_pe_core] time elapses: 4.99 sec

[bwa_sai2sam_pe_core] refine gapped alignments... 1.00 sec

[bwa_sai2sam_pe_core] print alignments... 2.21 sec

[bwa_sai2sam_pe_core] 122159104 sequences have been processed.

[bwa_sai2sam_pe_core] convert to sequence coordinate...

[infer_isize] (25, 50, 75) percentile: (453, 463, 473)

[infer_isize] low and high boundaries: 413 and 513 for estimating avg and std

[infer_isize] inferred external isize from 203952 pairs: 463.284 +/- 13.962

[infer_isize] skewness: 0.001; kurtosis: -0.143; ap_prior: 1.82e-05

[infer_isize] inferred maximum insert size: 560 (6.96 sigma)

[bwa_sai2sam_pe_core] time elapses: 29.77 sec

[bwa_sai2sam_pe_core] changing coordinates of 6171 alignments.

[bwa_sai2sam_pe_core] align unmapped mate...

[bwa_paired_sw] 11157 out of 11717 Q17 singletons are mated.

[bwa_paired_sw] 887 out of 1479 Q17 discordant pairs are fixed.

[bwa_sai2sam_pe_core] time elapses: 4.73 sec

[bwa_sai2sam_pe_core] refine gapped alignments... 0.96 sec

[bwa_sai2sam_pe_core] print alignments... 2.19 sec

[bwa_sai2sam_pe_core] 122421248 sequences have been processed.

[bwa_sai2sam_pe_core] convert to sequence coordinate...

[infer_isize] (25, 50, 75) percentile: (453, 463, 473)

[infer_isize] low and high boundaries: 413 and 513 for estimating avg and std

[infer_isize] inferred external isize from 204655 pairs: 463.248 +/- 13.916

[infer_isize] skewness: 0.002; kurtosis: -0.136; ap_prior: 1.56e-05

[infer_isize] inferred maximum insert size: 560 (6.96 sigma)

[bwa_sai2sam_pe_core] time elapses: 32.12 sec

[bwa_sai2sam_pe_core] changing coordinates of 5910 alignments.

[bwa_sai2sam_pe_core] align unmapped mate...

[bwa_paired_sw] 11135 out of 11690 Q17 singletons are mated.

[bwa_paired_sw] 838 out of 1423 Q17 discordant pairs are fixed.

[bwa_sai2sam_pe_core] time elapses: 4.92 sec

[bwa_sai2sam_pe_core] refine gapped alignments... 1.02 sec

[bwa_sai2sam_pe_core] print alignments... 2.23 sec

[bwa_sai2sam_pe_core] 122683392 sequences have been processed.

[bwa_sai2sam_pe_core] convert to sequence coordinate...

[infer_isize] (25, 50, 75) percentile: (453, 463, 473)

[infer_isize] low and high boundaries: 413 and 513 for estimating avg and std

[infer_isize] inferred external isize from 204137 pairs: 463.257 +/- 13.924

[infer_isize] skewness: 0.002; kurtosis: -0.140; ap_prior: 1.81e-05

[infer_isize] inferred maximum insert size: 560 (6.96 sigma)

[bwa_sai2sam_pe_core] time elapses: 27.64 sec

[bwa_sai2sam_pe_core] changing coordinates of 6051 alignments.

[bwa_sai2sam_pe_core] align unmapped mate...

[bwa_paired_sw] 11286 out of 11804 Q17 singletons are mated.

[bwa_paired_sw] 861 out of 1422 Q17 discordant pairs are fixed.

[bwa_sai2sam_pe_core] time elapses: 4.71 sec

[bwa_sai2sam_pe_core] refine gapped alignments... 1.01 sec

[bwa_sai2sam_pe_core] print alignments... 2.23 sec

[bwa_sai2sam_pe_core] 122945536 sequences have been processed.

[bwa_sai2sam_pe_core] convert to sequence coordinate...

[infer_isize] (25, 50, 75) percentile: (453, 463, 473)

[infer_isize] low and high boundaries: 413 and 513 for estimating avg and std

[infer_isize] inferred external isize from 204308 pairs: 463.273 +/- 13.955

[infer_isize] skewness: -0.006; kurtosis: -0.129; ap_prior: 1.72e-05

[infer_isize] inferred maximum insert size: 560 (6.96 sigma)

[bwa_sai2sam_pe_core] time elapses: 27.40 sec

[bwa_sai2sam_pe_core] changing coordinates of 6014 alignments.

[bwa_sai2sam_pe_core] align unmapped mate...

[bwa_paired_sw] 11231 out of 11774 Q17 singletons are mated.

[bwa_paired_sw] 915 out of 1485 Q17 discordant pairs are fixed.

[bwa_sai2sam_pe_core] time elapses: 4.73 sec

[bwa_sai2sam_pe_core] refine gapped alignments... 1.01 sec

[bwa_sai2sam_pe_core] print alignments... 2.17 sec

[bwa_sai2sam_pe_core] 123207680 sequences have been processed.

[bwa_sai2sam_pe_core] convert to sequence coordinate...

[infer_isize] (25, 50, 75) percentile: (453, 463, 473)

[infer_isize] low and high boundaries: 413 and 513 for estimating avg and std

[infer_isize] inferred external isize from 204475 pairs: 463.272 +/- 13.960

[infer_isize] skewness: 0.004; kurtosis: -0.161; ap_prior: 1.61e-05

[infer_isize] inferred maximum insert size: 560 (6.96 sigma)

[bwa_sai2sam_pe_core] time elapses: 28.16 sec

[bwa_sai2sam_pe_core] changing coordinates of 6064 alignments.

[bwa_sai2sam_pe_core] align unmapped mate...

[bwa_paired_sw] 11290 out of 11843 Q17 singletons are mated.

[bwa_paired_sw] 847 out of 1424 Q17 discordant pairs are fixed.

[bwa_sai2sam_pe_core] time elapses: 4.71 sec

[bwa_sai2sam_pe_core] refine gapped alignments... 0.97 sec

[bwa_sai2sam_pe_core] print alignments... 2.10 sec

[bwa_sai2sam_pe_core] 123469824 sequences have been processed.

[bwa_sai2sam_pe_core] convert to sequence coordinate...

[infer_isize] (25, 50, 75) percentile: (453, 463, 473)

[infer_isize] low and high boundaries: 413 and 513 for estimating avg and std

[infer_isize] inferred external isize from 204462 pairs: 463.297 +/- 13.924

[infer_isize] skewness: -0.001; kurtosis: -0.123; ap_prior: 1.63e-05

[infer_isize] inferred maximum insert size: 560 (6.96 sigma)

[bwa_sai2sam_pe_core] time elapses: 27.50 sec

[bwa_sai2sam_pe_core] changing coordinates of 5919 alignments.

[bwa_sai2sam_pe_core] align unmapped mate...

[bwa_paired_sw] 11632 out of 12132 Q17 singletons are mated.

[bwa_paired_sw] 869 out of 1431 Q17 discordant pairs are fixed.

[bwa_sai2sam_pe_core] time elapses: 4.80 sec

[bwa_sai2sam_pe_core] refine gapped alignments... 0.97 sec

[bwa_sai2sam_pe_core] print alignments... 2.08 sec

[bwa_sai2sam_pe_core] 123731968 sequences have been processed.

[bwa_sai2sam_pe_core] convert to sequence coordinate...

[infer_isize] (25, 50, 75) percentile: (453, 463, 473)

[infer_isize] low and high boundaries: 413 and 513 for estimating avg and std

[infer_isize] inferred external isize from 203998 pairs: 463.226 +/- 13.950

[infer_isize] skewness: 0.005; kurtosis: -0.160; ap_prior: 1.69e-05

[infer_isize] inferred maximum insert size: 560 (6.96 sigma)

[bwa_sai2sam_pe_core] time elapses: 27.58 sec

[bwa_sai2sam_pe_core] changing coordinates of 6034 alignments.

[bwa_sai2sam_pe_core] align unmapped mate...

[bwa_paired_sw] 11646 out of 12174 Q17 singletons are mated.

[bwa_paired_sw] 901 out of 1453 Q17 discordant pairs are fixed.

[bwa_sai2sam_pe_core] time elapses: 4.81 sec

[bwa_sai2sam_pe_core] refine gapped alignments... 0.95 sec

[bwa_sai2sam_pe_core] print alignments... 2.06 sec

[bwa_sai2sam_pe_core] 123994112 sequences have been processed.

[bwa_sai2sam_pe_core] convert to sequence coordinate...

[infer_isize] (25, 50, 75) percentile: (453, 463, 473)

[infer_isize] low and high boundaries: 413 and 513 for estimating avg and std

[infer_isize] inferred external isize from 203893 pairs: 463.194 +/- 13.957

[infer_isize] skewness: 0.006; kurtosis: -0.149; ap_prior: 1.74e-05

[infer_isize] inferred maximum insert size: 560 (6.96 sigma)

[bwa_sai2sam_pe_core] time elapses: 25.94 sec

[bwa_sai2sam_pe_core] changing coordinates of 5933 alignments.

[bwa_sai2sam_pe_core] align unmapped mate...

[bwa_paired_sw] 11684 out of 12291 Q17 singletons are mated.

[bwa_paired_sw] 901 out of 1456 Q17 discordant pairs are fixed.

[bwa_sai2sam_pe_core] time elapses: 4.84 sec

[bwa_sai2sam_pe_core] refine gapped alignments... 0.95 sec

[bwa_sai2sam_pe_core] print alignments... 2.06 sec

[bwa_sai2sam_pe_core] 124256256 sequences have been processed.

[bwa_sai2sam_pe_core] convert to sequence coordinate...

[infer_isize] (25, 50, 75) percentile: (453, 463, 473)

[infer_isize] low and high boundaries: 413 and 513 for estimating avg and std

[infer_isize] inferred external isize from 203781 pairs: 463.267 +/- 14.002

[infer_isize] skewness: 0.008; kurtosis: -0.151; ap_prior: 1.67e-05

[infer_isize] inferred maximum insert size: 561 (6.96 sigma)

[bwa_sai2sam_pe_core] time elapses: 29.89 sec

[bwa_sai2sam_pe_core] changing coordinates of 5862 alignments.

[bwa_sai2sam_pe_core] align unmapped mate...

[bwa_paired_sw] 11740 out of 12286 Q17 singletons are mated.

[bwa_paired_sw] 910 out of 1446 Q17 discordant pairs are fixed.

[bwa_sai2sam_pe_core] time elapses: 4.86 sec

[bwa_sai2sam_pe_core] refine gapped alignments... 0.95 sec

[bwa_sai2sam_pe_core] print alignments... 2.16 sec

[bwa_sai2sam_pe_core] 124518400 sequences have been processed.

[bwa_sai2sam_pe_core] convert to sequence coordinate...

[infer_isize] (25, 50, 75) percentile: (453, 463, 473)

[infer_isize] low and high boundaries: 413 and 513 for estimating avg and std

[infer_isize] inferred external isize from 203704 pairs: 463.294 +/- 13.947

[infer_isize] skewness: 0.004; kurtosis: -0.133; ap_prior: 1.83e-05

[infer_isize] inferred maximum insert size: 560 (6.96 sigma)

[bwa_sai2sam_pe_core] time elapses: 29.70 sec

[bwa_sai2sam_pe_core] changing coordinates of 5898 alignments.

[bwa_sai2sam_pe_core] align unmapped mate...

[bwa_paired_sw] 11660 out of 12194 Q17 singletons are mated.

[bwa_paired_sw] 937 out of 1500 Q17 discordant pairs are fixed.

[bwa_sai2sam_pe_core] time elapses: 4.84 sec

[bwa_sai2sam_pe_core] refine gapped alignments... 0.97 sec

[bwa_sai2sam_pe_core] print alignments... 2.11 sec

[bwa_sai2sam_pe_core] 124780544 sequences have been processed.

[bwa_sai2sam_pe_core] convert to sequence coordinate...

[infer_isize] (25, 50, 75) percentile: (453, 463, 473)

[infer_isize] low and high boundaries: 413 and 513 for estimating avg and std

[infer_isize] inferred external isize from 203879 pairs: 463.259 +/- 13.944

[infer_isize] skewness: 0.000; kurtosis: -0.120; ap_prior: 1.84e-05

[infer_isize] inferred maximum insert size: 560 (6.96 sigma)

[bwa_sai2sam_pe_core] time elapses: 29.48 sec

[bwa_sai2sam_pe_core] changing coordinates of 5973 alignments.

[bwa_sai2sam_pe_core] align unmapped mate...

[bwa_paired_sw] 11563 out of 12099 Q17 singletons are mated.

[bwa_paired_sw] 939 out of 1538 Q17 discordant pairs are fixed.

[bwa_sai2sam_pe_core] time elapses: 4.88 sec

[bwa_sai2sam_pe_core] refine gapped alignments... 0.98 sec

[bwa_sai2sam_pe_core] print alignments... 2.17 sec

[bwa_sai2sam_pe_core] 125042688 sequences have been processed.

[bwa_sai2sam_pe_core] convert to sequence coordinate...

[infer_isize] (25, 50, 75) percentile: (453, 463, 473)

[infer_isize] low and high boundaries: 413 and 513 for estimating avg and std

[infer_isize] inferred external isize from 203885 pairs: 463.269 +/- 13.943

[infer_isize] skewness: 0.002; kurtosis: -0.135; ap_prior: 1.71e-05

[infer_isize] inferred maximum insert size: 560 (6.96 sigma)

[bwa_sai2sam_pe_core] time elapses: 30.45 sec

[bwa_sai2sam_pe_core] changing coordinates of 5893 alignments.

[bwa_sai2sam_pe_core] align unmapped mate...

[bwa_paired_sw] 11738 out of 12281 Q17 singletons are mated.

[bwa_paired_sw] 858 out of 1437 Q17 discordant pairs are fixed.

[bwa_sai2sam_pe_core] time elapses: 5.01 sec

[bwa_sai2sam_pe_core] refine gapped alignments... 1.04 sec

[bwa_sai2sam_pe_core] print alignments... 2.22 sec

[bwa_sai2sam_pe_core] 125304832 sequences have been processed.

[bwa_sai2sam_pe_core] convert to sequence coordinate...

[infer_isize] (25, 50, 75) percentile: (453, 463, 473)

[infer_isize] low and high boundaries: 413 and 513 for estimating avg and std

[infer_isize] inferred external isize from 203686 pairs: 463.244 +/- 13.932

[infer_isize] skewness: 0.006; kurtosis: -0.126; ap_prior: 1.62e-05

[infer_isize] inferred maximum insert size: 560 (6.96 sigma)

[bwa_sai2sam_pe_core] time elapses: 32.03 sec

[bwa_sai2sam_pe_core] changing coordinates of 5965 alignments.

[bwa_sai2sam_pe_core] align unmapped mate...

[bwa_paired_sw] 11592 out of 12136 Q17 singletons are mated.

[bwa_paired_sw] 875 out of 1428 Q17 discordant pairs are fixed.

[bwa_sai2sam_pe_core] time elapses: 4.80 sec

[bwa_sai2sam_pe_core] refine gapped alignments... 1.01 sec

[bwa_sai2sam_pe_core] print alignments... 2.12 sec

[bwa_sai2sam_pe_core] 125566976 sequences have been processed.

[bwa_sai2sam_pe_core] convert to sequence coordinate...

[infer_isize] (25, 50, 75) percentile: (453, 463, 473)

[infer_isize] low and high boundaries: 413 and 513 for estimating avg and std

[infer_isize] inferred external isize from 203319 pairs: 463.236 +/- 13.921

[infer_isize] skewness: 0.008; kurtosis: -0.135; ap_prior: 1.67e-05

[infer_isize] inferred maximum insert size: 560 (6.96 sigma)

[bwa_sai2sam_pe_core] time elapses: 28.76 sec

[bwa_sai2sam_pe_core] changing coordinates of 6001 alignments.

[bwa_sai2sam_pe_core] align unmapped mate...

[bwa_paired_sw] 12115 out of 12667 Q17 singletons are mated.

[bwa_paired_sw] 883 out of 1462 Q17 discordant pairs are fixed.

[bwa_sai2sam_pe_core] time elapses: 4.94 sec

[bwa_sai2sam_pe_core] refine gapped alignments... 1.03 sec

[bwa_sai2sam_pe_core] print alignments... 2.20 sec

[bwa_sai2sam_pe_core] 125829120 sequences have been processed.

[bwa_sai2sam_pe_core] convert to sequence coordinate...

[infer_isize] (25, 50, 75) percentile: (453, 463, 473)

[infer_isize] low and high boundaries: 413 and 513 for estimating avg and std

[infer_isize] inferred external isize from 203561 pairs: 463.212 +/- 13.934

[infer_isize] skewness: -0.002; kurtosis: -0.136; ap_prior: 1.66e-05

[infer_isize] inferred maximum insert size: 560 (6.96 sigma)

[bwa_sai2sam_pe_core] time elapses: 28.75 sec

[bwa_sai2sam_pe_core] changing coordinates of 6175 alignments.

[bwa_sai2sam_pe_core] align unmapped mate...

[bwa_paired_sw] 11975 out of 12498 Q17 singletons are mated.

[bwa_paired_sw] 885 out of 1411 Q17 discordant pairs are fixed.

[bwa_sai2sam_pe_core] time elapses: 4.91 sec

[bwa_sai2sam_pe_core] refine gapped alignments... 1.00 sec

[bwa_sai2sam_pe_core] print alignments... 2.14 sec

[bwa_sai2sam_pe_core] 126091264 sequences have been processed.

[bwa_sai2sam_pe_core] convert to sequence coordinate...

[infer_isize] (25, 50, 75) percentile: (453, 463, 473)

[infer_isize] low and high boundaries: 413 and 513 for estimating avg and std

[infer_isize] inferred external isize from 203777 pairs: 463.275 +/- 13.958

[infer_isize] skewness: 0.003; kurtosis: -0.139; ap_prior: 1.67e-05

[infer_isize] inferred maximum insert size: 560 (6.96 sigma)

[bwa_sai2sam_pe_core] time elapses: 28.36 sec

[bwa_sai2sam_pe_core] changing coordinates of 6079 alignments.

[bwa_sai2sam_pe_core] align unmapped mate...

[bwa_paired_sw] 11900 out of 12399 Q17 singletons are mated.

[bwa_paired_sw] 875 out of 1388 Q17 discordant pairs are fixed.

[bwa_sai2sam_pe_core] time elapses: 4.88 sec

[bwa_sai2sam_pe_core] refine gapped alignments... 1.00 sec

[bwa_sai2sam_pe_core] print alignments... 2.12 sec

[bwa_sai2sam_pe_core] 126353408 sequences have been processed.

[bwa_sai2sam_pe_core] convert to sequence coordinate...

[infer_isize] (25, 50, 75) percentile: (453, 463, 473)

[infer_isize] low and high boundaries: 413 and 513 for estimating avg and std

[infer_isize] inferred external isize from 203621 pairs: 463.307 +/- 13.990

[infer_isize] skewness: 0.002; kurtosis: -0.131; ap_prior: 1.81e-05

[infer_isize] inferred maximum insert size: 561 (6.96 sigma)

[bwa_sai2sam_pe_core] time elapses: 24.19 sec

[bwa_sai2sam_pe_core] changing coordinates of 6025 alignments.

[bwa_sai2sam_pe_core] align unmapped mate...

[bwa_paired_sw] 11723 out of 12222 Q17 singletons are mated.

[bwa_paired_sw] 872 out of 1455 Q17 discordant pairs are fixed.

[bwa_sai2sam_pe_core] time elapses: 4.84 sec

[bwa_sai2sam_pe_core] refine gapped alignments... 0.96 sec

[bwa_sai2sam_pe_core] print alignments... 2.08 sec

[bwa_sai2sam_pe_core] 126615552 sequences have been processed.

[bwa_sai2sam_pe_core] convert to sequence coordinate...

[infer_isize] (25, 50, 75) percentile: (453, 463, 473)

[infer_isize] low and high boundaries: 413 and 513 for estimating avg and std

[infer_isize] inferred external isize from 203279 pairs: 463.278 +/- 13.938

[infer_isize] skewness: 0.002; kurtosis: -0.140; ap_prior: 1.60e-05

[infer_isize] inferred maximum insert size: 560 (6.96 sigma)

[bwa_sai2sam_pe_core] time elapses: 24.40 sec

[bwa_sai2sam_pe_core] changing coordinates of 6091 alignments.

[bwa_sai2sam_pe_core] align unmapped mate...

[bwa_paired_sw] 11847 out of 12362 Q17 singletons are mated.

[bwa_paired_sw] 917 out of 1477 Q17 discordant pairs are fixed.

[bwa_sai2sam_pe_core] time elapses: 4.87 sec

[bwa_sai2sam_pe_core] refine gapped alignments... 0.97 sec

[bwa_sai2sam_pe_core] print alignments... 2.07 sec

[bwa_sai2sam_pe_core] 126877696 sequences have been processed.

[bwa_sai2sam_pe_core] convert to sequence coordinate...

[infer_isize] (25, 50, 75) percentile: (453, 463, 473)

[infer_isize] low and high boundaries: 413 and 513 for estimating avg and std

[infer_isize] inferred external isize from 203235 pairs: 463.207 +/- 13.947

[infer_isize] skewness: -0.003; kurtosis: -0.133; ap_prior: 1.74e-05

[infer_isize] inferred maximum insert size: 560 (6.96 sigma)

[bwa_sai2sam_pe_core] time elapses: 27.01 sec

[bwa_sai2sam_pe_core] changing coordinates of 5988 alignments.

[bwa_sai2sam_pe_core] align unmapped mate...

[bwa_paired_sw] 12104 out of 12651 Q17 singletons are mated.

[bwa_paired_sw] 861 out of 1365 Q17 discordant pairs are fixed.

[bwa_sai2sam_pe_core] time elapses: 4.92 sec

[bwa_sai2sam_pe_core] refine gapped alignments... 0.97 sec

[bwa_sai2sam_pe_core] print alignments... 2.12 sec

[bwa_sai2sam_pe_core] 127139840 sequences have been processed.

[bwa_sai2sam_pe_core] convert to sequence coordinate...

[infer_isize] (25, 50, 75) percentile: (453, 463, 473)

[infer_isize] low and high boundaries: 413 and 513 for estimating avg and std

[infer_isize] inferred external isize from 203513 pairs: 463.256 +/- 13.950

[infer_isize] skewness: 0.003; kurtosis: -0.144; ap_prior: 1.84e-05

[infer_isize] inferred maximum insert size: 560 (6.96 sigma)

[bwa_sai2sam_pe_core] time elapses: 24.40 sec

[bwa_sai2sam_pe_core] changing coordinates of 5867 alignments.

[bwa_sai2sam_pe_core] align unmapped mate...

[bwa_paired_sw] 11968 out of 12497 Q17 singletons are mated.

[bwa_paired_sw] 887 out of 1451 Q17 discordant pairs are fixed.

[bwa_sai2sam_pe_core] time elapses: 4.91 sec

[bwa_sai2sam_pe_core] refine gapped alignments... 0.97 sec

[bwa_sai2sam_pe_core] print alignments... 2.13 sec

[bwa_sai2sam_pe_core] 127401984 sequences have been processed.

[bwa_sai2sam_pe_core] convert to sequence coordinate...

[infer_isize] (25, 50, 75) percentile: (453, 463, 473)

[infer_isize] low and high boundaries: 413 and 513 for estimating avg and std

[infer_isize] inferred external isize from 203399 pairs: 463.208 +/- 13.930

[infer_isize] skewness: 0.005; kurtosis: -0.151; ap_prior: 1.54e-05

[infer_isize] inferred maximum insert size: 560 (6.96 sigma)

[bwa_sai2sam_pe_core] time elapses: 24.56 sec

[bwa_sai2sam_pe_core] changing coordinates of 5941 alignments.

[bwa_sai2sam_pe_core] align unmapped mate...

[bwa_paired_sw] 12041 out of 12528 Q17 singletons are mated.

[bwa_paired_sw] 890 out of 1429 Q17 discordant pairs are fixed.

[bwa_sai2sam_pe_core] time elapses: 4.93 sec

[bwa_sai2sam_pe_core] refine gapped alignments... 0.99 sec

[bwa_sai2sam_pe_core] print alignments... 2.18 sec

[bwa_sai2sam_pe_core] 127664128 sequences have been processed.

[bwa_sai2sam_pe_core] convert to sequence coordinate...

[infer_isize] (25, 50, 75) percentile: (453, 463, 473)

[infer_isize] low and high boundaries: 413 and 513 for estimating avg and std

[infer_isize] inferred external isize from 203194 pairs: 463.237 +/- 13.984

[infer_isize] skewness: 0.003; kurtosis: -0.141; ap_prior: 1.61e-05

[infer_isize] inferred maximum insert size: 561 (6.96 sigma)

[bwa_sai2sam_pe_core] time elapses: 24.42 sec

[bwa_sai2sam_pe_core] changing coordinates of 5931 alignments.

[bwa_sai2sam_pe_core] align unmapped mate...

[bwa_paired_sw] 12178 out of 12697 Q17 singletons are mated.

[bwa_paired_sw] 875 out of 1419 Q17 discordant pairs are fixed.

[bwa_sai2sam_pe_core] time elapses: 4.96 sec

[bwa_sai2sam_pe_core] refine gapped alignments... 1.01 sec

[bwa_sai2sam_pe_core] print alignments... 2.15 sec

[bwa_sai2sam_pe_core] 127926272 sequences have been processed.

[bwa_sai2sam_pe_core] convert to sequence coordinate...

[infer_isize] (25, 50, 75) percentile: (453, 463, 473)

[infer_isize] low and high boundaries: 413 and 513 for estimating avg and std

[infer_isize] inferred external isize from 203617 pairs: 463.271 +/- 13.961

[infer_isize] skewness: 0.009; kurtosis: -0.136; ap_prior: 1.82e-05

[infer_isize] inferred maximum insert size: 560 (6.96 sigma)

[bwa_sai2sam_pe_core] time elapses: 24.34 sec

[bwa_sai2sam_pe_core] changing coordinates of 5927 alignments.

[bwa_sai2sam_pe_core] align unmapped mate...

[bwa_paired_sw] 11958 out of 12493 Q17 singletons are mated.

[bwa_paired_sw] 898 out of 1454 Q17 discordant pairs are fixed.

[bwa_sai2sam_pe_core] time elapses: 4.92 sec

[bwa_sai2sam_pe_core] refine gapped alignments... 0.99 sec

[bwa_sai2sam_pe_core] print alignments... 2.12 sec

[bwa_sai2sam_pe_core] 128188416 sequences have been processed.

[bwa_sai2sam_pe_core] convert to sequence coordinate...

[infer_isize] (25, 50, 75) percentile: (453, 463, 473)

[infer_isize] low and high boundaries: 413 and 513 for estimating avg and std

[infer_isize] inferred external isize from 202973 pairs: 463.243 +/- 13.965

[infer_isize] skewness: 0.000; kurtosis: -0.129; ap_prior: 1.73e-05

[infer_isize] inferred maximum insert size: 560 (6.96 sigma)

[bwa_sai2sam_pe_core] time elapses: 24.52 sec

[bwa_sai2sam_pe_core] changing coordinates of 5996 alignments.

[bwa_sai2sam_pe_core] align unmapped mate...

[bwa_paired_sw] 12389 out of 12913 Q17 singletons are mated.

[bwa_paired_sw] 911 out of 1461 Q17 discordant pairs are fixed.

[bwa_sai2sam_pe_core] time elapses: 5.03 sec

[bwa_sai2sam_pe_core] refine gapped alignments... 1.01 sec

[bwa_sai2sam_pe_core] print alignments... 2.20 sec

[bwa_sai2sam_pe_core] 128450560 sequences have been processed.

[bwa_sai2sam_pe_core] convert to sequence coordinate...

[infer_isize] (25, 50, 75) percentile: (453, 463, 473)

[infer_isize] low and high boundaries: 413 and 513 for estimating avg and std

[infer_isize] inferred external isize from 203197 pairs: 463.171 +/- 13.910

[infer_isize] skewness: 0.005; kurtosis: -0.134; ap_prior: 1.83e-05

[infer_isize] inferred maximum insert size: 560 (6.96 sigma)

[bwa_sai2sam_pe_core] time elapses: 25.10 sec

[bwa_sai2sam_pe_core] changing coordinates of 6026 alignments.

[bwa_sai2sam_pe_core] align unmapped mate...

[bwa_paired_sw] 12143 out of 12693 Q17 singletons are mated.

[bwa_paired_sw] 877 out of 1474 Q17 discordant pairs are fixed.

[bwa_sai2sam_pe_core] time elapses: 4.97 sec

[bwa_sai2sam_pe_core] refine gapped alignments... 1.01 sec

[bwa_sai2sam_pe_core] print alignments... 2.16 sec

[bwa_sai2sam_pe_core] 128712704 sequences have been processed.

[bwa_sai2sam_pe_core] convert to sequence coordinate...

[infer_isize] (25, 50, 75) percentile: (453, 463, 473)

[infer_isize] low and high boundaries: 413 and 513 for estimating avg and std

[infer_isize] inferred external isize from 203456 pairs: 463.244 +/- 13.909

[infer_isize] skewness: 0.003; kurtosis: -0.136; ap_prior: 1.79e-05

[infer_isize] inferred maximum insert size: 560 (6.96 sigma)

[bwa_sai2sam_pe_core] time elapses: 24.64 sec

[bwa_sai2sam_pe_core] changing coordinates of 5971 alignments.

[bwa_sai2sam_pe_core] align unmapped mate...

[bwa_paired_sw] 12171 out of 12695 Q17 singletons are mated.

[bwa_paired_sw] 869 out of 1452 Q17 discordant pairs are fixed.

[bwa_sai2sam_pe_core] time elapses: 4.97 sec

[bwa_sai2sam_pe_core] refine gapped alignments... 1.01 sec

[bwa_sai2sam_pe_core] print alignments... 2.17 sec

[bwa_sai2sam_pe_core] 128974848 sequences have been processed.

[bwa_sai2sam_pe_core] convert to sequence coordinate...

[infer_isize] (25, 50, 75) percentile: (453, 463, 473)

[infer_isize] low and high boundaries: 413 and 513 for estimating avg and std

[infer_isize] inferred external isize from 203209 pairs: 463.205 +/- 13.943

[infer_isize] skewness: 0.004; kurtosis: -0.141; ap_prior: 1.62e-05

[infer_isize] inferred maximum insert size: 560 (6.96 sigma)

[bwa_sai2sam_pe_core] time elapses: 24.67 sec

[bwa_sai2sam_pe_core] changing coordinates of 5867 alignments.

[bwa_sai2sam_pe_core] align unmapped mate...

[bwa_paired_sw] 12276 out of 12830 Q17 singletons are mated.

[bwa_paired_sw] 860 out of 1421 Q17 discordant pairs are fixed.

[bwa_sai2sam_pe_core] time elapses: 4.99 sec

[bwa_sai2sam_pe_core] refine gapped alignments... 1.00 sec

[bwa_sai2sam_pe_core] print alignments... 2.19 sec

[bwa_sai2sam_pe_core] 129236992 sequences have been processed.

[bwa_sai2sam_pe_core] convert to sequence coordinate...

[infer_isize] (25, 50, 75) percentile: (453, 463, 473)

[infer_isize] low and high boundaries: 413 and 513 for estimating avg and std

[infer_isize] inferred external isize from 203646 pairs: 463.203 +/- 13.958

[infer_isize] skewness: 0.004; kurtosis: -0.130; ap_prior: 1.58e-05

[infer_isize] inferred maximum insert size: 560 (6.96 sigma)

[bwa_sai2sam_pe_core] time elapses: 31.42 sec

[bwa_sai2sam_pe_core] changing coordinates of 6058 alignments.

[bwa_sai2sam_pe_core] align unmapped mate...

[bwa_paired_sw] 12153 out of 12713 Q17 singletons are mated.

[bwa_paired_sw] 872 out of 1395 Q17 discordant pairs are fixed.

[bwa_sai2sam_pe_core] time elapses: 5.21 sec

[bwa_sai2sam_pe_core] refine gapped alignments... 1.07 sec

[bwa_sai2sam_pe_core] print alignments... 2.17 sec

[bwa_sai2sam_pe_core] 129499136 sequences have been processed.

[bwa_sai2sam_pe_core] convert to sequence coordinate...

[infer_isize] (25, 50, 75) percentile: (453, 463, 473)

[infer_isize] low and high boundaries: 413 and 513 for estimating avg and std

[infer_isize] inferred external isize from 203069 pairs: 463.188 +/- 13.920

[infer_isize] skewness: 0.012; kurtosis: -0.127; ap_prior: 1.72e-05

[infer_isize] inferred maximum insert size: 560 (6.96 sigma)

[bwa_sai2sam_pe_core] time elapses: 47.43 sec

[bwa_sai2sam_pe_core] changing coordinates of 6072 alignments.

[bwa_sai2sam_pe_core] align unmapped mate...

[bwa_paired_sw] 12415 out of 12942 Q17 singletons are mated.

[bwa_paired_sw] 887 out of 1449 Q17 discordant pairs are fixed.

[bwa_sai2sam_pe_core] time elapses: 5.04 sec

[bwa_sai2sam_pe_core] refine gapped alignments... 1.04 sec

[bwa_sai2sam_pe_core] print alignments... 2.17 sec

[bwa_sai2sam_pe_core] 129761280 sequences have been processed.

[bwa_sai2sam_pe_core] convert to sequence coordinate...

[infer_isize] (25, 50, 75) percentile: (453, 463, 473)

[infer_isize] low and high boundaries: 413 and 513 for estimating avg and std

[infer_isize] inferred external isize from 203169 pairs: 463.163 +/- 13.974

[infer_isize] skewness: 0.010; kurtosis: -0.135; ap_prior: 1.85e-05

[infer_isize] inferred maximum insert size: 560 (6.96 sigma)

[bwa_sai2sam_pe_core] time elapses: 26.53 sec

[bwa_sai2sam_pe_core] changing coordinates of 5996 alignments.

[bwa_sai2sam_pe_core] align unmapped mate...

[bwa_paired_sw] 12288 out of 12868 Q17 singletons are mated.

[bwa_paired_sw] 905 out of 1502 Q17 discordant pairs are fixed.

[bwa_sai2sam_pe_core] time elapses: 5.05 sec

[bwa_sai2sam_pe_core] refine gapped alignments... 1.02 sec

[bwa_sai2sam_pe_core] print alignments... 2.13 sec

[bwa_sai2sam_pe_core] 130023424 sequences have been processed.

[bwa_sai2sam_pe_core] convert to sequence coordinate...

[infer_isize] (25, 50, 75) percentile: (453, 463, 473)

[infer_isize] low and high boundaries: 413 and 513 for estimating avg and std

[infer_isize] inferred external isize from 203161 pairs: 463.180 +/- 13.935

[infer_isize] skewness: 0.003; kurtosis: -0.139; ap_prior: 1.79e-05

[infer_isize] inferred maximum insert size: 560 (6.96 sigma)

[bwa_sai2sam_pe_core] time elapses: 26.52 sec

[bwa_sai2sam_pe_core] changing coordinates of 6037 alignments.

[bwa_sai2sam_pe_core] align unmapped mate...

[bwa_paired_sw] 12344 out of 12899 Q17 singletons are mated.

[bwa_paired_sw] 868 out of 1447 Q17 discordant pairs are fixed.

[bwa_sai2sam_pe_core] time elapses: 5.01 sec

[bwa_sai2sam_pe_core] refine gapped alignments... 0.96 sec

[bwa_sai2sam_pe_core] print alignments... 2.14 sec

[bwa_sai2sam_pe_core] 130285568 sequences have been processed.

[bwa_sai2sam_pe_core] convert to sequence coordinate...

[infer_isize] (25, 50, 75) percentile: (453, 463, 473)

[infer_isize] low and high boundaries: 413 and 513 for estimating avg and std

[infer_isize] inferred external isize from 203047 pairs: 463.168 +/- 13.939

[infer_isize] skewness: 0.012; kurtosis: -0.148; ap_prior: 1.71e-05

[infer_isize] inferred maximum insert size: 560 (6.96 sigma)

[bwa_sai2sam_pe_core] time elapses: 25.92 sec

[bwa_sai2sam_pe_core] changing coordinates of 5902 alignments.

[bwa_sai2sam_pe_core] align unmapped mate...

[bwa_paired_sw] 12227 out of 12768 Q17 singletons are mated.

[bwa_paired_sw] 896 out of 1448 Q17 discordant pairs are fixed.

[bwa_sai2sam_pe_core] time elapses: 4.98 sec

[bwa_sai2sam_pe_core] refine gapped alignments... 0.99 sec

[bwa_sai2sam_pe_core] print alignments... 2.15 sec

[bwa_sai2sam_pe_core] 130547712 sequences have been processed.

[bwa_sai2sam_pe_core] convert to sequence coordinate...

[infer_isize] (25, 50, 75) percentile: (453, 463, 473)

[infer_isize] low and high boundaries: 413 and 513 for estimating avg and std

[infer_isize] inferred external isize from 202187 pairs: 463.175 +/- 13.940

[infer_isize] skewness: 0.003; kurtosis: -0.134; ap_prior: 1.67e-05

[infer_isize] inferred maximum insert size: 560 (6.96 sigma)

[bwa_sai2sam_pe_core] time elapses: 25.73 sec

[bwa_sai2sam_pe_core] changing coordinates of 5780 alignments.

[bwa_sai2sam_pe_core] align unmapped mate...

[bwa_paired_sw] 13030 out of 13566 Q17 singletons are mated.

[bwa_paired_sw] 961 out of 1547 Q17 discordant pairs are fixed.

[bwa_sai2sam_pe_core] time elapses: 5.23 sec

[bwa_sai2sam_pe_core] refine gapped alignments... 1.00 sec

[bwa_sai2sam_pe_core] print alignments... 2.16 sec

[bwa_sai2sam_pe_core] 130809856 sequences have been processed.

[bwa_sai2sam_pe_core] convert to sequence coordinate...

[infer_isize] (25, 50, 75) percentile: (453, 463, 473)

[infer_isize] low and high boundaries: 413 and 513 for estimating avg and std

[infer_isize] inferred external isize from 202955 pairs: 463.178 +/- 13.937

[infer_isize] skewness: -0.001; kurtosis: -0.125; ap_prior: 1.70e-05

[infer_isize] inferred maximum insert size: 560 (6.96 sigma)

[bwa_sai2sam_pe_core] time elapses: 26.26 sec

[bwa_sai2sam_pe_core] changing coordinates of 5861 alignments.

[bwa_sai2sam_pe_core] align unmapped mate...

[bwa_paired_sw] 12574 out of 13163 Q17 singletons are mated.

[bwa_paired_sw] 892 out of 1449 Q17 discordant pairs are fixed.

[bwa_sai2sam_pe_core] time elapses: 5.10 sec

[bwa_sai2sam_pe_core] refine gapped alignments... 1.02 sec

[bwa_sai2sam_pe_core] print alignments... 2.15 sec

[bwa_sai2sam_pe_core] 131072000 sequences have been processed.

[bwa_sai2sam_pe_core] convert to sequence coordinate...

[infer_isize] (25, 50, 75) percentile: (453, 463, 473)

[infer_isize] low and high boundaries: 413 and 513 for estimating avg and std

[infer_isize] inferred external isize from 202386 pairs: 463.190 +/- 13.946

[infer_isize] skewness: 0.005; kurtosis: -0.140; ap_prior: 1.76e-05

[infer_isize] inferred maximum insert size: 560 (6.96 sigma)

[bwa_sai2sam_pe_core] time elapses: 26.30 sec

[bwa_sai2sam_pe_core] changing coordinates of 6032 alignments.

[bwa_sai2sam_pe_core] align unmapped mate...

[bwa_paired_sw] 12816 out of 13332 Q17 singletons are mated.

[bwa_paired_sw] 865 out of 1403 Q17 discordant pairs are fixed.

[bwa_sai2sam_pe_core] time elapses: 5.11 sec

[bwa_sai2sam_pe_core] refine gapped alignments... 1.02 sec

[bwa_sai2sam_pe_core] print alignments... 2.12 sec

[bwa_sai2sam_pe_core] 131334144 sequences have been processed.

[bwa_sai2sam_pe_core] convert to sequence coordinate...

[infer_isize] (25, 50, 75) percentile: (453, 463, 473)

[infer_isize] low and high boundaries: 413 and 513 for estimating avg and std

[infer_isize] inferred external isize from 202304 pairs: 463.199 +/- 14.000

[infer_isize] skewness: 0.003; kurtosis: -0.129; ap_prior: 1.76e-05

[infer_isize] inferred maximum insert size: 561 (6.96 sigma)

[bwa_sai2sam_pe_core] time elapses: 29.10 sec

[bwa_sai2sam_pe_core] changing coordinates of 5977 alignments.

[bwa_sai2sam_pe_core] align unmapped mate...

[bwa_paired_sw] 13003 out of 13574 Q17 singletons are mated.

[bwa_paired_sw] 895 out of 1469 Q17 discordant pairs are fixed.

[bwa_sai2sam_pe_core] time elapses: 5.20 sec

[bwa_sai2sam_pe_core] refine gapped alignments... 1.02 sec

[bwa_sai2sam_pe_core] print alignments... 2.16 sec

[bwa_sai2sam_pe_core] 131596288 sequences have been processed.

[bwa_sai2sam_pe_core] convert to sequence coordinate...

[infer_isize] (25, 50, 75) percentile: (453, 463, 473)

[infer_isize] low and high boundaries: 413 and 513 for estimating avg and std

[infer_isize] inferred external isize from 201852 pairs: 463.219 +/- 13.930

[infer_isize] skewness: 0.004; kurtosis: -0.126; ap_prior: 1.64e-05

[infer_isize] inferred maximum insert size: 560 (6.96 sigma)

[bwa_sai2sam_pe_core] time elapses: 29.56 sec

[bwa_sai2sam_pe_core] changing coordinates of 5843 alignments.

[bwa_sai2sam_pe_core] align unmapped mate...

[bwa_paired_sw] 13609 out of 14170 Q17 singletons are mated.

[bwa_paired_sw] 883 out of 1424 Q17 discordant pairs are fixed.

[bwa_sai2sam_pe_core] time elapses: 5.34 sec

[bwa_sai2sam_pe_core] refine gapped alignments... 1.02 sec

[bwa_sai2sam_pe_core] print alignments... 2.17 sec

[bwa_sai2sam_pe_core] 131858432 sequences have been processed.

[bwa_sai2sam_pe_core] convert to sequence coordinate...

[infer_isize] (25, 50, 75) percentile: (453, 463, 473)

[infer_isize] low and high boundaries: 413 and 513 for estimating avg and std

[infer_isize] inferred external isize from 201744 pairs: 463.140 +/- 13.925

[infer_isize] skewness: 0.005; kurtosis: -0.153; ap_prior: 1.61e-05

[infer_isize] inferred maximum insert size: 560 (6.96 sigma)

[bwa_sai2sam_pe_core] time elapses: 30.01 sec

[bwa_sai2sam_pe_core] changing coordinates of 5924 alignments.

[bwa_sai2sam_pe_core] align unmapped mate...

[bwa_paired_sw] 13298 out of 13866 Q17 singletons are mated.

[bwa_paired_sw] 927 out of 1482 Q17 discordant pairs are fixed.

[bwa_sai2sam_pe_core] time elapses: 5.36 sec

[bwa_sai2sam_pe_core] refine gapped alignments... 1.03 sec

[bwa_sai2sam_pe_core] print alignments... 2.20 sec

[bwa_sai2sam_pe_core] 132120576 sequences have been processed.

[bwa_sai2sam_pe_core] convert to sequence coordinate...

[infer_isize] (25, 50, 75) percentile: (453, 463, 473)

[infer_isize] low and high boundaries: 413 and 513 for estimating avg and std

[infer_isize] inferred external isize from 201835 pairs: 463.156 +/- 13.945

[infer_isize] skewness: -0.001; kurtosis: -0.140; ap_prior: 1.73e-05

[infer_isize] inferred maximum insert size: 560 (6.96 sigma)

[bwa_sai2sam_pe_core] time elapses: 32.38 sec

[bwa_sai2sam_pe_core] changing coordinates of 6060 alignments.

[bwa_sai2sam_pe_core] align unmapped mate...

[bwa_paired_sw] 13200 out of 13748 Q17 singletons are mated.

[bwa_paired_sw] 891 out of 1482 Q17 discordant pairs are fixed.

[bwa_sai2sam_pe_core] time elapses: 5.47 sec

[bwa_sai2sam_pe_core] refine gapped alignments... 1.07 sec

[bwa_sai2sam_pe_core] print alignments... 2.18 sec

[bwa_sai2sam_pe_core] 132382720 sequences have been processed.

[bwa_sai2sam_pe_core] convert to sequence coordinate...

[infer_isize] (25, 50, 75) percentile: (453, 463, 473)

[infer_isize] low and high boundaries: 413 and 513 for estimating avg and std

[infer_isize] inferred external isize from 201763 pairs: 463.154 +/- 13.914

[infer_isize] skewness: 0.002; kurtosis: -0.146; ap_prior: 1.89e-05

[infer_isize] inferred maximum insert size: 560 (6.96 sigma)

[bwa_sai2sam_pe_core] time elapses: 31.85 sec

[bwa_sai2sam_pe_core] changing coordinates of 6025 alignments.

[bwa_sai2sam_pe_core] align unmapped mate...

[bwa_paired_sw] 13551 out of 14140 Q17 singletons are mated.

[bwa_paired_sw] 914 out of 1481 Q17 discordant pairs are fixed.

[bwa_sai2sam_pe_core] time elapses: 5.58 sec

[bwa_sai2sam_pe_core] refine gapped alignments... 1.08 sec

[bwa_sai2sam_pe_core] print alignments... 2.14 sec

[bwa_sai2sam_pe_core] 132644864 sequences have been processed.

[bwa_sai2sam_pe_core] convert to sequence coordinate...

[infer_isize] (25, 50, 75) percentile: (453, 463, 473)

[infer_isize] low and high boundaries: 413 and 513 for estimating avg and std

[infer_isize] inferred external isize from 200861 pairs: 463.155 +/- 13.951

[infer_isize] skewness: 0.001; kurtosis: -0.143; ap_prior: 1.63e-05

[infer_isize] inferred maximum insert size: 560 (6.96 sigma)

[bwa_sai2sam_pe_core] time elapses: 30.67 sec

[bwa_sai2sam_pe_core] changing coordinates of 5897 alignments.

[bwa_sai2sam_pe_core] align unmapped mate...

[bwa_paired_sw] 14351 out of 14936 Q17 singletons are mated.

[bwa_paired_sw] 885 out of 1433 Q17 discordant pairs are fixed.

[bwa_sai2sam_pe_core] time elapses: 5.70 sec

[bwa_sai2sam_pe_core] refine gapped alignments... 1.01 sec

[bwa_sai2sam_pe_core] print alignments... 2.09 sec

[bwa_sai2sam_pe_core] 132907008 sequences have been processed.

[bwa_sai2sam_pe_core] convert to sequence coordinate...

[infer_isize] (25, 50, 75) percentile: (453, 463, 473)

[infer_isize] low and high boundaries: 413 and 513 for estimating avg and std

[infer_isize] inferred external isize from 201478 pairs: 463.119 +/- 13.891

[infer_isize] skewness: 0.007; kurtosis: -0.146; ap_prior: 1.66e-05

[infer_isize] inferred maximum insert size: 560 (6.96 sigma)

[bwa_sai2sam_pe_core] time elapses: 30.71 sec

[bwa_sai2sam_pe_core] changing coordinates of 5842 alignments.

[bwa_sai2sam_pe_core] align unmapped mate...

[bwa_paired_sw] 13690 out of 14240 Q17 singletons are mated.

[bwa_paired_sw] 872 out of 1424 Q17 discordant pairs are fixed.

[bwa_sai2sam_pe_core] time elapses: 5.55 sec

[bwa_sai2sam_pe_core] refine gapped alignments... 1.04 sec

[bwa_sai2sam_pe_core] print alignments... 2.16 sec

[bwa_sai2sam_pe_core] 133169152 sequences have been processed.

[bwa_sai2sam_pe_core] convert to sequence coordinate...

[infer_isize] (25, 50, 75) percentile: (453, 463, 473)

[infer_isize] low and high boundaries: 413 and 513 for estimating avg and std

[infer_isize] inferred external isize from 200662 pairs: 463.138 +/- 13.936

[infer_isize] skewness: 0.007; kurtosis: -0.146; ap_prior: 1.65e-05

[infer_isize] inferred maximum insert size: 560 (6.96 sigma)

[bwa_sai2sam_pe_core] time elapses: 31.38 sec

[bwa_sai2sam_pe_core] changing coordinates of 5936 alignments.

[bwa_sai2sam_pe_core] align unmapped mate...

[bwa_paired_sw] 14266 out of 14800 Q17 singletons are mated.

[bwa_paired_sw] 921 out of 1494 Q17 discordant pairs are fixed.

[bwa_sai2sam_pe_core] time elapses: 5.74 sec

[bwa_sai2sam_pe_core] refine gapped alignments... 1.07 sec

[bwa_sai2sam_pe_core] print alignments... 2.18 sec

[bwa_sai2sam_pe_core] 133431296 sequences have been processed.

[bwa_sai2sam_pe_core] convert to sequence coordinate...

[infer_isize] (25, 50, 75) percentile: (453, 463, 473)

[infer_isize] low and high boundaries: 413 and 513 for estimating avg and std

[infer_isize] inferred external isize from 201120 pairs: 463.118 +/- 13.951

[infer_isize] skewness: 0.004; kurtosis: -0.117; ap_prior: 1.47e-05

[infer_isize] inferred maximum insert size: 560 (6.96 sigma)

[bwa_sai2sam_pe_core] time elapses: 31.04 sec

[bwa_sai2sam_pe_core] changing coordinates of 6018 alignments.

[bwa_sai2sam_pe_core] align unmapped mate...

[bwa_paired_sw] 13835 out of 14417 Q17 singletons are mated.

[bwa_paired_sw] 890 out of 1426 Q17 discordant pairs are fixed.

[bwa_sai2sam_pe_core] time elapses: 5.59 sec

[bwa_sai2sam_pe_core] refine gapped alignments... 1.07 sec

[bwa_sai2sam_pe_core] print alignments... 2.23 sec

[bwa_sai2sam_pe_core] 133693440 sequences have been processed.

[bwa_sai2sam_pe_core] convert to sequence coordinate...

[infer_isize] (25, 50, 75) percentile: (453, 463, 473)

[infer_isize] low and high boundaries: 413 and 513 for estimating avg and std

[infer_isize] inferred external isize from 202022 pairs: 463.137 +/- 13.951

[infer_isize] skewness: -0.003; kurtosis: -0.136; ap_prior: 1.51e-05

[infer_isize] inferred maximum insert size: 560 (6.96 sigma)

[bwa_sai2sam_pe_core] time elapses: 31.26 sec

[bwa_sai2sam_pe_core] changing coordinates of 5846 alignments.

[bwa_sai2sam_pe_core] align unmapped mate...

[bwa_paired_sw] 13720 out of 14261 Q17 singletons are mated.

[bwa_paired_sw] 850 out of 1357 Q17 discordant pairs are fixed.

[bwa_sai2sam_pe_core] time elapses: 5.58 sec

[bwa_sai2sam_pe_core] refine gapped alignments... 1.10 sec

[bwa_sai2sam_pe_core] print alignments... 2.16 sec

[bwa_sai2sam_pe_core] 133955584 sequences have been processed.

[bwa_sai2sam_pe_core] convert to sequence coordinate...

[infer_isize] (25, 50, 75) percentile: (453, 463, 473)

[infer_isize] low and high boundaries: 413 and 513 for estimating avg and std

[infer_isize] inferred external isize from 200881 pairs: 463.165 +/- 13.925

[infer_isize] skewness: -0.006; kurtosis: -0.103; ap_prior: 1.70e-05

[infer_isize] inferred maximum insert size: 560 (6.96 sigma)

[bwa_sai2sam_pe_core] time elapses: 31.35 sec

[bwa_sai2sam_pe_core] changing coordinates of 5931 alignments.

[bwa_sai2sam_pe_core] align unmapped mate...

[bwa_paired_sw] 14314 out of 14810 Q17 singletons are mated.

[bwa_paired_sw] 925 out of 1427 Q17 discordant pairs are fixed.

[bwa_sai2sam_pe_core] time elapses: 5.53 sec

[bwa_sai2sam_pe_core] refine gapped alignments... 1.05 sec

[bwa_sai2sam_pe_core] print alignments... 2.17 sec

[bwa_sai2sam_pe_core] 134217728 sequences have been processed.

[bwa_sai2sam_pe_core] convert to sequence coordinate...

[infer_isize] (25, 50, 75) percentile: (453, 463, 473)

[infer_isize] low and high boundaries: 413 and 513 for estimating avg and std

[infer_isize] inferred external isize from 200738 pairs: 463.083 +/- 13.954

[infer_isize] skewness: 0.009; kurtosis: -0.140; ap_prior: 1.67e-05

[infer_isize] inferred maximum insert size: 560 (6.96 sigma)

[bwa_sai2sam_pe_core] time elapses: 26.50 sec

[bwa_sai2sam_pe_core] changing coordinates of 5940 alignments.

[bwa_sai2sam_pe_core] align unmapped mate...

[bwa_paired_sw] 14559 out of 15115 Q17 singletons are mated.

[bwa_paired_sw] 853 out of 1415 Q17 discordant pairs are fixed.

[bwa_sai2sam_pe_core] time elapses: 5.59 sec

[bwa_sai2sam_pe_core] refine gapped alignments... 1.00 sec

[bwa_sai2sam_pe_core] print alignments... 2.16 sec

[bwa_sai2sam_pe_core] 134479872 sequences have been processed.

[bwa_sai2sam_pe_core] convert to sequence coordinate...

[infer_isize] (25, 50, 75) percentile: (453, 463, 473)

[infer_isize] low and high boundaries: 413 and 513 for estimating avg and std

[infer_isize] inferred external isize from 201634 pairs: 463.125 +/- 13.937

[infer_isize] skewness: 0.008; kurtosis: -0.134; ap_prior: 1.64e-05

[infer_isize] inferred maximum insert size: 560 (6.96 sigma)

[bwa_sai2sam_pe_core] time elapses: 27.83 sec

[bwa_sai2sam_pe_core] changing coordinates of 5988 alignments.

[bwa_sai2sam_pe_core] align unmapped mate...

[bwa_paired_sw] 13720 out of 14209 Q17 singletons are mated.

[bwa_paired_sw] 874 out of 1385 Q17 discordant pairs are fixed.

[bwa_sai2sam_pe_core] time elapses: 5.36 sec

[bwa_sai2sam_pe_core] refine gapped alignments... 1.02 sec

[bwa_sai2sam_pe_core] print alignments... 2.16 sec

[bwa_sai2sam_pe_core] 134742016 sequences have been processed.

[bwa_sai2sam_pe_core] convert to sequence coordinate...

[infer_isize] (25, 50, 75) percentile: (453, 463, 473)

[infer_isize] low and high boundaries: 413 and 513 for estimating avg and std

[infer_isize] inferred external isize from 200241 pairs: 463.079 +/- 13.938

[infer_isize] skewness: 0.014; kurtosis: -0.132; ap_prior: 1.61e-05

[infer_isize] inferred maximum insert size: 560 (6.96 sigma)

[bwa_sai2sam_pe_core] time elapses: 27.38 sec

[bwa_sai2sam_pe_core] changing coordinates of 5782 alignments.

[bwa_sai2sam_pe_core] align unmapped mate...

[bwa_paired_sw] 15082 out of 15645 Q17 singletons are mated.

[bwa_paired_sw] 871 out of 1392 Q17 discordant pairs are fixed.

[bwa_sai2sam_pe_core] time elapses: 5.73 sec

[bwa_sai2sam_pe_core] refine gapped alignments... 1.04 sec

[bwa_sai2sam_pe_core] print alignments... 2.17 sec

[bwa_sai2sam_pe_core] 135004160 sequences have been processed.

[bwa_sai2sam_pe_core] convert to sequence coordinate...

[infer_isize] (25, 50, 75) percentile: (453, 463, 473)

[infer_isize] low and high boundaries: 413 and 513 for estimating avg and std

[infer_isize] inferred external isize from 200200 pairs: 463.098 +/- 13.973

[infer_isize] skewness: 0.004; kurtosis: -0.127; ap_prior: 1.67e-05

[infer_isize] inferred maximum insert size: 560 (6.96 sigma)

[bwa_sai2sam_pe_core] time elapses: 27.59 sec

[bwa_sai2sam_pe_core] changing coordinates of 5898 alignments.

[bwa_sai2sam_pe_core] align unmapped mate...

[bwa_paired_sw] 14677 out of 15202 Q17 singletons are mated.

[bwa_paired_sw] 932 out of 1467 Q17 discordant pairs are fixed.

[bwa_sai2sam_pe_core] time elapses: 5.68 sec

[bwa_sai2sam_pe_core] refine gapped alignments... 1.04 sec

[bwa_sai2sam_pe_core] print alignments... 2.22 sec

[bwa_sai2sam_pe_core] 135266304 sequences have been processed.

[bwa_sai2sam_pe_core] convert to sequence coordinate...

[infer_isize] (25, 50, 75) percentile: (453, 463, 473)

[infer_isize] low and high boundaries: 413 and 513 for estimating avg and std

[infer_isize] inferred external isize from 200790 pairs: 463.082 +/- 13.955

[infer_isize] skewness: 0.009; kurtosis: -0.136; ap_prior: 1.64e-05

[infer_isize] inferred maximum insert size: 560 (6.96 sigma)

[bwa_sai2sam_pe_core] time elapses: 32.26 sec

[bwa_sai2sam_pe_core] changing coordinates of 5823 alignments.

[bwa_sai2sam_pe_core] align unmapped mate...

[bwa_paired_sw] 14412 out of 14964 Q17 singletons are mated.

[bwa_paired_sw] 842 out of 1377 Q17 discordant pairs are fixed.

[bwa_sai2sam_pe_core] time elapses: 5.76 sec

[bwa_sai2sam_pe_core] refine gapped alignments... 1.14 sec

[bwa_sai2sam_pe_core] print alignments... 2.14 sec

[bwa_sai2sam_pe_core] 135528448 sequences have been processed.

[bwa_sai2sam_pe_core] convert to sequence coordinate...

[infer_isize] (25, 50, 75) percentile: (453, 463, 473)

[infer_isize] low and high boundaries: 413 and 513 for estimating avg and std

[infer_isize] inferred external isize from 200183 pairs: 463.072 +/- 13.885

[infer_isize] skewness: -0.001; kurtosis: -0.127; ap_prior: 1.80e-05

[infer_isize] inferred maximum insert size: 560 (6.96 sigma)

[bwa_sai2sam_pe_core] time elapses: 41.04 sec

[bwa_sai2sam_pe_core] changing coordinates of 5925 alignments.

[bwa_sai2sam_pe_core] align unmapped mate...

[bwa_paired_sw] 14600 out of 15147 Q17 singletons are mated.

[bwa_paired_sw] 910 out of 1476 Q17 discordant pairs are fixed.

[bwa_sai2sam_pe_core] time elapses: 5.83 sec

[bwa_sai2sam_pe_core] refine gapped alignments... 1.11 sec

[bwa_sai2sam_pe_core] print alignments... 2.19 sec

[bwa_sai2sam_pe_core] 135790592 sequences have been processed.

[bwa_sai2sam_pe_core] convert to sequence coordinate...

[infer_isize] (25, 50, 75) percentile: (453, 463, 473)

[infer_isize] low and high boundaries: 413 and 513 for estimating avg and std

[infer_isize] inferred external isize from 200261 pairs: 463.103 +/- 13.932

[infer_isize] skewness: -0.004; kurtosis: -0.131; ap_prior: 1.82e-05

[infer_isize] inferred maximum insert size: 560 (6.96 sigma)

[bwa_sai2sam_pe_core] time elapses: 31.08 sec

[bwa_sai2sam_pe_core] changing coordinates of 5960 alignments.

[bwa_sai2sam_pe_core] align unmapped mate...

[bwa_paired_sw] 14821 out of 15339 Q17 singletons are mated.

[bwa_paired_sw] 897 out of 1447 Q17 discordant pairs are fixed.

[bwa_sai2sam_pe_core] time elapses: 5.85 sec

[bwa_sai2sam_pe_core] refine gapped alignments... 1.10 sec

[bwa_sai2sam_pe_core] print alignments... 2.12 sec

[bwa_sai2sam_pe_core] 136052736 sequences have been processed.

[bwa_sai2sam_pe_core] convert to sequence coordinate...

[infer_isize] (25, 50, 75) percentile: (453, 463, 473)

[infer_isize] low and high boundaries: 413 and 513 for estimating avg and std

[infer_isize] inferred external isize from 199592 pairs: 463.094 +/- 13.929

[infer_isize] skewness: 0.006; kurtosis: -0.141; ap_prior: 1.86e-05

[infer_isize] inferred maximum insert size: 560 (6.96 sigma)

[bwa_sai2sam_pe_core] time elapses: 30.28 sec

[bwa_sai2sam_pe_core] changing coordinates of 5847 alignments.

[bwa_sai2sam_pe_core] align unmapped mate...

[bwa_paired_sw] 15223 out of 15785 Q17 singletons are mated.

[bwa_paired_sw] 896 out of 1481 Q17 discordant pairs are fixed.

[bwa_sai2sam_pe_core] time elapses: 5.90 sec

[bwa_sai2sam_pe_core] refine gapped alignments... 1.06 sec

[bwa_sai2sam_pe_core] print alignments... 2.12 sec

[bwa_sai2sam_pe_core] 136314880 sequences have been processed.

[bwa_sai2sam_pe_core] convert to sequence coordinate...

[infer_isize] (25, 50, 75) percentile: (453, 463, 473)

[infer_isize] low and high boundaries: 413 and 513 for estimating avg and std

[infer_isize] inferred external isize from 200327 pairs: 463.050 +/- 13.929

[infer_isize] skewness: 0.013; kurtosis: -0.144; ap_prior: 1.66e-05

[infer_isize] inferred maximum insert size: 560 (6.96 sigma)

[bwa_sai2sam_pe_core] time elapses: 29.86 sec

[bwa_sai2sam_pe_core] changing coordinates of 5967 alignments.

[bwa_sai2sam_pe_core] align unmapped mate...

[bwa_paired_sw] 14741 out of 15297 Q17 singletons are mated.

[bwa_paired_sw] 896 out of 1424 Q17 discordant pairs are fixed.

[bwa_sai2sam_pe_core] time elapses: 5.78 sec

[bwa_sai2sam_pe_core] refine gapped alignments... 1.05 sec

[bwa_sai2sam_pe_core] print alignments... 2.20 sec

[bwa_sai2sam_pe_core] 136577024 sequences have been processed.

[bwa_sai2sam_pe_core] convert to sequence coordinate...

[infer_isize] (25, 50, 75) percentile: (453, 463, 473)

[infer_isize] low and high boundaries: 413 and 513 for estimating avg and std

[infer_isize] inferred external isize from 200445 pairs: 463.074 +/- 13.904

[infer_isize] skewness: 0.005; kurtosis: -0.146; ap_prior: 1.73e-05

[infer_isize] inferred maximum insert size: 560 (6.96 sigma)

[bwa_sai2sam_pe_core] time elapses: 30.52 sec

[bwa_sai2sam_pe_core] changing coordinates of 6053 alignments.

[bwa_sai2sam_pe_core] align unmapped mate...

[bwa_paired_sw] 14481 out of 15028 Q17 singletons are mated.

[bwa_paired_sw] 895 out of 1451 Q17 discordant pairs are fixed.

[bwa_sai2sam_pe_core] time elapses: 5.70 sec

[bwa_sai2sam_pe_core] refine gapped alignments... 1.04 sec

[bwa_sai2sam_pe_core] print alignments... 2.18 sec

[bwa_sai2sam_pe_core] 136839168 sequences have been processed.

[bwa_sai2sam_pe_core] convert to sequence coordinate...

[infer_isize] (25, 50, 75) percentile: (453, 463, 473)

[infer_isize] low and high boundaries: 413 and 513 for estimating avg and std

[infer_isize] inferred external isize from 200507 pairs: 463.117 +/- 13.956

[infer_isize] skewness: 0.003; kurtosis: -0.131; ap_prior: 1.51e-05

[infer_isize] inferred maximum insert size: 560 (6.96 sigma)

[bwa_sai2sam_pe_core] time elapses: 30.58 sec

[bwa_sai2sam_pe_core] changing coordinates of 5809 alignments.

[bwa_sai2sam_pe_core] align unmapped mate...

[bwa_paired_sw] 14587 out of 15128 Q17 singletons are mated.

[bwa_paired_sw] 925 out of 1444 Q17 discordant pairs are fixed.

[bwa_sai2sam_pe_core] time elapses: 5.75 sec

[bwa_sai2sam_pe_core] refine gapped alignments... 1.07 sec

[bwa_sai2sam_pe_core] print alignments... 2.18 sec

[bwa_sai2sam_pe_core] 137101312 sequences have been processed.

[bwa_sai2sam_pe_core] convert to sequence coordinate...

[infer_isize] (25, 50, 75) percentile: (453, 463, 473)

[infer_isize] low and high boundaries: 413 and 513 for estimating avg and std

[infer_isize] inferred external isize from 200481 pairs: 463.071 +/- 13.929

[infer_isize] skewness: 0.002; kurtosis: -0.127; ap_prior: 1.76e-05

[infer_isize] inferred maximum insert size: 560 (6.96 sigma)

[bwa_sai2sam_pe_core] time elapses: 30.38 sec

[bwa_sai2sam_pe_core] changing coordinates of 5764 alignments.

[bwa_sai2sam_pe_core] align unmapped mate...

[bwa_paired_sw] 14629 out of 15209 Q17 singletons are mated.

[bwa_paired_sw] 966 out of 1527 Q17 discordant pairs are fixed.

[bwa_sai2sam_pe_core] time elapses: 5.83 sec

[bwa_sai2sam_pe_core] refine gapped alignments... 1.08 sec

[bwa_sai2sam_pe_core] print alignments... 2.18 sec

[bwa_sai2sam_pe_core] 137363456 sequences have been processed.

[bwa_sai2sam_pe_core] convert to sequence coordinate...

[infer_isize] (25, 50, 75) percentile: (453, 463, 473)

[infer_isize] low and high boundaries: 413 and 513 for estimating avg and std

[infer_isize] inferred external isize from 200687 pairs: 463.148 +/- 13.929

[infer_isize] skewness: -0.000; kurtosis: -0.125; ap_prior: 1.71e-05

[infer_isize] inferred maximum insert size: 560 (6.96 sigma)

[bwa_sai2sam_pe_core] time elapses: 30.86 sec

[bwa_sai2sam_pe_core] changing coordinates of 6038 alignments.

[bwa_sai2sam_pe_core] align unmapped mate...

[bwa_paired_sw] 14373 out of 14930 Q17 singletons are mated.

[bwa_paired_sw] 844 out of 1425 Q17 discordant pairs are fixed.

[bwa_sai2sam_pe_core] time elapses: 5.74 sec

[bwa_sai2sam_pe_core] refine gapped alignments... 1.07 sec

[bwa_sai2sam_pe_core] print alignments... 2.20 sec

[bwa_sai2sam_pe_core] 137625600 sequences have been processed.

[bwa_sai2sam_pe_core] convert to sequence coordinate...

[infer_isize] (25, 50, 75) percentile: (453, 463, 473)

[infer_isize] low and high boundaries: 413 and 513 for estimating avg and std

[infer_isize] inferred external isize from 200176 pairs: 463.101 +/- 13.920

[infer_isize] skewness: 0.001; kurtosis: -0.137; ap_prior: 1.76e-05

[infer_isize] inferred maximum insert size: 560 (6.96 sigma)

[bwa_sai2sam_pe_core] time elapses: 29.37 sec

[bwa_sai2sam_pe_core] changing coordinates of 5866 alignments.

[bwa_sai2sam_pe_core] align unmapped mate...

[bwa_paired_sw] 14662 out of 15223 Q17 singletons are mated.

[bwa_paired_sw] 922 out of 1491 Q17 discordant pairs are fixed.

[bwa_sai2sam_pe_core] time elapses: 5.67 sec

[bwa_sai2sam_pe_core] refine gapped alignments... 1.05 sec

[bwa_sai2sam_pe_core] print alignments... 2.19 sec

[bwa_sai2sam_pe_core] 137887744 sequences have been processed.

[bwa_sai2sam_pe_core] convert to sequence coordinate...

[infer_isize] (25, 50, 75) percentile: (453, 463, 473)

[infer_isize] low and high boundaries: 413 and 513 for estimating avg and std

[infer_isize] inferred external isize from 200288 pairs: 463.110 +/- 13.937

[infer_isize] skewness: 0.007; kurtosis: -0.144; ap_prior: 1.69e-05

[infer_isize] inferred maximum insert size: 560 (6.96 sigma)

[bwa_sai2sam_pe_core] time elapses: 29.43 sec

[bwa_sai2sam_pe_core] changing coordinates of 5955 alignments.

[bwa_sai2sam_pe_core] align unmapped mate...

[bwa_paired_sw] 14596 out of 15175 Q17 singletons are mated.

[bwa_paired_sw] 857 out of 1408 Q17 discordant pairs are fixed.

[bwa_sai2sam_pe_core] time elapses: 5.61 sec

[bwa_sai2sam_pe_core] refine gapped alignments... 1.06 sec

[bwa_sai2sam_pe_core] print alignments... 2.19 sec

[bwa_sai2sam_pe_core] 138149888 sequences have been processed.

[bwa_sai2sam_pe_core] convert to sequence coordinate...

[infer_isize] (25, 50, 75) percentile: (453, 463, 473)

[infer_isize] low and high boundaries: 413 and 513 for estimating avg and std

[infer_isize] inferred external isize from 200413 pairs: 463.083 +/- 13.886

[infer_isize] skewness: 0.005; kurtosis: -0.137; ap_prior: 1.73e-05

[infer_isize] inferred maximum insert size: 560 (6.96 sigma)

[bwa_sai2sam_pe_core] time elapses: 31.74 sec

[bwa_sai2sam_pe_core] changing coordinates of 5859 alignments.

[bwa_sai2sam_pe_core] align unmapped mate...

[bwa_paired_sw] 14513 out of 15064 Q17 singletons are mated.

[bwa_paired_sw] 889 out of 1452 Q17 discordant pairs are fixed.

[bwa_sai2sam_pe_core] time elapses: 5.86 sec

[bwa_sai2sam_pe_core] refine gapped alignments... 1.11 sec

[bwa_sai2sam_pe_core] print alignments... 2.17 sec

[bwa_sai2sam_pe_core] 138412032 sequences have been processed.

[bwa_sai2sam_pe_core] convert to sequence coordinate...

[infer_isize] (25, 50, 75) percentile: (453, 463, 473)

[infer_isize] low and high boundaries: 413 and 513 for estimating avg and std

[infer_isize] inferred external isize from 199844 pairs: 463.051 +/- 13.916

[infer_isize] skewness: 0.015; kurtosis: -0.131; ap_prior: 1.60e-05

[infer_isize] inferred maximum insert size: 560 (6.96 sigma)

[bwa_sai2sam_pe_core] time elapses: 31.76 sec

[bwa_sai2sam_pe_core] changing coordinates of 5760 alignments.

[bwa_sai2sam_pe_core] align unmapped mate...

[bwa_paired_sw] 15086 out of 15643 Q17 singletons are mated.

[bwa_paired_sw] 880 out of 1439 Q17 discordant pairs are fixed.

[bwa_sai2sam_pe_core] time elapses: 6.00 sec

[bwa_sai2sam_pe_core] refine gapped alignments... 1.15 sec

[bwa_sai2sam_pe_core] print alignments... 2.15 sec

[bwa_sai2sam_pe_core] 138674176 sequences have been processed.

[bwa_sai2sam_pe_core] convert to sequence coordinate...

[infer_isize] (25, 50, 75) percentile: (453, 463, 473)

[infer_isize] low and high boundaries: 413 and 513 for estimating avg and std

[infer_isize] inferred external isize from 199718 pairs: 462.996 +/- 13.954

[infer_isize] skewness: 0.011; kurtosis: -0.132; ap_prior: 1.78e-05

[infer_isize] inferred maximum insert size: 560 (6.96 sigma)

[bwa_sai2sam_pe_core] time elapses: 31.28 sec

[bwa_sai2sam_pe_core] changing coordinates of 5943 alignments.

[bwa_sai2sam_pe_core] align unmapped mate...

[bwa_paired_sw] 14976 out of 15527 Q17 singletons are mated.

[bwa_paired_sw] 906 out of 1464 Q17 discordant pairs are fixed.

[bwa_sai2sam_pe_core] time elapses: 5.96 sec

[bwa_sai2sam_pe_core] refine gapped alignments... 1.14 sec

[bwa_sai2sam_pe_core] print alignments... 2.12 sec

[bwa_sai2sam_pe_core] 138936320 sequences have been processed.

[bwa_sai2sam_pe_core] convert to sequence coordinate...

[infer_isize] (25, 50, 75) percentile: (453, 463, 473)

[infer_isize] low and high boundaries: 413 and 513 for estimating avg and std

[infer_isize] inferred external isize from 198751 pairs: 463.062 +/- 13.946

[infer_isize] skewness: 0.006; kurtosis: -0.136; ap_prior: 1.74e-05

[infer_isize] inferred maximum insert size: 560 (6.96 sigma)

[bwa_sai2sam_pe_core] time elapses: 31.32 sec

[bwa_sai2sam_pe_core] changing coordinates of 5856 alignments.

[bwa_sai2sam_pe_core] align unmapped mate...

[bwa_paired_sw] 15914 out of 16486 Q17 singletons are mated.

[bwa_paired_sw] 872 out of 1437 Q17 discordant pairs are fixed.

[bwa_sai2sam_pe_core] time elapses: 6.23 sec

[bwa_sai2sam_pe_core] refine gapped alignments... 1.13 sec

[bwa_sai2sam_pe_core] print alignments... 2.08 sec

[bwa_sai2sam_pe_core] 139198464 sequences have been processed.

[bwa_sai2sam_pe_core] convert to sequence coordinate...

[infer_isize] (25, 50, 75) percentile: (453, 463, 473)

[infer_isize] low and high boundaries: 413 and 513 for estimating avg and std

[infer_isize] inferred external isize from 198521 pairs: 463.081 +/- 13.925

[infer_isize] skewness: 0.006; kurtosis: -0.144; ap_prior: 1.86e-05

[infer_isize] inferred maximum insert size: 560 (6.96 sigma)

[bwa_sai2sam_pe_core] time elapses: 30.86 sec

[bwa_sai2sam_pe_core] changing coordinates of 5788 alignments.

[bwa_sai2sam_pe_core] align unmapped mate...

[bwa_paired_sw] 16144 out of 16768 Q17 singletons are mated.

[bwa_paired_sw] 867 out of 1397 Q17 discordant pairs are fixed.

[bwa_sai2sam_pe_core] time elapses: 6.24 sec

[bwa_sai2sam_pe_core] refine gapped alignments... 1.12 sec

[bwa_sai2sam_pe_core] print alignments... 2.08 sec

[bwa_sai2sam_pe_core] 139460608 sequences have been processed.

[bwa_sai2sam_pe_core] convert to sequence coordinate...

[infer_isize] (25, 50, 75) percentile: (453, 463, 473)

[infer_isize] low and high boundaries: 413 and 513 for estimating avg and std

[infer_isize] inferred external isize from 197504 pairs: 463.031 +/- 13.962

[infer_isize] skewness: -0.002; kurtosis: -0.146; ap_prior: 1.83e-05

[infer_isize] inferred maximum insert size: 560 (6.96 sigma)

[bwa_sai2sam_pe_core] time elapses: 30.88 sec

[bwa_sai2sam_pe_core] changing coordinates of 5814 alignments.

[bwa_sai2sam_pe_core] align unmapped mate...

[bwa_paired_sw] 16875 out of 17467 Q17 singletons are mated.

[bwa_paired_sw] 903 out of 1437 Q17 discordant pairs are fixed.

[bwa_sai2sam_pe_core] time elapses: 6.44 sec

[bwa_sai2sam_pe_core] refine gapped alignments... 1.13 sec

[bwa_sai2sam_pe_core] print alignments... 2.08 sec

[bwa_sai2sam_pe_core] 139722752 sequences have been processed.

[bwa_sai2sam_pe_core] convert to sequence coordinate...

[infer_isize] (25, 50, 75) percentile: (453, 463, 473)

[infer_isize] low and high boundaries: 413 and 513 for estimating avg and std

[infer_isize] inferred external isize from 198130 pairs: 463.042 +/- 13.963

[infer_isize] skewness: -0.001; kurtosis: -0.136; ap_prior: 1.68e-05

[infer_isize] inferred maximum insert size: 560 (6.96 sigma)

[bwa_sai2sam_pe_core] time elapses: 30.51 sec

[bwa_sai2sam_pe_core] changing coordinates of 5760 alignments.

[bwa_sai2sam_pe_core] align unmapped mate...

[bwa_paired_sw] 16892 out of 17458 Q17 singletons are mated.

[bwa_paired_sw] 861 out of 1415 Q17 discordant pairs are fixed.

[bwa_sai2sam_pe_core] time elapses: 6.45 sec

[bwa_sai2sam_pe_core] refine gapped alignments... 1.15 sec

[bwa_sai2sam_pe_core] print alignments... 2.20 sec

[bwa_sai2sam_pe_core] 139984896 sequences have been processed.

[bwa_sai2sam_pe_core] convert to sequence coordinate...

[infer_isize] (25, 50, 75) percentile: (453, 463, 473)

[infer_isize] low and high boundaries: 413 and 513 for estimating avg and std

[infer_isize] inferred external isize from 197210 pairs: 463.011 +/- 13.934

[infer_isize] skewness: 0.003; kurtosis: -0.135; ap_prior: 1.54e-05

[infer_isize] inferred maximum insert size: 560 (6.96 sigma)

[bwa_sai2sam_pe_core] time elapses: 30.96 sec

[bwa_sai2sam_pe_core] changing coordinates of 5902 alignments.

[bwa_sai2sam_pe_core] align unmapped mate...

[bwa_paired_sw] 17433 out of 18026 Q17 singletons are mated.

[bwa_paired_sw] 838 out of 1373 Q17 discordant pairs are fixed.

[bwa_sai2sam_pe_core] time elapses: 6.59 sec

[bwa_sai2sam_pe_core] refine gapped alignments... 1.18 sec

[bwa_sai2sam_pe_core] print alignments... 2.19 sec

[bwa_sai2sam_pe_core] 140247040 sequences have been processed.

[bwa_sai2sam_pe_core] convert to sequence coordinate...

[infer_isize] (25, 50, 75) percentile: (453, 463, 473)

[infer_isize] low and high boundaries: 413 and 513 for estimating avg and std

[infer_isize] inferred external isize from 196519 pairs: 463.002 +/- 13.960

[infer_isize] skewness: -0.005; kurtosis: -0.131; ap_prior: 1.75e-05

[infer_isize] inferred maximum insert size: 560 (6.96 sigma)

[bwa_sai2sam_pe_core] time elapses: 30.64 sec

[bwa_sai2sam_pe_core] changing coordinates of 5843 alignments.

[bwa_sai2sam_pe_core] align unmapped mate...

[bwa_paired_sw] 18053 out of 18610 Q17 singletons are mated.

[bwa_paired_sw] 954 out of 1455 Q17 discordant pairs are fixed.

[bwa_sai2sam_pe_core] time elapses: 6.79 sec

[bwa_sai2sam_pe_core] refine gapped alignments... 1.20 sec

[bwa_sai2sam_pe_core] print alignments... 2.20 sec

[bwa_sai2sam_pe_core] 140509184 sequences have been processed.

[bwa_sai2sam_pe_core] convert to sequence coordinate...

[infer_isize] (25, 50, 75) percentile: (453, 463, 473)

[infer_isize] low and high boundaries: 413 and 513 for estimating avg and std

[infer_isize] inferred external isize from 196529 pairs: 463.071 +/- 13.929

[infer_isize] skewness: 0.003; kurtosis: -0.122; ap_prior: 1.67e-05

[infer_isize] inferred maximum insert size: 560 (6.96 sigma)

[bwa_sai2sam_pe_core] time elapses: 31.34 sec

[bwa_sai2sam_pe_core] changing coordinates of 5630 alignments.

[bwa_sai2sam_pe_core] align unmapped mate...

[bwa_paired_sw] 18157 out of 18737 Q17 singletons are mated.

[bwa_paired_sw] 864 out of 1397 Q17 discordant pairs are fixed.

[bwa_sai2sam_pe_core] time elapses: 6.91 sec

[bwa_sai2sam_pe_core] refine gapped alignments... 1.31 sec

[bwa_sai2sam_pe_core] print alignments... 2.34 sec

[bwa_sai2sam_pe_core] 140771328 sequences have been processed.

[bwa_sai2sam_pe_core] convert to sequence coordinate...

[infer_isize] (25, 50, 75) percentile: (453, 463, 473)

[infer_isize] low and high boundaries: 413 and 513 for estimating avg and std

[infer_isize] inferred external isize from 195309 pairs: 462.994 +/- 13.908

[infer_isize] skewness: -0.006; kurtosis: -0.132; ap_prior: 1.51e-05

[infer_isize] inferred maximum insert size: 560 (6.96 sigma)

[bwa_sai2sam_pe_core] time elapses: 31.20 sec

[bwa_sai2sam_pe_core] changing coordinates of 5712 alignments.

[bwa_sai2sam_pe_core] align unmapped mate...

[bwa_paired_sw] 19497 out of 20086 Q17 singletons are mated.

[bwa_paired_sw] 886 out of 1395 Q17 discordant pairs are fixed.

[bwa_sai2sam_pe_core] time elapses: 7.29 sec

[bwa_sai2sam_pe_core] refine gapped alignments... 1.31 sec

[bwa_sai2sam_pe_core] print alignments... 2.40 sec

[bwa_sai2sam_pe_core] 141033472 sequences have been processed.

[bwa_sai2sam_pe_core] convert to sequence coordinate...

[infer_isize] (25, 50, 75) percentile: (453, 463, 473)

[infer_isize] low and high boundaries: 413 and 513 for estimating avg and std

[infer_isize] inferred external isize from 195163 pairs: 463.008 +/- 13.922

[infer_isize] skewness: 0.000; kurtosis: -0.134; ap_prior: 1.60e-05

[infer_isize] inferred maximum insert size: 560 (6.96 sigma)

[bwa_sai2sam_pe_core] time elapses: 35.54 sec

[bwa_sai2sam_pe_core] changing coordinates of 5554 alignments.

[bwa_sai2sam_pe_core] align unmapped mate...

[bwa_paired_sw] 19518 out of 20101 Q17 singletons are mated.

[bwa_paired_sw] 901 out of 1381 Q17 discordant pairs are fixed.

[bwa_sai2sam_pe_core] time elapses: 7.31 sec

[bwa_sai2sam_pe_core] refine gapped alignments... 1.34 sec

[bwa_sai2sam_pe_core] print alignments... 2.25 sec

[bwa_sai2sam_pe_core] 141295616 sequences have been processed.

[bwa_sai2sam_pe_core] convert to sequence coordinate...

[infer_isize] (25, 50, 75) percentile: (453, 463, 473)

[infer_isize] low and high boundaries: 413 and 513 for estimating avg and std

[infer_isize] inferred external isize from 194244 pairs: 462.975 +/- 13.928

[infer_isize] skewness: 0.003; kurtosis: -0.133; ap_prior: 1.66e-05

[infer_isize] inferred maximum insert size: 560 (6.96 sigma)

[bwa_sai2sam_pe_core] time elapses: 30.74 sec

[bwa_sai2sam_pe_core] changing coordinates of 5781 alignments.

[bwa_sai2sam_pe_core] align unmapped mate...

[bwa_paired_sw] 20563 out of 21196 Q17 singletons are mated.

[bwa_paired_sw] 873 out of 1395 Q17 discordant pairs are fixed.

[bwa_sai2sam_pe_core] time elapses: 7.30 sec

[bwa_sai2sam_pe_core] refine gapped alignments... 1.33 sec

[bwa_sai2sam_pe_core] print alignments... 2.22 sec

[bwa_sai2sam_pe_core] 141557760 sequences have been processed.

[bwa_sai2sam_pe_core] convert to sequence coordinate...

[infer_isize] (25, 50, 75) percentile: (453, 463, 473)

[infer_isize] low and high boundaries: 413 and 513 for estimating avg and std

[infer_isize] inferred external isize from 192985 pairs: 462.982 +/- 13.939

[infer_isize] skewness: 0.012; kurtosis: -0.136; ap_prior: 1.45e-05

[infer_isize] inferred maximum insert size: 560 (6.96 sigma)

[bwa_sai2sam_pe_core] time elapses: 31.14 sec

[bwa_sai2sam_pe_core] changing coordinates of 5625 alignments.

[bwa_sai2sam_pe_core] align unmapped mate...

[bwa_paired_sw] 21572 out of 22118 Q17 singletons are mated.

[bwa_paired_sw] 877 out of 1334 Q17 discordant pairs are fixed.

[bwa_sai2sam_pe_core] time elapses: 7.75 sec

[bwa_sai2sam_pe_core] refine gapped alignments... 1.34 sec

[bwa_sai2sam_pe_core] print alignments... 2.22 sec

[bwa_sai2sam_pe_core] 141819904 sequences have been processed.

[bwa_sai2sam_pe_core] convert to sequence coordinate...

[infer_isize] (25, 50, 75) percentile: (453, 463, 473)

[infer_isize] low and high boundaries: 413 and 513 for estimating avg and std

[infer_isize] inferred external isize from 192317 pairs: 462.997 +/- 13.998

[infer_isize] skewness: 0.006; kurtosis: -0.142; ap_prior: 1.64e-05

[infer_isize] inferred maximum insert size: 560 (6.96 sigma)

[bwa_sai2sam_pe_core] time elapses: 30.48 sec

[bwa_sai2sam_pe_core] changing coordinates of 5720 alignments.

[bwa_sai2sam_pe_core] align unmapped mate...

[bwa_paired_sw] 22062 out of 22656 Q17 singletons are mated.

[bwa_paired_sw] 868 out of 1386 Q17 discordant pairs are fixed.

[bwa_sai2sam_pe_core] time elapses: 7.86 sec

[bwa_sai2sam_pe_core] refine gapped alignments... 1.36 sec

[bwa_sai2sam_pe_core] print alignments... 2.18 sec

[bwa_sai2sam_pe_core] 142082048 sequences have been processed.

[bwa_sai2sam_pe_core] convert to sequence coordinate...

[infer_isize] (25, 50, 75) percentile: (453, 463, 473)

[infer_isize] low and high boundaries: 413 and 513 for estimating avg and std

[infer_isize] inferred external isize from 192384 pairs: 462.991 +/- 13.921

[infer_isize] skewness: -0.001; kurtosis: -0.153; ap_prior: 1.67e-05

[infer_isize] inferred maximum insert size: 560 (6.96 sigma)

[bwa_sai2sam_pe_core] time elapses: 30.12 sec

[bwa_sai2sam_pe_core] changing coordinates of 5444 alignments.

[bwa_sai2sam_pe_core] align unmapped mate...

[bwa_paired_sw] 22208 out of 22759 Q17 singletons are mated.

[bwa_paired_sw] 882 out of 1372 Q17 discordant pairs are fixed.

[bwa_sai2sam_pe_core] time elapses: 7.85 sec

[bwa_sai2sam_pe_core] refine gapped alignments... 1.36 sec

[bwa_sai2sam_pe_core] print alignments... 2.11 sec

[bwa_sai2sam_pe_core] 142344192 sequences have been processed.

[bwa_sai2sam_pe_core] convert to sequence coordinate...

[infer_isize] (25, 50, 75) percentile: (453, 463, 473)

[infer_isize] low and high boundaries: 413 and 513 for estimating avg and std

[infer_isize] inferred external isize from 192014 pairs: 462.985 +/- 13.924

[infer_isize] skewness: -0.003; kurtosis: -0.141; ap_prior: 1.61e-05

[infer_isize] inferred maximum insert size: 560 (6.96 sigma)

[bwa_sai2sam_pe_core] time elapses: 29.95 sec

[bwa_sai2sam_pe_core] changing coordinates of 5595 alignments.

[bwa_sai2sam_pe_core] align unmapped mate...

[bwa_paired_sw] 22653 out of 23224 Q17 singletons are mated.

[bwa_paired_sw] 886 out of 1370 Q17 discordant pairs are fixed.

[bwa_sai2sam_pe_core] time elapses: 8.00 sec

[bwa_sai2sam_pe_core] refine gapped alignments... 1.40 sec

[bwa_sai2sam_pe_core] print alignments... 2.09 sec

[bwa_sai2sam_pe_core] 142606336 sequences have been processed.

[bwa_sai2sam_pe_core] convert to sequence coordinate...

[infer_isize] (25, 50, 75) percentile: (453, 463, 473)

[infer_isize] low and high boundaries: 413 and 513 for estimating avg and std

[infer_isize] inferred external isize from 191154 pairs: 462.878 +/- 13.960

[infer_isize] skewness: 0.004; kurtosis: -0.154; ap_prior: 1.67e-05

[infer_isize] inferred maximum insert size: 560 (6.96 sigma)

[bwa_sai2sam_pe_core] time elapses: 29.41 sec

[bwa_sai2sam_pe_core] changing coordinates of 5532 alignments.

[bwa_sai2sam_pe_core] align unmapped mate...

[bwa_paired_sw] 23479 out of 24056 Q17 singletons are mated.

[bwa_paired_sw] 881 out of 1356 Q17 discordant pairs are fixed.

[bwa_sai2sam_pe_core] time elapses: 8.22 sec

[bwa_sai2sam_pe_core] refine gapped alignments... 1.39 sec

[bwa_sai2sam_pe_core] print alignments... 2.06 sec

[bwa_sai2sam_pe_core] 142868480 sequences have been processed.

[bwa_sai2sam_pe_core] convert to sequence coordinate...

[infer_isize] (25, 50, 75) percentile: (453, 463, 473)

[infer_isize] low and high boundaries: 413 and 513 for estimating avg and std

[infer_isize] inferred external isize from 190082 pairs: 462.896 +/- 13.918

[infer_isize] skewness: 0.016; kurtosis: -0.143; ap_prior: 1.59e-05

[infer_isize] inferred maximum insert size: 560 (6.96 sigma)

[bwa_sai2sam_pe_core] time elapses: 29.35 sec

[bwa_sai2sam_pe_core] changing coordinates of 5545 alignments.

[bwa_sai2sam_pe_core] align unmapped mate...

[bwa_paired_sw] 23918 out of 24512 Q17 singletons are mated.

[bwa_paired_sw] 864 out of 1377 Q17 discordant pairs are fixed.

[bwa_sai2sam_pe_core] time elapses: 8.35 sec

[bwa_sai2sam_pe_core] refine gapped alignments... 1.38 sec

[bwa_sai2sam_pe_core] print alignments... 2.06 sec

[bwa_sai2sam_pe_core] 143130624 sequences have been processed.

[bwa_sai2sam_pe_core] convert to sequence coordinate...

[infer_isize] (25, 50, 75) percentile: (453, 463, 472)

[infer_isize] low and high boundaries: 415 and 510 for estimating avg and std

[infer_isize] inferred external isize from 189142 pairs: 462.855 +/- 13.918

[infer_isize] skewness: 0.014; kurtosis: -0.147; ap_prior: 1.49e-05

[infer_isize] inferred maximum insert size: 560 (6.96 sigma)

[bwa_sai2sam_pe_core] time elapses: 29.21 sec

[bwa_sai2sam_pe_core] changing coordinates of 5474 alignments.

[bwa_sai2sam_pe_core] align unmapped mate...

[bwa_paired_sw] 24997 out of 25596 Q17 singletons are mated.

[bwa_paired_sw] 798 out of 1291 Q17 discordant pairs are fixed.

[bwa_sai2sam_pe_core] time elapses: 8.60 sec

[bwa_sai2sam_pe_core] refine gapped alignments... 1.40 sec

[bwa_sai2sam_pe_core] print alignments... 2.11 sec

[bwa_sai2sam_pe_core] 143392768 sequences have been processed.

[bwa_sai2sam_pe_core] convert to sequence coordinate...

[infer_isize] (25, 50, 75) percentile: (453, 463, 473)

[infer_isize] low and high boundaries: 413 and 513 for estimating avg and std

[infer_isize] inferred external isize from 188213 pairs: 462.845 +/- 13.949

[infer_isize] skewness: 0.014; kurtosis: -0.143; ap_prior: 1.59e-05

[infer_isize] inferred maximum insert size: 560 (6.96 sigma)

[bwa_sai2sam_pe_core] time elapses: 29.57 sec

[bwa_sai2sam_pe_core] changing coordinates of 5499 alignments.

[bwa_sai2sam_pe_core] align unmapped mate...

[bwa_paired_sw] 25134 out of 25753 Q17 singletons are mated.

[bwa_paired_sw] 848 out of 1353 Q17 discordant pairs are fixed.

[bwa_sai2sam_pe_core] time elapses: 8.67 sec

[bwa_sai2sam_pe_core] refine gapped alignments... 1.43 sec

[bwa_sai2sam_pe_core] print alignments... 2.14 sec

[bwa_sai2sam_pe_core] 143654912 sequences have been processed.

[bwa_sai2sam_pe_core] convert to sequence coordinate...

[infer_isize] (25, 50, 75) percentile: (453, 463, 473)

[infer_isize] low and high boundaries: 413 and 513 for estimating avg and std

[infer_isize] inferred external isize from 188746 pairs: 462.885 +/- 13.945

[infer_isize] skewness: 0.010; kurtosis: -0.123; ap_prior: 1.45e-05

[infer_isize] inferred maximum insert size: 560 (6.96 sigma)

[bwa_sai2sam_pe_core] time elapses: 29.83 sec

[bwa_sai2sam_pe_core] changing coordinates of 5575 alignments.

[bwa_sai2sam_pe_core] align unmapped mate...

[bwa_paired_sw] 25164 out of 25763 Q17 singletons are mated.

[bwa_paired_sw] 894 out of 1370 Q17 discordant pairs are fixed.

[bwa_sai2sam_pe_core] time elapses: 8.74 sec

[bwa_sai2sam_pe_core] refine gapped alignments... 1.48 sec

[bwa_sai2sam_pe_core] print alignments... 2.26 sec

[bwa_sai2sam_pe_core] 143917056 sequences have been processed.

[bwa_sai2sam_pe_core] convert to sequence coordinate...

[infer_isize] (25, 50, 75) percentile: (453, 463, 473)

[infer_isize] low and high boundaries: 413 and 513 for estimating avg and std

[infer_isize] inferred external isize from 188125 pairs: 462.879 +/- 13.962

[infer_isize] skewness: 0.008; kurtosis: -0.137; ap_prior: 1.62e-05

[infer_isize] inferred maximum insert size: 560 (6.96 sigma)

[bwa_sai2sam_pe_core] time elapses: 28.21 sec

[bwa_sai2sam_pe_core] changing coordinates of 5448 alignments.

[bwa_sai2sam_pe_core] align unmapped mate...

[bwa_paired_sw] 25721 out of 26272 Q17 singletons are mated.

[bwa_paired_sw] 883 out of 1342 Q17 discordant pairs are fixed.

[bwa_sai2sam_pe_core] time elapses: 8.63 sec

[bwa_sai2sam_pe_core] refine gapped alignments... 1.40 sec

[bwa_sai2sam_pe_core] print alignments... 2.24 sec

[bwa_sai2sam_pe_core] 144179200 sequences have been processed.

[bwa_sai2sam_pe_core] convert to sequence coordinate...

[infer_isize] (25, 50, 75) percentile: (453, 463, 473)

[infer_isize] low and high boundaries: 413 and 513 for estimating avg and std

[infer_isize] inferred external isize from 206073 pairs: 463.274 +/- 13.943

[infer_isize] skewness: 0.004; kurtosis: -0.133; ap_prior: 1.72e-05

[infer_isize] inferred maximum insert size: 560 (6.96 sigma)

[bwa_sai2sam_pe_core] time elapses: 32.24 sec

[bwa_sai2sam_pe_core] changing coordinates of 5928 alignments.

[bwa_sai2sam_pe_core] align unmapped mate...

[bwa_paired_sw] 9724 out of 10303 Q17 singletons are mated.

[bwa_paired_sw] 839 out of 1474 Q17 discordant pairs are fixed.

[bwa_sai2sam_pe_core] time elapses: 4.56 sec

[bwa_sai2sam_pe_core] refine gapped alignments... 1.04 sec

[bwa_sai2sam_pe_core] print alignments... 2.24 sec

[bwa_sai2sam_pe_core] 144441344 sequences have been processed.

[bwa_sai2sam_pe_core] convert to sequence coordinate...

[infer_isize] (25, 50, 75) percentile: (453, 463, 473)

[infer_isize] low and high boundaries: 413 and 513 for estimating avg and std

[infer_isize] inferred external isize from 205440 pairs: 463.313 +/- 13.958

[infer_isize] skewness: -0.010; kurtosis: -0.127; ap_prior: 1.78e-05

[infer_isize] inferred maximum insert size: 560 (6.96 sigma)

[bwa_sai2sam_pe_core] time elapses: 34.38 sec

[bwa_sai2sam_pe_core] changing coordinates of 6066 alignments.

[bwa_sai2sam_pe_core] align unmapped mate...

[bwa_paired_sw] 9993 out of 10529 Q17 singletons are mated.

[bwa_paired_sw] 895 out of 1522 Q17 discordant pairs are fixed.

[bwa_sai2sam_pe_core] time elapses: 4.64 sec

[bwa_sai2sam_pe_core] refine gapped alignments... 1.03 sec

[bwa_sai2sam_pe_core] print alignments... 2.24 sec

[bwa_sai2sam_pe_core] 144703488 sequences have been processed.

[bwa_sai2sam_pe_core] convert to sequence coordinate...

[infer_isize] (25, 50, 75) percentile: (453, 463, 473)

[infer_isize] low and high boundaries: 413 and 513 for estimating avg and std

[infer_isize] inferred external isize from 206171 pairs: 463.362 +/- 13.929

[infer_isize] skewness: -0.005; kurtosis: -0.137; ap_prior: 1.75e-05

[infer_isize] inferred maximum insert size: 560 (6.96 sigma)

[bwa_sai2sam_pe_core] time elapses: 33.51 sec

[bwa_sai2sam_pe_core] changing coordinates of 6187 alignments.

[bwa_sai2sam_pe_core] align unmapped mate...

[bwa_paired_sw] 9500 out of 10050 Q17 singletons are mated.

[bwa_paired_sw] 892 out of 1511 Q17 discordant pairs are fixed.

[bwa_sai2sam_pe_core] time elapses: 4.48 sec

[bwa_sai2sam_pe_core] refine gapped alignments... 1.03 sec

[bwa_sai2sam_pe_core] print alignments... 2.21 sec

[bwa_sai2sam_pe_core] 144965632 sequences have been processed.

[bwa_sai2sam_pe_core] convert to sequence coordinate...

[infer_isize] (25, 50, 75) percentile: (453, 463, 473)

[infer_isize] low and high boundaries: 413 and 513 for estimating avg and std

[infer_isize] inferred external isize from 205863 pairs: 463.272 +/- 13.945

[infer_isize] skewness: -0.001; kurtosis: -0.131; ap_prior: 1.89e-05

[infer_isize] inferred maximum insert size: 560 (6.96 sigma)

[bwa_sai2sam_pe_core] time elapses: 32.66 sec

[bwa_sai2sam_pe_core] changing coordinates of 6021 alignments.

[bwa_sai2sam_pe_core] align unmapped mate...

[bwa_paired_sw] 9498 out of 10074 Q17 singletons are mated.

[bwa_paired_sw] 884 out of 1520 Q17 discordant pairs are fixed.

[bwa_sai2sam_pe_core] time elapses: 4.27 sec

[bwa_sai2sam_pe_core] refine gapped alignments... 0.94 sec

[bwa_sai2sam_pe_core] print alignments... 2.13 sec

[bwa_sai2sam_pe_core] 145227776 sequences have been processed.

[bwa_sai2sam_pe_core] convert to sequence coordinate...

[infer_isize] (25, 50, 75) percentile: (453, 463, 473)

[infer_isize] low and high boundaries: 413 and 513 for estimating avg and std

[infer_isize] inferred external isize from 205807 pairs: 463.279 +/- 13.897

[infer_isize] skewness: 0.004; kurtosis: -0.166; ap_prior: 1.50e-05

[infer_isize] inferred maximum insert size: 560 (6.96 sigma)

[bwa_sai2sam_pe_core] time elapses: 30.32 sec

[bwa_sai2sam_pe_core] changing coordinates of 6162 alignments.

[bwa_sai2sam_pe_core] align unmapped mate...

[bwa_paired_sw] 9557 out of 10099 Q17 singletons are mated.

[bwa_paired_sw] 933 out of 1537 Q17 discordant pairs are fixed.

[bwa_sai2sam_pe_core] time elapses: 4.30 sec

[bwa_sai2sam_pe_core] refine gapped alignments... 0.92 sec

[bwa_sai2sam_pe_core] print alignments... 2.20 sec

[bwa_sai2sam_pe_core] 145489920 sequences have been processed.

[bwa_sai2sam_pe_core] convert to sequence coordinate...

[infer_isize] (25, 50, 75) percentile: (453, 463, 473)

[infer_isize] low and high boundaries: 413 and 513 for estimating avg and std

[infer_isize] inferred external isize from 206331 pairs: 463.241 +/- 13.919

[infer_isize] skewness: 0.001; kurtosis: -0.133; ap_prior: 1.84e-05

[infer_isize] inferred maximum insert size: 560 (6.96 sigma)

[bwa_sai2sam_pe_core] time elapses: 29.74 sec

[bwa_sai2sam_pe_core] changing coordinates of 5997 alignments.

[bwa_sai2sam_pe_core] align unmapped mate...

[bwa_paired_sw] 9495 out of 10102 Q17 singletons are mated.

[bwa_paired_sw] 901 out of 1511 Q17 discordant pairs are fixed.

[bwa_sai2sam_pe_core] time elapses: 4.29 sec

[bwa_sai2sam_pe_core] refine gapped alignments... 0.93 sec

[bwa_sai2sam_pe_core] print alignments... 2.21 sec

[bwa_sai2sam_pe_core] 145752064 sequences have been processed.

[bwa_sai2sam_pe_core] convert to sequence coordinate...

[infer_isize] (25, 50, 75) percentile: (453, 463, 473)

[infer_isize] low and high boundaries: 413 and 513 for estimating avg and std

[infer_isize] inferred external isize from 205880 pairs: 463.313 +/- 13.951

[infer_isize] skewness: 0.008; kurtosis: -0.145; ap_prior: 1.76e-05

[infer_isize] inferred maximum insert size: 560 (6.96 sigma)

[bwa_sai2sam_pe_core] time elapses: 29.95 sec

[bwa_sai2sam_pe_core] changing coordinates of 5877 alignments.

[bwa_sai2sam_pe_core] align unmapped mate...

[bwa_paired_sw] 9579 out of 10102 Q17 singletons are mated.

[bwa_paired_sw] 900 out of 1501 Q17 discordant pairs are fixed.

[bwa_sai2sam_pe_core] time elapses: 4.29 sec

[bwa_sai2sam_pe_core] refine gapped alignments... 0.91 sec

[bwa_sai2sam_pe_core] print alignments... 2.21 sec

[bwa_sai2sam_pe_core] 146014208 sequences have been processed.

[bwa_sai2sam_pe_core] convert to sequence coordinate...

[infer_isize] (25, 50, 75) percentile: (453, 463, 473)

[infer_isize] low and high boundaries: 413 and 513 for estimating avg and std

[infer_isize] inferred external isize from 205791 pairs: 463.328 +/- 13.957

[infer_isize] skewness: 0.001; kurtosis: -0.142; ap_prior: 1.77e-05

[infer_isize] inferred maximum insert size: 560 (6.96 sigma)

[bwa_sai2sam_pe_core] time elapses: 30.70 sec

[bwa_sai2sam_pe_core] changing coordinates of 6070 alignments.

[bwa_sai2sam_pe_core] align unmapped mate...

[bwa_paired_sw] 9576 out of 10171 Q17 singletons are mated.

[bwa_paired_sw] 906 out of 1505 Q17 discordant pairs are fixed.

[bwa_sai2sam_pe_core] time elapses: 4.32 sec

[bwa_sai2sam_pe_core] refine gapped alignments... 0.92 sec

[bwa_sai2sam_pe_core] print alignments... 2.15 sec

[bwa_sai2sam_pe_core] 146276352 sequences have been processed.

[bwa_sai2sam_pe_core] convert to sequence coordinate...

[infer_isize] (25, 50, 75) percentile: (453, 463, 473)

[infer_isize] low and high boundaries: 413 and 513 for estimating avg and std

[infer_isize] inferred external isize from 206044 pairs: 463.280 +/- 13.950

[infer_isize] skewness: 0.009; kurtosis: -0.153; ap_prior: 1.83e-05

[infer_isize] inferred maximum insert size: 560 (6.96 sigma)

[bwa_sai2sam_pe_core] time elapses: 30.59 sec

[bwa_sai2sam_pe_core] changing coordinates of 6039 alignments.

[bwa_sai2sam_pe_core] align unmapped mate...

[bwa_paired_sw] 9625 out of 10151 Q17 singletons are mated.

[bwa_paired_sw] 910 out of 1556 Q17 discordant pairs are fixed.

[bwa_sai2sam_pe_core] time elapses: 4.37 sec

[bwa_sai2sam_pe_core] refine gapped alignments... 0.93 sec

[bwa_sai2sam_pe_core] print alignments... 2.23 sec

[bwa_sai2sam_pe_core] 146538496 sequences have been processed.

[bwa_sai2sam_pe_core] convert to sequence coordinate...

[infer_isize] (25, 50, 75) percentile: (453, 463, 473)

[infer_isize] low and high boundaries: 413 and 513 for estimating avg and std

[infer_isize] inferred external isize from 205995 pairs: 463.284 +/- 13.962

[infer_isize] skewness: -0.002; kurtosis: -0.132; ap_prior: 1.77e-05

[infer_isize] inferred maximum insert size: 560 (6.96 sigma)

[bwa_sai2sam_pe_core] time elapses: 32.39 sec

[bwa_sai2sam_pe_core] changing coordinates of 6027 alignments.

[bwa_sai2sam_pe_core] align unmapped mate...

[bwa_paired_sw] 9502 out of 10104 Q17 singletons are mated.

[bwa_paired_sw] 879 out of 1458 Q17 discordant pairs are fixed.

[bwa_sai2sam_pe_core] time elapses: 4.48 sec

[bwa_sai2sam_pe_core] refine gapped alignments... 0.97 sec

[bwa_sai2sam_pe_core] print alignments... 2.24 sec

[bwa_sai2sam_pe_core] 146800640 sequences have been processed.

[bwa_sai2sam_pe_core] convert to sequence coordinate...

[infer_isize] (25, 50, 75) percentile: (453, 463, 473)

[infer_isize] low and high boundaries: 413 and 513 for estimating avg and std

[infer_isize] inferred external isize from 205902 pairs: 463.276 +/- 14.008

[infer_isize] skewness: -0.001; kurtosis: -0.141; ap_prior: 1.91e-05

[infer_isize] inferred maximum insert size: 561 (6.96 sigma)

[bwa_sai2sam_pe_core] time elapses: 33.20 sec

[bwa_sai2sam_pe_core] changing coordinates of 5964 alignments.

[bwa_sai2sam_pe_core] align unmapped mate...

[bwa_paired_sw] 9478 out of 10026 Q17 singletons are mated.

[bwa_paired_sw] 926 out of 1584 Q17 discordant pairs are fixed.

[bwa_sai2sam_pe_core] time elapses: 4.55 sec

[bwa_sai2sam_pe_core] refine gapped alignments... 1.03 sec

[bwa_sai2sam_pe_core] print alignments... 2.31 sec

[bwa_sai2sam_pe_core] 147062784 sequences have been processed.

[bwa_sai2sam_pe_core] convert to sequence coordinate...

[infer_isize] (25, 50, 75) percentile: (453, 463, 473)

[infer_isize] low and high boundaries: 413 and 513 for estimating avg and std

[infer_isize] inferred external isize from 206139 pairs: 463.279 +/- 13.941

[infer_isize] skewness: 0.004; kurtosis: -0.147; ap_prior: 1.70e-05

[infer_isize] inferred maximum insert size: 560 (6.96 sigma)

[bwa_sai2sam_pe_core] time elapses: 31.56 sec

[bwa_sai2sam_pe_core] changing coordinates of 6003 alignments.

[bwa_sai2sam_pe_core] align unmapped mate...

[bwa_paired_sw] 9718 out of 10252 Q17 singletons are mated.

[bwa_paired_sw] 867 out of 1487 Q17 discordant pairs are fixed.

[bwa_sai2sam_pe_core] time elapses: 4.51 sec

[bwa_sai2sam_pe_core] refine gapped alignments... 1.02 sec

[bwa_sai2sam_pe_core] print alignments... 2.29 sec

[bwa_sai2sam_pe_core] 147324928 sequences have been processed.

[bwa_sai2sam_pe_core] convert to sequence coordinate...

[infer_isize] (25, 50, 75) percentile: (453, 463, 473)

[infer_isize] low and high boundaries: 413 and 513 for estimating avg and std

[infer_isize] inferred external isize from 205650 pairs: 463.249 +/- 13.972

[infer_isize] skewness: -0.003; kurtosis: -0.123; ap_prior: 1.64e-05

[infer_isize] inferred maximum insert size: 560 (6.96 sigma)

[bwa_sai2sam_pe_core] time elapses: 32.16 sec

[bwa_sai2sam_pe_core] changing coordinates of 6020 alignments.

[bwa_sai2sam_pe_core] align unmapped mate...

[bwa_paired_sw] 9761 out of 10316 Q17 singletons are mated.

[bwa_paired_sw] 891 out of 1512 Q17 discordant pairs are fixed.

[bwa_sai2sam_pe_core] time elapses: 4.58 sec

[bwa_sai2sam_pe_core] refine gapped alignments... 1.04 sec

[bwa_sai2sam_pe_core] print alignments... 2.29 sec

[bwa_sai2sam_pe_core] 147587072 sequences have been processed.

[bwa_sai2sam_pe_core] convert to sequence coordinate...

[infer_isize] (25, 50, 75) percentile: (453, 463, 473)

[infer_isize] low and high boundaries: 413 and 513 for estimating avg and std

[infer_isize] inferred external isize from 206221 pairs: 463.303 +/- 13.913

[infer_isize] skewness: -0.002; kurtosis: -0.152; ap_prior: 1.68e-05

[infer_isize] inferred maximum insert size: 560 (6.96 sigma)

[bwa_sai2sam_pe_core] time elapses: 31.86 sec

[bwa_sai2sam_pe_core] changing coordinates of 5986 alignments.

[bwa_sai2sam_pe_core] align unmapped mate...

[bwa_paired_sw] 9322 out of 9894 Q17 singletons are mated.

[bwa_paired_sw] 874 out of 1455 Q17 discordant pairs are fixed.

[bwa_sai2sam_pe_core] time elapses: 4.42 sec

[bwa_sai2sam_pe_core] refine gapped alignments... 1.02 sec

[bwa_sai2sam_pe_core] print alignments... 2.25 sec

[bwa_sai2sam_pe_core] 147849216 sequences have been processed.

[bwa_sai2sam_pe_core] convert to sequence coordinate...

[infer_isize] (25, 50, 75) percentile: (453, 463, 473)

[infer_isize] low and high boundaries: 413 and 513 for estimating avg and std

[infer_isize] inferred external isize from 205737 pairs: 463.272 +/- 13.975

[infer_isize] skewness: 0.002; kurtosis: -0.127; ap_prior: 1.71e-05

[infer_isize] inferred maximum insert size: 561 (6.96 sigma)

[bwa_sai2sam_pe_core] time elapses: 32.25 sec

[bwa_sai2sam_pe_core] changing coordinates of 6156 alignments.

[bwa_sai2sam_pe_core] align unmapped mate...

[bwa_paired_sw] 9439 out of 9946 Q17 singletons are mated.

[bwa_paired_sw] 890 out of 1457 Q17 discordant pairs are fixed.

[bwa_sai2sam_pe_core] time elapses: 4.44 sec

[bwa_sai2sam_pe_core] refine gapped alignments... 1.01 sec

[bwa_sai2sam_pe_core] print alignments... 2.17 sec

[bwa_sai2sam_pe_core] 148111360 sequences have been processed.

[bwa_sai2sam_pe_core] convert to sequence coordinate...

[infer_isize] (25, 50, 75) percentile: (453, 463, 473)

[infer_isize] low and high boundaries: 413 and 513 for estimating avg and std

[infer_isize] inferred external isize from 205849 pairs: 463.260 +/- 13.939

[infer_isize] skewness: 0.003; kurtosis: -0.150; ap_prior: 1.79e-05

[infer_isize] inferred maximum insert size: 560 (6.96 sigma)

[bwa_sai2sam_pe_core] time elapses: 31.69 sec

[bwa_sai2sam_pe_core] changing coordinates of 6058 alignments.

[bwa_sai2sam_pe_core] align unmapped mate...

[bwa_paired_sw] 9573 out of 10103 Q17 singletons are mated.

[bwa_paired_sw] 952 out of 1534 Q17 discordant pairs are fixed.

[bwa_sai2sam_pe_core] time elapses: 4.54 sec

[bwa_sai2sam_pe_core] refine gapped alignments... 0.97 sec

[bwa_sai2sam_pe_core] print alignments... 2.25 sec

[bwa_sai2sam_pe_core] 148373504 sequences have been processed.

[bwa_sai2sam_pe_core] convert to sequence coordinate...

[infer_isize] (25, 50, 75) percentile: (453, 463, 473)

[infer_isize] low and high boundaries: 413 and 513 for estimating avg and std

[infer_isize] inferred external isize from 205837 pairs: 463.269 +/- 13.948

[infer_isize] skewness: 0.004; kurtosis: -0.148; ap_prior: 1.70e-05

[infer_isize] inferred maximum insert size: 560 (6.96 sigma)

[bwa_sai2sam_pe_core] time elapses: 31.43 sec

[bwa_sai2sam_pe_core] changing coordinates of 6089 alignments.

[bwa_sai2sam_pe_core] align unmapped mate...

[bwa_paired_sw] 9612 out of 10185 Q17 singletons are mated.

[bwa_paired_sw] 880 out of 1468 Q17 discordant pairs are fixed.

[bwa_sai2sam_pe_core] time elapses: 4.44 sec

[bwa_sai2sam_pe_core] refine gapped alignments... 0.94 sec

[bwa_sai2sam_pe_core] print alignments... 2.21 sec

[bwa_sai2sam_pe_core] 148635648 sequences have been processed.

[bwa_sai2sam_pe_core] convert to sequence coordinate...

[infer_isize] (25, 50, 75) percentile: (453, 463, 473)

[infer_isize] low and high boundaries: 413 and 513 for estimating avg and std

[infer_isize] inferred external isize from 206002 pairs: 463.284 +/- 13.937

[infer_isize] skewness: 0.000; kurtosis: -0.147; ap_prior: 1.68e-05

[infer_isize] inferred maximum insert size: 560 (6.96 sigma)

[bwa_sai2sam_pe_core] time elapses: 31.33 sec

[bwa_sai2sam_pe_core] changing coordinates of 6069 alignments.

[bwa_sai2sam_pe_core] align unmapped mate...

[bwa_paired_sw] 9329 out of 9900 Q17 singletons are mated.

[bwa_paired_sw] 915 out of 1524 Q17 discordant pairs are fixed.

[bwa_sai2sam_pe_core] time elapses: 4.38 sec

[bwa_sai2sam_pe_core] refine gapped alignments... 0.94 sec

[bwa_sai2sam_pe_core] print alignments... 2.20 sec

[bwa_sai2sam_pe_core] 148897792 sequences have been processed.

[bwa_sai2sam_pe_core] convert to sequence coordinate...

[infer_isize] (25, 50, 75) percentile: (453, 463, 473)

[infer_isize] low and high boundaries: 413 and 513 for estimating avg and std

[infer_isize] inferred external isize from 205869 pairs: 463.254 +/- 13.995

[infer_isize] skewness: 0.001; kurtosis: -0.143; ap_prior: 1.62e-05

[infer_isize] inferred maximum insert size: 561 (6.96 sigma)

[bwa_sai2sam_pe_core] time elapses: 32.08 sec

[bwa_sai2sam_pe_core] changing coordinates of 6061 alignments.

[bwa_sai2sam_pe_core] align unmapped mate...

[bwa_paired_sw] 9385 out of 9986 Q17 singletons are mated.

[bwa_paired_sw] 917 out of 1514 Q17 discordant pairs are fixed.

[bwa_sai2sam_pe_core] time elapses: 4.41 sec

[bwa_sai2sam_pe_core] refine gapped alignments... 0.95 sec

[bwa_sai2sam_pe_core] print alignments... 2.22 sec

[bwa_sai2sam_pe_core] 149159936 sequences have been processed.

[bwa_sai2sam_pe_core] convert to sequence coordinate...

[infer_isize] (25, 50, 75) percentile: (453, 463, 473)

[infer_isize] low and high boundaries: 413 and 513 for estimating avg and std

[infer_isize] inferred external isize from 205587 pairs: 463.249 +/- 13.929

[infer_isize] skewness: 0.009; kurtosis: -0.144; ap_prior: 1.79e-05

[infer_isize] inferred maximum insert size: 560 (6.96 sigma)

[bwa_sai2sam_pe_core] time elapses: 31.66 sec

[bwa_sai2sam_pe_core] changing coordinates of 6148 alignments.

[bwa_sai2sam_pe_core] align unmapped mate...

[bwa_paired_sw] 9544 out of 10087 Q17 singletons are mated.

[bwa_paired_sw] 910 out of 1502 Q17 discordant pairs are fixed.

[bwa_sai2sam_pe_core] time elapses: 4.51 sec

[bwa_sai2sam_pe_core] refine gapped alignments... 0.96 sec

[bwa_sai2sam_pe_core] print alignments... 2.17 sec

[bwa_sai2sam_pe_core] 149422080 sequences have been processed.

[bwa_sai2sam_pe_core] convert to sequence coordinate...

[infer_isize] (25, 50, 75) percentile: (453, 463, 473)

[infer_isize] low and high boundaries: 413 and 513 for estimating avg and std

[infer_isize] inferred external isize from 205983 pairs: 463.270 +/- 13.949

[infer_isize] skewness: 0.009; kurtosis: -0.150; ap_prior: 1.67e-05

[infer_isize] inferred maximum insert size: 560 (6.96 sigma)

[bwa_sai2sam_pe_core] time elapses: 32.20 sec

[bwa_sai2sam_pe_core] changing coordinates of 6018 alignments.

[bwa_sai2sam_pe_core] align unmapped mate...

[bwa_paired_sw] 9477 out of 9993 Q17 singletons are mated.

[bwa_paired_sw] 878 out of 1517 Q17 discordant pairs are fixed.

[bwa_sai2sam_pe_core] time elapses: 4.50 sec

[bwa_sai2sam_pe_core] refine gapped alignments... 1.01 sec

[bwa_sai2sam_pe_core] print alignments... 2.19 sec

[bwa_sai2sam_pe_core] 149684224 sequences have been processed.

[bwa_sai2sam_pe_core] convert to sequence coordinate...

[infer_isize] (25, 50, 75) percentile: (453, 463, 473)

[infer_isize] low and high boundaries: 413 and 513 for estimating avg and std

[infer_isize] inferred external isize from 205913 pairs: 463.261 +/- 13.951

[infer_isize] skewness: 0.008; kurtosis: -0.156; ap_prior: 1.56e-05

[infer_isize] inferred maximum insert size: 560 (6.96 sigma)

[bwa_sai2sam_pe_core] time elapses: 32.07 sec

[bwa_sai2sam_pe_core] changing coordinates of 5984 alignments.

[bwa_sai2sam_pe_core] align unmapped mate...

[bwa_paired_sw] 9607 out of 10172 Q17 singletons are mated.

[bwa_paired_sw] 877 out of 1505 Q17 discordant pairs are fixed.

[bwa_sai2sam_pe_core] time elapses: 4.52 sec

[bwa_sai2sam_pe_core] refine gapped alignments... 1.00 sec

[bwa_sai2sam_pe_core] print alignments... 2.27 sec

[bwa_sai2sam_pe_core] 149946368 sequences have been processed.

[bwa_sai2sam_pe_core] convert to sequence coordinate...

[infer_isize] (25, 50, 75) percentile: (453, 463, 473)

[infer_isize] low and high boundaries: 413 and 513 for estimating avg and std

[infer_isize] inferred external isize from 206086 pairs: 463.233 +/- 13.938

[infer_isize] skewness: 0.000; kurtosis: -0.137; ap_prior: 1.88e-05

[infer_isize] inferred maximum insert size: 560 (6.96 sigma)

[bwa_sai2sam_pe_core] time elapses: 32.35 sec

[bwa_sai2sam_pe_core] changing coordinates of 5912 alignments.

[bwa_sai2sam_pe_core] align unmapped mate...

[bwa_paired_sw] 9306 out of 9849 Q17 singletons are mated.

[bwa_paired_sw] 919 out of 1506 Q17 discordant pairs are fixed.

[bwa_sai2sam_pe_core] time elapses: 4.46 sec

[bwa_sai2sam_pe_core] refine gapped alignments... 1.01 sec

[bwa_sai2sam_pe_core] print alignments... 2.24 sec

[bwa_sai2sam_pe_core] 150208512 sequences have been processed.

[bwa_sai2sam_pe_core] convert to sequence coordinate...

[infer_isize] (25, 50, 75) percentile: (453, 463, 473)

[infer_isize] low and high boundaries: 413 and 513 for estimating avg and std

[infer_isize] inferred external isize from 206253 pairs: 463.243 +/- 13.950

[infer_isize] skewness: 0.001; kurtosis: -0.142; ap_prior: 1.74e-05

[infer_isize] inferred maximum insert size: 560 (6.96 sigma)

[bwa_sai2sam_pe_core] time elapses: 32.20 sec

[bwa_sai2sam_pe_core] changing coordinates of 5936 alignments.

[bwa_sai2sam_pe_core] align unmapped mate...

[bwa_paired_sw] 9372 out of 9904 Q17 singletons are mated.

[bwa_paired_sw] 898 out of 1475 Q17 discordant pairs are fixed.

[bwa_sai2sam_pe_core] time elapses: 4.45 sec

[bwa_sai2sam_pe_core] refine gapped alignments... 1.02 sec

[bwa_sai2sam_pe_core] print alignments... 2.28 sec

[bwa_sai2sam_pe_core] 150470656 sequences have been processed.

[bwa_sai2sam_pe_core] convert to sequence coordinate...

[infer_isize] (25, 50, 75) percentile: (453, 463, 473)

[infer_isize] low and high boundaries: 413 and 513 for estimating avg and std

[infer_isize] inferred external isize from 205698 pairs: 463.261 +/- 13.960

[infer_isize] skewness: -0.001; kurtosis: -0.148; ap_prior: 1.73e-05

[infer_isize] inferred maximum insert size: 560 (6.96 sigma)

[bwa_sai2sam_pe_core] time elapses: 32.27 sec

[bwa_sai2sam_pe_core] changing coordinates of 6111 alignments.

[bwa_sai2sam_pe_core] align unmapped mate...

[bwa_paired_sw] 9437 out of 10010 Q17 singletons are mated.

[bwa_paired_sw] 865 out of 1469 Q17 discordant pairs are fixed.

[bwa_sai2sam_pe_core] time elapses: 4.47 sec

[bwa_sai2sam_pe_core] refine gapped alignments... 1.01 sec

[bwa_sai2sam_pe_core] print alignments... 2.32 sec

[bwa_sai2sam_pe_core] 150732800 sequences have been processed.

[bwa_sai2sam_pe_core] convert to sequence coordinate...

[infer_isize] (25, 50, 75) percentile: (453, 463, 473)

[infer_isize] low and high boundaries: 413 and 513 for estimating avg and std

[infer_isize] inferred external isize from 205624 pairs: 463.243 +/- 13.964

[infer_isize] skewness: 0.006; kurtosis: -0.126; ap_prior: 1.62e-05

[infer_isize] inferred maximum insert size: 560 (6.96 sigma)

[bwa_sai2sam_pe_core] time elapses: 31.73 sec

[bwa_sai2sam_pe_core] changing coordinates of 6013 alignments.

[bwa_sai2sam_pe_core] align unmapped mate...

[bwa_paired_sw] 9437 out of 9992 Q17 singletons are mated.

[bwa_paired_sw] 894 out of 1484 Q17 discordant pairs are fixed.

[bwa_sai2sam_pe_core] time elapses: 4.47 sec

[bwa_sai2sam_pe_core] refine gapped alignments... 1.02 sec

[bwa_sai2sam_pe_core] print alignments... 2.25 sec

[bwa_sai2sam_pe_core] 150994944 sequences have been processed.

[bwa_sai2sam_pe_core] convert to sequence coordinate...

[infer_isize] (25, 50, 75) percentile: (453, 463, 473)

[infer_isize] low and high boundaries: 413 and 513 for estimating avg and std

[infer_isize] inferred external isize from 206009 pairs: 463.264 +/- 13.962

[infer_isize] skewness: 0.003; kurtosis: -0.137; ap_prior: 1.81e-05

[infer_isize] inferred maximum insert size: 560 (6.96 sigma)

[bwa_sai2sam_pe_core] time elapses: 31.98 sec

[bwa_sai2sam_pe_core] changing coordinates of 6061 alignments.

[bwa_sai2sam_pe_core] align unmapped mate...

[bwa_paired_sw] 9383 out of 9909 Q17 singletons are mated.

[bwa_paired_sw] 934 out of 1524 Q17 discordant pairs are fixed.

[bwa_sai2sam_pe_core] time elapses: 4.47 sec

[bwa_sai2sam_pe_core] refine gapped alignments... 1.02 sec

[bwa_sai2sam_pe_core] print alignments... 2.33 sec

[bwa_sai2sam_pe_core] 151257088 sequences have been processed.

[bwa_sai2sam_pe_core] convert to sequence coordinate...

[infer_isize] (25, 50, 75) percentile: (453, 463, 473)

[infer_isize] low and high boundaries: 413 and 513 for estimating avg and std

[infer_isize] inferred external isize from 205665 pairs: 463.253 +/- 13.993

[infer_isize] skewness: -0.001; kurtosis: -0.154; ap_prior: 1.77e-05

[infer_isize] inferred maximum insert size: 561 (6.96 sigma)

[bwa_sai2sam_pe_core] time elapses: 32.73 sec

[bwa_sai2sam_pe_core] changing coordinates of 6100 alignments.

[bwa_sai2sam_pe_core] align unmapped mate...

[bwa_paired_sw] 9608 out of 10109 Q17 singletons are mated.

[bwa_paired_sw] 902 out of 1520 Q17 discordant pairs are fixed.

[bwa_sai2sam_pe_core] time elapses: 4.53 sec

[bwa_sai2sam_pe_core] refine gapped alignments... 1.00 sec

[bwa_sai2sam_pe_core] print alignments... 8.65 sec

[bwa_sai2sam_pe_core] 151519232 sequences have been processed.

[bwa_sai2sam_pe_core] convert to sequence coordinate...

[infer_isize] (25, 50, 75) percentile: (453, 463, 473)

[infer_isize] low and high boundaries: 413 and 513 for estimating avg and std

[infer_isize] inferred external isize from 206229 pairs: 463.252 +/- 13.921

[infer_isize] skewness: 0.002; kurtosis: -0.142; ap_prior: 1.89e-05

[infer_isize] inferred maximum insert size: 560 (6.96 sigma)

[bwa_sai2sam_pe_core] time elapses: 145.90 sec

[bwa_sai2sam_pe_core] changing coordinates of 6029 alignments.

[bwa_sai2sam_pe_core] align unmapped mate...

[bwa_paired_sw] 9238 out of 9775 Q17 singletons are mated.

[bwa_paired_sw] 928 out of 1521 Q17 discordant pairs are fixed.

[bwa_sai2sam_pe_core] time elapses: 22.43 sec

[bwa_sai2sam_pe_core] refine gapped alignments... 5.44 sec

[bwa_sai2sam_pe_core] print alignments... 11.42 sec

[bwa_sai2sam_pe_core] 151781376 sequences have been processed.

[bwa_sai2sam_pe_core] convert to sequence coordinate...

[infer_isize] (25, 50, 75) percentile: (453, 463, 473)

[infer_isize] low and high boundaries: 413 and 513 for estimating avg and std

[infer_isize] inferred external isize from 205871 pairs: 463.242 +/- 13.932

[infer_isize] skewness: 0.003; kurtosis: -0.150; ap_prior: 1.67e-05

[infer_isize] inferred maximum insert size: 560 (6.96 sigma)

[bwa_sai2sam_pe_core] time elapses: 139.29 sec

[bwa_sai2sam_pe_core] changing coordinates of 6118 alignments.

[bwa_sai2sam_pe_core] align unmapped mate...

[bwa_paired_sw] 9425 out of 9933 Q17 singletons are mated.

[bwa_paired_sw] 927 out of 1537 Q17 discordant pairs are fixed.

[bwa_sai2sam_pe_core] time elapses: 20.94 sec

[bwa_sai2sam_pe_core] refine gapped alignments... 5.22 sec

[bwa_sai2sam_pe_core] print alignments... 13.55 sec

[bwa_sai2sam_pe_core] 152043520 sequences have been processed.

[bwa_sai2sam_pe_core] convert to sequence coordinate...

[infer_isize] (25, 50, 75) percentile: (453, 463, 473)

[infer_isize] low and high boundaries: 413 and 513 for estimating avg and std

[infer_isize] inferred external isize from 205766 pairs: 463.245 +/- 13.973

[infer_isize] skewness: -0.005; kurtosis: -0.126; ap_prior: 1.76e-05

[infer_isize] inferred maximum insert size: 560 (6.96 sigma)

[bwa_sai2sam_pe_core] time elapses: 133.72 sec

[bwa_sai2sam_pe_core] changing coordinates of 5977 alignments.

[bwa_sai2sam_pe_core] align unmapped mate...

[bwa_paired_sw] 9384 out of 9910 Q17 singletons are mated.

[bwa_paired_sw] 907 out of 1515 Q17 discordant pairs are fixed.

[bwa_sai2sam_pe_core] time elapses: 21.13 sec

[bwa_sai2sam_pe_core] refine gapped alignments... 4.66 sec

[bwa_sai2sam_pe_core] print alignments... 11.67 sec

[bwa_sai2sam_pe_core] 152305664 sequences have been processed.

[bwa_sai2sam_pe_core] convert to sequence coordinate...

[infer_isize] (25, 50, 75) percentile: (453, 463, 473)

[infer_isize] low and high boundaries: 413 and 513 for estimating avg and std

[infer_isize] inferred external isize from 206030 pairs: 463.255 +/- 13.952

[infer_isize] skewness: 0.003; kurtosis: -0.145; ap_prior: 1.71e-05

[infer_isize] inferred maximum insert size: 560 (6.96 sigma)

[bwa_sai2sam_pe_core] time elapses: 34.73 sec

[bwa_sai2sam_pe_core] changing coordinates of 5984 alignments.

[bwa_sai2sam_pe_core] align unmapped mate...

[bwa_paired_sw] 9456 out of 10009 Q17 singletons are mated.

[bwa_paired_sw] 893 out of 1460 Q17 discordant pairs are fixed.

[bwa_sai2sam_pe_core] time elapses: 4.26 sec

[bwa_sai2sam_pe_core] refine gapped alignments... 0.91 sec

[bwa_sai2sam_pe_core] print alignments... 2.17 sec

[bwa_sai2sam_pe_core] 152567808 sequences have been processed.

[bwa_sai2sam_pe_core] convert to sequence coordinate...

[infer_isize] (25, 50, 75) percentile: (453, 463, 473)

[infer_isize] low and high boundaries: 413 and 513 for estimating avg and std

[infer_isize] inferred external isize from 205996 pairs: 463.278 +/- 13.947

[infer_isize] skewness: -0.005; kurtosis: -0.136; ap_prior: 1.98e-05

[infer_isize] inferred maximum insert size: 560 (6.96 sigma)

[bwa_sai2sam_pe_core] time elapses: 27.54 sec

[bwa_sai2sam_pe_core] changing coordinates of 6133 alignments.

[bwa_sai2sam_pe_core] align unmapped mate...

[bwa_paired_sw] 9152 out of 9651 Q17 singletons are mated.

[bwa_paired_sw] 935 out of 1526 Q17 discordant pairs are fixed.

[bwa_sai2sam_pe_core] time elapses: 4.18 sec

[bwa_sai2sam_pe_core] refine gapped alignments... 0.93 sec

[bwa_sai2sam_pe_core] print alignments... 2.18 sec

[bwa_sai2sam_pe_core] 152829952 sequences have been processed.

[bwa_sai2sam_pe_core] convert to sequence coordinate...

[infer_isize] (25, 50, 75) percentile: (453, 463, 473)

[infer_isize] low and high boundaries: 413 and 513 for estimating avg and std

[infer_isize] inferred external isize from 205496 pairs: 463.262 +/- 13.942

[infer_isize] skewness: -0.003; kurtosis: -0.157; ap_prior: 1.62e-05

[infer_isize] inferred maximum insert size: 560 (6.96 sigma)

[bwa_sai2sam_pe_core] time elapses: 27.18 sec

[bwa_sai2sam_pe_core] changing coordinates of 6032 alignments.

[bwa_sai2sam_pe_core] align unmapped mate...

[bwa_paired_sw] 9610 out of 10171 Q17 singletons are mated.

[bwa_paired_sw] 871 out of 1522 Q17 discordant pairs are fixed.

[bwa_sai2sam_pe_core] time elapses: 4.29 sec

[bwa_sai2sam_pe_core] refine gapped alignments... 0.93 sec

[bwa_sai2sam_pe_core] print alignments... 2.16 sec

[bwa_sai2sam_pe_core] 153092096 sequences have been processed.

[bwa_sai2sam_pe_core] convert to sequence coordinate...

[infer_isize] (25, 50, 75) percentile: (453, 463, 473)

[infer_isize] low and high boundaries: 413 and 513 for estimating avg and std

[infer_isize] inferred external isize from 205680 pairs: 463.211 +/- 13.966

[infer_isize] skewness: 0.004; kurtosis: -0.125; ap_prior: 1.66e-05

[infer_isize] inferred maximum insert size: 560 (6.96 sigma)

[bwa_sai2sam_pe_core] time elapses: 27.52 sec

[bwa_sai2sam_pe_core] changing coordinates of 6036 alignments.

[bwa_sai2sam_pe_core] align unmapped mate...

[bwa_paired_sw] 9410 out of 9922 Q17 singletons are mated.

[bwa_paired_sw] 949 out of 1579 Q17 discordant pairs are fixed.

[bwa_sai2sam_pe_core] time elapses: 4.27 sec

[bwa_sai2sam_pe_core] refine gapped alignments... 0.93 sec

[bwa_sai2sam_pe_core] print alignments... 2.17 sec

[bwa_sai2sam_pe_core] 153354240 sequences have been processed.

[bwa_sai2sam_pe_core] convert to sequence coordinate...

[infer_isize] (25, 50, 75) percentile: (453, 463, 473)

[infer_isize] low and high boundaries: 413 and 513 for estimating avg and std

[infer_isize] inferred external isize from 206233 pairs: 463.237 +/- 13.967

[infer_isize] skewness: -0.012; kurtosis: -0.121; ap_prior: 1.72e-05

[infer_isize] inferred maximum insert size: 560 (6.96 sigma)

[bwa_sai2sam_pe_core] time elapses: 27.05 sec

[bwa_sai2sam_pe_core] changing coordinates of 5966 alignments.

[bwa_sai2sam_pe_core] align unmapped mate...

[bwa_paired_sw] 9263 out of 9788 Q17 singletons are mated.

[bwa_paired_sw] 901 out of 1477 Q17 discordant pairs are fixed.

[bwa_sai2sam_pe_core] time elapses: 4.20 sec

[bwa_sai2sam_pe_core] refine gapped alignments... 0.95 sec

[bwa_sai2sam_pe_core] print alignments... 2.15 sec

[bwa_sai2sam_pe_core] 153616384 sequences have been processed.

[bwa_sai2sam_pe_core] convert to sequence coordinate...

[infer_isize] (25, 50, 75) percentile: (453, 463, 473)

[infer_isize] low and high boundaries: 413 and 513 for estimating avg and std

[infer_isize] inferred external isize from 205699 pairs: 463.246 +/- 13.931

[infer_isize] skewness: 0.004; kurtosis: -0.143; ap_prior: 1.75e-05

[infer_isize] inferred maximum insert size: 560 (6.96 sigma)

[bwa_sai2sam_pe_core] time elapses: 29.49 sec

[bwa_sai2sam_pe_core] changing coordinates of 6092 alignments.

[bwa_sai2sam_pe_core] align unmapped mate...

[bwa_paired_sw] 9195 out of 9713 Q17 singletons are mated.

[bwa_paired_sw] 915 out of 1523 Q17 discordant pairs are fixed.

[bwa_sai2sam_pe_core] time elapses: 4.23 sec

[bwa_sai2sam_pe_core] refine gapped alignments... 0.94 sec

[bwa_sai2sam_pe_core] print alignments... 2.16 sec

[bwa_sai2sam_pe_core] 153878528 sequences have been processed.

[bwa_sai2sam_pe_core] convert to sequence coordinate...

[infer_isize] (25, 50, 75) percentile: (453, 463, 473)

[infer_isize] low and high boundaries: 413 and 513 for estimating avg and std

[infer_isize] inferred external isize from 205738 pairs: 463.238 +/- 13.938

[infer_isize] skewness: 0.002; kurtosis: -0.132; ap_prior: 1.66e-05

[infer_isize] inferred maximum insert size: 560 (6.96 sigma)

[bwa_sai2sam_pe_core] time elapses: 29.47 sec

[bwa_sai2sam_pe_core] changing coordinates of 6046 alignments.

[bwa_sai2sam_pe_core] align unmapped mate...

[bwa_paired_sw] 9445 out of 9947 Q17 singletons are mated.

[bwa_paired_sw] 831 out of 1466 Q17 discordant pairs are fixed.

[bwa_sai2sam_pe_core] time elapses: 4.21 sec

[bwa_sai2sam_pe_core] refine gapped alignments... 0.92 sec

[bwa_sai2sam_pe_core] print alignments... 2.14 sec

[bwa_sai2sam_pe_core] 154140672 sequences have been processed.

[bwa_sai2sam_pe_core] convert to sequence coordinate...

[infer_isize] (25, 50, 75) percentile: (453, 463, 473)

[infer_isize] low and high boundaries: 413 and 513 for estimating avg and std

[infer_isize] inferred external isize from 205932 pairs: 463.190 +/- 13.940

[infer_isize] skewness: 0.005; kurtosis: -0.149; ap_prior: 1.74e-05

[infer_isize] inferred maximum insert size: 560 (6.96 sigma)

[bwa_sai2sam_pe_core] time elapses: 35.99 sec

[bwa_sai2sam_pe_core] changing coordinates of 6003 alignments.

[bwa_sai2sam_pe_core] align unmapped mate...

[bwa_paired_sw] 9364 out of 9946 Q17 singletons are mated.

[bwa_paired_sw] 879 out of 1503 Q17 discordant pairs are fixed.

[bwa_sai2sam_pe_core] time elapses: 4.27 sec

[bwa_sai2sam_pe_core] refine gapped alignments... 0.92 sec

[bwa_sai2sam_pe_core] print alignments... 2.11 sec

[bwa_sai2sam_pe_core] 154402816 sequences have been processed.

[bwa_sai2sam_pe_core] convert to sequence coordinate...

[infer_isize] (25, 50, 75) percentile: (453, 463, 473)

[infer_isize] low and high boundaries: 413 and 513 for estimating avg and std

[infer_isize] inferred external isize from 206062 pairs: 463.199 +/- 13.974

[infer_isize] skewness: -0.005; kurtosis: -0.136; ap_prior: 1.77e-05

[infer_isize] inferred maximum insert size: 560 (6.96 sigma)

[bwa_sai2sam_pe_core] time elapses: 29.03 sec

[bwa_sai2sam_pe_core] changing coordinates of 6003 alignments.

[bwa_sai2sam_pe_core] align unmapped mate...

[bwa_paired_sw] 9208 out of 9785 Q17 singletons are mated.

[bwa_paired_sw] 883 out of 1510 Q17 discordant pairs are fixed.

[bwa_sai2sam_pe_core] time elapses: 4.24 sec

[bwa_sai2sam_pe_core] refine gapped alignments... 0.92 sec

[bwa_sai2sam_pe_core] print alignments... 2.15 sec

[bwa_sai2sam_pe_core] 154664960 sequences have been processed.

[bwa_sai2sam_pe_core] convert to sequence coordinate...

[infer_isize] (25, 50, 75) percentile: (453, 463, 473)

[infer_isize] low and high boundaries: 413 and 513 for estimating avg and std

[infer_isize] inferred external isize from 206464 pairs: 463.235 +/- 13.976

[infer_isize] skewness: 0.007; kurtosis: -0.142; ap_prior: 1.56e-05

[infer_isize] inferred maximum insert size: 561 (6.96 sigma)

[bwa_sai2sam_pe_core] time elapses: 31.39 sec

[bwa_sai2sam_pe_core] changing coordinates of 6085 alignments.

[bwa_sai2sam_pe_core] align unmapped mate...

[bwa_paired_sw] 9310 out of 9808 Q17 singletons are mated.

[bwa_paired_sw] 874 out of 1452 Q17 discordant pairs are fixed.

[bwa_sai2sam_pe_core] time elapses: 4.33 sec

[bwa_sai2sam_pe_core] refine gapped alignments... 0.95 sec

[bwa_sai2sam_pe_core] print alignments... 2.21 sec

[bwa_sai2sam_pe_core] 154927104 sequences have been processed.

[bwa_sai2sam_pe_core] convert to sequence coordinate...

[infer_isize] (25, 50, 75) percentile: (453, 463, 473)

[infer_isize] low and high boundaries: 413 and 513 for estimating avg and std

[infer_isize] inferred external isize from 205928 pairs: 463.224 +/- 13.960

[infer_isize] skewness: -0.000; kurtosis: -0.137; ap_prior: 1.69e-05

[infer_isize] inferred maximum insert size: 560 (6.96 sigma)

[bwa_sai2sam_pe_core] time elapses: 31.30 sec

[bwa_sai2sam_pe_core] changing coordinates of 6080 alignments.

[bwa_sai2sam_pe_core] align unmapped mate...

[bwa_paired_sw] 9268 out of 9778 Q17 singletons are mated.

[bwa_paired_sw] 897 out of 1514 Q17 discordant pairs are fixed.

[bwa_sai2sam_pe_core] time elapses: 4.39 sec

[bwa_sai2sam_pe_core] refine gapped alignments... 0.97 sec

[bwa_sai2sam_pe_core] print alignments... 2.18 sec

[bwa_sai2sam_pe_core] 155189248 sequences have been processed.

[bwa_sai2sam_pe_core] convert to sequence coordinate...

[infer_isize] (25, 50, 75) percentile: (453, 463, 473)

[infer_isize] low and high boundaries: 413 and 513 for estimating avg and std

[infer_isize] inferred external isize from 205494 pairs: 463.167 +/- 13.965

[infer_isize] skewness: 0.004; kurtosis: -0.132; ap_prior: 1.64e-05

[infer_isize] inferred maximum insert size: 560 (6.96 sigma)

[bwa_sai2sam_pe_core] time elapses: 33.22 sec

[bwa_sai2sam_pe_core] changing coordinates of 6044 alignments.

[bwa_sai2sam_pe_core] align unmapped mate...

[bwa_paired_sw] 9485 out of 10006 Q17 singletons are mated.

[bwa_paired_sw] 898 out of 1494 Q17 discordant pairs are fixed.

[bwa_sai2sam_pe_core] time elapses: 4.48 sec

[bwa_sai2sam_pe_core] refine gapped alignments... 0.98 sec

[bwa_sai2sam_pe_core] print alignments... 2.23 sec

[bwa_sai2sam_pe_core] 155451392 sequences have been processed.

[bwa_sai2sam_pe_core] convert to sequence coordinate...

[infer_isize] (25, 50, 75) percentile: (453, 463, 473)

[infer_isize] low and high boundaries: 413 and 513 for estimating avg and std

[infer_isize] inferred external isize from 205739 pairs: 463.266 +/- 13.960

[infer_isize] skewness: 0.002; kurtosis: -0.145; ap_prior: 1.86e-05

[infer_isize] inferred maximum insert size: 560 (6.96 sigma)

[bwa_sai2sam_pe_core] time elapses: 32.50 sec

[bwa_sai2sam_pe_core] changing coordinates of 6131 alignments.

[bwa_sai2sam_pe_core] align unmapped mate...

[bwa_paired_sw] 9339 out of 9816 Q17 singletons are mated.

[bwa_paired_sw] 891 out of 1499 Q17 discordant pairs are fixed.

[bwa_sai2sam_pe_core] time elapses: 4.47 sec

[bwa_sai2sam_pe_core] refine gapped alignments... 0.99 sec

[bwa_sai2sam_pe_core] print alignments... 2.24 sec

[bwa_sai2sam_pe_core] 155713536 sequences have been processed.

[bwa_sai2sam_pe_core] convert to sequence coordinate...

[infer_isize] (25, 50, 75) percentile: (453, 463, 473)

[infer_isize] low and high boundaries: 413 and 513 for estimating avg and std

[infer_isize] inferred external isize from 205488 pairs: 463.230 +/- 13.948

[infer_isize] skewness: 0.006; kurtosis: -0.110; ap_prior: 1.83e-05

[infer_isize] inferred maximum insert size: 560 (6.96 sigma)

[bwa_sai2sam_pe_core] time elapses: 32.39 sec

[bwa_sai2sam_pe_core] changing coordinates of 6179 alignments.

[bwa_sai2sam_pe_core] align unmapped mate...

[bwa_paired_sw] 9478 out of 10053 Q17 singletons are mated.

[bwa_paired_sw] 895 out of 1496 Q17 discordant pairs are fixed.

[bwa_sai2sam_pe_core] time elapses: 4.57 sec

[bwa_sai2sam_pe_core] refine gapped alignments... 1.01 sec

[bwa_sai2sam_pe_core] print alignments... 2.27 sec

[bwa_sai2sam_pe_core] 155975680 sequences have been processed.

[bwa_sai2sam_pe_core] convert to sequence coordinate...

[infer_isize] (25, 50, 75) percentile: (453, 463, 473)

[infer_isize] low and high boundaries: 413 and 513 for estimating avg and std

[infer_isize] inferred external isize from 206023 pairs: 463.213 +/- 13.946

[infer_isize] skewness: -0.006; kurtosis: -0.120; ap_prior: 1.76e-05

[infer_isize] inferred maximum insert size: 560 (6.96 sigma)

[bwa_sai2sam_pe_core] time elapses: 31.30 sec

[bwa_sai2sam_pe_core] changing coordinates of 5945 alignments.

[bwa_sai2sam_pe_core] align unmapped mate...

[bwa_paired_sw] 9417 out of 9987 Q17 singletons are mated.

[bwa_paired_sw] 881 out of 1497 Q17 discordant pairs are fixed.

[bwa_sai2sam_pe_core] time elapses: 4.24 sec

[bwa_sai2sam_pe_core] refine gapped alignments... 0.97 sec

[bwa_sai2sam_pe_core] print alignments... 2.21 sec

[bwa_sai2sam_pe_core] 156237824 sequences have been processed.

[bwa_sai2sam_pe_core] convert to sequence coordinate...

[infer_isize] (25, 50, 75) percentile: (453, 463, 473)

[infer_isize] low and high boundaries: 413 and 513 for estimating avg and std

[infer_isize] inferred external isize from 205585 pairs: 463.229 +/- 13.948

[infer_isize] skewness: -0.002; kurtosis: -0.154; ap_prior: 1.73e-05

[infer_isize] inferred maximum insert size: 560 (6.96 sigma)

[bwa_sai2sam_pe_core] time elapses: 30.02 sec

[bwa_sai2sam_pe_core] changing coordinates of 6153 alignments.

[bwa_sai2sam_pe_core] align unmapped mate...

[bwa_paired_sw] 9651 out of 10192 Q17 singletons are mated.

[bwa_paired_sw] 890 out of 1427 Q17 discordant pairs are fixed.

[bwa_sai2sam_pe_core] time elapses: 4.28 sec

[bwa_sai2sam_pe_core] refine gapped alignments... 0.93 sec

[bwa_sai2sam_pe_core] print alignments... 2.17 sec

[bwa_sai2sam_pe_core] 156499968 sequences have been processed.

[bwa_sai2sam_pe_core] convert to sequence coordinate...

[infer_isize] (25, 50, 75) percentile: (453, 463, 473)

[infer_isize] low and high boundaries: 413 and 513 for estimating avg and std

[infer_isize] inferred external isize from 205412 pairs: 463.201 +/- 13.940

[infer_isize] skewness: 0.006; kurtosis: -0.123; ap_prior: 1.80e-05

[infer_isize] inferred maximum insert size: 560 (6.96 sigma)

[bwa_sai2sam_pe_core] time elapses: 29.11 sec

[bwa_sai2sam_pe_core] changing coordinates of 6031 alignments.

[bwa_sai2sam_pe_core] align unmapped mate...

[bwa_paired_sw] 9782 out of 10332 Q17 singletons are mated.

[bwa_paired_sw] 890 out of 1494 Q17 discordant pairs are fixed.

[bwa_sai2sam_pe_core] time elapses: 4.35 sec

[bwa_sai2sam_pe_core] refine gapped alignments... 0.95 sec

[bwa_sai2sam_pe_core] print alignments... 2.21 sec

[bwa_sai2sam_pe_core] 156762112 sequences have been processed.

[bwa_sai2sam_pe_core] convert to sequence coordinate...

[infer_isize] (25, 50, 75) percentile: (453, 463, 473)

[infer_isize] low and high boundaries: 413 and 513 for estimating avg and std

[infer_isize] inferred external isize from 205412 pairs: 463.180 +/- 13.958

[infer_isize] skewness: -0.000; kurtosis: -0.121; ap_prior: 1.65e-05

[infer_isize] inferred maximum insert size: 560 (6.96 sigma)

[bwa_sai2sam_pe_core] time elapses: 37.10 sec

[bwa_sai2sam_pe_core] changing coordinates of 5999 alignments.

[bwa_sai2sam_pe_core] align unmapped mate...

[bwa_paired_sw] 9751 out of 10296 Q17 singletons are mated.

[bwa_paired_sw] 902 out of 1465 Q17 discordant pairs are fixed.

[bwa_sai2sam_pe_core] time elapses: 4.42 sec

[bwa_sai2sam_pe_core] refine gapped alignments... 0.96 sec

[bwa_sai2sam_pe_core] print alignments... 2.19 sec

[bwa_sai2sam_pe_core] 157024256 sequences have been processed.

[bwa_sai2sam_pe_core] convert to sequence coordinate...

[infer_isize] (25, 50, 75) percentile: (453, 463, 473)

[infer_isize] low and high boundaries: 413 and 513 for estimating avg and std

[infer_isize] inferred external isize from 205492 pairs: 463.240 +/- 13.909

[infer_isize] skewness: 0.010; kurtosis: -0.128; ap_prior: 1.73e-05

[infer_isize] inferred maximum insert size: 560 (6.96 sigma)

[bwa_sai2sam_pe_core] time elapses: 64.47 sec

[bwa_sai2sam_pe_core] changing coordinates of 5966 alignments.

[bwa_sai2sam_pe_core] align unmapped mate...

[bwa_paired_sw] 9764 out of 10305 Q17 singletons are mated.

[bwa_paired_sw] 879 out of 1496 Q17 discordant pairs are fixed.

[bwa_sai2sam_pe_core] time elapses: 4.57 sec

[bwa_sai2sam_pe_core] refine gapped alignments... 1.00 sec

[bwa_sai2sam_pe_core] print alignments... 2.22 sec

[bwa_sai2sam_pe_core] 157286400 sequences have been processed.

[bwa_sai2sam_pe_core] convert to sequence coordinate...

[infer_isize] (25, 50, 75) percentile: (453, 463, 473)

[infer_isize] low and high boundaries: 413 and 513 for estimating avg and std

[infer_isize] inferred external isize from 205103 pairs: 463.218 +/- 13.945

[infer_isize] skewness: 0.001; kurtosis: -0.127; ap_prior: 1.75e-05

[infer_isize] inferred maximum insert size: 560 (6.96 sigma)

[bwa_sai2sam_pe_core] time elapses: 35.95 sec

[bwa_sai2sam_pe_core] changing coordinates of 6082 alignments.

[bwa_sai2sam_pe_core] align unmapped mate...

[bwa_paired_sw] 9941 out of 10466 Q17 singletons are mated.

[bwa_paired_sw] 824 out of 1452 Q17 discordant pairs are fixed.

[bwa_sai2sam_pe_core] time elapses: 4.58 sec

[bwa_sai2sam_pe_core] refine gapped alignments... 1.02 sec

[bwa_sai2sam_pe_core] print alignments... 2.20 sec

[bwa_sai2sam_pe_core] 157548544 sequences have been processed.

[bwa_sai2sam_pe_core] convert to sequence coordinate...

[infer_isize] (25, 50, 75) percentile: (453, 463, 473)

[infer_isize] low and high boundaries: 413 and 513 for estimating avg and std

[infer_isize] inferred external isize from 205250 pairs: 463.129 +/- 13.941

[infer_isize] skewness: 0.000; kurtosis: -0.133; ap_prior: 1.77e-05

[infer_isize] inferred maximum insert size: 560 (6.96 sigma)

[bwa_sai2sam_pe_core] time elapses: 32.01 sec

[bwa_sai2sam_pe_core] changing coordinates of 6036 alignments.

[bwa_sai2sam_pe_core] align unmapped mate...

[bwa_paired_sw] 9697 out of 10244 Q17 singletons are mated.

[bwa_paired_sw] 898 out of 1530 Q17 discordant pairs are fixed.

[bwa_sai2sam_pe_core] time elapses: 4.60 sec

[bwa_sai2sam_pe_core] refine gapped alignments... 1.04 sec

[bwa_sai2sam_pe_core] print alignments... 2.17 sec

[bwa_sai2sam_pe_core] 157810688 sequences have been processed.

[bwa_sai2sam_pe_core] convert to sequence coordinate...

[infer_isize] (25, 50, 75) percentile: (453, 463, 473)

[infer_isize] low and high boundaries: 413 and 513 for estimating avg and std

[infer_isize] inferred external isize from 205492 pairs: 463.180 +/- 13.982

[infer_isize] skewness: 0.004; kurtosis: -0.115; ap_prior: 1.82e-05

[infer_isize] inferred maximum insert size: 560 (6.96 sigma)

[bwa_sai2sam_pe_core] time elapses: 30.90 sec

[bwa_sai2sam_pe_core] changing coordinates of 5892 alignments.

[bwa_sai2sam_pe_core] align unmapped mate...

[bwa_paired_sw] 9866 out of 10384 Q17 singletons are mated.

[bwa_paired_sw] 899 out of 1517 Q17 discordant pairs are fixed.

[bwa_sai2sam_pe_core] time elapses: 4.48 sec

[bwa_sai2sam_pe_core] refine gapped alignments... 0.99 sec

[bwa_sai2sam_pe_core] print alignments... 2.18 sec

[bwa_sai2sam_pe_core] 158072832 sequences have been processed.

[bwa_sai2sam_pe_core] convert to sequence coordinate...

[infer_isize] (25, 50, 75) percentile: (453, 463, 473)

[infer_isize] low and high boundaries: 413 and 513 for estimating avg and std

[infer_isize] inferred external isize from 205536 pairs: 463.242 +/- 13.940

[infer_isize] skewness: 0.004; kurtosis: -0.128; ap_prior: 1.93e-05

[infer_isize] inferred maximum insert size: 560 (6.96 sigma)

[bwa_sai2sam_pe_core] time elapses: 30.29 sec

[bwa_sai2sam_pe_core] changing coordinates of 6095 alignments.

[bwa_sai2sam_pe_core] align unmapped mate...

[bwa_paired_sw] 9569 out of 10100 Q17 singletons are mated.

[bwa_paired_sw] 938 out of 1546 Q17 discordant pairs are fixed.

[bwa_sai2sam_pe_core] time elapses: 4.42 sec

[bwa_sai2sam_pe_core] refine gapped alignments... 0.99 sec

[bwa_sai2sam_pe_core] print alignments... 2.13 sec

[bwa_sai2sam_pe_core] 158334976 sequences have been processed.

[bwa_sai2sam_pe_core] convert to sequence coordinate...

[infer_isize] (25, 50, 75) percentile: (453, 463, 473)

[infer_isize] low and high boundaries: 413 and 513 for estimating avg and std

[infer_isize] inferred external isize from 205072 pairs: 463.151 +/- 13.959

[infer_isize] skewness: 0.016; kurtosis: -0.140; ap_prior: 1.74e-05

[infer_isize] inferred maximum insert size: 560 (6.96 sigma)

[bwa_sai2sam_pe_core] time elapses: 30.93 sec

[bwa_sai2sam_pe_core] changing coordinates of 6070 alignments.

[bwa_sai2sam_pe_core] align unmapped mate...

[bwa_paired_sw] 9738 out of 10329 Q17 singletons are mated.

[bwa_paired_sw] 854 out of 1506 Q17 discordant pairs are fixed.

[bwa_sai2sam_pe_core] time elapses: 4.44 sec

[bwa_sai2sam_pe_core] refine gapped alignments... 0.95 sec

[bwa_sai2sam_pe_core] print alignments... 2.08 sec

[bwa_sai2sam_pe_core] 158597120 sequences have been processed.

[bwa_sai2sam_pe_core] convert to sequence coordinate...

[infer_isize] (25, 50, 75) percentile: (453, 463, 473)

[infer_isize] low and high boundaries: 413 and 513 for estimating avg and std

[infer_isize] inferred external isize from 205261 pairs: 463.163 +/- 13.921

[infer_isize] skewness: -0.001; kurtosis: -0.131; ap_prior: 1.78e-05

[infer_isize] inferred maximum insert size: 560 (6.96 sigma)

[bwa_sai2sam_pe_core] time elapses: 31.15 sec

[bwa_sai2sam_pe_core] changing coordinates of 6113 alignments.

[bwa_sai2sam_pe_core] align unmapped mate...

[bwa_paired_sw] 9786 out of 10326 Q17 singletons are mated.

[bwa_paired_sw] 903 out of 1503 Q17 discordant pairs are fixed.

[bwa_sai2sam_pe_core] time elapses: 4.44 sec

[bwa_sai2sam_pe_core] refine gapped alignments... 0.93 sec

[bwa_sai2sam_pe_core] print alignments... 2.06 sec

[bwa_sai2sam_pe_core] 158859264 sequences have been processed.

[bwa_sai2sam_pe_core] convert to sequence coordinate...

[infer_isize] (25, 50, 75) percentile: (453, 463, 473)

[infer_isize] low and high boundaries: 413 and 513 for estimating avg and std

[infer_isize] inferred external isize from 205280 pairs: 463.186 +/- 13.946

[infer_isize] skewness: 0.001; kurtosis: -0.128; ap_prior: 1.73e-05

[infer_isize] inferred maximum insert size: 560 (6.96 sigma)

[bwa_sai2sam_pe_core] time elapses: 31.05 sec

[bwa_sai2sam_pe_core] changing coordinates of 5976 alignments.

[bwa_sai2sam_pe_core] align unmapped mate...

[bwa_paired_sw] 9903 out of 10439 Q17 singletons are mated.

[bwa_paired_sw] 851 out of 1449 Q17 discordant pairs are fixed.

[bwa_sai2sam_pe_core] time elapses: 4.46 sec

[bwa_sai2sam_pe_core] refine gapped alignments... 0.91 sec

[bwa_sai2sam_pe_core] print alignments... 2.07 sec

[bwa_sai2sam_pe_core] 159121408 sequences have been processed.

[bwa_sai2sam_pe_core] convert to sequence coordinate...

[infer_isize] (25, 50, 75) percentile: (453, 463, 473)

[infer_isize] low and high boundaries: 413 and 513 for estimating avg and std

[infer_isize] inferred external isize from 204985 pairs: 463.157 +/- 13.957

[infer_isize] skewness: -0.000; kurtosis: -0.137; ap_prior: 1.68e-05

[infer_isize] inferred maximum insert size: 560 (6.96 sigma)

[bwa_sai2sam_pe_core] time elapses: 30.85 sec

[bwa_sai2sam_pe_core] changing coordinates of 6033 alignments.

[bwa_sai2sam_pe_core] align unmapped mate...

[bwa_paired_sw] 10150 out of 10718 Q17 singletons are mated.

[bwa_paired_sw] 912 out of 1541 Q17 discordant pairs are fixed.

[bwa_sai2sam_pe_core] time elapses: 4.57 sec

[bwa_sai2sam_pe_core] refine gapped alignments... 0.95 sec

[bwa_sai2sam_pe_core] print alignments... 2.05 sec

[bwa_sai2sam_pe_core] 159383552 sequences have been processed.

[bwa_sai2sam_pe_core] convert to sequence coordinate...

[infer_isize] (25, 50, 75) percentile: (453, 463, 473)

[infer_isize] low and high boundaries: 413 and 513 for estimating avg and std

[infer_isize] inferred external isize from 204768 pairs: 463.180 +/- 13.935

[infer_isize] skewness: 0.004; kurtosis: -0.144; ap_prior: 1.79e-05

[infer_isize] inferred maximum insert size: 560 (6.96 sigma)

[bwa_sai2sam_pe_core] time elapses: 31.28 sec

[bwa_sai2sam_pe_core] changing coordinates of 5982 alignments.

[bwa_sai2sam_pe_core] align unmapped mate...

[bwa_paired_sw] 10151 out of 10691 Q17 singletons are mated.

[bwa_paired_sw] 861 out of 1422 Q17 discordant pairs are fixed.

[bwa_sai2sam_pe_core] time elapses: 4.51 sec

[bwa_sai2sam_pe_core] refine gapped alignments... 0.96 sec

[bwa_sai2sam_pe_core] print alignments... 2.13 sec

[bwa_sai2sam_pe_core] 159645696 sequences have been processed.

[bwa_sai2sam_pe_core] convert to sequence coordinate...

[infer_isize] (25, 50, 75) percentile: (453, 463, 473)

[infer_isize] low and high boundaries: 413 and 513 for estimating avg and std

[infer_isize] inferred external isize from 205250 pairs: 463.185 +/- 13.940

[infer_isize] skewness: -0.008; kurtosis: -0.134; ap_prior: 1.82e-05

[infer_isize] inferred maximum insert size: 560 (6.96 sigma)

[bwa_sai2sam_pe_core] time elapses: 32.36 sec

[bwa_sai2sam_pe_core] changing coordinates of 5957 alignments.

[bwa_sai2sam_pe_core] align unmapped mate...

[bwa_paired_sw] 9868 out of 10408 Q17 singletons are mated.

[bwa_paired_sw] 947 out of 1547 Q17 discordant pairs are fixed.

[bwa_sai2sam_pe_core] time elapses: 4.63 sec

[bwa_sai2sam_pe_core] refine gapped alignments... 0.97 sec

[bwa_sai2sam_pe_core] print alignments... 2.24 sec

[bwa_sai2sam_pe_core] 159907840 sequences have been processed.

[bwa_sai2sam_pe_core] convert to sequence coordinate...

[infer_isize] (25, 50, 75) percentile: (453, 463, 473)

[infer_isize] low and high boundaries: 413 and 513 for estimating avg and std

[infer_isize] inferred external isize from 204408 pairs: 463.160 +/- 13.935

[infer_isize] skewness: -0.004; kurtosis: -0.124; ap_prior: 1.86e-05

[infer_isize] inferred maximum insert size: 560 (6.96 sigma)

[bwa_sai2sam_pe_core] time elapses: 32.21 sec

[bwa_sai2sam_pe_core] changing coordinates of 6075 alignments.

[bwa_sai2sam_pe_core] align unmapped mate...

[bwa_paired_sw] 10432 out of 11000 Q17 singletons are mated.

[bwa_paired_sw] 884 out of 1482 Q17 discordant pairs are fixed.

[bwa_sai2sam_pe_core] time elapses: 4.77 sec

[bwa_sai2sam_pe_core] refine gapped alignments... 1.03 sec

[bwa_sai2sam_pe_core] print alignments... 2.31 sec

[bwa_sai2sam_pe_core] 160169984 sequences have been processed.

[bwa_sai2sam_pe_core] convert to sequence coordinate...

[infer_isize] (25, 50, 75) percentile: (453, 463, 473)

[infer_isize] low and high boundaries: 413 and 513 for estimating avg and std

[infer_isize] inferred external isize from 204687 pairs: 463.146 +/- 13.911

[infer_isize] skewness: 0.001; kurtosis: -0.136; ap_prior: 1.92e-05

[infer_isize] inferred maximum insert size: 560 (6.96 sigma)

[bwa_sai2sam_pe_core] time elapses: 33.04 sec

[bwa_sai2sam_pe_core] changing coordinates of 5992 alignments.

[bwa_sai2sam_pe_core] align unmapped mate...

[bwa_paired_sw] 10001 out of 10575 Q17 singletons are mated.

[bwa_paired_sw] 925 out of 1556 Q17 discordant pairs are fixed.

[bwa_sai2sam_pe_core] time elapses: 4.72 sec

[bwa_sai2sam_pe_core] refine gapped alignments... 1.05 sec

[bwa_sai2sam_pe_core] print alignments... 2.28 sec

[bwa_sai2sam_pe_core] 160432128 sequences have been processed.

[bwa_sai2sam_pe_core] convert to sequence coordinate...

[infer_isize] (25, 50, 75) percentile: (453, 463, 473)

[infer_isize] low and high boundaries: 413 and 513 for estimating avg and std

[infer_isize] inferred external isize from 204707 pairs: 463.130 +/- 13.913

[infer_isize] skewness: 0.004; kurtosis: -0.123; ap_prior: 1.68e-05

[infer_isize] inferred maximum insert size: 560 (6.96 sigma)

[bwa_sai2sam_pe_core] time elapses: 40.96 sec

[bwa_sai2sam_pe_core] changing coordinates of 5949 alignments.

[bwa_sai2sam_pe_core] align unmapped mate...

[bwa_paired_sw] 10132 out of 10685 Q17 singletons are mated.

[bwa_paired_sw] 877 out of 1489 Q17 discordant pairs are fixed.

[bwa_sai2sam_pe_core] time elapses: 4.69 sec

[bwa_sai2sam_pe_core] refine gapped alignments... 1.06 sec

[bwa_sai2sam_pe_core] print alignments... 2.28 sec

[bwa_sai2sam_pe_core] 160694272 sequences have been processed.

[bwa_sai2sam_pe_core] convert to sequence coordinate...

[infer_isize] (25, 50, 75) percentile: (453, 463, 473)

[infer_isize] low and high boundaries: 413 and 513 for estimating avg and std

[infer_isize] inferred external isize from 204639 pairs: 463.142 +/- 13.947

[infer_isize] skewness: 0.006; kurtosis: -0.141; ap_prior: 1.84e-05

[infer_isize] inferred maximum insert size: 560 (6.96 sigma)

[bwa_sai2sam_pe_core] time elapses: 34.90 sec

[bwa_sai2sam_pe_core] changing coordinates of 5932 alignments.

[bwa_sai2sam_pe_core] align unmapped mate...

[bwa_paired_sw] 10094 out of 10632 Q17 singletons are mated.

[bwa_paired_sw] 899 out of 1531 Q17 discordant pairs are fixed.

[bwa_sai2sam_pe_core] time elapses: 4.67 sec

[bwa_sai2sam_pe_core] refine gapped alignments... 1.02 sec

[bwa_sai2sam_pe_core] print alignments... 2.24 sec

[bwa_sai2sam_pe_core] 160956416 sequences have been processed.

[bwa_sai2sam_pe_core] convert to sequence coordinate...

[infer_isize] (25, 50, 75) percentile: (453, 463, 473)

[infer_isize] low and high boundaries: 413 and 513 for estimating avg and std

[infer_isize] inferred external isize from 204618 pairs: 463.149 +/- 13.943

[infer_isize] skewness: 0.016; kurtosis: -0.152; ap_prior: 1.77e-05

[infer_isize] inferred maximum insert size: 560 (6.96 sigma)

[bwa_sai2sam_pe_core] time elapses: 31.93 sec

[bwa_sai2sam_pe_core] changing coordinates of 6016 alignments.

[bwa_sai2sam_pe_core] align unmapped mate...

[bwa_paired_sw] 10331 out of 10856 Q17 singletons are mated.

[bwa_paired_sw] 928 out of 1539 Q17 discordant pairs are fixed.

[bwa_sai2sam_pe_core] time elapses: 4.65 sec

[bwa_sai2sam_pe_core] refine gapped alignments... 0.95 sec

[bwa_sai2sam_pe_core] print alignments... 2.12 sec

[bwa_sai2sam_pe_core] 161218560 sequences have been processed.

[bwa_sai2sam_pe_core] convert to sequence coordinate...

[infer_isize] (25, 50, 75) percentile: (453, 463, 473)

[infer_isize] low and high boundaries: 413 and 513 for estimating avg and std

[infer_isize] inferred external isize from 204485 pairs: 463.103 +/- 13.948

[infer_isize] skewness: 0.002; kurtosis: -0.137; ap_prior: 1.69e-05

[infer_isize] inferred maximum insert size: 560 (6.96 sigma)

[bwa_sai2sam_pe_core] time elapses: 32.28 sec

[bwa_sai2sam_pe_core] changing coordinates of 6151 alignments.

[bwa_sai2sam_pe_core] align unmapped mate...

[bwa_paired_sw] 10346 out of 10914 Q17 singletons are mated.

[bwa_paired_sw] 888 out of 1490 Q17 discordant pairs are fixed.

[bwa_sai2sam_pe_core] time elapses: 4.72 sec

[bwa_sai2sam_pe_core] refine gapped alignments... 0.97 sec

[bwa_sai2sam_pe_core] print alignments... 2.08 sec

[bwa_sai2sam_pe_core] 161480704 sequences have been processed.

[bwa_sai2sam_pe_core] convert to sequence coordinate...

[infer_isize] (25, 50, 75) percentile: (453, 463, 473)

[infer_isize] low and high boundaries: 413 and 513 for estimating avg and std

[infer_isize] inferred external isize from 204562 pairs: 463.193 +/- 13.927

[infer_isize] skewness: -0.001; kurtosis: -0.138; ap_prior: 1.82e-05

[infer_isize] inferred maximum insert size: 560 (6.96 sigma)

[bwa_sai2sam_pe_core] time elapses: 31.72 sec

[bwa_sai2sam_pe_core] changing coordinates of 6105 alignments.

[bwa_sai2sam_pe_core] align unmapped mate...

[bwa_paired_sw] 10257 out of 10793 Q17 singletons are mated.

[bwa_paired_sw] 914 out of 1493 Q17 discordant pairs are fixed.

[bwa_sai2sam_pe_core] time elapses: 4.70 sec

[bwa_sai2sam_pe_core] refine gapped alignments... 0.98 sec

[bwa_sai2sam_pe_core] print alignments... 2.10 sec

[bwa_sai2sam_pe_core] 161742848 sequences have been processed.

[bwa_sai2sam_pe_core] convert to sequence coordinate...

[infer_isize] (25, 50, 75) percentile: (453, 463, 473)

[infer_isize] low and high boundaries: 413 and 513 for estimating avg and std

[infer_isize] inferred external isize from 204550 pairs: 463.118 +/- 13.956

[infer_isize] skewness: 0.005; kurtosis: -0.132; ap_prior: 1.76e-05

[infer_isize] inferred maximum insert size: 560 (6.96 sigma)

[bwa_sai2sam_pe_core] time elapses: 31.52 sec

[bwa_sai2sam_pe_core] changing coordinates of 5993 alignments.

[bwa_sai2sam_pe_core] align unmapped mate...

[bwa_paired_sw] 10256 out of 10796 Q17 singletons are mated.

[bwa_paired_sw] 946 out of 1565 Q17 discordant pairs are fixed.

[bwa_sai2sam_pe_core] time elapses: 4.72 sec

[bwa_sai2sam_pe_core] refine gapped alignments... 0.98 sec

[bwa_sai2sam_pe_core] print alignments... 2.07 sec

[bwa_sai2sam_pe_core] 162004992 sequences have been processed.

[bwa_sai2sam_pe_core] convert to sequence coordinate...

[infer_isize] (25, 50, 75) percentile: (453, 463, 473)

[infer_isize] low and high boundaries: 413 and 513 for estimating avg and std

[infer_isize] inferred external isize from 205253 pairs: 463.116 +/- 13.967

[infer_isize] skewness: 0.017; kurtosis: -0.145; ap_prior: 1.86e-05

[infer_isize] inferred maximum insert size: 560 (6.96 sigma)

[bwa_sai2sam_pe_core] time elapses: 31.09 sec

[bwa_sai2sam_pe_core] changing coordinates of 5980 alignments.

[bwa_sai2sam_pe_core] align unmapped mate...

[bwa_paired_sw] 10085 out of 10602 Q17 singletons are mated.

[bwa_paired_sw] 900 out of 1462 Q17 discordant pairs are fixed.

[bwa_sai2sam_pe_core] time elapses: 4.62 sec

[bwa_sai2sam_pe_core] refine gapped alignments... 0.97 sec

[bwa_sai2sam_pe_core] print alignments... 2.13 sec

[bwa_sai2sam_pe_core] 162267136 sequences have been processed.

[bwa_sai2sam_pe_core] convert to sequence coordinate...

[infer_isize] (25, 50, 75) percentile: (453, 463, 473)

[infer_isize] low and high boundaries: 413 and 513 for estimating avg and std

[infer_isize] inferred external isize from 204308 pairs: 463.094 +/- 13.914

[infer_isize] skewness: 0.010; kurtosis: -0.141; ap_prior: 1.83e-05

[infer_isize] inferred maximum insert size: 560 (6.96 sigma)

[bwa_sai2sam_pe_core] time elapses: 31.83 sec

[bwa_sai2sam_pe_core] changing coordinates of 6046 alignments.

[bwa_sai2sam_pe_core] align unmapped mate...

[bwa_paired_sw] 10431 out of 10976 Q17 singletons are mated.

[bwa_paired_sw] 908 out of 1505 Q17 discordant pairs are fixed.

[bwa_sai2sam_pe_core] time elapses: 4.63 sec

[bwa_sai2sam_pe_core] refine gapped alignments... 0.94 sec

[bwa_sai2sam_pe_core] print alignments... 2.14 sec

[bwa_sai2sam_pe_core] 162529280 sequences have been processed.

[bwa_sai2sam_pe_core] convert to sequence coordinate...

[infer_isize] (25, 50, 75) percentile: (453, 463, 473)

[infer_isize] low and high boundaries: 413 and 513 for estimating avg and std

[infer_isize] inferred external isize from 204200 pairs: 463.114 +/- 13.953

[infer_isize] skewness: 0.009; kurtosis: -0.140; ap_prior: 1.71e-05

[infer_isize] inferred maximum insert size: 560 (6.96 sigma)

[bwa_sai2sam_pe_core] time elapses: 31.16 sec

[bwa_sai2sam_pe_core] changing coordinates of 5801 alignments.

[bwa_sai2sam_pe_core] align unmapped mate...

[bwa_paired_sw] 10697 out of 11228 Q17 singletons are mated.

[bwa_paired_sw] 892 out of 1460 Q17 discordant pairs are fixed.

[bwa_sai2sam_pe_core] time elapses: 4.74 sec

[bwa_sai2sam_pe_core] refine gapped alignments... 0.98 sec

[bwa_sai2sam_pe_core] print alignments... 2.22 sec

[bwa_sai2sam_pe_core] 162791424 sequences have been processed.

[bwa_sai2sam_pe_core] convert to sequence coordinate...

[infer_isize] (25, 50, 75) percentile: (453, 463, 473)

[infer_isize] low and high boundaries: 413 and 513 for estimating avg and std

[infer_isize] inferred external isize from 204232 pairs: 463.080 +/- 13.938

[infer_isize] skewness: -0.002; kurtosis: -0.139; ap_prior: 1.65e-05

[infer_isize] inferred maximum insert size: 560 (6.96 sigma)

[bwa_sai2sam_pe_core] time elapses: 32.20 sec

[bwa_sai2sam_pe_core] changing coordinates of 6032 alignments.

[bwa_sai2sam_pe_core] align unmapped mate...

[bwa_paired_sw] 10637 out of 11200 Q17 singletons are mated.

[bwa_paired_sw] 919 out of 1498 Q17 discordant pairs are fixed.

[bwa_sai2sam_pe_core] time elapses: 4.82 sec

[bwa_sai2sam_pe_core] refine gapped alignments... 0.99 sec

[bwa_sai2sam_pe_core] print alignments... 2.22 sec

[bwa_sai2sam_pe_core] 163053568 sequences have been processed.

[bwa_sai2sam_pe_core] convert to sequence coordinate...

[infer_isize] (25, 50, 75) percentile: (453, 463, 473)

[infer_isize] low and high boundaries: 413 and 513 for estimating avg and std

[infer_isize] inferred external isize from 204068 pairs: 463.108 +/- 13.933

[infer_isize] skewness: 0.004; kurtosis: -0.131; ap_prior: 1.77e-05

[infer_isize] inferred maximum insert size: 560 (6.96 sigma)

[bwa_sai2sam_pe_core] time elapses: 32.81 sec

[bwa_sai2sam_pe_core] changing coordinates of 5959 alignments.

[bwa_sai2sam_pe_core] align unmapped mate...

[bwa_paired_sw] 10522 out of 11083 Q17 singletons are mated.

[bwa_paired_sw] 824 out of 1428 Q17 discordant pairs are fixed.

[bwa_sai2sam_pe_core] time elapses: 4.77 sec

[bwa_sai2sam_pe_core] refine gapped alignments... 1.04 sec

[bwa_sai2sam_pe_core] print alignments... 2.28 sec

[bwa_sai2sam_pe_core] 163315712 sequences have been processed.

[bwa_sai2sam_pe_core] convert to sequence coordinate...

[infer_isize] (25, 50, 75) percentile: (453, 463, 473)

[infer_isize] low and high boundaries: 413 and 513 for estimating avg and std

[infer_isize] inferred external isize from 204243 pairs: 463.086 +/- 13.915

[infer_isize] skewness: -0.005; kurtosis: -0.119; ap_prior: 1.68e-05

[infer_isize] inferred maximum insert size: 560 (6.96 sigma)

[bwa_sai2sam_pe_core] time elapses: 31.84 sec

[bwa_sai2sam_pe_core] changing coordinates of 5896 alignments.

[bwa_sai2sam_pe_core] align unmapped mate...

[bwa_paired_sw] 10853 out of 11355 Q17 singletons are mated.

[bwa_paired_sw] 887 out of 1451 Q17 discordant pairs are fixed.

[bwa_sai2sam_pe_core] time elapses: 4.87 sec

[bwa_sai2sam_pe_core] refine gapped alignments... 1.05 sec

[bwa_sai2sam_pe_core] print alignments... 2.29 sec

[bwa_sai2sam_pe_core] 163577856 sequences have been processed.

[bwa_sai2sam_pe_core] convert to sequence coordinate...

[infer_isize] (25, 50, 75) percentile: (453, 463, 473)

[infer_isize] low and high boundaries: 413 and 513 for estimating avg and std

[infer_isize] inferred external isize from 203988 pairs: 463.063 +/- 13.934

[infer_isize] skewness: 0.002; kurtosis: -0.122; ap_prior: 1.65e-05

[infer_isize] inferred maximum insert size: 560 (6.96 sigma)

[bwa_sai2sam_pe_core] time elapses: 32.06 sec

[bwa_sai2sam_pe_core] changing coordinates of 5884 alignments.

[bwa_sai2sam_pe_core] align unmapped mate...

[bwa_paired_sw] 10811 out of 11319 Q17 singletons are mated.

[bwa_paired_sw] 914 out of 1502 Q17 discordant pairs are fixed.

[bwa_sai2sam_pe_core] time elapses: 4.88 sec

[bwa_sai2sam_pe_core] refine gapped alignments... 1.08 sec

[bwa_sai2sam_pe_core] print alignments... 2.31 sec

[bwa_sai2sam_pe_core] 163840000 sequences have been processed.

[bwa_sai2sam_pe_core] convert to sequence coordinate...

[infer_isize] (25, 50, 75) percentile: (453, 463, 473)

[infer_isize] low and high boundaries: 413 and 513 for estimating avg and std

[infer_isize] inferred external isize from 203430 pairs: 463.173 +/- 13.961

[infer_isize] skewness: 0.012; kurtosis: -0.140; ap_prior: 1.79e-05

[infer_isize] inferred maximum insert size: 560 (6.96 sigma)

[bwa_sai2sam_pe_core] time elapses: 30.55 sec

[bwa_sai2sam_pe_core] changing coordinates of 6082 alignments.

[bwa_sai2sam_pe_core] align unmapped mate...

[bwa_paired_sw] 10757 out of 11339 Q17 singletons are mated.

[bwa_paired_sw] 955 out of 1516 Q17 discordant pairs are fixed.

[bwa_sai2sam_pe_core] time elapses: 4.74 sec

[bwa_sai2sam_pe_core] refine gapped alignments... 1.04 sec

[bwa_sai2sam_pe_core] print alignments... 2.27 sec

[bwa_sai2sam_pe_core] 164102144 sequences have been processed.

[bwa_sai2sam_pe_core] convert to sequence coordinate...

[infer_isize] (25, 50, 75) percentile: (453, 463, 473)

[infer_isize] low and high boundaries: 413 and 513 for estimating avg and std

[infer_isize] inferred external isize from 203343 pairs: 463.143 +/- 13.947

[infer_isize] skewness: 0.003; kurtosis: -0.105; ap_prior: 1.82e-05

[infer_isize] inferred maximum insert size: 560 (6.96 sigma)

[bwa_sai2sam_pe_core] time elapses: 30.71 sec

[bwa_sai2sam_pe_core] changing coordinates of 6156 alignments.

[bwa_sai2sam_pe_core] align unmapped mate...

[bwa_paired_sw] 11130 out of 11691 Q17 singletons are mated.

[bwa_paired_sw] 854 out of 1461 Q17 discordant pairs are fixed.

[bwa_sai2sam_pe_core] time elapses: 4.72 sec

[bwa_sai2sam_pe_core] refine gapped alignments... 0.95 sec

[bwa_sai2sam_pe_core] print alignments... 2.11 sec

[bwa_sai2sam_pe_core] 164364288 sequences have been processed.

[bwa_sai2sam_pe_core] convert to sequence coordinate...

[infer_isize] (25, 50, 75) percentile: (453, 463, 473)

[infer_isize] low and high boundaries: 413 and 513 for estimating avg and std

[infer_isize] inferred external isize from 203685 pairs: 463.063 +/- 13.956

[infer_isize] skewness: 0.002; kurtosis: -0.126; ap_prior: 1.82e-05

[infer_isize] inferred maximum insert size: 560 (6.96 sigma)

[bwa_sai2sam_pe_core] time elapses: 29.94 sec

[bwa_sai2sam_pe_core] changing coordinates of 6065 alignments.

[bwa_sai2sam_pe_core] align unmapped mate...

[bwa_paired_sw] 11065 out of 11599 Q17 singletons are mated.

[bwa_paired_sw] 849 out of 1429 Q17 discordant pairs are fixed.

[bwa_sai2sam_pe_core] time elapses: 4.68 sec

[bwa_sai2sam_pe_core] refine gapped alignments... 0.94 sec

[bwa_sai2sam_pe_core] print alignments... 2.16 sec

[bwa_sai2sam_pe_core] 164626432 sequences have been processed.

[bwa_sai2sam_pe_core] convert to sequence coordinate...

[infer_isize] (25, 50, 75) percentile: (453, 463, 473)

[infer_isize] low and high boundaries: 413 and 513 for estimating avg and std

[infer_isize] inferred external isize from 203682 pairs: 463.062 +/- 13.975

[infer_isize] skewness: 0.001; kurtosis: -0.136; ap_prior: 1.78e-05

[infer_isize] inferred maximum insert size: 560 (6.96 sigma)

[bwa_sai2sam_pe_core] time elapses: 29.98 sec

[bwa_sai2sam_pe_core] changing coordinates of 6026 alignments.

[bwa_sai2sam_pe_core] align unmapped mate...

[bwa_paired_sw] 10956 out of 11501 Q17 singletons are mated.

[bwa_paired_sw] 851 out of 1422 Q17 discordant pairs are fixed.

[bwa_sai2sam_pe_core] time elapses: 4.66 sec

[bwa_sai2sam_pe_core] refine gapped alignments... 0.94 sec

[bwa_sai2sam_pe_core] print alignments... 2.20 sec

[bwa_sai2sam_pe_core] 164888576 sequences have been processed.

[bwa_sai2sam_pe_core] convert to sequence coordinate...

[infer_isize] (25, 50, 75) percentile: (453, 463, 473)

[infer_isize] low and high boundaries: 413 and 513 for estimating avg and std

[infer_isize] inferred external isize from 203551 pairs: 463.098 +/- 13.973

[infer_isize] skewness: 0.012; kurtosis: -0.133; ap_prior: 1.61e-05

[infer_isize] inferred maximum insert size: 560 (6.96 sigma)

[bwa_sai2sam_pe_core] time elapses: 30.28 sec

[bwa_sai2sam_pe_core] changing coordinates of 5844 alignments.

[bwa_sai2sam_pe_core] align unmapped mate...

[bwa_paired_sw] 11109 out of 11678 Q17 singletons are mated.

[bwa_paired_sw] 854 out of 1408 Q17 discordant pairs are fixed.

[bwa_sai2sam_pe_core] time elapses: 4.66 sec

[bwa_sai2sam_pe_core] refine gapped alignments... 0.95 sec

[bwa_sai2sam_pe_core] print alignments... 2.18 sec

[bwa_sai2sam_pe_core] 165150720 sequences have been processed.

[bwa_sai2sam_pe_core] convert to sequence coordinate...

[infer_isize] (25, 50, 75) percentile: (453, 463, 473)

[infer_isize] low and high boundaries: 413 and 513 for estimating avg and std

[infer_isize] inferred external isize from 203253 pairs: 463.077 +/- 13.948

[infer_isize] skewness: 0.005; kurtosis: -0.147; ap_prior: 1.85e-05

[infer_isize] inferred maximum insert size: 560 (6.96 sigma)

[bwa_sai2sam_pe_core] time elapses: 30.30 sec

[bwa_sai2sam_pe_core] changing coordinates of 5999 alignments.

[bwa_sai2sam_pe_core] align unmapped mate...

[bwa_paired_sw] 11176 out of 11704 Q17 singletons are mated.

[bwa_paired_sw] 889 out of 1464 Q17 discordant pairs are fixed.

[bwa_sai2sam_pe_core] time elapses: 4.72 sec

[bwa_sai2sam_pe_core] refine gapped alignments... 0.94 sec

[bwa_sai2sam_pe_core] print alignments... 2.16 sec

[bwa_sai2sam_pe_core] 165412864 sequences have been processed.

[bwa_sai2sam_pe_core] convert to sequence coordinate...

[infer_isize] (25, 50, 75) percentile: (453, 463, 473)

[infer_isize] low and high boundaries: 413 and 513 for estimating avg and std

[infer_isize] inferred external isize from 203167 pairs: 463.082 +/- 13.957

[infer_isize] skewness: 0.002; kurtosis: -0.140; ap_prior: 1.84e-05

[infer_isize] inferred maximum insert size: 560 (6.96 sigma)

[bwa_sai2sam_pe_core] time elapses: 30.22 sec

[bwa_sai2sam_pe_core] changing coordinates of 6042 alignments.

[bwa_sai2sam_pe_core] align unmapped mate...

[bwa_paired_sw] 11118 out of 11693 Q17 singletons are mated.

[bwa_paired_sw] 877 out of 1511 Q17 discordant pairs are fixed.

[bwa_sai2sam_pe_core] time elapses: 4.71 sec

[bwa_sai2sam_pe_core] refine gapped alignments... 0.95 sec

[bwa_sai2sam_pe_core] print alignments... 2.19 sec

[bwa_sai2sam_pe_core] 165675008 sequences have been processed.

[bwa_sai2sam_pe_core] convert to sequence coordinate...

[infer_isize] (25, 50, 75) percentile: (453, 463, 473)

[infer_isize] low and high boundaries: 413 and 513 for estimating avg and std

[infer_isize] inferred external isize from 202594 pairs: 463.044 +/- 13.964

[infer_isize] skewness: 0.007; kurtosis: -0.128; ap_prior: 1.78e-05

[infer_isize] inferred maximum insert size: 560 (6.96 sigma)

[bwa_sai2sam_pe_core] time elapses: 35.98 sec

[bwa_sai2sam_pe_core] changing coordinates of 5988 alignments.

[bwa_sai2sam_pe_core] align unmapped mate...

[bwa_paired_sw] 11573 out of 12181 Q17 singletons are mated.

[bwa_paired_sw] 930 out of 1573 Q17 discordant pairs are fixed.

[bwa_sai2sam_pe_core] time elapses: 5.11 sec

[bwa_sai2sam_pe_core] refine gapped alignments... 1.03 sec

[bwa_sai2sam_pe_core] print alignments... 2.21 sec

[bwa_sai2sam_pe_core] 165937152 sequences have been processed.

[bwa_sai2sam_pe_core] convert to sequence coordinate...

[infer_isize] (25, 50, 75) percentile: (453, 463, 473)

[infer_isize] low and high boundaries: 413 and 513 for estimating avg and std

[infer_isize] inferred external isize from 203303 pairs: 463.116 +/- 13.988

[infer_isize] skewness: 0.003; kurtosis: -0.131; ap_prior: 1.74e-05

[infer_isize] inferred maximum insert size: 560 (6.96 sigma)

[bwa_sai2sam_pe_core] time elapses: 41.33 sec

[bwa_sai2sam_pe_core] changing coordinates of 5943 alignments.

[bwa_sai2sam_pe_core] align unmapped mate...

[bwa_paired_sw] 11426 out of 12003 Q17 singletons are mated.

[bwa_paired_sw] 853 out of 1446 Q17 discordant pairs are fixed.

[bwa_sai2sam_pe_core] time elapses: 5.03 sec

[bwa_sai2sam_pe_core] refine gapped alignments... 1.04 sec

[bwa_sai2sam_pe_core] print alignments... 2.33 sec

[bwa_sai2sam_pe_core] 166199296 sequences have been processed.

[bwa_sai2sam_pe_core] convert to sequence coordinate...

[infer_isize] (25, 50, 75) percentile: (453, 463, 473)

[infer_isize] low and high boundaries: 413 and 513 for estimating avg and std

[infer_isize] inferred external isize from 203234 pairs: 463.065 +/- 13.967

[infer_isize] skewness: 0.008; kurtosis: -0.129; ap_prior: 1.69e-05

[infer_isize] inferred maximum insert size: 560 (6.96 sigma)

[bwa_sai2sam_pe_core] time elapses: 32.31 sec

[bwa_sai2sam_pe_core] changing coordinates of 6045 alignments.

[bwa_sai2sam_pe_core] align unmapped mate...

[bwa_paired_sw] 11306 out of 11887 Q17 singletons are mated.

[bwa_paired_sw] 867 out of 1442 Q17 discordant pairs are fixed.

[bwa_sai2sam_pe_core] time elapses: 5.01 sec

[bwa_sai2sam_pe_core] refine gapped alignments... 1.05 sec

[bwa_sai2sam_pe_core] print alignments... 2.30 sec

[bwa_sai2sam_pe_core] 166461440 sequences have been processed.

[bwa_sai2sam_pe_core] convert to sequence coordinate...

[infer_isize] (25, 50, 75) percentile: (453, 463, 473)

[infer_isize] low and high boundaries: 413 and 513 for estimating avg and std

[infer_isize] inferred external isize from 203138 pairs: 463.113 +/- 13.965

[infer_isize] skewness: 0.012; kurtosis: -0.135; ap_prior: 1.82e-05

[infer_isize] inferred maximum insert size: 560 (6.96 sigma)

[bwa_sai2sam_pe_core] time elapses: 31.87 sec

[bwa_sai2sam_pe_core] changing coordinates of 5946 alignments.

[bwa_sai2sam_pe_core] align unmapped mate...

[bwa_paired_sw] 11549 out of 12088 Q17 singletons are mated.

[bwa_paired_sw] 976 out of 1562 Q17 discordant pairs are fixed.

[bwa_sai2sam_pe_core] time elapses: 4.99 sec

[bwa_sai2sam_pe_core] refine gapped alignments... 1.03 sec

[bwa_sai2sam_pe_core] print alignments... 2.23 sec

[bwa_sai2sam_pe_core] 166723584 sequences have been processed.

[bwa_sai2sam_pe_core] convert to sequence coordinate...

[infer_isize] (25, 50, 75) percentile: (453, 463, 473)

[infer_isize] low and high boundaries: 413 and 513 for estimating avg and std

[infer_isize] inferred external isize from 203620 pairs: 463.046 +/- 13.951

[infer_isize] skewness: -0.003; kurtosis: -0.148; ap_prior: 1.91e-05

[infer_isize] inferred maximum insert size: 560 (6.96 sigma)

[bwa_sai2sam_pe_core] time elapses: 30.80 sec

[bwa_sai2sam_pe_core] changing coordinates of 5999 alignments.

[bwa_sai2sam_pe_core] align unmapped mate...

[bwa_paired_sw] 11016 out of 11554 Q17 singletons are mated.

[bwa_paired_sw] 982 out of 1628 Q17 discordant pairs are fixed.

[bwa_sai2sam_pe_core] time elapses: 4.91 sec

[bwa_sai2sam_pe_core] refine gapped alignments... 0.99 sec

[bwa_sai2sam_pe_core] print alignments... 2.20 sec

[bwa_sai2sam_pe_core] 166985728 sequences have been processed.

[bwa_sai2sam_pe_core] convert to sequence coordinate...

[infer_isize] (25, 50, 75) percentile: (453, 463, 473)

[infer_isize] low and high boundaries: 413 and 513 for estimating avg and std

[infer_isize] inferred external isize from 202924 pairs: 463.055 +/- 13.957

[infer_isize] skewness: 0.008; kurtosis: -0.134; ap_prior: 1.62e-05

[infer_isize] inferred maximum insert size: 560 (6.96 sigma)

[bwa_sai2sam_pe_core] time elapses: 32.18 sec

[bwa_sai2sam_pe_core] changing coordinates of 5944 alignments.

[bwa_sai2sam_pe_core] align unmapped mate...

[bwa_paired_sw] 11487 out of 12071 Q17 singletons are mated.

[bwa_paired_sw] 908 out of 1469 Q17 discordant pairs are fixed.

[bwa_sai2sam_pe_core] time elapses: 5.01 sec

[bwa_sai2sam_pe_core] refine gapped alignments... 1.02 sec

[bwa_sai2sam_pe_core] print alignments... 2.25 sec

[bwa_sai2sam_pe_core] 167247872 sequences have been processed.

[bwa_sai2sam_pe_core] convert to sequence coordinate...

[infer_isize] (25, 50, 75) percentile: (453, 463, 473)

[infer_isize] low and high boundaries: 413 and 513 for estimating avg and std

[infer_isize] inferred external isize from 202860 pairs: 463.021 +/- 13.964

[infer_isize] skewness: -0.002; kurtosis: -0.136; ap_prior: 1.69e-05

[infer_isize] inferred maximum insert size: 560 (6.96 sigma)

[bwa_sai2sam_pe_core] time elapses: 31.06 sec

[bwa_sai2sam_pe_core] changing coordinates of 6015 alignments.

[bwa_sai2sam_pe_core] align unmapped mate...

[bwa_paired_sw] 11570 out of 12076 Q17 singletons are mated.

[bwa_paired_sw] 903 out of 1527 Q17 discordant pairs are fixed.

[bwa_sai2sam_pe_core] time elapses: 4.99 sec

[bwa_sai2sam_pe_core] refine gapped alignments... 1.02 sec

[bwa_sai2sam_pe_core] print alignments... 2.21 sec

[bwa_sai2sam_pe_core] 167510016 sequences have been processed.

[bwa_sai2sam_pe_core] convert to sequence coordinate...

[infer_isize] (25, 50, 75) percentile: (453, 463, 473)

[infer_isize] low and high boundaries: 413 and 513 for estimating avg and std

[infer_isize] inferred external isize from 202691 pairs: 463.067 +/- 13.953

[infer_isize] skewness: 0.004; kurtosis: -0.146; ap_prior: 1.72e-05

[infer_isize] inferred maximum insert size: 560 (6.96 sigma)

[bwa_sai2sam_pe_core] time elapses: 31.00 sec

[bwa_sai2sam_pe_core] changing coordinates of 6022 alignments.

[bwa_sai2sam_pe_core] align unmapped mate...

[bwa_paired_sw] 11800 out of 12338 Q17 singletons are mated.

[bwa_paired_sw] 881 out of 1478 Q17 discordant pairs are fixed.

[bwa_sai2sam_pe_core] time elapses: 5.04 sec

[bwa_sai2sam_pe_core] refine gapped alignments... 0.99 sec

[bwa_sai2sam_pe_core] print alignments... 2.19 sec

[bwa_sai2sam_pe_core] 167772160 sequences have been processed.

[bwa_sai2sam_pe_core] convert to sequence coordinate...

[infer_isize] (25, 50, 75) percentile: (453, 463, 473)

[infer_isize] low and high boundaries: 413 and 513 for estimating avg and std

[infer_isize] inferred external isize from 202602 pairs: 463.121 +/- 13.938

[infer_isize] skewness: 0.009; kurtosis: -0.120; ap_prior: 1.73e-05

[infer_isize] inferred maximum insert size: 560 (6.96 sigma)

[bwa_sai2sam_pe_core] time elapses: 30.80 sec

[bwa_sai2sam_pe_core] changing coordinates of 5948 alignments.

[bwa_sai2sam_pe_core] align unmapped mate...

[bwa_paired_sw] 11963 out of 12555 Q17 singletons are mated.

[bwa_paired_sw] 885 out of 1446 Q17 discordant pairs are fixed.

[bwa_sai2sam_pe_core] time elapses: 5.12 sec

[bwa_sai2sam_pe_core] refine gapped alignments... 1.00 sec

[bwa_sai2sam_pe_core] print alignments... 2.13 sec

[bwa_sai2sam_pe_core] 168034304 sequences have been processed.

[bwa_sai2sam_pe_core] convert to sequence coordinate...

[infer_isize] (25, 50, 75) percentile: (453, 463, 473)

[infer_isize] low and high boundaries: 413 and 513 for estimating avg and std

[infer_isize] inferred external isize from 202344 pairs: 463.005 +/- 13.948

[infer_isize] skewness: 0.007; kurtosis: -0.135; ap_prior: 1.66e-05

[infer_isize] inferred maximum insert size: 560 (6.96 sigma)

[bwa_sai2sam_pe_core] time elapses: 30.27 sec

[bwa_sai2sam_pe_core] changing coordinates of 5788 alignments.

[bwa_sai2sam_pe_core] align unmapped mate...

[bwa_paired_sw] 12627 out of 13143 Q17 singletons are mated.

[bwa_paired_sw] 853 out of 1447 Q17 discordant pairs are fixed.

[bwa_sai2sam_pe_core] time elapses: 5.24 sec

[bwa_sai2sam_pe_core] refine gapped alignments... 0.98 sec

[bwa_sai2sam_pe_core] print alignments... 2.09 sec

[bwa_sai2sam_pe_core] 168296448 sequences have been processed.

[bwa_sai2sam_pe_core] convert to sequence coordinate...

[infer_isize] (25, 50, 75) percentile: (453, 463, 473)

[infer_isize] low and high boundaries: 413 and 513 for estimating avg and std

[infer_isize] inferred external isize from 201341 pairs: 463.046 +/- 13.997

[infer_isize] skewness: 0.012; kurtosis: -0.136; ap_prior: 1.66e-05

[infer_isize] inferred maximum insert size: 560 (6.96 sigma)

[bwa_sai2sam_pe_core] time elapses: 30.41 sec

[bwa_sai2sam_pe_core] changing coordinates of 5917 alignments.

[bwa_sai2sam_pe_core] align unmapped mate...

[bwa_paired_sw] 12963 out of 13548 Q17 singletons are mated.

[bwa_paired_sw] 840 out of 1404 Q17 discordant pairs are fixed.

[bwa_sai2sam_pe_core] time elapses: 5.30 sec

[bwa_sai2sam_pe_core] refine gapped alignments... 0.98 sec

[bwa_sai2sam_pe_core] print alignments... 2.19 sec

[bwa_sai2sam_pe_core] 168558592 sequences have been processed.

[bwa_sai2sam_pe_core] convert to sequence coordinate...

[infer_isize] (25, 50, 75) percentile: (453, 463, 473)

[infer_isize] low and high boundaries: 413 and 513 for estimating avg and std

[infer_isize] inferred external isize from 201374 pairs: 463.062 +/- 13.964

[infer_isize] skewness: 0.012; kurtosis: -0.145; ap_prior: 1.67e-05

[infer_isize] inferred maximum insert size: 560 (6.96 sigma)

[bwa_sai2sam_pe_core] time elapses: 30.71 sec

[bwa_sai2sam_pe_core] changing coordinates of 5867 alignments.

[bwa_sai2sam_pe_core] align unmapped mate...

[bwa_paired_sw] 12728 out of 13312 Q17 singletons are mated.

[bwa_paired_sw] 914 out of 1467 Q17 discordant pairs are fixed.

[bwa_sai2sam_pe_core] time elapses: 5.31 sec

[bwa_sai2sam_pe_core] refine gapped alignments... 1.00 sec

[bwa_sai2sam_pe_core] print alignments... 2.16 sec

[bwa_sai2sam_pe_core] 168820736 sequences have been processed.

[bwa_sai2sam_pe_core] convert to sequence coordinate...

[infer_isize] (25, 50, 75) percentile: (453, 463, 473)

[infer_isize] low and high boundaries: 413 and 513 for estimating avg and std

[infer_isize] inferred external isize from 201142 pairs: 462.977 +/- 13.940

[infer_isize] skewness: 0.001; kurtosis: -0.129; ap_prior: 1.71e-05

[infer_isize] inferred maximum insert size: 560 (6.96 sigma)

[bwa_sai2sam_pe_core] time elapses: 33.87 sec

[bwa_sai2sam_pe_core] changing coordinates of 5982 alignments.

[bwa_sai2sam_pe_core] align unmapped mate...

[bwa_paired_sw] 13016 out of 13576 Q17 singletons are mated.

[bwa_paired_sw] 905 out of 1461 Q17 discordant pairs are fixed.

[bwa_sai2sam_pe_core] time elapses: 5.46 sec

[bwa_sai2sam_pe_core] refine gapped alignments... 1.09 sec

[bwa_sai2sam_pe_core] print alignments... 2.30 sec

[bwa_sai2sam_pe_core] 169082880 sequences have been processed.

[bwa_sai2sam_pe_core] convert to sequence coordinate...

[infer_isize] (25, 50, 75) percentile: (453, 463, 473)

[infer_isize] low and high boundaries: 413 and 513 for estimating avg and std

[infer_isize] inferred external isize from 201399 pairs: 462.976 +/- 13.955

[infer_isize] skewness: 0.005; kurtosis: -0.122; ap_prior: 1.92e-05

[infer_isize] inferred maximum insert size: 560 (6.96 sigma)

[bwa_sai2sam_pe_core] time elapses: 31.72 sec

[bwa_sai2sam_pe_core] changing coordinates of 5913 alignments.

[bwa_sai2sam_pe_core] align unmapped mate...

[bwa_paired_sw] 13122 out of 13677 Q17 singletons are mated.

[bwa_paired_sw] 897 out of 1440 Q17 discordant pairs are fixed.

[bwa_sai2sam_pe_core] time elapses: 5.57 sec

[bwa_sai2sam_pe_core] refine gapped alignments... 1.10 sec

[bwa_sai2sam_pe_core] print alignments... 2.27 sec

[bwa_sai2sam_pe_core] 169345024 sequences have been processed.

[bwa_sai2sam_pe_core] convert to sequence coordinate...

[infer_isize] (25, 50, 75) percentile: (453, 463, 473)

[infer_isize] low and high boundaries: 413 and 513 for estimating avg and std

[infer_isize] inferred external isize from 200451 pairs: 462.966 +/- 13.942

[infer_isize] skewness: 0.006; kurtosis: -0.123; ap_prior: 1.55e-05

[infer_isize] inferred maximum insert size: 560 (6.96 sigma)

[bwa_sai2sam_pe_core] time elapses: 32.65 sec

[bwa_sai2sam_pe_core] changing coordinates of 5989 alignments.

[bwa_sai2sam_pe_core] align unmapped mate...

[bwa_paired_sw] 13644 out of 14217 Q17 singletons are mated.

[bwa_paired_sw] 843 out of 1437 Q17 discordant pairs are fixed.

[bwa_sai2sam_pe_core] time elapses: 5.68 sec

[bwa_sai2sam_pe_core] refine gapped alignments... 1.13 sec

[bwa_sai2sam_pe_core] print alignments... 2.34 sec

[bwa_sai2sam_pe_core] 169607168 sequences have been processed.

[bwa_sai2sam_pe_core] convert to sequence coordinate...

[infer_isize] (25, 50, 75) percentile: (453, 463, 473)

[infer_isize] low and high boundaries: 413 and 513 for estimating avg and std

[infer_isize] inferred external isize from 200109 pairs: 462.933 +/- 13.934

[infer_isize] skewness: 0.003; kurtosis: -0.130; ap_prior: 1.64e-05

[infer_isize] inferred maximum insert size: 560 (6.96 sigma)

[bwa_sai2sam_pe_core] time elapses: 32.50 sec

[bwa_sai2sam_pe_core] changing coordinates of 5957 alignments.

[bwa_sai2sam_pe_core] align unmapped mate...

[bwa_paired_sw] 14011 out of 14582 Q17 singletons are mated.

[bwa_paired_sw] 837 out of 1386 Q17 discordant pairs are fixed.

[bwa_sai2sam_pe_core] time elapses: 5.78 sec

[bwa_sai2sam_pe_core] refine gapped alignments... 1.14 sec

[bwa_sai2sam_pe_core] print alignments... 2.25 sec

[bwa_sai2sam_pe_core] 169869312 sequences have been processed.

[bwa_sai2sam_pe_core] convert to sequence coordinate...

[infer_isize] (25, 50, 75) percentile: (453, 463, 473)

[infer_isize] low and high boundaries: 413 and 513 for estimating avg and std

[infer_isize] inferred external isize from 200015 pairs: 462.957 +/- 13.931

[infer_isize] skewness: 0.007; kurtosis: -0.136; ap_prior: 1.68e-05

[infer_isize] inferred maximum insert size: 560 (6.96 sigma)

[bwa_sai2sam_pe_core] time elapses: 32.36 sec

[bwa_sai2sam_pe_core] changing coordinates of 6089 alignments.

[bwa_sai2sam_pe_core] align unmapped mate...

[bwa_paired_sw] 13900 out of 14473 Q17 singletons are mated.

[bwa_paired_sw] 938 out of 1507 Q17 discordant pairs are fixed.

[bwa_sai2sam_pe_core] time elapses: 5.75 sec

[bwa_sai2sam_pe_core] refine gapped alignments... 1.11 sec

[bwa_sai2sam_pe_core] print alignments... 2.26 sec

[bwa_sai2sam_pe_core] 170131456 sequences have been processed.

[bwa_sai2sam_pe_core] convert to sequence coordinate...

[infer_isize] (25, 50, 75) percentile: (453, 463, 473)

[infer_isize] low and high boundaries: 413 and 513 for estimating avg and std

[infer_isize] inferred external isize from 199406 pairs: 462.948 +/- 13.941

[infer_isize] skewness: 0.009; kurtosis: -0.146; ap_prior: 1.99e-05

[infer_isize] inferred maximum insert size: 560 (6.96 sigma)

[bwa_sai2sam_pe_core] time elapses: 31.48 sec

[bwa_sai2sam_pe_core] changing coordinates of 5846 alignments.

[bwa_sai2sam_pe_core] align unmapped mate...

[bwa_paired_sw] 14980 out of 15511 Q17 singletons are mated.

[bwa_paired_sw] 890 out of 1409 Q17 discordant pairs are fixed.

[bwa_sai2sam_pe_core] time elapses: 5.97 sec

[bwa_sai2sam_pe_core] refine gapped alignments... 1.12 sec

[bwa_sai2sam_pe_core] print alignments... 2.23 sec

[bwa_sai2sam_pe_core] 170393600 sequences have been processed.

[bwa_sai2sam_pe_core] convert to sequence coordinate...

[infer_isize] (25, 50, 75) percentile: (453, 463, 473)

[infer_isize] low and high boundaries: 413 and 513 for estimating avg and std

[infer_isize] inferred external isize from 198063 pairs: 462.967 +/- 13.942

[infer_isize] skewness: 0.003; kurtosis: -0.126; ap_prior: 1.79e-05

[infer_isize] inferred maximum insert size: 560 (6.96 sigma)

[bwa_sai2sam_pe_core] time elapses: 30.69 sec

[bwa_sai2sam_pe_core] changing coordinates of 5765 alignments.

[bwa_sai2sam_pe_core] align unmapped mate...

[bwa_paired_sw] 15937 out of 16485 Q17 singletons are mated.

[bwa_paired_sw] 848 out of 1339 Q17 discordant pairs are fixed.

[bwa_sai2sam_pe_core] time elapses: 6.18 sec

[bwa_sai2sam_pe_core] refine gapped alignments... 1.14 sec

[bwa_sai2sam_pe_core] print alignments... 2.24 sec

[bwa_sai2sam_pe_core] 170655744 sequences have been processed.

[bwa_sai2sam_pe_core] convert to sequence coordinate...

[infer_isize] (25, 50, 75) percentile: (453, 463, 473)

[infer_isize] low and high boundaries: 413 and 513 for estimating avg and std

[infer_isize] inferred external isize from 199030 pairs: 463.010 +/- 13.931

[infer_isize] skewness: 0.006; kurtosis: -0.127; ap_prior: 1.65e-05

[infer_isize] inferred maximum insert size: 560 (6.96 sigma)

[bwa_sai2sam_pe_core] time elapses: 30.73 sec

[bwa_sai2sam_pe_core] changing coordinates of 5726 alignments.

[bwa_sai2sam_pe_core] align unmapped mate...

[bwa_paired_sw] 15213 out of 15768 Q17 singletons are mated.

[bwa_paired_sw] 914 out of 1459 Q17 discordant pairs are fixed.

[bwa_sai2sam_pe_core] time elapses: 6.04 sec

[bwa_sai2sam_pe_core] refine gapped alignments... 1.19 sec

[bwa_sai2sam_pe_core] print alignments... 2.23 sec

[bwa_sai2sam_pe_core] 170917888 sequences have been processed.

[bwa_sai2sam_pe_core] convert to sequence coordinate...

[infer_isize] (25, 50, 75) percentile: (453, 463, 473)

[infer_isize] low and high boundaries: 413 and 513 for estimating avg and std

[infer_isize] inferred external isize from 197865 pairs: 463.011 +/- 13.966

[infer_isize] skewness: 0.001; kurtosis: -0.138; ap_prior: 1.69e-05

[infer_isize] inferred maximum insert size: 560 (6.96 sigma)

[bwa_sai2sam_pe_core] time elapses: 31.40 sec

[bwa_sai2sam_pe_core] changing coordinates of 5721 alignments.

[bwa_sai2sam_pe_core] align unmapped mate...

[bwa_paired_sw] 16256 out of 16837 Q17 singletons are mated.

[bwa_paired_sw] 945 out of 1453 Q17 discordant pairs are fixed.

[bwa_sai2sam_pe_core] time elapses: 6.36 sec

[bwa_sai2sam_pe_core] refine gapped alignments... 1.20 sec

[bwa_sai2sam_pe_core] print alignments... 2.24 sec

[bwa_sai2sam_pe_core] 171180032 sequences have been processed.

[bwa_sai2sam_pe_core] convert to sequence coordinate...

[infer_isize] (25, 50, 75) percentile: (453, 463, 473)

[infer_isize] low and high boundaries: 413 and 513 for estimating avg and std

[infer_isize] inferred external isize from 197597 pairs: 462.940 +/- 13.894

[infer_isize] skewness: 0.004; kurtosis: -0.133; ap_prior: 1.64e-05

[infer_isize] inferred maximum insert size: 560 (6.96 sigma)

[bwa_sai2sam_pe_core] time elapses: 30.56 sec

[bwa_sai2sam_pe_core] changing coordinates of 5642 alignments.

[bwa_sai2sam_pe_core] align unmapped mate...

[bwa_paired_sw] 16573 out of 17160 Q17 singletons are mated.

[bwa_paired_sw] 860 out of 1392 Q17 discordant pairs are fixed.

[bwa_sai2sam_pe_core] time elapses: 6.40 sec

[bwa_sai2sam_pe_core] refine gapped alignments... 1.22 sec

[bwa_sai2sam_pe_core] print alignments... 2.22 sec

[bwa_sai2sam_pe_core] 171442176 sequences have been processed.

[bwa_sai2sam_pe_core] convert to sequence coordinate...

[infer_isize] (25, 50, 75) percentile: (453, 463, 473)

[infer_isize] low and high boundaries: 413 and 513 for estimating avg and std

[infer_isize] inferred external isize from 197005 pairs: 462.967 +/- 13.955

[infer_isize] skewness: -0.005; kurtosis: -0.124; ap_prior: 1.62e-05

[infer_isize] inferred maximum insert size: 560 (6.96 sigma)

[bwa_sai2sam_pe_core] time elapses: 31.01 sec

[bwa_sai2sam_pe_core] changing coordinates of 5812 alignments.

[bwa_sai2sam_pe_core] align unmapped mate...

[bwa_paired_sw] 17190 out of 17842 Q17 singletons are mated.

[bwa_paired_sw] 879 out of 1427 Q17 discordant pairs are fixed.

[bwa_sai2sam_pe_core] time elapses: 6.58 sec

[bwa_sai2sam_pe_core] refine gapped alignments... 1.23 sec

[bwa_sai2sam_pe_core] print alignments... 2.19 sec

[bwa_sai2sam_pe_core] 171704320 sequences have been processed.

[bwa_sai2sam_pe_core] convert to sequence coordinate...

[infer_isize] (25, 50, 75) percentile: (453, 463, 473)

[infer_isize] low and high boundaries: 413 and 513 for estimating avg and std

[infer_isize] inferred external isize from 195169 pairs: 462.884 +/- 13.887

[infer_isize] skewness: 0.007; kurtosis: -0.113; ap_prior: 1.69e-05

[infer_isize] inferred maximum insert size: 560 (6.96 sigma)

[bwa_sai2sam_pe_core] time elapses: 30.59 sec

[bwa_sai2sam_pe_core] changing coordinates of 5658 alignments.

[bwa_sai2sam_pe_core] align unmapped mate...

[bwa_paired_sw] 18336 out of 18966 Q17 singletons are mated.

[bwa_paired_sw] 893 out of 1409 Q17 discordant pairs are fixed.

[bwa_sai2sam_pe_core] time elapses: 6.93 sec

[bwa_sai2sam_pe_core] refine gapped alignments... 1.25 sec

[bwa_sai2sam_pe_core] print alignments... 2.22 sec

[bwa_sai2sam_pe_core] 171966464 sequences have been processed.

[bwa_sai2sam_pe_core] convert to sequence coordinate...

[infer_isize] (25, 50, 75) percentile: (453, 463, 472)

[infer_isize] low and high boundaries: 415 and 510 for estimating avg and std

[infer_isize] inferred external isize from 190555 pairs: 462.822 +/- 13.923

[infer_isize] skewness: 0.015; kurtosis: -0.171; ap_prior: 1.65e-05

[infer_isize] inferred maximum insert size: 560 (6.96 sigma)

[bwa_sai2sam_pe_core] time elapses: 30.62 sec

[bwa_sai2sam_pe_core] changing coordinates of 5609 alignments.

[bwa_sai2sam_pe_core] align unmapped mate...

[bwa_paired_sw] 22752 out of 23396 Q17 singletons are mated.

[bwa_paired_sw] 803 out of 1366 Q17 discordant pairs are fixed.

[bwa_sai2sam_pe_core] time elapses: 8.06 sec

[bwa_sai2sam_pe_core] refine gapped alignments... 1.26 sec

[bwa_sai2sam_pe_core] print alignments... 2.16 sec

[bwa_sai2sam_pe_core] 172228608 sequences have been processed.

[bwa_sai2sam_pe_core] convert to sequence coordinate...

[infer_isize] (25, 50, 75) percentile: (453, 463, 473)

[infer_isize] low and high boundaries: 413 and 513 for estimating avg and std

[infer_isize] inferred external isize from 194020 pairs: 462.868 +/- 13.924

[infer_isize] skewness: 0.001; kurtosis: -0.127; ap_prior: 1.51e-05

[infer_isize] inferred maximum insert size: 560 (6.96 sigma)

[bwa_sai2sam_pe_core] time elapses: 30.74 sec

[bwa_sai2sam_pe_core] changing coordinates of 5651 alignments.

[bwa_sai2sam_pe_core] align unmapped mate...

[bwa_paired_sw] 19692 out of 20308 Q17 singletons are mated.

[bwa_paired_sw] 879 out of 1395 Q17 discordant pairs are fixed.

[bwa_sai2sam_pe_core] time elapses: 7.31 sec

[bwa_sai2sam_pe_core] refine gapped alignments... 1.31 sec

[bwa_sai2sam_pe_core] print alignments... 2.28 sec

[bwa_sai2sam_pe_core] 172490752 sequences have been processed.

[bwa_sai2sam_pe_core] convert to sequence coordinate...

[infer_isize] (25, 50, 75) percentile: (453, 463, 473)

[infer_isize] low and high boundaries: 413 and 513 for estimating avg and std

[infer_isize] inferred external isize from 192353 pairs: 462.824 +/- 13.946

[infer_isize] skewness: 0.009; kurtosis: -0.135; ap_prior: 1.60e-05

[infer_isize] inferred maximum insert size: 560 (6.96 sigma)

[bwa_sai2sam_pe_core] time elapses: 30.98 sec

[bwa_sai2sam_pe_core] changing coordinates of 5728 alignments.

[bwa_sai2sam_pe_core] align unmapped mate...

[bwa_paired_sw] 21223 out of 21826 Q17 singletons are mated.

[bwa_paired_sw] 848 out of 1339 Q17 discordant pairs are fixed.

[bwa_sai2sam_pe_core] time elapses: 7.67 sec

[bwa_sai2sam_pe_core] refine gapped alignments... 1.33 sec

[bwa_sai2sam_pe_core] print alignments... 2.31 sec

[bwa_sai2sam_pe_core] 172752896 sequences have been processed.

[bwa_sai2sam_pe_core] convert to sequence coordinate...

[infer_isize] (25, 50, 75) percentile: (453, 462, 472)

[infer_isize] low and high boundaries: 415 and 510 for estimating avg and std

[infer_isize] inferred external isize from 84374 pairs: 462.794 +/- 13.876

[infer_isize] skewness: 0.018; kurtosis: -0.176; ap_prior: 1.62e-05

[infer_isize] inferred maximum insert size: 559 (6.96 sigma)

[bwa_sai2sam_pe_core] time elapses: 17.01 sec

[bwa_sai2sam_pe_core] changing coordinates of 2428 alignments.

[bwa_sai2sam_pe_core] align unmapped mate...

[bwa_paired_sw] 11252 out of 11535 Q17 singletons are mated.

[bwa_paired_sw] 378 out of 597 Q17 discordant pairs are fixed.

[bwa_sai2sam_pe_core] time elapses: 4.58 sec

[bwa_sai2sam_pe_core] refine gapped alignments... 0.57 sec

[bwa_sai2sam_pe_core] print alignments... 1.03 sec

[bwa_sai2sam_pe_core] 172870680 sequences have been processed.

[main] Version: 0.6.2-r126

[main] CMD: /sapfs/actrec/ngs_tools/bwa-0.6.2/bwa sampe -N 100 -f /sapfs/actrec/users/pratik/HPVDetector/siha_wgs/SRR1609142_1SRR1609142_2.sam /sapfs/actrec/users/pratik/HPVDetector/HPVDetector_v1.0/Human_HPV_index_files/human_HPV /sapfs/actrec/users/pratik/HPVDetector/siha_wgs/SRR1609142_1.sai /sapfs/actrec/users/pratik/HPVDetector/siha_wgs/SRR1609142_2.sai /sapfs/actrec/users/pratik/down/siha/SRR1609142_1.fastq /sapfs/actrec/users/pratik/down/siha/SRR1609142_2.fastq

[main] Real time: 29034.669 sec; CPU: 28850.519 sec

Please check /sapfs/actrec/users/pratik/HPVDetector/siha_wgs/SRR1609142_1SRR1609142_2_HPV_Annotated.txt file

Please check /sapfs/actrec/users/pratik/HPVDetector/siha_wgs/SRR1609142_1SRR1609142_2_HPV_HumanGene_Annotated.txt file

Please check /sapfs/actrec/users/pratik/HPVDetector/siha_wgs/SRR1609142_1SRR1609142_2_HPV_HumanGene_Annotated_Cyto.txt file
