# Supplementary material for: NGS-based approach to determine the presence of HPV and their sites of integration in human cancer genome
Source: Br J Cancer. 2015 May 14;112(12):1958–65. doi: 10.1038/bjc.2015.121 (PMC4580395; doi:10.1038/bjc.2015.121)
Supplement: Supplementary Material 2 [file bjc2015121x2.doc]

**
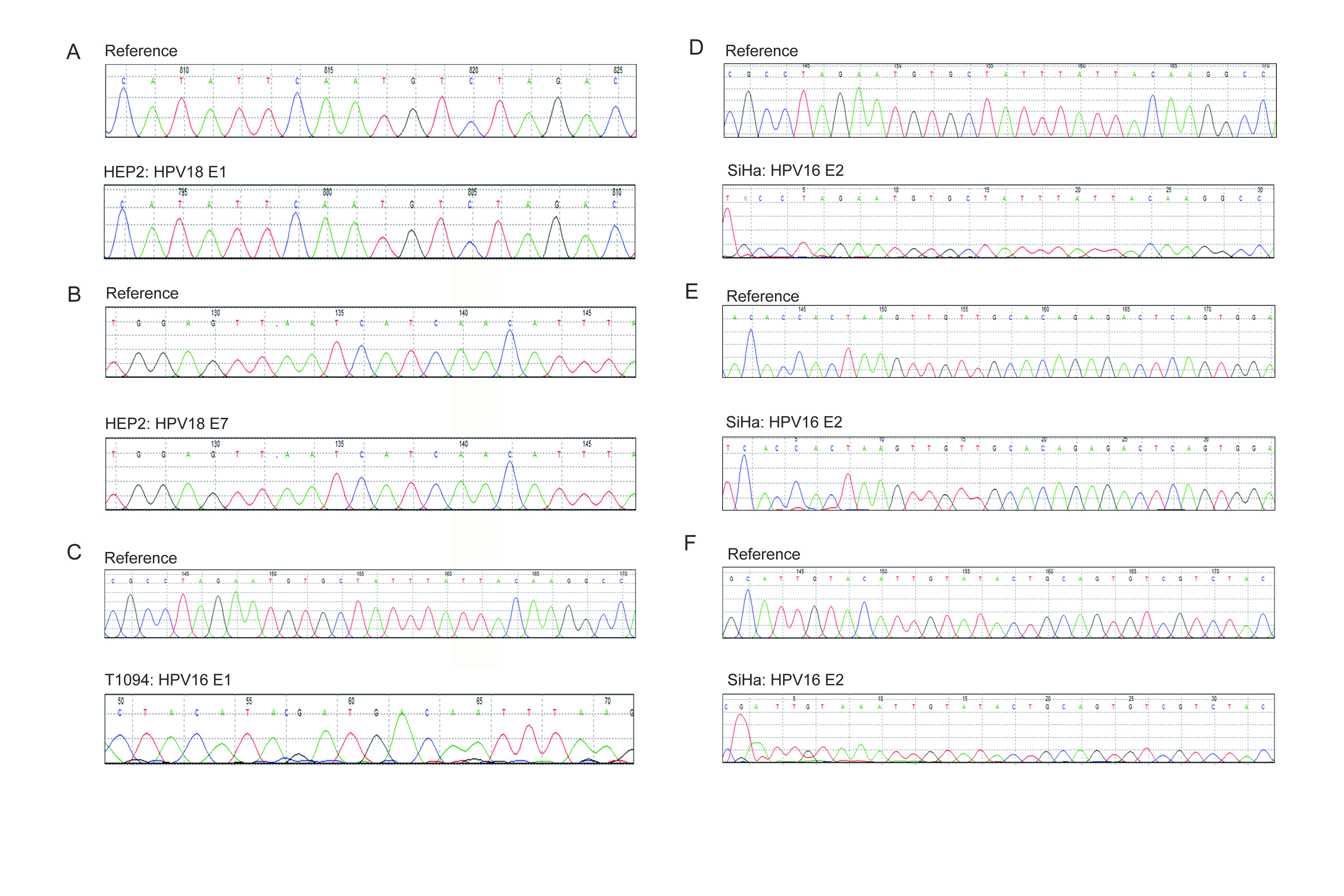
**

**Supplementary Figure 1. Sanger sequencing based validation of HPV integrant**

Presence of HPV was validated by sequencing HPV genes in the cervical and HNSCC cell lines using Sanger sequencing. Sequencing traces were analysed and compared to GenBank reference database using mutation surveyor. Each of the sequence trace is shown with alignment to corresponding reference sequence: (A) E1 gene and (B) E7 gene from cDNA of Hep2 cell line, (C) E1 gene from genomic DNA of cervical tumor sample (D-F) HPV16 E2 reads in SiHa cells.


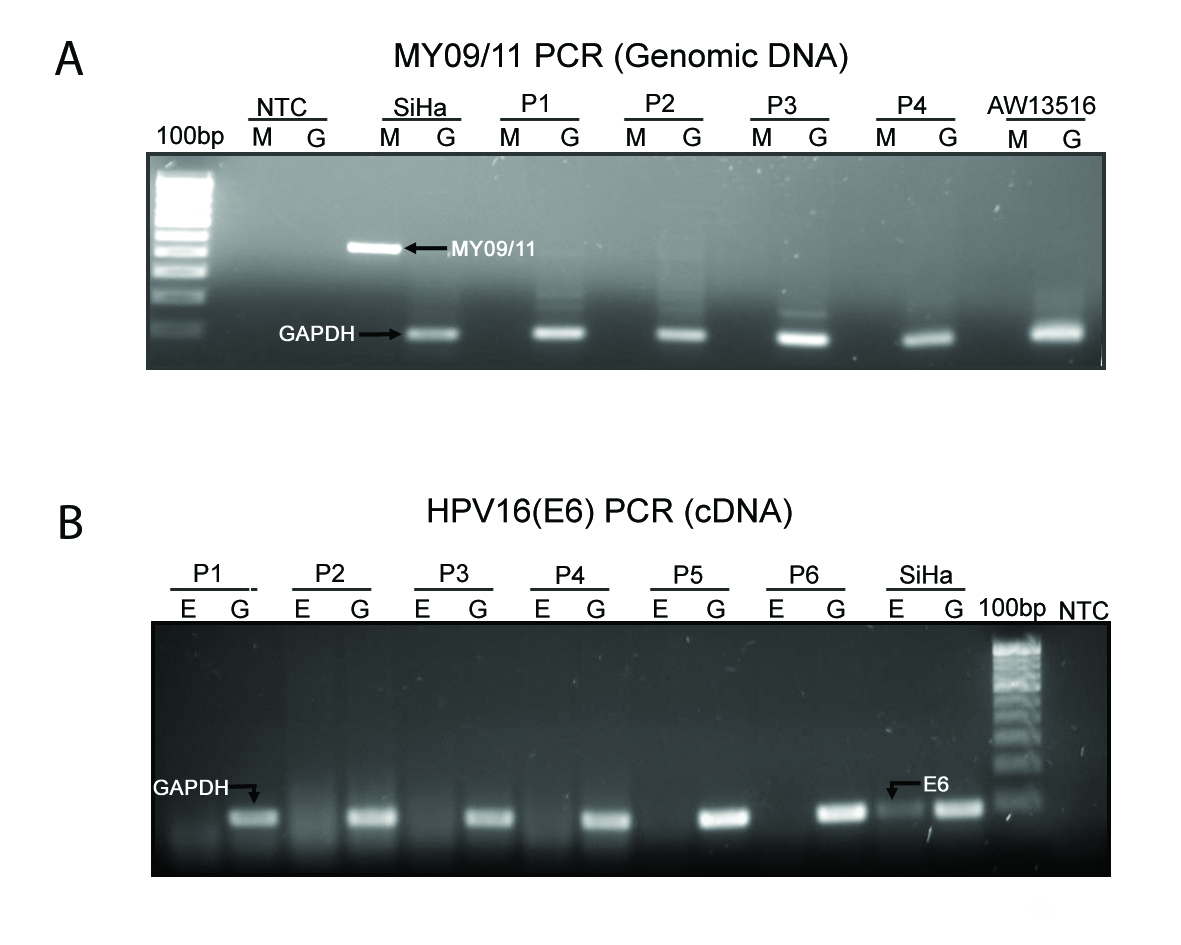


**Supplementary Figure 2. Validation of HPV negative TSCC samples by PCR.**

(A) PCR was performed on genomic DNA using MY09/11 primers. SiHa cell line was used as positive control for HPV and AW1356 cell line as negative control and GAPDH as internal control for genomic DNA. NTC - no template control; M - MY09/11; G - GAPDH; P1 to P4 – Tongue Squamous Cell Carcinoma (TSCC) patient samples. MY09/11 amplifies 450bp and GAPDH 100bp indicated using black arrow.

(B) PCR of E6 gene of HPV16 was performed on cDNA msde form TSCC tumor samples along with SiHa cell line as positive control. P1 to P6 - TSCC patient samples; E - E6 band; G – GAPDH band, NTC - no template control. E6 amplifies 86bp and GAPDH 100bp indicated using black arrow.


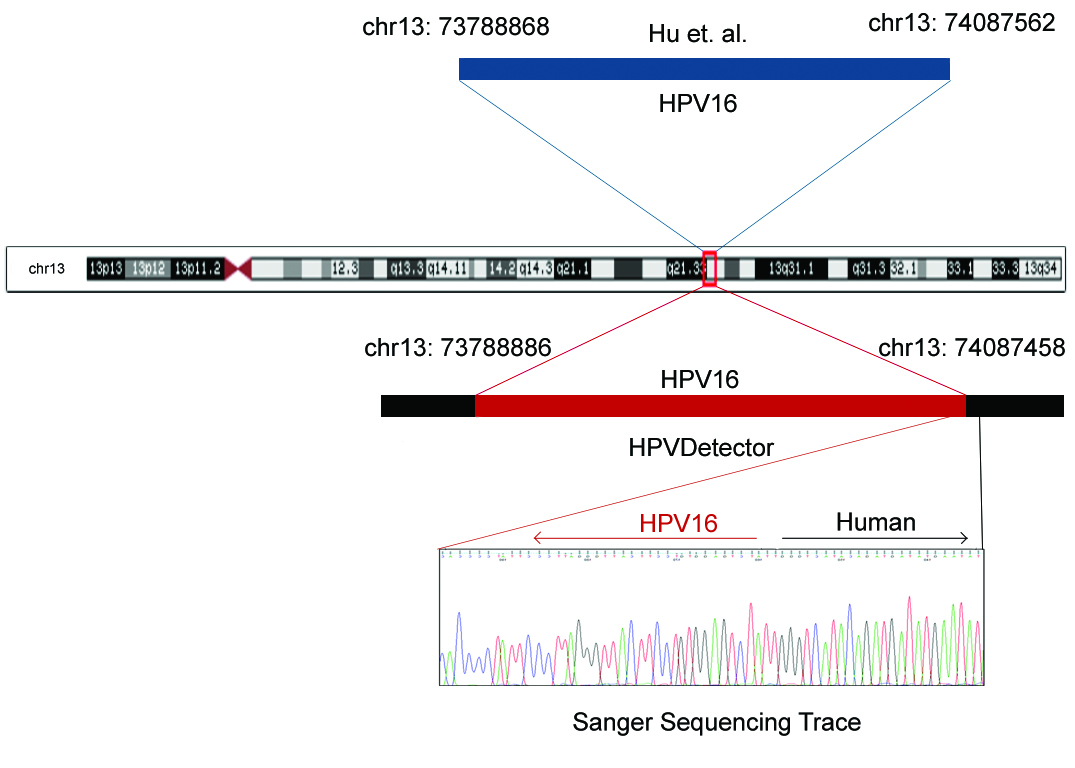


**Supplementary Figure 3. Integration of HPV16 on chr13 of human genome**

HPV16 integration on human chromosome 13 q21.3 near KLF5-KLF12 genes as reported in literature and detected by HPVDetector and Hu at el. 2015. Sanger sequencing trace confirming the junction of HPV16 integration is shown.

**Supplementary Table 1: Human-HPV integration sites detected**

**List of integration sites in 7 Cervical Cancer Exome, SiHa cell line and 1 Head & Neck Transcriptome Samples.**

| **Sample Name & Sequencing Type** | **HPV Type** | **HPV Position** | **HPV Gene** | **Chromosomal Position** | **Human Gene** | **Chromosomal Loci** |
| --- | --- | --- | --- | --- | --- | --- |
| T1099- Exome | HPV16 | 1198 | E1 | 1.45E+08 | PDE4DIP | chr1q21.1 |
|  | HPV16 | 1460 | E1 | 1.48E+08 | NBPF8 | chr1q21.2 |
|  | HPV16 | 3050 | E2 | 1.90E+08 | COL5A2 | chr2q32.2 |
|  | HPV16 | 4998 | L2 | 52548381 | STAB1 | chr3p21.1 |
|  | HPV16 | 3551 | E2 | 1.83E+08 | MCCC1 | chr3q27.1 |
|  | HPV16 | 3551 | E4 | 1.83E+08 | MCCC1 | chr3q27.1 |
|  | HPV16 | 5506 | L2 | 26916036 | CDH9 | chr5p14.1 |
|  | HPV16 | 2140 | E1 | 66453167 | SBDS | chr7q11.21 |
|  | HPV16 | 3555 | E2 | 1.44E+08 | Null | chr7q35 |
|  | HPV16 | 3555 | E4 | 1.44E+08 | Null | chr7q35 |
|  | HPV16 | 1022 | E1 | 1.17E+08 | DFNB31 | chr9q32 |
|  | HPV16 | 6700 | L1 | 71969040 | PPA1 | chr10q22.1 |
|  | HPV16 | 7008 | L1 | 60993893 | PGA4 | chr11q12.2 |
|  | HPV16 | 5283 | L2 | 3431528 | Null | chr11p15.4 |
|  | HPV16 | 2808 | E1 | 48156852 | ITGA3 | chr17q21.33 |
|  | HPV16 | 2808 | E2 | 48156852 | ITGA3 | chr17q21.33 |
|  | HPV16 | 4246 | L2 | 29759114 | AP1B1 | chr22q12.2 |
|  | HPV16 | 4036 | E5 | 39907754 | SMCR7L | chr22q13.1 |
|  | HPV16 | 1047 | E1 | 38580120 | Null | chrXp11.4 |
| T1123- Exome | HPV16 | 6522 | L1 | 53539600 | PODN | chr1p32.3 |
|  | **HPV16** | **4244** | **L2** | **2.36E+08** | **LYST** | **chr1q42.3** |
|  | HPV16 | 5672 | L1 | 1.28E+08 | Null | chr9q33.3 |
|  | HPV16 | 1597 | E1 | 1.06E+08 | Null | chr12q23.3 |
| T755- Exome | HPV16 | 7257 | Null | 33141335 | LINC00486 | chr2p22.3 |
|  | **HPV16** | **4975** | **L2** | **1.41E+08** | **SLC25A36** | **chr3q23** |
|  | HPV16 | 4839 | L2 | 1.32E+08 | Null | chr12q24.33 |
|  | HPV16 | 7525 | Null | 96738327 | Null | chr15q26.2 |
|  | HPV16 | 4861 | L2 | 3024720 | ARSF | chrXp22.33 |
|  | HPV16 | 3542 | E2 | 1.49E+08 | Null | chrXq28 |
|  | HPV16 | 3542 | E4 | 1.49E+08 | Null | chrXq28 |
| T887- Exome | HPV16 | 3059 | E2 | 1.58E+08 | CD5L | chr1q23.1 |
|  | HPV16 | 4271 | L2 | 10421693 | KIF1B | chr1p36.22 |
|  | HPV16 | 2258 | E1 | 13094415 | IQSEC1 | chr3p25.2 |
|  | HPV16 | 1984 | E1 | 68905159 | Null | chr4q13.2 |
|  | HPV16 | 973 | E1 | 1.02E+08 | PAM | chr5q21.1 |
|  | HPV16 | 6087 | L1 | 81217176 | Null | chr13q31.1 |
|  | HPV16 | 7179 | Null | 40383461 | RIT2 | chr18q12.3 |
| T938- Exome | HPV31 | 6629 | L1 | 42041208 | HIVEP3 | chr1p34.2 |
|  | HPV31 | 239 | E6 | 1.09E+08 | RANBP2 | chr2q12.3 |
| T1094- Exome | HPV16 | 2416 | E1 | 1.53E+08 | S100A9 | chr1q21.3 |
|  | HPV16 | 4193 | Null | 1.57E+08 | NR4A2 | chr2q24.1 |
|  | HPV16 | 478 | E6 | 6211560 | MLLT1 | chr19p13.3 |
| T959- Exome | HPV16 | 223 | E6 | 1.58E+08 | Null | chr4q32.1 |
| HEP2- Transcriptome | HPV18 | 429 | E6 | 1.72E+08 | CYBRD1 | chr2q31.1 |
|  | HPV18 | 637 | E7 | 1.75E+08 | SP3 | chr2q31.1 |
|  | HPV18 | 1099 | E1 | 71234417 | FAM135A | chr6q13 |
|  | HPV18 | 57 | Null | 74227485 | EEF1A1 | chr6q13 |
|  | HPV18 | 1482 | E1 | 11908669 | C10orf47 | chr10p14 |
|  | HPV18 | 644 | E7 | 21668931 | GOLT1B | chr12p12.1 |
|  | HPV18 | 2404 | E1 | 38804096 | SMARCE1 | chr17q21.2 |
|  | HPV18 | 129 | E6 | 57280963 | PRR11 | chr17q22 |
|  | HPV18 | 1309 | E1 | 62496367 | DDX5 | chr17q23.3 |
|  | HPV18 | 1945 | E1 | 76968674 | LGALS3BP | chr17q25.3 |
|  | HPV18 | 1719 | E1 | 43716480 | ABCG1 | chr21q22.3 |
|  | HPV18 | 920 | E1 | 23803445 | SAT1 | chrXp22.11 |
| SiHa | HPV16 | 616 | E7 | 60580191 | FHIT | chr3p14.2 |
|  | HPV16 | 2529 | E1 | 60580319 | FHIT | chr3p14.2 |
|  | HPV16 | 2027 | E1 | 57290746 | PRIM2 | chr6p11.2 |
|  | HPV16 | 198 | E6 | 95769188 | DPY19L4 | chr8q22.1 |
|  | **HPV16** | **3694** | **E2** | **73788886** | **Null** | **chr13q22.1** |
|  | **HPV16** | **2777** | **E2** | **74087458** | **Null** | **chr13q22.1** |
|  | HPV16 | 454 | E6 | 100177815 | TM9SF2 | chr13q32.3 |
|  | HPV16 | 298 | E6 | 18597469 | ELL | chr19p13.11 |

**Supplementary Table 2 –** Primer sequences used for validation

| **Primer for** | **Forward primer sequence (5’-3’)** | **Reverse primer sequence (5’-3’)** |
| --- | --- | --- |
| HEP2-HPV E1 | CCCATATTCAATGTCTAGACTG | ACTTAAACCTTTGGCAACTGTT |
| HEP2-HPV E6 | CAGAAACCGTTGAATCCAGC | CAGCACGAATGGCACTGG |
| HEP2-HPV E7 | ATGAAATTCCGGTTGACCTTC | CGGGCTGGTAAATGTTGATG |
| T1094-HPV E1 | GTTAGATGATGCTACAGTGCCCTG | GCTGGAACATCCATAGAAACCAAATT |
| MY09 | CGTCCMARRGGAWACTGATC |  |
| MY11 |  | GCMCAGGGWCATAAYAATGG |
| GP-5 | TTTGTTACTGTGGTAGATAC |  |
| GP-6 |  | ACTAAATGTCAAATAAAAAG |
| HPV16 seq 1 | GCCTAGAATGTGCTATTTATTACAAGG | CGTTAGTTGCAGTTCAATTGCTT |
| HPV16 seq 2 | CACCACTAAGTTGTTGCACAGA | TCACCTTTTAAATGTACTATGGGTGT |
| HPV16 seq 3 | ATTGTAAATTGTATACTGCAGTGTCGT | ACAAAAATTGGTCACGTTGC |
| Human seq 1 | AATCCCAGCTACTCGGGAG | AGAGAGTCTCGCCCTGTCAC |
| Human seq 2 | AAGACGTGGCATCCTCAAAC | GCTGACTAACCCTGGACAGA |
| GAPDH | CAAGGCTGTGGGCAAGGTC | TCCACCACTGACACGTTGG |
| HPV16 E6 | TTACTGCGACGTGAGGTGTA | GGAATCTTTGCTTTTTGTCC |

#### HPV Detector User Guide

#### Pre-requisites, installation, and execution of HPV Detector:

1. **Pre-requisites:**
2. Linux/Unix based Operating System
3. RAM: 6GB or more.
4. Burrows Wheeler Aligner (BWA) (minimum Ver. 0.6.*).
5. Awk scripting language (generally included in Linux/Unix system)
6. Yad for GUI (generally included in Linux/Unix system)
7. **Installation:**

Decompress the HPVDetector_v0.1.tar.gz file to a suitable location.

HPV Detector package bundle zip is composed of following files:

1. HPV Detector programme files.
2. Directory of indexed HPV reference genome.
3. Directory of indexed Human-HPV pseudo reference genome files.
4. Directory of HPV-Human Gene Annotation file.
5. In case YAD is required to be installed:

Command for RHEL/Fedora/Cent OS: sudo yum install yad

Command for Ubuntu: sudo apt-get install yad

1. In case BWA is required to be installed, run following commands:

wget –O bwa-0.6.2.tar.bz2 http://sourceforge.net/projects/bio-bwa/files/bwa-0.6.2.tar.bz2/download

tar -xvf bwa-0.6.2.tar.bz2

cd bwa-0.6.2/

make

1. In case Picard tool is required to be installed, run following commands:

wget -O picard-tools-1.100.zip http://sourceforge.net/projects/picard/files/picard-tools/1.100/picard-tools-1.100.zip/download

unzip picard-tools-1.100.zip

1. **Tools & software’s used while testing HPV Detector:**
2. BWA version 0.6.2-r126
3. Picard Tools version 1.100
4. Sam Tools version 0.1.18 (r982:295)
5. Bio-perl for GenBank data parsing. Ver. 1.006901
6. Linux operating system Fedora 20 (x86_64)
7. Yad 0.25
8. **Execution - GUI:**

HPV Detector can be executed with GUI by running HPVdetector_GUI from Linux/UNIX shell:

> ./HPVdetector_GUI

Or

> bash HPVdetector_GUI

####
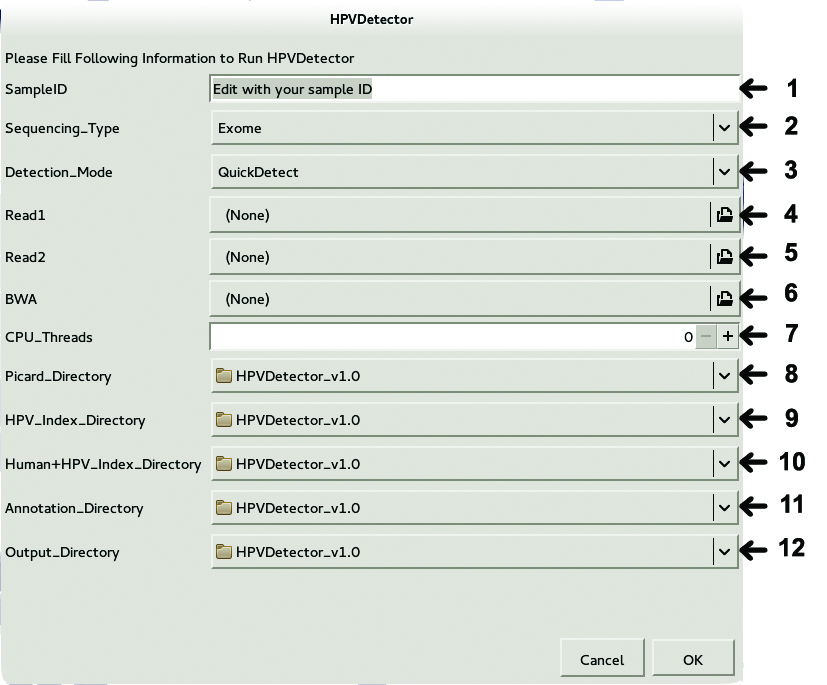


**Supplementary Figure 4. The GUI of HPVDetector**

Upon successful execution, HPVDetector GUI will pop-up with 12 options for user selection. User can easily select required options using point-and-click interface and clicking on OK button will start running HPVDetector.

Step-1: Provide the sample ID

Step-2: Select sequencing type from Exome, Genome or Trenscriptome

Step-3: Select detection mode from QuickDetect or Integration

Step-4: Select input fastq file (forward reads fastq)

Step-5: Select input fastq file (reverse reads fastq)

Step-6: Select BWA executable

Step-7: Set the number of CPU threads as per your system (most modern CPUs have at least two cores/threads)

Step-8: Select directory where Picard Tools are installed

Step-9: Select directory where HPV_index is stored

Step-10: Select directory where Human-HPV_index is stored

Step-11: Select directory where Human-HPV Annotation files are stored


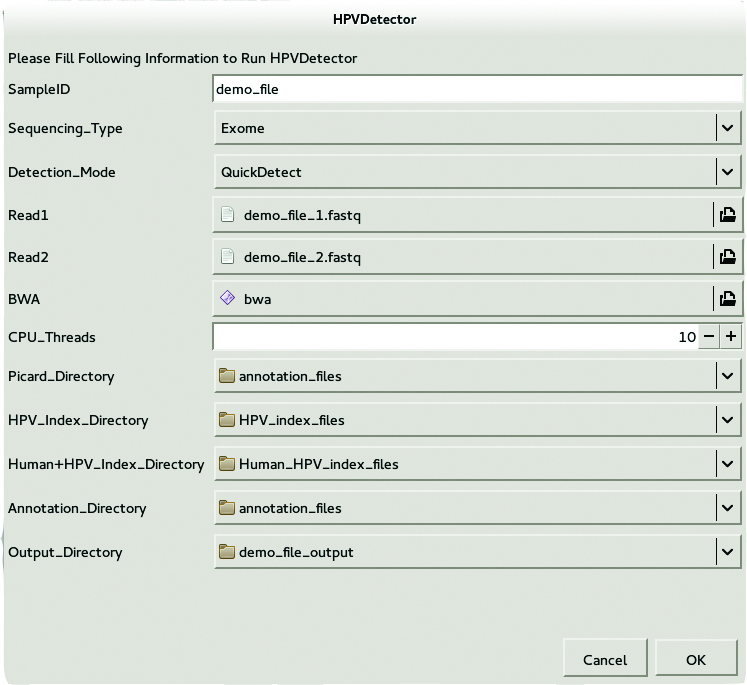
Step-12: Select a directory where output files should be stored

**Supplementary Figure 5. A typical selection of options with supplied supporting files.**

After selecting appropriate options, program can be executed by clinking on OK button. Progress can be monitored on screen and after completion, program will point to respective output files.

1. **Execution – command line:**

HPVDetector can also be executed from command line of Linux/Unix systems. Edit the config.txt file in root directory of HPVDetector to make changes of the variables/path required for the tool. There should be no changes made other than following:

1. mode should be specified as “Integration” or “QuickDetect”

Ex. mode=QuickDetect

1. bwa should be specified as path to the BWA 0.6.2 executable

Ex. bwa=/home/bwa-0.6.2/bwa

1. picard should be specified as path to the Picard tool 1.100 directory

Ex. picard=/home/HPVDetector_v1.0/picard-tools-1.100/

1. hpv_bwa_index should be specified as path to HPV_index_files supplied with the tool

Ex. hpv_bwa_index=/home/ HPVDetector_v1.0/HPV_index_files/HPV_143types_EBVref

1. hpv_human_bwa_index should be specified as path to Human_HPV_index_files supplied with the tool

Ex. hpv_human_bwa_index=/home/HPVDetector_v1.0/Human_HPV_index_files/human_HPV

1. annotation_dir should be specified as path to annotation_files supplied with the tool

Ex. annotation_dir=/home/HPVDetector_v1.0/annotation_files/

1. threads should be specified as whole number (should not exceed the actual number of threads available in the system)

Ex. threads=4

After making above changes, execute HPVDetector as:

./HPVDetector config.txt <read1.fastq> <read2.fastq> <analysis mode> <output path>

<analysis mode> could be WGS, Transcriptome or Exome

Ex.:

./HPVDetector config.txt /siha/read_1.fastq /siha/read_2.fastq WGS /siha_output
